# Global, regional, and national trends in the morbidity gap and contributing diseases, injuries, and risk factors, 1990–2023: a systematic analysis for the Global Burden of Disease Study 2023

**DOI:** 10.1016/S2468-2667(26)00098-8

**Published:** 2026-07-21

**Authors:** Simon I Hay, Paul Nam, Haaris Saqib, Susan A McLaughlin, Catherine Bisignano, Samuel M Ostroff, Marie Ng, Shuhei Nomura, Austin E Schumacher, Christopher J L Murray

**Affiliations:** a Institute for Health Metrics and Evaluation, University of Washington, Seattle, WA, USA; b Department of Health Metrics Sciences, School of Medicine, University of Washington, Seattle, WA, USA; c Jackson School of International Studies, University of Washington, Seattle, WA, USA; d Yong Loo Lin School of Medicine, National University of Singapore, Singapore, Singapore; e Global Health Policy Lab, International Research Institute of Disaster Science, Tohoku University, Miyagi, Japan

## Abstract

**Background:**

As survival improves globally, the central public health challenge increasingly shifts from extending life to extending healthy lifespan. The compression of morbidity hypothesis proposes that effective prevention and care will delay disability more than death, reducing the morbidity gap—ie, the duration or proportion of life spent in poor health. We aimed to comprehensively estimate changes in the morbidity gap globally, regionally, and nationally from 1990 to 2023, by sociodemographic development levels and sex, and analysed leading diseases and injuries and their underlying risk factors contributing to years lived in poor health.

**Methods:**

Using Global Burden of Diseases, Injuries, and Risk Factors Study (GBD) 2023 estimates of life expectancy and healthy life expectancy (HALE) at birth for 204 countries and territories from 1990 to 2023, we calculated the morbidity gap as life expectancy minus HALE, and the proportional morbidity gap as this difference divided by life expectancy. Results are presented globally, by GBD super-region and country, Socio-demographic Index (SDI) quintile, and sex. We decomposed the morbidity gap into cause-specific and risk-specific contributions, assigning to each cause group its proportional share of years lived with disability (YLD), adjusted for comorbidity to avoid double counting disability for co-occurring conditions. We report mean estimates and 95% uncertainty intervals (UIs) derived from 250 or more draws from posterior distributions.

**Findings:**

From 1990 to 2023, the global morbidity gap increased from 8·8 years (95% UI 6·7–11·2) to 10·7 (8·2–13·7), an increase of 1·9 years (1·3–2·6), or 21·9% (16·0–27·9), with point estimates suggesting widening morbidity gaps in 203 of 204 countries and territories. Globally in 2023, an average of 14·5% of life was spent in poor health, compared to 13·6% in 1990. From 1990 to 2023, across locations, age-specific morbidity gaps widened across the adult life course, rather than concentrating in the final years of life. In 2023, morbidity gaps were largest in the high SDI quintile and smallest in the low SDI quintile. Countries with longer life expectancies had larger morbidity gaps and both had a positive relationship with SDI; however, the relationship between life expectancy and the proportional morbidity gap was less clear. A small number of chronic, largely non-fatal causes were the primary contributors to the morbidity gap, with musculoskeletal disorders (especially low back pain), mental disorders (depressive and anxiety disorders), sense organ diseases (age-related hearing loss), unintentional injuries (falls), and other non-communicable diseases together accounting for 57·4% of unhealthy years in 2023, globally. High fasting plasma glucose, high body-mass index, and child and maternal malnutrition were the leading risk factors contributing to the global morbidity gap.

**Interpretation:**

The expansion of the morbidity gap across nearly all locations indicates that improvements in survival have consistently outpaced reductions in non-fatal health loss across geographies, SDI levels, and sexes, with most populations now spending a decade or more of life in poor health across the lifespan. Progress towards healthy ageing should therefore be assessed not only by reduced mortality, but also by declines in years lived in poor health, with emphasis placed on extending healthspan.

**Funding:**

The Gates Foundation.

## Introduction

Over the past century, life expectancy has risen sharply worldwide, driven initially by reductions in infectious, maternal, and child mortality, and later by advances in chronic disease prevention and treatment.^1^ As longer life became more common,^2^, ^3^ attention shifted from simply extending lifespan to extending healthspan, with three contrasting hypotheses for demographic change emerging. Gruenberg^4^ warned of an expansion of morbidity, whereby survival gains would produce more years lived with disease, Fries^5^, ^6^, ^7^ predicted a compression of morbidity, with health-promotion and risk-reduction strategies postponing disability into a shorter period late in life, and Manton^8^ proposed a dynamic equilibrium, whereby chronic conditions would become more prevalent but less disabling. Central to Fries’ hypothesis was the prediction of a reduction in years lived with morbidity; subsequent reinterpretations introduced the concept that unhealthy years would decline only proportionally as longevity increases.^9^, ^10^, ^11^

**Research in context**

**Evidence before this study**

The compression of morbidity hypothesis, first articulated by Fries in 1980, proposed that effective prevention and risk reduction would delay the onset of disability more than death, resulting in fewer absolute years and a smaller proportion of life lived in poor health. To review the literature on this topic, we searched PubMed and Web of Science from Jan 1, 1980 to Oct 1, 2025 using the terms “compression of morbidity”, “morbidity gap”, “healthy life expectancy”, “healthspan”, “life expectancy”, and “Global Burden of Disease”, with no language restrictions. Previous studies, including analyses using earlier iterations of the Global Burden of Diseases, Injuries, and Risk Factors Study (GBD) and regional or national cohorts, reported evidence of relative compression primarily in data before 1980 and in high-income settings, alongside persistent or expanding morbidity in many low-income and middle-income countries; however, over the past decade, studies have consistently found evidence of expanding morbidity worldwide. Few studies have quantified both absolute and relative changes in unhealthy years over the past three decades at a global scale, systematically assessed differences by sociodemographic development and sex, or decomposed the morbidity gap into cause-specific and risk-factor-specific contributors.

**Added value of this study**

This study provides a comprehensive global assessment of the compression of morbidity hypothesis using consistent and comparable estimates of life expectancy and healthy life expectancy from GBD 2023 for 204 countries and territories from 1990 to 2023. We extend previous work by examining trends across all GBD super-regions and Socio-demographic Index (SDI) quintiles, assessing differences by sex, and decomposing the morbidity gap into leading causes and risk factors. We show that expansion of morbidity occurred globally and across nearly all levels of geographical aggregation, with point estimates suggesting increases in 203 of 204 countries and territories. In 2023, musculoskeletal disorders, mental disorders, sense organ diseases, unintentional injuries, and other non-communicable diseases together accounted for more than half of the unhealthy years lived globally. We show that the expansion of morbidity is driven by a consistent core of chronic conditions that are largely non-fatal, though with marked heterogeneity across levels of sociodemographic development. These decompositions likewise identify a substantial share of unhealthy years that are attributable to potentially modifiable risk factors, such as high fasting plasma glucose, high body-mass index, and tobacco use, as well as nutritional contributors driven by child and maternal malnutrition, particularly in lower SDI settings. By providing these comprehensive morbidity gap estimates and their leading contributors, this study offers actionable evidence to inform national, regional, and global health priority setting.

**Implications of all the available evidence**

Taken together, the evidence shows that over the past three decades, gains in survival have not been matched by comparable gains in healthspan. From 1990 to 2023, life expectancy increased more rapidly than healthy life expectancy, resulting in an expansion of the morbidity gap. Morbidity gaps were largest in the highest SDI settings and smallest in the lowest settings, reflecting differences in lifespan rather than healthier ageing. Decomposition of the morbidity gap shows that this expansion is driven largely by non-fatal conditions, underpinned by a set of modifiable risk factors that vary somewhat by geographical and sociodemographic settings. These findings indicate that health-system performance should be judged not only by reductions in mortality, but also by progress in reducing years lived in poor health, and that future gains in healthy ageing will depend on targeted reduction of risk factor exposures, and improved delivery of rehabilitation, mental health, and chronic care services.

These hypotheses have profound implications. At the individual level, they inform healthy lifestyle choices and clinical decisions about screening and prevention.^12^ For health systems, they shape the balance between preventive strategies, curative care, and long-term management of chronic conditions.^13^ At the societal level, they raise questions about the sustainability of social care, particularly as populations age,^14^ and about the allocation of resources between prevention, treatment, and innovation.^15^

Since the publication of Fries’ hypothesis, evidence for compression of morbidity has been mixed, with earlier studies finding signs of relative compression, especially in high-income countries, following improvements in cardiovascular disease prevention.^9^ By contrast, in many low-income and middle-income countries, declines in premature mortality have been accompanied by rising years lived with disability (YLDs) from non-fatal conditions, suggesting morbidity expansion.^1^ Indeed, a preponderance of work finds an expansion of morbidity across populations, particularly when using absolute measures.^16^, ^17^, ^18^, ^19^ Notably, after 2020, the COVID-19 pandemic introduced a major global disruption, reversing gains in both life expectancy and healthy life expectancy (HALE) in many settings^20^ and amplifying existing inequalities.^21^

The Global Burden of Diseases, Injuries, and Risk Factors Study (GBD) 2023 provides the opportunity to reassess the compression of morbidity hypothesis using a comprehensive and internally consistent global time series from 1990 to 2023.^22^, ^23^, ^24^ Here, we use GBD 2023 estimates to evaluate changes in the morbidity gap (life expectancy minus HALE: defined as the number of years lived in poor health) and in the proportional morbidity gap (morbidity gap divided by life expectancy: defined as the proportion of life expectancy spent in poor health). The comprehensive nature of GBD 2023 affords us the opportunity to examine morbidity gap trends by geography, Socio-demographic Index (SDI) level,^22^ and sex, to identify leading disease, injury, and risk factor contributors to the morbidity gap, and to examine disruptions associated with the COVID-19 pandemic.^20^ Our findings provide actionable evidence for governments, health systems, and researchers, helping to guide national prioritisation and prompting a re-evaluation of how population health progress is defined in the era after Sustainable Development Goals (SDGs) expire in 2030, by identifying where targeted interventions, preventive policies, and scientific innovation are most likely to yield gains in aligning healthspan with lifespan.^1^

## Methods

### Overview

We used data from GBD 2023 to analyse global, regional, and national trends in life expectancy, HALE, and the morbidity gap from 1990 to 2023.^22^, ^23^, ^24^ We examined absolute and relative changes, by SDI quintile and sex, and decomposed these changes by cause and risk factor. Analyses were completed with Python version 3.10.4, Stata version 13.1, and R version 4.2.1. The statistical code used for data processing and analysis is available upon request (engage@healthdata.org)**.** GBD 2023 complies with the GATHER statement.^25^

### Study design and data sources

GBD 2023 provides annual estimates of mortality, morbidity, and population health for 204 countries and territories, with full methodological detail in published GBD 2023 capstone papers and their appendices.^22^, ^23^, ^24^ In brief, data inputs included vital registration systems, censuses, household surveys, sample registration systems, and disease registries. Where primary data were incomplete, standard GBD modelling frameworks were applied, including the Cause of Death Ensemble model (CODEm),^23^ spatiotemporal Gaussian process regression (ST-GPR),^22^, ^23^, ^24^ and disease modelling meta-regression (DisMod-MR) for non-fatal outcomes.^24^, ^26^ Population denominators were estimated using GBD's demographic models to ensure consistency across locations and years.^22^

### Outcome definitions

Life expectancy was defined as the average number of years a person can expect to live from birth, calculated from age-specific mortality rates.^22^ Life expectancy calculations assume that mortality at the time of birth remains constant. Age-specific survival probabilities were derived from the period life table implied by GBD 2023 age-specific all-cause mortality rates, with survival at age x computed as the cumulative product of 1 minus the probability of death across ages 0 to x, representing the proportion of a synthetic birth cohort expected to be alive at each age under the mortality conditions of the specified year. HALE was defined as expected years of life at birth lived in full health, calculated by adjusting life expectancy for disability using YLDs^22^ and disability weights derived from population surveys.^27^ The morbidity gap was defined as the absolute difference between life expectancy and HALE. The proportion of life expectancy spent in poor health ([life expectancy – HALE] / life expectancy) is also referred to as the proportional morbidity gap. We defined the COVID-19 pandemic period as calendar years 2020–22, which are shaded in figures to highlight disruption to trends.

### Analytical methods

We estimated the morbidity gap globally, for seven super-regions and 204 countries and territories, by SDI quintiles, and by sex using GBD 2023 estimates of life expectancy and HALE.^22^, ^24^ Time series were constructed for 1990–2023, and population-weighted means were reported. We decomposed the morbidity gap into cause-specific contributions by assigning to each cause group its proportional share of YLDs using GBD 2023 YLD estimates, which are adjusted for the co-occurrence of non-fatal causes within an individual.^24^ To do so, we multiplied person-years lived between age x and x + n by YLD rates for all GBD Level 2 causes, which produced estimates of the number of years of the morbidity gap each cause contributed by cause, location, and year. The same methods were applied to decompose the morbidity gap into GBD Level 3 causes (more specific causes disaggregated from Level 2 cause groups) and risk factor-specific contributions.

All estimates were generated using 250 or more draws from the posterior distributions of the relevant models.^24^ We report the mean of these draws as the point estimate and the 2·5th to 97·5th percentiles as 95% uncertainty intervals (UIs). In figures, UIs are represented as shaded ribbons, and in tables, as ranges or appended values where appropriate.

### Role of the funding source

The funder of the study had no role in study design, data analysis, data interpretation, or writing of this report.

## Results

Between 1990 and 2023, global life expectancy at birth increased from 64·6 years (95% UI 64·5–64·8) to 73·8 (73·6–74·1), and HALE rose from 55·9 years (53·5–57·9) to 63·1 (60·2–65·6; appendix 1 pp 4, 10–17). These increases resulted in an expansion in the morbidity gap from 8·8 years (6·7–11·2) in 1990 to 10·7 (8·2–13·7) in 2023 (figure 1; table), an increase of 1·9 years (1·3–2·6) or 21·9% (16·0–27·9). These findings indicate more years lived in poor health in 2023 than in 1990. Globally in 2023, an average of 14·5% of life was spent in poor health, compared with 13·6% in 1990. Between 1990 and 2023, increases in life expectancy were larger than increases in HALE in all but one country or territory (appendix 1 pp 5, 10–17), equating to a nearly universal increase in the morbidity gap (table). The COVID-19 pandemic temporarily disrupted these trends. Compared with levels before the pandemic in 2020, global life expectancy declined to 71·9 years (71·9–72·0) in 2021 and HALE declined to 61·7 years (58·8–64·0), with little net change in the global morbidity gap, which decreased slightly to 10·3 years (7·9–13·1; figure 1, table; appendix 1 pp 4, 10–17).

**Figure 1:**
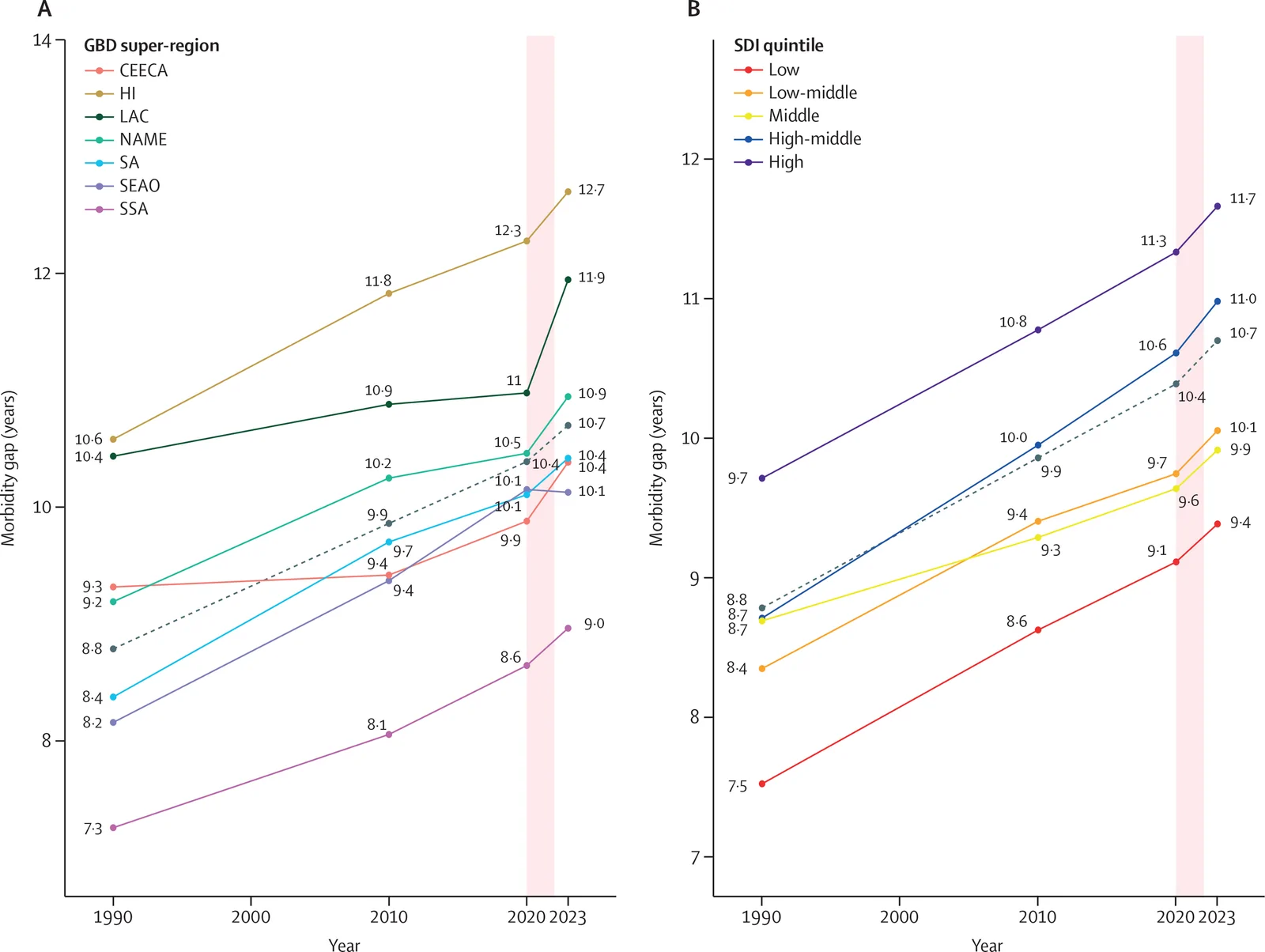
Morbidity gap by GBD super-region (A) and SDI quintile (B), 1990–2023 Pink vertical shading indicates the COVID-19 pandemic period (2020–22). The dashed line in each panel represents the global average. CEECA=central Europe, eastern Europe, and central Asia. GBD=Global Burden of Diseases, Injuries, and Risk Factors Study. HI=high income. LAC=Latin America and Caribbean. NAME=north Africa and Middle East. SA=south Asia. SDI=Socio-demographic Index. SEAO= southeast Asia, east Asia and Oceania. SSA=sub-Saharan Africa.

**Table:** The morbidity gap in 1990, 2010, 2020, 2021, 2022, and 2023, and absolute and relative changes for 1990–2023, globally, by SDI quintile, and for GBD super-regions and countries and territories

	**1990**	**2010**	**2020**	**2021**	**2022**	**2023**	**Rank (2023)**	**Absolute change, years (1990–2023)**	**Relative change, % (1990–2023)**
**Global**	**8·8 (6·7 to 11·2)**	**9·9 (7·5 to 12·5)**	**10·4 (8·0 to 13·3)**	**10·3 (7·9 to 13·1)**	**10·6 (8·1 to 13·6)**	**10·7 (8·2 to 13·7)**	..	**1·9 (1·3 to 2·6)**	**21·9 (16·0 to 27·9)**
**Central Europe, Eastern Europe, and Central Asia**	**9·3 (7·1 to 11·9)**	**9·4 (7·2 to 12·0)**	**9·9 (7·6 to 12·8)**	**9·8 (7·5 to 12·6)**	**10·2 (7·7 to 13·2)**	**10·4 (7·9 to 13·4)**	..	**1·1 (0·5 to 1·6)**	**11·5 (6·4 to 17·0)**
Albania	9·5 (7·2 to 12·3)	10·4 (7·8 to 13·5)	10·5 (8·0 to 13·6)	10·7 (8·2 to 13·8)	11·1 (8·5 to 14·2)	11·1 (8·4 to 14·2)	74	1·5 (0·5 to 2·5)	16·2 (5·6 to 27·3)
Armenia	9·3 (7·0 to 11·9)	9·6 (7·3 to 12·3)	9·8 (7·4 to 12·5)	10·7 (8·1 to 13·6)	11·2 (8·4 to 14·2)	11·5 (8·7 to 14·8)	49	2·2 (1·2 to 3·4)	24·3 (13·5 to 37·2)
Azerbaijan	8·6 (6·4 to 11·1)	9·1 (6·9 to 11·6)	9·1 (6·9 to 11·6)	9·4 (7·1 to 12·0)	9·9 (7·5 to 12·6)	9·8 (7·2 to 12·5)	128	1·2 (0·4 to 2·2)	14·6 (4·3 to 26·3)
Belarus	9·3 (7·0 to 11·9)	9·2 (7·0 to 11·8)	9·9 (7·5 to 12·9)	10·1 (7·6 to 12·9)	10·0 (7·5 to 12·9)	10·1 (7·6 to 13·0)	117	0·8 (0·1 to 1·6)	9·2 (1·5 to 17·2)
Bosnia and Herzegovina	9·5 (7·3 to 12·3)	10·3 (7·9 to 13·3)	10·6 (8·2 to 13·7)	10·5 (8·2 to 13·5)	11·0 (8·5 to 14·1)	11·2 (8·4 to 14·4)	66	1·6 (0·7 to 2·6)	17·4 (8·0 to 27·1)
Bulgaria	9·2 (7·0 to 11·8)	9·6 (7·3 to 12·4)	9·8 (7·5 to 12·7)	9·6 (7·3 to 12·5)	10·1 (7·7 to 13·1)	10·3 (7·8 to 13·4)	107	1·1 (0·6 to 1·8)	12·3 (6·5 to 18·6)
Croatia	9·4 (7·2 to 12·0)	10·2 (7·8 to 13·2)	10·9 (8·3 to 13·8)	10·9 (8·4 to 14·1)	11·0 (8·4 to 14·2)	11·3 (8·7 to 14·4)	58	1·9 (1·2 to 2·6)	20·0 (13·8 to 26·8)
Czechia	9·6 (7·3 to 12·3)	11·0 (8·4 to 14·1)	11·3 (8·7 to 14·5)	11·3 (8·6 to 14·4)	11·6 (8·9 to 14·9)	11·8 (9·0 to 15·0)	39	2·2 (1·6 to 3·0)	22·8 (17·4 to 28·4)
Estonia	9·2 (7·0 to 11·8)	10·3 (7·8 to 13·2)	11·1 (8·5 to 14·1)	10·8 (8·3 to 13·9)	11·1 (8·4 to 14·3)	11·3 (8·6 to 14·5)	56	2·1 (1·4 to 2·9)	22·7 (16·0 to 29·7)
Georgia	8·7 (6·6 to 11·4)	9·3 (7·0 to 12·1)	10·0 (7·6 to 12·9)	9·8 (7·3 to 12·6)	10·3 (7·7 to 13·2)	10·4 (7·7 to 13·6)	101	1·7 (0·7 to 2·7)	19·6 (8·3 to 31·3)
Hungary	9·5 (7·2 to 12·2)	10·1 (7·6 to 12·9)	10·3 (8·0 to 13·3)	10·1 (7·8 to 13·2)	10·6 (8·1 to 13·7)	10·8 (8·2 to 13·9)	87	1·3 (0·8 to 2·0)	14·1 (8·8 to 20·0)
Kazakhstan	9·0 (6·9 to 11·5)	8·9 (6·8 to 11·4)	9·4 (7·1 to 12·1)	9·2 (7·1 to 11·9)	10·1 (7·7 to 12·9)	10·3 (7·7 to 13·2)	109	1·3 (0·5 to 2·1)	14·2 (5·8 to 23·2)
Kyrgyzstan	8·8 (6·6 to 11·3)	8·8 (6·6 to 11·4)	9·2 (7·0 to 11·9)	9·7 (7·4 to 12·5)	10·1 (7·6 to 13·2)	10·1 (7·6 to 13·1)	118	1·3 (0·4 to 2·3)	15·3 (4·5 to 27·7)
Latvia	9·5 (7·2 to 12·1)	9·7 (7·3 to 12·4)	10·3 (7·8 to 13·3)	9·9 (7·6 to 12·8)	10·3 (7·8 to 13·2)	10·5 (8·0 to 13·7)	95	1·1 (0·5 to 1·8)	11·5 (5·0 to 18·8)
Lithuania	9·7 (7·3 to 12·3)	9·9 (7·5 to 12·7)	10·5 (8·0 to 13·6)	10·4 (7·9 to 13·5)	10·7 (8·1 to 13·8)	11·0 (8·4 to 14·0)	77	1·4 (0·7 to 2·1)	14·1 (7·5 to 20·6)
Moldova	8·9 (6·6 to 11·5)	9·0 (6·7 to 11·7)	9·8 (7·4 to 12·6)	9·7 (7·4 to 12·4)	10·4 (7·9 to 13·5)	10·5 (7·9 to 13·6)	98	1·6 (0·7 to 2·6)	18·6 (8·7 to 30·4)
Mongolia	7·6 (5·6 to 10·0)	8·1 (6·1 to 10·5)	9·3 (7·0 to 11·9)	8·9 (6·8 to 11·7)	9·5 (7·0 to 12·3)	9·7 (7·0 to 12·6)	133	2·1 (0·8 to 3·4)	28·5 (12·3 to 46·0)
Montenegro	9·2 (7·0 to 11·8)	9·7 (7·4 to 12·5)	10·0 (7·6 to 13·0)	9·8 (7·5 to 12·6)	10·6 (8·2 to 13·7)	10·7 (8·1 to 13·8)	89	1·5 (0·8 to 2·3)	16·5 (9·7 to 23·4)
North Macedonia	9·0 (6·9 to 11·5)	9·6 (7·3 to 12·4)	10·0 (7·7 to 12·8)	10·0 (7·6 to 12·7)	10·5 (8·0 to 13·4)	10·6 (8·0 to 13·5)	93	1·6 (0·8 to 2·4)	17·9 (9·7 to 26·1)
Poland	9·7 (7·3 to 12·5)	10·4 (7·9 to 13·3)	10·7 (8·2 to 13·8)	10·5 (8·1 to 13·7)	11·1 (8·5 to 14·3)	11·3 (8·6 to 14·6)	55	1·7 (1·1 to 2·3)	17·1 (11·6 to 22·1)
Romania	9·2 (7·0 to 11·8)	9·4 (7·1 to 12·1)	9·6 (7·4 to 12·5)	9·5 (7·3 to 12·3)	10·0 (7·6 to 13·0)	10·2 (7·8 to 13·2)	111	1·0 (0·5 to 1·6)	11·0 (5·4 to 16·5)
Russia	9·4 (7·1 to 12·1)	9·3 (7·0 to 11·9)	9·9 (7·5 to 12·9)	9·8 (7·5 to 12·6)	10·4 (7·8 to 13·4)	10·4 (7·8 to 13·4)	105	0·9 (0·0 to 1·7)	9·7 (0·1 to 18·1)
Serbia	8·5 (6·4 to 10·9)	9·2 (7·1 to 11·9)	9·5 (7·3 to 12·2)	9·4 (7·2 to 12·1)	9·9 (7·5 to 12·7)	10·0 (7·4 to 12·8)	121	1·5 (0·6 to 2·5)	17·7 (7·0 to 29·6)
Slovakia	9·3 (7·1 to 11·9)	10·1 (7·7 to 13·1)	10·5 (8·0 to 13·5)	10·0 (7·6 to 13·0)	10·6 (8·1 to 13·7)	10·9 (8·4 to 14·0)	80	1·7 (1·0 to 2·4)	18·1 (11·7 to 24·7)
Slovenia	9·8 (7·4 to 12·6)	11·3 (8·6 to 14·4)	11·6 (8·9 to 14·9)	11·8 (9·1 to 15·1)	12·1 (9·2 to 15·4)	12·3 (9·4 to 15·7)	20	2·5 (1·7 to 3·3)	25·3 (19·9 to 31·3)
Tajikistan	8·5 (6·3 to 11·2)	9·1 (6·8 to 11·7)	9·3 (7·1 to 11·9)	9·2 (6·9 to 11·9)	9·3 (6·9 to 12·1)	9·2 (6·6 to 12·2)	156	0·7 (−0·5 to 2·1)	8·8 (−6·2 to 25·2)
Turkmenistan	8·3 (6·3 to 10·8)	8·7 (6·4 to 11·1)	8·8 (6·5 to 11·4)	8·8 (6·5 to 11·6)	9·2 (6·6 to 12·0)	9·2 (6·6 to 12·2)	158	0·9 (−0·3 to 2·2)	10·4 (−4·2 to 26·6)
Ukraine	9·7 (7·4 to 12·3)	9·3 (7·0 to 11·8)	9·9 (7·5 to 12·7)	9·6 (7·4 to 12·3)	9·5 (5·5 to 13·4)	10·3 (7·7 to 13·4)	108	0·6 (−0·3 to 1·5)	6·2 (−2·9 to 15·9)
Uzbekistan	8·7 (6·5 to 11·3)	8·6 (6·5 to 11·1)	9·0 (6·9 to 11·8)	9·4 (7·1 to 12·1)	9·3 (7·0 to 12·1)	9·2 (6·9 to 11·9)	159	0·5 (−0·2 to 1·2)	5·7 (−2·1 to 15·0)
**High income**	**10·6 (8·1 to 13·3)**	**11·8 (9·0 to 14·9)**	**12·3 (9·4 to 15·5)**	**12·3 (9·5 to 15·6)**	**12·5 (9·6 to 15·8)**	**12·7 (9·8 to 16·1)**	..	**2·1 (1·6 to 2·7)**	**20·1 (16·7 to 23·7)**
Andorra	10·5 (7·9 to 13·4)	11·5 (8·8 to 14·8)	11·9 (9·0 to 15·0)	12·3 (9·2 to 15·6)	12·2 (9·1 to 15·4)	12·3 (9·3 to 15·6)	22	1·8 (0·9 to 2·7)	17·1 (9·3 to 25·6)
Argentina	9·4 (7·1 to 12·0)	10·0 (7·6 to 12·8)	10·4 (8·0 to 13·4)	10·1 (7·7 to 13·0)	10·6 (8·1 to 13·6)	10·9 (8·3 to 13·9)	84	1·5 (0·9 to 2·3)	16·5 (10·2 to 22·5)
Australia	11·0 (8·3 to 13·9)	12·9 (9·9 to 16·4)	13·7 (10·5 to 17·3)	13·7 (10·5 to 17·3)	13·6 (10·4 to 17·2)	13·9 (10·6 to 17·5)	2	2·9 (2·1 to 3·8)	26·9 (22·7 to 31·6)
Austria	9·9 (7·5 to 12·7)	11·2 (8·6 to 14·3)	11·6 (9·0 to 15·1)	11·7 (9·0 to 15·2)	11·8 (9·1 to 15·2)	11·9 (9·2 to 15·3)	33	2·1 (1·5 to 2·8)	21·1 (16·1 to 25·4)
Belgium	9·9 (7·5 to 12·6)	11·4 (8·7 to 14·5)	11·7 (8·9 to 15·0)	12·2 (9·3 to 15·6)	12·3 (9·3 to 15·7)	12·3 (9·4 to 15·8)	21	2·4 (1·7 to 3·1)	24·2 (19·1 to 28·6)
Brunei	8·7 (6·6 to 11·2)	9·5 (7·1 to 12·3)	10·0 (7·6 to 12·9)	10·0 (7·6 to 12·8)	9·9 (7·6 to 12·7)	10·2 (7·7 to 13·1)	115	1·4 (0·6 to 2·2)	16·2 (7·4 to 25·8)
Canada	11·4 (8·6 to 14·5)	12·9 (9·9 to 16·3)	13·4 (10·3 to 16·8)	13·5 (10·4 to 16·9)	13·4 (10·3 to 16·8)	13·7 (10·5 to 17·2)	3	2·3 (1·6 to 3·0)	20·3 (14·6 to 25·3)
Chile	9·9 (7·5 to 12·7)	10·9 (8·2 to 13·8)	11·4 (8·6 to 14·5)	11·3 (8·6 to 14·4)	11·6 (8·8 to 14·8)	12·0 (9·1 to 15·3)	30	2·2 (1·5 to 2·9)	22·0 (16·7 to 28·2)
Cyprus	9·2 (6·9 to 12·2)	10·2 (7·9 to 13·2)	10·8 (8·2 to 14·0)	10·8 (8·2 to 13·9)	11·0 (8·1 to 14·1)	11·2 (8·4 to 14·4)	63	2·0 (0·7 to 3·2)	21·8 (8·5 to 36·8)
Denmark	9·8 (7·4 to 12·6)	10·7 (8·2 to 13·7)	11·5 (8·8 to 14·6)	11·5 (8·8 to 14·7)	11·5 (8·8 to 14·8)	11·7 (8·9 to 14·9)	46	1·9 (1·3 to 2·6)	19·6 (14·9 to 24·6)
Finland	9·9 (7·5 to 12·6)	11·5 (8·8 to 14·6)	11·8 (9·0 to 15·0)	11·9 (9·1 to 15·1)	11·7 (9·0 to 14·9)	12·0 (9·1 to 15·2)	32	2·1 (1·5 to 2·8)	21·6 (16·4 to 26·6)
France	10·3 (7·8 to 13·1)	11·6 (8·8 to 14·6)	12·2 (9·3 to 15·6)	12·4 (9·5 to 16·0)	12·4 (9·5 to 16·0)	12·5 (9·5 to 16·0)	13	2·2 (1·6 to 2·9)	21·7 (17·2 to 26·4)
Germany	10·1 (7·6 to 12·8)	11·5 (8·8 to 14·9)	11·9 (9·1 to 15·1)	11·8 (9·0 to 15·0)	11·7 (9·0 to 14·8)	11·9 (9·1 to 15·3)	34	1·8 (1·2 to 2·5)	18·1 (13·3 to 23·2)
Greece	10·0 (7·6 to 12·6)	11·2 (8·5 to 14·1)	11·7 (9·0 to 14·9)	11·5 (8·8 to 14·8)	11·6 (8·9 to 15·0)	11·9 (9·1 to 15·2)	36	1·9 (1·2 to 2·5)	18·5 (13·7 to 23·5)
Greenland	8·4 (6·3 to 10·8)	9·6 (7·3 to 12·2)	10·1 (7·6 to 13·1)	10·2 (7·7 to 13·1)	10·5 (7·9 to 13·4)	10·8 (8·2 to 13·8)	88	2·4 (1·3 to 3·3)	28·2 (16·3 to 39·6)
Iceland	10·2 (7·8 to 13·0)	11·5 (8·7 to 14·6)	12·2 (9·3 to 15·3)	12·3 (9·3 to 15·4)	12·2 (9·2 to 15·4)	12·3 (9·3 to 15·4)	23	2·0 (1·4 to 2·7)	20·1 (14·4 to 26·1)
Ireland	9·4 (7·1 to 12·1)	11·0 (8·4 to 14·0)	11·8 (9·0 to 15·4)	12·0 (9·2 to 15·6)	12·1 (9·2 to 15·6)	12·2 (9·3 to 15·8)	24	2·8 (2·0 to 3·7)	30·1 (24·2 to 36·4)
Israel	9·8 (7·5 to 12·6)	11·1 (8·4 to 14·3)	11·5 (8·8 to 14·8)	11·5 (8·8 to 14·9)	11·6 (8·7 to 14·9)	11·7 (8·9 to 15·2)	43	2·0 (1·3 to 2·7)	20·2 (14·7 to 26·4)
Italy	10·0 (7·6 to 12·8)	10·9 (8·3 to 13·9)	11·2 (8·6 to 14·5)	11·4 (8·7 to 14·7)	11·4 (8·7 to 14·6)	11·8 (9·0 to 15·0)	42	1·8 (1·2 to 2·4)	17·6 (13·5 to 21·5)
Japan	10·2 (7·7 to 13·1)	11·3 (8·7 to 14·6)	11·8 (9·0 to 15·0)	11·8 (9·1 to 15·0)	11·7 (9·0 to 15·0)	11·8 (9·0 to 15·1)	40	1·5 (1·1 to 2·1)	15·1 (11·6 to 18·7)
Luxembourg	9·7 (7·3 to 12·3)	11·0 (8·4 to 14·2)	11·7 (8·9 to 15·0)	11·9 (9·0 to 15·3)	12·0 (9·1 to 15·4)	12·1 (9·1 to 15·6)	29	2·4 (1·6 to 3·3)	24·8 (18·0 to 31·9)
Malta	9·4 (7·1 to 12·2)	10·6 (8·1 to 13·7)	11·3 (8·7 to 14·4)	11·5 (8·7 to 14·6)	11·5 (8·7 to 14·7)	11·8 (9·0 to 15·1)	41	2·4 (1·6 to 3·3)	25·7 (18·9 to 34·0)
Monaco	9·5 (7·1 to 12·2)	10·0 (7·5 to 13·1)	10·9 (8·2 to 13·9)	11·0 (8·2 to 14·1)	11·2 (8·2 to 14·4)	11·3 (8·5 to 14·7)	57	1·9 (0·6 to 3·2)	20·0 (6·6 to 35·3)
Netherlands	10·2 (7·8 to 13·0)	13·2 (10·1 to 16·7)	12·3 (9·4 to 15·7)	12·5 (9·6 to 15·9)	12·7 (9·7 to 16·2)	12·9 (9·9 to 16·4)	5	2·8 (2·0 to 3·6)	27·2 (22·5 to 31·8)
New Zealand	11·3 (8·6 to 14·6)	12·5 (9·6 to 16·0)	13·1 (10·1 to 16·6)	13·1 (10·0 to 16·5)	12·9 (9·8 to 16·3)	13·1 (10·0 to 16·7)	4	1·7 (1·2 to 2·3)	15·5 (11·4 to 19·4)
Norway	10·3 (7·8 to 13·1)	11·4 (8·7 to 14·5)	12·1 (9·3 to 15·5)	12·2 (9·4 to 15·6)	12·2 (9·3 to 15·5)	12·4 (9·5 to 15·8)	17	2·2 (1·6 to 2·8)	21·2 (16·7 to 25·5)
Portugal	9·7 (7·4 to 12·5)	11·1 (8·4 to 14·2)	11·7 (8·9 to 15·1)	11·7 (9·0 to 15·2)	11·8 (9·0 to 15·2)	12·1 (9·2 to 15·6)	27	2·4 (1·6 to 3·2)	24·2 (17·6 to 30·3)
San Marino	10·3 (7·7 to 13·1)	11·9 (9·0 to 15·2)	12·3 (9·3 to 15·7)	12·8 (9·6 to 16·3)	12·5 (9·5 to 15·9)	12·9 (9·8 to 16·4)	6	2·6 (1·3 to 4·0)	25·8 (13·8 to 41·6)
Singapore	8·9 (6·8 to 11·5)	10·3 (7·8 to 13·2)	11·2 (8·5 to 14·3)	11·1 (8·5 to 14·1)	11·0 (8·4 to 14·0)	11·2 (8·5 to 14·3)	62	2·4 (1·6 to 3·1)	26·6 (19·8 to 33·3)
South Korea	9·4 (7·1 to 12·2)	10·9 (8·3 to 13·9)	11·5 (8·8 to 14·6)	11·5 (8·8 to 14·6)	11·3 (8·6 to 14·3)	11·6 (8·8 to 14·7)	48	2·2 (1·5 to 3·0)	23·6 (16·3 to 30·4)
Spain	10·2 (7·8 to 13·0)	11·1 (8·5 to 14·2)	11·5 (8·9 to 14·7)	11·9 (9·1 to 15·2)	11·9 (9·1 to 15·1)	12·1 (9·3 to 15·3)	28	1·9 (1·2 to 2·6)	18·2 (13·4 to 23·0)
Sweden	9·7 (7·3 to 12·4)	10·9 (8·3 to 13·9)	11·9 (9·1 to 15·2)	12·2 (9·4 to 15·6)	12·3 (9·4 to 15·6)	12·4 (9·6 to 15·8)	18	2·8 (2·0 to 3·5)	28·5 (23·2 to 33·8)
Switzerland	10·6 (8·0 to 13·5)	11·8 (9·0 to 15·0)	12·3 (9·4 to 15·6)	12·5 (9·6 to 16·0)	12·7 (9·6 to 16·2)	12·8 (9·8 to 16·5)	7	2·2 (1·6 to 3·0)	21·0 (16·2 to 25·6)
UK	10·0 (7·6 to 12·6)	11·6 (8·8 to 14·8)	12·2 (9·3 to 15·5)	12·5 (9·5 to 15·8)	12·7 (9·7 to 16·1)	12·7 (9·7 to 16·1)	9	2·7 (2·0 to 3·5)	27·3 (23·8 to 31·4)
USA	11·9 (9·2 to 15·0)	13·0 (10·0 to 16·2)	13·2 (10·3 to 16·5)	13·2 (10·3 to 16·5)	13·6 (10·5 to 17·0)	14·0 (10·8 to 17·5)	1	2·0 (1·5 to 2·5)	17·1 (13·4 to 20·7)
Uruguay	9·5 (7·1 to 12·2)	10·2 (7·8 to 13·2)	10·8 (8·3 to 13·9)	10·2 (7·8 to 13·1)	10·6 (8·1 to 13·5)	10·9 (8·3 to 14·0)	81	1·5 (0·8 to 2·1)	15·6 (8·8 to 22·1)
**Latin America and Caribbean**	**10·4 (7·9 to 13·4)**	**10·9 (8·3 to 13·9)**	**11·0 (8·4 to 14·1)**	**10·8 (8·3 to 13·8)**	**11·8 (9·0 to 15·1)**	**11·9 (9·2 to 15·3)**	..	**1·5 (1·0 to 2·1)**	**14·6 (9·8 to 19·2)**
Antigua and Barbuda	10·0 (7·4 to 13·1)	11·1 (8·3 to 14·4)	11·3 (8·5 to 14·6)	11·1 (8·4 to 14·3)	11·4 (8·5 to 14·8)	11·6 (8·6 to 14·9)	47	1·5 (0·3 to 2·7)	15·6 (3·2 to 29·0)
Bahamas	9·5 (7·1 to 12·2)	9·8 (7·4 to 12·8)	9·7 (7·3 to 12·7)	9·0 (6·7 to 11·8)	10·0 (7·4 to 13·1)	10·4 (7·7 to 13·6)	106	0·9 (0·0 to 1·8)	9·4 (0·1 to 19·3)
Barbados	9·9 (7·4 to 12·8)	10·9 (8·3 to 14·1)	10·8 (8·2 to 14·0)	10·6 (8·2 to 13·7)	10·7 (8·0 to 13·8)	11·1 (8·4 to 14·4)	73	1·2 (0·3 to 2·0)	11·7 (3·5 to 20·7)
Belize	10·0 (7·5 to 12·9)	10·4 (7·9 to 13·5)	10·6 (8·0 to 13·6)	10·4 (7·9 to 13·4)	11·0 (8·3 to 14·3)	11·2 (8·6 to 14·5)	61	1·3 (0·4 to 2·3)	13·0 (4·3 to 24·1)
Bermuda	9·7 (7·3 to 12·4)	11·2 (8·5 to 14·4)	11·4 (8·7 to 14·6)	11·3 (8·5 to 14·6)	11·5 (8·6 to 14·8)	11·7 (8·8 to 15·1)	45	2·1 (1·2 to 2·9)	21·5 (13·1 to 29·2)
Bolivia	8·6 (6·5 to 11·2)	9·2 (6·9 to 11·9)	8·0 (6·0 to 10·4)	8·5 (6·4 to 11·0)	10·0 (7·4 to 12·8)	10·2 (7·5 to 13·2)	114	1·5 (0·6 to 2·5)	18·0 (7·2 to 29·4)
Brazil	10·4 (7·9 to 13·4)	11·2 (8·5 to 14·2)	11·7 (8·9 to 14·8)	11·4 (8·7 to 14·4)	12·2 (9·3 to 15·5)	12·5 (9·5 to 15·9)	16	2·1 (1·4 to 2·9)	20·0 (14·4 to 25·9)
Colombia	10·5 (8·0 to 13·5)	11·5 (8·7 to 14·5)	11·3 (8·7 to 14·5)	11·0 (8·4 to 14·1)	11·9 (9·1 to 15·2)	12·2 (9·4 to 15·4)	25	1·7 (1·0 to 2·4)	16·2 (9·6 to 22·7)
Costa Rica	11·2 (8·6 to 14·4)	12·1 (9·2 to 15·3)	12·4 (9·5 to 15·7)	12·2 (9·4 to 15·3)	12·5 (9·5 to 15·7)	12·5 (9·6 to 15·8)	14	1·3 (0·7 to 2·0)	11·8 (6·3 to 17·8)
Cuba	10·2 (7·6 to 13·0)	10·9 (8·2 to 13·9)	11·0 (8·4 to 14·1)	9·9 (7·6 to 12·9)	11·0 (8·2 to 14·4)	11·3 (8·4 to 14·7)	59	1·1 (0·0 to 2·4)	11·3 (0·4 to 23·1)
Dominica	10·3 (7·8 to 13·3)	10·6 (8·0 to 13·8)	10·8 (8·2 to 14·1)	10·7 (8·0 to 13·9)	11·0 (8·3 to 14·4)	11·2 (8·5 to 14·7)	64	0·9 (0·1 to 1·8)	8·6 (0·5 to 17·5)
Dominican Republic	9·7 (7·4 to 12·6)	10·7 (8·0 to 13·9)	10·2 (7·8 to 13·2)	10·4 (8·0 to 13·5)	10·4 (8·0 to 13·6)	10·5 (8·0 to 13·8)	99	0·8 (−0·1 to 1·7)	8·0 (−1·2 to 17·8)
Ecuador	9·6 (7·3 to 12·4)	10·2 (7·6 to 13·0)	10·0 (7·6 to 12·8)	10·5 (8·0 to 13·4)	11·1 (8·4 to 14·1)	11·1 (8·4 to 14·1)	69	1·5 (0·8 to 2·3)	15·8 (9·2 to 23·6)
El Salvador	10·1 (7·6 to 12·9)	10·7 (8·1 to 13·9)	10·2 (7·8 to 13·0)	10·1 (7·6 to 12·7)	10·6 (8·1 to 13·5)	10·6 (8·0 to 13·6)	91	0·6 (−0·4 to 1·5)	5·8 (−3·8 to 16·4)
Grenada	9·7 (7·2 to 12·5)	10·8 (8·1 to 13·8)	10·9 (8·1 to 13·9)	10·4 (7·6 to 13·4)	11·0 (8·1 to 14·1)	11·2 (8·5 to 14·4)	60	1·6 (0·2 to 2·9)	16·7 (2·7 to 31·9)
Guatemala	9·4 (7·2 to 12·2)	10·9 (8·2 to 14·0)	11·0 (8·4 to 14·0)	10·5 (8·1 to 13·4)	11·3 (8·6 to 14·6)	11·7 (8·8 to 15·3)	44	2·3 (1·0 to 3·5)	24·5 (11·7 to 39·4)
Guyana	9·2 (6·7 to 12·0)	9·5 (7·0 to 12·3)	10·0 (7·3 to 12·9)	9·5 (6·9 to 12·1)	9·8 (7·1 to 12·7)	10·0 (7·2 to 13·0)	122	0·7 (−0·9 to 2·3)	8·3 (−8·5 to 27·1)
Haiti	7·2 (5·2 to 9·5)	3·7 (1·1 to 6·1)	8·8 (6·6 to 11·4)	8·8 (6·6 to 11·4)	8·8 (6·5 to 11·6)	8·7 (6·3 to 11·2)	179	1·5 (0·1 to 2·9)	21·9 (0·8 to 44·1)
Honduras	10·3 (7·8 to 13·0)	10·8 (8·3 to 13·7)	10·6 (8·2 to 13·5)	10·3 (7·9 to 13·2)	11·5 (8·7 to 14·6)	11·5 (8·7 to 14·6)	50	1·2 (0·4 to 2·1)	11·7 (3·8 to 20·5)
Jamaica	10·3 (7·8 to 13·3)	10·9 (8·3 to 14·0)	10·5 (7·7 to 13·6)	10·3 (7·6 to 13·3)	10·8 (8·0 to 14·0)	11·1 (8·3 to 14·5)	72	0·8 (−0·5 to 2·1)	7·8 (−5·5 to 23·0)
Mexico	11·6 (8·7 to 14·8)	11·9 (9·0 to 15·1)	11·0 (8·4 to 14·0)	11·2 (8·6 to 14·2)	12·4 (9·6 to 15·8)	12·6 (9·7 to 16·2)	11	1·1 (0·6 to 1·6)	9·3 (5·9 to 13·4)
Nicaragua	10·5 (7·9 to 13·6)	10·8 (8·2 to 13·8)	10·2 (7·7 to 13·0)	10·2 (7·9 to 13·2)	11·4 (8·6 to 14·5)	11·8 (8·6 to 15·2)	38	1·4 (0·1 to 2·6)	13·1 (0·5 to 26·2)
Panama	10·8 (8·2 to 13·8)	11·3 (8·6 to 14·3)	11·3 (8·6 to 14·3)	11·6 (8·9 to 14·6)	12·0 (9·1 to 15·1)	12·0 (9·2 to 15·5)	31	1·3 (0·6 to 2·0)	12·0 (5·7 to 18·7)
Paraguay	10·5 (8·0 to 13·5)	10·2 (7·7 to 13·1)	10·3 (7·8 to 13·3)	9·2 (7·0 to 11·9)	10·5 (7·8 to 13·6)	10·7 (8·0 to 13·7)	90	0·2 (−0·7 to 1·1)	2·0 (−6·8 to 11·1)
Peru	10·6 (8·0 to 13·8)	11·1 (8·4 to 14·2)	9·7 (7·3 to 12·6)	9·6 (7·3 to 12·3)	11·3 (8·6 to 14·4)	11·1 (8·5 to 14·2)	75	0·5 (−0·2 to 1·2)	4·8 (−2·0 to 12·3)
Puerto Rico	10·7 (8·1 to 13·8)	11·7 (8·8 to 14·9)	12·4 (9·5 to 15·8)	12·4 (9·5 to 15·9)	12·4 (9·5 to 15·9)	12·7 (9·7 to 16·2)	10	2·0 (1·2 to 2·8)	18·6 (11·6 to 26·3)
Saint Kitts and Nevis	9·4 (7·0 to 12·3)	10·2 (7·6 to 13·2)	10·6 (7·9 to 13·8)	10·4 (7·9 to 13·5)	10·8 (8·1 to 14·0)	10·9 (8·2 to 14·2)	83	1·6 (0·6 to 2·6)	16·9 (6·7 to 28·3)
Saint Lucia	10·0 (7·4 to 12·9)	11·0 (8·3 to 14·2)	11·5 (8·6 to 14·9)	11·0 (8·2 to 14·4)	11·7 (8·7 to 15·2)	11·9 (8·8 to 15·4)	37	1·9 (0·3 to 3·3)	19·3 (3·9 to 34·9)
Saint Vincent and the Grenadines	10·0 (7·4 to 13·0)	10·6 (7·8 to 13·9)	10·8 (8·2 to 14·0)	10·9 (8·2 to 14·2)	11·1 (8·2 to 14·5)	11·1 (8·2 to 14·7)	68	1·1 (−0·2 to 2·5)	11·7 (−2·3 to 26·4)
Suriname	9·9 (7·5 to 12·8)	10·3 (7·8 to 13·5)	10·6 (8·0 to 13·9)	9·6 (7·3 to 12·7)	10·8 (8·2 to 14·0)	11·1 (8·4 to 14·4)	71	1·2 (0·3 to 2·1)	12·3 (2·9 to 21·6)
Trinidad and Tobago	9·6 (7·3 to 12·5)	10·7 (8·1 to 14·1)	10·7 (8·2 to 13·9)	10·0 (7·6 to 12·9)	10·6 (8·0 to 13·7)	11·0 (8·4 to 14·3)	79	1·3 (0·5 to 2·3)	14·0 (4·8 to 23·6)
Venezuela	10·2 (7·8 to 13·1)	10·8 (8·2 to 13·8)	10·3 (7·8 to 13·4)	10·2 (7·8 to 13·3)	10·4 (7·8 to 13·5)	10·5 (7·8 to 13·4)	100	0·3 (−0·4 to 1·1)	3·3 (−4·1 to 11·0)
Virgin Islands	9·3 (6·8 to 12·2)	10·5 (7·9 to 13·6)	11·2 (8·3 to 14·4)	11·0 (8·2 to 14·2)	11·2 (8·2 to 14·4)	11·1 (8·2 to 14·4)	70	1·8 (0·3 to 3·2)	19·7 (4·0 to 36·9)
**North Africa and Middle East**	**9·2 (7·0 to 11·8)**	**10·2 (7·8 to 13·1)**	**10·5 (8·0 to 13·4)**	**10·6 (8·1 to 13·5)**	**11·0 (8·4 to 14·0)**	**10·9 (8·4 to 14·1)**	..	**1·8 (1·1 to 2·5)**	**19·2 (12·1 to 27·1)**
Afghanistan	10·9 (7·7 to 14·6)	11·6 (8·7 to 15·4)	11·6 (8·4 to 15·0)	11·2 (8·3 to 14·5)	12·4 (9·4 to 15·9)	12·2 (9·3 to 15·9)	26	1·3 (−0·4 to 3·0)	12·5 (−3·2 to 31·1)
Algeria	9·3 (7·0 to 12·0)	10·2 (7·8 to 13·3)	10·5 (8·0 to 13·6)	10·6 (8·1 to 13·8)	10·8 (8·2 to 13·9)	10·8 (8·1 to 14·0)	86	1·5 (0·7 to 2·3)	16·3 (7·8 to 25·1)
Bahrain	9·2 (6·9 to 12·0)	10·1 (7·6 to 13·2)	10·8 (8·1 to 14·0)	10·8 (8·0 to 13·9)	11·1 (8·1 to 14·6)	11·2 (8·3 to 14·6)	67	2·0 (0·7 to 3·3)	21·7 (8·3 to 37·2)
Egypt	8·7 (6·6 to 11·3)	9·2 (7·0 to 11·7)	9·6 (7·3 to 12·3)	9·6 (7·4 to 12·3)	9·8 (7·2 to 12·6)	9·8 (7·2 to 12·7)	132	1·0 (−0·1 to 2·2)	11·7 (−1·1 to 25·6)
Iran	9·7 (7·3 to 12·5)	11·4 (8·6 to 14·5)	11·1 (8·4 to 14·3)	11·4 (8·6 to 14·7)	12·3 (9·3 to 15·7)	12·5 (9·6 to 16·1)	15	2·8 (1·9 to 3·9)	29·2 (20·0 to 38·9)
Iraq	9·8 (7·5 to 12·6)	9·8 (7·3 to 12·5)	9·9 (7·6 to 12·8)	10·5 (8·0 to 13·4)	10·8 (8·2 to 13·8)	10·6 (8·1 to 13·7)	92	0·8 (−0·1 to 1·7)	8·7 (−0·5 to 17·7)
Jordan	9·3 (7·1 to 12·1)	10·2 (7·7 to 13·1)	10·5 (8·1 to 13·4)	10·3 (7·9 to 13·1)	11·3 (8·6 to 14·4)	11·4 (8·6 to 14·6)	54	2·1 (1·2 to 2·9)	22·1 (13·6 to 31·0)
Kuwait	9·5 (7·1 to 12·2)	11·3 (8·6 to 14·3)	11·7 (8·9 to 14·8)	11·8 (9·0 to 15·0)	12·3 (9·3 to 15·8)	12·4 (9·4 to 16·0)	19	3·0 (1·9 to 4·0)	31·5 (22·5 to 40·2)
Lebanon	11·9 (8·9 to 15·4)	12·4 (9·4 to 15·8)	10·8 (8·2 to 13·9)	12·3 (9·4 to 15·6)	12·5 (9·4 to 16·0)	12·8 (9·5 to 16·5)	8	0·9 (−0·7 to 2·4)	7·7 (−6·4 to 21·9)
Libya	9·5 (7·1 to 12·3)	10·4 (7·9 to 13·4)	10·8 (8·0 to 14·0)	10·4 (7·8 to 13·4)	11·4 (8·5 to 14·7)	10·4 (7·7 to 13·5)	102	0·9 (−0·2 to 1·9)	9·5 (−1·7 to 21·5)
Morocco	8·7 (6·4 to 11·3)	9·5 (7·2 to 12·4)	9·8 (7·5 to 12·8)	10·1 (7·6 to 12·9)	10·1 (7·6 to 13·3)	10·0 (7·4 to 13·2)	120	1·4 (0·1 to 2·6)	16·2 (1·7 to 32·3)
Oman	9·5 (7·0 to 12·3)	10·0 (7·6 to 12·9)	9·9 (7·5 to 12·8)	9·9 (7·4 to 12·7)	10·8 (8·1 to 13·8)	10·9 (8·1 to 14·0)	85	1·4 (0·3 to 2·4)	14·8 (3·3 to 27·8)
Palestine	10·1 (7·8 to 13·1)	10·6 (8·1 to 13·7)	11·3 (8·6 to 14·5)	11·1 (8·5 to 14·3)	11·5 (8·8 to 14·8)	9·8 (7·0 to 12·8)	126	−0·3 (−1·6 to 1·0)	−2·7 (−17·2 to 11·2)
Qatar	9·2 (6·8 to 12·0)	10·4 (7·8 to 13·3)	11·6 (8·7 to 14·8)	11·9 (8·9 to 15·2)	12·0 (8·9 to 15·3)	11·9 (8·8 to 15·3)	35	2·7 (1·5 to 4·1)	30·2 (16·4 to 46·2)
Saudi Arabia	9·5 (7·1 to 12·5)	10·0 (7·5 to 12·9)	10·3 (7·6 to 13·3)	10·7 (8·2 to 13·9)	10·9 (8·3 to 14·1)	11·0 (8·4 to 14·4)	76	1·6 (0·4 to 2·7)	16·7 (4·4 to 28·8)
Sudan	7·6 (5·4 to 10·1)	8·9 (6·6 to 11·7)	9·4 (7·0 to 12·3)	9·7 (7·1 to 12·7)	9·8 (7·1 to 12·7)	9·7 (7·0 to 12·8)	136	2·1 (0·6 to 3·7)	28·0 (7·6 to 51·9)
Syria	10·0 (7·5 to 12·9)	10·7 (8·1 to 13·8)	12·2 (9·3 to 15·7)	12·3 (9·4 to 15·7)	12·7 (9·7 to 16·3)	12·6 (9·6 to 16·2)	12	2·6 (1·6 to 3·7)	26·6 (16·9 to 37·4)
Tunisia	9·4 (7·2 to 11·9)	10·2 (7·7 to 12·9)	10·2 (7·7 to 13·0)	9·8 (7·4 to 12·5)	11·0 (8·3 to 13·9)	11·2 (8·5 to 14·2)	65	1·8 (1·1 to 2·6)	19·0 (12·2 to 26·2)
Türkiye	9·4 (7·0 to 12·0)	11·0 (8·3 to 13·8)	10·9 (8·3 to 13·9)	10·8 (8·2 to 13·8)	11·1 (8·5 to 14·2)	11·0 (8·1 to 14·1)	78	1·6 (0·5 to 2·9)	17·4 (4·8 to 31·4)
United Arab Emirates	9·0 (6·8 to 11·7)	10·2 (7·7 to 13·1)	11·1 (8·4 to 14·3)	11·2 (8·5 to 14·3)	11·5 (8·8 to 14·8)	11·4 (8·5 to 14·7)	52	2·4 (1·3 to 3·5)	26·7 (16·0 to 37·8)
Yemen	9·0 (6·5 to 11·7)	10·3 (7·6 to 13·2)	10·6 (7·8 to 13·6)	10·9 (8·1 to 14·1)	11·4 (8·5 to 14·8)	11·4 (8·6 to 14·8)	51	2·5 (0·9 to 4·0)	28·1 (10·0 to 47·5)
**South Asia**	**8·4 (6·2 to 11·0)**	**9·7 (7·4 to 12·4)**	**10·1 (7·6 to 13·1)**	**9·9 (7·5 to 12·8)**	**10·5 (7·8 to 13·5)**	**10·4 (7·8 to 13·4)**	..	**2·0 (0·9 to 3·2)**	**24·7 (11·4 to 39·6)**
Bangladesh	8·1 (6·0 to 10·5)	9·2 (6·9 to 11·9)	9·5 (7·1 to 12·5)	9·5 (7·0 to 12·2)	9·9 (7·4 to 12·9)	9·8 (7·3 to 12·7)	127	1·7 (0·4 to 3·1)	22·0 (5·2 to 42·0)
Bhutan	7·7 (5·5 to 10·0)	9·2 (6·8 to 11·9)	9·6 (7·1 to 12·4)	9·7 (7·1 to 12·5)	9·7 (7·2 to 12·7)	9·8 (7·2 to 12·7)	129	2·1 (0·7 to 3·6)	27·6 (10·0 to 49·6)
India	8·4 (6·2 to 11·0)	9·8 (7·4 to 12·5)	10·2 (7·7 to 13·2)	10·0 (7·5 to 12·9)	10·6 (7·9 to 13·8)	10·5 (7·8 to 13·6)	96	2·2 (0·9 to 3·5)	26·1 (10·5 to 44·3)
Nepal	8·6 (6·3 to 11·5)	10·0 (7·7 to 12·9)	10·2 (7·7 to 13·1)	9·8 (7·3 to 12·6)	10·6 (7·8 to 13·8)	10·5 (7·7 to 13·6)	97	1·9 (0·4 to 3·4)	22·5 (4·5 to 44·1)
Pakistan	8·6 (6·3 to 11·2)	9·6 (7·3 to 12·5)	9·8 (7·1 to 12·7)	9·8 (7·1 to 12·8)	10·5 (7·8 to 13·8)	10·4 (7·7 to 13·7)	103	1·8 (0·4 to 3·3)	21·0 (4·4 to 41·5)
**Southeast Asia, East Asia, and Oceania**	**8·2 (6·2 to 10·4)**	**9·4 (7·1 to 12·0)**	**10·1 (7·8 to 12·9)**	**9·9 (7·6 to 12·6)**	**10·0 (7·5 to 12·8)**	**10·1 (7·5 to 13·0)**	..	**2·0 (1·0 to 3·0)**	**24·3 (13·0 to 36·1)**
American Samoa	8·5 (6·3 to 11·3)	8·8 (6·5 to 11·5)	9·0 (6·7 to 11·9)	8·6 (6·2 to 11·2)	9·2 (6·5 to 12·1)	9·4 (6·7 to 12·3)	148	0·9 (−0·5 to 2·1)	10·5 (−5·6 to 27·0)
Cambodia	7·9 (5·8 to 10·5)	9·1 (6·8 to 11·9)	9·5 (7·1 to 12·4)	9·4 (7·0 to 12·4)	9·6 (7·2 to 12·6)	9·5 (7·0 to 12·4)	141	1·6 (0·2 to 2·9)	20·5 (2·0 to 41·2)
China	8·1 (6·1 to 10·4)	9·6 (7·3 to 12·3)	10·6 (8·2 to 13·6)	10·5 (8·1 to 13·4)	10·5 (7·8 to 13·4)	10·6 (7·8 to 13·6)	94	2·5 (1·2 to 3·8)	30·7 (15·2 to 47·7)
Cook Islands	8·2 (6·1 to 10·6)	9·1 (6·8 to 11·8)	9·4 (7·0 to 12·2)	9·2 (6·8 to 11·8)	9·6 (7·0 to 12·4)	9·7 (7·1 to 12·6)	135	1·5 (0·7 to 2·4)	18·6 (8·5 to 28·6)
Federated States of Micronesia	7·1 (5·3 to 9·2)	7·9 (5·9 to 10·3)	8·4 (6·3 to 10·9)	8·1 (6·1 to 10·6)	8·6 (6·5 to 11·2)	8·7 (6·5 to 11·5)	176	1·6 (0·8 to 2·5)	23·3 (11·6 to 35·7)
Fiji	8·5 (6·4 to 11·0)	8·4 (6·3 to 10·9)	8·8 (6·6 to 11·4)	8·5 (6·4 to 11·0)	8·9 (6·6 to 11·5)	9·0 (6·7 to 11·6)	163	0·5 (−0·3 to 1·4)	6·1 (−3·3 to 17·3)
Guam	8·6 (6·5 to 11·1)	9·5 (7·2 to 12·2)	9·5 (7·1 to 12·5)	9·4 (7·1 to 12·2)	9·6 (7·1 to 12·4)	9·7 (7·2 to 12·8)	134	1·1 (0·3 to 1·9)	12·2 (3·6 to 22·0)
Indonesia	8·4 (6·1 to 11·0)	8·7 (6·6 to 11·3)	8·9 (6·5 to 11·4)	8·3 (6·0 to 10·9)	9·2 (6·7 to 12·2)	9·2 (6·5 to 12·0)	157	0·8 (−0·6 to 2·2)	10·0 (−7·1 to 27·7)
Kiribati	9·0 (6·8 to 11·7)	9·5 (7·1 to 12·2)	9·9 (7·5 to 12·9)	9·6 (7·3 to 12·6)	10·1 (7·6 to 13·2)	10·2 (7·5 to 13·4)	113	1·2 (0·3 to 2·1)	13·4 (3·1 to 24·9)
Laos	5·9 (4·0 to 8·0)	7·1 (5·2 to 9·2)	7·3 (5·3 to 9·5)	7·3 (5·4 to 9·6)	7·4 (5·2 to 9·7)	7·4 (5·1 to 9·8)	202	1·5 (−0·2 to 3·2)	26·3 (−3·7 to 63·0)
Malaysia	8·5 (6·5 to 10·8)	9·1 (6·7 to 11·7)	9·5 (7·2 to 12·1)	8·8 (6·7 to 11·2)	9·2 (7·0 to 11·7)	9·6 (7·3 to 12·2)	137	1·1 (0·4 to 1·7)	12·5 (5·2 to 20·3)
Maldives	8·0 (6·1 to 10·3)	8·6 (6·5 to 11·2)	8·8 (6·7 to 11·5)	8·7 (6·6 to 11·3)	8·8 (6·6 to 11·4)	8·8 (6·6 to 11·4)	174	0·9 (0·1 to 1·6)	10·9 (1·1 to 20·3)
Marshall Islands	7·3 (5·5 to 9·5)	7·6 (5·7 to 10·0)	7·7 (5·7 to 10·2)	7·5 (5·6 to 9·9)	7·9 (6·0 to 10·3)	7·9 (6·0 to 10·4)	196	0·6 (−0·2 to 1·4)	8·5 (−1·7 to 20·5)
Mauritius	8·7 (6·6 to 11·3)	9·8 (7·5 to 12·6)	10·2 (7·7 to 13·1)	9·8 (7·5 to 12·5)	10·0 (7·6 to 12·8)	10·1 (7·7 to 13·0)	119	1·4 (0·6 to 2·2)	15·9 (7·3 to 24·9)
Myanmar	6·8 (5·0 to 9·1)	7·5 (5·5 to 9·7)	7·9 (5·7 to 10·5)	7·6 (5·5 to 10·1)	8·0 (5·5 to 10·5)	8·0 (5·5 to 10·7)	195	1·2 (−0·3 to 2·6)	17·8 (−6·1 to 42·3)
Nauru	6·4 (4·8 to 8·4)	6·5 (4·9 to 8·5)	6·7 (4·9 to 8·8)	6·3 (4·6 to 8·4)	6·8 (5·0 to 9·1)	6·9 (5·0 to 9·2)	204	0·5 (−0·4 to 1·4)	8·2 (−6·5 to 22·8)
Niue	8·2 (6·1 to 10·6)	8·9 (6·7 to 11·4)	9·3 (7·1 to 12·0)	8·9 (6·8 to 11·5)	9·1 (7·0 to 11·8)	9·3 (7·0 to 11·9)	155	1·0 (0·3 to 1·7)	12·3 (3·8 to 21·5)
North Korea	8·5 (6·2 to 11·2)	8·8 (6·6 to 11·6)	9·2 (7·0 to 12·2)	9·3 (6·8 to 12·2)	9·3 (6·8 to 12·1)	9·4 (6·8 to 12·4)	146	0·9 (−0·5 to 2·2)	10·7 (−5·2 to 27·9)
Northern Mariana Islands	7·8 (5·9 to 10·1)	8·3 (6·3 to 10·8)	8·5 (6·4 to 11·1)	8·2 (6·2 to 10·7)	8·7 (6·6 to 11·4)	8·9 (6·6 to 11·6)	168	1·0 (0·3 to 1·8)	13·3 (3·4 to 23·9)
Palau	7·9 (5·9 to 10·3)	8·5 (6·3 to 10·9)	8·6 (6·4 to 11·2)	8·3 (6·1 to 10·9)	8·8 (6·4 to 11·5)	8·9 (6·5 to 11·7)	166	1·0 (0·3 to 1·9)	13·3 (3·3 to 24·4)
Papua New Guinea	7·7 (5·6 to 10·3)	7·9 (5·8 to 10·3)	8·3 (6·0 to 11·0)	8·3 (6·0 to 10·9)	8·4 (5·8 to 10·9)	8·4 (5·8 to 11·2)	186	0·7 (−0·7 to 2·0)	9·1 (−9·1 to 27·9)
Philippines	8·7 (6·6 to 11·1)	8·4 (6·4 to 10·8)	8·7 (6·6 to 11·1)	7·9 (6·0 to 10·0)	8·6 (6·5 to 10·9)	8·9 (6·7 to 11·3)	170	0·2 (−0·4 to 0·7)	2·3 (−4·0 to 8·5)
Samoa	9·0 (6·8 to 11·6)	9·2 (6·9 to 11·8)	9·3 (7·0 to 11·9)	9·1 (6·9 to 11·7)	9·5 (7·1 to 12·2)	9·5 (7·1 to 12·3)	139	0·5 (−0·3 to 1·4)	5·7 (−3·1 to 15·2)
Seychelles	8·2 (6·2 to 10·6)	8·6 (6·4 to 11·3)	9·5 (7·1 to 12·3)	8·6 (6·5 to 11·3)	9·4 (7·1 to 12·1)	9·4 (7·1 to 12·1)	149	1·2 (0·4 to 2·0)	14·5 (5·0 to 25·3)
Solomon Islands	6·5 (4·8 to 8·5)	7·1 (5·3 to 9·3)	7·1 (5·3 to 9·4)	7·1 (5·3 to 9·4)	7·2 (5·4 to 9·4)	7·3 (5·3 to 9·5)	203	0·8 (0·0 to 1·6)	12·5 (0·6 to 26·4)
Sri Lanka	8·7 (6·6 to 11·1)	9·3 (7·0 to 11·8)	10·0 (7·6 to 12·7)	9·6 (7·4 to 12·2)	9·7 (7·4 to 12·3)	9·8 (7·4 to 12·5)	131	1·1 (0·4 to 1·8)	12·9 (5·1 to 20·8)
Taiwan*	9·1 (6·8 to 11·6)	10·6 (8·0 to 13·5)	11·4 (8·7 to 14·3)	11·4 (8·7 to 14·4)	11·2 (8·5 to 14·2)	11·4 (8·7 to 14·5)	53	2·4 (1·7 to 3·1)	26·3 (21·1 to 31·1)
Thailand	9·3 (7·0 to 11·8)	9·5 (7·2 to 12·0)	10·2 (7·8 to 12·9)	10·0 (7·6 to 12·6)	9·9 (7·5 to 12·5)	10·2 (7·8 to 12·9)	112	0·9 (0·4 to 1·4)	9·6 (4·7 to 14·7)
Timor-Leste	7·2 (5·2 to 9·5)	8·4 (6·3 to 10·9)	8·4 (6·2 to 10·8)	8·4 (6·1 to 10·9)	8·6 (6·2 to 11·2)	8·6 (6·1 to 11·1)	182	1·3 (−0·1 to 2·7)	19·0 (−1·0 to 41·8)
Tokelau	9·1 (6·8 to 11·8)	9·6 (7·2 to 12·6)	10·1 (7·7 to 13·3)	9·8 (7·4 to 12·9)	10·0 (7·5 to 13·2)	10·1 (7·6 to 13·2)	116	1·1 (0·2 to 1·9)	11·8 (2·8 to 21·1)
Tonga	8·8 (6·7 to 11·4)	9·0 (6·7 to 11·7)	9·2 (6·8 to 12·2)	8·8 (6·5 to 11·7)	9·3 (6·9 to 12·3)	9·4 (6·9 to 12·4)	145	0·6 (−0·2 to 1·5)	6·9 (−2·5 to 16·7)
Tuvalu	7·2 (5·4 to 9·5)	7·6 (5·8 to 9·9)	7·4 (5·5 to 9·8)	7·2 (5·3 to 9·5)	7·6 (5·6 to 10·0)	7·6 (5·6 to 10·0)	201	0·4 (−0·5 to 1·3)	5·4 (−6·4 to 18·0)
Vanuatu	8·1 (6·1 to 10·4)	8·4 (6·4 to 11·0)	8·6 (6·5 to 11·2)	8·5 (6·5 to 11·2)	8·8 (6·7 to 11·3)	8·9 (6·7 to 11·4)	172	0·8 (−0·1 to 1·6)	9·5 (−1·2 to 21·1)
Viet Nam	8·7 (6·4 to 11·2)	9·0 (6·7 to 11·6)	9·3 (6·9 to 12·0)	9·1 (6·8 to 11·8)	9·3 (6·8 to 11·9)	9·4 (6·8 to 12·2)	147	0·7 (−0·7 to 2·1)	8·3 (−8·3 to 25·9)
**Sub-Saharan Africa**	**7·3 (5·4 to 9·4)**	**8·1 (6·1 to 10·4)**	**8·6 (6·6 to 11·1)**	**8·6 (6·5 to 11·0)**	**9·0 (6·8 to 11·6)**	**9·0 (6·8 to 11·5)**	..	**1·7 (1·0 to 2·4)**	**23·7 (15·1 to 33·5)**
Angola	7·1 (5·1 to 9·4)	8·6 (6·4 to 11·3)	9·1 (6·9 to 11·9)	9·0 (6·6 to 11·7)	9·5 (6·9 to 12·6)	9·5 (6·7 to 12·3)	142	2·4 (0·6 to 4·2)	34·4 (9·4 to 62·7)
Benin	7·6 (5·6 to 10·0)	8·5 (6·3 to 11·1)	8·9 (6·5 to 11·6)	9·0 (6·6 to 11·8)	9·1 (6·6 to 11·8)	9·1 (6·6 to 11·9)	162	1·5 (0·1 to 2·9)	20·0 (1·1 to 43·3)
Botswana	9·0 (6·7 to 11·4)	9·7 (7·3 to 12·4)	10·0 (7·5 to 12·7)	9·2 (6·9 to 11·7)	10·1 (7·5 to 12·9)	10·2 (7·6 to 13·1)	110	1·3 (0·3 to 2·2)	14·3 (3·8 to 25·3)
Burkina Faso	7·2 (5·1 to 9·5)	8·6 (6·3 to 11·2)	9·4 (7·1 to 12·0)	9·8 (7·2 to 12·6)	9·7 (7·2 to 12·7)	9·5 (6·9 to 12·4)	140	2·3 (0·8 to 3·9)	33·5 (9·8 to 59·7)
Burundi	6·0 (4·3 to 8·0)	8·0 (5·9 to 10·4)	8·3 (6·1 to 10·7)	8·1 (6·0 to 10·5)	8·5 (6·3 to 10·8)	8·4 (5·9 to 11·0)	187	2·3 (0·7 to 3·9)	40·0 (11·7 to 76·8)
Cabo Verde	9·4 (7·2 to 12·4)	10·0 (7·5 to 12·9)	10·0 (7·4 to 12·8)	10·0 (7·3 to 13·0)	10·4 (7·6 to 13·4)	10·4 (7·6 to 13·4)	104	0·9 (−0·3 to 2·3)	10·2 (−3·4 to 26·9)
Cameroon	7·7 (5·5 to 10·1)	7·3 (5·4 to 9·5)	8·0 (5·9 to 10·2)	7·8 (5·7 to 10·0)	8·3 (6·0 to 10·6)	8·3 (6·1 to 10·5)	191	0·6 (−0·7 to 2·1)	8·7 (−8·6 to 28·6)
Central African Republic	7·5 (5·3 to 9·9)	7·4 (5·3 to 9·7)	8·4 (6·1 to 10·9)	8·7 (6·3 to 11·4)	8·7 (6·4 to 11·5)	8·7 (6·4 to 11·2)	177	1·3 (−0·2 to 2·7)	17·7 (−2·3 to 40·5)
Chad	6·3 (4·5 to 8·5)	7·1 (5·2 to 9·2)	7·6 (5·6 to 9·8)	8·0 (5·9 to 10·5)	7·8 (5·7 to 10·1)	7·8 (5·6 to 10·2)	199	1·5 (0·0 to 2·9)	24·0 (−0·1 to 51·6)
Comoros	9·3 (7·0 to 12·3)	10·3 (7·7 to 13·4)	10·4 (7·8 to 13·6)	9·9 (7·3 to 12·9)	10·9 (8·1 to 14·0)	10·9 (8·0 to 14·2)	82	1·6 (0·2 to 3·0)	17·6 (2·0 to 34·8)
Congo (Brazzaville)	7·7 (5·7 to 10·2)	8·8 (6·4 to 11·3)	9·0 (6·6 to 11·6)	9·0 (6·7 to 11·7)	9·3 (6·8 to 12·0)	9·1 (6·5 to 12·0)	160	1·4 (−0·1 to 2·9)	18·6 (−0·9 to 40·1)
Côte d’Ivoire	8·1 (6·0 to 10·6)	8·2 (6·1 to 10·6)	8·7 (6·4 to 11·3)	9·0 (6·5 to 11·7)	9·0 (6·4 to 11·6)	8·8 (6·3 to 11·7)	173	0·8 (−0·7 to 2·2)	10·2 (−9·1 to 30·5)
DR Congo	8·1 (5·8 to 10·6)	8·5 (6·4 to 10·9)	8·6 (6·3 to 11·2)	8·9 (6·5 to 11·5)	8·9 (6·4 to 11·6)	8·9 (6·3 to 11·5)	169	0·8 (−0·6 to 2·2)	10·7 (−6·8 to 31·5)
Djibouti	7·7 (5·7 to 10·3)	8·2 (6·0 to 10·7)	8·6 (6·3 to 11·3)	8·4 (6·0 to 11·0)	8·9 (6·3 to 11·8)	8·9 (6·3 to 11·9)	167	1·2 (−0·4 to 2·6)	15·3 (−5·1 to 37·1)
Equatorial Guinea	7·0 (5·0 to 9·3)	8·0 (5·9 to 10·2)	8·2 (6·0 to 10·9)	8·4 (6·1 to 11·2)	8·5 (6·1 to 11·4)	8·4 (5·8 to 11·1)	188	1·3 (−0·4 to 2·9)	19·6 (−5·1 to 47·9)
Eritrea	6·7 (4·4 to 9·3)	8·5 (6·3 to 11·3)	8·8 (6·5 to 11·5)	8·9 (6·4 to 11·6)	8·5 (6·2 to 11·2)	8·7 (6·2 to 11·6)	175	2·0 (0·2 to 4·1)	31·5 (2·2 to 70·4)
Eswatini	6·8 (4·9 to 9·0)	6·5 (4·6 to 8·6)	7·9 (5·7 to 10·4)	7·8 (5·6 to 10·3)	8·5 (6·1 to 11·3)	8·7 (6·1 to 11·4)	180	1·9 (0·1 to 3·6)	28·3 (1·2 to 58·9)
Ethiopia	5·9 (4·2 to 7·9)	7·7 (5·8 to 10·0)	8·2 (6·0 to 10·7)	8·1 (5·9 to 10·5)	8·6 (6·2 to 11·4)	8·4 (6·2 to 11·0)	183	2·5 (1·0 to 4·0)	43·9 (18·2 to 76·4)
Gabon	8·9 (6·6 to 11·6)	9·3 (7·0 to 12·1)	9·7 (7·1 to 12·4)	10·0 (7·2 to 12·8)	10·1 (7·4 to 13·0)	9·9 (7·3 to 12·8)	124	1·0 (−0·4 to 2·4)	11·5 (−3·9 to 30·2)
Ghana	7·7 (5·5 to 10·1)	8·2 (6·2 to 10·8)	8·5 (6·3 to 11·2)	8·5 (6·3 to 11·3)	8·7 (6·3 to 11·4)	8·6 (6·1 to 11·7)	181	1·0 (−0·4 to 2·5)	13·3 (−6·2 to 34·6)
Guinea	6·4 (4·5 to 8·6)	7·7 (5·6 to 10·0)	8·3 (6·2 to 10·6)	8·5 (6·2 to 11·0)	8·5 (6·1 to 11·1)	8·4 (6·0 to 11·0)	184	2·0 (0·4 to 3·6)	31·7 (6·3 to 64·7)
Guinea-Bissau	7·0 (5·0 to 9·5)	7·8 (5·6 to 10·1)	8·1 (6·0 to 10·6)	8·5 (6·2 to 11·2)	8·5 (6·0 to 11·1)	8·4 (6·1 to 11·0)	190	1·4 (−0·3 to 2·9)	20·3 (−3·9 to 47·7)
Kenya	8·0 (5·9 to 10·6)	8·4 (6·2 to 10·8)	8·8 (6·6 to 11·5)	8·7 (6·3 to 11·4)	9·6 (7·1 to 12·5)	9·5 (6·9 to 12·4)	143	1·4 (−0·1 to 2·8)	18·2 (−0·9 to 38·8)
Lesotho	7·3 (5·4 to 9·6)	7·2 (5·3 to 9·4)	7·8 (5·6 to 10·2)	7·6 (5·6 to 10·0)	8·1 (5·8 to 10·7)	8·1 (5·7 to 10·6)	193	0·8 (−0·7 to 2·2)	10·9 (−9·4 to 32·1)
Liberia	7·1 (5·1 to 9·4)	8·7 (6·3 to 11·5)	8·3 (6·0 to 11·0)	7·9 (5·7 to 10·5)	8·9 (6·5 to 11·8)	8·9 (6·3 to 11·7)	171	1·7 (0·2 to 3·2)	24·8 (3·0 to 51·0)
Madagascar	6·4 (4·5 to 8·5)	7·3 (5·4 to 9·5)	7·6 (5·5 to 9·9)	7·6 (5·5 to 9·9)	7·7 (5·5 to 9·9)	7·7 (5·5 to 9·9)	200	1·2 (−0·1 to 2·7)	19·9 (−1·1 to 47·6)
Malawi	5·7 (4·1 to 7·8)	7·4 (5·4 to 9·8)	7·8 (5·7 to 10·4)	6·7 (4·8 to 9·0)	8·0 (5·6 to 10·7)	8·0 (5·6 to 10·5)	194	2·3 (0·6 to 3·9)	40·7 (10·1 to 75·6)
Mali	7·0 (4·9 to 9·2)	8·7 (6·4 to 11·3)	8·8 (6·5 to 11·5)	8·9 (6·6 to 11·6)	9·3 (6·9 to 12·1)	9·3 (6·8 to 12·4)	151	2·3 (0·7 to 3·9)	34·1 (10·1 to 62·4)
Mauritania	8·0 (5·8 to 10·5)	8·5 (6·3 to 11·2)	8·7 (6·4 to 11·5)	8·8 (6·5 to 11·6)	8·9 (6·6 to 11·6)	9·0 (6·6 to 11·6)	165	1·0 (−0·3 to 2·4)	12·9 (−3·5 to 33·2)
Mozambique	7·9 (5·6 to 10·3)	8·7 (6·3 to 11·3)	9·3 (6·9 to 12·2)	8·8 (6·5 to 11·5)	9·6 (7·0 to 12·9)	9·8 (7·0 to 13·1)	130	1·9 (0·3 to 3·7)	24·3 (3·1 to 48·9)
Namibia	7·8 (6·0 to 10·1)	8·3 (6·3 to 10·7)	8·7 (6·7 to 11·3)	7·5 (5·7 to 9·7)	9·0 (6·8 to 11·6)	9·3 (6·9 to 11·9)	154	1·4 (0·5 to 2·4)	18·5 (6·6 to 31·4)
Niger	5·6 (3·7 to 7·6)	7·1 (5·3 to 9·4)	7·5 (5·6 to 9·8)	7·8 (5·7 to 10·0)	7·8 (5·6 to 10·3)	7·8 (5·5 to 10·3)	198	2·2 (0·6 to 3·8)	41·9 (9·5 to 84·6)
Nigeria	7·3 (5·2 to 9·8)	8·2 (6·0 to 10·8)	9·0 (6·7 to 11·7)	9·3 (6·9 to 12·0)	9·4 (6·9 to 12·3)	9·3 (6·8 to 12·2)	152	2·0 (0·4 to 3·6)	28·5 (5·7 to 53·2)
Rwanda	5·6 (3·9 to 7·6)	8·3 (6·1 to 10·8)	8·4 (6·3 to 10·8)	8·2 (6·0 to 10·7)	8·5 (6·2 to 11·1)	8·4 (6·1 to 11·0)	185	2·8 (1·2 to 4·3)	52·4 (19·9 to 91·7)
Senegal	8·1 (5·9 to 10·6)	9·3 (6·9 to 12·1)	9·5 (7·1 to 12·3)	9·5 (7·0 to 12·4)	10·0 (7·2 to 13·0)	9·8 (7·1 to 12·8)	125	1·8 (0·3 to 3·2)	22·3 (4·4 to 42·6)
Sierra Leone	6·5 (4·6 to 8·6)	7·4 (5·3 to 9·7)	8·0 (5·7 to 10·5)	8·2 (5·9 to 10·7)	8·2 (5·8 to 10·8)	8·2 (5·7 to 10·6)	192	1·7 (0·2 to 3·3)	26·7 (2·6 to 56·5)
Somalia	7·1 (5·0 to 9·5)	6·8 (4·7 to 9·1)	8·9 (6·6 to 11·5)	8·3 (6·0 to 11·0)	9·2 (6·5 to 12·2)	9·1 (6·7 to 11·9)	161	2·0 (0·4 to 3·6)	28·7 (6·1 to 55·2)
South Africa	9·7 (7·0 to 12·7)	8·0 (6·0 to 10·3)	9·4 (7·1 to 12·0)	8·9 (6·8 to 11·3)	9·9 (7·5 to 12·6)	10·0 (7·5 to 12·7)	123	0·3 (−0·7 to 1·3)	3·6 (−7·3 to 15·1)
South Sudan	6·9 (4·9 to 9·3)	7·7 (5·6 to 10·0)	7·9 (5·8 to 10·4)	8·2 (6·0 to 10·8)	8·1 (5·8 to 10·8)	7·9 (5·7 to 10·6)	197	1·0 (−0·6 to 2·5)	15·2 (−7·2 to 43·1)
São Tomé and Príncipe	8·2 (6·1 to 10·8)	8·4 (6·2 to 11·0)	8·5 (6·3 to 11·0)	8·8 (6·5 to 11·3)	9·2 (6·8 to 11·9)	9·3 (6·6 to 12·1)	153	1·0 (−0·3 to 2·4)	13·0 (−3·5 to 31·7)
Tanzania	7·7 (5·8 to 10·2)	8·4 (6·2 to 11·0)	9·1 (6·8 to 12·0)	8·8 (6·5 to 11·5)	9·6 (7·0 to 12·5)	9·5 (7·0 to 12·5)	138	1·8 (0·4 to 3·3)	23·8 (5·2 to 46·9)
The Gambia	8·2 (6·1 to 10·7)	9·0 (6·6 to 11·8)	8·7 (6·4 to 11·4)	8·8 (6·5 to 11·6)	9·3 (6·8 to 12·2)	9·4 (6·9 to 12·2)	150	1·1 (−0·2 to 2·6)	14·1 (−2·8 to 33·5)
Togo	7·7 (5·7 to 10·2)	8·1 (6·0 to 10·5)	8·7 (6·6 to 11·3)	8·8 (6·6 to 11·5)	9·0 (6·5 to 11·8)	9·0 (6·3 to 11·9)	164	1·3 (−0·1 to 2·7)	16·9 (−2·0 to 37·1)
Uganda	6·6 (4·7 to 8·8)	7·9 (5·8 to 10·2)	8·4 (6·2 to 11·0)	7·9 (5·8 to 10·5)	8·7 (6·3 to 11·4)	8·7 (6·4 to 11·4)	178	2·2 (0·6 to 3·6)	33·6 (9·3 to 60·9)
Zambia	6·4 (4·5 to 8·4)	7·4 (5·5 to 9·9)	7·9 (5·8 to 10·5)	7·5 (5·4 to 9·9)	8·4 (6·0 to 11·2)	8·4 (5·9 to 11·0)	189	1·9 (0·3 to 3·6)	31·0 (4·7 to 63·2)
Zimbabwe	8·8 (6·4 to 11·4)	7·9 (5·9 to 10·2)	8·9 (6·6 to 11·6)	8·2 (5·9 to 11·0)	9·4 (6·8 to 12·3)	9·4 (6·8 to 12·2)	144	0·6 (−0·6 to 2·1)	7·6 (−7·9 to 24·8)
**Low SDI**	**7·5 (5·7 to 9·8)**	**8·6 (6·6 to 11·1)**	**9·1 (7·0 to 11·8)**	**9·0 (6·9 to 11·5)**	**9·5 (7·2 to 12·2)**	**9·4 (7·0 to 12·1)**	..	**1·9 (1·1 to 2·6)**	**24·9 (15·8 to 34·8)**
**Low-middle SDI**	**8·4 (6·1 to 10·8)**	**9·4 (7·2 to 12·0)**	**9·7 (7·3 to 12·7)**	**9·6 (7·3 to 12·4)**	**10·1 (7·6 to 13·0)**	**10·1 (7·6 to 13·0)**	..	**1·7 (0·8 to 2·6)**	**20·6 (10·2 to 32·3)**
**Middle SDI**	**8·7 (6·6 to 11·1)**	**9·3 (7·1 to 11·8)**	**9·6 (7·3 to 12·2)**	**9·4 (7·1 to 12·0)**	**9·9 (7·5 to 12·6)**	**9·9 (7·5 to 12·8)**	..	**1·2 (0·6 to 1·9)**	**14·1 (7·4 to 20·7)**
**High-middle SDI**	**8·7 (6·6 to 11·1)**	**10·0 (7·6 to 12·7)**	**10·6 (8·1 to 13·6)**	**10·4 (8·0 to 13·3)**	**10·9 (8·3 to 13·9)**	**11·0 (8·3 to 14·1)**	..	**2·3 (1·4 to 3·2)**	**26·1 (18·0 to 35·1)**
**High SDI**	**9·7 (7·4 to 12·4)**	**10·8 (8·3 to 13·7)**	**11·3 (8·7 to 14·4)**	**11·3 (8·7 to 14·4)**	**11·5 (8·8 to 14·6)**	**11·7 (8·9 to 14·8)**	..	**1·9 (1·4 to 2·7)**	**20·1 (15·5 to 25·0)**

* The United Nations recognises Taiwan as a province of China.

The magnitude of change in the morbidity gap varied substantially by GBD super-region, though all seven super-regions saw an increase in both years and proportion of life expectancy spent in poor health (figure 1A; table). In 2023, the high-income super-region had the largest morbidity gap at 12·7 years (95% UI 9·8–16·1), equating to 15·7% of life spent in poor health (versus 13·9% in 1990). Since 1990, the morbidity gap in this super-region increased by 2·1 years (1·6–2·7), or 20·1% (16·7–23·7). By contrast, the sub-Saharan African super-region had the smallest morbidity gap in 2023 at 9·0 years (6·8–11·5; 14·0% of life expectancy, versus 13·9% in 1990). Between 1990 and 2023, the morbidity gap in sub-Saharan Africa increased by 1·7 years (1·0–2·4), or 23·7% (15·1–33·5).

The greatest morbidity gaps at the national level in 2023 were in the USA at 14·0 years (95% UI 10·8–17·5), Australia at 13·9 years (10·6–17·5), and Canada at 13·7 years (10·5–17·2; table; appendix 1 p 5). Conversely, the smallest morbidity gaps were in Nauru at 6·9 years (5·0–9·2), the Solomon Islands at 7·3 years (5·3–9·5), and Laos at 7·4 years (5·1–9·8; table). From 1990 to 2023, 203 of 204 countries and territories (99·5%) experienced an increase in the morbidity gap, with changes ranging from –0·3 years (–1·6 to 1·0) in Palestine (the only country that did not have a positive change) to 3·0 years (1·9–4·0) in Kuwait and 2·9 years (2·1–3·8) in Australia.

The proportion of life spent in poor health (ie, proportional morbidity gap) increased in 173 countries and territories (84·8%) over the study period (appendix 1 p 6). This included all high-income countries. The Netherlands experienced the largest absolute increase, going from 13·2% of life expectancy in 1990 to 15·7% in 2023. In 2023, the largest proportional morbidity gaps were in Afghanistan (17·9% of life expectancy) and the USA (17·8% of life expectancy).

In both years analysed (1990 and 2023), there was a clear relationship between the morbidity gap and life expectancy, with a higher morbidity gap strongly associated with higher life expectancy (figure 2). Higher SDI countries tended to have both longer life expectancy and more years in poor health. An association between life expectancy and the proportional morbidity gap was less clear, suggesting that although longer-lived populations experience more years of morbidity, the proportional burden is less related to development level.

**Figure 2:**
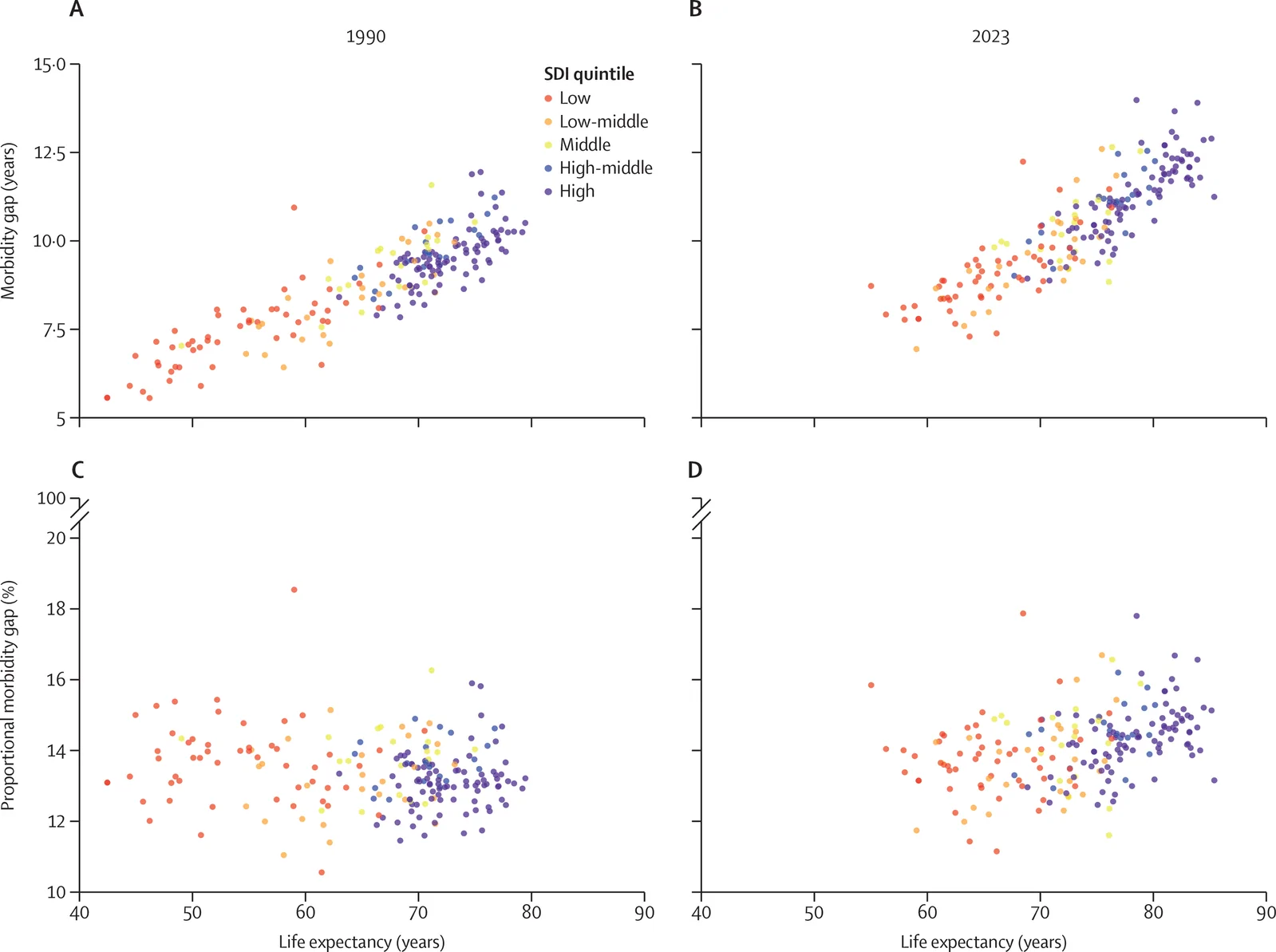
Comparison of the morbidity gap (A, B) and proportional morbidity gap (C, D) versus life expectancy across countries and territories, 1990 (A, C) and 2023 (B, D) SDI=Socio-demographic Index.

The morbidity gap varied substantially across SDI quintiles (figure 1B; table). In 2023, the high SDI group exhibited the largest morbidity gap, at 11·7 years (95% UI 8·9–14·8), an increase of 1·9 years (1·4–2·7) from 1990 (a 20·1% [15·5–25·0] increase). This socio-demographic group also had the largest proportional morbidity gap in 2023, with 14·6% of life expectancy spent in poor health, an increase of 1·2 percentage points from 1990. Conversely, the low SDI quintile had the smallest morbidity gap in 2023, at 9·4 years (7·0–12·1). Although this finding reflected an expansion of morbidity in absolute terms—the low SDI morbidity gap increased by 1·9 years (1·1–2·6) from 1990, a 24·9% (15·8–34·8) increase—the proportional morbidity gap in low SDI only changed from 14·1% in 1990 to 14·2% in 2023. Between 1990 and 2023, the high-middle SDI quintile experienced the largest increase in morbidity gap, with an increase of 2·3 years (1·4–3·2, or 26·1% [18·0–35·1]), whereas the middle SDI group had the smallest increase at 1·2 years (0·6–1·9; 14·1% [7·4–20·7]).

Between 1990 and 2023, the age-specific morbidity gap (represented by the area between the survival probability and health-adjusted survival probability curves in figure 3) both expanded across ages and shifted towards older age groups globally and across super-regions and SDI quintiles. For example, when considering the global curves (figures 3A–B), the morbidity gap was markedly wider in 2023 than in 1990 across the adult life course, whereas the age groups with the widest morbidity gap were likewise older in 2023 than in 1990. These findings demonstrate that although the greatest concentration of disability is shifting to older ages over time, it is still expanding across age groups, with non-fatal health loss continuing to accumulate over the adult life course, not just in the oldest ages. The shape of the curves varied by location, with the largest concentration of health loss occurring in older ages in higher SDI settings than in lower ones, but the pattern of widening morbidity gaps between 1990 and 2023 across adult ages was consistent across most locations, including at the national level (appendix 2 pp 7–109).

**Figure 3:**
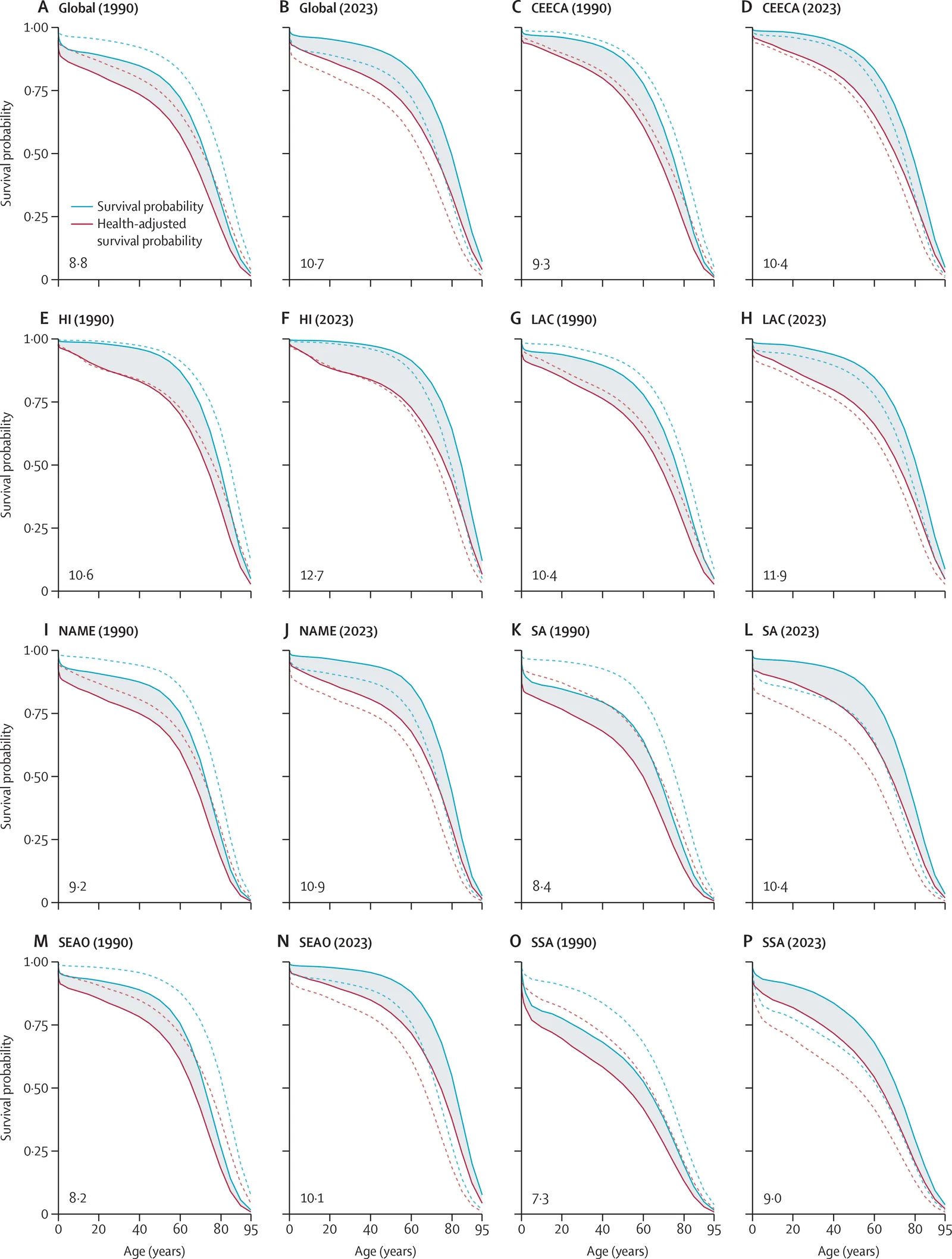

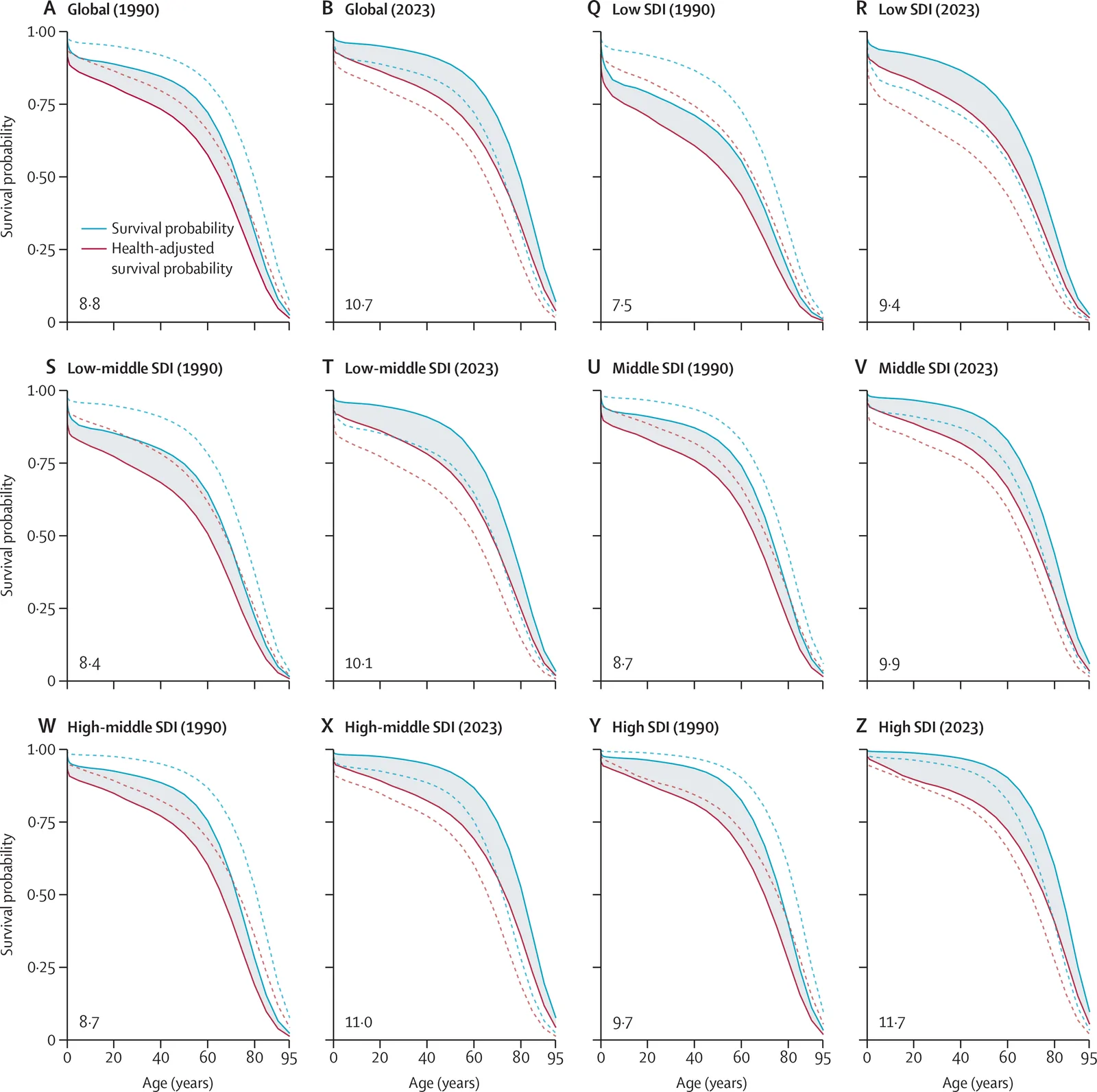
Survival and health-adjusted survival probability by age, globally and by GBD super-region (top plot) and SDI quintile (bottom plot), 1990 and 2023 Survival probability (blue) and health-adjusted survival probability (red) are shown by single year of age from birth to 95 years for the global population (A, B), seven GBD super-regions (C–P), and SDI quintiles (Q–Z). Panels show estimates for 1990 and 2023; dashed curves are included to aid visual comparison between time periods. The grey shaded area between the survival and health-adjusted survival curves represents the morbidity gap across the life course. Numbers in the lower left of each panel indicate the average morbidity gap in years. CEECA=central Europe, eastern Europe, and central Asia. GBD=Global Burden of Diseases, Injuries, and Risk Factors Study. HI=high income. LAC=Latin America and Caribbean. NAME=north Africa and Middle East. SA=south Asia. SDI= Socio-demographic Index. SEAO=southeast Asia, east Asia and Oceania. SSA=sub-Saharan Africa.

With respect to cause-group contributions to the morbidity gap in 2023, the following top five cause groups, largely composed of non-fatal conditions, together accounted for 57·4% of the global morbidity gap; musculoskeletal disorders (namely low back pain; appendix 1 p 6) were the single largest contributor at 1·8 years (95% UI 1·2–2·6) of life spent in poor health, followed by mental disorders (namely depressive disorders and anxiety disorders) at 1·6 years (1·1–2·2), sense organ diseases (notably age-related hearing loss) at 1·1 years (0·8–1·5), unintentional injuries (particularly falls) at 0·9 years (0·6–1·2), and other non-communicable diseases at 0·8 years (0·6–1·1; figure 4). In the high-income super-region, musculoskeletal disorders and mental disorders together accounted for more than one-third of the morbidity gap, with additional contributions from unintentional injuries, neurological disorders, and sense organ diseases collectively representing 61·8% of the 12·7-year morbidity gap. High SDI settings had similar contributions to the morbidity gap (appendix 1 p 7). In sub-Saharan Africa, although musculoskeletal and mental disorders remained the leading contributors, nutritional deficiencies, respiratory infections and tuberculosis, and neglected tropical diseases and malaria were also among the ten leading contributors. Nutritional deficiencies were likewise a top five contributor in low SDI settings (appendix 1 p 7). These patterns indicate that although chronic non-fatal conditions dominate the morbidity gap globally, infectious and nutritional disorders that also cause considerable fatal burden still contribute substantially to non-fatal health loss in some lower SDI contexts, particularly in sub-Saharan Africa.

**Figure 4:**
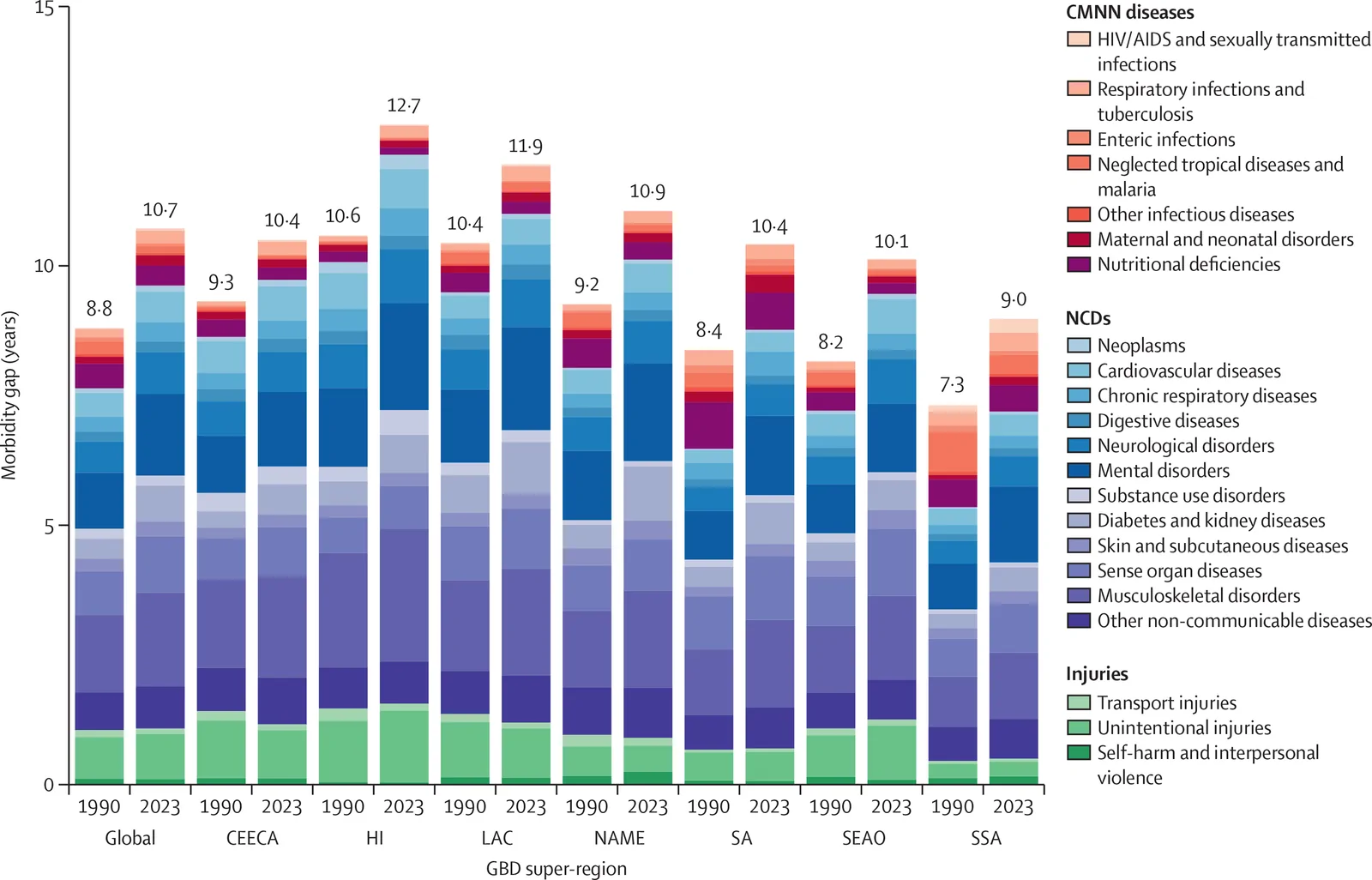
GBD Level 2 cause decomposition of the morbidity gap globally and by GBD super-region, 1990 and 2023 Total morbidity gap values (years) are displayed above each bar. GBD categorises causes into a four-level hierarchy. Level 1 includes three broad cause categories (eg, NCDs), whereas Levels 2–4 are composed of increasingly specific cause groups and causes (eg, Level 2: mental disorders; Level 3: depressive disorders; and Level 4: major depressive disorder). CEECA=central Europe, eastern Europe, and central Asia. CMNN=communicable, maternal, neonatal, and nutritional. GBD=Global Burden of Diseases, Injuries, and Risk Factors Study. HI=high income. LAC=Latin America and Caribbean. NAME=north Africa and Middle East. NCDs=non-communicable diseases. SA=south Asia. SEAO= southeast Asia, east Asia and Oceania. SSA=sub-Saharan Africa.

In terms of risk factor contributions to the morbidity gap, high fasting plasma glucose, high body-mass index (BMI), child and maternal malnutrition (most notably iron deficiency; appendix 1 p 8), and tobacco use (smoking) were the leading GBD Level 2 risk factors contributing to the global morbidity gap in 2023 (figure 5). When only considering disaggregated Level 3 risk factors, particulate matter air pollution emerged as the leading contributor (appendix 1 p 8). High SDI and high-income settings were characterised by greater contributions from high BMI, high fasting plasma glucose, and tobacco use, whereas in sub-Saharan Africa and low-SDI settings, child and maternal malnutrition, high fasting plasma glucose, air pollution, and in the case of sub-Saharan Africa, high BMI were most prominent. Drug use and high alcohol use were among the ten leading risk factors contributing to the morbidity gap only in the high-income super-region, whereas unsafe sex emerged as a leading risk only in sub-Saharan Africa. These findings demonstrate that the morbidity gap is shaped by exposure to a combination of chronic metabolic and behavioural risk factors and persistent environmental and nutritional risks, with substantial variation by region and level of sociodemographic development.

**Figure 5:**
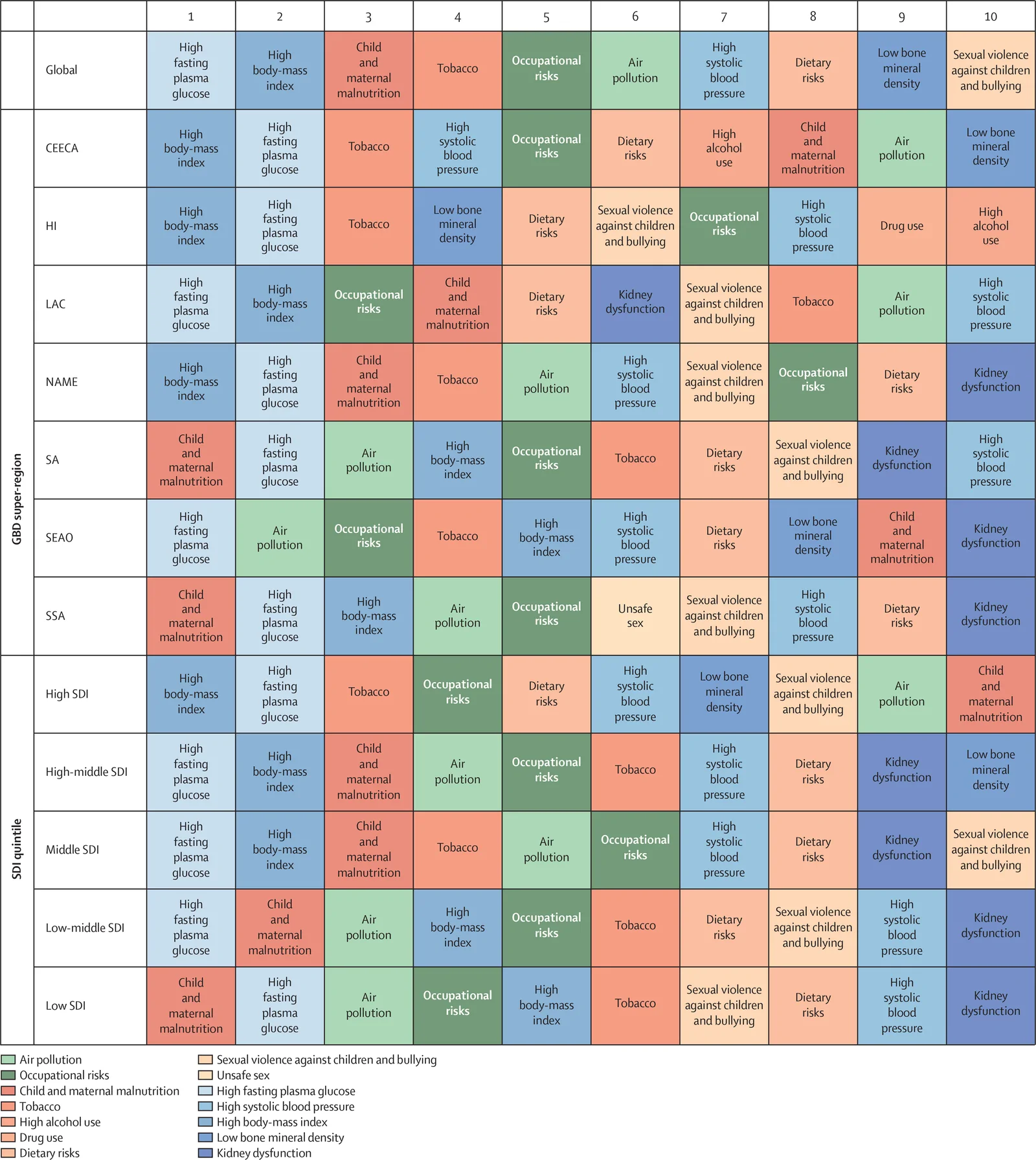
Leading ten GBD Level 2 risk factors contributing to the morbidity gap, by GBD super-region and SDI quintile, 2023 The ten leading GBD Level 2 risk factors contributing to the morbidity gap are ranked from left (highest contribution) to right (tenth highest) for the global population, each GBD super-region, and SDI quintile in 2023. Colours denote Level 2 risk factor categories. GBD categorises risk factors into a four-level hierarchy. Level 1 includes three broad cause categories (eg, behavioural risks), whereas Levels 2–4 are composed of increasingly specific risk groups and risks (eg, Level 2: child and maternal malnutrition; Level 3: low birthweight and short gestation; and Level 4: low birthweight). CEECA=central Europe, eastern Europe, and central Asia. GBD=Global Burden of Diseases, Injuries, and Risk Factors Study. HI=high income. LAC=Latin America and Caribbean. NAME=north Africa and Middle East. SA=south Asia. SDI=Socio-demographic Index. SEAO=southeast Asia, east Asia, and Oceania. SSA=sub-Saharan Africa.

With respect to sex differences in the morbidity gap, females lived longer than males and consistently spent more years in poor health across all geographies, highlighting the so-called gender health paradox^28^, ^29^ (appendix 1 pp 9, 24–53). We estimated a morbidity gap of 10·0 years (95% UI 7·6–12·8) for females (equating to 14·9% of life expectancy) and 7·6 years (5·8–9·8) for males (12·3% of life expectancy) in 1990. By 2023, the morbidity gap rose to 12·1 years (9·2–15·6), a 21·3% (14·7–28·6) increase, for females (15·9% of life expectancy) and to 9·3 years (7·0–11·9), a 22·2% (15·1–30·5) increase, for males (13·0% of life expectancy). Females experienced more years in poor health in all SDI quintiles (appendix 1 pp 24–53).

## Discussion

From 1990 to 2023, the global morbidity gap widened from nearly 9 years to almost 11 years, an increase of approximately 22%, indicating that as life expectancy increases, a growing number of years are being spent in poor health. This trend was consistent across locations, with point estimates suggesting an increasing morbidity gap in 203 of 204 countries and territories. These patterns reflect a widening morbidity gap across adult age groups, rather than a concentration of disability in the final years of life. In 2023, the morbidity gap was largest in the high SDI quintile and smallest in the low SDI quintile, reflecting differences in lifespan rather than healthier ageing. Globally, musculoskeletal disorders (eg, low back pain), mental disorders (eg, depressive disorders and anxiety disorders), sense organ diseases (eg, age-related hearing loss), unintentional injuries (eg, falls), and other non-communicable diseases together accounted for over 50% of the years lived in poor health, with nutritional disorders also contributing substantially in some lower SDI contexts. Globally, high fasting plasma glucose, high BMI, child and maternal malnutrition (especially iron deficiency), and tobacco use (particularly smoking) were leading risk factors contributing to the morbidity gap. Across geography and SDI strata, females lived longer than males but spent more years in poor health, reinforcing persistent sex differences in lifespan and healthspan.

This analysis provides a comprehensive evaluation of Fries’ hypothesis of morbidity compression,^5^, ^6^, ^7^ covering 204 countries and territories over more than three decades. Between 1990 and 2023, gains in survival consistently outpaced reductions in non-fatal health loss, leading to a rise in the morbidity gap across all GBD super-regions, SDI quintiles, and almost all countries. Our survival and health-adjusted survival curves provide complementary age-specific evidence: across regions, the separation between survival and health-adjusted survival curves persisted throughout adulthood and older ages, indicating that the expanding morbidity gap reflects progressive accumulation of non-fatal health loss from adulthood onward, rather than a concentration of morbidity in the final years of life alone*.* Notably, although our results suggest that absolute and proportional morbidity gap measures do not entirely agree—with the proportion of life expectancy spent in poor health declining in about 15% of countries, consistent with Permanyer and Bramajo's report^18^ (reinforcing that conclusions about morbidity compression depend on the metric chosen, including crucial information that can be further derived from analyses assessing variability in healthy lifespans across ages and populations)^30^, ^31^, ^32^—our overall findings indicate widespread morbidity expansion, underscoring the divergence between gains in longevity and improvements in healthspan. This is consistent with recent work by Garmany and Terzic (in 2024 and 2025),^16^, ^17^ Permanyer and Bramajo (2023),^18^ and Salomon and colleagues (2012).^19^ Taken together, this accumulating evidence of widespread morbidity expansion suggests an urgent need to develop morbidity mitigation strategies informed by the cause-related and risk-factor-related composition of the morbidity gap.

The absence of morbidity compression reflects the underlying composition of remaining disease burden. Once reductions in YLLs dominate past gains, further increases in survival will, by arithmetic necessity, translate into additional YLDs unless the incidence, severity, or duration of non-fatal conditions is reduced. Thus, interventions that substantially reduce case fatality without a commensurate reduction in disease incidence convert what were once fatal events into chronic survivorship with disability.^33^ Such shifts mechanically widen the morbidity gap, even as they represent major successes in life extension. For example, in addition to the accumulation of disability in later life, morbidity in settings such as south Asia and sub-Saharan Africa is substantially influenced by survival of early-life infectious conditions and severe childhood undernutrition that were historically highly fatal and that, when survived, often come with long-term sequelae. These conditions can result in persistent neurodevelopmental, cognitive, or sensory impairments.^34^, ^35^ Morbidity in these contexts is also increasingly influenced by the growing contribution of non-communicable diseases that emerge earlier in the life course than in higher-income settings and are associated with accelerated disease trajectories and earlier functional decline.^36^

Not only does reducing case fatality rates generally translate to greater disability from the given cause, but it also allows for the individual to experience disability from other, largely non-fatal causes by virtue of surviving. Thus, once reductions in mortality have largely exhausted causes dominated by YLLs, the remaining cause mix shifts towards chronic, non-fatal conditions in which further survival gains tend to add YLDs rather than subtract them. This pattern is shown in our cause-specific decomposition results, wherein the 2023 morbidity gap is dominated by conditions characterised by high ratios of YLDs relative to YLLs. These contrasting patterns underscore that comparable gains in longevity can give rise to markedly different morbidity trajectories, reinforcing the need for context-specific strategies to reduce disability and support healthy ageing across the life course.

This framing reconciles Fries’ hypothesis of compression, Gruenberg's thesis of expansion, and Manton's concept of dynamic equilibrium, which are often treated as competing explanations. In practice, they represent alternative outcomes of the same accounting constraint, determined by how survival gains are distributed between YLLs averted and YLDs accrued. Compression arises when survival gains are concentrated in YLL-heavy causes; expansion arises when fatal disease is converted into chronic disability; and dynamic equilibrium arises when prevalence increases but severity declines, reducing YLDs per case.

Future gains in healthy ageing will therefore depend less on further reductions in mortality and more on preventing disease onset, reducing disability severity, and shortening the duration of non-fatal conditions that dominate years lived in poor health. Economic modelling further indicates that improving health by targeting ageing could offer larger economic gains than increasing life expectancy by eliminating specific diseases.^37^ The cause and risk patterns identified here indicate that progress will require sustained investment in prevention, rehabilitation, access to assistive technologies, mental health services, and chronic care, alongside continued action on modifiable risk factors.

Decomposition analyses show that across geographic and SDI settings, but especially in high-SDI countries, musculoskeletal disorders and mental health conditions are the dominant contributors to the morbidity gap, making them clear priorities for prevention and clinical management, alongside investment in rehabilitation,^38^ access to assistive technologies,^39^ and integrated long-term care models, as articulated in WHO's healthy ageing framework.^40^ Likewise, a set of potentially modifiable risk factors—led by high BMI, high fasting plasma glucose, and tobacco use in the high-income super-region and high-SDI settings, and by child and maternal malnutrition, high fasting plasma glucose, and either high BMI or air pollution in sub-Saharan Africa, south Asia, and low-SDI settings—emerge as potential targets for interventions to reduce morbidity. These findings highlight the need for continued focus on mitigation strategies for metabolic and behavioural risks, increased investment in basic health system capacity, alongside robust preparations for the epidemiological transition to chronic non-fatal conditions.

Additionally, although our results confirm well established evidence that longevity gains are larger for females, the persistence of wider morbidity gap for females indicates that progress in healthy ageing will depend on reducing late-life disability and chronic conditions that disproportionately affect females, particularly musculoskeletal disorders and mental health conditions. The concentration of morbidity in high-income regions among mental disorders, substance use disorders, and injury-related causes likewise raises the possibility that conditions grouped as so-called diseases of despair contribute to the widening gap between lifespan and healthspan, highlighting an important priority for future disaggregated analyses and policy response.

These findings have direct relevance for monitoring progress towards SDG 3, which aims to ensure healthy lives and promote wellbeing for all, at all ages. In particular, our results inform interpretation of SDG target 3.4, which focuses on reducing premature mortality from non-communicable diseases and promoting mental health and wellbeing. Although substantial progress has been made in reducing premature mortality, assessments of progress towards SDG 3.4 based on mortality alone substantially understate the remaining disease burden. The expansion of the morbidity gap also bears directly on SDG target 3.8 on universal health coverage, highlighting unmet needs in long-term disease management, rehabilitation, chronic care, and functional support, which are central components of effective coverage across the life course. This observation implies that current Universal Health Coverage monitoring frameworks, which remain heavily weighted towards survival-focused interventions, might need recalibration to capture effective coverage for interventions that reduce YLDs. Taken together, these findings strengthen the case that progress towards SDG 3 should be judged not only by gains in longevity, but by sustained improvements in healthspan and reductions in years of life lived in poor health.

Our study has several limitations. Importantly, although our point estimates consistently indicate morbidity gap expansion across geographies, uncertainty intervals for location-specific morbidity gap estimates overlap between time points; therefore, our conclusions regarding morbidity expansion are based on the consistency and directionality of trends across locations rather than on statistically significant country-specific changes. The wide uncertainty in morbidity gap estimates reflects the considerable challenges—as noted by Permanyer and Bramajo^18^—of precisely estimating non-fatal health loss, and indeed also highlights the overall limitations associated with using measures based on comparing mean estimates of life expectancy and HALE to capture information about trends in healthy lifespans and the distribution of morbidity across ages and populations.^30^, ^31^, ^32^ First, estimates of HALE and the morbidity gap rely on YLDs derived from disability weights that are based on population surveys and modelling, which might not fully capture variation in health perceptions, cultural context, or severity of disability across settings and over time. Second, temporal YLD trends might also reflect improvements in diagnostic technology, expanded screening, and evolving disease classification criteria, in addition to true morbidity. GBD tries to account for these detection artifacts using crosswalking, adjustment tools (MR-BRT), and a tool to model temporal trends (DisMod-AT), but accurately quantifying morbidity trends over time remains a challenge. Third, although GBD 2023 incorporates extensive data sources and rigorous modelling, data availability and quality remain uneven across countries and causes, with particularly limited data in low-income settings. Greater reliance on statistical models results in wider uncertainty around estimates for data-poor populations. Fourth, the morbidity gap is a summary measure that does not capture heterogeneity in the timing, severity, or clustering of disability across the life course (eg, years of morbidity could be distributed uniformly across the lifespan or compressed into the final years of life, although age-specific patterns are captured in the survival probability curves), or differences in comorbidity patterns within populations. Fifth, cause and risk factor decompositions attribute years lived in poor health based on proportional contributions and do not imply causality or reflect the dynamic interactions between conditions and risk exposures. Sixth, our analysis is based on aggregated national and regional estimates and therefore masks important and potentially substantial subnational, socioeconomic, and within-country inequalities in healthspan and morbidity. Finally, our estimates reflect population-level trends, not individual experiences, with some individuals experiencing far more or fewer years of poor health than the mean.

Importantly, our study highlights several priorities for research and policy to understand and reduce the years lived in poor health. First, improved measurement of morbidity is essential: disability weights and HALE estimates rely on self-reported data that might not fully capture differences in health perception or cultural context, particularly in low- and middle-income countries. Strengthening surveillance systems and expanding population health surveys would reduce dependence on statistical modelling and improve the sensitivity of healthspan metrics to changes in disability severity over time. Second, finer-grained analyses are needed to assess which interventions are most effective at addressing the major contributors to the morbidity gap, particularly as non-fatal conditions now dominate unhealthy years in most populations. Third, the persistence of inequalities between SDI groups indicates a need for further research on the structural determinants of healthspan, including socioeconomic conditions, access to care, and health system resilience. Fourth, the COVID-19 pandemic underscores the value of tracking morbidity and healthspan metrics in near real time to capture shocks and recovery trajectories. Finally, decompositions of the morbidity gap by location can be extended to scenario modelling to assess the extent to which specific interventions, risk factor reductions, or innovations could alter the structural bounds of compression of morbidity.

In conclusion, this study assesses the compression of morbidity hypothesis using estimates of life expectancy and HALE to calculate the morbidity gap for 204 countries and territories from 1990 to 2023. Although longevity has increased substantially over the past three decades, these gains have not been accompanied by commensurate reductions in the years lived in poor health. Instead, the morbidity gap has expanded in almost all countries, so that most populations now experience a decade or more of life with disability. This expansion reflects the growing dominance of chronic, largely non-fatal conditions, particularly musculoskeletal, mental, and sensory disorders. By decomposing the morbidity gap into its leading causes and risk factors, this analysis shows that once causes dominated by YLLs have been reduced, further gains in survival increasingly translate into YLDs unless the severity, duration, or incidence of non-fatal conditions is reduced through investment in prevention efforts, clinical management, rehabilitation services, and more. These findings have implications for labour markets, social protection systems, and the built environment in ageing societies and demonstrate that progress in healthy ageing should be evaluated primarily by gains in healthspan, not lifespan. This approach will require a fundamental reorientation of global health priorities towards preventing and mitigating the conditions accounting for the largest share of unhealthy years, alongside sustained action on modifiable risk factors.

### Contributors

### Data sharing

For detailed information on GBD 2023 data sources and estimates, please visit the Global Health Data Exchange GBD 2023 website at http://ghdx.healthdata.org/gbd-2023. Metadata and citations for GBD 2023 input data are available in the GBD 2023 Sources Tool (https://sources.healthdata.org/collection/sources-2023), whereas GBD 2023 estimates used in the analysis in this study, including estimates of life expectancy and healthy life expectancy, are available to view and download in the GBD Results Tool (https://vizhub.healthdata.org/gbd-results/).

## Declaration of interests

This work was funded by the Gates Foundation. Funding is paid to the authors’ institution and the funder had no role in the development of this Article. SN declares grant funding from the Ministry of Education, Culture, Sports, Science and Technology of Japan (24H00663). All other authors declare no competing interests.
