# Supplementary material for: Global, regional, and national trends in the morbidity gap and contributing diseases, injuries, and risk factors, 1990–2023: a systematic analysis for the Global Burden of Disease Study 2023
Source: Lancet Public Health. 2026 Jul 21;11(8):e487–505. doi: 10.1016/S2468-2667(26)00098-8 (PMC13752251; doi:10.1016/S2468-2667(26)00098-8)
Supplement: Supplementary file 1 [file mmc1.pdf]

THE LANCET
Public Health

Supplementary appendix 1

This appendix formed part of the original submission and has been peer reviewed. We post it as supplied by the authors.

Supplement to: Hay SI, Nam P, Saqib H, et al. Global, regional, and national trends in the morbidity gap and contributing diseases, injuries, and risk factors, 1990–2023: a systematic analysis for the Global Burden of Disease Study 2023. *Lancet Public Health* 2026; **11:** e487–505.

Appendix 1: Supplementary figures and tables for "Global, regional, and national trends in the morbidity gap and contributing diseases, injuries, and risk factors, 1990–2023: a systematic analysis for the Global Burden of Disease Study 2023"

Table of Contents

Appendix 1 figures....3

Figure S1. Life expectancy and healthy life expectancy globally and by GBD super-region, 1990–2023 4

Figure S2. Global distribution of life expectancy, healthy life expectancy, and the morbidity gap, 1990 and 2023 5

Figure S3. Leading ten GBD Level 3 causes contributing to the morbidity gap, by GBD super-region and Socio-demographic Index quintile, 2023 6

Figure S4. Leading ten GBD Level 2 causes contributing to the morbidity gap by GBD super-region and Socio-demographic Index quintile, 2023 7

Figure S5. Leading ten GBD Level 3 risk factors contributing to the morbidity gap, by GBD super-region and Socio-demographic Index quintile, 2023 8

Figure S6. Sex differences in the morbidity gap globally and by GBD super-region, 1990–2023 9

Appendix 1 tables 10

Table S1: Life expectancy, 1990, 2010, 2020, 2021, 2022, and 2023, and absolute and relative changes, 1990–2023, for all sexes combined, globally, by SDI quintile, and for GBD super-regions and countries and territories....10

Table S2: Healthy life expectancy, 1990, 2010, 2020, 2021, 2022, and 2023, and absolute and relative changes, 1990–2023, for all sexes combined, globally, by SDI quintile, and for GBD super-regions and countries and territories....14

Table S3: Proportional morbidity gap (MG), 1990 and 2023, and absolute change, 1990–2023, for all sexes combined, globally, by SDI quintile, and for GBD super-regions and countries and territories....18

Table S4: Male life expectancy, 1990, 2010, 2020, 2021, 2022, and 2023, and absolute and relative changes, 1990–2023, globally, by SDI quintile, and for GBD super-regions and countries and territories....24

Table S5: Male healthy life expectancy, 1990, 2010, 2020, 2021, 2022, and 2023, and absolute and relative changes, 1990–2023, globally, by SDI quintile, and for GBD super-regions and countries and territories....29

Table S6: Male morbidity gap, 1990, 2010, 2020, 2021, 2022, and 2023, and absolute and relative changes, 1990–2023, globally, by SDI quintile, and for GBD super-regions and countries and territories....34

Table S7: Female life expectancy, 1990, 2010, 2020, 2021, 2022, and 2023, and absolute and relative changes, 1990–2023, globally, by SDI quintile, and for GBD super-regions and countries and territories....39

Table S8: Female healthy life expectancy, 1990, 2010, 2020, 2021, 2022, and 2023, and absolute and relative changes, 1990–2023, globally, by SDI quintile, and for GBD super-regions and countries and territories....44

Table S9: Female morbidity gap, 1990, 2010, 2020, 2021, 2022, and 2023, and absolute and relative changes, 1990–2023, globally, by SDI quintile, and for GBD super-regions and countries and territories....49

Appendix 1 figures

Figure S1. Life expectancy and healthy life expectancy, globally and by GBD super-region, 1990–2023

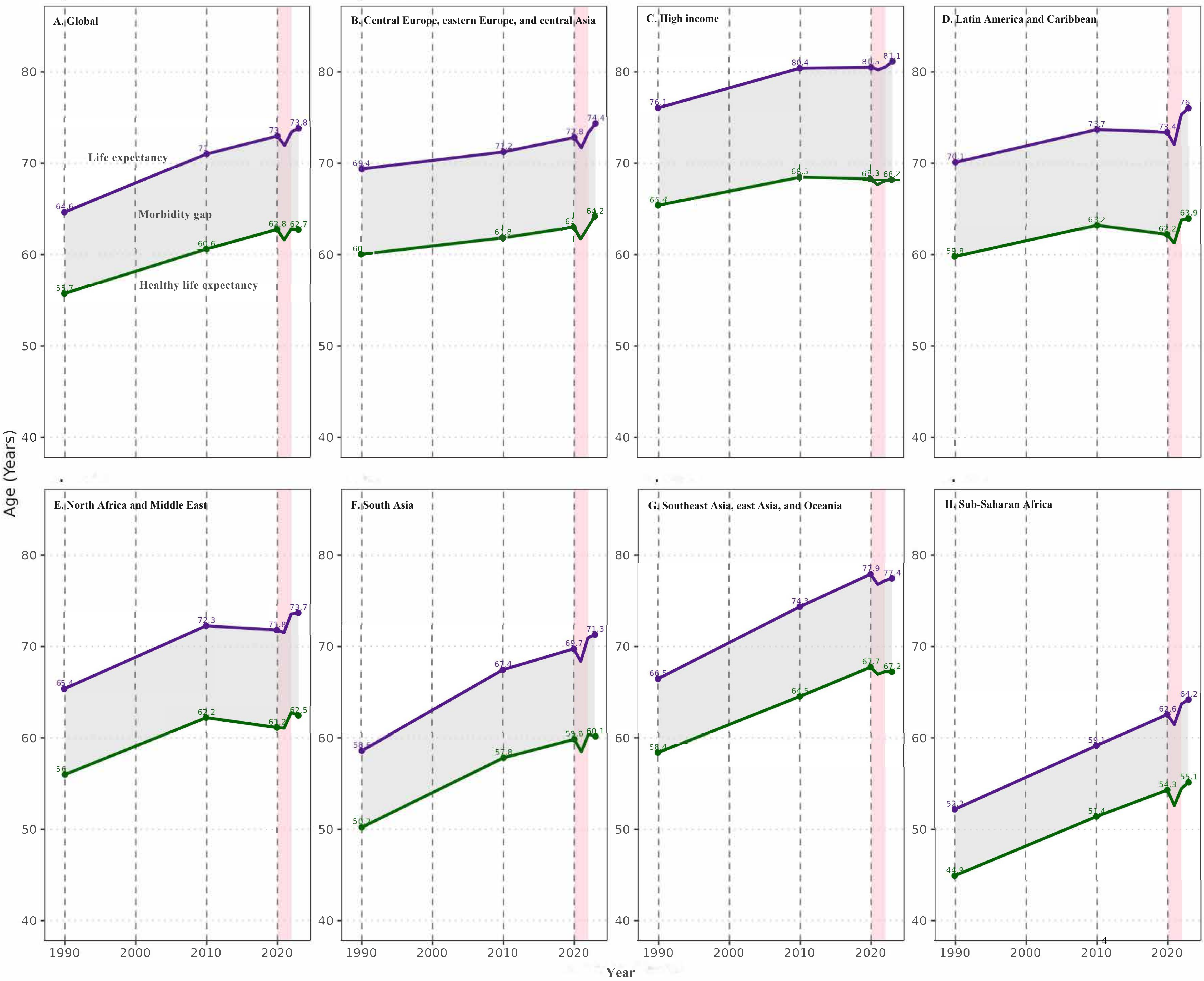

4

Figure S2. Global distribution of life expectancy, healthy life expectancy, and the morbidity gap, 1990 and 2023

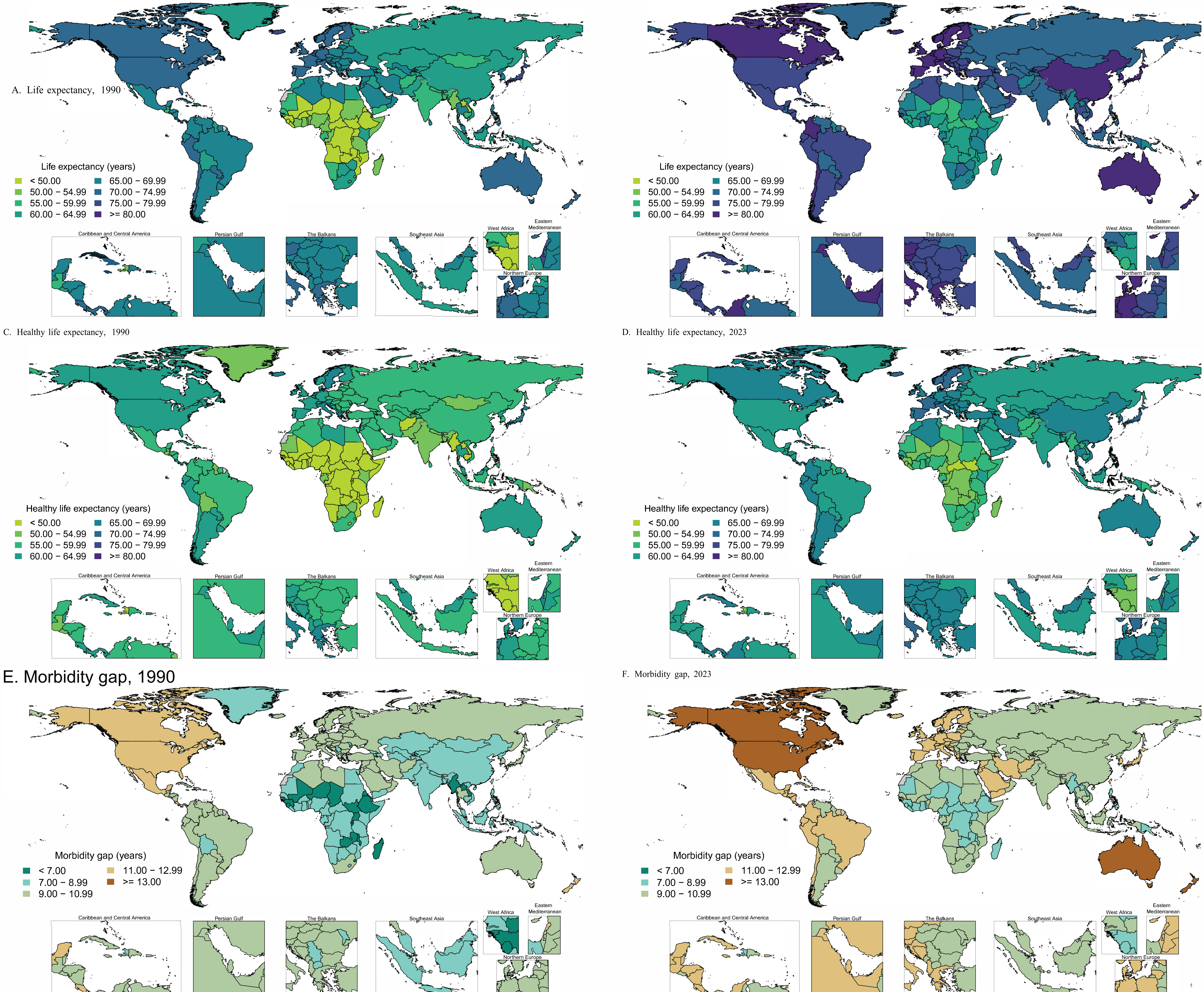

Figure S3. Leading ten GBD Level 3 causes contributing to the morbidity gap, by GBD super-region and Socio-demographic Index quintile, 2023

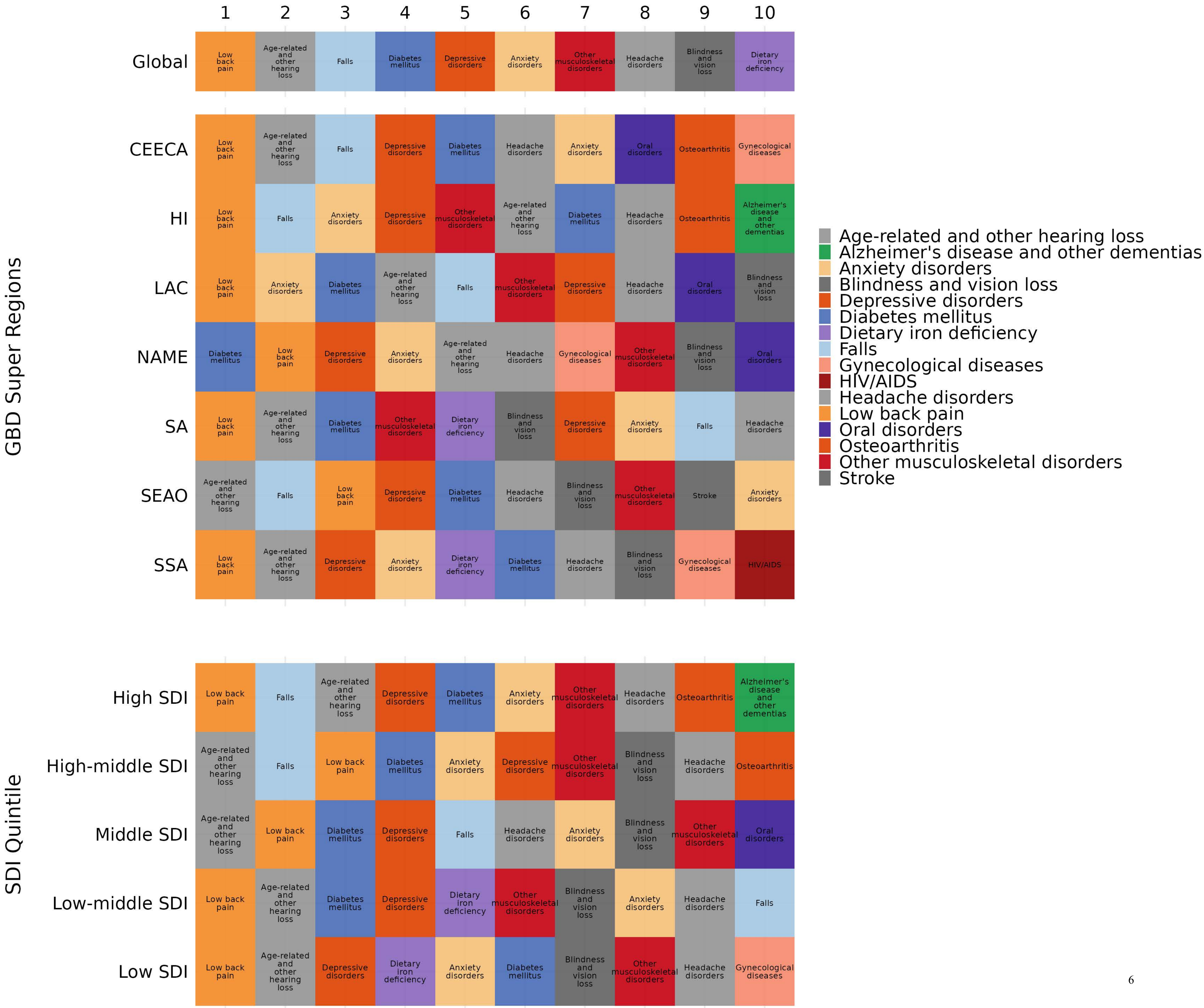

Figure S4. Leading ten GBD Level 2 causes contributing to the morbidity gap by GBD super-region and Socio-demographic Index quintile, 2023

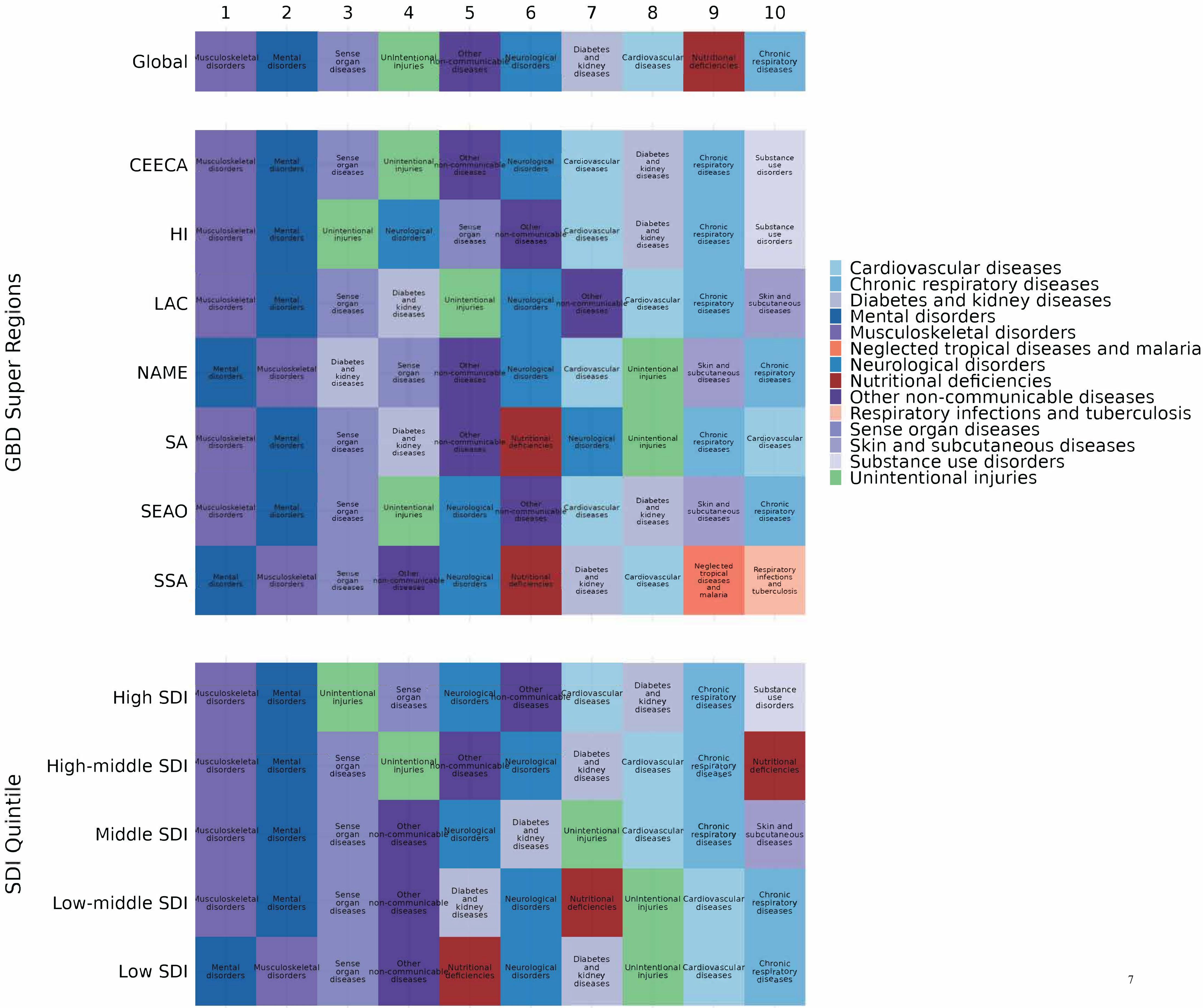

Figure S5. Leading ten GBD Level 3 risk factors contributing to the morbidity gap, by GBD super-region and Socio-demographic Index quintile, 2023

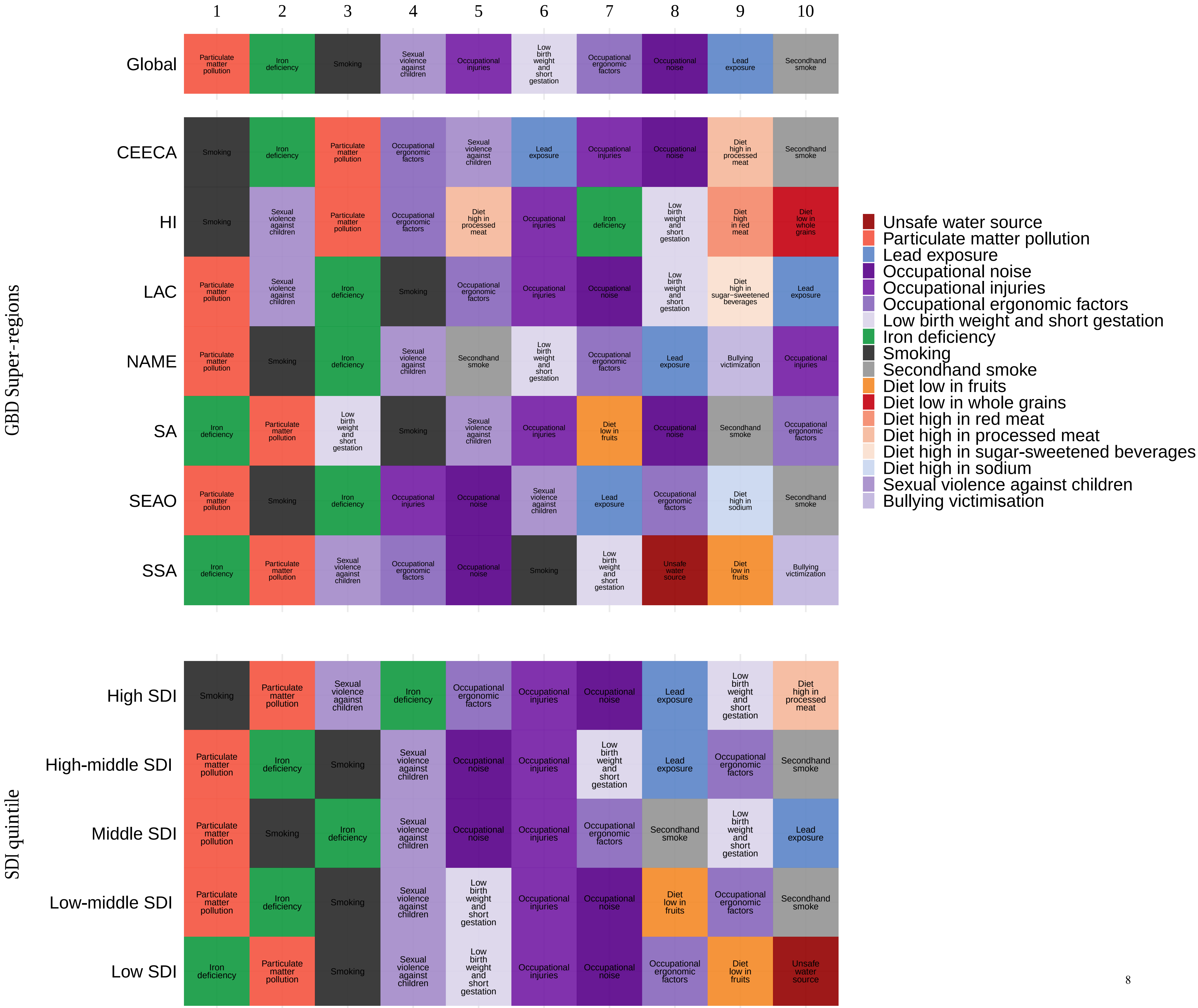

Figure S6. Sex differences in the morbidity gap globally and by GBD super-region, 1990–2023

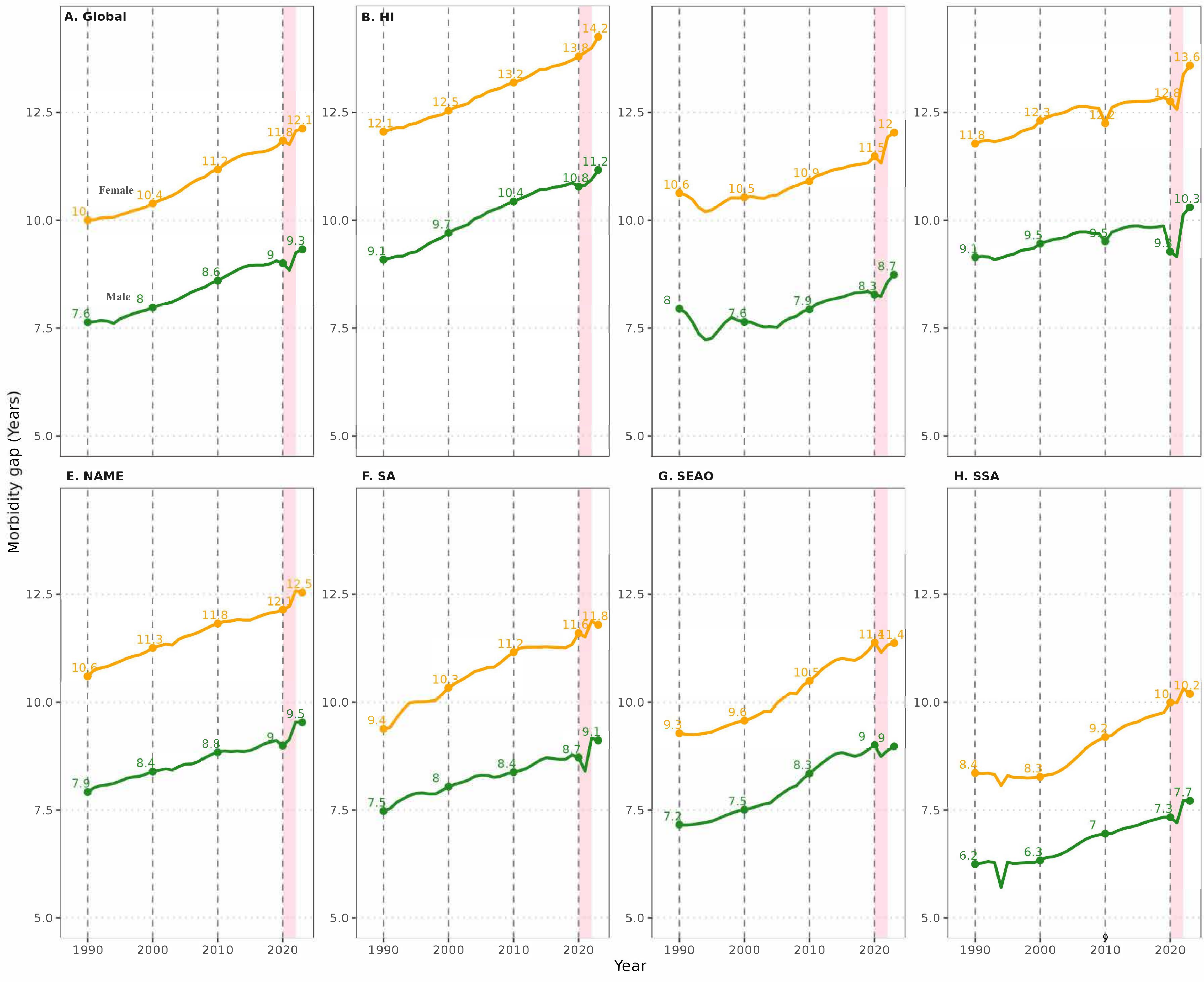

Table S1: Life expectancy, 1990, 2010, 2020, 2021, 2022, and 2023, and absolute and relative changes, 1990–2023, for all sexes combined, globally, by SDI quintile, and for GBD super-regions and countries and territories

Location	1990	2010	2020	2021	2022	2023	Rank (2023)	Absolute Change (1990-2023)	Relative Change, % (1990-2023)
**Global**	**64·6 (64·5 to 64·8)**	**71·0 (70·9 to 71·1)**	**73·0 (72·9 to 73·0)**	**71·9 (71·9 to 72·0)**	**73·4 (73·2 to 73·6)**	**73·8 (73·6 to 74·1)**		**9·2 (8·9 to 9·5)**	**14·2 (13·8 to 14·7)**
**Central Europe, Eastern Europe, and Central Asia**	**69·4 (69·3 to 69·4)**	**71·2 (71·2 to 71·3)**	**72·8 (72·8 to 72·8)**	**71·7 (71·7 to 71·7)**	**73·3 (72·9 to 73·6)**	**74·4 (74·1 to 74·5)**		**5·0 (4·7 to 5·2)**	**7·2 (6·8 to 7·4)**
Albania	71·9 (71·5 to 72·2)	78·1 (77·7 to 78·5)	77·4 (77·2 to 77·6)	76·9 (76·7 to 77·1)	78·7 (78·3 to 79·1)	79·6 (79·2 to 80·1)	42	7·8 (7·2 to 8·4)	10·8 (10·0 to 11·7)
Armenia	70·4 (70·1 to 70·8)	74·0 (73·7 to 74·4)	70·7 (70·4 to 71·1)	75·0 (74·8 to 75·2)	77·6 (77·2 to 78·1)	79·6 (79·1 to 80·1)	43	9·2 (8·5 to 9·8)	13·0 (12·1 to 13·9)
Azerbaijan	66·1 (65·8 to 66·5)	71·5 (71·2 to 71·7)	70·1 (69·9 to 70·3)	70·9 (70·7 to 71·1)	73·1 (72·7 to 73·5)	72·6 (72·1 to 73·1)	120	6·4 (5·8 to 7·1)	9·7 (8·8 to 10·7)
Belarus	71·0 (70·8 to 71·1)	70·6 (70·4 to 70·7)	74·2 (74·0 to 74·5)	73·9 (73·5 to 74·2)	73·8 (73·4 to 74·2)	74·5 (74·0 to 74·9)	104	3·5 (3·0 to 4·0)	5·0 (4·3 to 5·6)
Bosnia and Herzegovina	72·4 (72·2 to 72·6)	76·2 (76·0 to 76·4)	75·8 (75·7 to 75·9)	74·6 (74·5 to 74·7)	76·6 (76·3 to 76·8)	77·7 (77·2 to 78·2)	57	5·3 (4·7 to 5·8)	7·3 (6·5 to 8·0)
Bulgaria	71·3 (71·1 to 71·4)	74·0 (73·9 to 74·1)	74·2 (74·1 to 74·2)	72·2 (72·2 to 72·3)	74·5 (74·4 to 74·7)	75·9 (75·7 to 76·1)	88	4·7 (4·4 to 4·9)	6·6 (6·2 to 6·9)
Croatia	72·5 (72·4 to 72·6)	76·6 (76·5 to 76·8)	77·7 (77·6 to 77·8)	77·0 (76·8 to 77·1)	77·5 (77·3 to 77·7)	78·6 (78·4 to 78·8)	50	6·1 (5·8 to 6·3)	8·4 (8·0 to 8·7)
Czechia	71·5 (71·4 to 71·6)	77·6 (77·5 to 77·7)	78·3 (78·3 to 78·4)	77·4 (77·3 to 77·4)	79·1 (79·0 to 79·2)	79·9 (79·8 to 80·0)	41	8·4 (8·2 to 8·5)	11·7 (11·4 to 11·9)
Estonia	70·0 (69·8 to 70·2)	75·5 (75·3 to 75·7)	78·5 (78·4 to 78·6)	77·4 (77·3 to 77·5)	77·8 (77·6 to 78·1)	78·7 (78·5 to 79·0)	48	8·7 (8·4 to 9·0)	12·4 (12·0 to 12·9)
Georgia	69·2 (68·9 to 69·5)	71·7 (71·4 to 72·1)	73·2 (73·0 to 73·3)	71·3 (71·1 to 71·5)	73·5 (73·1 to 73·9)	74·7 (74·3 to 75·2)	102	5·5 (5·0 to 6·1)	8·0 (7·2 to 8·8)
Hungary	69·4 (69·3 to 69·5)	74·6 (74·5 to 74·7)	75·7 (75·7 to 75·7)	74·3 (74·2 to 74·3)	76·0 (75·8 to 76·1)	76·8 (76·7 to 77·0)	68	7·4 (7·2 to 7·6)	10·7 (10·4 to 11·0)
Kazakhstan	67·7 (67·4 to 67·9)	68·7 (68·4 to 68·9)	71·7 (71·6 to 71·8)	70·7 (70·6 to 70·8)	74·1 (73·9 to 74·4)	74·9 (74·6 to 75·2)	100	7·2 (6·8 to 7·6)	10·6 (10·0 to 11·3)
Kyrgyzstan	66·8 (66·4 to 67·2)	69·4 (69·0 to 69·7)	71·6 (71·4 to 71·7)	72·3 (72·1 to 72·5)	74·7 (74·3 to 75·2)	75·2 (74·8 to 75·7)	95	8·4 (7·8 to 9·1)	12·6 (11·7 to 13·7)
Latvia	69·9 (69·8 to 70·1)	73·3 (73·1 to 73·5)	75·8 (75·7 to 75·9)	73·7 (73·6 to 73·8)	74·8 (74·6 to 75·1)	75·8 (75·5 to 76·1)	89	5·9 (5·5 to 6·2)	8·4 (7·9 to 8·8)
Lithuania	70·9 (70·8 to 71·1)	72·9 (72·7 to 73·0)	75·2 (75·1 to 75·3)	74·3 (74·2 to 74·4)	75·5 (75·3 to 75·7)	76·6 (76·4 to 76·9)	72	5·7 (5·4 to 6·0)	8·1 (7·6 to 8·5)
Moldova	68·2 (67·8 to 68·5)	70·5 (70·2 to 70·8)	73·1 (72·9 to 73·3)	72·2 (72·0 to 72·4)	75·2 (74·8 to 75·7)	76·0 (75·5 to 76·6)	85	7·8 (7·2 to 8·5)	11·5 (10·5 to 12·4)
Mongolia	61·4 (61·0 to 61·9)	66·9 (66·5 to 67·4)	72·6 (72·3 to 72·8)	70·6 (70·3 to 70·8)	72·1 (71·6 to 72·7)	73·1 (72·4 to 73·7)	113	11·6 (10·9 to 12·4)	19·0 (17·6 to 20·3)
Montenegro	73·1 (72·9 to 73·3)	75·1 (74·9 to 75·3)	75·2 (75·1 to 75·3)	73·2 (73·1 to 73·4)	76·2 (76·0 to 76·5)	77·1 (76·8 to 77·3)	64	4·0 (3·6 to 4·3)	5·4 (5·0 to 5·9)
North Macedonia	71·1 (71·0 to 71·3)	74·9 (74·7 to 75·0)	74·6 (74·5 to 74·6)	73·3 (73·2 to 73·4)	75·9 (75·7 to 76·1)	77·0 (76·6 to 77·4)	65	5·9 (5·5 to 6·3)	8·3 (7·7 to 8·9)
Poland	71·2 (71·1 to 71·3)	76·4 (76·3 to 76·5)	76·7 (76·6 to 76·7)	75·7 (75·7 to 75·7)	77·5 (77·4 to 77·6)	78·6 (78·6 to 78·7)	49	7·5 (7·3 to 7·6)	10·5 (10·3 to 10·7)
Romania	70·1 (70·0 to 70·2)	73·9 (73·8 to 74·0)	74·3 (74·3 to 74·4)	73·0 (73·0 to 73·0)	75·3 (75·1 to 75·4)	76·3 (76·1 to 76·4)	78	6·2 (6·0 to 6·3)	8·8 (8·6 to 9·1)
Russia	69·4 (69·3 to 69·4)	69·2 (69·2 to 69·3)	72·0 (71·9 to 72·0)	70·6 (70·6 to 70·7)	72·8 (72·5 to 73·1)	73·2 (72·6 to 73·6)	109	3·8 (3·3 to 4·2)	5·5 (4·7 to 6·1)
Serbia	70·1 (69·8 to 70·3)	74·1 (73·9 to 74·3)	74·7 (74·6 to 74·8)	73·3 (73·2 to 73·4)	75·3 (75·0 to 75·5)	76·0 (75·4 to 76·6)	87	5·9 (5·2 to 6·6)	8·5 (7·5 to 9·4)
Slovakia	71·0 (70·9 to 71·1)	75·5 (75·4 to 75·6)	76·9 (76·8 to 77·0)	74·6 (74·6 to 74·7)	76·9 (76·8 to 77·1)	78·2 (78·0 to 78·3)	53	7·2 (7·0 to 7·4)	10·1 (9·8 to 10·4)
Slovenia	73·3 (73·1 to 73·4)	79·3 (79·1 to 79·4)	80·4 (80·3 to 80·5)	80·6 (80·5 to 80·6)	81·1 (80·9 to 81·2)	81·6 (81·5 to 81·8)	24	8·4 (8·1 to 8·6)	11·4 (11·1 to 11·8)
Tajikistan	66·5 (66·1 to 66·9)	71·7 (71·3 to 72·0)	71·9 (71·7 to 72·1)	70·8 (70·3 to 71·4)	71·3 (70·6 to 72·1)	71·8 (71·0 to 72·5)	130	5·3 (4·4 to 6·1)	7·9 (6·6 to 9·3)
Turkmenistan	66·0 (65·6 to 66·5)	69·4 (69·0 to 69·9)	69·1 (68·4 to 69·7)	68·7 (67·9 to 69·4)	70·5 (69·7 to 71·3)	71·1 (70·2 to 72·0)	136	5·1 (4·1 to 6·0)	7·7 (6·2 to 9·2)
Ukraine	70·6 (70·5 to 70·7)	70·4 (70·3 to 70·5)	72·3 (72·3 to 72·4)	70·8 (70·7 to 70·8)	68·4 (65·8 to 70·3)	72·5 (71·9 to 73·2)	123	2·0 (1·3 to 2·6)	2·8 (1·9 to 3·7)
Uzbekistan	68·3 (68·0 to 68·5)	70·1 (69·9 to 70·3)	71·2 (71·1 to 71·3)	71·6 (71·5 to 71·7)	72·1 (71·8 to 72·4)	72·5 (72·2 to 72·8)	124	4·2 (3·8 to 4·6)	6·1 (5·6 to 6·7)
**High-income**	**76·1 (76·1 to 76·1)**	**80·4 (80·4 to 80·4)**	**80·5 (80·5 to 80·5)**	**80·2 (80·2 to 80·2)**	**80·5 (80·5 to 80·5)**	**81·1 (81·1 to 81·2)**		**5·1 (5·0 to 5·1)**	**6·6 (6·6 to 6·7)**
Andorra	79·4 (79·1 to 79·7)	82·7 (82·5 to 83·0)	82·7 (82·4 to 83·0)	83·4 (83·1 to 83·7)	83·2 (82·9 to 83·6)	83·9 (83·4 to 84·3)	6	4·4 (3·9 to 5·0)	5·6 (4·9 to 6·3)
Argentina	72·3 (72·3 to 72·4)	75·7 (75·7 to 75·8)	76·4 (76·4 to 76·5)	74·6 (74·6 to 74·6)	76·3 (76·2 to 76·4)	77·6 (77·3 to 77·8)	58	5·2 (4·9 to 5·5)	7·2 (6·8 to 7·6)
Australia	76·8 (76·7 to 76·9)	82·0 (81·9 to 82·0)	83·8 (83·8 to 83·8)	83·7 (83·7 to 83·7)	83·1 (83·0 to 83·1)	83·9 (83·8 to 83·9)	5	7·1 (7·0 to 7·2)	9·2 (9·1 to 9·3)
Austria	75·8 (75·7 to 75·9)	80·4 (80·3 to 80·5)	81·3 (81·2 to 81·3)	81·2 (81·1 to 81·2)	81·5 (81·4 to 81·6)	81·9 (81·8 to 82·0)	21	6·1 (6·0 to 6·2)	8·1 (7·9 to 8·2)
Belgium	76·0 (75·9 to 76·1)	80·0 (79·9 to 80·1)	80·4 (80·4 to 80·5)	81·5 (81·4 to 81·5)	81·6 (81·5 to 81·7)	82·1 (82·0 to 82·2)	18	6·1 (6·0 to 6·3)	8·1 (7·9 to 8·2)
Brunei	71·8 (71·6 to 72·1)	76·5 (76·3 to 76·8)	78·0 (77·8 to 78·3)	77·7 (77·5 to 78·0)	77·4 (77·1 to 77·7)	78·1 (77·6 to 78·5)	55	6·2 (5·7 to 6·8)	8·7 (7·9 to 9·4)
Canada	77·4 (77·3 to 77·4)	81·5 (81·5 to 81·6)	81·6 (81·6 to 81·6)	81·5 (81·5 to 81·6)	81·2 (81·2 to 81·3)	81·9 (81·7 to 82·1)	22	4·5 (4·3 to 4·7)	5·8 (5·6 to 6·1)
Chile	73·5 (73·4 to 73·6)	78·8 (78·7 to 78·9)	79·4 (79·3 to 79·4)	78·7 (78·7 to 78·8)	79·6 (79·5 to 79·7)	80·8 (80·7 to 80·9)	33	7·3 (7·1 to 7·5)	9·9 (9·7 to 10·2)
Cyprus	75·0 (74·5 to 75·6)	79·3 (78·8 to 79·8)	80·1 (79·7 to 80·5)	79·7 (79·3 to 80·1)	80·0 (79·4 to 80·6)	80·5 (79·9 to 81·1)	35	5·5 (4·6 to 6·3)	7·3 (6·1 to 8·5)
Denmark	75·0 (74·9 to 75·1)	79·3 (79·2 to 79·4)	81·5 (81·4 to 81·5)	81·4 (81·4 to 81·4)	81·2 (81·1 to 81·3)	81·5 (81·4 to 81·7)	26	6·5 (6·4 to 6·7)	8·7 (8·5 to 8·9)
Finland	75·2 (75·1 to 75·3)	80·1 (80·0 to 80·2)	81·9 (81·8 to 81·9)	81·9 (81·8 to 81·9)	81·2 (81·0 to 81·3)	81·7 (81·6 to 81·8)	23	6·5 (6·3 to 6·7)	8·6 (8·4 to 8·9)
France	77·3 (77·3 to 77·3)	81·5 (81·4 to 81·5)	82·3 (82·3 to 82·3)	82·8 (82·8 to 82·8)	82·4 (82·3 to 82·4)	82·6 (82·6 to 82·7)	15	5·3 (5·3 to 5·4)	6·9 (6·8 to 7·0)
Germany	75·6 (75·5 to 75·6)	80·3 (80·3 to 80·4)	81·0 (81·0 to 81·0)	80·6 (80·6 to 80·6)	80·5 (80·5 to 80·6)	80·9 (80·7 to 81·1)	32	5·4 (5·2 to 5·6)	7·1 (6·8 to 7·4)
Greece	77·1 (77·0 to 77·1)	80·3 (80·2 to 80·4)	81·2 (81·2 to 81·2)	80·2 (80·1 to 80·2)	80·3 (80·2 to 80·4)	81·0 (80·8 to 81·1)	31	3·9 (3·8 to 4·1)	5·1 (4·9 to 5·3)
Greenland	63·0 (62·7 to 63·3)	68·3 (68·1 to 68·6)	69·8 (69·5 to 70·1)	69·5 (69·2 to 69·8)	70·7 (70·3 to 71·1)	71·6 (71·1 to 72·0)	133	8·6 (8·0 to 9·1)	13·6 (12·7 to 14·5)
Iceland	77·9 (77·7 to 78·1)	81·7 (81·5 to 81·9)	82·7 (82·6 to 82·9)	82·8 (82·6 to 83·0)	82·2 (81·9 to 82·5)	82·5 (82·2 to 82·8)	17	4·6 (4·2 to 4·9)	5·9 (5·4 to 6·3)
Ireland	74·9 (74·8 to 75·1)	80·6 (80·4 to 80·7)	82·2 (82·1 to 82·2)	82·3 (82·3 to 82·4)	82·2 (82·0 to 82·3)	82·5 (82·4 to 82·7)	16	7·6 (7·4 to 7·8)	10·1 (9·9 to 10·4)
Israel	77·3 (77·2 to 77·5)	82·0 (81·9 to 82·1)	83·0 (82·9 to 83·0)	82·8 (82·8 to 82·9)	83·1 (83·0 to 83·2)	83·3 (83·1 to 83·4)	9	5·9 (5·8 to 6·1)	7·7 (7·4 to 7·9)
Italy	77·0 (77·0 to 77·1)	81·9 (81·8 to 81·9)	82·0 (82·0 to 82·0)	82·4 (82·3 to 82·4)	82·2 (82·1 to 82·2)	83·0 (82·9 to 83·0)	14	5·9 (5·9 to 6·0)	7·7 (7·6 to 7·8)
Japan	79·2 (79·1 to 79·2)	83·1 (83·1 to 83·1)	84·8 (84·8 to 84·8)	84·6 (84·6 to 84·6)	84·2 (84·2 to 84·2)	84·1 (84·1 to 84·1)	4	4·9 (4·9 to 5·0)	6·3 (6·2 to 6·3)
Luxembourg	75·2 (75·0 to 75·4)	80·9 (80·7 to 81·1)	82·5 (82·3 to 82·6)	82·8 (82·6 to 82·9)	83·0 (82·7 to 83·2)	83·2 (82·9 to 83·5)	10	8·0 (7·6 to 8·3)	10·6 (10·1 to 11·1)
Malta	76·3 (76·0 to 76·5)	80·9 (80·7 to 81·1)	82·2 (82·0 to 82·3)	82·3 (82·2 to 82·5)	82·3 (82·0 to 82·6)	83·0 (82·7 to 83·3)	12	6·8 (6·4 to 7·1)	8·9 (8·4 to 9·3)

Monaco	76·2 (75·6 to 76·7)	78·3 (77·8 to 78·8)	79·6 (79·1 to 80·1)	79·3 (78·7 to 79·9)	79·8 (79·1 to 80·6)	80·4 (79·6 to 81·2)	37	4·2 (3·3 to 5·2)	5·6 (4·3 to 6·8)
Netherlands	76·9 (76·8 to 77·0)	80·7 (80·6 to 80·8)	81·2 (81·2 to 81·2)	81·2 (81·2 to 81·2)	81·5 (81·4 to 81·6)	82·0 (81·9 to 82·1)	19	5·1 (5·0 to 5·2)	6·6 (6·5 to 6·7)
New Zealand	75·5 (75·4 to 75·7)	80·9 (80·8 to 81·1)	82·3 (82·2 to 82·3)	82·1 (82·0 to 82·1)	81·4 (81·2 to 81·5)	81·6 (81·5 to 81·8)	25	6·1 (5·9 to 6·3)	8·1 (7·8 to 8·3)
Norway	76·6 (76·5 to 76·7)	81·0 (80·9 to 81·1)	83·1 (83·0 to 83·1)	83·1 (83·0 to 83·1)	82·5 (82·4 to 82·6)	83·0 (82·9 to 83·1)	13	6·4 (6·2 to 6·5)	8·3 (8·1 to 8·5)
Portugal	74·2 (74·1 to 74·3)	80·1 (80·0 to 80·2)	81·4 (81·4 to 81·4)	81·4 (81·3 to 81·4)	81·3 (81·2 to 81·4)	82·0 (81·9 to 82·1)	20	7·8 (7·6 to 7·9)	10·5 (10·3 to 10·7)
San Marino	78·4 (77·9 to 79·0)	84·2 (83·7 to 84·7)	84·0 (83·5 to 84·4)	84·7 (84·0 to 85·2)	84·0 (83·4 to 84·6)	85·1 (84·4 to 85·8)	2	6·7 (5·8 to 7·6)	8·5 (7·3 to 9·7)
Singapore	75·6 (75·4 to 75·8)	82·8 (82·7 to 83·0)	85·7 (85·7 to 85·8)	85·3 (85·2 to 85·4)	84·6 (84·4 to 84·8)	85·4 (85·2 to 85·5)	1	9·8 (9·5 to 10·0)	12·9 (12·6 to 13·3)
South Korea	71·7 (71·5 to 72·0)	80·7 (80·5 to 80·9)	83·5 (83·4 to 83·6)	83·5 (83·4 to 83·5)	82·6 (82·4 to 82·7)	83·4 (83·3 to 83·6)	7	11·7 (11·4 to 11·9)	16·3 (15·8 to 16·7)
Spain	76·8 (76·8 to 76·9)	82·0 (81·9 to 82·0)	82·1 (82·1 to 82·2)	82·9 (82·9 to 82·9)	82·4 (82·4 to 82·5)	83·2 (83·1 to 83·2)	11	6·3 (6·3 to 6·4)	8·2 (8·1 to 8·3)
Sweden	77·7 (77·6 to 77·8)	81·6 (81·5 to 81·6)	82·4 (82·4 to 82·4)	83·0 (82·9 to 83·0)	83·1 (83·0 to 83·2)	83·3 (83·2 to 83·4)	8	5·6 (5·5 to 5·7)	7·2 (7·0 to 7·3)
Switzerland	77·7 (77·6 to 77·8)	82·4 (82·3 to 82·5)	83·1 (83·1 to 83·2)	83·6 (83·6 to 83·7)	83·7 (83·6 to 83·8)	84·4 (84·3 to 84·5)	3	6·7 (6·6 to 6·9)	8·7 (8·5 to 8·9)
UK	75·7 (75·7 to 75·8)	80·4 (80·4 to 80·4)	80·4 (80·3 to 80·4)	80·6 (80·6 to 80·6)	81·0 (81·0 to 81·0)	81·0 (80·9 to 81·0)	30	5·2 (5·2 to 5·3)	6·9 (6·8 to 7·0)
USA	75·5 (75·5 to 75·5)	78·7 (78·7 to 78·7)	77·1 (77·1 to 77·1)	76·4 (76·4 to 76·5)	77·6 (77·5 to 77·6)	78·5 (78·5 to 78·5)	51	3·0 (3·0 to 3·0)	4·0 (4·0 to 4·0)
Uruguay	73·1 (72·9 to 73·3)	76·4 (76·3 to 76·6)	77·9 (77·8 to 77·9)	75·1 (75·0 to 75·2)	76·2 (76·0 to 76·3)	77·4 (77·2 to 77·6)	60	4·3 (4·1 to 4·6)	5·9 (5·6 to 6·3)
**Latin America and Caribbean**	**70·1 (70·0 to 70·2)**	**73·7 (73·6 to 73·8)**	**73·4 (73·4 to 73·4)**	**72·1 (72·0 to 72·1)**	**75·3 (75·3 to 75·4)**	**76·0 (76·0 to 76·1)**		**5·9 (5·8 to 6·0)**	**8·5 (8·3 to 8·6)**
Antigua and Barbuda	73·3 (72·9 to 73·7)	77·5 (77·1 to 77·9)	77·3 (76·9 to 77·7)	76·3 (75·9 to 76·8)	77·3 (76·6 to 77·8)	77·8 (77·1 to 78·4)	56	4·5 (3·6 to 5·2)	6·1 (5·0 to 7·2)
Bahamas	71·0 (70·7 to 71·2)	72·4 (72·2 to 72·6)	71·2 (70·9 to 71·5)	67·4 (67·1 to 67·7)	71·5 (71·2 to 71·9)	72·8 (72·4 to 73·3)	119	1·9 (1·4 to 2·4)	2·7 (1·9 to 3·4)
Barbados	74·0 (73·7 to 74·3)	77·1 (76·8 to 77·3)	76·5 (76·2 to 76·8)	75·5 (75·1 to 75·9)	75·6 (75·1 to 76·0)	76·7 (76·2 to 77·1)	71	2·7 (2·2 to 3·2)	3·6 (2·9 to 4·3)
Belize	73·2 (72·9 to 73·5)	73·8 (73·5 to 74·1)	73·2 (72·9 to 73·5)	71·6 (71·3 to 71·9)	73·8 (73·4 to 74·4)	75·2 (74·7 to 75·8)	96	2·0 (1·4 to 2·7)	2·8 (1·9 to 3·7)
Bermuda	74·2 (74·0 to 74·4)	80·6 (80·4 to 80·8)	80·7 (80·5 to 80·9)	79·8 (79·5 to 80·0)	80·1 (79·8 to 80·4)	80·7 (80·4 to 81·1)	34	6·5 (6·1 to 6·9)	8·7 (8·2 to 9·4)
Bolivia	63·0 (62·8 to 63·3)	69·4 (69·1 to 69·6)	63·8 (63·5 to 64·1)	64·6 (64·3 to 65·0)	70·6 (70·2 to 70·9)	71·9 (71·4 to 72·3)	128	8·8 (8·3 to 9·4)	14·0 (13·1 to 14·9)
Brazil	69·7 (69·5 to 69·8)	74·9 (74·8 to 75·0)	75·2 (75·2 to 75·2)	73·3 (73·2 to 73·3)	76·1 (76·0 to 76·2)	76·9 (76·8 to 77·0)	67	7·2 (7·0 to 7·4)	10·3 (10·1 to 10·6)
Colombia	71·9 (71·7 to 72·0)	78·0 (77·9 to 78·2)	77·5 (77·4 to 77·5)	75·3 (75·2 to 75·3)	78·7 (78·6 to 78·8)	80·1 (80·0 to 80·3)	38	8·2 (8·0 to 8·4)	11·5 (11·1 to 11·8)
Costa Rica	76·7 (76·5 to 76·9)	79·8 (79·6 to 79·9)	79·4 (79·3 to 79·5)	77·9 (77·8 to 78·0)	79·0 (78·8 to 79·2)	79·4 (79·2 to 79·7)	44	2·7 (2·4 to 3·0)	3·6 (3·2 to 3·9)
Cuba	75·3 (75·1 to 75·6)	78·5 (78·2 to 78·7)	77·8 (77·7 to 78·0)	73·6 (73·5 to 73·7)	77·5 (76·8 to 78·2)	78·1 (77·3 to 78·9)	54	2·8 (1·9 to 3·6)	3·7 (2·6 to 4·8)
Dominica	74·5 (74·2 to 74·7)	75·5 (75·3 to 75·8)	75·3 (75·0 to 75·6)	74·3 (73·9 to 74·7)	75·7 (75·3 to 76·1)	76·1 (75·7 to 76·6)	79	1·7 (1·2 to 2·1)	2·2 (1·6 to 2·9)
Dominican Republic	70·9 (70·6 to 71·1)	75·0 (74·7 to 75·3)	72·0 (71·8 to 72·1)	72·2 (72·0 to 72·4)	72·4 (72·1 to 72·6)	73·0 (72·6 to 73·5)	117	2·1 (1·7 to 2·6)	3·0 (2·3 to 3·7)
Ecuador	70·9 (70·8 to 71·1)	75·8 (75·6 to 76·0)	73·7 (73·6 to 73·8)	74·8 (74·7 to 74·9)	76·7 (76·5 to 76·9)	76·7 (76·5 to 76·9)	69	5·8 (5·5 to 6·1)	8·2 (7·7 to 8·6)
El Salvador	68·5 (68·3 to 68·8)	73·5 (73·3 to 73·8)	71·3 (71·0 to 71·7)	70·5 (70·0 to 71·0)	72·7 (72·1 to 73·3)	73·1 (72·5 to 73·7)	112	4·6 (3·9 to 5·3)	6·7 (5·7 to 7·7)
Grenada	70·6 (70·1 to 71·1)	75·1 (74·7 to 75·6)	74·6 (74·1 to 75·1)	72·2 (71·6 to 72·8)	74·3 (73·5 to 75·0)	75·2 (74·3 to 76·0)	97	4·6 (3·5 to 5·6)	6·4 (5·0 to 7·9)
Guatemala	62·2 (61·8 to 62·6)	71·3 (71·0 to 71·6)	71·2 (71·1 to 71·3)	68·8 (68·7 to 69·0)	71·8 (71·5 to 72·2)	73·2 (72·4 to 74·0)	108	11·0 (10·1 to 11·9)	17·7 (16·2 to 19·2)
Guyana	64·8 (64·3 to 65·4)	67·8 (67·3 to 68·3)	69·6 (69·1 to 70·1)	66·8 (66·4 to 67·2)	68·3 (67·6 to 69·1)	69·0 (68·0 to 69·9)	151	4·1 (3·0 to 5·2)	6·4 (4·6 to 8·0)
Haiti	51·4 (50·7 to 52·0)	31·0 (29·2 to 32·7)	61·6 (61·0 to 62·1)	60·9 (60·2 to 61·5)	61·4 (60·6 to 62·2)	61·0 (60·2 to 61·9)	196	9·7 (8·6 to 10·7)	18·9 (16·6 to 21·0)
Honduras	70·5 (70·3 to 70·7)	74·1 (73·9 to 74·3)	72·6 (72·3 to 72·8)	70·8 (70·5 to 71·1)	75·5 (75·2 to 75·8)	76·1 (75·7 to 76·5)	82	5·6 (5·1 to 6·0)	7·9 (7·3 to 8·5)
Jamaica	74·9 (74·5 to 75·3)	77·3 (76·9 to 77·7)	74·5 (74·0 to 75·1)	72·9 (72·2 to 73·7)	74·9 (74·1 to 75·7)	76·1 (75·2 to 77·1)	80	1·2 (0·3 to 2·3)	1·6 (0·3 to 3·0)
Mexico	71·1 (71·0 to 71·3)	75·4 (75·4 to 75·5)	71·4 (71·3 to 71·4)	71·1 (71·1 to 71·1)	75·3 (75·2 to 75·4)	76·3 (76·2 to 76·4)	76	5·2 (5·0 to 5·3)	7·3 (7·1 to 7·5)
Nicaragua	71·0 (70·5 to 71·4)	75·0 (74·6 to 75·4)	71·9 (71·8 to 72·1)	71·5 (71·3 to 71·7)	75·3 (74·9 to 75·8)	76·7 (75·9 to 77·5)	70	5·8 (4·7 to 6·6)	8·1 (6·6 to 9·4)
Panama	76·1 (75·9 to 76·3)	78·1 (77·9 to 78·3)	76·7 (76·6 to 76·8)	77·1 (77·0 to 77·2)	78·6 (78·4 to 78·9)	79·2 (78·8 to 79·5)	45	3·1 (2·7 to 3·5)	4·1 (3·5 to 4·6)
Paraguay	75·0 (74·7 to 75·2)	73·8 (73·6 to 74·0)	72·4 (72·3 to 72·5)	67·3 (67·1 to 67·4)	72·4 (72·0 to 72·8)	73·0 (72·5 to 73·5)	116	-1·9 (-2·5 to -1·4)	-2·6 (-3·3 to -1·9)
Peru	72·9 (72·6 to 73·1)	79·0 (78·8 to 79·2)	72·4 (72·3 to 72·5)	71·0 (71·0 to 71·1)	78·0 (77·8 to 78·2)	77·1 (76·8 to 77·4)	63	4·2 (3·9 to 4·6)	5·8 (5·3 to 6·4)
Puerto Rico	74·7 (74·5 to 74·8)	79·1 (79·0 to 79·3)	80·6 (80·5 to 80·7)	80·3 (80·1 to 80·4)	80·1 (79·9 to 80·3)	81·0 (80·7 to 81·2)	29	6·3 (6·0 to 6·6)	8·5 (8·1 to 8·9)
Saint Kitts and Nevis	68·8 (68·6 to 69·1)	73·6 (73·4 to 73·9)	74·1 (73·9 to 74·4)	73·1 (72·7 to 73·4)	74·3 (73·9 to 74·6)	74·8 (74·4 to 75·2)	101	6·0 (5·6 to 6·5)	8·7 (8·1 to 9·4)
Saint Lucia	70·6 (70·1 to 71·1)	75·6 (75·2 to 76·1)	76·7 (76·2 to 77·2)	74·4 (73·6 to 75·0)	76·6 (75·8 to 77·3)	77·4 (76·5 to 78·3)	61	6·8 (5·8 to 7·8)	9·6 (8·1 to 11·1)
Saint Vincent and the Grenadines	71·7 (71·2 to 72·1)	73·8 (73·3 to 74·3)	74·2 (73·7 to 74·7)	74·1 (73·5 to 74·8)	74·9 (74·1 to 75·7)	75·1 (74·3 to 76·0)	98	3·5 (2·5 to 4·5)	4·8 (3·4 to 6·3)
Suriname	70·7 (70·5 to 70·9)	72·4 (72·2 to 72·6)	72·2 (72·0 to 72·4)	67·9 (67·7 to 68·1)	72·1 (71·8 to 72·3)	73·1 (72·8 to 73·5)	111	2·4 (2·0 to 2·8)	3·4 (2·8 to 3·9)
Trinidad and Tobago	69·0 (68·8 to 69·2)	73·1 (72·9 to 73·3)	72·8 (72·6 to 73·1)	69·5 (69·1 to 69·8)	71·5 (71·1 to 71·9)	73·2 (72·8 to 73·5)	110	4·1 (3·7 to 4·5)	6·0 (5·3 to 6·6)
Venezuela	71·7 (71·5 to 71·8)	74·6 (74·4 to 74·7)	71·8 (71·5 to 72·1)	70·9 (70·5 to 71·2)	71·6 (71·2 to 72·1)	71·8 (71·3 to 72·2)	129	0·1 (-0·4 to 0·6)	0·2 (-0·5 to 0·8)
Virgin Islands	71·8 (71·4 to 72·3)	76·0 (75·5 to 76·5)	77·2 (76·7 to 77·8)	75·6 (74·9 to 76·3)	76·4 (75·5 to 77·2)	76·4 (75·6 to 77·3)	74	4·6 (3·7 to 5·6)	6·4 (5·1 to 7·8)
**North Africa and Middle East**	**65·4 (65·2 to 65·5)**	**72·3 (72·2 to 72·3)**	**71·8 (71·7 to 71·9)**	**71·5 (71·4 to 71·6)**	**73·5 (73·4 to 73·7)**	**73·7 (73·4 to 74·0)**		**8·3 (8·0 to 8·6)**	**12·7 (12·3 to 13·2)**
Afghanistan	59·0 (58·4 to 59·7)	65·6 (65·1 to 66·1)	64·6 (64·0 to 65·2)	62·0 (61·3 to 62·7)	68·0 (67·1 to 68·8)	68·5 (67·6 to 69·3)	154	9·5 (8·4 to 10·5)	16·1 (14·1 to 18·0)
Algeria	68·4 (68·2 to 68·7)	74·5 (74·3 to 74·7)	74·7 (74·6 to 74·9)	75·0 (74·8 to 75·1)	75·7 (75·5 to 75·9)	76·0 (75·7 to 76·3)	86	7·5 (7·1 to 8·0)	11·0 (10·4 to 11·6)
Bahrain	69·9 (69·4 to 70·3)	74·9 (74·4 to 75·3)	76·1 (75·7 to 76·4)	75·5 (75·0 to 76·1)	77·0 (76·3 to 77·7)	77·2 (76·5 to 78·0)	62	7·4 (6·5 to 8·2)	10·6 (9·3 to 11·8)
Egypt	63·8 (63·6 to 64·0)	68·7 (68·6 to 68·8)	69·1 (69·0 to 69·2)	68·4 (68·3 to 68·5)	69·6 (69·0 to 70·3)	70·0 (69·2 to 70·8)	147	6·2 (5·4 to 7·1)	9·8 (8·4 to 11·1)
Iran	66·4 (66·1 to 66·7)	76·4 (76·2 to 76·6)	74·2 (74·0 to 74·4)	74·8 (74·6 to 75·1)	78·1 (77·8 to 78·3)	78·8 (78·5 to 79·2)	47	12·4 (12·0 to 12·9)	18·7 (17·9 to 19·6)
Iraq	66·6 (66·4 to 66·8)	68·4 (68·1 to 68·7)	67·5 (67·2 to 67·8)	68·7 (68·4 to 69·0)	70·8 (70·4 to 71·1)	71·1 (70·6 to 71·5)	137	4·4 (3·9 to 4·9)	6·7 (5·8 to 7·4)
Jordan	70·7 (70·5 to 71·0)	74·9 (74·7 to 75·1)	75·3 (75·1 to 75·4)	73·8 (73·6 to 73·9)	77·5 (77·3 to 77·7)	78·5 (78·1 to 78·8)	52	7·7 (7·3 to 8·2)	10·9 (10·3 to 11·6)

Kuwait	71·8 (71·5 to 72·0)	78·0 (77·8 to 78·2)	78·6 (78·4 to 78·9)	78·6 (78·4 to 78·8)	80·5 (80·2 to 80·8)	81·1 (80·7 to 81·5)	28	9·3 (8·8 to 9·8)	13·0 (12·3 to 13·7)
Lebanon	74·7 (74·1 to 75·3)	80·4 (80·1 to 80·7)	72·9 (72·7 to 73·1)	77·0 (76·7 to 77·2)	78·1 (77·4 to 78·9)	78·9 (78·1 to 79·7)	46	4·2 (3·2 to 5·3)	5·6 (4·3 to 7·1)
Libya	70·5 (70·2 to 70·7)	74·0 (73·9 to 74·2)	72·2 (71·9 to 72·5)	70·3 (69·9 to 70·6)	74·5 (74·1 to 74·8)	70·1 (69·5 to 70·7)	143	-0·3 (-1·0 to 0·3)	-0·5 (-1·4 to 0·5)
Morocco	65·0 (64·7 to 65·4)	70·9 (70·6 to 71·3)	71·6 (71·3 to 71·9)	71·7 (71·4 to 72·1)	72·7 (72·2 to 73·3)	73·0 (72·3 to 73·7)	115	8·0 (7·2 to 8·7)	12·3 (11·0 to 13·5)
Oman	68·7 (68·3 to 69·2)	73·9 (73·6 to 74·3)	73·5 (73·2 to 73·8)	72·6 (72·3 to 72·9)	76·3 (75·8 to 76·7)	77·0 (76·4 to 77·5)	66	8·2 (7·5 to 9·0)	12·0 (10·9 to 13·1)
Palestine	70·8 (70·6 to 71·1)	74·4 (74·2 to 74·6)	76·0 (75·8 to 76·1)	74·6 (74·4 to 74·7)	76·6 (76·4 to 76·8)	65·9 (64·9 to 66·9)	168	-4·9 (-6·0 to -4·0)	-6·9 (-8·4 to -5·6)
Qatar	71·7 (71·3 to 72·3)	77·4 (77·0 to 77·9)	80·5 (80·1 to 80·8)	80·7 (80·3 to 81·2)	81·3 (80·7 to 82·0)	81·4 (80·6 to 82·1)	27	9·6 (8·7 to 10·5)	13·4 (12·1 to 14·6)
Saudi Arabia	68·4 (68·0 to 68·8)	71·6 (71·3 to 71·9)	72·4 (72·1 to 72·6)	73·5 (73·4 to 73·6)	74·2 (73·8 to 74·5)	74·5 (73·9 to 75·1)	103	6·1 (5·4 to 6·8)	9·0 (7·8 to 10·0)
Sudan	55·9 (55·2 to 56·5)	65·6 (65·1 to 66·1)	67·6 (67·1 to 68·2)	68·2 (67·5 to 68·9)	69·3 (68·5 to 70·0)	68·9 (68·0 to 69·7)	152	13·0 (11·9 to 14·1)	23·3 (21·1 to 25·4)
Syria	69·1 (68·9 to 69·3)	74·5 (74·4 to 74·7)	74·4 (74·1 to 74·6)	74·2 (74·0 to 74·5)	76·1 (75·8 to 76·4)	75·4 (75·0 to 75·8)	93	6·3 (5·9 to 6·8)	9·2 (8·5 to 9·8)
Tunisia	69·0 (68·8 to 69·2)	73·8 (73·6 to 74·0)	72·7 (72·7 to 72·8)	70·4 (70·3 to 70·5)	74·9 (74·7 to 75·1)	75·6 (75·3 to 75·9)	91	6·6 (6·3 to 7·0)	9·6 (9·1 to 10·2)
Türkiye	68·1 (67·8 to 68·3)	77·3 (77·1 to 77·5)	76·2 (76·2 to 76·3)	75·4 (75·3 to 75·4)	76·9 (76·7 to 77·0)	76·1 (75·3 to 76·8)	83	8·0 (7·2 to 8·8)	11·8 (10·6 to 13·0)
United Arab Emirates	70·4 (70·0 to 70·7)	76·5 (76·2 to 76·9)	79·5 (79·2 to 79·8)	79·5 (79·1 to 79·8)	80·1 (79·7 to 80·5)	80·1 (79·6 to 80·6)	39	9·7 (9·0 to 10·4)	13·8 (12·8 to 14·8)
Yemen	59·7 (59·1 to 60·4)	69·2 (68·7 to 69·7)	67·9 (67·3 to 68·5)	68·6 (67·9 to 69·2)	71·0 (70·2 to 71·7)	71·7 (70·8 to 72·5)	132	12·0 (11·0 to 13·0)	20·0 (18·2 to 21·9)
**South Asia**	**58·6 (58·2 to 59·0)**	**67·4 (67·2 to 67·7)**	**69·8 (69·6 to 69·9)**	**68·3 (68·2 to 68·5)**	**70·9 (70·3 to 71·5)**	**71·2 (70·5 to 71·9)**		**12·6 (11·8 to 13·4)**	**21·5 (20·1 to 23·0)**
Bangladesh	57·0 (56·4 to 57·6)	68·3 (67·8 to 68·7)	69·6 (69·0 to 70·1)	68·4 (67·7 to 69·0)	70·9 (70·2 to 71·7)	71·1 (70·2 to 71·8)	138	14·0 (13·1 to 15·0)	24·6 (22·8 to 26·5)
Bhutan	59·4 (58·7 to 60·0)	69·4 (68·9 to 69·9)	72·3 (71·7 to 72·8)	72·5 (71·8 to 73·1)	72·4 (71·7 to 73·2)	72·6 (71·8 to 73·5)	121	13·2 (12·2 to 14·3)	22·3 (20·5 to 24·3)
India	58·5 (57·9 to 59·0)	67·6 (67·2 to 67·9)	70·3 (70·1 to 70·5)	68·8 (68·6 to 69·0)	71·2 (70·5 to 72·0)	71·6 (70·8 to 72·4)	134	13·1 (12·1 to 14·1)	22·4 (20·7 to 24·2)
Nepal	58·1 (57·5 to 58·8)	70·6 (70·2 to 71·0)	72·2 (71·7 to 72·8)	70·1 (69·5 to 70·8)	73·4 (72·6 to 74·1)	73·5 (72·7 to 74·4)	107	15·4 (14·3 to 16·5)	26·5 (24·5 to 28·6)
Pakistan	62·1 (61·5 to 62·7)	66·5 (66·0 to 67·1)	66·8 (66·2 to 67·4)	66·4 (65·6 to 67·1)	69·6 (68·7 to 70·4)	70·0 (69·1 to 70·9)	146	7·9 (6·9 to 9·0)	12·7 (11·0 to 14·5)
**Southeast Asia, East Asia, and Oceania**	**66·5 (66·2 to 66·9)**	**74·3 (74·1 to 74·6)**	**77·9 (77·8 to 78·0)**	**76·8 (76·6 to 77·0)**	**77·2 (76·7 to 77·7)**	**77·4 (76·9 to 78·0)**		**10·9 (10·3 to 11·6)**	**16·4 (15·4 to 17·5)**
American Samoa	69·4 (68·9 to 69·8)	70·0 (69·5 to 70·5)	70·2 (69·6 to 70·7)	67·8 (67·1 to 68·6)	70·3 (69·6 to 71·1)	70·9 (70·0 to 71·8)	139	1·5 (0·6 to 2·5)	2·2 (0·8 to 3·6)
Cambodia	58·3 (57·7 to 58·9)	70·1 (69·5 to 70·6)	73·4 (72·8 to 73·9)	73·0 (72·3 to 73·7)	73·3 (72·6 to 74·1)	73·1 (72·2 to 73·9)	114	14·8 (13·8 to 15·8)	25·3 (23·5 to 27·4)
China	66·8 (66·4 to 67·3)	76·0 (75·7 to 76·3)	80·7 (80·6 to 80·7)	80·2 (80·0 to 80·5)	79·9 (79·2 to 80·5)	80·1 (79·3 to 80·8)	40	13·3 (12·4 to 14·2)	19·8 (18·4 to 21·3)
Cook Islands	68·1 (67·9 to 68·4)	72·2 (71·9 to 72·4)	72·7 (72·4 to 73·0)	71·3 (71·0 to 71·7)	72·7 (72·3 to 73·1)	72·9 (72·5 to 73·4)	118	4·8 (4·3 to 5·4)	7·1 (6·3 to 7·9)
Federated States of Micronesia	62·1 (61·9 to 62·4)	65·5 (65·3 to 65·8)	66·3 (66·0 to 66·6)	64·8 (64·5 to 65·1)	66·5 (66·1 to 66·8)	67·0 (66·5 to 67·4)	161	4·8 (4·3 to 5·4)	7·8 (6·9 to 8·7)
Fiji	67·4 (67·1 to 67·6)	67·3 (67·1 to 67·5)	68·0 (67·9 to 68·2)	66·3 (66·1 to 66·5)	67·7 (67·4 to 68·0)	67·7 (67·3 to 68·1)	158	0·3 (-0·2 to 0·8)	0·5 (-0·2 to 1·1)
Guam	74·0 (73·8 to 74·3)	76·6 (76·3 to 76·8)	75·5 (75·2 to 75·7)	74·3 (74·0 to 74·6)	75·0 (74·6 to 75·3)	75·5 (75·0 to 75·9)	92	1·5 (1·0 to 2·0)	2·0 (1·3 to 2·7)
Indonesia	65·0 (64·4 to 65·6)	70·2 (69·6 to 70·8)	71·4 (70·7 to 72·0)	68·6 (67·8 to 69·3)	72·4 (71·5 to 73·3)	72·5 (71·5 to 73·4)	125	7·5 (6·4 to 8·5)	11·5 (9·7 to 13·1)
Kiribati	65·0 (64·7 to 65·3)	68·9 (68·7 to 69·2)	70·5 (70·2 to 70·9)	69·1 (68·7 to 69·4)	70·8 (70·4 to 71·2)	71·2 (70·7 to 71·7)	135	6·2 (5·6 to 6·8)	9·6 (8·7 to 10·5)
Laos	50·8 (50·0 to 51·5)	63·9 (63·4 to 64·5)	66·2 (65·6 to 66·8)	66·2 (65·5 to 66·9)	66·0 (65·1 to 66·8)	66·1 (65·2 to 67·0)	167	15·4 (14·2 to 16·6)	30·3 (27·7 to 33·2)
Malaysia	71·4 (71·1 to 71·7)	74·9 (74·4 to 75·4)	76·6 (76·4 to 76·7)	73·3 (73·2 to 73·4)	75·0 (74·7 to 75·3)	76·4 (76·1 to 76·7)	75	5·0 (4·6 to 5·4)	7·0 (6·4 to 7·6)
Maldives	65·0 (64·7 to 65·2)	74·7 (74·5 to 74·9)	76·0 (75·8 to 76·2)	75·1 (74·9 to 75·3)	75·6 (75·4 to 75·9)	76·1 (75·7 to 76·4)	84	11·1 (10·7 to 11·5)	17·1 (16·3 to 17·7)
Marshall Islands	61·6 (61·4 to 61·8)	63·1 (62·8 to 63·3)	63·7 (63·4 to 64·0)	62·3 (62·0 to 62·6)	63·8 (63·5 to 64·2)	64·1 (63·6 to 64·6)	180	2·5 (2·0 to 3·0)	4·1 (3·2 to 4·9)
Mauritius	69·8 (69·6 to 70·1)	74·0 (73·8 to 74·3)	74·6 (74·4 to 74·7)	72·8 (72·7 to 73·0)	73·4 (73·1 to 73·7)	73·8 (73·5 to 74·2)	105	4·0 (3·6 to 4·4)	5·7 (5·1 to 6·4)
Myanmar	54·8 (54·1 to 55·3)	63·4 (62·9 to 63·9)	65·8 (65·2 to 66·3)	64·0 (63·2 to 64·7)	65·5 (64·5 to 66·4)	65·4 (64·4 to 66·4)	171	10·7 (9·4 to 11·8)	19·5 (17·2 to 21·7)
Nauru	58·1 (57·8 to 58·3)	57·3 (57·1 to 57·6)	58·4 (58·1 to 58·7)	56·2 (55·9 to 56·6)	58·7 (58·3 to 59·1)	59·0 (58·5 to 59·6)	199	1·0 (0·4 to 1·6)	1·7 (0·6 to 2·7)
Niue	69·5 (69·3 to 69·7)	71·7 (71·4 to 71·9)	72·8 (72·5 to 73·1)	71·1 (70·7 to 71·4)	71·9 (71·6 to 72·3)	72·3 (71·8 to 72·8)	126	2·8 (2·3 to 3·3)	4·0 (3·2 to 4·8)
North Korea	71·5 (71·0 to 72·0)	72·7 (72·2 to 73·3)	73·6 (73·1 to 74·1)	73·8 (73·1 to 74·5)	73·7 (72·9 to 74·5)	73·7 (73·0 to 74·6)	106	2·3 (1·3 to 3·3)	3·2 (1·8 to 4·6)
Northern Mariana Islands	68·4 (68·1 to 68·6)	69·9 (69·7 to 70·1)	70·0 (69·7 to 70·3)	68·5 (68·2 to 68·8)	70·2 (69·9 to 70·5)	70·8 (70·4 to 71·2)	140	2·4 (1·9 to 3·0)	3·6 (2·8 to 4·3)
Palau	66·3 (66·1 to 66·5)	68·2 (67·9 to 68·4)	68·3 (68·0 to 68·6)	66·7 (66·4 to 67·0)	68·5 (68·1 to 68·8)	68·9 (68·4 to 69·3)	153	2·6 (2·1 to 3·1)	3·9 (3·1 to 4·6)
Papua New Guinea	62·0 (61·5 to 62·5)	64·4 (63·9 to 64·9)	66·5 (65·9 to 67·1)	65·9 (65·2 to 66·7)	66·2 (65·4 to 67·0)	66·3 (65·5 to 67·2)	164	4·4 (3·3 to 5·4)	7·0 (5·4 to 8·7)
Philippines	68·8 (68·6 to 69·0)	70·8 (70·7 to 70·9)	71·7 (71·7 to 71·8)	67·8 (67·8 to 67·8)	70·8 (70·6 to 70·9)	72·1 (71·9 to 72·2)	127	3·3 (3·0 to 3·5)	4·7 (4·3 to 5·2)
Samoa	71·3 (71·1 to 71·6)	72·7 (72·4 to 72·9)	72·5 (72·2 to 72·8)	71·1 (70·7 to 71·4)	72·5 (72·1 to 72·9)	72·6 (72·1 to 73·1)	122	1·2 (0·7 to 1·8)	1·7 (0·9 to 2·5)
Seychelles	70·5 (70·3 to 70·8)	73·2 (73·0 to 73·4)	75·8 (75·6 to 76·1)	72·2 (71·9 to 72·4)	75·0 (74·6 to 75·4)	75·1 (74·7 to 75·4)	99	4·5 (4·1 to 5·0)	6·4 (5·8 to 7·1)
Solomon Islands	61·4 (61·2 to 61·7)	64·4 (64·1 to 64·6)	63·6 (63·2 to 63·9)	63·1 (62·8 to 63·5)	63·5 (63·2 to 63·9)	63·7 (63·3 to 64·2)	181	2·3 (1·8 to 2·8)	3·8 (2·9 to 4·6)
Sri Lanka	69·4 (69·2 to 69·6)	74·2 (74·0 to 74·4)	78·2 (78·0 to 78·3)	76·4 (76·3 to 76·5)	76·5 (76·3 to 76·7)	76·6 (76·4 to 76·8)	73	7·2 (6·9 to 7·5)	10·3 (9·9 to 10·8)
Taiwan*	74·2 (74·1 to 74·3)	79·3 (79·3 to 79·4)	81·1 (81·1 to 81·2)	80·9 (80·9 to 81·0)	80·0 (79·9 to 80·1)	80·5 (80·4 to 80·6)	36	6·3 (6·2 to 6·5)	8·5 (8·3 to 8·8)
Thailand	71·9 (71·8 to 72·0)	75·1 (75·0 to 75·2)	77·8 (77·7 to 77·9)	76·8 (76·7 to 76·9)	76·5 (76·4 to 76·6)	77·5 (77·3 to 77·7)	59	5·6 (5·4 to 5·8)	7·8 (7·5 to 8·1)
Timor-Leste	57·4 (56·8 to 58·1)	68·5 (68·0 to 69·0)	69·3 (68·7 to 69·9)	69·1 (68·4 to 69·8)	69·7 (68·9 to 70·5)	69·9 (69·0 to 70·8)	149	12·4 (11·4 to 13·5)	21·6 (19·7 to 23·7)
Tokelau	71·9 (71·7 to 72·1)	74·3 (74·1 to 74·5)	75·9 (75·7 to 76·2)	74·4 (74·1 to 74·7)	75·0 (74·7 to 75·3)	75·4 (74·9 to 75·8)	94	3·5 (3·0 to 4·0)	4·8 (4·2 to 5·5)
Tonga	70·6 (70·3 to 70·8)	71·7 (71·4 to 71·9)	71·6 (71·2 to 71·9)	69·6 (69·3 to 69·9)	71·4 (71·0 to 71·7)	71·7 (71·3 to 72·2)	131	1·1 (0·6 to 1·7)	1·6 (0·9 to 2·4)
Tuvalu	59·7 (59·4 to 60·0)	62·9 (62·7 to 63·2)	63·1 (62·8 to 63·4)	61·6 (61·2 to 61·9)	63·1 (62·7 to 63·5)	63·3 (62·8 to 63·7)	184	3·6 (3·0 to 4·1)	6·0 (5·1 to 6·8)
Vanuatu	66·5 (66·2 to 66·7)	69·4 (69·2 to 69·7)	69·8 (69·5 to 70·1)	69·1 (68·8 to 69·5)	70·0 (69·6 to 70·4)	70·3 (69·7 to 70·8)	142	3·8 (3·1 to 4·4)	5·7 (4·7 to 6·6)
Viet Nam	69·2 (68·8 to 69·7)	74·2 (73·6 to 74·7)	75·9 (75·3 to 76·4)	75·1 (74·4 to 75·8)	75·7 (74·8 to 76·6)	76·1 (75·2 to 77·1)	81	6·8 (5·8 to 7·9)	9·9 (8·4 to 11·4)
**Sub-Saharan Africa**	**52·1 (52·0 to 52·3)**	**59·2 (59·0 to 59·3)**	**62·6 (62·4 to 62·7)**	**61·4 (61·3 to 61·6)**	**63·7 (63·5 to 63·9)**	**64·2 (64·0 to 64·4)**		**12·0 (11·7 to 12·3)**	**23·1 (22·4 to 23·7)**

Angola	49·7 (49·0 to 50·3)	60·0 (59·5 to 60·6)	62·8 (62·3 to 63·4)	61·8 (61·1 to 62·5)	64·0 (63·3 to 64·8)	64·3 (63·3 to 65·2)	178	14·6 (13·4 to 15·7)	29·5 (26·9 to 31·9)
Benin	54·2 (53·6 to 54·8)	62·5 (61·9 to 63·0)	65·1 (64·6 to 65·7)	65·4 (64·7 to 66·1)	65·9 (65·1 to 66·7)	66·2 (65·3 to 67·0)	166	12·0 (11·0 to 13·0)	22·1 (20·1 to 24·1)
Botswana	64·4 (64·1 to 64·6)	65·8 (65·5 to 66·0)	69·0 (68·8 to 69·3)	65·8 (65·6 to 66·1)	69·3 (69·0 to 69·6)	70·1 (69·6 to 70·5)	144	5·7 (5·2 to 6·2)	8·9 (8·1 to 9·7)
Burkina Faso	50·0 (49·3 to 50·7)	63·1 (62·6 to 63·7)	67·2 (66·7 to 67·7)	67·2 (66·6 to 67·8)	67·8 (67·1 to 68·5)	67·7 (67·0 to 68·5)	157	17·7 (16·7 to 18·8)	35·5 (33·0 to 37·9)
Burundi	48·0 (47·3 to 48·7)	59·0 (58·4 to 59·5)	61·2 (60·6 to 61·8)	59·5 (58·9 to 60·2)	61·6 (60·8 to 62·4)	61·8 (61·0 to 62·6)	190	13·8 (12·7 to 14·9)	28·8 (26·1 to 31·3)
Cabo Verde	69·6 (69·2 to 70·1)	75·0 (74·5 to 75·4)	73·3 (72·8 to 73·8)	72·3 (71·7 to 72·9)	74·8 (74·1 to 75·4)	75·7 (74·9 to 76·4)	90	6·0 (5·2 to 6·9)	8·7 (7·4 to 9·9)
Cameroon	55·0 (54·4 to 55·6)	55·7 (55·1 to 56·3)	60·2 (59·6 to 60·7)	59·0 (58·4 to 59·6)	61·5 (60·7 to 62·3)	62·0 (61·2 to 62·8)	189	7·0 (6·0 to 8·0)	12·6 (10·8 to 14·6)
Central African Republic	48·5 (47·8 to 49·1)	50·8 (50·2 to 51·4)	53·8 (53·2 to 54·4)	53·9 (53·3 to 54·6)	54·6 (53·8 to 55·3)	55·0 (54·2 to 55·9)	204	6·6 (5·5 to 7·7)	13·6 (11·1 to 16·0)
Chad	48·1 (47·5 to 48·8)	54·4 (53·8 to 54·9)	56·5 (56·0 to 57·1)	57·8 (57·2 to 58·4)	57·7 (57·0 to 58·4)	58·0 (57·3 to 58·8)	201	9·9 (8·9 to 10·9)	20·5 (18·2 to 22·8)
Comoros	66·5 (66·0 to 67·1)	74·8 (74·3 to 75·3)	74·3 (73·8 to 74·8)	71·6 (71·0 to 72·2)	75·4 (74·7 to 76·1)	76·3 (75·5 to 77·1)	77	9·7 (8·8 to 10·7)	14·6 (13·2 to 16·2)
Congo (Brazzaville)	55·2 (54·6 to 55·8)	62·0 (61·5 to 62·5)	62·7 (62·2 to 63·3)	61·9 (61·2 to 62·6)	63·5 (62·7 to 64·2)	63·7 (62·8 to 64·6)	182	8·5 (7·5 to 9·6)	15·4 (13·4 to 17·5)
Côte d'Ivoire	54·5 (53·9 to 55·1)	59·6 (59·1 to 60·2)	63·1 (62·5 to 63·6)	63·2 (62·6 to 63·8)	63·9 (63·2 to 64·6)	64·3 (63·4 to 65·1)	179	9·7 (8·7 to 10·8)	17·9 (15·8 to 20·0)
DR Congo	52·2 (51·5 to 52·8)	57·1 (56·5 to 57·6)	59·6 (59·0 to 60·2)	59·7 (59·1 to 60·4)	60·7 (59·9 to 61·4)	61·3 (60·5 to 62·2)	193	9·2 (8·2 to 10·2)	17·5 (15·5 to 19·8)
Djibouti	61·5 (61·0 to 62·1)	64·9 (64·4 to 65·5)	67·0 (66·5 to 67·6)	65·3 (64·6 to 65·9)	67·8 (67·0 to 68·6)	68·4 (67·4 to 69·3)	155	6·8 (5·8 to 7·9)	11·1 (9·4 to 12·9)
Equatorial Guinea	49·0 (48·4 to 49·7)	59·4 (58·9 to 60·0)	60·1 (59·5 to 60·7)	59·9 (59·2 to 60·6)	61·1 (60·3 to 61·9)	61·3 (60·4 to 62·3)	194	12·3 (11·2 to 13·5)	25·1 (22·6 to 27·7)
Eritrea	45·0 (43·9 to 46·0)	60·0 (59·4 to 60·6)	62·1 (61·5 to 62·6)	62·1 (61·4 to 62·8)	61·3 (60·5 to 62·1)	62·9 (61·9 to 63·8)	185	17·9 (16·5 to 19·4)	39·8 (36·2 to 44·0)
Eswatini	56·4 (55·8 to 57·0)	48·1 (47·4 to 48·8)	57·0 (56·4 to 57·7)	55·6 (54·8 to 56·3)	59·3 (58·4 to 60·2)	60·8 (59·7 to 61·8)	197	4·4 (3·2 to 5·6)	7·7 (5·6 to 10·0)
Ethiopia	44·5 (43·8 to 45·2)	60·5 (60·0 to 61·1)	63·7 (63·2 to 64·2)	62·1 (61·5 to 62·7)	64·4 (63·6 to 65·2)	64·7 (63·9 to 65·6)	175	20·2 (19·2 to 21·4)	45·5 (42·8 to 48·4)
Gabon	62·0 (61·5 to 62·5)	64·7 (64·2 to 65·3)	65·4 (64·8 to 65·9)	65·1 (64·4 to 65·8)	66·6 (65·8 to 67·4)	67·0 (66·2 to 67·9)	160	5·0 (4·0 to 6·1)	8·1 (6·4 to 9·8)
Ghana	56·2 (55·6 to 56·7)	62·6 (62·0 to 63·1)	64·3 (63·8 to 64·9)	63·7 (63·1 to 64·4)	65·0 (64·2 to 65·8)	65·4 (64·6 to 66·3)	170	9·3 (8·2 to 10·3)	16·5 (14·5 to 18·5)
Guinea	48·5 (47·8 to 49·2)	58·6 (58·0 to 59·1)	61·1 (60·6 to 61·7)	61·2 (60·6 to 61·9)	62·0 (61·2 to 62·7)	62·5 (61·7 to 63·3)	186	14·0 (12·9 to 15·1)	28·8 (26·2 to 31·4)
Guinea-Bissau	50·7 (49·9 to 51·4)	57·6 (57·0 to 58·1)	59·3 (58·7 to 59·9)	59·8 (59·2 to 60·4)	60·6 (59·9 to 61·3)	61·2 (60·4 to 62·0)	195	10·5 (9·4 to 11·6)	20·8 (18·3 to 23·1)
Kenya	62·0 (61·4 to 62·5)	64·4 (63·9 to 64·9)	66·6 (66·0 to 67·1)	65·2 (64·5 to 65·8)	69·4 (68·6 to 70·1)	69·9 (69·0 to 70·8)	148	8·0 (7·0 to 9·0)	12·9 (11·2 to 14·6)
Lesotho	58·9 (58·4 to 59·4)	53·3 (52·7 to 53·9)	56·3 (55·6 to 57·0)	54·9 (54·2 to 55·6)	57·2 (56·3 to 58·1)	57·9 (56·9 to 58·8)	202	-1·0 (-2·1 to 0·0)	-1·7 (-3·5 to 0·1)
Liberia	46·8 (46·1 to 47·5)	57·9 (57·3 to 58·5)	58·2 (57·6 to 58·8)	56·2 (55·5 to 56·8)	60·8 (60·1 to 61·6)	61·5 (60·7 to 62·3)	191	14·7 (13·5 to 15·8)	31·3 (28·7 to 34·0)
Madagascar	51·8 (51·2 to 52·4)	60·7 (60·2 to 61·2)	61·6 (61·0 to 62·1)	60·8 (60·2 to 61·3)	61·8 (61·1 to 62·6)	62·5 (61·7 to 63·3)	187	10·7 (9·6 to 11·7)	20·7 (18·4 to 22·8)
Malawi	45·6 (45·0 to 46·3)	57·3 (56·8 to 57·9)	61·2 (60·6 to 61·8)	55·6 (54·9 to 56·3)	61·2 (60·4 to 62·1)	62·0 (61·1 to 62·8)	188	16·4 (15·2 to 17·5)	35·8 (32·8 to 38·7)
Mali	48·2 (47·5 to 49·0)	60·8 (60·2 to 61·3)	61·3 (60·8 to 61·9)	61·0 (60·3 to 61·7)	62·8 (62·0 to 63·5)	63·6 (62·8 to 64·3)	183	15·3 (14·2 to 16·4)	31·8 (29·1 to 34·5)
Mauritania	60·6 (60·1 to 61·1)	66·0 (65·5 to 66·5)	66·1 (65·6 to 66·6)	65·4 (64·9 to 66·1)	66·9 (66·2 to 67·6)	68·1 (67·4 to 68·8)	156	7·5 (6·6 to 8·4)	12·3 (10·8 to 13·9)
Mozambique	52·3 (51·5 to 53·0)	60·0 (59·4 to 60·6)	62·8 (62·2 to 63·3)	60·5 (59·9 to 61·2)	64·0 (63·1 to 64·7)	64·9 (64·0 to 65·7)	172	12·6 (11·4 to 13·7)	24·0 (21·6 to 26·4)
Namibia	60·1 (59·9 to 60·4)	62·2 (61·9 to 62·4)	64·2 (63·9 to 64·5)	58·6 (58·3 to 59·0)	65·0 (64·6 to 65·3)	66·4 (65·9 to 66·9)	163	6·2 (5·7 to 6·8)	10·3 (9·4 to 11·3)
Niger	42·5 (41·5 to 43·4)	55·6 (54·9 to 56·2)	57·5 (57·0 to 58·1)	58·2 (57·6 to 58·9)	58·8 (58·0 to 59·5)	59·2 (58·4 to 60·0)	198	16·7 (15·6 to 17·9)	39·4 (36·0 to 42·8)
Nigeria	51·4 (50·6 to 52·1)	59·3 (58·8 to 59·9)	63·0 (62·5 to 63·5)	63·3 (62·7 to 63·9)	64·3 (63·6 to 65·0)	64·5 (63·7 to 65·3)	177	13·1 (12·0 to 14·2)	25·6 (23·2 to 28·0)
Rwanda	46·2 (45·5 to 46·9)	62·4 (61·9 to 62·9)	65·0 (64·5 to 65·5)	63·5 (62·9 to 64·1)	64·8 (64·1 to 65·6)	64·8 (64·0 to 65·6)	174	18·6 (17·5 to 19·6)	40·2 (37·6 to 42·8)
Senegal	57·5 (56·9 to 58·1)	67·1 (66·6 to 67·6)	68·4 (68·0 to 69·0)	67·1 (66·5 to 67·7)	69·7 (69·0 to 70·4)	70·1 (69·2 to 70·8)	145	12·6 (11·6 to 13·6)	22·0 (20·1 to 23·8)
Sierra Leone	47·0 (46·3 to 47·7)	53·9 (53·3 to 54·4)	57·3 (56·7 to 57·9)	58·1 (57·5 to 58·8)	58·6 (57·8 to 59·3)	58·9 (58·0 to 59·7)	200	11·9 (10·9 to 12·9)	25·3 (22·8 to 27·9)
Somalia	52·2 (51·5 to 52·9)	52·4 (51·6 to 53·2)	63·0 (62·4 to 63·5)	59·6 (59·0 to 60·3)	64·4 (63·6 to 65·1)	64·8 (64·1 to 65·6)	173	12·6 (11·6 to 13·7)	24·2 (22·0 to 26·5)
South Africa	67·7 (67·1 to 68·4)	58·2 (57·9 to 58·4)	64·4 (64·3 to 64·5)	61·6 (61·6 to 61·7)	65·7 (65·4 to 65·9)	66·5 (66·3 to 66·8)	162	-1·2 (-1·9 to -0·5)	-1·7 (-2·7 to -0·7)
South Sudan	50·1 (49·4 to 50·8)	56·0 (55·4 to 56·6)	55·8 (55·2 to 56·4)	56·0 (55·4 to 56·7)	56·3 (55·4 to 57·0)	56·4 (55·5 to 57·2)	203	6·3 (5·2 to 7·4)	12·5 (10·3 to 14·9)
São Tomé and Príncipe	63·6 (63·0 to 64·1)	66·5 (66·0 to 67·0)	67·0 (66·5 to 67·6)	67·0 (66·4 to 67·6)	69·1 (68·5 to 69·9)	70·3 (69·5 to 71·2)	141	6·7 (5·8 to 7·7)	10·6 (9·1 to 12·1)
Tanzania	55·0 (54·3 to 55·6)	62·4 (61·9 to 63·0)	67·2 (66·7 to 67·7)	65·2 (64·6 to 65·8)	68·9 (68·2 to 69·7)	69·4 (68·6 to 70·3)	150	14·4 (13·4 to 15·5)	26·2 (24·2 to 28·4)
The Gambia	60·8 (60·2 to 61·4)	65·8 (65·3 to 66·2)	64·4 (63·9 to 64·9)	63·7 (63·0 to 64·3)	66·4 (65·6 to 67·1)	67·3 (66·5 to 68·0)	159	6·4 (5·4 to 7·5)	10·6 (8·9 to 12·3)
Togo	55·8 (55·1 to 56·3)	59·5 (59·0 to 60·1)	63·4 (62·8 to 63·9)	63·3 (62·6 to 63·8)	64·1 (63·3 to 64·8)	64·5 (63·7 to 65·4)	176	8·8 (7·8 to 9·7)	15·7 (13·8 to 17·6)
Uganda	46·9 (46·3 to 47·6)	59·3 (58·8 to 59·9)	64·6 (64·0 to 65·1)	61·6 (61·0 to 62·2)	65·1 (64·3 to 65·8)	65·6 (64·8 to 66·5)	169	18·7 (17·5 to 19·8)	39·9 (37·0 to 42·6)
Zambia	48·8 (48·2 to 49·5)	55·4 (54·8 to 55·9)	59·2 (58·6 to 59·8)	56·3 (55·6 to 57·0)	60·9 (60·0 to 61·7)	61·5 (60·6 to 62·3)	192	12·6 (11·5 to 13·7)	25·8 (23·4 to 28·3)
Zimbabwe	64·8 (64·2 to 65·4)	56·7 (56·1 to 57·3)	63·5 (62·9 to 64·1)	59·5 (58·8 to 60·2)	64·9 (64·1 to 65·8)	66·3 (65·3 to 67·2)	165	1·5 (0·5 to 2·6)	2·3 (0·7 to 4·0)
**Low SDI**	**53·3 (53·1 to 53·5)**	**61·9 (61·8 to 62·1)**	**64·8 (64·7 to 64·9)**	**63·7 (63·5 to 63·8)**	**65·9 (65·7 to 66·2)**	**66·3 (66·0 to 66·6)**		**13·0 (12·6 to 13·3)**	**24·3 (23·6 to 25·0)**
**Low-middle SDI**	**59·4 (59·0 to 59·7)**	**66·8 (66·6 to 67·0)**	**68·7 (68·5 to 68·8)**	**67·5 (67·3 to 67·7)**	**69·7 (69·3 to 70·1)**	**70·0 (69·5 to 70·5)**		**10·6 (10·0 to 11·2)**	**17·9 (16·9 to 18·9)**
**Middle SDI**	**65·9 (65·7 to 66·1)**	**71·4 (71·2 to 71·6)**	**72·7 (72·5 to 72·8)**	**71·1 (70·9 to 71·3)**	**73·1 (72·8 to 73·4)**	**73·4 (73·1 to 73·7)**		**7·5 (7·1 to 7·8)**	**11·4 (10·8 to 11·9)**
**High-middle SDI**	**66·5 (66·3 to 66·9)**	**73·5 (73·3 to 73·7)**	**75·6 (75·5 to 75·7)**	**74·5 (74·3 to 74·6)**	**76·2 (75·8 to 76·6)**	**76·6 (76·2 to 77·0)**		**10·0 (9·6 to 10·6)**	**15·1 (14·3 to 15·9)**
**High SDI**	**72·3 (72·1 to 72·4)**	**77·4 (77·3 to 77·5)**	**79·1 (79·1 to 79·1)**	**78·5 (78·4 to 78·5)**	**79·1 (78·9 to 79·2)**	**79·6 (79·4 to 79·8)**		**7·3 (7·1 to 7·6)**	**10·1 (9·8 to 10·5)**

*United Nations convention recognises Taiwan as a province of China

Table S2: Healthy life expectancy, 1990, 2010, 2020, 2021, 2022, and 2023, and absolute and relative changes, 1990–2023, for all sexes combined, globally, by SDI quintile, and for GBD super-regions and countries and territories

Location	1990	2010	2020	2021	2022	2023	Rank (2023)	Absolute Change (1990-2023)	Relative Change, % (1990-2023)
**Global**	**55·9 (53·5 to 57·9)**	**61·1 (58·5 to 63·4)**	**62·6 (59·7 to 65·0)**	**61·7 (58·8 to 64·0)**	**62·8 (59·9 to 65·3)**	**63·1 (60·2 to 65·6)**		**7·3 (6·7 to 7·8)**	**13·0 (12·2 to 13·7)**
**Central Europe, Eastern Europe, and Central Asia**	**60·1 (57·5 to 62·3)**	**61·8 (59·2 to 64·1)**	**62·9 (60·1 to 65·3)**	**61·9 (59·1 to 64·2)**	**63·1 (60·2 to 65·5)**	**64·0 (61·0 to 66·4)**		**3·9 (3·4 to 4·4)**	**6·5 (5·8 to 7·2)**
Albania	62·3 (59·6 to 64·6)	67·7 (64·8 to 70·2)	66·9 (63·9 to 69·4)	66·2 (63·1 to 68·6)	67·6 (64·5 to 70·1)	68·6 (65·4 to 71·3)	36	6·3 (5·4 to 7·0)	10·0 (8·8 to 11·3)
Armenia	61·2 (58·7 to 63·4)	64·5 (61·8 to 66·8)	61·0 (58·3 to 63·2)	64·4 (61·5 to 66·9)	66·4 (63·6 to 69·1)	68·1 (64·9 to 70·9)	41	6·9 (6·0 to 7·8)	11·3 (10·0 to 12·5)
Azerbaijan	57·6 (55·1 to 59·7)	62·4 (59·9 to 64·6)	61·0 (58·6 to 63·2)	61·5 (59·0 to 63·8)	63·2 (60·4 to 65·6)	62·8 (60·1 to 65·3)	119	5·2 (4·5 to 5·9)	9·1 (7·8 to 10·3)
Belarus	61·7 (59·1 to 64·0)	61·4 (58·9 to 63·6)	64·3 (61·5 to 66·7)	63·8 (60·9 to 66·2)	63·8 (60·9 to 66·2)	64·4 (61·5 to 66·8)	96	2·7 (2·1 to 3·3)	4·3 (3·4 to 5·3)
Bosnia and Herzegovina	62·9 (60·2 to 65·1)	65·8 (62·9 to 68·3)	65·2 (62·2 to 67·6)	64·1 (61·0 to 66·4)	65·6 (62·4 to 68·1)	66·6 (63·4 to 69·2)	59	3·6 (2·9 to 4·4)	5·8 (4·7 to 6·9)
Bulgaria	62·1 (59·5 to 64·2)	64·4 (61·7 to 66·7)	64·3 (61·5 to 66·6)	62·6 (59·7 to 64·9)	64·4 (61·5 to 66·8)	65·6 (62·5 to 68·1)	74	3·5 (2·9 to 4·1)	5·7 (4·8 to 6·4)
Croatia	63·1 (60·5 to 65·3)	66·4 (63·4 to 68·8)	66·8 (63·9 to 69·3)	66·0 (63·0 to 68·6)	66·5 (63·3 to 69·1)	67·3 (64·2 to 69·9)	48	4·2 (3·5 to 4·8)	6·7 (5·7 to 7·5)
Czechia	61·9 (59·3 to 64·2)	66·6 (63·6 to 69·2)	67·0 (63·9 to 69·7)	66·1 (63·0 to 68·7)	67·4 (64·2 to 70·1)	68·1 (64·8 to 70·8)	42	6·2 (5·4 to 6·8)	10·0 (9·0 to 10·7)
Estonia	60·8 (58·2 to 63·0)	65·2 (62·3 to 67·6)	67·4 (64·5 to 70·0)	66·5 (63·6 to 69·0)	66·8 (63·8 to 69·3)	67·4 (64·2 to 70·1)	45	6·6 (5·9 to 7·2)	10·9 (10·0 to 11·7)
Georgia	60·5 (58·0 to 62·5)	62·4 (59·7 to 64·6)	63·2 (60·3 to 65·5)	61·5 (58·8 to 63·9)	63·2 (60·3 to 65·7)	64·3 (61·3 to 66·9)	98	3·8 (2·9 to 4·7)	6·3 (5·0 to 7·6)
Hungary	59·9 (57·2 to 62·2)	64·6 (61·7 to 67·0)	65·3 (62·5 to 67·7)	64·1 (61·2 to 66·5)	65·4 (62·4 to 67·8)	66·0 (63·0 to 68·6)	69	6·1 (5·5 to 6·6)	10·2 (9·2 to 11·0)
Kazakhstan	58·7 (56·2 to 60·8)	59·7 (57·3 to 61·9)	62·4 (59·7 to 64·6)	61·4 (58·8 to 63·6)	64·0 (61·2 to 66·5)	64·6 (61·7 to 67·0)	92	5·9 (5·2 to 6·5)	10·1 (9·0 to 11·3)
Kyrgyzstan	58·0 (55·6 to 60·1)	60·5 (58·1 to 62·7)	62·3 (59·8 to 64·5)	62·6 (59·9 to 64·9)	64·5 (61·7 to 66·9)	65·1 (62·2 to 67·5)	86	7·1 (6·4 to 7·8)	12·2 (11·0 to 13·4)
Latvia	60·5 (57·8 to 62·8)	63·6 (60·8 to 66·0)	65·4 (62·6 to 67·9)	63·8 (60·9 to 66·1)	64·6 (61·7 to 67·0)	65·2 (62·2 to 67·7)	84	4·8 (4·2 to 5·3)	7·9 (7·0 to 8·8)
Lithuania	61·3 (58·6 to 63·6)	62·9 (60·2 to 65·3)	64·7 (61·7 to 67·1)	63·9 (60·9 to 66·4)	64·8 (61·7 to 67·4)	65·6 (62·7 to 68·2)	76	4·4 (3·7 to 4·9)	7·1 (6·1 to 7·9)
Moldova	59·3 (56·7 to 61·4)	61·5 (58·8 to 63·8)	63·3 (60·6 to 65·6)	62·5 (59·8 to 64·8)	64·8 (61·9 to 67·3)	65·5 (62·7 to 68·0)	79	6·2 (5·4 to 6·8)	10·4 (9·3 to 11·4)
Mongolia	53·9 (51·6 to 55·7)	58·8 (56·5 to 60·7)	63·3 (60·7 to 65·5)	61·6 (58·9 to 63·8)	62·7 (59·9 to 65·1)	63·4 (60·6 to 66·0)	107	9·5 (8·4 to 10·5)	17·7 (16·0 to 19·2)
Montenegro	63·9 (61·3 to 66·1)	65·4 (62·7 to 67·7)	65·2 (62·3 to 67·5)	63·4 (60·7 to 65·7)	65·6 (62·6 to 68·0)	66·3 (63·3 to 68·9)	62	2·5 (1·8 to 3·0)	3·8 (2·8 to 4·7)
North Macedonia	62·1 (59·7 to 64·2)	65·3 (62·5 to 67·6)	64·6 (61·8 to 66·9)	63·3 (60·6 to 65·6)	65·4 (62·5 to 67·9)	66·4 (63·5 to 68·8)	61	4·3 (3·7 to 4·9)	6·9 (6·0 to 7·7)
Poland	61·5 (58·8 to 63·8)	66·0 (63·1 to 68·5)	66·0 (63·0 to 68·5)	65·2 (62·1 to 67·6)	66·4 (63·3 to 69·0)	67·3 (64·1 to 70·0)	46	5·8 (5·2 to 6·4)	9·4 (8·6 to 10·2)
Romania	60·9 (58·3 to 63·1)	64·5 (61·8 to 66·7)	64·7 (61·9 to 67·0)	63·5 (60·8 to 65·7)	65·3 (62·4 to 67·6)	66·1 (63·1 to 68·4)	68	5·2 (4·6 to 5·6)	8·5 (7·7 to 9·4)
Russia	59·9 (57·3 to 62·2)	60·0 (57·4 to 62·2)	62·1 (59·2 to 64·4)	60·9 (58·0 to 63·1)	62·4 (59·5 to 64·9)	62·8 (59·9 to 65·4)	117	2·9 (2·4 to 3·5)	4·9 (3·9 to 5·8)
Serbia	61·6 (59·1 to 63·6)	64·9 (62·3 to 67·0)	65·2 (62·5 to 67·4)	63·9 (61·2 to 66·1)	65·4 (62·6 to 67·7)	66·0 (63·3 to 68·6)	71	4·4 (3·7 to 5·2)	7·2 (6·1 to 8·3)
Slovakia	61·7 (59·1 to 63·9)	65·4 (62·4 to 67·8)	66·4 (63·4 to 68·9)	64·6 (61·7 to 67·0)	66·3 (63·2 to 68·8)	67·2 (64·2 to 69·8)	50	5·5 (4·8 to 6·1)	8·9 (7·9 to 9·7)
Slovenia	63·4 (60·7 to 65·8)	67·9 (64·9 to 70·6)	68·8 (65·6 to 71·5)	68·7 (65·5 to 71·5)	69·0 (65·7 to 71·8)	69·3 (66·0 to 72·2)	25	5·9 (5·1 to 6·6)	9·3 (8·4 to 10·1)
Tajikistan	58·0 (55·5 to 60·1)	62·6 (60·0 to 64·8)	62·6 (60·0 to 64·8)	61·6 (59·0 to 63·9)	62·1 (59·3 to 64·2)	62·5 (59·8 to 65·0)	120	4·5 (3·7 to 5·4)	7·8 (6·3 to 9·3)
Turkmenistan	57·7 (55·4 to 59·6)	60·7 (58·4 to 63·0)	60·3 (57·7 to 62·4)	59·8 (57·3 to 62·1)	61·3 (58·7 to 63·8)	61·9 (59·2 to 64·3)	130	4·3 (3·4 to 5·1)	7·4 (5·8 to 8·8)
Ukraine	60·9 (58·3 to 63·2)	61·1 (58·6 to 63·4)	62·5 (59·7 to 64·8)	61·1 (58·4 to 63·4)	58·9 (55·6 to 62·0)	62·2 (59·3 to 64·7)	126	1·4 (0·8 to 1·9)	2·2 (1·3 to 3·2)
Uzbekistan	59·6 (57·0 to 61·8)	61·6 (59·2 to 63·7)	62·2 (59·5 to 64·3)	62·2 (59·5 to 64·5)	62·8 (59·9 to 65·0)	63·3 (60·6 to 65·5)	108	3·7 (3·0 to 4·3)	6·2 (5·1 to 7·2)
**High-income**	**65·5 (62·7 to 68·0)**	**68·6 (65·5 to 71·3)**	**68·2 (65·0 to 71·0)**	**67·9 (64·6 to 70·7)**	**68·1 (64·8 to 70·9)**	**68·4 (65·1 to 71·4)**		**2·9 (2·3 to 3·5)**	**4·5 (3·7 to 5·1)**
Andorra	68·9 (66·0 to 71·4)	71·2 (68·0 to 73·9)	70·8 (67·7 to 73·6)	71·1 (67·7 to 74·0)	71·1 (67·8 to 74·0)	71·6 (68·5 to 74·5)	6	2·6 (2·0 to 3·3)	3·8 (2·9 to 4·7)
Argentina	62·9 (60·3 to 65·2)	65·7 (63·0 to 68·1)	66·0 (63·1 to 68·5)	64·4 (61·6 to 66·9)	65·7 (62·8 to 68·2)	66·6 (63·7 to 69·2)	58	3·7 (3·0 to 4·3)	5·9 (4·9 to 6·6)
Australia	65·9 (62·9 to 68·5)	69·1 (65·6 to 72·1)	70·1 (66·5 to 73·3)	70·0 (66·4 to 73·2)	69·5 (65·9 to 72·7)	70·0 (66·4 to 73·2)	17	4·1 (3·3 to 4·9)	6·3 (5·3 to 7·2)
Austria	65·9 (63·2 to 68·3)	69·2 (66·2 to 71·8)	69·6 (66·3 to 72·3)	69·4 (66·1 to 72·1)	69·6 (66·3 to 72·4)	70·0 (66·7 to 72·7)	18	4·0 (3·4 to 4·6)	6·1 (5·3 to 6·8)
Belgium	66·1 (63·4 to 68·4)	68·6 (65·6 to 71·2)	68·7 (65·5 to 71·4)	69·3 (66·0 to 72·1)	69·3 (66·0 to 72·2)	69·8 (66·4 to 72·6)	21	3·7 (3·1 to 4·3)	5·6 (4·6 to 6·4)
Brunei	63·1 (60·7 to 65·2)	67·0 (64·3 to 69·2)	68·0 (65·4 to 70·4)	67·8 (65·1 to 70·1)	67·5 (64·8 to 69·8)	67·9 (65·3 to 70·4)	43	4·8 (4·2 to 5·4)	7·7 (6·8 to 8·6)
Canada	66·0 (62·9 to 68·7)	68·6 (65·2 to 71·6)	68·3 (64·9 to 71·3)	68·1 (64·6 to 71·1)	67·9 (64·4 to 70·9)	68·2 (64·8 to 71·3)	40	2·2 (1·5 to 2·9)	3·3 (2·4 to 4·3)
Chile	63·6 (60·9 to 66·0)	67·9 (65·0 to 70·5)	68·0 (64·9 to 70·7)	67·4 (64·4 to 70·2)	68·0 (64·8 to 70·8)	68·7 (65·5 to 71·6)	33	5·1 (4·4 to 5·8)	8·1 (7·0 to 8·8)
Cyprus	65·8 (63·0 to 68·0)	69·1 (66·2 to 71·3)	69·3 (66·2 to 71·9)	68·9 (65·8 to 71·5)	69·0 (66·0 to 71·7)	69·3 (66·2 to 72·2)	26	3·5 (2·5 to 4·5)	5·3 (3·9 to 6·7)
Denmark	65·3 (62·5 to 67·6)	68·6 (65·7 to 71·1)	70·0 (66·9 to 72·6)	69·9 (66·7 to 72·6)	69·6 (66·4 to 72·3)	69·9 (66·7 to 72·6)	20	4·6 (4·0 to 5·2)	7·0 (6·2 to 7·7)
Finland	65·4 (62·7 to 67·7)	68·7 (65·7 to 71·3)	70·0 (66·9 to 72·8)	70·0 (66·8 to 72·7)	69·4 (66·3 to 72·2)	69·7 (66·5 to 72·5)	22	4·4 (3·7 to 5·0)	6·7 (5·8 to 7·5)
France	67·0 (64·3 to 69·5)	69·9 (66·9 to 72·7)	70·1 (66·8 to 73·0)	70·3 (66·8 to 73·3)	70·0 (66·5 to 72·9)	70·1 (66·7 to 73·1)	16	3·1 (2·4 to 3·8)	4·6 (3·7 to 5·4)
Germany	65·5 (62·7 to 68·0)	68·8 (65·5 to 71·5)	69·2 (65·9 to 72·0)	68·9 (65·7 to 71·6)	68·8 (65·7 to 71·6)	69·0 (65·8 to 71·9)	31	3·5 (2·9 to 4·1)	5·4 (4·6 to 6·1)
Greece	67·0 (64·5 to 69·5)	69·1 (66·2 to 71·7)	69·5 (66·3 to 72·2)	68·7 (65·5 to 71·4)	68·7 (65·5 to 71·4)	69·1 (65·8 to 71·9)	29	2·1 (1·4 to 2·6)	3·1 (2·2 to 3·9)
Greenland	54·6 (52·3 to 56·6)	58·8 (56·1 to 61·1)	59·6 (56·8 to 62·2)	59·3 (56·6 to 61·7)	60·2 (57·3 to 62·8)	60·8 (57·8 to 63·3)	141	6·2 (5·4 to 7·0)	11·4 (10·2 to 12·7)
Iceland	67·7 (65·0 to 70·0)	70·2 (67·3 to 73·0)	70·5 (67·4 to 73·4)	70·5 (67·4 to 73·4)	70·1 (66·9 to 73·0)	70·2 (67·1 to 73·1)	15	2·5 (2·0 to 3·1)	3·7 (3·0 to 4·5)
Ireland	65·5 (62·9 to 67·8)	69·5 (66·5 to 72·2)	70·3 (66·9 to 73·1)	70·3 (66·9 to 73·1)	70·1 (66·6 to 72·9)	70·3 (66·8 to 73·2)	14	4·8 (3·9 to 5·5)	7·3 (6·1 to 8·2)
Israel	67·6 (64·8 to 69·9)	70·9 (67·7 to 73·6)	71·5 (68·2 to 74·2)	71·3 (68·0 to 74·1)	71·5 (68·3 to 74·4)	71·5 (68·1 to 74·4)	7	4·0 (3·2 to 4·6)	5·8 (5·0 to 6·7)
Italy	67·0 (64·2 to 69·4)	71·0 (68·0 to 73·6)	70·8 (67·7 to 73·5)	71·0 (67·8 to 73·6)	70·8 (67·6 to 73·4)	71·2 (68·0 to 74·0)	9	4·2 (3·6 to 4·7)	6·2 (5·5 to 6·8)
Japan	68·9 (66·1 to 71·4)	71·8 (68·6 to 74·4)	73·0 (69·8 to 75·7)	72·8 (69·6 to 75·5)	72·5 (69·2 to 75·2)	72·3 (69·1 to 75·1)	2	3·4 (2·9 to 3·8)	4·9 (4·3 to 5·4)
Luxembourg	65·5 (62·9 to 67·9)	69·9 (66·8 to 72·5)	70·7 (67·6 to 73·5)	70·8 (67·6 to 73·7)	71·0 (67·7 to 73·9)	71·1 (67·8 to 74·0)	10	5·6 (4·8 to 6·3)	8·5 (7·6 to 9·3)
Malta	66·9 (64·2 to 69·2)	70·2 (67·2 to 72·8)	70·8 (67·9 to 73·5)	70·9 (67·7 to 73·6)	70·8 (67·6 to 73·5)	71·2 (68·1 to 74·1)	8	4·4 (3·6 to 5·1)	6·5 (5·5 to 7·4)

Monaco	66·7 (64·2 to 68·9)	68·3 (65·3 to 70·7)	68·8 (65·8 to 71·4)	68·3 (65·2 to 71·0)	68·7 (65·6 to 71·3)	69·1 (66·0 to 71·7)	27	2·4 (1·4 to 3·3)	3·6 (2·1 to 4·9)
Netherlands	66·8 (64·0 to 69·2)	67·5 (64·0 to 70·6)	68·9 (65·6 to 71·8)	68·7 (65·3 to 71·6)	68·9 (65·3 to 71·8)	69·1 (65·6 to 72·1)	30	2·3 (1·5 to 3·0)	3·5 (2·3 to 4·4)
New Zealand	64·2 (61·0 to 66·9)	68·4 (65·0 to 71·3)	69·2 (65·7 to 72·2)	69·0 (65·6 to 72·1)	68·5 (65·1 to 71·5)	68·5 (65·1 to 71·6)	37	4·3 (3·8 to 4·9)	6·8 (6·1 to 7·4)
Norway	66·4 (63·5 to 68·8)	69·6 (66·5 to 72·3)	70·9 (67·6 to 73·8)	70·8 (67·5 to 73·7)	70·4 (67·0 to 73·2)	70·6 (67·2 to 73·5)	13	4·2 (3·6 to 4·8)	6·3 (5·5 to 7·0)
Portugal	64·4 (61·7 to 66·8)	69·0 (65·9 to 71·6)	69·7 (66·4 to 72·5)	69·6 (66·3 to 72·4)	69·5 (66·2 to 72·3)	69·9 (66·4 to 72·7)	19	5·4 (4·6 to 6·2)	8·4 (7·3 to 9·4)
San Marino	68·2 (65·4 to 70·6)	72·3 (69·1 to 75·1)	71·7 (68·1 to 74·7)	71·9 (68·4 to 75·0)	71·5 (68·1 to 74·6)	72·2 (68·9 to 75·2)	3	4·1 (3·1 to 4·9)	6·0 (4·7 to 7·1)
Singapore	66·7 (64·1 to 68·8)	72·5 (69·6 to 75·0)	74·5 (71·5 to 77·2)	74·2 (71·3 to 76·8)	73·6 (70·7 to 76·2)	74·1 (71·2 to 76·8)	1	7·4 (6·8 to 8·1)	11·1 (10·3 to 12·0)
South Korea	62·4 (59·6 to 64·6)	69·9 (66·9 to 72·4)	72·0 (68·9 to 74·7)	72·0 (68·9 to 74·7)	71·3 (68·3 to 74·0)	71·8 (68·8 to 74·5)	4	9·5 (8·7 to 10·2)	15·2 (14·4 to 16·1)
Spain	66·6 (63·8 to 69·0)	70·8 (67·8 to 73·5)	70·6 (67·5 to 73·3)	71·0 (67·8 to 73·7)	70·6 (67·4 to 73·4)	71·1 (67·9 to 73·9)	11	4·5 (3·8 to 5·1)	6·7 (5·8 to 7·4)
Sweden	68·0 (65·3 to 70·4)	70·6 (67·7 to 73·2)	70·5 (67·2 to 73·2)	70·8 (67·5 to 73·6)	70·8 (67·5 to 73·6)	70·9 (67·5 to 73·7)	12	2·8 (2·0 to 3·6)	4·2 (3·1 to 5·2)
Switzerland	67·1 (64·2 to 69·6)	70·6 (67·4 to 73·4)	70·8 (67·6 to 73·7)	71·1 (67·7 to 74·0)	71·0 (67·5 to 74·1)	71·6 (68·0 to 74·6)	5	4·5 (3·8 to 5·2)	6·7 (5·8 to 7·5)
UK	65·8 (63·2 to 68·1)	68·8 (65·7 to 71·5)	68·1 (64·9 to 71·0)	68·1 (64·8 to 71·1)	68·3 (65·0 to 71·3)	68·3 (64·9 to 71·3)	39	2·5 (1·7 to 3·2)	3·8 (2·7 to 4·7)
USA	63·5 (60·5 to 66·3)	65·7 (62·5 to 68·7)	63·8 (60·5 to 66·8)	63·2 (59·9 to 66·2)	64·0 (60·6 to 67·0)	64·5 (61·0 to 67·7)	93	1·0 (0·5 to 1·5)	1·5 (0·8 to 2·3)
Uruguay	63·6 (60·9 to 65·9)	66·2 (63·3 to 68·6)	67·0 (64·0 to 69·5)	64·9 (62·0 to 67·3)	65·6 (62·7 to 68·1)	66·5 (63·4 to 69·0)	60	2·8 (2·3 to 3·4)	4·5 (3·6 to 5·3)
**Latin America and Caribbean**	**59·6 (56·7 to 62·2)**	**62·8 (59·9 to 65·4)**	**62·4 (59·4 to 65·0)**	**61·2 (58·3 to 63·7)**	**63·6 (60·4 to 66·3)**	**64·1 (60·8 to 66·8)**		**4·4 (3·9 to 5·0)**	**7·4 (6·6 to 8·2)**
Antigua and Barbuda	63·3 (60·3 to 65·8)	66·4 (63·1 to 69·1)	66·0 (63·0 to 68·8)	65·3 (62·0 to 68·0)	65·8 (62·4 to 68·8)	66·2 (62·7 to 68·9)	64	2·9 (1·9 to 3·8)	4·6 (3·1 to 6·0)
Bahamas	61·5 (58·8 to 63·8)	62·6 (59·7 to 65·0)	61·5 (58·6 to 63·7)	58·4 (55·7 to 60·6)	61·5 (58·5 to 63·9)	62·5 (59·3 to 65·0)	123	1·0 (0·2 to 1·7)	1·6 (0·3 to 2·8)
Barbados	64·1 (61·3 to 66·6)	66·2 (63·2 to 68·8)	65·7 (62·6 to 68·3)	64·9 (61·9 to 67·4)	64·9 (61·9 to 67·5)	65·6 (62·6 to 68·3)	77	1·5 (0·7 to 2·2)	2·4 (1·2 to 3·4)
Belize	63·2 (60·4 to 65·6)	63·4 (60·4 to 65·8)	62·6 (59·7 to 65·1)	61·2 (58·3 to 63·7)	62·8 (59·9 to 65·5)	64·0 (60·8 to 66·6)	99	0·8 (0·0 to 1·4)	1·2 (0·0 to 2·2)
Bermuda	64·6 (61·8 to 66·8)	69·4 (66·3 to 72·1)	69·3 (66·2 to 72·0)	68·5 (65·3 to 71·2)	68·6 (65·4 to 71·4)	69·0 (65·6 to 71·8)	32	4·4 (3·7 to 5·2)	6·9 (5·8 to 7·9)
Bolivia	54·4 (51·9 to 56·6)	60·1 (57·6 to 62·4)	55·8 (53·5 to 57·8)	56·1 (53·7 to 58·2)	60·6 (57·7 to 63·2)	61·7 (58·8 to 64·3)	131	7·3 (6·5 to 8·0)	13·4 (12·1 to 14·7)
Brazil	59·3 (56·4 to 61·8)	63·7 (60·7 to 66·3)	63·5 (60·4 to 66·3)	61·9 (58·9 to 64·6)	63·9 (60·7 to 66·8)	64·4 (61·0 to 67·3)	95	5·1 (4·4 to 5·8)	8·6 (7·5 to 9·5)
Colombia	61·3 (58·4 to 63·8)	66·6 (63·6 to 69·3)	66·2 (63·1 to 68·7)	64·3 (61·3 to 66·9)	66·8 (63·6 to 69·6)	67·9 (64·7 to 70·7)	44	6·5 (5·9 to 7·2)	10·7 (9·7 to 11·8)
Costa Rica	65·5 (62·4 to 68·1)	67·7 (64·6 to 70·5)	67·0 (63·8 to 69·9)	65·7 (62·6 to 68·4)	66·5 (63·3 to 69·4)	66·9 (63·7 to 69·8)	53	1·4 (0·8 to 2·0)	2·2 (1·3 to 3·0)
Cuba	65·2 (62·4 to 67·6)	67·6 (64·5 to 70·3)	66·8 (63·7 to 69·4)	63·7 (60·8 to 66·0)	66·5 (63·2 to 69·1)	66·9 (63·4 to 69·5)	54	1·7 (0·8 to 2·4)	2·6 (1·3 to 3·6)
Dominica	64·2 (61·2 to 66·7)	64·9 (61·7 to 67·5)	64·5 (61·3 to 67·1)	63·6 (60·5 to 66·2)	64·7 (61·4 to 67·3)	64·9 (61·5 to 67·6)	89	0·8 (0·0 to 1·5)	1·2 (0·1 to 2·3)
Dominican Republic	61·1 (58·3 to 63·5)	64·3 (61·2 to 66·9)	61·8 (58·7 to 64·2)	61·8 (58·7 to 64·2)	62·0 (58·9 to 64·4)	62·5 (59·2 to 64·9)	122	1·4 (0·6 to 2·1)	2·3 (0·9 to 3·5)
Ecuador	61·3 (58·6 to 63·6)	65·6 (62·9 to 68·1)	63·7 (61·0 to 66·2)	64·3 (61·4 to 66·8)	65·6 (62·6 to 68·3)	65·6 (62·6 to 68·3)	75	4·3 (3·5 to 4·9)	7·0 (5·9 to 7·9)
El Salvador	58·5 (55·7 to 60·9)	62·9 (59·9 to 65·4)	61·1 (58·4 to 63·5)	60·5 (57·7 to 62·9)	62·1 (59·3 to 64·6)	62·5 (59·6 to 65·0)	121	4·0 (3·4 to 4·7)	6·9 (5·8 to 8·2)
Grenada	61·0 (58·2 to 63·4)	64·3 (61·4 to 66·7)	63·7 (60·7 to 66·4)	61·8 (58·9 to 64·4)	63·3 (60·3 to 66·1)	63·9 (60·8 to 66·7)	101	3·0 (2·2 to 3·8)	4·9 (3·5 to 6·1)
Guatemala	52·8 (50·1 to 55·0)	60·4 (57·4 to 63·1)	60·2 (57·2 to 62·7)	58·3 (55·4 to 60·7)	60·5 (57·4 to 63·2)	61·5 (58·0 to 64·4)	133	8·7 (7·7 to 9·8)	16·5 (14·7 to 18·1)
Guyana	55·6 (53·1 to 58·1)	58·2 (55·7 to 60·6)	59·6 (56·8 to 62·2)	57·3 (54·7 to 59·7)	58·5 (55·8 to 61·0)	59·0 (56·3 to 61·4)	155	3·4 (2·3 to 4·5)	6·1 (4·1 to 8·2)
Haiti	44·2 (42·1 to 46·1)	27·3 (25·6 to 28·9)	52·8 (50·3 to 54·8)	52·1 (49·5 to 54·2)	52·5 (49·8 to 54·7)	52·3 (50·0 to 54·4)	196	8·2 (7·2 to 9·2)	18·5 (16·3 to 20·7)
Honduras	60·3 (57·5 to 62·7)	63·3 (60·6 to 65·9)	62·0 (59·1 to 64·4)	60·4 (57·6 to 62·9)	64·0 (60·9 to 66·8)	64·6 (61·5 to 67·5)	91	4·4 (3·7 to 5·0)	7·3 (6·2 to 8·4)
Jamaica	64·6 (61·7 to 67·0)	66·4 (63·3 to 69·0)	64·0 (61·0 to 66·6)	62·7 (59·7 to 65·2)	64·1 (61·1 to 66·8)	65·0 (61·9 to 67·8)	88	0·4 (-0·4 to 1·4)	0·7 (-0·7 to 2·2)
Mexico	59·6 (56·4 to 62·4)	63·6 (60·4 to 66·4)	60·4 (57·4 to 62·9)	59·9 (56·9 to 62·5)	62·9 (59·5 to 65·7)	63·7 (60·2 to 66·6)	104	4·1 (3·6 to 4·5)	6·9 (6·2 to 7·6)
Nicaragua	60·5 (57·5 to 63·0)	64·2 (61·3 to 66·7)	61·8 (58·9 to 64·1)	61·3 (58·4 to 63·6)	63·9 (60·9 to 66·5)	64·9 (61·7 to 67·9)	90	4·4 (3·6 to 5·3)	7·3 (5·9 to 8·7)
Panama	65·3 (62·4 to 67·9)	66·8 (63·8 to 69·5)	65·4 (62·5 to 68·0)	65·5 (62·5 to 68·2)	66·7 (63·6 to 69·4)	67·1 (64·0 to 69·9)	51	1·8 (1·2 to 2·4)	2·8 (1·9 to 3·6)
Paraguay	64·4 (61·5 to 67·0)	63·6 (60·7 to 66·1)	62·1 (59·1 to 64·5)	58·1 (55·5 to 60·3)	61·9 (58·9 to 64·5)	62·3 (59·4 to 64·9)	124	-2·1 (-2·8 to -1·5)	-3·3 (-4·4 to -2·3)
Peru	62·3 (59·2 to 64·9)	67·9 (64·8 to 70·5)	62·7 (59·8 to 65·0)	61·4 (58·7 to 63·8)	66·6 (63·6 to 69·4)	66·0 (63·0 to 68·5)	70	3·7 (3·1 to 4·3)	6·0 (5·0 to 7·2)
Puerto Rico	63·9 (60·9 to 66·5)	67·5 (64·3 to 70·3)	68·3 (64·9 to 71·1)	67·8 (64·4 to 70·7)	67·7 (64·2 to 70·4)	68·3 (64·7 to 71·3)	38	4·3 (3·6 to 5·1)	6·8 (5·7 to 7·9)
Saint Kitts and Nevis	59·4 (56·6 to 61·8)	63·4 (60·5 to 65·9)	63·6 (60·5 to 66·1)	62·7 (59·7 to 65·2)	63·5 (60·3 to 66·1)	63·9 (60·8 to 66·6)	102	4·4 (3·6 to 5·3)	7·5 (6·1 to 8·9)
Saint Lucia	60·7 (57·8 to 63·1)	64·6 (61·5 to 67·4)	65·2 (62·0 to 68·0)	63·3 (60·2 to 65·9)	64·9 (61·4 to 67·8)	65·6 (62·1 to 68·4)	78	4·9 (3·8 to 6·0)	8·0 (6·3 to 9·6)
Saint Vincent and the Grenadines	61·7 (58·8 to 64·1)	63·2 (59·9 to 65·8)	63·4 (60·3 to 66·1)	63·3 (60·1 to 65·9)	63·7 (60·3 to 66·4)	64·0 (60·7 to 66·8)	100	2·3 (1·3 to 3·2)	3·8 (2·1 to 5·2)
Suriname	60·8 (58·0 to 63·2)	62·0 (59·0 to 64·6)	61·6 (58·4 to 64·0)	58·2 (55·3 to 60·5)	61·3 (58·0 to 63·8)	62·0 (58·7 to 64·7)	128	1·2 (0·3 to 2·0)	1·9 (0·6 to 3·2)
Trinidad and Tobago	59·4 (56·6 to 61·6)	62·4 (59·1 to 65·0)	62·1 (59·0 to 64·6)	59·5 (56·6 to 61·8)	60·9 (57·9 to 63·5)	62·2 (59·0 to 64·8)	127	2·8 (1·9 to 3·5)	4·7 (3·3 to 5·9)
Venezuela	61·5 (58·6 to 63·8)	63·8 (60·8 to 66·3)	61·5 (58·4 to 63·9)	60·6 (57·6 to 63·0)	61·2 (58·2 to 63·7)	61·3 (58·4 to 63·9)	135	-0·2 (-0·8 to 0·4)	-0·3 (-1·3 to 0·6)
Virgin Islands	62·5 (59·9 to 64·9)	65·6 (62·3 to 68·1)	66·1 (62·9 to 68·9)	64·6 (61·6 to 67·2)	65·2 (62·1 to 68·2)	65·3 (62·4 to 68·3)	81	2·8 (1·7 to 3·9)	4·5 (2·8 to 6·0)
**North Africa and Middle East**	**56·2 (53·6 to 58·4)**	**62·0 (59·1 to 64·5)**	**61·3 (58·4 to 63·8)**	**61·0 (58·0 to 63·5)**	**62·6 (59·5 to 65·1)**	**62·7 (59·7 to 65·2)**		**6·6 (5·9 to 7·2)**	**11·7 (10·6 to 12·7)**
Afghanistan	48·1 (44·5 to 51·2)	54·0 (50·5 to 56·8)	53·1 (49·7 to 56·0)	50·8 (47·6 to 53·7)	55·6 (52·3 to 58·4)	56·2 (52·9 to 59·1)	173	8·2 (6·9 to 9·6)	17·0 (14·1 to 20·4)
Algeria	59·2 (56·5 to 61·4)	64·3 (61·2 to 66·7)	64·2 (61·2 to 66·6)	64·3 (61·4 to 66·8)	65·0 (62·0 to 67·5)	65·2 (62·1 to 67·7)	85	6·0 (5·3 to 6·7)	10·2 (9·1 to 11·1)
Bahrain	60·7 (58·0 to 62·9)	64·7 (61·7 to 67·2)	65·3 (62·2 to 67·9)	64·8 (61·7 to 67·4)	65·8 (62·6 to 68·6)	66·1 (63·1 to 68·8)	66	5·4 (4·4 to 6·2)	8·9 (7·3 to 10·2)
Egypt	55·0 (52·6 to 57·1)	59·5 (57·1 to 61·7)	59·5 (56·8 to 61·8)	58·7 (56·1 to 61·0)	59·8 (57·0 to 62·2)	60·3 (57·4 to 62·6)	145	5·2 (4·3 to 6·0)	9·5 (8·0 to 10·9)
Iran	56·7 (54·0 to 59·0)	65·0 (61·9 to 67·7)	63·1 (60·0 to 65·8)	63·4 (60·2 to 66·2)	65·8 (62·4 to 68·7)	66·3 (62·8 to 69·3)	63	9·6 (8·7 to 10·4)	17·0 (15·7 to 18·2)
Iraq	56·9 (54·1 to 59·1)	58·6 (55·9 to 61·0)	57·6 (54·8 to 59·9)	58·3 (55·4 to 60·7)	60·0 (57·0 to 62·6)	60·5 (57·6 to 62·9)	143	3·6 (2·9 to 4·3)	6·3 (5·2 to 7·6)
Jordan	61·4 (58·7 to 63·7)	64·6 (61·8 to 67·1)	64·7 (61·9 to 67·2)	63·4 (60·7 to 65·9)	66·2 (63·1 to 68·9)	67·1 (63·9 to 69·9)	52	5·7 (4·9 to 6·4)	9·2 (8·1 to 10·2)

Kuwait	62·3 (59·6 to 64·7)	66·7 (63·7 to 69·4)	66·9 (63·9 to 69·6)	66·8 (63·7 to 69·5)	68·1 (64·8 to 71·1)	68·7 (65·2 to 71·7)	34	6·4 (5·4 to 7·2)	10·2 (9·0 to 11·2)
Lebanon	62·8 (59·3 to 65·7)	68·0 (64·6 to 71·0)	62·1 (59·1 to 64·7)	64·7 (61·4 to 67·6)	65·6 (62·2 to 68·4)	66·2 (62·6 to 69·3)	65	3·3 (2·2 to 4·6)	5·3 (3·6 to 7·7)
Libya	60·9 (58·2 to 63·2)	63·7 (60·7 to 66·2)	61·5 (58·4 to 64·1)	59·9 (57·0 to 62·3)	63·1 (59·9 to 65·8)	59·7 (56·6 to 62·3)	150	-1·2 (-2·1 to -0·5)	-2·0 (-3·4 to -0·8)
Morocco	56·4 (53·8 to 58·4)	61·4 (58·5 to 63·7)	61·8 (58·9 to 64·0)	61·6 (58·8 to 64·1)	62·6 (59·5 to 65·1)	63·0 (60·0 to 65·5)	115	6·6 (5·7 to 7·6)	11·7 (10·1 to 13·6)
Oman	59·2 (56·6 to 61·5)	63·9 (61·2 to 66·3)	63·6 (60·8 to 66·0)	62·7 (59·9 to 65·1)	65·5 (62·6 to 68·1)	66·1 (63·1 to 68·7)	67	6·9 (6·1 to 7·6)	11·6 (10·4 to 12·8)
Palestine	60·7 (57·8 to 63·0)	63·8 (60·7 to 66·3)	64·7 (61·6 to 67·3)	63·4 (60·4 to 66·0)	65·1 (61·9 to 67·7)	56·1 (53·3 to 58·7)	175	-4·6 (-5·6 to -3·7)	-7·6 (-9·1 to -6·1)
Qatar	62·6 (60·0 to 64·8)	67·0 (64·1 to 69·6)	68·9 (65·7 to 71·7)	68·8 (65·5 to 71·7)	69·3 (66·1 to 72·3)	69·5 (66·2 to 72·5)	24	6·9 (6·0 to 7·9)	11·0 (9·7 to 12·3)
Saudi Arabia	58·9 (56·2 to 61·2)	61·6 (58·7 to 64·0)	62·1 (59·1 to 64·5)	62·8 (59·7 to 65·4)	63·3 (60·0 to 65·8)	63·5 (60·4 to 66·0)	106	4·6 (3·7 to 5·3)	7·7 (6·4 to 9·0)
Sudan	48·3 (46·0 to 50·3)	56·7 (54·1 to 58·9)	58·2 (55·4 to 60·5)	58·5 (55·6 to 61·0)	59·5 (56·6 to 62·0)	59·2 (56·3 to 61·5)	153	10·9 (9·8 to 12·0)	22·6 (20·4 to 24·8)
Syria	59·1 (56·3 to 61·5)	63·8 (60·8 to 66·4)	62·2 (58·8 to 65·1)	62·0 (58·6 to 64·9)	63·4 (59·8 to 66·3)	62·8 (59·3 to 65·8)	116	3·7 (2·7 to 4·6)	6·3 (4·6 to 7·7)
Tunisia	59·6 (57·0 to 61·8)	63·7 (61·0 to 66·0)	62·5 (59·8 to 65·0)	60·6 (58·0 to 63·0)	63·9 (60·9 to 66·6)	64·4 (61·4 to 67·0)	94	4·9 (4·1 to 5·4)	8·2 (7·1 to 9·0)
Türkiye	58·7 (56·1 to 61·0)	66·3 (63·5 to 69·0)	65·3 (62·4 to 67·9)	64·5 (61·6 to 67·1)	65·8 (62·7 to 68·4)	65·1 (62·0 to 67·9)	87	6·4 (5·5 to 7·2)	10·9 (9·4 to 12·3)
United Arab Emirates	61·3 (58·8 to 63·5)	66·4 (63·6 to 68·8)	68·4 (65·4 to 71·1)	68·2 (65·1 to 70·9)	68·6 (65·5 to 71·4)	68·6 (65·5 to 71·4)	35	7·3 (6·5 to 8·2)	11·9 (10·8 to 13·0)
Yemen	50·8 (48·2 to 53·1)	58·9 (55·9 to 61·5)	57·3 (54·5 to 59·9)	57·6 (54·5 to 60·3)	59·6 (56·2 to 62·3)	60·3 (57·1 to 63·1)	144	9·5 (8·5 to 10·5)	18·7 (16·7 to 20·6)
**South Asia**	**50·3 (47·7 to 52·4)**	**57·7 (55·0 to 60·0)**	**59·7 (56·7 to 62·1)**	**58·5 (55·6 to 60·8)**	**60·4 (57·4 to 62·9)**	**60·8 (57·9 to 63·3)**		**10·5 (9·7 to 11·3)**	**21·0 (19·4 to 22·5)**
Bangladesh	49·0 (46·6 to 50·9)	59·1 (56·4 to 61·4)	60·0 (57·1 to 62·3)	58·9 (56·3 to 61·2)	61·0 (58·2 to 63·5)	61·3 (58·6 to 63·6)	137	12·3 (11·3 to 13·4)	25·1 (23·2 to 27·2)
Bhutan	51·7 (49·5 to 53·8)	60·2 (57·6 to 62·4)	62·6 (59·9 to 64·9)	62·8 (60·0 to 65·2)	62·7 (59·9 to 65·1)	62·8 (60·2 to 65·2)	118	11·1 (10·3 to 12·2)	21·5 (19·8 to 23·4)
India	50·1 (47·5 to 52·2)	57·8 (55·0 to 60·0)	60·1 (57·1 to 62·6)	58·8 (55·9 to 61·2)	60·6 (57·6 to 63·2)	61·0 (58·1 to 63·5)	139	10·9 (10·0 to 11·8)	21·9 (20·1 to 23·7)
Nepal	49·5 (47·0 to 51·6)	60·6 (57·8 to 62·8)	62·1 (59·2 to 64·4)	60·3 (57·6 to 62·6)	62·8 (59·7 to 65·5)	63·0 (60·0 to 65·5)	114	13·5 (12·4 to 14·6)	27·2 (25·1 to 29·6)
Pakistan	53·5 (51·0 to 55·7)	56·9 (54·2 to 59·2)	57·1 (54·1 to 59·4)	56·5 (53·5 to 59·1)	59·1 (55·9 to 61·7)	59·6 (56·2 to 62·2)	151	6·1 (5·0 to 7·1)	11·4 (9·6 to 13·2)
**Southeast Asia, East Asia, and Oceania**	**58·3 (56·1 to 60·3)**	**65·0 (62·4 to 67·2)**	**67·8 (65·0 to 70·1)**	**66·9 (64·3 to 69·2)**	**67·2 (64·5 to 69·6)**	**67·3 (64·6 to 69·9)**		**9·0 (8·2 to 9·7)**	**15·3 (14·4 to 16·3)**
American Samoa	60·8 (58·3 to 62·9)	61·2 (58·6 to 63·3)	61·1 (58·3 to 63·3)	59·3 (56·7 to 61·4)	61·1 (58·5 to 63·6)	61·5 (58·8 to 64·0)	132	0·7 (-0·2 to 1·5)	1·1 (-0·3 to 2·4)
Cambodia	50·4 (48·0 to 52·3)	61·0 (58·3 to 63·2)	63·9 (61·2 to 66·3)	63·5 (60·6 to 65·8)	63·8 (60·9 to 66·1)	63·6 (60·8 to 65·8)	105	13·2 (12·2 to 14·1)	26·2 (24·0 to 28·3)
China	58·7 (56·6 to 60·7)	66·4 (63·7 to 68·7)	70·0 (67·1 to 72·5)	69·7 (66·9 to 72·1)	69·4 (66·6 to 72·0)	69·5 (66·7 to 72·3)	23	10·8 (9·8 to 11·8)	18·4 (17·1 to 19·6)
Cook Islands	60·0 (57·5 to 61·9)	63·0 (60·4 to 65·3)	63·3 (60·6 to 65·6)	62·2 (59·5 to 64·4)	63·1 (60·3 to 65·5)	63·3 (60·3 to 65·7)	109	3·3 (2·6 to 4·0)	5·5 (4·4 to 6·6)
Federated States of Micronesia	55·0 (53·1 to 56·7)	57·6 (55·3 to 59·6)	57·9 (55·3 to 59·9)	56·7 (54·2 to 58·7)	57·9 (55·2 to 60·0)	58·2 (55·6 to 60·4)	159	3·2 (2·5 to 3·9)	5·8 (4·6 to 7·0)
Fiji	58·9 (56·4 to 61·0)	58·9 (56·5 to 61·0)	59·3 (56·7 to 61·4)	57·8 (55·3 to 59·9)	58·7 (56·2 to 61·1)	58·7 (56·1 to 61·0)	157	-0·2 (-0·8 to 0·4)	-0·3 (-1·4 to 0·8)
Guam	65·4 (63·0 to 67·5)	67·1 (64·4 to 69·4)	65·9 (63·1 to 68·2)	64·9 (62·2 to 67·3)	65·4 (62·6 to 67·8)	65·8 (62·8 to 68·2)	72	0·4 (-0·3 to 1·0)	0·7 (-0·4 to 1·6)
Indonesia	56·6 (54·2 to 58·8)	61·5 (59·0 to 63·5)	62·5 (60·1 to 64·7)	60·3 (58·0 to 62·3)	63·2 (60·5 to 65·4)	63·2 (60·6 to 65·9)	110	6·6 (5·7 to 7·6)	11·7 (10·2 to 13·3)
Kiribati	56·0 (53·4 to 58·1)	59·5 (56·8 to 61·7)	60·7 (57·7 to 63·0)	59·5 (56·5 to 61·7)	60·7 (57·6 to 63·1)	61·0 (57·8 to 63·4)	140	5·0 (4·2 to 5·7)	9·0 (7·6 to 10·2)
Laos	44·9 (42·9 to 46·6)	56·9 (54·8 to 58·6)	58·9 (56·8 to 60·8)	58·9 (56·9 to 60·7)	58·6 (56·4 to 60·6)	58·7 (56·4 to 60·8)	156	13·9 (12·8 to 15·1)	31·0 (28·4 to 34·1)
Malaysia	62·9 (60·7 to 64·9)	65·8 (63·3 to 68·1)	67·1 (64·4 to 69·3)	64·4 (62·1 to 66·5)	65·8 (63·3 to 68·0)	66·8 (64·3 to 69·2)	55	3·9 (3·4 to 4·4)	6·3 (5·5 to 7·0)
Maldives	57·0 (54·7 to 58·9)	66·0 (63·5 to 68·1)	67·2 (64·6 to 69·3)	66·4 (63·8 to 68·5)	66·8 (64·3 to 69·0)	67·2 (64·7 to 69·4)	49	10·2 (9·6 to 10·8)	17·9 (16·9 to 19·0)
Marshall Islands	54·2 (52·1 to 56·0)	55·4 (53·2 to 57·4)	55·9 (53·5 to 57·8)	54·8 (52·5 to 56·6)	55·9 (53·7 to 57·9)	56·2 (53·8 to 58·1)	174	1·9 (1·3 to 2·5)	3·5 (2·4 to 4·5)
Mauritius	61·1 (58·6 to 63·2)	64·2 (61·5 to 66·6)	64·4 (61·5 to 66·8)	63·0 (60·3 to 65·3)	63·4 (60·6 to 65·7)	63·7 (60·9 to 66·2)	103	2·6 (2·0 to 3·3)	4·3 (3·3 to 5·3)
Myanmar	47·9 (45·8 to 49·6)	55·9 (53·7 to 57·8)	57·8 (55·5 to 59·8)	56·4 (54·1 to 58·4)	57·5 (55·0 to 59·7)	57·4 (55·0 to 59·6)	162	9·5 (8·5 to 10·5)	19·8 (17·7 to 22·1)
Nauru	51·7 (49·7 to 53·2)	50·8 (48·9 to 52·4)	51·7 (49·7 to 53·5)	49·9 (47·9 to 51·6)	51·8 (49·7 to 53·6)	52·1 (49·9 to 54·0)	198	0·5 (-0·3 to 1·1)	0·9 (-0·6 to 2·1)
Niue	61·2 (59·0 to 63·3)	62·7 (60·2 to 64·9)	63·5 (60·8 to 65·7)	62·1 (59·6 to 64·2)	62·8 (60·2 to 65·0)	63·0 (60·4 to 65·2)	113	1·8 (1·3 to 2·2)	2·9 (2·1 to 3·6)
North Korea	62·9 (60·3 to 65·0)	63·9 (61·3 to 66·0)	64·3 (61·5 to 66·5)	64·5 (61·7 to 66·7)	64·3 (61·6 to 66·6)	64·3 (61·4 to 66·7)	97	1·4 (0·5 to 2·2)	2·2 (0·8 to 3·5)
Northern Mariana Islands	60·5 (58·3 to 62·5)	61·6 (59·2 to 63·6)	61·5 (58·9 to 63·6)	60·3 (57·8 to 62·3)	61·5 (59·0 to 63·6)	61·9 (59·5 to 64·2)	129	1·4 (0·8 to 2·0)	2·3 (1·3 to 3·3)
Palau	58·4 (56·1 to 60·3)	59·7 (57·2 to 61·8)	59·7 (57·1 to 61·8)	58·4 (55·9 to 60·4)	59·7 (57·0 to 62·0)	59·9 (57·2 to 62·3)	147	1·5 (0·9 to 2·1)	2·6 (1·5 to 3·6)
Papua New Guinea	54·3 (51·8 to 56·4)	56·6 (54·3 to 58·6)	58·2 (55·8 to 60·3)	57·6 (55·2 to 59·8)	57·8 (55·4 to 60·1)	58·0 (55·4 to 60·2)	160	3·7 (2·7 to 4·5)	6·8 (5·0 to 8·4)
Philippines	60·1 (57·7 to 62·1)	62·3 (60·0 to 64·4)	63·1 (60·7 to 65·1)	59·9 (57·8 to 61·8)	62·1 (59·8 to 64·2)	63·2 (60·8 to 65·2)	111	3·1 (2·6 to 3·5)	5·1 (4·4 to 6·0)
Samoa	62·3 (59·8 to 64·6)	63·5 (60·9 to 65·7)	63·2 (60·5 to 65·4)	62·0 (59·4 to 64·1)	63·0 (60·3 to 65·4)	63·0 (60·2 to 65·5)	112	0·7 (0·1 to 1·2)	1·2 (0·2 to 2·0)
Seychelles	62·3 (59·9 to 64·3)	64·6 (62·0 to 66·7)	66·4 (63·7 to 68·7)	63·5 (60·9 to 65·7)	65·7 (63·0 to 67·9)	65·7 (63·0 to 67·9)	73	3·4 (2·6 to 4·0)	5·4 (4·3 to 6·4)
Solomon Islands	54·9 (53·0 to 56·6)	57·3 (55·1 to 59·0)	56·5 (54·3 to 58·2)	56·0 (53·9 to 57·7)	56·3 (54·2 to 58·1)	56·5 (54·4 to 58·2)	170	1·5 (1·0 to 2·1)	2·8 (1·7 to 3·8)
Sri Lanka	60·8 (58·4 to 62·8)	64·9 (62·4 to 67·1)	68·1 (65·4 to 70·5)	66·8 (64·2 to 68·9)	66·8 (64·1 to 69·1)	66·8 (64·1 to 69·1)	56	6·1 (5·5 to 6·7)	10·0 (9·1 to 11·0)
Taiwan*	65·1 (62·6 to 67·3)	68·8 (65·8 to 71·3)	69·7 (66·8 to 72·4)	69·6 (66·6 to 72·2)	68·8 (65·8 to 71·4)	69·1 (66·0 to 71·7)	28	4·0 (3·3 to 4·6)	6·1 (5·1 to 6·9)
Thailand	62·6 (60·1 to 64·8)	65·7 (63·2 to 67·9)	67·7 (65·0 to 70·0)	66·8 (64·2 to 69·1)	66·6 (64·0 to 68·9)	67·3 (64·6 to 69·7)	47	4·7 (4·3 to 5·2)	7·6 (6·9 to 8·3)
Timor-Leste	50·2 (48·1 to 52·0)	60·1 (57·8 to 62·1)	60·8 (58·6 to 62·9)	60·7 (58·3 to 62·7)	61·1 (58·7 to 63·3)	61·3 (58·9 to 63·6)	136	11·1 (10·2 to 12·0)	22·1 (20·2 to 24·0)
Tokelau	62·8 (60·2 to 65·1)	64·6 (61·8 to 67·0)	65·8 (62·7 to 68·2)	64·6 (61·6 to 67·0)	65·0 (62·0 to 67·5)	65·3 (62·2 to 67·7)	83	2·4 (1·8 to 3·0)	3·9 (2·9 to 4·8)
Tonga	61·8 (59·2 to 63·9)	62·7 (60·0 to 64·9)	62·4 (59·4 to 64·7)	60·8 (58·0 to 63·0)	62·0 (59·1 to 64·4)	62·3 (59·2 to 64·7)	125	0·5 (-0·2 to 1·1)	0·9 (-0·3 to 1·8)
Tuvalu	52·5 (50·3 to 54·2)	55·3 (53·0 to 57·1)	55·7 (53·3 to 57·6)	54·4 (52·1 to 56·3)	55·6 (53·1 to 57·5)	55·7 (53·4 to 57·5)	177	3·2 (2·6 to 3·9)	6·1 (4·8 to 7·3)
Vanuatu	58·4 (56·1 to 60·3)	61·0 (58·5 to 62·9)	61·2 (58·7 to 63·2)	60·6 (58·1 to 62·6)	61·2 (58·7 to 63·3)	61·4 (58·9 to 63·5)	134	3·0 (2·4 to 3·6)	5·2 (4·2 to 6·2)
Viet Nam	60·5 (58·1 to 62·7)	65·2 (62·7 to 67·3)	66·6 (63·9 to 68·9)	66·0 (63·3 to 68·1)	66·4 (63·8 to 68·7)	66·7 (64·1 to 69·0)	57	6·1 (5·3 to 7·0)	10·2 (8·7 to 11·7)
**Sub-Saharan Africa**	**44·9 (42·8 to 46·7)**	**51·1 (48·8 to 53·1)**	**53·9 (51·5 to 56·0)**	**52·9 (50·5 to 54·9)**	**54·7 (52·1 to 56·8)**	**55·2 (52·7 to 57·4)**		**10·3 (9·6 to 10·9)**	**23·0 (21·7 to 24·2)**

Angola	42·6 (40·5 to 44·6)	51·4 (49·0 to 53·5)	53·7 (51·0 to 55·9)	52·8 (50·1 to 55·1)	54·5 (51·6 to 57·1)	54·8 (52·2 to 57·3)	182	12·2 (11·0 to 13·5)	28·7 (26·1 to 31·5)
Benin	46·6 (44·4 to 48·6)	54·0 (51·4 to 56·2)	56·2 (53·7 to 58·5)	56·4 (53·9 to 58·7)	56·8 (54·3 to 59·2)	57·1 (54·4 to 59·5)	163	10·5 (9·5 to 11·5)	22·5 (20·5 to 24·8)
Botswana	55·4 (53·0 to 57·5)	56·1 (53·5 to 58·5)	59·0 (56·3 to 61·5)	56·6 (54·1 to 58·9)	59·2 (56·4 to 61·6)	59·9 (57·1 to 62·4)	149	4·4 (3·7 to 5·2)	8·0 (6·7 to 9·3)
Burkina Faso	42·9 (40·6 to 44·8)	54·5 (52·0 to 56·7)	57·8 (55·1 to 60·1)	57·4 (54·5 to 59·9)	58·1 (55·0 to 60·6)	58·2 (55·5 to 60·7)	158	15·4 (14·2 to 16·8)	35·9 (33·1 to 39·5)
Burundi	42·0 (40·1 to 43·6)	51·0 (48·6 to 52·9)	52·9 (50·5 to 54·9)	51·4 (49·3 to 53·3)	53·1 (50·9 to 55·1)	53·4 (51·2 to 55·6)	190	11·5 (10·4 to 12·7)	27·4 (24·9 to 30·1)
Cabo Verde	60·2 (57·4 to 62·4)	64·9 (62·0 to 67·4)	63·3 (60·8 to 65·8)	62·2 (59·3 to 64·9)	64·4 (61·4 to 67·1)	65·3 (62·4 to 67·7)	82	5·1 (4·1 to 6·0)	8·5 (6·8 to 10·0)
Cameroon	47·3 (45·0 to 49·3)	48·4 (46·4 to 50·2)	52·2 (50·1 to 54·2)	51·2 (49·2 to 53·2)	53·2 (50·9 to 55·3)	53·6 (51·5 to 55·7)	189	6·3 (5·5 to 7·3)	13·4 (11·5 to 15·8)
Central African Republic	41·0 (38·7 to 42·9)	43·4 (41·3 to 45·3)	45·4 (43·1 to 47·4)	45·2 (42·6 to 47·4)	45·8 (43·3 to 47·9)	46·3 (44·1 to 48·5)	204	5·3 (4·4 to 6·3)	13·0 (10·6 to 15·5)
Chad	41·8 (39·9 to 43·4)	47·3 (45·3 to 49·0)	48·9 (46·7 to 50·7)	49·8 (47·5 to 51·8)	49·9 (47·5 to 51·7)	50·2 (47·9 to 52·3)	201	8·4 (7·3 to 9·4)	20·1 (17·5 to 22·6)
Comoros	57·2 (54·6 to 59·5)	64·5 (61·4 to 67·0)	63·9 (60·9 to 66·3)	61·7 (58·8 to 64·1)	64·5 (61·4 to 67·1)	65·4 (62·2 to 68·0)	80	8·1 (7·2 to 9·2)	14·2 (12·4 to 16·1)
Congo (Brazzaville)	47·5 (45·0 to 49·5)	53·2 (50·8 to 55·4)	53·7 (51·3 to 56·0)	52·9 (50·5 to 55·1)	54·2 (51·7 to 56·4)	54·6 (51·9 to 57·1)	184	7·1 (6·1 to 8·1)	14·9 (13·1 to 17·0)
Côte d'Ivoire	46·5 (44·1 to 48·4)	51·4 (49·2 to 53·4)	54·4 (52·0 to 56·6)	54·3 (51·7 to 56·6)	54·9 (52·3 to 57·2)	55·4 (52·8 to 57·8)	179	9·0 (8·0 to 10·0)	19·3 (16·9 to 21·8)
DR Congo	44·1 (41·8 to 46·1)	48·6 (46·3 to 50·7)	51·0 (48·6 to 53·1)	50·9 (48·4 to 53·1)	51·7 (49·3 to 54·1)	52·5 (50·0 to 54·9)	195	8·3 (7·4 to 9·4)	18·9 (16·4 to 21·2)
Djibouti	53·8 (51·3 to 55·7)	56·7 (54·3 to 59·0)	58·5 (55·9 to 60·6)	56·9 (54·5 to 59·1)	58·9 (56·4 to 61·4)	59·5 (56·9 to 61·8)	152	5·7 (4·6 to 6·8)	10·6 (8·7 to 12·6)
Equatorial Guinea	42·0 (39·8 to 43·9)	51·4 (49·2 to 53·6)	51·8 (49·5 to 54·0)	51·4 (48·9 to 53·5)	52·6 (49·9 to 54·8)	53·0 (50·5 to 55·3)	192	11·0 (9·9 to 12·1)	26·1 (23·2 to 28·9)
Eritrea	38·2 (35·9 to 40·2)	51·5 (48·8 to 53·7)	53·3 (50·7 to 55·4)	53·2 (50·5 to 55·3)	52·8 (50·3 to 54·9)	54·1 (51·5 to 56·6)	186	15·9 (14·6 to 17·2)	41·7 (37·5 to 46·5)
Eswatini	49·6 (47·6 to 51·3)	41·6 (39·7 to 43·4)	49·1 (46·9 to 51·1)	47·8 (45·5 to 49·8)	50·8 (48·2 to 52·9)	52·1 (49·5 to 54·2)	197	2·5 (1·2 to 3·7)	5·0 (2·6 to 7·4)
Ethiopia	38·6 (36·7 to 40·2)	52·8 (50·7 to 54·6)	55·5 (53·1 to 57·6)	54·0 (51·6 to 56·1)	55·8 (53·3 to 58·1)	56·3 (54·0 to 58·3)	172	17·7 (16·7 to 18·9)	46·0 (43·2 to 48·7)
Gabon	53·1 (50·6 to 55·3)	55·4 (52·8 to 57·8)	55·7 (53·1 to 58·1)	55·1 (52·3 to 57·7)	56·5 (53·6 to 59·2)	57·1 (54·4 to 59·6)	164	4·0 (3·0 to 4·9)	7·5 (5·6 to 9·4)
Ghana	48·5 (46·3 to 50·5)	54·4 (51·8 to 56·5)	55·9 (53·4 to 57·9)	55·2 (52·6 to 57·3)	56·3 (53·7 to 58·6)	56·8 (53·8 to 59·2)	168	8·3 (7·2 to 9·3)	17·1 (14·7 to 19·3)
Guinea	42·1 (40·2 to 44·0)	50·8 (48·6 to 52·8)	52·8 (50·6 to 54·9)	52·7 (50·2 to 54·8)	53·4 (50·9 to 55·7)	54·1 (51·6 to 56·4)	187	12·0 (10·8 to 13·1)	28·5 (25·6 to 31·4)
Guinea-Bissau	43·7 (41·5 to 45·5)	49·8 (47·5 to 51·9)	51·2 (48·7 to 53·2)	51·4 (48·8 to 53·5)	52·1 (49·6 to 54·3)	52·8 (50·3 to 54·9)	193	9·2 (8·2 to 10·2)	21·0 (18·3 to 23·4)
Kenya	53·9 (51·7 to 55·9)	56·0 (53·7 to 58·2)	57·8 (55·3 to 60·0)	56·5 (54·0 to 58·7)	59·8 (56·9 to 62·1)	60·5 (57·9 to 62·8)	142	6·6 (5·6 to 7·6)	12·2 (10·3 to 13·9)
Lesotho	51·6 (49·5 to 53·4)	46·1 (44·0 to 47·8)	48·5 (46·2 to 50·5)	47·3 (45·0 to 49·2)	49·1 (46·6 to 51·2)	49·8 (47·4 to 51·9)	202	-1·8 (-2·9 to -0·8)	-3·5 (-5·5 to -1·5)
Liberia	39·7 (37·7 to 41·5)	49·2 (46·6 to 51·5)	49·9 (47·3 to 52·0)	48·3 (46·0 to 50·3)	52·0 (49·2 to 54·1)	52·6 (49·7 to 55·0)	194	12·9 (11·9 to 14·0)	32·6 (29·8 to 35·6)
Madagascar	45·4 (43·3 to 47·1)	53·4 (51·3 to 55·1)	54·0 (51·8 to 56·0)	53·2 (51·0 to 55·1)	54·1 (51·9 to 56·2)	54·8 (52·9 to 56·8)	183	9·5 (8·6 to 10·4)	20·9 (18·5 to 23·1)
Malawi	39·9 (38·0 to 41·5)	50·0 (47·6 to 51·8)	53·4 (50·9 to 55·4)	48·9 (46·7 to 50·6)	53·3 (50·7 to 55·4)	54·0 (51·6 to 56·2)	188	14·1 (12·9 to 15·3)	35·3 (32·6 to 38·2)
Mali	41·3 (39·1 to 43·2)	52·1 (49·4 to 54·4)	52·5 (50·0 to 54·8)	52·0 (49·5 to 54·3)	53·5 (51·0 to 55·8)	54·2 (51·5 to 56·6)	185	13·0 (11·8 to 14·2)	31·5 (28·4 to 34·6)
Mauritania	52·7 (50·3 to 54·7)	57·5 (55·0 to 59·6)	57·4 (54·7 to 59·6)	56·7 (53·9 to 58·8)	58·0 (55·4 to 60·3)	59·1 (56·6 to 61·3)	154	6·5 (5·4 to 7·4)	12·3 (10·4 to 14·0)
Mozambique	44·4 (42·0 to 46·4)	51·3 (48·7 to 53·5)	53·4 (50·6 to 55·8)	51·7 (49·0 to 53·9)	54·3 (51·3 to 56·7)	55·1 (51·9 to 57·7)	181	10·7 (9·3 to 11·9)	24·1 (21·4 to 26·9)
Namibia	52·3 (49·9 to 54·2)	53·9 (51·3 to 55·9)	55·5 (53·0 to 57·6)	51·1 (48·9 to 52·8)	55·9 (53·5 to 58·0)	57·1 (54·5 to 59·3)	165	4·8 (4·0 to 5·5)	9·1 (7·7 to 10·4)
Niger	36·9 (34·8 to 38·4)	48·5 (46·5 to 50·2)	50·0 (48·0 to 51·8)	50·4 (48·3 to 52·3)	51·0 (48·8 to 53·1)	51·4 (49·2 to 53·6)	199	14·5 (13·5 to 15·9)	39·4 (36·2 to 43·2)
Nigeria	44·1 (41·7 to 46·1)	51·1 (48·5 to 53·1)	54·0 (51·3 to 56·3)	54·0 (51·4 to 56·3)	54·9 (52·1 to 57·2)	55·2 (52·5 to 57·6)	180	11·1 (10·0 to 12·2)	25·2 (22·5 to 27·9)
Rwanda	40·7 (39·0 to 42·2)	54·1 (51·7 to 56·2)	56·6 (54·4 to 58·8)	55·3 (53·0 to 57·4)	56·3 (53·8 to 58·4)	56·4 (53·9 to 58·4)	171	15·7 (14·6 to 17·0)	38·7 (36·1 to 41·8)
Senegal	49·4 (47·0 to 51·5)	57·8 (55·1 to 60·1)	58·9 (56·1 to 61·4)	57·6 (54·9 to 60·0)	59·7 (56·9 to 62·2)	60·2 (57·5 to 62·8)	146	10·8 (9·8 to 11·8)	22·0 (19·8 to 24·1)
Sierra Leone	40·5 (38·6 to 42·2)	46·4 (44·3 to 48·3)	49·3 (46·8 to 51·4)	49·9 (47·5 to 52·1)	50·4 (48·1 to 52·5)	50·7 (48·2 to 52·9)	200	10·2 (9·1 to 11·3)	25·2 (22·4 to 27·9)
Somalia	45·1 (42·8 to 47·1)	45·6 (43·6 to 47·5)	54·1 (51·6 to 56·2)	51·3 (48·8 to 53·5)	55·1 (52·5 to 57·6)	55·7 (53·0 to 58·0)	176	10·6 (9·4 to 11·8)	23·5 (20·7 to 26·2)
South Africa	58·1 (55·3 to 60·5)	50·2 (47·9 to 52·0)	55·1 (52·4 to 57·3)	52·8 (50·4 to 54·9)	55·8 (53·1 to 58·1)	56·6 (53·8 to 59·0)	169	-1·5 (-2·2 to -0·8)	-2·6 (-3·8 to -1·4)
South Sudan	43·2 (41·1 to 45·1)	48·3 (46·2 to 50·2)	47·9 (45·4 to 49·8)	47·8 (45·3 to 49·8)	48·2 (45·6 to 50·2)	48·4 (45·9 to 50·5)	203	5·3 (4·0 to 6·3)	12·2 (9·7 to 14·6)
São Tomé and Príncipe	55·3 (52·8 to 57·3)	58·1 (55·7 to 60·2)	58·5 (56·2 to 60·6)	58·2 (55·8 to 60·4)	60·0 (57·3 to 62·1)	61·0 (58·4 to 63·4)	138	5·7 (4·7 to 6·6)	10·3 (8·6 to 11·9)
Tanzania	47·3 (44·9 to 49·1)	54·0 (51·5 to 56·1)	58·1 (55·3 to 60·3)	56·4 (53·9 to 58·5)	59·3 (56·6 to 61·6)	59·9 (57·1 to 62·4)	148	12·6 (11·7 to 13·7)	26·7 (24·7 to 28·9)
The Gambia	52·6 (50·2 to 54·6)	56·8 (54·1 to 59·1)	55·7 (52·9 to 57·9)	54·8 (52·1 to 57·2)	57·0 (54·2 to 59·4)	57·9 (54·9 to 60·1)	161	5·3 (4·3 to 6·3)	10·1 (8·3 to 12·0)
Togo	48·1 (45·8 to 49·9)	51·4 (49·1 to 53·5)	54·7 (52·2 to 56·7)	54·4 (51·9 to 56·5)	55·1 (52·4 to 57·3)	55·6 (52·7 to 58·0)	178	7·5 (6·4 to 8·5)	15·6 (13·5 to 17·6)
Uganda	40·4 (38·2 to 42·1)	51·5 (49·1 to 53·5)	56·1 (53·6 to 58·2)	53·7 (51·3 to 55·6)	56·3 (53·6 to 58·5)	56·9 (54·2 to 59·1)	166	16·5 (15·5 to 17·6)	41·0 (38·4 to 43·5)
Zambia	42·4 (40·6 to 44·2)	47·9 (45·7 to 50·0)	51·2 (48·9 to 53·3)	48·8 (46·6 to 50·8)	52·5 (50·0 to 54·7)	53·1 (50·8 to 55·4)	191	10·7 (9·5 to 11·9)	25·2 (22·4 to 27·9)
Zimbabwe	56·0 (53·6 to 58·3)	48·8 (46·9 to 50·8)	54·6 (52·1 to 56·8)	51·3 (48·8 to 53·4)	55·6 (52·9 to 57·9)	56·8 (54·2 to 59·4)	167	0·9 (-0·1 to 1·7)	1·5 (-0·1 to 3·0)
**Low SDI**	**45·8 (43·7 to 47·6)**	**53·3 (50·9 to 55·4)**	**55·7 (53·0 to 57·8)**	**54·7 (52·1 to 56·7)**	**56·5 (53·8 to 58·7)**	**56·9 (54·2 to 59·2)**		**11·1 (10·5 to 11·7)**	**24·2 (23·1 to 25·5)**
**Low-middle SDI**	**51·0 (48·6 to 53·1)**	**57·4 (54·8 to 59·6)**	**58·9 (56·0 to 61·3)**	**57·9 (55·1 to 60·2)**	**59·6 (56·8 to 61·9)**	**59·9 (57·1 to 62·3)**		**8·9 (8·2 to 9·6)**	**17·4 (16·2 to 18·7)**
**Middle SDI**	**57·2 (54·9 to 59·3)**	**62·1 (59·7 to 64·2)**	**63·0 (60·4 to 65·3)**	**61·7 (59·2 to 63·9)**	**63·2 (60·6 to 65·5)**	**63·5 (60·8 to 65·8)**		**6·3 (5·7 to 6·8)**	**11·0 (10·2 to 11·8)**
**High-middle SDI**	**57·8 (55·5 to 59·9)**	**63·6 (60·9 to 65·9)**	**65·0 (62·0 to 67·4)**	**64·0 (61·1 to 66·4)**	**65·3 (62·4 to 67·9)**	**65·6 (62·6 to 68·3)**		**7·8 (7·0 to 8·5)**	**13·4 (12·3 to 14·3)**
**High SDI**	**62·6 (60·0 to 64·8)**	**66·6 (63·9 to 69·1)**	**67·7 (64·7 to 70·3)**	**67·2 (64·1 to 69·8)**	**67·6 (64·5 to 70·2)**	**67·9 (64·9 to 70·6)**		**5·4 (4·7 to 5·9)**	**8·6 (7·8 to 9·2)**

*United Nations convention recognises Taiwan as a province of China

Table S3: Proportional morbidity gap (MG), 1990 and 2023, and absolute change, 1990–2023, for all sexes combined, globally, by SDI quintile, and for GBD super-regions and countries and territories

Absolute change was calculated based on unrounded proportional MG values and then rounded to 1 decimal place, leading to some discrepancies between the absolute change values in this table and what would be calculated based on the rounded proportional MGs.

Location	Proportional MG, 1990 (%)	Proportional MG, 2023 (%)	Absolute change, 1990–2023 (in percentage points)
**Global**	**13.6**	**14.5**	**0.9**
**Central Europe, Eastern Europe, and Central Asia**	**13.4**	**14.0**	**0.5**
Albania	13.3	13.9	0.6
Armenia	13.1	14.4	1.3
Azerbaijan	12.9	13.5	0.6
Belarus	13.0	13.6	0.5
Bosnia and Herzegovina	13.1	14.4	1.2
Bulgaria	12.8	13.5	0.7
Croatia	13.0	14.4	1.4
Czechia	13.4	14.8	1.3
Estonia	13.2	14.4	1.2
Georgia	12.6	14.0	1.3
Hungary	13.6	14.0	0.4
Kazakhstan	13.3	13.7	0.4
Kyrgyzstan	13.1	13.4	0.3
Latvia	13.5	13.9	0.4
Lithuania	13.6	14.4	0.8
Moldova	13.0	13.8	0.8
Mongolia	12.3	13.3	1.0
Montenegro	12.6	13.9	1.3
North Macedonia	12.6	13.7	1.1
Poland	13.6	14.4	0.8
Romania	13.1	13.4	0.3
Russia	13.6	14.2	0.5
Serbia	12.1	13.1	1.0
Slovakia	13.1	14.0	0.9
Slovenia	13.4	15.1	1.7
Tajikistan	12.8	12.8	0.1
Turkmenistan	12.6	12.9	0.3
Ukraine	13.7	14.2	0.5
Uzbekistan	12.7	12.7	-0.1
**High income**	**13.9**	**15.7**	**1.7**
Andorra	13.2	14.7	1.4
Argentina	13.0	14.1	1.1

Australia	14.3	16.6	2.3
Austria	13.0	14.6	1.6
Belgium	13.1	15.0	1.9
Brunei	12.2	13.0	0.8
Canada	14.7	16.7	2.0
Chile	13.4	14.9	1.5
Cyprus	12.3	13.9	1.6
Denmark	13.0	14.3	1.3
Finland	13.1	14.6	1.5
France	13.3	15.2	1.8
Germany	13.3	14.7	1.4
Greece	13.0	14.7	1.7
Greenland	13.3	15.0	1.7
Iceland	13.1	14.9	1.8
Ireland	12.6	14.8	2.3
Israel	12.6	14.1	1.5
Italy	13.0	14.2	1.2
Japan	12.9	14.0	1.1
Luxembourg	12.9	14.5	1.6
Malta	12.3	14.2	1.9
Monaco	12.4	14.1	1.7
Netherlands	13.2	15.7	2.5
New Zealand	15.0	16.0	1.0
Norway	13.4	15.0	1.6
Portugal	13.1	14.8	1.6
San Marino	13.1	15.1	2.1
Singapore	11.8	13.2	1.4
South Korea	13.1	13.9	0.8
Spain	13.3	14.5	1.2
Sweden	12.5	14.9	2.5
Switzerland	13.7	15.2	1.5
UK	13.2	15.7	2.5
USA	15.8	17.8	2.0
Uruguay	13.0	14.1	1.2
**Latin America and Caribbean**	**14.9**	**15.7**	**0.8**
Antigua and Barbuda	13.7	14.9	1.2
Bahamas	13.4	14.2	0.9

Barbados	13.4	14.4	1.0
Belize	13.6	14.9	1.3
Bermuda	13.0	14.5	1.5
Bolivia	13.7	14.1	0.5
Brazil	14.9	16.2	1.3
Colombia	14.7	15.3	0.6
Costa Rica	14.6	15.8	1.2
Cuba	13.5	14.4	1.0
Dominica	13.9	14.7	0.8
Dominican Republic	13.8	14.4	0.6
Ecuador	13.5	14.5	0.9
El Salvador	14.7	14.5	-0.1
Grenada	13.7	15.0	1.3
Guatemala	15.1	16.0	0.9
Guyana	14.2	14.5	0.2
Haiti	14.0	14.3	0.3
Honduras	14.6	15.1	0.5
Jamaica	13.8	14.6	0.8
Mexico	16.3	16.6	0.3
Nicaragua	14.8	15.4	0.7
Panama	14.1	15.2	1.1
Paraguay	14.0	14.7	0.6
Peru	14.5	14.4	-0.2
Puerto Rico	14.3	15.7	1.3
Saint Kitts and Nevis	13.6	14.6	1.0
Saint Lucia	14.1	15.3	1.2
Saint Vincent and the Grenadines	14.0	14.8	0.9
Suriname	14.0	15.2	1.2
Trinidad and Tobago	13.9	15.0	1.0
Venezuela	14.2	14.6	0.4
Virgin Islands	12.9	14.5	1.6
**North Africa and Middle East**	**14.1**	**14.9**	**0.8**
Afghanistan	18.5	17.9	-0.7
Algeria	13.6	14.2	0.6
Bahrain	13.1	14.4	1.3
Egypt	13.7	13.9	0.2
Iran	14.6	15.9	1.3

Iraq	14.7	14.9	0.3
Jordan	13.2	14.5	1.3
Kuwait	13.2	15.3	2.1
Lebanon	15.9	16.2	0.3
Libya	13.5	14.9	1.3
Morocco	13.3	13.8	0.4
Oman	13.9	14.2	0.3
Palestine	14.2	14.9	0.6
Qatar	12.8	14.6	1.9
Saudi Arabia	13.9	14.8	1.0
Sudan	13.6	14.0	0.5
Syria	14.4	16.7	2.3
Tunisia	13.6	14.8	1.1
Türkiye	13.8	14.4	0.7
United Arab Emirates	12.8	14.3	1.4
Yemen	15.0	15.9	1.0
**South Asia**	**14.3**	**14.6**	**0.3**
Bangladesh	14.1	13.8	-0.3
Bhutan	13.0	13.5	0.5
India	14.3	14.7	0.4
Nepal	14.8	14.3	-0.5
Pakistan	13.9	14.9	1.0
**Southeast Asia, East Asia, and Oceania**	**12.3**	**13.1**	**0.8**
American Samoa	12.3	13.3	1.0
Cambodia	13.6	13.0	-0.6
China	12.1	13.2	1.1
Cook Islands	12.0	13.3	1.3
Federated States of Micronesia	11.4	13.0	1.6
Fiji	12.6	13.3	0.7
Guam	11.7	12.8	1.2
Indonesia	12.9	12.7	-0.2
Kiribati	13.9	14.3	0.5
Laos	11.6	11.2	-0.5
Malaysia	12.0	12.6	0.6
Maldives	12.3	11.6	-0.7
Marshall Islands	11.9	12.4	0.5
Mauritius	12.5	13.7	1.2

Myanmar	12.4	12.2	-0.2
Nauru	11.1	11.8	0.7
Niue	11.9	12.8	0.9
North Korea	11.9	12.8	0.8
Northern Mariana Islands	11.5	12.5	1.1
Palau	11.9	13.0	1.1
Papua New Guinea	12.4	12.6	0.2
Philippines	12.6	12.3	-0.3
Samoa	12.7	13.1	0.5
Seychelles	11.6	12.5	0.9
Solomon Islands	10.6	11.4	0.9
Sri Lanka	12.5	12.8	0.3
Taiwan*	12.2	14.2	2.0
Thailand	13.0	13.2	0.2
Timor-Leste	12.6	12.3	-0.3
Tokelau	12.6	13.4	0.8
Tonga	12.5	13.1	0.7
Tuvalu	12.1	12.0	-0.1
Vanuatu	12.2	12.6	0.4
Viet Nam	12.6	12.4	-0.2
**Sub-Saharan Africa**	**13.9**	**14.0**	**0.1**
Angola	14.2	14.7	0.5
Benin	14.0	13.7	-0.3
Botswana	13.9	14.6	0.7
Burkina Faso	14.3	14.0	-0.3
Burundi	12.6	13.6	1.0
Cabo Verde	13.5	13.7	0.2
Cameroon	14.0	13.4	-0.5
Central African Republic	15.4	15.8	0.5
Chad	13.1	13.4	0.3
Comoros	14.0	14.3	0.3
Congo (Brazzaville)	14.0	14.4	0.3
Côte d'Ivoire	14.8	13.8	-1.0
DR Congo	15.4	14.5	-1.0
Djibouti	12.6	13.0	0.4
Equatorial Guinea	14.3	13.6	-0.7
Eritrea	15.0	13.9	-1.1

Eswatini	12.0	14.2	2.2
Ethiopia	13.3	13.0	-0.2
Gabon	14.4	14.8	0.4
Ghana	13.6	13.2	-0.4
Guinea	13.3	13.5	0.2
Guinea-Bissau	13.8	13.7	-0.1
Kenya	12.9	13.5	0.6
Lesotho	12.4	14.0	1.6
Liberia	15.3	14.4	-0.8
Madagascar	12.4	12.2	-0.2
Malawi	12.6	12.9	0.4
Mali	14.5	14.6	0.2
Mauritania	13.1	13.2	0.0
Mozambique	15.1	15.1	0.0
Namibia	13.0	14.0	1.0
Niger	13.1	13.2	0.1
Nigeria	14.2	14.4	0.2
Rwanda	12.0	13.0	0.9
Senegal	14.0	14.0	0.0
Sierra Leone	13.8	13.8	0.1
Somalia	13.7	14.1	0.4
South Africa	14.3	15	0.7
South Sudan	13.8	14.0	0.2
São Tomé and Príncipe	13.0	13.2	0.3
Tanzania	14.1	13.7	-0.3
The Gambia	13.5	13.9	0.4
Togo	13.8	13.9	0.1
Uganda	14.0	13.3	-0.7
Zambia	13.1	13.6	0.5
Zimbabwe	13.6	14.2	0.7
**Low SDI**	14.1	14.2	0.0
**Low-middle SDI**	14.1	14.4	0.3
**Middle SDI**	13.2	13.5	0.3
**High-middle SDI**	13.1	14.3	1.2
**High SDI**	13.4	14.6	1.2

*United Nations convention recognises Taiwan as a province of China

Table S4: Male life expectancy, 1990, 2010, 2020, 2021, 2022, and 2023, and absolute and relative changes, 1990–2023, globally, by SDI quintile, and for GBD super-regions and countries and territories

Location	1990	2010	2020	2021	2022	2023	Rank (2023)	Absolute Change (1990-2023)	Relative Change, % (1990-2023)
**Global**	**62·2 (62·0 to 62·4)**	**68·4 (68·3 to 68·6)**	**70·4 (70·4 to 70·5)**	**69·3 (69·2 to 69·4)**	**71·0 (70·7 to 71·3)**	**71·5 (71·2 to 71·8)**		**9·3 (8·9 to 9·7)**	**15·0 (14·4 to 15·6)**
**Central Europe, Eastern Europe, and Central Asia**	**64·8 (64·7 to 64·8)**	**66·6 (66·5 to 66·6)**	**68·6 (68·6 to 68·6)**	**67·8 (67·8 to 67·9)**	**68·8 (68·2 to 69·2)**	**69·9 (69·5 to 70·1)**		**5·1 (4·7 to 5·3)**	**7·8 (7·2 to 8·2)**
Albania	69·2 (68·6 to 69·7)	75·9 (75·4 to 76·4)	75·0 (74·7 to 75·2)	74·5 (74·2 to 74·7)	76·2 (75·6 to 76·8)	77·3 (76·6 to 77·9)	40	8·1 (7·2 to 8·9)	11·7 (10·4 to 13·0)
Armenia	67·0 (66·5 to 67·5)	70·7 (70·1 to 71·1)	65·1 (64·5 to 65·6)	71·7 (71·4 to 72·0)	74·0 (73·3 to 74·7)	76·3 (75·5 to 77·1)	49	9·3 (8·3 to 10·2)	13·9 (12·4 to 15·3)
Azerbaijan	62·1 (61·5 to 62·6)	68·8 (68·4 to 69·2)	66·8 (66·6 to 67·1)	68·5 (68·3 to 68·8)	70·5 (69·9 to 71·1)	69·9 (69·2 to 70·6)	125	7·8 (6·9 to 8·7)	12·6 (11·1 to 14·1)
Belarus	66·0 (65·8 to 66·2)	64·7 (64·5 to 64·9)	69·1 (68·8 to 69·5)	68·9 (68·5 to 69·4)	68·9 (68·3 to 69·5)	69·5 (68·9 to 70·2)	130	3·5 (2·8 to 4·2)	5·3 (4·2 to 6·4)
Bosnia and Herzegovina	69·5 (69·2 to 69·8)	73·7 (73·5 to 74·0)	73·1 (72·9 to 73·2)	72·0 (71·9 to 72·1)	74·0 (73·7 to 74·4)	75·3 (74·6 to 76·0)	55	5·8 (5·1 to 6·6)	8·4 (7·3 to 9·5)
Bulgaria	68·0 (67·9 to 68·2)	70·6 (70·4 to 70·8)	70·7 (70·6 to 70·8)	68·9 (68·8 to 69·0)	71·1 (70·8 to 71·3)	72·5 (72·2 to 72·7)	95	4·4 (4·1 to 4·7)	6·5 (6·0 to 7·0)
Croatia	68·8 (68·5 to 68·9)	73·4 (73·2 to 73·5)	74·6 (74·5 to 74·8)	73·8 (73·7 to 74·0)	74·4 (74·1 to 74·6)	75·5 (75·2 to 75·8)	54	6·7 (6·3 to 7·1)	9·8 (9·2 to 10·3)
Czechia	67·7 (67·6 to 67·9)	74·5 (74·3 to 74·6)	75·3 (75·3 to 75·4)	74·2 (74·2 to 74·3)	76·2 (76·0 to 76·3)	76·9 (76·8 to 77·1)	43	9·2 (9·0 to 9·4)	13·6 (13·2 to 13·9)
Estonia	64·8 (64·6 to 65·1)	70·3 (70·1 to 70·6)	74·1 (73·9 to 74·2)	72·9 (72·8 to 73·1)	73·3 (72·9 to 73·6)	74·2 (73·8 to 74·6)	71	9·4 (8·9 to 9·9)	14·5 (13·7 to 15·2)
Georgia	65·1 (64·6 to 65·5)	67·0 (66·5 to 67·4)	68·7 (68·4 to 68·9)	67·2 (66·9 to 67·4)	69·0 (68·4 to 69·6)	70·1 (69·4 to 70·7)	118	5·0 (4·2 to 5·8)	7·7 (6·4 to 9·0)
Hungary	65·2 (65·0 to 65·3)	70·7 (70·6 to 70·8)	72·3 (72·2 to 72·3)	70·8 (70·7 to 70·9)	72·5 (72·3 to 72·7)	73·4 (73·2 to 73·6)	84	8·2 (8·0 to 8·5)	12·7 (12·3 to 13·1)
Kazakhstan	62·9 (62·6 to 63·3)	63·8 (63·5 to 64·2)	67·5 (67·4 to 67·7)	66·9 (66·8 to 67·0)	70·0 (69·6 to 70·4)	70·8 (70·3 to 71·2)	108	7·9 (7·2 to 8·4)	12·5 (11·5 to 13·4)
Kyrgyzstan	62·7 (62·1 to 63·2)	65·4 (64·9 to 65·8)	68·3 (68·0 to 68·5)	69·6 (69·3 to 69·8)	72·0 (71·3 to 72·6)	72·5 (71·8 to 73·2)	94	9·8 (9·0 to 10·7)	15·7 (14·3 to 17·2)
Latvia	64·7 (64·5 to 65·0)	68·1 (67·9 to 68·4)	71·0 (70·9 to 71·2)	68·9 (68·7 to 69·0)	69·9 (69·5 to 70·2)	70·7 (70·3 to 71·1)	110	6·0 (5·6 to 6·5)	9·3 (8·6 to 10·0)
Lithuania	65·9 (65·6 to 66·1)	67·2 (67·0 to 67·5)	70·2 (70·1 to 70·3)	69·6 (69·5 to 69·7)	70·9 (70·5 to 71·2)	71·9 (71·5 to 72·2)	101	6·0 (5·6 to 6·4)	9·1 (8·4 to 9·7)
Moldova	64·7 (64·2 to 65·2)	66·3 (65·9 to 66·8)	69·1 (68·9 to 69·4)	68·4 (68·1 to 68·6)	70·9 (70·2 to 71·5)	71·6 (70·9 to 72·3)	103	6·9 (6·0 to 7·8)	10·7 (9·3 to 12·1)
Mongolia	58·1 (57·4 to 58·7)	62·7 (62·1 to 63·3)	68·2 (67·8 to 68·5)	66·3 (66·0 to 66·7)	67·2 (66·4 to 68·0)	68·0 (67·1 to 68·9)	144	10·0 (8·8 to 11·1)	17·1 (15·1 to 19·3)
Montenegro	70·2 (70·0 to 70·5)	72·7 (72·4 to 72·9)	72·7 (72·5 to 72·9)	70·5 (70·3 to 70·6)	73·6 (73·3 to 74·0)	74·7 (74·3 to 75·1)	64	4·5 (4·0 to 4·9)	6·4 (5·7 to 7·0)
North Macedonia	69·0 (68·8 to 69·3)	73·0 (72·8 to 73·3)	72·3 (72·2 to 72·5)	71·2 (71·1 to 71·4)	73·9 (73·6 to 74·2)	75·2 (74·6 to 75·7)	58	6·2 (5·5 to 6·7)	8·9 (8·0 to 9·8)
Poland	66·8 (66·7 to 66·9)	72·3 (72·2 to 72·4)	72·7 (72·7 to 72·7)	71·9 (71·8 to 71·9)	73·7 (73·5 to 73·8)	75·0 (74·8 to 75·1)	60	8·2 (8·0 to 8·3)	12·2 (12·0 to 12·5)
Romania	66·9 (66·8 to 67·1)	70·2 (70·1 to 70·4)	70·5 (70·5 to 70·6)	69·4 (69·4 to 69·5)	71·5 (71·3 to 71·6)	72·5 (72·3 to 72·7)	93	5·6 (5·3 to 5·8)	8·3 (7·9 to 8·7)
Russia	64·0 (63·9 to 64·0)	63·5 (63·5 to 63·6)	67·2 (67·1 to 67·2)	66·3 (66·3 to 66·3)	67·6 (67·1 to 68·0)	67·6 (66·7 to 68·3)	148	3·7 (2·8 to 4·3)	5·7 (4·3 to 6·8)
Serbia	67·2 (66·9 to 67·5)	71·7 (71·4 to 71·9)	72·3 (72·2 to 72·4)	71·0 (70·9 to 71·1)	73·1 (72·8 to 73·4)	73·9 (73·0 to 74·7)	75	6·7 (5·7 to 7·6)	10·0 (8·5 to 11·3)
Slovakia	66·7 (66·6 to 66·9)	71·7 (71·5 to 71·9)	73·5 (73·4 to 73·6)	71·2 (71·1 to 71·3)	73·4 (73·2 to 73·6)	74·8 (74·6 to 75·0)	62	8·1 (7·8 to 8·4)	12·1 (11·6 to 12·6)
Slovenia	69·1 (68·9 to 69·4)	76·0 (75·8 to 76·1)	77·6 (77·5 to 77·7)	77·6 (77·5 to 77·7)	78·2 (77·9 to 78·5)	79·0 (78·7 to 79·2)	30	9·8 (9·5 to 10·1)	14·2 (13·7 to 14·7)
Tajikistan	63·6 (62·9 to 64·2)	69·9 (69·4 to 70·4)	70·6 (70·3 to 70·8)	69·6 (68·9 to 70·4)	70·2 (69·2 to 71·3)	70·6 (69·5 to 71·7)	111	7·1 (5·8 to 8·3)	11·1 (9·1 to 13·1)
Turkmenistan	62·5 (61·9 to 63·1)	66·0 (65·5 to 66·6)	65·9 (65·0 to 66·7)	65·6 (64·6 to 66·6)	67·5 (66·4 to 68·6)	68·2 (67·0 to 69·5)	140	5·8 (4·4 to 7·1)	9·2 (7·0 to 11·4)
Ukraine	65·7 (65·6 to 65·8)	65·4 (65·3 to 65·5)	67·5 (67·4 to 67·5)	66·3 (66·2 to 66·3)	61·6 (58·1 to 64·0)	66·6 (65·9 to 67·2)	155	0·9 (0·2 to 1·5)	1·3 (0·3 to 2·3)
Uzbekistan	65·2 (64·9 to 65·6)	67·5 (67·2 to 67·9)	68·6 (68·4 to 68·7)	69·1 (68·9 to 69·2)	69·6 (69·2 to 69·9)	70·0 (69·6 to 70·3)	123	4·8 (4·2 to 5·3)	7·3 (6·5 to 8·2)
**High income**	**72·7 (72·7 to 72·7)**	**77·6 (77·6 to 77·7)**	**77·8 (77·8 to 77·8)**	**77·5 (77·5 to 77·5)**	**77·9 (77·9 to 77·9)**	**78·5 (78·5 to 78·6)**		**5·9 (5·8 to 5·9)**	**8·1 (8·0 to 8·1)**
Andorra	76·5 (76·1 to 76·9)	80·3 (79·9 to 80·6)	80·4 (80·0 to 80·8)	81·1 (80·7 to 81·6)	81·1 (80·6 to 81·7)	81·8 (81·2 to 82·4)	5	5·2 (4·5 to 6·0)	6·9 (5·9 to 7·8)
Argentina	68·9 (68·8 to 69·0)	72·5 (72·4 to 72·6)	73·3 (73·3 to 73·4)	71·4 (71·4 to 71·4)	73·2 (73·1 to 73·3)	74·7 (74·3 to 75·0)	65	5·7 (5·3 to 6·1)	8·3 (7·7 to 8·9)
Australia	73·8 (73·7 to 73·9)	79·7 (79·6 to 79·8)	81·7 (81·7 to 81·8)	81·7 (81·7 to 81·7)	81·1 (81·0 to 81·2)	81·9 (81·8 to 82·0)	4	8·1 (8·0 to 8·3)	11·0 (10·8 to 11·2)
Austria	72·3 (72·2 to 72·4)	77·6 (77·5 to 77·7)	78·8 (78·8 to 78·9)	78·7 (78·6 to 78·7)	79·1 (78·9 to 79·2)	79·5 (79·4 to 79·7)	25	7·3 (7·1 to 7·4)	10·1 (9·8 to 10·3)
Belgium	72·6 (72·5 to 72·8)	77·3 (77·2 to 77·4)	78·0 (77·9 to 78·1)	79·1 (79·1 to 79·2)	79·4 (79·3 to 79·5)	80·0 (79·9 to 80·1)	21	7·3 (7·2 to 7·5)	10·1 (9·9 to 10·4)
Brunei	70·2 (69·9 to 70·6)	75·1 (74·8 to 75·4)	76·7 (76·4 to 77·1)	76·4 (76·0 to 76·8)	76·0 (75·5 to 76·5)	76·9 (76·2 to 77·5)	46	6·6 (5·8 to 7·4)	9·4 (8·3 to 10·5)
Canada	74·2 (74·1 to 74·2)	79·3 (79·2 to 79·3)	79·3 (79·3 to 79·3)	79·1 (79·1 to 79·1)	79·0 (78·9 to 79·0)	79·6 (79·4 to 79·9)	24	5·5 (5·2 to 5·8)	7·4 (7·0 to 7·8)
Chile	70·1 (69·9 to 70·3)	75·9 (75·7 to 76·1)	76·5 (76·5 to 76·6)	75·8 (75·8 to 75·9)	76·8 (76·7 to 77·0)	78·2 (78·0 to 78·4)	34	8·1 (7·9 to 8·4)	11·6 (11·2 to 11·9)
Cyprus	72·8 (71·9 to 73·6)	77·1 (76·4 to 77·8)	78·2 (77·7 to 78·7)	77·8 (77·3 to 78·4)	78·1 (77·3 to 78·9)	78·6 (77·7 to 79·5)	31	5·8 (4·6 to 7·0)	7·9 (6·2 to 9·7)
Denmark	72·2 (72·1 to 72·4)	77·2 (77·0 to 77·3)	79·5 (79·4 to 79·6)	79·5 (79·4 to 79·6)	79·2 (79·1 to 79·4)	79·5 (79·4 to 79·7)	26	7·3 (7·1 to 7·5)	10·1 (9·8 to 10·4)
Finland	71·1 (71·0 to 71·3)	76·9 (76·7 to 77·0)	79·1 (79·1 to 79·2)	79·2 (79·1 to 79·3)	78·6 (78·4 to 78·7)	79·2 (79·0 to 79·3)	27	8·0 (7·8 to 8·3)	11·3 (10·9 to 11·6)
France	73·1 (73·1 to 73·2)	78·2 (78·1 to 78·2)	79·4 (79·4 to 79·4)	79·8 (79·8 to 79·8)	79·4 (79·4 to 79·5)	79·7 (79·6 to 79·8)	23	6·6 (6·5 to 6·6)	9·0 (8·8 to 9·1)
Germany	72·1 (72·1 to 72·2)	77·8 (77·7 to 77·8)	78·6 (78·6 to 78·6)	78·2 (78·2 to 78·2)	78·2 (78·2 to 78·2)	78·5 (78·2 to 78·8)	32	6·4 (6·1 to 6·7)	8·8 (8·4 to 9·2)
Greece	74·7 (74·6 to 74·8)	77·8 (77·6 to 77·9)	78·7 (78·6 to 78·7)	77·6 (77·5 to 77·6)	77·6 (77·5 to 77·8)	78·3 (78·1 to 78·4)	33	3·5 (3·3 to 3·7)	4·7 (4·5 to 5·0)
Greenland	60·0 (59·6 to 60·4)	66·4 (66·0 to 66·7)	68·1 (67·7 to 68·5)	67·7 (67·3 to 68·2)	69·1 (68·6 to 69·7)	70·0 (69·3 to 70·7)	121	10·0 (9·2 to 10·8)	16·6 (15·2 to 18·0)
Iceland	75·5 (75·1 to 75·8)	79·9 (79·6 to 80·2)	81·2 (81·0 to 81·4)	81·4 (81·2 to 81·7)	80·7 (80·3 to 81·1)	80·8 (80·4 to 81·2)	13	5·3 (4·8 to 5·8)	7·1 (6·3 to 7·8)
Ireland	72·3 (72·1 to 72·5)	78·3 (78·2 to 78·5)	80·3 (80·2 to 80·4)	80·6 (80·5 to 80·6)	80·4 (80·3 to 80·6)	80·8 (80·6 to 81·0)	14	8·5 (8·2 to 8·8)	11·8 (11·4 to 12·1)
Israel	75·6 (75·4 to 75·8)	80·1 (79·9 to 80·3)	81·0 (80·9 to 81·0)	80·8 (80·8 to 80·9)	81·1 (80·9 to 81·3)	81·2 (81·0 to 81·3)	10	5·6 (5·3 to 5·9)	7·4 (7·0 to 7·8)
Italy	73·7 (73·6 to 73·7)	79·2 (79·2 to 79·3)	79·7 (79·6 to 79·7)	80·1 (80·1 to 80·1)	79·8 (79·7 to 79·9)	80·7 (80·7 to 80·8)	15	7·1 (7·0 to 7·1)	9·6 (9·4 to 9·7)
Japan	76·0 (76·0 to 76·1)	79·7 (79·7 to 79·8)	81·7 (81·7 to 81·7)	81·6 (81·6 to 81·6)	81·2 (81·2 to 81·2)	81·0 (81·0 to 81·1)	11	5·0 (5·0 to 5·1)	6·6 (6·5 to 6·7)

Luxembourg	71·7 (71·4 to 72·0)	78·4 (78·1 to 78·7)	80·3 (80·1 to 80·5)	80·6 (80·3 to 80·9)	81·0 (80·5 to 81·3)	81·2 (80·8 to 81·7)	9	9·5 (8·9 to 10·0)	13·2 (12·4 to 14·0)
Malta	74·1 (73·7 to 74·4)	78·7 (78·4 to 79·0)	80·1 (79·9 to 80·3)	80·6 (80·4 to 80·8)	80·5 (80·1 to 80·9)	81·4 (81·0 to 81·8)	7	7·3 (6·8 to 7·8)	9·9 (9·1 to 10·6)
Monaco	72·8 (72·0 to 73·5)	75·1 (74·3 to 75·8)	76·7 (76·0 to 77·5)	76·5 (75·7 to 77·3)	77·3 (76·2 to 78·3)	77·9 (76·8 to 79·0)	36	5·2 (3·9 to 6·4)	7·1 (5·3 to 8·9)
Netherlands	73·8 (73·7 to 73·9)	78·7 (78·6 to 78·8)	79·5 (79·5 to 79·5)	79·5 (79·5 to 79·6)	79·9 (79·8 to 80·1)	80·4 (80·3 to 80·5)	18	6·7 (6·5 to 6·8)	9·0 (8·8 to 9·2)
New Zealand	72·7 (72·5 to 72·8)	79·0 (78·8 to 79·2)	80·5 (80·4 to 80·6)	80·3 (80·3 to 80·4)	79·6 (79·4 to 79·8)	79·9 (79·7 to 80·1)	22	7·2 (7·0 to 7·5)	10·0 (9·6 to 10·3)
Norway	73·5 (73·4 to 73·6)	78·8 (78·6 to 78·9)	81·3 (81·3 to 81·4)	81·4 (81·4 to 81·5)	80·8 (80·7 to 81·0)	81·4 (81·2 to 81·5)	8	7·9 (7·7 to 8·1)	10·7 (10·4 to 11·0)
Portugal	70·7 (70·6 to 70·8)	76·9 (76·8 to 77·1)	78·5 (78·4 to 78·5)	78·4 (78·4 to 78·5)	78·3 (78·2 to 78·5)	79·0 (78·8 to 79·1)	29	8·3 (8·1 to 8·5)	11·7 (11·4 to 12·0)
San Marino	74·9 (74·1 to 75·6)	81·6 (80·8 to 82·3)	82·0 (81·3 to 82·7)	82·7 (81·9 to 83·5)	82·1 (81·1 to 83·0)	83·2 (82·1 to 84·2)	1	8·3 (7·0 to 9·5)	11·1 (9·3 to 12·8)
Singapore	72·9 (72·7 to 73·1)	80·4 (80·2 to 80·6)	83·5 (83·4 to 83·6)	83·2 (83·1 to 83·3)	82·6 (82·3 to 82·8)	83·1 (82·9 to 83·4)	2	10·2 (9·9 to 10·6)	14·0 (13·5 to 14·5)
South Korea	67·5 (67·2 to 67·8)	77·2 (77·0 to 77·5)	80·5 (80·5 to 80·6)	80·6 (80·5 to 80·6)	79·8 (79·6 to 80·0)	80·6 (80·4 to 80·8)	16	13·1 (12·7 to 13·5)	19·4 (18·8 to 20·0)
Spain	73·2 (73·2 to 73·3)	78·9 (78·9 to 79·0)	79·4 (79·3 to 79·4)	80·1 (80·0 to 80·1)	79·6 (79·5 to 79·7)	80·4 (80·3 to 80·5)	19	7·1 (7·0 to 7·3)	9·8 (9·6 to 9·9)
Sweden	74·9 (74·8 to 75·0)	79·6 (79·5 to 79·7)	80·6 (80·5 to 80·6)	81·2 (81·2 to 81·2)	81·4 (81·3 to 81·5)	81·6 (81·5 to 81·7)	6	6·7 (6·6 to 6·9)	9·0 (8·8 to 9·2)
Switzerland	74·2 (74·1 to 74·4)	80·1 (80·0 to 80·2)	81·1 (81·1 to 81·2)	81·7 (81·6 to 81·7)	81·7 (81·6 to 81·8)	82·6 (82·4 to 82·7)	3	8·3 (8·1 to 8·5)	11·2 (10·9 to 11·5)
UK	72·9 (72·8 to 72·9)	78·3 (78·3 to 78·4)	78·3 (78·3 to 78·3)	78·6 (78·6 to 78·6)	79·0 (79·0 to 79·1)	79·0 (79·0 to 79·1)	28	6·1 (6·1 to 6·2)	8·4 (8·3 to 8·5)
USA	72·0 (72·0 to 72·0)	76·3 (76·2 to 76·3)	74·4 (74·4 to 74·4)	73·6 (73·6 to 73·6)	74·9 (74·9 to 74·9)	75·9 (75·9 to 75·9)	50	3·9 (3·9 to 3·9)	5·4 (5·4 to 5·5)
Uruguay	69·5 (69·3 to 69·7)	72·7 (72·5 to 72·9)	74·3 (74·2 to 74·4)	71·4 (71·3 to 71·5)	72·5 (72·3 to 72·8)	74·1 (73·8 to 74·4)	72	4·5 (4·2 to 4·9)	6·5 (6·0 to 7·1)
**Latin America and Caribbean**	**67·1 (67·0 to 67·2)**	**71·0 (70·9 to 71·1)**	**69·9 (69·8 to 69·9)**	**68·7 (68·6 to 68·7)**	**72·3 (72·2 to 72·4)**	**73·0 (72·9 to 73·1)**		**6·0 (5·8 to 6·1)**	**8·9 (8·7 to 9·1)**
Antigua and Barbuda	70·5 (69·8 to 71·2)	74·9 (74·2 to 75·5)	74·9 (74·2 to 75·6)	74·1 (73·3 to 74·8)	75·3 (74·2 to 76·3)	75·8 (74·6 to 76·9)	53	5·3 (4·0 to 6·6)	7·5 (5·6 to 9·5)
Bahamas	67·2 (66·8 to 67·5)	69·3 (68·9 to 69·6)	67·9 (67·5 to 68·3)	64·0 (63·5 to 64·5)	68·5 (68·0 to 69·0)	69·8 (69·2 to 70·5)	126	2·7 (1·9 to 3·4)	4·0 (2·9 to 5·1)
Barbados	71·4 (71·0 to 71·8)	74·5 (74·1 to 74·8)	74·1 (73·6 to 74·5)	73·1 (72·6 to 73·7)	73·2 (72·6 to 73·8)	74·3 (73·7 to 75·0)	69	2·9 (2·2 to 3·7)	4·1 (3·1 to 5·2)
Belize	71·1 (70·6 to 71·5)	71·3 (70·8 to 71·7)	70·3 (69·9 to 70·7)	68·8 (68·2 to 69·2)	71·1 (70·5 to 71·8)	72·6 (71·8 to 73·3)	92	1·5 (0·7 to 2·5)	2·2 (0·9 to 3·5)
Bermuda	70·4 (70·1 to 70·8)	77·5 (77·2 to 77·8)	77·5 (77·1 to 77·8)	76·8 (76·4 to 77·1)	77·4 (76·9 to 77·8)	78·0 (77·5 to 78·5)	35	7·5 (7·0 to 8·2)	10·7 (9·9 to 11·6)
Bolivia	60·5 (60·1 to 60·9)	67·1 (66·7 to 67·4)	60·7 (60·3 to 61·1)	61·6 (61·1 to 62·0)	68·0 (67·5 to 68·5)	69·3 (68·7 to 70·0)	132	8·8 (8·1 to 9·6)	14·6 (13·3 to 15·9)
Brazil	66·1 (65·9 to 66·3)	71·3 (71·2 to 71·5)	71·6 (71·5 to 71·6)	69·8 (69·7 to 69·8)	72·7 (72·6 to 72·9)	73·5 (73·3 to 73·7)	83	7·4 (7·1 to 7·6)	11·2 (10·7 to 11·5)
Colombia	68·5 (68·3 to 68·7)	74·9 (74·7 to 75·1)	74·1 (74·0 to 74·1)	71·8 (71·7 to 71·8)	75·5 (75·3 to 75·7)	77·0 (76·8 to 77·2)	42	8·5 (8·2 to 8·8)	12·4 (11·9 to 12·9)
Costa Rica	74·5 (74·2 to 74·7)	77·2 (77·0 to 77·4)	76·7 (76·5 to 76·8)	75·1 (74·9 to 75·2)	76·4 (76·1 to 76·7)	76·9 (76·6 to 77·2)	45	2·4 (1·9 to 2·8)	3·2 (2·6 to 3·7)
Cuba	73·5 (73·1 to 73·9)	76·5 (76·2 to 76·8)	75·5 (75·3 to 75·6)	71·3 (71·1 to 71·4)	75·4 (74·3 to 76·4)	75·8 (74·7 to 77·0)	52	2·4 (1·1 to 3·6)	3·2 (1·5 to 4·9)
Dominica	71·9 (71·5 to 72·2)	73·0 (72·7 to 73·4)	72·9 (72·5 to 73·3)	71·9 (71·4 to 72·4)	73·3 (72·7 to 73·9)	73·8 (73·2 to 74·4)	77	1·9 (1·2 to 2·7)	2·7 (1·7 to 3·7)
Dominican Republic	68·4 (68·0 to 68·8)	72·1 (71·8 to 72·5)	68·5 (68·3 to 68·8)	69·0 (68·7 to 69·3)	69·2 (68·8 to 69·6)	69·9 (69·3 to 70·6)	124	1·5 (0·8 to 2·3)	2·2 (1·1 to 3·3)
Ecuador	68·7 (68·5 to 69·0)	73·2 (72·9 to 73·4)	70·4 (70·3 to 70·4)	71·7 (71·6 to 71·8)	73·7 (73·4 to 73·9)	73·6 (73·3 to 73·9)	81	4·8 (4·4 to 5·2)	7·1 (6·5 to 7·6)
El Salvador	63·9 (63·5 to 64·4)	69·0 (68·5 to 69·5)	66·4 (65·8 to 67·0)	65·8 (65·0 to 66·6)	68·5 (67·5 to 69·3)	69·0 (68·0 to 70·0)	135	5·1 (4·0 to 6·2)	7·9 (6·2 to 9·7)
Grenada	68·1 (67·4 to 68·8)	72·8 (72·1 to 73·5)	72·0 (71·2 to 72·7)	69·4 (68·6 to 70·2)	72·2 (71·1 to 73·3)	73·4 (72·1 to 74·7)	86	5·3 (3·8 to 6·7)	7·8 (5·6 to 9·9)
Guatemala	59·7 (59·1 to 60·3)	68·4 (68·0 to 68·8)	68·1 (67·9 to 68·3)	65·8 (65·6 to 66·0)	69·3 (68·7 to 69·8)	70·8 (69·6 to 72·0)	107	11·1 (9·7 to 12·4)	18·5 (16·1 to 20·8)
Guyana	61·9 (61·1 to 62·6)	64·7 (64·0 to 65·5)	66·6 (65·9 to 67·3)	63·6 (63·0 to 64·3)	65·2 (64·2 to 66·3)	65·8 (64·3 to 67·0)	160	3·9 (2·2 to 5·4)	6·3 (3·6 to 8·8)
Haiti	50·5 (49·6 to 51·4)	34·8 (33·1 to 36·4)	60·4 (59·6 to 61·3)	59·9 (58·9 to 60·7)	60·5 (59·3 to 61·5)	59·9 (58·7 to 61·2)	193	9·4 (8·0 to 10·9)	18·6 (15·6 to 21·9)
Honduras	68·1 (67·7 to 68·4)	72·5 (72·2 to 72·8)	70·4 (70·1 to 70·8)	68·7 (68·2 to 69·1)	73·8 (73·4 to 74·3)	74·4 (73·9 to 75·0)	68	6·4 (5·7 to 7·1)	9·3 (8·4 to 10·4)
Jamaica	73·4 (72·8 to 74·1)	75·5 (74·9 to 76·0)	71·5 (70·7 to 72·3)	70·0 (69·0 to 71·1)	72·0 (70·9 to 73·3)	73·5 (72·2 to 74·8)	82	0·1 (-1·3 to 1·4)	0·1 (-1·7 to 1·9)
Mexico	68·4 (68·2 to 68·5)	72·8 (72·7 to 72·9)	67·6 (67·5 to 67·6)	67·6 (67·5 to 67·6)	72·2 (72·1 to 72·3)	73·4 (73·3 to 73·5)	85	5·1 (4·8 to 5·2)	7·4 (7·1 to 7·7)
Nicaragua	68·0 (67·4 to 68·7)	72·2 (71·7 to 72·8)	68·6 (68·3 to 68·8)	68·5 (68·2 to 68·7)	72·6 (72·0 to 73·3)	74·1 (72·9 to 75·1)	73	6·0 (4·7 to 7·3)	8·9 (6·9 to 10·7)
Panama	73·8 (73·5 to 74·1)	75·2 (75·0 to 75·5)	73·6 (73·5 to 73·8)	74·2 (74·1 to 74·4)	76·1 (75·7 to 76·4)	76·6 (76·0 to 77·1)	48	2·8 (2·2 to 3·4)	3·8 (2·9 to 4·6)
Paraguay	73·3 (72·9 to 73·6)	71·1 (70·8 to 71·4)	69·2 (69·1 to 69·4)	64·2 (64·1 to 64·4)	69·4 (68·8 to 70·0)	70·0 (69·3 to 70·8)	119	-3·3 (-4·0 to -2·5)	-4·4 (-5·5 to -3·4)
Peru	71·0 (70·6 to 71·3)	77·3 (77·0 to 77·6)	69·3 (69·2 to 69·4)	68·3 (68·2 to 68·4)	76·0 (75·7 to 76·3)	75·3 (74·9 to 75·7)	56	4·3 (3·8 to 4·9)	6·1 (5·3 to 6·9)
Puerto Rico	70·3 (70·0 to 70·5)	75·5 (75·2 to 75·7)	76·7 (76·5 to 76·8)	76·1 (75·9 to 76·3)	76·1 (75·8 to 76·5)	77·3 (77·0 to 77·7)	38	7·0 (6·6 to 7·5)	10·0 (9·4 to 10·7)
Saint Kitts and Nevis	66·1 (65·7 to 66·5)	70·5 (70·2 to 70·9)	71·3 (71·0 to 71·7)	70·2 (69·8 to 70·7)	71·6 (71·1 to 72·1)	72·2 (71·6 to 72·8)	96	6·1 (5·4 to 6·8)	9·3 (8·2 to 10·4)
Saint Lucia	68·0 (67·3 to 68·8)	72·7 (72·0 to 73·4)	73·8 (73·0 to 74·5)	71·6 (70·6 to 72·6)	74·0 (72·8 to 75·1)	74·8 (73·5 to 76·0)	61	6·8 (5·2 to 8·3)	10·0 (7·6 to 12·3)
Saint Vincent and the Grenadines	69·3 (68·6 to 70·0)	71·7 (71·0 to 72·3)	71·7 (71·0 to 72·4)	71·6 (70·8 to 72·5)	72·5 (71·3 to 73·7)	72·7 (71·5 to 74·0)	91	3·4 (2·1 to 4·9)	5·0 (3·0 to 7·1)
Suriname	68·1 (67·8 to 68·4)	69·5 (69·2 to 69·7)	69·2 (68·9 to 69·4)	64·7 (64·4 to 65·0)	69·2 (68·9 to 69·6)	70·4 (69·9 to 70·9)	112	2·3 (1·7 to 2·9)	3·3 (2·5 to 4·2)
Trinidad and Tobago	66·8 (66·5 to 67·1)	70·3 (70·0 to 70·6)	69·7 (69·3 to 70·1)	66·3 (65·8 to 66·7)	68·5 (67·9 to 69·0)	70·3 (69·8 to 70·8)	115	3·5 (2·8 to 4·1)	5·2 (4·2 to 6·1)
Venezuela	68·8 (68·6 to 69·0)	70·6 (70·4 to 70·7)	67·8 (67·4 to 68·3)	67·0 (66·4 to 67·5)	68·0 (67·4 to 68·7)	68·2 (67·5 to 68·9)	141	-0·6 (-1·3 to 0·1)	-0·9 (-1·9 to 0·2)
Virgin Islands	68·7 (68·0 to 69·4)	71·3 (70·5 to 72·0)	71·8 (71·0 to 72·7)	69·9 (68·9 to 71·0)	71·2 (70·0 to 72·4)	71·7 (70·3 to 73·1)	102	3·0 (1·6 to 4·3)	4·3 (2·3 to 6·3)
**North Africa and Middle East**	**63·8 (63·6 to 63·9)**	**70·5 (70·4 to 70·7)**	**70·0 (69·9 to 70·1)**	**69·9 (69·8 to 70·0)**	**72·0 (71·8 to 72·3)**	**72·1 (71·8 to 72·5)**		**8·4 (8·0 to 8·8)**	**13·1 (12·5 to 13·8)**
Afghanistan	58·1 (57·2 to 58·9)	65·0 (64·2 to 65·6)	63·0 (62·2 to 63·7)	59·8 (58·9 to 60·7)	67·5 (66·4 to 68·6)	68·1 (67·0 to 69·3)	143	10·1 (8·6 to 11·5)	17·4 (14·7 to 20·0)
Algeria	67·0 (66·7 to 67·3)	73·4 (73·1 to 73·7)	73·4 (73·2 to 73·7)	73·7 (73·4 to 73·9)	74·3 (74·0 to 74·7)	74·5 (74·1 to 75·0)	67	7·5 (7·0 to 8·1)	11·2 (10·4 to 12·1)
Bahrain	69·0 (68·4 to 69·6)	74·1 (73·6 to 74·6)	75·1 (74·6 to 75·5)	74·5 (73·8 to 75·1)	75·7 (74·8 to 76·6)	75·9 (74·9 to 77·0)	51	6·9 (5·8 to 8·0)	10·0 (8·4 to 11·7)

Egypt	63·0 (62·7 to 63·2)	67·6 (67·4 to 67·8)	68·1 (68·0 to 68·2)	67·6 (67·4 to 67·7)	68·8 (67·8 to 69·7)	69·1 (68·0 to 70·2)	133	6·2 (5·0 to 7·3)	9·8 (8·0 to 11·5)
Iran	65·2 (64·9 to 65·5)	74·5 (74·3 to 74·8)	72·4 (72·1 to 72·7)	73·2 (72·9 to 73·5)	76·4 (76·0 to 76·8)	77·2 (76·7 to 77·7)	41	12·0 (11·4 to 12·6)	18·4 (17·4 to 19·4)
Iraq	63·6 (63·3 to 63·8)	65·5 (65·1 to 65·8)	64·4 (64·0 to 64·8)	65·8 (65·4 to 66·3)	68·0 (67·5 to 68·5)	68·3 (67·7 to 69·0)	139	4·8 (4·1 to 5·5)	7·5 (6·5 to 8·7)
Jordan	68·0 (67·7 to 68·4)	73·3 (73·1 to 73·5)	73·9 (73·7 to 74·1)	72·7 (72·5 to 72·9)	76·0 (75·7 to 76·3)	76·9 (76·4 to 77·4)	44	8·9 (8·3 to 9·5)	13·0 (12·1 to 14·0)
Kuwait	69·9 (69·5 to 70·4)	77·4 (77·2 to 77·7)	78·4 (78·1 to 78·6)	78·4 (78·2 to 78·7)	80·2 (79·8 to 80·5)	80·9 (80·4 to 81·4)	12	10·9 (10·3 to 11·6)	15·6 (14·7 to 16·7)
Lebanon	72·3 (71·5 to 73·2)	78·7 (78·1 to 79·2)	69·7 (69·4 to 70·0)	73·9 (73·6 to 74·3)	75·9 (74·9 to 77·0)	76·8 (75·6 to 78·1)	47	4·5 (3·1 to 6·0)	6·2 (4·2 to 8·4)
Libya	68·4 (68·1 to 68·7)	72·8 (72·6 to 73·1)	70·8 (70·4 to 71·2)	69·6 (69·1 to 70·0)	73·5 (73·0 to 73·9)	70·3 (69·7 to 70·9)	116	1·9 (1·2 to 2·6)	2·8 (1·7 to 3·8)
Morocco	65·0 (64·4 to 65·5)	71·2 (70·7 to 71·8)	71·8 (71·4 to 72·2)	72·0 (71·6 to 72·5)	73·0 (72·3 to 73·8)	73·3 (72·5 to 74·2)	88	8·4 (7·3 to 9·4)	12·9 (11·2 to 14·5)
Oman	66·2 (65·7 to 66·8)	71·5 (71·1 to 71·9)	71·5 (71·2 to 71·9)	70·8 (70·4 to 71·2)	74·5 (73·9 to 75·1)	75·2 (74·4 to 75·9)	59	8·9 (8·0 to 9·8)	13·5 (12·0 to 14·9)
Palestine	68·3 (68·0 to 68·7)	72·6 (72·3 to 72·8)	74·3 (74·1 to 74·5)	72·8 (72·6 to 73·0)	74·9 (74·6 to 75·2)	60·7 (59·4 to 62·0)	186	-7·6 (-9·0 to -6·4)	-11·1 (-13·1 to -9·4)
Qatar	71·4 (70·8 to 72·0)	77·2 (76·7 to 77·8)	79·7 (79·3 to 80·2)	80·0 (79·5 to 80·5)	80·5 (79·7 to 81·3)	80·6 (79·6 to 81·6)	17	9·2 (8·1 to 10·4)	13·0 (11·3 to 14·7)
Saudi Arabia	66·3 (65·8 to 66·9)	69·7 (69·4 to 70·1)	71·6 (71·3 to 71·9)	72·9 (72·8 to 73·1)	73·6 (73·2 to 74·0)	73·8 (73·0 to 74·6)	78	7·5 (6·5 to 8·5)	11·3 (9·7 to 12·9)
Sudan	54·6 (53·8 to 55·4)	64·3 (63·5 to 65·0)	66·6 (65·8 to 67·3)	67·2 (66·3 to 68·2)	68·3 (67·3 to 69·3)	67·3 (66·2 to 68·5)	153	12·7 (11·3 to 14·1)	23·4 (20·5 to 26·0)
Syria	67·3 (67·0 to 67·6)	73·2 (73·0 to 73·4)	72·3 (71·9 to 72·7)	72·5 (72·1 to 72·9)	74·4 (74·0 to 74·9)	73·9 (73·4 to 74·4)	74	6·6 (6·0 to 7·2)	9·9 (9·0 to 10·8)
Tunisia	66·8 (66·5 to 67·1)	71·2 (71·0 to 71·5)	70·3 (70·2 to 70·4)	68·2 (68·0 to 68·3)	72·7 (72·5 to 72·9)	73·4 (72·9 to 73·8)	87	6·6 (6·1 to 7·1)	9·9 (9·1 to 10·6)
Türkiye	65·2 (64·9 to 65·6)	74·6 (74·3 to 74·8)	73·4 (73·3 to 73·5)	72·8 (72·7 to 72·8)	74·3 (74·0 to 74·4)	73·9 (72·9 to 74·9)	76	8·7 (7·6 to 9·8)	13·3 (11·7 to 15·0)
United Arab Emirates	69·5 (69·0 to 70·0)	76·2 (75·8 to 76·6)	79·9 (79·5 to 80·3)	79·8 (79·3 to 80·2)	80·4 (79·8 to 80·9)	80·2 (79·5 to 80·9)	20	10·7 (9·8 to 11·5)	15·4 (14·1 to 16·6)
Yemen	57·3 (56·5 to 58·2)	66·9 (66·2 to 67·6)	64·2 (63·5 to 65·0)	65·2 (64·4 to 66·1)	68·5 (67·4 to 69·5)	69·4 (68·4 to 70·6)	131	12·1 (10·7 to 13·5)	21·2 (18·5 to 23·7)
**South Asia**	**58·3 (57·7 to 58·9)**	**65·8 (65·4 to 66·2)**	**68·3 (68·0 to 68·5)**	**66·4 (66·2 to 66·7)**	**69·7 (68·9 to 70·5)**	**70·1 (69·1 to 71·0)**		**11·8 (10·6 to 12·9)**	**20·2 (18·1 to 22·2)**
Bangladesh	57·5 (56·7 to 58·3)	67·4 (66·7 to 68·0)	68·8 (68·0 to 69·5)	67·6 (66·7 to 68·6)	70·7 (69·7 to 71·9)	71·0 (69·9 to 72·2)	105	13·5 (12·2 to 14·9)	23·5 (21·0 to 26·1)
Bhutan	60·0 (59·3 to 60·8)	69·4 (68·6 to 70·1)	72·4 (71·6 to 73·2)	72·7 (71·8 to 73·6)	72·7 (71·6 to 73·8)	72·9 (71·7 to 74·1)	89	12·8 (11·5 to 14·3)	21·4 (18·9 to 24·0)
India	58·1 (57·4 to 58·8)	65·8 (65·3 to 66·2)	68·6 (68·4 to 68·9)	66·6 (66·4 to 66·8)	69·9 (68·9 to 70·9)	70·2 (69·1 to 71·3)	117	12·1 (10·7 to 13·5)	20·9 (18·3 to 23·4)
Nepal	57·9 (57·1 to 58·8)	68·5 (68·0 to 69·1)	70·0 (69·2 to 70·8)	67·5 (66·6 to 68·4)	71·7 (70·6 to 72·8)	71·9 (70·7 to 73·1)	99	14·0 (12·5 to 15·5)	24·2 (21·4 to 27·1)
Pakistan	61·7 (60·9 to 62·4)	65·9 (65·1 to 66·6)	66·4 (65·5 to 67·3)	66·2 (65·1 to 67·2)	69·6 (68·3 to 70·8)	70·0 (68·7 to 71·2)	122	8·3 (6·8 to 9·8)	13·4 (11·1 to 16·1)
**Southeast Asia, East Asia, and Oceania**	**63·9 (63·4 to 64·4)**	**71·7 (71·3 to 72·0)**	**75·3 (75·1 to 75·4)**	**74·1 (73·8 to 74·3)**	**74·4 (73·7 to 75·2)**	**74·8 (74·1 to 75·6)**		**11·0 (10·1 to 11·9)**	**17·2 (15·7 to 18·7)**
American Samoa	66·3 (65·6 to 67·0)	67·8 (67·1 to 68·5)	67·1 (66·4 to 67·8)	64·5 (63·5 to 65·4)	67·2 (66·1 to 68·3)	68·0 (66·8 to 69·2)	145	1·7 (0·3 to 3·0)	2·6 (0·5 to 4·6)
Cambodia	55·2 (54·2 to 56·1)	67·0 (66·3 to 67·8)	70·9 (70·2 to 71·8)	70·3 (69·4 to 71·3)	71·0 (69·9 to 72·2)	70·8 (69·5 to 72·0)	109	15·6 (14·1 to 17·1)	28·2 (25·2 to 31·5)
China	64·3 (63·7 to 64·9)	73·5 (73·0 to 73·9)	78·2 (78·1 to 78·3)	77·7 (77·3 to 78·0)	77·2 (76·3 to 78·2)	77·6 (76·6 to 78·7)	37	13·4 (12·0 to 14·6)	20·8 (18·6 to 22·9)
Cook Islands	65·6 (65·2 to 65·9)	69·2 (68·9 to 69·6)	69·6 (69·1 to 70·0)	68·0 (67·5 to 68·5)	69·5 (69·0 to 70·0)	69·7 (69·0 to 70·4)	127	4·1 (3·4 to 4·9)	6·3 (5·1 to 7·5)
Federated States of Micronesia	57·9 (57·6 to 58·2)	61·8 (61·5 to 62·2)	62·9 (62·5 to 63·2)	61·3 (60·9 to 61·7)	63·1 (62·7 to 63·5)	63·7 (63·0 to 64·2)	164	5·7 (5·1 to 6·4)	9·9 (8·8 to 11·1)
Fiji	64·7 (64·4 to 65·0)	65·5 (65·2 to 65·8)	66·3 (66·1 to 66·6)	64·7 (64·4 to 64·9)	66·0 (65·6 to 66·3)	66·0 (65·4 to 66·5)	158	1·3 (0·7 to 1·9)	2·0 (1·0 to 3·0)
Guam	71·3 (70·9 to 71·6)	73·1 (72·7 to 73·4)	72·2 (71·8 to 72·6)	70·9 (70·5 to 71·3)	71·4 (70·8 to 71·9)	71·9 (71·3 to 72·5)	100	0·7 (-0·1 to 1·3)	0·9 (-0·1 to 1·9)
Indonesia	62·5 (61·6 to 63·3)	67·3 (66·5 to 68·1)	68·6 (67·8 to 69·5)	65·8 (64·8 to 66·9)	69·9 (68·7 to 71·1)	70·0 (68·7 to 71·3)	120	7·5 (6·1 to 9·0)	12·1 (9·7 to 14·4)
Kiribati	61·0 (60·6 to 61·3)	65·1 (64·8 to 65·5)	66·9 (66·4 to 67·3)	65·3 (64·9 to 65·8)	67·2 (66·6 to 67·7)	67·6 (66·8 to 68·3)	149	6·6 (5·8 to 7·4)	10·8 (9·5 to 12·3)
Laos	48·9 (47·9 to 50·1)	62·2 (61·4 to 63·0)	65·1 (64·3 to 66·0)	65·2 (64·2 to 66·2)	65·0 (63·7 to 66·2)	65·2 (63·9 to 66·5)	162	16·2 (14·5 to 18·0)	33·2 (29·3 to 37·3)
Malaysia	69·3 (68·9 to 69·7)	72·8 (72·1 to 73·5)	74·6 (74·4 to 74·8)	71·3 (71·1 to 71·5)	73·0 (72·6 to 73·4)	74·6 (74·2 to 75·1)	66	5·3 (4·7 to 5·9)	7·6 (6·7 to 8·5)
Maldives	65·0 (64·7 to 65·4)	73·9 (73·6 to 74·2)	75·3 (74·9 to 75·6)	74·4 (74·0 to 74·7)	74·9 (74·5 to 75·3)	75·2 (74·7 to 75·7)	57	10·2 (9·6 to 10·8)	15·7 (14·7 to 16·7)
Marshall Islands	58·7 (58·4 to 59·1)	61·4 (61·1 to 61·7)	62·5 (62·1 to 62·9)	61·1 (60·6 to 61·5)	62·8 (62·2 to 63·2)	63·1 (62·4 to 63·7)	168	4·4 (3·6 to 5·1)	7·4 (6·2 to 8·6)
Mauritius	66·1 (65·7 to 66·4)	70·6 (70·3 to 70·9)	71·2 (71·0 to 71·4)	69·4 (69·2 to 69·7)	70·0 (69·6 to 70·5)	70·3 (69·8 to 70·8)	114	4·3 (3·7 to 4·9)	6·4 (5·5 to 7·4)
Myanmar	51·0 (50·1 to 51·9)	59·5 (58·8 to 60·4)	61·9 (61·1 to 62·8)	60·0 (58·9 to 61·0)	61·6 (60·0 to 62·9)	61·6 (60·1 to 63·0)	182	10·5 (8·9 to 12·2)	20·7 (17·1 to 24·1)
Nauru	55·4 (55·0 to 55·8)	55·4 (55·0 to 55·8)	56·6 (56·1 to 57·0)	54·3 (53·8 to 54·8)	56·8 (56·3 to 57·4)	57·2 (56·5 to 57·9)	200	1·8 (1·0 to 2·6)	3·3 (1·9 to 4·7)
Niue	65·9 (65·6 to 66·2)	68·7 (68·4 to 69·1)	69·5 (69·1 to 69·9)	67·6 (67·1 to 68·0)	69·2 (68·6 to 69·7)	69·5 (68·8 to 70·2)	129	3·6 (2·9 to 4·4)	5·5 (4·4 to 6·7)
North Korea	67·7 (67·0 to 68·4)	69·7 (69·0 to 70·4)	71·0 (70·2 to 71·8)	71·3 (70·3 to 72·2)	71·2 (70·2 to 72·3)	71·3 (70·2 to 72·5)	104	3·6 (2·3 to 4·9)	5·3 (3·4 to 7·3)
Northern Mariana Islands	66·0 (65·7 to 66·3)	67·9 (67·6 to 68·3)	68·0 (67·7 to 68·4)	66·8 (66·4 to 67·2)	68·4 (68·0 to 68·9)	69·0 (68·5 to 69·7)	134	3·0 (2·4 to 3·7)	4·6 (3·6 to 5·6)
Palau	64·0 (63·6 to 64·3)	66·5 (66·2 to 66·8)	67·1 (66·7 to 67·5)	65·3 (64·9 to 65·8)	67·3 (66·8 to 67·8)	67·7 (67·2 to 68·4)	147	3·8 (3·1 to 4·5)	5·9 (4·8 to 7·1)
Papua New Guinea	60·5 (59·8 to 61·2)	63·6 (62·9 to 64·3)	65·9 (65·0 to 66·7)	65·1 (64·1 to 66·1)	65·6 (64·5 to 66·8)	65·8 (64·6 to 67·1)	159	5·3 (3·9 to 6·8)	8·8 (6·4 to 11·4)
Philippines	65·6 (65·4 to 65·9)	67·4 (67·2 to 67·6)	68·5 (68·4 to 68·5)	64·8 (64·8 to 64·9)	67·6 (67·4 to 67·8)	68·9 (68·6 to 69·2)	136	3·3 (2·9 to 3·7)	5·0 (4·4 to 5·6)
Samoa	68·6 (68·2 to 69·0)	70·5 (70·2 to 70·9)	70·7 (70·3 to 71·2)	69·2 (68·7 to 69·7)	70·8 (70·2 to 71·3)	70·9 (70·2 to 71·6)	106	2·3 (1·4 to 3·1)	3·3 (2·1 to 4·5)
Seychelles	66·1 (65·7 to 66·4)	69·3 (69·0 to 69·7)	72·7 (72·4 to 73·0)	70·1 (69·7 to 70·4)	72·5 (72·1 to 73·0)	72·2 (71·7 to 72·7)	97	6·1 (5·5 to 6·7)	9·2 (8·3 to 10·1)
Solomon Islands	59·1 (58·8 to 59·5)	62·0 (61·6 to 62·3)	61·6 (61·2 to 62·1)	61·1 (60·6 to 61·5)	61·7 (61·2 to 62·2)	61·9 (61·3 to 62·6)	179	2·8 (2·1 to 3·6)	4·8 (3·6 to 6·0)
Sri Lanka	65·5 (65·2 to 65·9)	70·7 (70·4 to 70·9)	75·4 (75·3 to 75·6)	73·6 (73·5 to 73·7)	73·5 (73·3 to 73·8)	73·6 (73·3 to 73·9)	80	8·1 (7·6 to 8·5)	12·3 (11·6 to 13·0)
Taiwan*	71·9 (71·7 to 72·0)	76·4 (76·3 to 76·5)	78·1 (78·0 to 78·1)	77·8 (77·7 to 77·9)	76·8 (76·7 to 76·9)	77·3 (77·2 to 77·5)	39	5·4 (5·2 to 5·7)	7·5 (7·2 to 7·9)
Thailand	68·6 (68·4 to 68·8)	72·0 (71·8 to 72·1)	74·0 (73·9 to 74·1)	72·8 (72·7 to 72·9)	72·6 (72·4 to 72·8)	73·7 (73·4 to 73·9)	79	5·1 (4·8 to 5·4)	7·4 (7·0 to 7·9)
Timor-Leste	56·8 (56·0 to 57·7)	67·7 (67·0 to 68·4)	68·8 (67·9 to 69·7)	68·7 (67·7 to 69·7)	69·4 (68·2 to 70·5)	69·6 (68·4 to 70·9)	128	12·8 (11·2 to 14·4)	22·5 (19·6 to 25·6)

Tokelau	70·1 (69·8 to 70·4)	73·4 (73·0 to 73·7)	75·0 (74·6 to 75·4)	73·0 (72·6 to 73·5)	74·2 (73·7 to 74·7)	74·7 (74·1 to 75·4)	63	4·7 (3·9 to 5·4)	6·6 (5·5 to 7·7)
Tonga	68·0 (67·7 to 68·3)	69·8 (69·5 to 70·1)	70·2 (69·8 to 70·6)	68·3 (67·9 to 68·8)	70·0 (69·5 to 70·5)	70·4 (69·7 to 71·0)	113	2·4 (1·7 to 3·1)	3·6 (2·5 to 4·6)
Tuvalu	56·2 (55·9 to 56·6)	60·0 (59·7 to 60·4)	60·3 (59·8 to 60·7)	58·5 (58·0 to 59·0)	60·3 (59·7 to 60·8)	60·4 (59·7 to 61·1)	190	4·1 (3·4 to 4·9)	7·4 (6·1 to 8·8)
Vanuatu	62·9 (62·5 to 63·3)	66·2 (65·8 to 66·6)	66·9 (66·5 to 67·4)	66·0 (65·5 to 66·5)	67·1 (66·5 to 67·7)	67·4 (66·6 to 68·2)	151	4·6 (3·6 to 5·5)	7·2 (5·8 to 8·7)
Viet Nam	64·9 (64·3 to 65·4)	69·7 (69·0 to 70·5)	71·6 (70·8 to 72·4)	70·8 (69·9 to 71·7)	71·5 (70·3 to 72·6)	71·9 (70·6 to 73·2)	98	7·0 (5·6 to 8·5)	10·9 (8·6 to 13·1)
**Sub-Saharan Africa**	**50·0 (49·7 to 50·2)**	**57·3 (57·1 to 57·5)**	**60·3 (60·1 to 60·5)**	**59·0 (58·8 to 59·2)**	**61·6 (61·4 to 61·9)**	**62·2 (62·0 to 62·5)**		**12·3 (11·9 to 12·6)**	**24·6 (23·7 to 25·4)**
Angola	46·4 (45·5 to 47·4)	56·4 (55·6 to 57·1)	59·0 (58·2 to 59·7)	57·9 (57·0 to 58·8)	60·4 (59·4 to 61·5)	60·7 (59·5 to 62·0)	187	14·3 (12·8 to 15·8)	30·9 (27·2 to 34·5)
Benin	51·3 (50·4 to 52·1)	59·4 (58·6 to 60·1)	62·2 (61·4 to 62·9)	62·5 (61·6 to 63·4)	63·1 (62·1 to 64·2)	63·4 (62·3 to 64·6)	165	12·2 (10·8 to 13·6)	23·7 (20·8 to 26·8)
Botswana	61·0 (60·7 to 61·4)	63·1 (62·8 to 63·4)	66·2 (65·9 to 66·5)	63·7 (63·4 to 64·1)	66·6 (66·2 to 67·1)	67·4 (66·7 to 67·9)	152	6·3 (5·6 to 7·0)	10·4 (9·2 to 11·5)
Burkina Faso	48·3 (47·3 to 49·3)	60·9 (60·1 to 61·6)	64·7 (64·0 to 65·5)	64·6 (63·7 to 65·4)	65·5 (64·5 to 66·5)	65·4 (64·3 to 66·5)	161	17·1 (15·6 to 18·5)	35·4 (31·8 to 39·0)
Burundi	45·5 (44·5 to 46·5)	56·6 (55·8 to 57·4)	58·8 (58·0 to 59·6)	57·0 (56·0 to 57·9)	59·4 (58·4 to 60·5)	59·8 (58·6 to 61·0)	194	14·3 (12·7 to 15·8)	31·4 (27·5 to 35·2)
Cabo Verde	66·4 (65·8 to 67·1)	71·2 (70·5 to 71·8)	69·6 (68·9 to 70·3)	68·7 (67·8 to 69·5)	71·7 (70·8 to 72·7)	72·8 (71·8 to 73·9)	90	6·4 (5·2 to 7·7)	9·6 (7·7 to 11·6)
Cameroon	53·3 (52·4 to 54·1)	55·0 (54·2 to 55·8)	59·1 (58·3 to 59·8)	57·9 (57·0 to 58·7)	60·6 (59·6 to 61·6)	61·1 (59·9 to 62·2)	185	7·8 (6·3 to 9·2)	14·6 (11·7 to 17·4)
Central African Republic	46·9 (46·0 to 47·8)	50·6 (49·7 to 51·4)	52·9 (52·1 to 53·7)	52·9 (52·0 to 53·8)	53·7 (52·6 to 54·7)	54·3 (53·2 to 55·4)	203	7·4 (6·1 to 8·9)	15·8 (12·8 to 19·1)
Chad	46·7 (45·8 to 47·6)	52·9 (52·0 to 53·7)	54·9 (54·2 to 55·6)	56·3 (55·5 to 57·2)	56·3 (55·3 to 57·4)	56·7 (55·6 to 57·8)	201	10·0 (8·6 to 11·4)	21·4 (18·1 to 24·8)
Comoros	64·4 (63·6 to 65·2)	72·9 (72·2 to 73·6)	71·8 (71·1 to 72·5)	68·6 (67·7 to 69·4)	73·1 (72·1 to 74·1)	74·3 (73·1 to 75·5)	70	9·9 (8·5 to 11·3)	15·4 (13·2 to 17·7)
Congo (Brazzaville)	53·7 (52·9 to 54·6)	61·0 (60·3 to 61·6)	61·6 (60·9 to 62·4)	60·9 (59·9 to 61·8)	62·7 (61·6 to 63·8)	62·9 (61·7 to 64·1)	173	9·2 (7·7 to 10·6)	17·2 (14·3 to 20·0)
Côte d'Ivoire	53·0 (52·2 to 53·9)	58·5 (57·7 to 59·3)	61·3 (60·5 to 62·0)	61·3 (60·4 to 62·1)	62·1 (61·1 to 63·1)	62·6 (61·4 to 63·7)	176	9·6 (8·2 to 10·9)	18·0 (15·2 to 20·8)
DR Congo	49·9 (49·1 to 50·8)	55·6 (54·8 to 56·4)	57·7 (57·0 to 58·5)	57·9 (57·1 to 58·8)	59·1 (58·1 to 60·1)	60·0 (58·9 to 61·1)	192	10·0 (8·6 to 11·3)	20·1 (17·1 to 23·0)
Djibouti	59·4 (58·7 to 60·3)	63·5 (62·7 to 64·3)	65·5 (64·7 to 66·3)	63·5 (62·6 to 64·4)	66·5 (65·4 to 67·6)	67·2 (66·0 to 68·5)	154	7·8 (6·3 to 9·3)	13·1 (10·6 to 15·6)
Equatorial Guinea	45·9 (45·1 to 46·8)	56·8 (56·1 to 57·5)	56·8 (56·0 to 57·6)	56·8 (55·9 to 57·7)	58·2 (57·1 to 59·4)	58·6 (57·2 to 59·8)	197	12·6 (11·2 to 14·1)	27·6 (24·1 to 31·0)
Eritrea	40·5 (39·0 to 41·9)	58·3 (57·4 to 59·1)	60·2 (59·4 to 61·1)	60·2 (59·1 to 61·2)	59·1 (57·9 to 60·3)	61·3 (60·0 to 62·5)	183	20·8 (18·9 to 22·8)	51·5 (45·5 to 58·2)
Eswatini	52·5 (51·6 to 53·3)	45·0 (44·0 to 46·0)	55·7 (54·8 to 56·6)	54·5 (53·5 to 55·5)	57·7 (56·4 to 58·9)	58·8 (57·4 to 60·2)	196	6·3 (4·7 to 8·0)	12·0 (8·9 to 15·4)
Ethiopia	41·5 (40·6 to 42·3)	58·9 (58·2 to 59·6)	61·4 (60·7 to 62·2)	59·4 (58·5 to 60·3)	62·5 (61·4 to 63·5)	63·0 (61·8 to 64·1)	171	21·5 (20·0 to 23·0)	51·9 (47·4 to 56·3)
Gabon	59·7 (58·8 to 60·4)	63·9 (63·2 to 64·6)	64·8 (64·0 to 65·6)	64·7 (63·8 to 65·6)	66·0 (64·9 to 67·1)	66·4 (65·2 to 67·7)	156	6·7 (5·3 to 8·3)	11·3 (8·8 to 13·9)
Ghana	54·2 (53·4 to 54·9)	59·8 (59·1 to 60·5)	61·5 (60·7 to 62·2)	60·9 (60·0 to 61·8)	62·4 (61·4 to 63·4)	62·9 (61·7 to 64·0)	172	8·8 (7·3 to 10·2)	16·2 (13·4 to 19·0)
Guinea	47·9 (46·9 to 48·9)	57·6 (56·9 to 58·4)	60·1 (59·4 to 60·8)	60·2 (59·3 to 61·0)	61·1 (60·0 to 62·2)	61·8 (60·6 to 62·9)	181	13·9 (12·4 to 15·4)	29·0 (25·5 to 32·7)
Guinea-Bissau	48·8 (47·8 to 49·8)	56·7 (55·9 to 57·4)	58·2 (57·4 to 59·0)	58·8 (58·0 to 59·7)	59·8 (58·8 to 60·9)	60·5 (59·4 to 61·6)	188	11·8 (10·3 to 13·3)	24·2 (20·8 to 27·7)
Kenya	60·2 (59·4 to 60·9)	62·6 (61·9 to 63·4)	64·1 (63·3 to 64·8)	62·7 (61·9 to 63·5)	67·5 (66·4 to 68·6)	68·2 (66·9 to 69·4)	142	8·0 (6·6 to 9·4)	13·3 (10·9 to 15·7)
Lesotho	55·1 (54·4 to 55·9)	50·1 (49·3 to 51·0)	54·0 (53·1 to 54·9)	53·1 (52·1 to 54·1)	54·7 (53·4 to 55·9)	55·3 (53·9 to 56·7)	202	0·2 (-1·4 to 1·6)	0·3 (-2·6 to 3·0)
Liberia	43·4 (42·4 to 44·4)	56·8 (56·0 to 57·5)	56·4 (55·6 to 57·2)	54·5 (53·7 to 55·4)	59·7 (58·7 to 60·7)	60·4 (59·3 to 61·5)	189	17·0 (15·4 to 18·5)	39·1 (34·8 to 43·4)
Madagascar	50·5 (49·7 to 51·4)	59·3 (58·6 to 59·9)	59·8 (59·0 to 60·5)	58·6 (57·8 to 59·5)	60·3 (59·3 to 61·3)	61·2 (60·1 to 62·2)	184	10·6 (9·3 to 12·0)	21·0 (18·1 to 23·9)
Malawi	43·9 (42·9 to 44·8)	55·0 (54·2 to 55·9)	58·4 (57·6 to 59·2)	52·6 (51·8 to 53·5)	58·6 (57·5 to 59·7)	59·7 (58·5 to 60·8)	195	15·8 (14·3 to 17·3)	36·0 (32·1 to 40·1)
Mali	47·7 (46·6 to 48·8)	59·1 (58·3 to 59·9)	59·7 (59·0 to 60·5)	59·3 (58·5 to 60·2)	61·4 (60·4 to 62·5)	62·4 (61·2 to 63·5)	177	14·7 (13·1 to 16·2)	30·8 (27·0 to 34·6)
Mauritania	59·5 (58·7 to 60·2)	65·0 (64·3 to 65·7)	65·0 (64·3 to 65·7)	64·6 (63·8 to 65·4)	66·2 (65·2 to 67·2)	67·5 (66·5 to 68·6)	150	8·0 (6·8 to 9·4)	13·5 (11·3 to 15·9)
Mozambique	49·5 (48·5 to 50·4)	56·9 (56·1 to 57·7)	59·4 (58·7 to 60·2)	57·0 (56·2 to 57·9)	61·0 (59·9 to 62·0)	62·0 (60·8 to 63·2)	178	12·6 (11·0 to 14·2)	25·4 (22·0 to 29·2)
Namibia	56·4 (56·0 to 56·8)	58·9 (58·5 to 59·3)	61·0 (60·5 to 61·4)	55·1 (54·6 to 55·5)	61·9 (61·3 to 62·4)	63·4 (62·6 to 64·1)	166	7·0 (6·1 to 7·8)	12·3 (10·8 to 13·9)
Niger	42·1 (40·8 to 43·4)	54·4 (53·5 to 55·3)	56·2 (55·4 to 57·0)	56·9 (56·1 to 57·8)	57·7 (56·7 to 58·7)	58·2 (57·1 to 59·3)	198	16·1 (14·5 to 17·8)	38·2 (33·3 to 43·2)
Nigeria	50·3 (49·3 to 51·2)	57·9 (57·2 to 58·8)	61·2 (60·5 to 62·0)	61·5 (60·6 to 62·4)	62·8 (61·8 to 63·9)	63·1 (61·9 to 64·1)	170	12·8 (11·3 to 14·3)	25·5 (22·1 to 29·0)
Rwanda	43·0 (42·0 to 43·9)	59·3 (58·6 to 60·0)	61·8 (61·1 to 62·6)	60·1 (59·2 to 61·0)	61·8 (60·8 to 62·9)	61·8 (60·7 to 63·0)	180	18·9 (17·4 to 20·4)	44·0 (39·9 to 48·2)
Senegal	55·2 (54·3 to 56·1)	65·1 (64·4 to 65·8)	66·4 (65·7 to 67·1)	64·8 (64·0 to 65·6)	68·1 (67·1 to 69·1)	68·5 (67·4 to 69·7)	138	13·3 (11·9 to 14·7)	24·2 (21·4 to 27·0)
Sierra Leone	44·9 (43·9 to 46·0)	52·1 (51·3 to 53·0)	55·4 (54·5 to 56·2)	56·4 (55·5 to 57·2)	56·9 (55·9 to 57·9)	57·3 (56·1 to 58·5)	199	12·4 (10·9 to 13·9)	27·7 (23·9 to 31·5)
Somalia	50·2 (49·2 to 51·2)	50·3 (49·4 to 51·2)	60·4 (59·6 to 61·1)	56·7 (55·9 to 57·5)	62·1 (61·1 to 63·2)	62·6 (61·6 to 63·7)	175	12·4 (10·9 to 13·9)	24·7 (21·5 to 28·1)
South Africa	63·0 (62·1 to 63·9)	55·4 (55·1 to 55·7)	61·2 (61·1 to 61·3)	58·5 (58·4 to 58·6)	62·4 (62·1 to 62·7)	63·3 (63·0 to 63·5)	167	0·3 (-0·7 to 1·2)	0·4 (-1·2 to 2·0)
South Sudan	47·8 (46·8 to 48·7)	54·0 (53·2 to 54·8)	53·4 (52·6 to 54·2)	53·5 (52·6 to 54·5)	54·0 (52·9 to 55·1)	54·1 (52·9 to 55·4)	204	6·4 (4·9 to 7·9)	13·4 (10·1 to 16·7)
São Tomé and Príncipe	61·7 (60·9 to 62·5)	64·3 (63·6 to 65·0)	65·0 (64·4 to 65·7)	65·0 (64·2 to 65·9)	67·3 (66·4 to 68·2)	68·6 (67·4 to 69·7)	137	6·8 (5·5 to 8·3)	11·1 (8·8 to 13·5)
Tanzania	53·8 (52·9 to 54·6)	61·5 (60·7 to 62·3)	65·4 (64·6 to 66·2)	63·7 (62·8 to 64·5)	67·2 (66·2 to 68·3)	67·8 (66·6 to 68·9)	146	14·0 (12·6 to 15·6)	26·1 (23·2 to 29·3)
The Gambia	59·0 (58·1 to 59·8)	64·3 (63·5 to 65·0)	62·6 (61·9 to 63·4)	62·1 (61·2 to 63·0)	65·1 (64·0 to 66·2)	66·1 (64·9 to 67·2)	157	7·1 (5·7 to 8·5)	12·1 (9·7 to 14·7)
Togo	53·4 (52·5 to 54·3)	57·7 (56·9 to 58·5)	61·3 (60·6 to 62·1)	61·3 (60·3 to 62·2)	62·2 (61·2 to 63·2)	62·8 (61·6 to 63·9)	174	9·3 (7·9 to 10·7)	17·5 (14·6 to 20·3)
Uganda	44·3 (43·5 to 45·2)	56·5 (55·7 to 57·3)	61·5 (60·7 to 62·3)	58·3 (57·4 to 59·2)	62·4 (61·3 to 63·4)	63·1 (61·9 to 64·3)	169	18·8 (17·2 to 20·3)	42·5 (38·3 to 46·4)
Zambia	47·8 (46·9 to 48·7)	53·9 (53·1 to 54·6)	57·1 (56·3 to 58·0)	53·8 (52·9 to 54·8)	59·5 (58·3 to 60·8)	60·4 (59·1 to 61·6)	191	12·5 (11·0 to 14·0)	26·2 (22·9 to 29·7)
Zimbabwe	61·4 (60·6 to 62·1)	54·8 (53·9 to 55·6)	61·0 (60·2 to 61·8)	57·0 (56·1 to 57·9)	62·5 (61·3 to 63·6)	63·8 (62·5 to 65·1)	163	2·4 (0·9 to 4·0)	3·9 (1·4 to 6·6)
**Low SDI**	**52·3 (52·0 to 52·6)**	**60·5 (60·3 to 60·7)**	**63·1 (62·9 to 63·3)**	**61·7 (61·5 to 61·9)**	**64·6 (64·3 to 65·0)**	**65·1 (64·7 to 65·5)**		**12·8 (12·3 to 13·2)**	**24·4 (23·4 to 25·4)**
**Low-middle SDI**	**58·3 (57·9 to 58·8)**	**64·8 (64·5 to 65·1)**	**66·8 (66·6 to 67·0)**	**65·3 (65·1 to 65·5)**	**68·1 (67·5 to 68·7)**	**68·4 (67·6 to 69·1)**		**10·0 (9·2 to 10·9)**	**17·2 (15·7 to 18·7)**

**Middle SDI**	**63·4 (63·1 to 63·7)**	**68·6 (68·4 to 68·9)**	**69·9 (69·7 to 70·1)**	**68·4 (68·1 to 68·7)**	**70·5 (70·1 to 70·9)**	**70·9 (70·5 to 71·3)**	**7·5 (7·0 to 8·0)**	**11·8 (11·0 to 12·6)**
**High-middle SDI**	**64·2 (63·8 to 64·6)**	**71·0 (70·7 to 71·2)**	**72·9 (72·8 to 73·0)**	**71·7 (71·5 to 71·9)**	**73·6 (73·1 to 74·1)**	**74·2 (73·6 to 74·7)**	**9·9 (9·2 to 10·7)**	**15·5 (14·3 to 16·7)**
**High SDI**	**68·8 (68·6 to 69·0)**	**74·4 (74·3 to 74·5)**	**76·3 (76·2 to 76·3)**	**75·7 (75·6 to 75·8)**	**76·2 (75·9 to 76·5)**	**76·8 (76·5 to 77·1)**	**8·0 (7·7 to 8·4)**	**11·7 (11·1 to 12·2)**

*United Nations convention recognises Taiwan as a province of China

Table S5: Male healthy life expectancy, 1990, 2010, 2020, 2021, 2022, and 2023, and absolute and relative changes, 1990–2023, globally, by SDI quintile, and for GBD super-regions and countries and territories

Location	1990	2010	2020	2021	2022	2023	Rank (2023)	Absolute Change (1990-2023)	Relative Change, % (1990-2023)
**Global**	**54·5 (52·4 to 56·4)**	**59·8 (57·5 to 61·9)**	**61·4 (58·9 to 63·5)**	**60·5 (58·0 to 62·5)**	**61·8 (59·3 to 63·9)**	**62·2 (59·6 to 64·4)**		**7·6 (7·1 to 8·2)**	**14·0 (13·2 to 14·7)**
**Central Europe, Eastern Europe, and Central Asia**	**56·8 (54·6 to 58·7)**	**58·6 (56·4 to 60·6)**	**60·3 (58·0 to 62·3)**	**59·6 (57·3 to 61·5)**	**60·2 (57·9 to 62·4)**	**61·1 (58·7 to 63·2)**		**4·3 (3·9 to 4·7)**	**7·5 (6·8 to 8·3)**
Albania	60·5 (58·1 to 62·7)	66·4 (63·7 to 68·7)	65·6 (63·1 to 67·8)	64·9 (62·3 to 67·2)	66·3 (63·4 to 68·7)	67·3 (64·6 to 69·8)	39	6·8 (6·0 to 7·6)	11·3 (9·9 to 12·6)
Armenia	58·9 (56·5 to 60·9)	62·4 (60·1 to 64·4)	57·1 (54·8 to 59·1)	62·6 (59·8 to 64·8)	64·3 (61·7 to 66·7)	66·3 (63·5 to 68·6)	45	7·4 (6·4 to 8·3)	12·6 (11·0 to 14·1)
Azerbaijan	55·0 (52·9 to 56·8)	61·0 (58·9 to 63·0)	59·1 (57·0 to 61·1)	60·4 (58·2 to 62·5)	61·9 (59·5 to 64·0)	61·4 (59·0 to 63·5)	116	6·4 (5·6 to 7·3)	11·6 (10·1 to 13·2)
Belarus	58·2 (56·1 to 60·1)	57·2 (55·1 to 59·0)	61·0 (58·6 to 63·0)	60·7 (58·4 to 62·6)	60·6 (58·4 to 62·6)	61·2 (58·7 to 63·4)	120	3·0 (2·3 to 3·5)	5·1 (3·9 to 6·0)
Bosnia and Herzegovina	60·8 (58·2 to 62·8)	64·2 (61·6 to 66·4)	63·6 (60·9 to 65·8)	62·6 (59·9 to 64·7)	64·1 (61·3 to 66·4)	65·1 (62·3 to 67·5)	62	4·4 (3·5 to 5·1)	7·2 (5·7 to 8·4)
Bulgaria	59·8 (57·5 to 61·7)	62·1 (59·7 to 64·1)	62·1 (59·8 to 64·1)	60·6 (58·2 to 62·6)	62·2 (59·7 to 64·3)	63·4 (60·8 to 65·6)	92	3·6 (3·1 to 4·1)	6·1 (5·2 to 6·8)
Croatia	60·4 (58·0 to 62·4)	64·2 (61·7 to 66·4)	64·9 (62·4 to 67·1)	64·1 (61·5 to 66·4)	64·5 (61·9 to 66·9)	65·3 (62·5 to 67·8)	57	4·9 (4·3 to 5·6)	8·2 (7·2 to 9·2)
Czechia	59·4 (57·0 to 61·4)	64·7 (62·1 to 67·0)	65·4 (62·7 to 67·7)	64·4 (61·7 to 66·7)	65·8 (63·0 to 68·2)	66·5 (63·6 to 68·9)	43	7·1 (6·3 to 7·7)	11·9 (10·9 to 12·7)
Estonia	57·0 (54·8 to 58·9)	61·7 (59·2 to 63·8)	64·7 (62·3 to 67·0)	63·8 (61·4 to 65·9)	64·0 (61·5 to 66·2)	64·6 (62·1 to 67·0)	72	7·6 (6·9 to 8·3)	13·4 (12·4 to 14·2)
Georgia	57·7 (55·6 to 59·5)	59·2 (56·9 to 61·1)	60·3 (57·9 to 62·3)	58·9 (56·7 to 61·0)	60·3 (57·9 to 62·6)	61·3 (58·5 to 63·6)	118	3·6 (2·6 to 4·4)	6·2 (4·7 to 7·7)
Hungary	57·1 (54·8 to 59·1)	62·1 (59·6 to 64·1)	63·3 (60·8 to 65·3)	62·1 (59·6 to 64·1)	63·3 (60·9 to 65·5)	64·0 (61·5 to 66·3)	78	6·9 (6·4 to 7·4)	12·2 (11·3 to 12·9)
Kazakhstan	55·5 (53·2 to 57·3)	56·4 (54·2 to 58·2)	59·7 (57·5 to 61·6)	59·2 (56·9 to 61·0)	61·5 (59·1 to 63·7)	62·1 (59·7 to 64·3)	108	6·7 (6·0 to 7·3)	12·0 (10·9 to 13·3)
Kyrgyzstan	55·4 (53·2 to 57·0)	58·0 (55·8 to 59·8)	60·6 (58·4 to 62·5)	61·3 (58·9 to 63·4)	63·2 (60·7 to 65·2)	63·8 (61·3 to 65·9)	85	8·5 (7·6 to 9·4)	15·3 (13·5 to 16·9)
Latvia	56·7 (54·5 to 58·6)	60·1 (57·8 to 62·1)	62·5 (60·1 to 64·5)	60·8 (58·5 to 62·6)	61·4 (59·1 to 63·4)	62·1 (59·5 to 64·2)	110	5·4 (4·8 to 5·9)	9·5 (8·5 to 10·4)
Lithuania	57·6 (55·3 to 59·6)	59·0 (56·7 to 60·9)	61·5 (59·0 to 63·5)	60·9 (58·6 to 62·9)	61·9 (59·4 to 64·0)	62·6 (60·1 to 64·8)	101	5·0 (4·3 to 5·4)	8·6 (7·6 to 9·5)
Moldova	57·1 (54·8 to 58·9)	58·8 (56·6 to 60·8)	61·1 (58·9 to 63·0)	60·4 (58·1 to 62·2)	62·3 (59·8 to 64·5)	62·9 (60·5 to 65·0)	95	5·9 (5·0 to 6·6)	10·3 (8·9 to 11·5)
Mongolia	51·6 (49·6 to 53·2)	55·8 (53·7 to 57·4)	60·2 (58·0 to 62·1)	58·7 (56·4 to 60·6)	59·3 (57·1 to 61·3)	59·9 (57·5 to 62·2)	140	8·3 (7·1 to 9·4)	16·0 (13·9 to 18·3)
Montenegro	61·9 (59·6 to 64·0)	63·9 (61·4 to 66·0)	63·8 (61·2 to 65·9)	61·9 (59·4 to 63·9)	64·1 (61·6 to 66·3)	65·0 (62·3 to 67·4)	66	3·1 (2·4 to 3·7)	5·0 (3·9 to 5·9)
North Macedonia	60·9 (58·6 to 62·8)	64·2 (61·6 to 66·3)	63·4 (60·9 to 65·5)	62·3 (59·8 to 64·4)	64·4 (61·8 to 66·7)	65·5 (62·9 to 67·7)	56	4·7 (4·0 to 5·3)	7·6 (6·7 to 8·6)
Poland	58·5 (56·1 to 60·5)	63·2 (60·7 to 65·5)	63·5 (60·9 to 65·7)	62·7 (60·1 to 64·9)	64·0 (61·3 to 66·3)	65·0 (62·3 to 67·4)	65	6·5 (5·9 to 7·1)	11·2 (10·4 to 12·0)
Romania	58·5 (56·1 to 60·6)	62·0 (59·6 to 63·9)	62·2 (59·9 to 64·2)	61·2 (58·9 to 63·1)	62·7 (60·4 to 64·8)	63·5 (61·1 to 65·7)	89	5·0 (4·5 to 5·4)	8·6 (7·8 to 9·5)
Russia	56·0 (53·8 to 57·9)	55·9 (53·9 to 57·8)	59·1 (56·9 to 61·0)	58·3 (56·0 to 60·1)	59·1 (56·8 to 61·2)	59·2 (56·8 to 61·4)	148	3·2 (2·4 to 3·9)	5·7 (4·2 to 6·9)
Serbia	59·5 (57·3 to 61·4)	63·3 (60·9 to 65·2)	63·7 (61·4 to 65·8)	62·6 (60·2 to 64·6)	64·2 (61·7 to 66·2)	64·8 (62·1 to 67·2)	68	5·3 (4·3 to 6·2)	8·9 (7·3 to 10·3)
Slovakia	58·6 (56·4 to 60·5)	62·8 (60·2 to 64·9)	64·2 (61·8 to 66·4)	62·5 (60·0 to 64·5)	64·1 (61·5 to 66·3)	65·1 (62·4 to 67·3)	63	6·5 (5·8 to 7·1)	11·0 (10·1 to 11·9)
Slovenia	60·4 (57·9 to 62·5)	65·7 (62·9 to 68·1)	67·2 (64·3 to 69·6)	67·1 (64·1 to 69·5)	67·4 (64·4 to 69·9)	67·9 (64·8 to 70·5)	34	7·4 (6·6 to 8·2)	12·3 (11·3 to 13·1)
Tajikistan	56·1 (53·9 to 58·1)	61·8 (59·6 to 63·9)	62·2 (59·9 to 64·2)	61·3 (59·0 to 63·4)	61·8 (59·3 to 63·9)	62·2 (59·5 to 64·6)	106	6·1 (4·9 to 7·1)	10·9 (8·7 to 12·7)
Turkmenistan	55·3 (53·2 to 57·1)	58·5 (56·4 to 60·5)	58·3 (56·0 to 60·3)	58·0 (55·7 to 60·1)	59·5 (57·0 to 61·6)	60·3 (57·7 to 62·6)	135	4·9 (3·7 to 6·0)	8·9 (6·6 to 10·9)
Ukraine	57·6 (55·3 to 59·5)	57·8 (55·6 to 59·6)	59·5 (57·2 to 61·4)	58·4 (56·2 to 60·3)	54·2 (50·9 to 57·2)	58·3 (56·0 to 60·3)	155	0·8 (0·2 to 1·4)	1·3 (0·3 to 2·4)
Uzbekistan	57·8 (55·7 to 59·7)	60·1 (57·8 to 62·0)	60·8 (58·4 to 62·6)	61·0 (58·6 to 63·0)	61·5 (59·1 to 63·5)	62·0 (59·7 to 64·0)	111	4·2 (3·6 to 4·9)	7·2 (6·1 to 8·4)
**High income**	**63·6 (61·2 to 65·8)**	**67·2 (64·5 to 69·6)**	**67·0 (64·3 to 69·5)**	**66·7 (63·9 to 69·2)**	**66·9 (64·1 to 69·4)**	**67·4 (64·5 to 69·9)**		**3·8 (3·2 to 4·2)**	**5·9 (5·2 to 6·5)**
Andorra	67·5 (65·0 to 69·6)	70·2 (67·5 to 72·6)	70·2 (67·5 to 72·7)	70·5 (67·7 to 73·1)	70·6 (67·8 to 73·1)	71·2 (68·3 to 73·7)	4	3·7 (2·9 to 4·4)	5·4 (4·4 to 6·4)
Argentina	60·9 (58·6 to 62·9)	63·9 (61·5 to 65·9)	64·4 (61·9 to 66·5)	62·8 (60·4 to 64·9)	64·2 (61·6 to 66·3)	65·3 (62·6 to 67·6)	58	4·4 (3·8 to 5·0)	7·3 (6·4 to 8·1)
Australia	64·1 (61·5 to 66·4)	68·2 (65·1 to 70·9)	69·5 (66·4 to 72·3)	69·5 (66·4 to 72·3)	69·0 (65·8 to 71·7)	69·5 (66·4 to 72·5)	17	5·4 (4·7 to 6·2)	8·4 (7·5 to 9·4)
Austria	63·7 (61·3 to 65·8)	67·6 (64·9 to 70·0)	68·5 (65·7 to 70·8)	68·3 (65·5 to 70·7)	68·5 (65·8 to 71·0)	68·9 (66·1 to 71·4)	23	5·2 (4·6 to 5·8)	8·2 (7·3 to 8·9)
Belgium	64·2 (61·9 to 66·2)	67·5 (64·7 to 69·9)	68·0 (65·2 to 70·3)	68·7 (65·8 to 71·1)	68·8 (65·9 to 71·3)	69·3 (66·3 to 71·8)	19	5·0 (4·3 to 5·6)	7·8 (6·9 to 8·6)
Brunei	62·1 (59·9 to 64·0)	66·2 (63·9 to 68·3)	67·4 (64·9 to 69·5)	67·1 (64·6 to 69·2)	66·8 (64·4 to 68·9)	67·4 (64·8 to 69·5)	38	5·3 (4·6 to 6·0)	8·5 (7·4 to 9·6)
Canada	64·5 (61·8 to 66·9)	68·0 (64·9 to 70·7)	67·6 (64·6 to 70·3)	67·4 (64·3 to 70·1)	67·2 (64·2 to 70·0)	67·7 (64·7 to 70·5)	36	3·2 (2·5 to 3·8)	4·9 (3·9 to 5·8)
Chile	61·7 (59·3 to 63·7)	66·5 (64·0 to 68·8)	66·7 (64·0 to 69·1)	66·1 (63·4 to 68·5)	66·9 (64·0 to 69·3)	67·8 (64·9 to 70·3)	35	6·1 (5·4 to 6·7)	9·9 (9·0 to 10·8)
Cyprus	64·8 (62·2 to 66·9)	68·1 (65·5 to 70·1)	68·8 (66·1 to 71·0)	68·4 (65·7 to 70·8)	68·5 (65·7 to 71·1)	68·8 (66·0 to 71·3)	25	4·0 (2·9 to 5·1)	6·2 (4·4 to 7·9)
Denmark	63·7 (61·2 to 65·7)	67·6 (65·0 to 69·8)	69·3 (66·4 to 71·6)	69·2 (66·3 to 71·6)	68·9 (66·0 to 71·3)	69·1 (66·3 to 71·5)	22	5·4 (4·8 to 6·0)	8·5 (7·8 to 9·2)
Finland	62·5 (60·2 to 64·6)	66·5 (63·8 to 68·9)	68·5 (65·7 to 71·0)	68·5 (65·6 to 70·9)	68·0 (65·2 to 70·4)	68·3 (65·4 to 70·9)	28	5·8 (5·1 to 6·4)	9·3 (8·3 to 10·1)
France	64·4 (62·0 to 66·5)	68·1 (65·5 to 70·5)	68·8 (65·8 to 71·2)	69·0 (66·1 to 71·5)	68·6 (65·8 to 71·2)	68·8 (65·9 to 71·4)	24	4·4 (3·8 to 5·0)	6·8 (6·0 to 7·5)
Germany	63·6 (61·3 to 65·7)	67·8 (65·0 to 70·1)	68·3 (65·6 to 70·7)	68·0 (65·3 to 70·3)	68·0 (65·3 to 70·4)	68·2 (65·4 to 70·5)	29	4·5 (3·9 to 5·1)	7·1 (6·3 to 7·8)
Greece	65·9 (63·6 to 68·0)	67·8 (65·2 to 70·1)	68·5 (65·8 to 70·8)	67·6 (64·9 to 69·9)	67·5 (64·8 to 69·8)	68·0 (65·2 to 70·3)	32	2·1 (1·6 to 2·6)	3·1 (2·4 to 3·9)
Greenland	52·8 (50·7 to 54·6)	57·9 (55·5 to 59·9)	59·1 (56·6 to 61·4)	58·7 (56·2 to 60·9)	59·7 (57·2 to 62·0)	60·4 (57·7 to 62·7)	134	7·6 (6·6 to 8·4)	14·4 (12·8 to 15·9)
Iceland	66·6 (64·3 to 68·6)	69·7 (67·0 to 72·2)	70·4 (67·6 to 73·0)	70·5 (67·7 to 73·0)	69·9 (67·1 to 72·5)	70·0 (67·2 to 72·4)	13	3·4 (2·7 to 4·0)	5·0 (4·1 to 5·9)
Ireland	64·0 (61·6 to 66·0)	68·6 (65·8 to 70·9)	69·8 (66·8 to 72·2)	69·9 (66·8 to 72·4)	69·7 (66·7 to 72·3)	69·9 (66·9 to 72·5)	15	5·9 (5·1 to 6·6)	9·2 (8·1 to 10·0)
Israel	67·0 (64·5 to 69·0)	70·3 (67·5 to 72·6)	70·9 (68·0 to 73·2)	70·7 (68·0 to 73·1)	71·0 (68·1 to 73·5)	70·9 (68·0 to 73·3)	6	3·9 (3·3 to 4·5)	5·8 (5·0 to 6·6)
Italy	65·1 (62·7 to 67·2)	69·6 (67·0 to 71·9)	69·8 (67·1 to 72·1)	70·1 (67·4 to 72·4)	69·8 (67·2 to 72·1)	70·4 (67·8 to 72·8)	9	5·3 (4·8 to 5·8)	8·2 (7·5 to 8·8)
Japan	67·1 (64·7 to 69·3)	69·8 (67·1 to 72·2)	71·3 (68·6 to 73·7)	71·2 (68·4 to 73·6)	70·9 (68·1 to 73·3)	70·7 (68·0 to 73·1)	7	3·6 (3·2 to 4·0)	5·4 (4·8 to 5·8)

Luxembourg	63·4 (61·2 to 65·4)	68·7 (66·1 to 71·1)	70·1 (67·3 to 72·5)	70·2 (67·4 to 72·7)	70·4 (67·6 to 72·9)	70·6 (67·7 to 73·3)	8	7·2 (6·5 to 8·0)	11·3 (10·4 to 12·3)
Malta	65·9 (63·6 to 67·9)	69·4 (66·6 to 71·6)	70·2 (67·5 to 72·5)	70·5 (67·7 to 72·9)	70·4 (67·5 to 72·6)	71·0 (68·1 to 73·4)	5	5·0 (4·3 to 5·8)	7·6 (6·6 to 8·6)
Monaco	64·8 (62·6 to 66·8)	66·5 (63·8 to 68·6)	67·4 (64·8 to 69·7)	67·0 (64·4 to 69·4)	67·7 (64·8 to 70·0)	68·1 (65·4 to 70·3)	31	3·4 (2·3 to 4·6)	5·2 (3·6 to 7·0)
Netherlands	65·2 (62·9 to 67·3)	66·8 (63·6 to 69·6)	68·9 (66·0 to 71·4)	68·8 (65·8 to 71·3)	69·0 (65·9 to 71·5)	69·2 (66·2 to 71·9)	21	4·0 (3·2 to 4·7)	6·1 (5·1 to 7·1)
New Zealand	62·9 (60·2 to 65·2)	67·9 (65·0 to 70·5)	68·9 (65·8 to 71·6)	68·8 (65·6 to 71·4)	68·2 (65·2 to 70·9)	68·4 (65·3 to 71·0)	27	5·5 (4·9 to 6·0)	8·8 (8·1 to 9·4)
Norway	64·7 (62·2 to 66·8)	68·6 (65·8 to 71·1)	70·6 (67·7 to 73·1)	70·6 (67·7 to 73·1)	70·1 (67·1 to 72·6)	70·3 (67·4 to 72·9)	11	5·7 (5·1 to 6·2)	8·8 (8·0 to 9·5)
Portugal	62·2 (59·9 to 64·3)	67·3 (64·6 to 69·6)	68·4 (65·6 to 70·8)	68·3 (65·4 to 70·7)	68·1 (65·4 to 70·5)	68·5 (65·6 to 71·0)	26	6·3 (5·5 to 7·0)	10·1 (9·1 to 11·0)
San Marino	66·1 (63·6 to 68·3)	71·0 (68·1 to 73·5)	71·1 (68·0 to 73·8)	71·4 (68·2 to 74·1)	70·9 (68·0 to 73·7)	71·7 (68·7 to 74·4)	2	5·6 (4·5 to 6·6)	8·5 (6·8 to 9·8)
Singapore	64·9 (62·6 to 66·7)	71·0 (68·3 to 73·2)	73·1 (70·4 to 75·5)	72·9 (70·2 to 75·3)	72·4 (69·7 to 74·8)	72·8 (70·0 to 75·2)	1	7·9 (7·2 to 8·6)	12·2 (11·3 to 13·1)
South Korea	59·3 (56·9 to 61·2)	67·4 (64·7 to 69·7)	70·1 (67·4 to 72·5)	70·2 (67·3 to 72·6)	69·6 (66·8 to 71·9)	70·2 (67·4 to 72·7)	12	10·8 (10·1 to 11·5)	18·3 (17·3 to 19·4)
Spain	64·5 (62·1 to 66·6)	69·2 (66·5 to 71·5)	69·4 (66·7 to 71·7)	69·7 (67·1 to 72·1)	69·3 (66·7 to 71·6)	69·9 (67·3 to 72·3)	14	5·4 (4·8 to 6·0)	8·4 (7·5 to 9·1)
Sweden	66·3 (63·8 to 68·4)	69·6 (66·9 to 71·9)	69·8 (66·8 to 72·3)	70·2 (67·1 to 72·7)	70·3 (67·3 to 72·9)	70·4 (67·3 to 73·0)	10	4·1 (3·4 to 4·9)	6·2 (5·2 to 7·1)
Switzerland	64·9 (62·4 to 67·2)	69·6 (66·7 to 72·1)	70·3 (67·6 to 72·8)	70·6 (67·7 to 73·2)	70·6 (67·5 to 73·2)	71·2 (68·2 to 73·9)	3	6·4 (5·7 to 7·0)	9·8 (9·0 to 10·5)
UK	64·3 (62·0 to 66·3)	68·2 (65·5 to 70·6)	67·7 (64·9 to 70·2)	67·8 (64·9 to 70·3)	68·0 (65·1 to 70·6)	68·0 (65·1 to 70·6)	33	3·7 (3·0 to 4·3)	5·7 (4·8 to 6·5)
USA	61·8 (59·1 to 64·2)	64·6 (61·8 to 67·3)	62·7 (59·9 to 65·3)	62·0 (59·2 to 64·6)	62·9 (60·0 to 65·5)	63·5 (60·6 to 66·3)	90	1·7 (1·2 to 2·2)	2·8 (2·1 to 3·5)
Uruguay	61·5 (59·1 to 63·5)	64·0 (61·4 to 66·1)	65·2 (62·5 to 67·3)	62·9 (60·4 to 64·9)	63·7 (61·2 to 65·7)	64·8 (62·1 to 66·9)	67	3·3 (2·7 to 3·8)	5·4 (4·4 to 6·2)
**Latin America and Caribbean**	**57·9 (55·4 to 60·1)**	**61·5 (58·9 to 63·7)**	**60·6 (58·0 to 62·8)**	**59·5 (57·0 to 61·7)**	**62·1 (59·4 to 64·5)**	**62·7 (59·9 to 65·1)**		**4·8 (4·3 to 5·2)**	**8·3 (7·6 to 9·0)**
Antigua and Barbuda	61·6 (58·9 to 63·9)	64·9 (61·8 to 67·2)	64·9 (61·8 to 67·4)	64·2 (61·1 to 66·6)	65·0 (61·8 to 67·7)	65·3 (62·3 to 68·1)	59	3·8 (2·5 to 4·9)	6·1 (4·2 to 8·0)
Bahamas	59·2 (56·9 to 61·1)	60·8 (58·3 to 62·9)	59·6 (57·0 to 61·5)	56·4 (54·1 to 58·3)	59·8 (57·2 to 62·0)	60·8 (58·2 to 63·1)	126	1·6 (0·8 to 2·4)	2·8 (1·4 to 4·0)
Barbados	62·7 (60·4 to 64·9)	65·0 (62·2 to 67·3)	64·6 (61·8 to 67·0)	63·9 (61·3 to 66·2)	63·8 (61·4 to 66·1)	64·6 (61·9 to 66·9)	73	1·9 (1·2 to 2·6)	3·0 (1·9 to 4·1)
Belize	62·2 (59·7 to 64·4)	62·1 (59·4 to 64·3)	61·1 (58·5 to 63·3)	59·7 (57·2 to 61·9)	61·4 (58·7 to 63·8)	62·7 (60·0 to 65·0)	99	0·4 (-0·4 to 1·2)	0·7 (-0·7 to 2·0)
Bermuda	62·3 (60·0 to 64·3)	67·7 (64·9 to 70·1)	67·5 (64·6 to 70·0)	66·9 (64·1 to 69·3)	67·2 (64·3 to 69·7)	67·6 (64·4 to 70·2)	37	5·3 (4·6 to 6·2)	8·6 (7·5 to 9·8)
Bolivia	53·1 (50·8 to 54·9)	59·1 (56·9 to 61·1)	54·2 (52·2 to 55·8)	54·6 (52·6 to 56·4)	59·6 (57·2 to 61·8)	60·7 (58·1 to 62·9)	128	7·7 (6·9 to 8·5)	14·4 (13·0 to 16·1)
Brazil	57·3 (54·7 to 59·4)	61·8 (59·2 to 64·0)	61·8 (59·2 to 64·1)	60·2 (57·7 to 62·5)	62·4 (59·6 to 64·8)	63·0 (60·2 to 65·4)	94	5·7 (5·1 to 6·3)	10·0 (9·0 to 10·8)
Colombia	59·3 (56·8 to 61·5)	64·9 (62·2 to 67·3)	64·5 (61·9 to 66·7)	62·5 (60·0 to 64·7)	65·3 (62·6 to 67·7)	66·4 (63·6 to 68·9)	44	7·2 (6·6 to 7·7)	12·1 (11·2 to 13·1)
Costa Rica	64·8 (62·0 to 67·0)	66·7 (63·8 to 69·2)	66·0 (63·1 to 68·6)	64·6 (61·8 to 67·0)	65·5 (62·8 to 68·2)	66·0 (63·1 to 68·6)	51	1·2 (0·6 to 1·8)	1·9 (0·9 to 2·8)
Cuba	64·5 (62·0 to 66·7)	66·9 (64·1 to 69·3)	65·8 (63·0 to 68·1)	62·7 (60·1 to 64·7)	65·7 (62·7 to 68·2)	65·9 (62·8 to 68·4)	52	1·4 (0·3 to 2·4)	2·2 (0·5 to 3·7)
Dominica	62·9 (60·2 to 65·1)	63·7 (61·0 to 66·0)	63·3 (60·5 to 65·6)	62·5 (59·7 to 64·8)	63·6 (60·6 to 66·0)	63·9 (60·8 to 66·3)	82	1·0 (0·4 to 1·8)	1·7 (0·6 to 2·8)
Dominican Republic	60·0 (57·5 to 62·0)	62·8 (60·2 to 65·1)	59·9 (57·3 to 61·9)	60·1 (57·4 to 62·2)	60·3 (57·6 to 62·4)	60·9 (58·0 to 63·1)	125	0·9 (0·2 to 1·7)	1·5 (0·3 to 2·8)
Ecuador	60·2 (57·7 to 62·2)	64·1 (61·5 to 66·4)	61·9 (59·6 to 64·0)	62·7 (60·2 to 64·9)	64·0 (61·3 to 66·4)	63·9 (61·3 to 66·2)	81	3·7 (3·1 to 4·3)	6·2 (5·1 to 7·1)
El Salvador	55·0 (52·3 to 57·2)	59·5 (56·9 to 61·8)	57·6 (55·0 to 59·6)	57·2 (54·8 to 59·4)	59·2 (56·6 to 61·3)	59·7 (57·0 to 62·0)	142	4·7 (3·8 to 5·7)	8·6 (6·8 to 10·5)
Grenada	59·6 (57·0 to 61·8)	63·1 (60·4 to 65·3)	62·3 (59·5 to 64·7)	60·3 (57·7 to 62·5)	62·2 (59·4 to 64·9)	63·1 (60·2 to 65·5)	93	3·5 (2·5 to 4·6)	5·9 (4·2 to 7·6)
Guatemala	51·2 (48·5 to 53·3)	58·6 (55·9 to 61·1)	58·4 (55·7 to 60·6)	56·6 (53·9 to 58·7)	59·1 (56·2 to 61·5)	60·2 (56·8 to 62·9)	136	9·0 (7·9 to 10·2)	17·6 (15·2 to 19·9)
Guyana	53·9 (51·6 to 56·0)	56·6 (54·4 to 58·7)	58·0 (55·6 to 60·4)	55·7 (53·4 to 57·7)	56·9 (54·3 to 59·2)	57·3 (54·8 to 59·6)	160	3·4 (2·1 to 4·9)	6·4 (4·0 to 9·1)
Haiti	44·2 (42·2 to 45·8)	31·1 (29·5 to 32·7)	52·9 (50·5 to 55·0)	52·3 (50·0 to 54·4)	52·8 (50·2 to 55·2)	52·4 (50·2 to 54·6)	193	8·2 (7·0 to 9·5)	18·7 (15·8 to 21·7)
Honduras	58·9 (56·3 to 61·1)	62·6 (59·9 to 64·9)	61·1 (58·4 to 63·2)	59·6 (56·9 to 61·8)	63·6 (60·7 to 66·1)	64·2 (61·2 to 66·8)	76	5·4 (4·6 to 6·1)	9·1 (7·8 to 10·5)
Jamaica	64·2 (61·7 to 66·5)	65·8 (63·0 to 68·1)	62·4 (59·8 to 64·9)	61·2 (58·7 to 63·5)	62·7 (60·0 to 65·2)	63·9 (60·9 to 66·5)	83	-0·4 (-1·4 to 0·7)	-0·6 (-2·2 to 1·0)
Mexico	57·8 (54·9 to 60·4)	62·2 (59·3 to 64·7)	58·3 (55·7 to 60·5)	58·1 (55·4 to 60·3)	61·4 (58·4 to 63·9)	62·3 (59·3 to 64·8)	104	4·5 (4·1 to 4·9)	7·8 (7·1 to 8·6)
Nicaragua	58·7 (55·8 to 60·9)	62·6 (60·0 to 64·8)	59·9 (57·4 to 62·0)	59·6 (57·1 to 61·7)	62·6 (59·9 to 65·1)	63·6 (60·7 to 66·2)	88	4·9 (3·9 to 5·9)	8·3 (6·6 to 10·0)
Panama	64·2 (61·6 to 66·4)	65·3 (62·5 to 67·7)	63·9 (61·3 to 66·2)	64·2 (61·6 to 66·6)	65·6 (62·8 to 68·0)	66·1 (63·0 to 68·6)	47	1·8 (1·1 to 2·5)	2·9 (1·8 to 3·9)
Paraguay	64·0 (61·4 to 66·2)	62·3 (59·8 to 64·5)	60·4 (58·0 to 62·6)	56·4 (54·1 to 58·3)	60·3 (57·7 to 62·7)	60·8 (58·1 to 63·3)	127	-3·2 (-4·0 to -2·5)	-5·0 (-6·3 to -3·9)
Peru	61·6 (58·9 to 63·9)	67·4 (64·6 to 69·7)	61·1 (58·6 to 63·1)	60·2 (57·8 to 62·2)	66·1 (63·4 to 68·5)	65·6 (62·9 to 67·7)	55	3·9 (3·3 to 4·6)	6·4 (5·3 to 7·6)
Puerto Rico	61·1 (58·4 to 63·3)	65·2 (62·5 to 67·6)	65·8 (62·8 to 68·3)	65·2 (62·3 to 67·8)	65·2 (62·2 to 67·7)	66·1 (62·9 to 68·7)	48	4·9 (4·2 to 5·7)	8·1 (6·9 to 9·2)
Saint Kitts and Nevis	57·9 (55·5 to 60·0)	61·4 (58·8 to 63·6)	61·9 (59·1 to 64·2)	60·9 (58·2 to 63·3)	61·9 (59·2 to 64·3)	62·4 (59·5 to 64·9)	103	4·5 (3·7 to 5·3)	7·7 (6·3 to 9·2)
Saint Lucia	59·5 (57·0 to 61·6)	63·1 (60·5 to 65·5)	63·6 (60·6 to 66·2)	61·8 (58·8 to 64·4)	63·6 (60·5 to 66·4)	64·2 (61·0 to 66·9)	75	4·7 (3·4 to 5·9)	7·9 (5·8 to 9·9)
Saint Vincent and the Grenadines	60·6 (58·1 to 62·7)	62·1 (59·1 to 64·6)	62·0 (59·2 to 64·4)	61·9 (59·1 to 64·2)	62·5 (59·6 to 65·0)	62·7 (59·7 to 65·4)	98	2·1 (1·0 to 3·2)	3·5 (1·8 to 5·3)
Suriname	59·5 (57·0 to 61·7)	60·5 (57·8 to 62·7)	60·0 (57·2 to 62·2)	56·5 (54·0 to 58·4)	59·8 (57·2 to 62·1)	60·7 (57·8 to 63·0)	129	1·1 (0·3 to 2·0)	1·9 (0·5 to 3·3)
Trinidad and Tobago	58·4 (56·1 to 60·4)	60·9 (58·0 to 63·2)	60·3 (57·8 to 62·4)	57·7 (55·3 to 59·6)	59·3 (56·6 to 61·4)	60·7 (57·8 to 63·1)	130	2·2 (1·4 to 3·0)	3·8 (2·4 to 5·1)
Venezuela	59·9 (57·4 to 62·0)	61·2 (58·5 to 63·6)	59·0 (56·3 to 61·1)	58·3 (55·6 to 60·3)	59·0 (56·6 to 61·3)	59·2 (56·8 to 61·5)	149	-0·7 (-1·3 to 0·0)	-1·2 (-2·2 to -0·1)
Virgin Islands	60·6 (58·2 to 62·7)	62·2 (59·5 to 64·5)	62·2 (59·3 to 64·6)	60·6 (57·9 to 62·9)	61·6 (58·8 to 64·3)	62·0 (59·2 to 64·8)	112	1·4 (0·1 to 2·8)	2·4 (0·1 to 4·6)
**North Africa and Middle East**	**55·8 (53·5 to 57·7)**	**61·7 (59·2 to 63·8)**	**61·0 (58·5 to 63·1)**	**60·7 (58·2 to 62·9)**	**62·5 (59·9 to 64·8)**	**62·6 (60·0 to 64·8)**		**6·8 (6·1 to 7·3)**	**12·1 (11·1 to 13·1)**
Afghanistan	48·0 (44·7 to 51·1)	54·2 (50·7 to 56·9)	52·5 (49·4 to 55·1)	49·8 (46·8 to 52·3)	55·9 (52·7 to 58·8)	56·7 (53·5 to 59·5)	162	8·7 (7·3 to 10·4)	18·1 (14·6 to 22·0)
Algeria	59·0 (56·7 to 61·0)	64·5 (61·8 to 66·6)	64·3 (61·7 to 66·4)	64·4 (61·9 to 66·6)	65·0 (62·4 to 67·2)	65·2 (62·5 to 67·4)	61	6·2 (5·4 to 6·8)	10·5 (9·3 to 11·4)
Bahrain	60·9 (58·5 to 62·9)	65·0 (62·3 to 67·1)	65·4 (62·5 to 67·8)	64·8 (61·9 to 67·2)	65·8 (62·7 to 68·1)	66·0 (63·0 to 68·5)	50	5·1 (4·0 to 6·0)	8·3 (6·7 to 9·8)

Egypt	55·4 (53·1 to 57·2)	59·7 (57·6 to 61·7)	59·7 (57·5 to 61·7)	59·2 (56·9 to 61·2)	60·2 (57·7 to 62·6)	60·6 (58·0 to 62·7)	131	5·3 (4·4 to 6·2)	9·5 (7·8 to 11·0)
Iran	56·7 (54·2 to 58·7)	64·6 (61·8 to 67·0)	62·8 (60·1 to 65·0)	63·2 (60·5 to 65·5)	65·6 (62·8 to 68·1)	66·2 (63·3 to 68·7)	46	9·5 (8·6 to 10·3)	16·8 (15·5 to 18·0)
Iraq	55·0 (52·5 to 56·9)	57·0 (54·5 to 59·0)	56·1 (53·6 to 58·0)	56·9 (54·4 to 58·9)	58·8 (56·1 to 60·9)	59·2 (56·6 to 61·4)	150	4·2 (3·5 to 5·0)	7·7 (6·3 to 9·1)
Jordan	60·5 (58·4 to 62·4)	64·7 (62·3 to 66·9)	65·0 (62·6 to 67·0)	63·9 (61·5 to 66·0)	66·4 (63·7 to 68·7)	67·2 (64·4 to 69·7)	40	6·7 (5·8 to 7·5)	11·0 (9·7 to 12·2)
Kuwait	61·6 (59·1 to 63·7)	67·1 (64·2 to 69·5)	67·5 (64·6 to 70·1)	67·4 (64·5 to 70·0)	68·7 (65·6 to 71·4)	69·3 (66·0 to 72·1)	20	7·7 (6·7 to 8·6)	12·4 (11·0 to 13·7)
Lebanon	61·8 (58·6 to 64·4)	67·7 (64·6 to 70·3)	60·6 (58·1 to 62·8)	63·5 (60·6 to 65·9)	64·9 (61·9 to 67·4)	65·6 (62·4 to 68·4)	53	3·8 (2·4 to 5·2)	6·1 (3·9 to 8·5)
Libya	60·3 (58·0 to 62·3)	63·7 (61·2 to 65·9)	61·3 (58·6 to 63·6)	60·3 (57·7 to 62·5)	63·4 (60·5 to 65·7)	60·9 (58·1 to 63·2)	124	0·6 (-0·2 to 1·3)	1·0 (-0·3 to 2·2)
Morocco	57·3 (54·9 to 59·1)	62·7 (60·2 to 64·7)	63·1 (60·7 to 65·2)	63·1 (60·7 to 65·3)	64·0 (61·3 to 66·2)	64·4 (61·7 to 66·5)	74	7·1 (6·1 to 8·3)	12·4 (10·7 to 14·5)
Oman	58·2 (56·0 to 60·2)	62·9 (60·4 to 65·0)	62·9 (60·3 to 65·0)	62·2 (59·6 to 64·3)	65·0 (62·4 to 67·4)	65·6 (62·6 to 67·9)	54	7·4 (6·5 to 8·1)	12·7 (11·3 to 14·1)
Palestine	60·0 (57·6 to 61·9)	63·4 (60·8 to 65·6)	64·5 (61·8 to 66·7)	63·2 (60·6 to 65·4)	64·9 (62·1 to 67·3)	52·8 (50·4 to 55·1)	191	-7·2 (-8·4 to -6·2)	-12·0 (-13·8 to -10·2)
Qatar	63·0 (60·5 to 65·1)	67·4 (64·7 to 69·7)	69·0 (66·1 to 71·6)	68·9 (65·9 to 71·6)	69·4 (66·4 to 72·3)	69·7 (66·7 to 72·3)	16	6·7 (5·8 to 7·8)	10·6 (9·2 to 12·2)
Saudi Arabia	58·0 (55·6 to 60·1)	60·9 (58·3 to 63·0)	62·2 (59·7 to 64·5)	63·0 (60·3 to 65·4)	63·5 (60·7 to 65·9)	63·6 (60·8 to 66·1)	87	5·6 (4·7 to 6·5)	9·7 (8·3 to 11·2)
Sudan	48·1 (46·0 to 50·0)	56·6 (54·3 to 58·6)	58·4 (56·0 to 60·7)	58·8 (56·2 to 61·1)	59·8 (57·3 to 62·0)	59·0 (56·4 to 61·2)	151	10·8 (9·6 to 12·1)	22·5 (19·8 to 25·1)
Syria	58·9 (56·5 to 60·9)	64·0 (61·3 to 66·1)	61·6 (58·6 to 64·0)	61·6 (58·7 to 64·1)	63·1 (60·0 to 65·6)	62·7 (59·6 to 65·3)	97	3·8 (2·8 to 4·7)	6·5 (4·9 to 7·9)
Tunisia	58·7 (56·5 to 60·7)	62·6 (60·2 to 64·6)	61·6 (59·2 to 63·7)	59·8 (57·5 to 61·9)	63·2 (60·7 to 65·4)	63·7 (61·0 to 65·9)	86	5·0 (4·3 to 5·5)	8·5 (7·4 to 9·4)
Türkiye	57·5 (55·3 to 59·4)	65·6 (63·1 to 67·8)	64·5 (62·0 to 66·6)	63·9 (61·3 to 66·0)	65·1 (62·5 to 67·2)	64·7 (62·4 to 67·1)	69	7·2 (6·3 to 8·2)	12·6 (10·8 to 14·2)
United Arab Emirates	61·3 (58·9 to 63·3)	66·7 (63·9 to 69·0)	69·3 (66·3 to 71·8)	69·1 (66·2 to 71·7)	69·4 (66·5 to 72·2)	69·4 (66·5 to 72·2)	18	8·1 (7·2 to 9·1)	13·2 (11·9 to 14·6)
Yemen	50·1 (47·8 to 52·3)	58·5 (56·2 to 60·9)	55·9 (53·5 to 58·0)	56·5 (53·8 to 58·7)	59·1 (56·3 to 61·3)	60·0 (57·1 to 62·4)	139	9·9 (8·7 to 11·3)	19·8 (17·5 to 22·3)
**South Asia**	**50·8 (48·6 to 53·0)**	**57·4 (54·9 to 59·4)**	**59·5 (56·9 to 61·7)**	**58·0 (55·6 to 60·0)**	**60·6 (58·1 to 62·9)**	**61·0 (58·4 to 63·3)**		**10·1 (9·2 to 11·0)**	**19·9 (18·0 to 21·7)**
Bangladesh	50·2 (47·9 to 52·1)	59·3 (56·9 to 61·4)	60·4 (58·0 to 62·5)	59·4 (57·1 to 61·6)	61·9 (59·4 to 64·2)	62·2 (59·8 to 64·4)	107	12·0 (10·7 to 13·2)	23·8 (21·2 to 26·8)
Bhutan	53·2 (51·3 to 55·3)	61·2 (58·8 to 63·3)	63·8 (61·4 to 65·9)	64·0 (61·6 to 66·4)	63·9 (61·5 to 66·1)	64·1 (61·6 to 66·4)	77	10·9 (9·8 to 12·1)	20·5 (18·3 to 22·5)
India	50·6 (48·4 to 52·7)	57·3 (54·8 to 59·4)	59·9 (57·2 to 62·0)	58·2 (55·8 to 60·3)	60·7 (58·2 to 63·0)	61·1 (58·5 to 63·5)	122	10·4 (9·4 to 11·6)	20·6 (18·3 to 22·8)
Nepal	50·4 (48·0 to 52·3)	59·9 (57·5 to 62·0)	61·2 (58·7 to 63·4)	59·2 (56·7 to 61·3)	62·3 (59·6 to 64·8)	62·6 (59·8 to 65·3)	100	12·3 (11·0 to 13·7)	24·4 (21·6 to 27·1)
Pakistan	53·9 (51·5 to 56·0)	57·2 (54·6 to 59·4)	57·7 (54·9 to 59·8)	57·4 (54·5 to 59·9)	60·0 (57·3 to 62·4)	60·5 (57·5 to 63·0)	132	6·6 (5·2 to 7·8)	12·2 (9·9 to 14·3)
**Southeast Asia, East Asia, and Oceania**	**56·7 (54·8 to 58·5)**	**63·3 (61·0 to 65·3)**	**66·3 (63·8 to 68·3)**	**65·3 (62·9 to 67·3)**	**65·6 (63·0 to 67·9)**	**65·9 (63·4 to 68·2)**		**9·2 (8·4 to 10·1)**	**16·2 (14·8 to 17·5)**
American Samoa	58·6 (56·1 to 60·4)	59·8 (57·4 to 62·0)	59·2 (56·6 to 61·1)	57·1 (54·8 to 59·0)	59·1 (56·5 to 61·4)	59·6 (57·0 to 61·9)	143	1·0 (0·0 to 2·1)	1·7 (0·0 to 3·6)
Cambodia	48·4 (46·2 to 50·3)	59·3 (56·7 to 61·2)	62·7 (60·1 to 65·0)	62·2 (59·5 to 64·2)	62·7 (60·1 to 64·8)	62·5 (60·0 to 64·7)	102	14·1 (12·7 to 15·4)	29·1 (26·0 to 31·9)
China	57·2 (55·3 to 58·9)	64·8 (62·4 to 66·8)	68·6 (66·0 to 70·8)	68·3 (65·7 to 70·3)	67·9 (65·1 to 70·4)	68·2 (65·5 to 70·7)	30	11·0 (10·0 to 12·2)	19·3 (17·4 to 21·2)
Cook Islands	58·4 (56·2 to 60·1)	61·2 (58·8 to 63·2)	61·3 (58·9 to 63·4)	60·1 (57·7 to 62·1)	61·1 (58·6 to 63·3)	61·3 (58·6 to 63·6)	119	2·9 (2·1 to 3·7)	5·0 (3·7 to 6·2)
Federated States of Micronesia	51·9 (50·1 to 53·4)	54·9 (52·8 to 56·6)	55·5 (53·1 to 57·3)	54·2 (51·8 to 56·0)	55·5 (53·0 to 57·4)	55·9 (53·5 to 57·9)	164	4·0 (3·2 to 4·8)	7·7 (6·3 to 9·2)
Fiji	57·3 (55·1 to 59·1)	58·1 (55·9 to 59·9)	58·5 (56·3 to 60·4)	57·0 (54·9 to 58·9)	57·9 (55·6 to 60·0)	57·8 (55·5 to 59·9)	158	0·5 (-0·3 to 1·3)	0·9 (-0·6 to 2·3)
Guam	63·6 (61·5 to 65·6)	64·8 (62·4 to 66·9)	63·8 (61·4 to 65·9)	62·7 (60·3 to 64·7)	63·1 (60·5 to 65·2)	63·5 (60·7 to 65·6)	91	-0·2 (-0·9 to 0·5)	-0·3 (-1·4 to 0·8)
Indonesia	55·2 (53·1 to 57·2)	59·9 (57·8 to 61·8)	61·1 (58·9 to 63·0)	58·8 (56·6 to 60·5)	62·0 (59·6 to 64·1)	62·1 (59·6 to 64·4)	109	6·9 (5·8 to 8·0)	12·5 (10·3 to 14·5)
Kiribati	53·2 (50·9 to 55·2)	57·1 (54·7 to 59·1)	58·5 (55·8 to 60·6)	57·2 (54·8 to 59·3)	58·6 (56·1 to 60·7)	58·9 (56·1 to 61·0)	152	5·7 (4·8 to 6·4)	10·7 (9·0 to 12·1)
Laos	43·7 (42·0 to 45·4)	56·0 (54·2 to 57·6)	58·7 (56·6 to 60·5)	58·6 (56·8 to 60·5)	58·4 (56·3 to 60·3)	58·6 (56·2 to 60·6)	153	14·9 (13·4 to 16·3)	34·0 (30·3 to 37·8)
Malaysia	61·6 (59·6 to 63·5)	64·8 (62·5 to 66·9)	66·1 (63·9 to 68·1)	63·5 (61·5 to 65·3)	64·8 (62·7 to 66·8)	66·0 (63·7 to 68·2)	49	4·4 (3·8 to 5·0)	7·2 (6·2 to 8·1)
Maldives	57·7 (55·7 to 59·5)	66·1 (63·8 to 68·0)	67·2 (64·8 to 69·1)	66·5 (64·1 to 68·4)	66·8 (64·6 to 68·8)	67·2 (64·9 to 69·0)	41	9·4 (8·8 to 10·1)	16·3 (15·2 to 17·6)
Marshall Islands	52·3 (50·5 to 54·0)	54·5 (52·5 to 56·3)	55·4 (53·3 to 57·2)	54·2 (52·2 to 56·0)	55·5 (53·4 to 57·4)	55·8 (53·6 to 57·6)	165	3·4 (2·7 to 4·2)	6·6 (5·2 to 7·9)
Mauritius	58·8 (56·6 to 60·6)	62·1 (59·7 to 64·2)	62·4 (59·8 to 64·5)	60·9 (58·6 to 63·0)	61·4 (59·0 to 63·4)	61·6 (59·2 to 63·9)	114	2·9 (2·2 to 3·6)	4·9 (3·8 to 5·9)
Myanmar	45·2 (43·2 to 46·7)	53·2 (51·2 to 54·9)	55·2 (53·0 to 57·1)	53·5 (51·5 to 55·5)	54·7 (52·5 to 56·8)	54·8 (52·3 to 56·9)	178	9·6 (8·2 to 11·0)	21·3 (18·0 to 24·4)
Nauru	49·7 (47·9 to 51·0)	49·5 (47·7 to 50·8)	50·5 (48·6 to 52·2)	48·6 (46·7 to 50·2)	50·6 (48·5 to 52·2)	50·9 (48·8 to 52·6)	199	1·2 (0·3 to 2·0)	2·3 (0·6 to 4·1)
Niue	58·6 (56·5 to 60·5)	60·7 (58·4 to 62·7)	61·1 (58·6 to 63·2)	59·6 (57·3 to 61·6)	60·9 (58·4 to 62·8)	61·1 (58·6 to 63·1)	121	2·5 (1·8 to 3·1)	4·3 (3·1 to 5·3)
North Korea	60·5 (58·1 to 62·3)	62·0 (59·8 to 63·9)	62·8 (60·1 to 64·8)	63·0 (60·4 to 65·1)	62·9 (60·4 to 65·0)	62·9 (60·2 to 65·0)	96	2·4 (1·2 to 3·5)	4·0 (2·0 to 5·7)
Northern Mariana Islands	59·0 (57·0 to 60·7)	60·4 (58·2 to 62·3)	60·3 (57·9 to 62·2)	59·3 (56·9 to 61·2)	60·5 (58·1 to 62·5)	60·9 (58·6 to 62·9)	123	1·9 (1·2 to 2·7)	3·2 (2·1 to 4·5)
Palau	56·8 (54·7 to 58·6)	58·8 (56·5 to 60·7)	59·0 (56·6 to 61·2)	57·6 (55·2 to 59·7)	59·0 (56·3 to 61·3)	59·3 (56·7 to 61·6)	146	2·5 (1·7 to 3·2)	4·4 (3·1 to 5·6)
Papua New Guinea	53·4 (51·1 to 55·5)	56·2 (54·1 to 58·1)	58·1 (55·7 to 60·3)	57·4 (55·0 to 59·5)	57·8 (55·5 to 60·0)	57·9 (55·3 to 60·3)	156	4·5 (3·2 to 5·8)	8·4 (6·2 to 10·7)
Philippines	58·1 (55·8 to 59·9)	60·3 (58·2 to 62·0)	61·2 (59·2 to 62·9)	58·2 (56·4 to 59·7)	60·3 (58·3 to 62·0)	61·3 (59·2 to 63·1)	117	3·2 (2·9 to 3·8)	5·6 (4·8 to 6·6)
Samoa	60·6 (58·3 to 62·6)	62·3 (60·0 to 64·2)	62·3 (59·9 to 64·4)	61·0 (58·6 to 63·1)	62·2 (59·5 to 64·4)	62·2 (59·5 to 64·4)	105	1·6 (0·9 to 2·3)	2·7 (1·4 to 3·9)
Seychelles	59·2 (57·1 to 60·8)	62·0 (59·8 to 63·9)	64·5 (62·2 to 66·4)	62·3 (60·1 to 64·2)	64·2 (61·8 to 66·3)	63·9 (61·5 to 65·9)	80	4·7 (4·0 to 5·4)	7·9 (6·7 to 9·1)
Solomon Islands	53·3 (51·5 to 54·9)	55·7 (53·7 to 57·3)	55·3 (53·3 to 56·9)	54·8 (52·8 to 56·3)	55·2 (53·2 to 56·9)	55·4 (53·4 to 57·1)	170	2·0 (1·3 to 2·7)	3·8 (2·4 to 5·1)
Sri Lanka	58·0 (55·9 to 59·9)	62·6 (60·4 to 64·6)	66·6 (64·1 to 68·7)	65·2 (62·8 to 67·2)	65·0 (62·6 to 67·1)	65·1 (62·7 to 67·2)	64	7·0 (6·5 to 7·6)	12·1 (11·2 to 13·2)
Taiwan*	63·7 (61·4 to 65·6)	67·0 (64·4 to 69·2)	68·0 (65·3 to 70·4)	67·7 (65·1 to 70·1)	66·9 (64·3 to 69·2)	67·2 (64·5 to 69·5)	42	3·5 (2·9 to 4·0)	5·4 (4·6 to 6·2)
Thailand	60·2 (58·0 to 62·3)	63·5 (61·4 to 65·5)	65·0 (62·7 to 67·1)	64·1 (61·7 to 66·2)	63·9 (61·6 to 66·0)	64·7 (62·2 to 66·8)	71	4·4 (3·9 to 4·8)	7·3 (6·6 to 8·0)
Timor-Leste	50·1 (48·1 to 51·9)	59·8 (57·7 to 61·8)	60·9 (58·8 to 62·9)	60·8 (58·4 to 62·9)	61·3 (59·0 to 63·3)	61·6 (59·2 to 63·6)	115	11·5 (10·3 to 12·7)	23·0 (20·7 to 25·2)

Tokelau	61·9 (59·3 to 63·9)	64·4 (61·8 to 66·6)	65·5 (62·6 to 67·8)	64·0 (61·2 to 66·2)	64·9 (62·0 to 67·2)	65·2 (62·2 to 67·8)	60	3·3 (2·6 to 4·1)	5·4 (4·1 to 6·6)
Tonga	60·4 (58·2 to 62·2)	61·9 (59·5 to 63·9)	62·0 (59·4 to 64·1)	60·5 (57·9 to 62·5)	61·7 (58·9 to 64·0)	62·0 (59·2 to 64·3)	113	1·6 (0·7 to 2·3)	2·6 (1·2 to 3·8)
Tuvalu	50·1 (48·2 to 51·6)	53·3 (51·2 to 54·9)	53·8 (51·6 to 55·4)	52·3 (50·2 to 53·9)	53·7 (51·5 to 55·3)	53·8 (51·6 to 55·6)	183	3·7 (3·0 to 4·4)	7·4 (6·0 to 9·0)
Vanuatu	55·8 (53·8 to 57·6)	58·8 (56·6 to 60·6)	59·3 (57·0 to 61·1)	58·5 (56·3 to 60·3)	59·3 (57·0 to 61·2)	59·6 (57·2 to 61·4)	144	3·8 (3·0 to 4·6)	6·7 (5·4 to 8·3)
Viet Nam	57·6 (55·5 to 59·5)	62·2 (60·1 to 64·0)	63·8 (61·5 to 65·8)	63·2 (61·1 to 64·9)	63·7 (61·4 to 65·7)	64·0 (61·6 to 66·3)	79	6·4 (5·4 to 7·5)	11·2 (9·3 to 13·2)
**Sub-Saharan Africa**	**43·7 (41·9 to 45·3)**	**50·3 (48·3 to 52·0)**	**53·0 (50·9 to 54·7)**	**51·8 (49·8 to 53·6)**	**53·9 (51·7 to 55·8)**	**54·5 (52·3 to 56·3)**		**10·8 (10·2 to 11·4)**	**24·7 (23·4 to 26·0)**
Angola	40·6 (38·6 to 42·3)	49·3 (47·3 to 51·2)	51·5 (49·3 to 53·4)	50·6 (48·6 to 52·4)	52·6 (50·1 to 54·9)	52·9 (50·7 to 55·1)	189	12·3 (11·1 to 13·8)	30·4 (27·1 to 33·9)
Benin	45·0 (43·1 to 46·7)	52·4 (50·4 to 54·2)	54·9 (52·9 to 56·9)	55·1 (53·0 to 57·2)	55·6 (53·5 to 57·8)	55·9 (53·6 to 58·1)	163	10·9 (9·8 to 12·3)	24·2 (21·2 to 27·2)
Botswana	53·5 (51·4 to 55·4)	54·7 (52·3 to 56·8)	57·6 (55·3 to 59·8)	55·7 (53·6 to 57·8)	57·9 (55·6 to 60·2)	58·6 (56·1 to 60·9)	154	5·1 (4·2 to 6·0)	9·5 (7·9 to 11·1)
Burkina Faso	42·0 (39·9 to 43·7)	53·5 (51·1 to 55·4)	56·8 (54·3 to 58·7)	56·3 (53·9 to 58·4)	57·2 (54·7 to 59·4)	57·3 (54·6 to 59·5)	161	15·2 (13·7 to 16·8)	36·3 (32·1 to 40·4)
Burundi	40·5 (38·7 to 41·9)	49·5 (47·4 to 51·2)	51·5 (49·4 to 53·2)	49·9 (47·9 to 51·7)	51·9 (49·8 to 53·9)	52·3 (50·0 to 54·4)	194	11·8 (10·4 to 13·3)	29·3 (25·3 to 33·3)
Cabo Verde	58·5 (56·0 to 60·3)	62·8 (60·5 to 65·0)	61·3 (59·1 to 63·4)	60·3 (58·0 to 62·5)	62·9 (60·4 to 65·3)	63·9 (61·1 to 66·1)	84	5·4 (4·3 to 6·5)	9·2 (7·2 to 11·0)
Cameroon	46·6 (44·4 to 48·5)	48·6 (46·6 to 50·3)	52·1 (50·2 to 53·9)	51·2 (49·3 to 52·9)	53·3 (51·4 to 55·4)	53·7 (51·7 to 55·8)	185	7·2 (6·1 to 8·5)	15·4 (13·0 to 18·3)
Central African Republic	40·3 (38·0 to 42·2)	43·8 (41·6 to 45·7)	45·2 (43·1 to 47·2)	45·0 (42·6 to 47·2)	45·8 (43·1 to 47·8)	46·3 (44·2 to 48·5)	204	6·0 (4·9 to 7·3)	14·9 (12·1 to 18·4)
Chad	41·2 (39·2 to 42·7)	46·6 (44·7 to 48·3)	48·3 (46·3 to 50·0)	49·4 (47·1 to 51·1)	49·5 (47·4 to 51·3)	49·9 (47·7 to 51·8)	201	8·7 (7·4 to 10·0)	21·2 (17·7 to 24·7)
Comoros	56·2 (53·9 to 58·4)	63·8 (60·9 to 66·2)	62·9 (60·2 to 65·0)	60·3 (57·7 to 62·5)	63·7 (60·9 to 66·3)	64·7 (61·9 to 67·3)	70	8·5 (7·3 to 9·9)	15·1 (13·1 to 17·8)
Congo (Brazzaville)	47·1 (45·0 to 48·8)	53·3 (51·2 to 55·3)	53·8 (51·4 to 55·9)	53·0 (50·8 to 55·3)	54·5 (52·1 to 56·6)	54·9 (52·3 to 57·0)	175	7·7 (6·2 to 9·1)	16·5 (13·4 to 19·2)
Côte d'Ivoire	45·9 (43·8 to 47·6)	51·2 (49·1 to 53·1)	53·8 (51·7 to 55·9)	53·6 (51·3 to 55·8)	54·4 (52·0 to 56·5)	55·0 (52·5 to 57·1)	174	9·0 (7·8 to 10·2)	19·6 (16·8 to 22·7)
DR Congo	43·1 (41·0 to 45·0)	48·2 (46·1 to 50·2)	50·4 (48·2 to 52·2)	50·4 (48·1 to 52·2)	51·4 (49·2 to 53·4)	52·2 (49·8 to 54·3)	195	9·1 (7·7 to 10·3)	21·1 (17·4 to 24·2)
Djibouti	52·8 (50·5 to 54·6)	56·3 (54·0 to 58·1)	57·9 (55·7 to 59·9)	56·2 (54·0 to 58·2)	58·6 (56·3 to 60·9)	59·3 (56·8 to 61·4)	147	6·5 (5·3 to 7·8)	12·4 (10·0 to 14·9)
Equatorial Guinea	40·0 (38·1 to 41·7)	50·0 (48·0 to 51·9)	50·0 (48·1 to 51·9)	49·7 (47·4 to 51·7)	51·1 (48·8 to 53·1)	51·5 (49·2 to 53·6)	197	11·4 (10·1 to 12·9)	28·6 (24·6 to 32·6)
Eritrea	34·6 (32·4 to 36·5)	50·3 (48·0 to 52·4)	52·3 (49·9 to 54·3)	52·1 (49·6 to 54·3)	51·5 (49·3 to 53·7)	53·3 (50·9 to 55·5)	186	18·7 (16·8 to 20·3)	54·0 (47·1 to 61·5)
Eswatini	47·1 (45·5 to 48·6)	39·7 (38·1 to 41·2)	48·7 (46·6 to 50·7)	47·6 (45·4 to 49·6)	50·2 (47·6 to 52·2)	51·3 (48·9 to 53·3)	198	4·2 (2·7 to 5·7)	8·9 (5·7 to 12·1)
Ethiopia	36·5 (34·8 to 38·1)	52·0 (50·0 to 53·8)	54·2 (52·0 to 56·1)	52·5 (50·4 to 54·4)	54·9 (52·7 to 56·9)	55·5 (53·3 to 57·7)	169	19·1 (17·8 to 20·6)	52·3 (47·9 to 56·8)
Gabon	52·2 (49·9 to 54·3)	55·9 (53·3 to 57·9)	56·3 (53·9 to 58·4)	55·9 (53·3 to 58·1)	57·2 (54·7 to 59·6)	57·7 (55·2 to 60·0)	159	5·5 (4·0 to 6·7)	10·5 (7·7 to 12·9)
Ghana	47·6 (45·5 to 49·4)	53·0 (50·9 to 54·7)	54·5 (52·4 to 56·5)	53·9 (51·8 to 55·9)	55·1 (52·9 to 57·2)	55·7 (53·2 to 57·8)	167	8·0 (6·5 to 9·4)	16·9 (13·7 to 19·6)
Guinea	42·1 (40·2 to 43·9)	50·9 (48·9 to 52·8)	53·0 (51·0 to 54·7)	52·8 (50·7 to 54·7)	53·7 (51·6 to 55·8)	54·4 (52·1 to 56·5)	181	12·3 (10·8 to 13·6)	29·1 (25·3 to 32·8)
Guinea-Bissau	42·7 (40·7 to 44·6)	49·8 (47·8 to 51·6)	51·2 (49·1 to 53·0)	51·6 (49·4 to 53·5)	52·5 (50·4 to 54·5)	53·3 (51·1 to 55·1)	187	10·6 (9·3 to 12·0)	24·7 (21·2 to 28·5)
Kenya	53·4 (51·3 to 55·1)	55·4 (53·4 to 57·3)	56·6 (54·4 to 58·5)	55·4 (53·1 to 57·4)	59·2 (56·6 to 61·4)	60·0 (57·3 to 62·1)	138	6·6 (5·2 to 7·8)	12·3 (9·7 to 14·6)
Lesotho	49·0 (47·3 to 50·7)	44·1 (42·1 to 45·8)	47·2 (45·1 to 49·3)	46·4 (44·4 to 48·5)	47·8 (45·6 to 49·8)	48·3 (46·2 to 50·5)	202	-0·7 (-2·0 to 0·7)	-1·4 (-4·2 to 1·3)
Liberia	37·4 (35·6 to 39·0)	49·0 (46·6 to 51·0)	49·3 (47·0 to 51·2)	47·8 (45·8 to 49·6)	51·9 (49·5 to 53·9)	52·6 (50·1 to 55·0)	192	15·2 (13·9 to 16·5)	40·7 (36·3 to 44·6)
Madagascar	44·8 (42·8 to 46·5)	52·9 (50·7 to 54·5)	53·2 (51·3 to 55·0)	52·1 (50·2 to 54·0)	53·6 (51·6 to 55·6)	54·4 (52·6 to 56·4)	180	9·6 (8·4 to 10·7)	21·5 (18·6 to 24·4)
Malawi	39·0 (37·4 to 40·2)	48·9 (46·7 to 50·5)	52·0 (49·8 to 53·6)	47·2 (45·6 to 48·6)	52·0 (49·7 to 53·8)	52·9 (50·7 to 54·9)	188	14·0 (12·6 to 15·3)	35·8 (32·0 to 39·5)
Mali	41·5 (39·4 to 43·3)	51·6 (49·2 to 53·5)	52·2 (50·0 to 54·1)	51·7 (49·5 to 53·6)	53·4 (51·1 to 55·3)	54·3 (51·6 to 56·5)	182	12·8 (11·3 to 14·4)	30·9 (27·1 to 34·7)
Mauritania	52·6 (50·5 to 54·4)	57·6 (55·4 to 59·6)	57·1 (55·2 to 59·5)	57·1 (54·5 to 59·2)	58·5 (56·2 to 60·7)	59·7 (57·3 to 61·7)	141	7·1 (5·8 to 8·2)	13·4 (11·1 to 15·7)
Mozambique	42·5 (40·4 to 44·3)	49·4 (47·2 to 51·4)	51·7 (49·4 to 53·8)	49·8 (47·7 to 51·8)	52·9 (50·5 to 55·2)	53·8 (51·1 to 55·9)	184	11·2 (9·6 to 12·7)	26·4 (22·4 to 30·5)
Namibia	49·8 (47·8 to 51·4)	51·8 (49·7 to 53·5)	53·6 (51·4 to 55·3)	48·9 (47·1 to 50·4)	54·1 (52·0 to 56·0)	55·4 (53·0 to 57·3)	171	5·5 (4·6 to 6·4)	11·1 (9·3 to 12·8)
Niger	37·1 (35·1 to 38·7)	48·2 (46·3 to 50·0)	49·8 (47·9 to 51·5)	50·3 (48·2 to 52·1)	51·0 (48·9 to 52·9)	51·5 (49·5 to 53·6)	196	14·4 (13·0 to 16·3)	38·7 (34·4 to 44·1)
Nigeria	43·8 (41·6 to 45·6)	50·8 (48·5 to 52·8)	53·6 (51·4 to 55·6)	53·7 (51·4 to 55·7)	54·8 (52·2 to 56·9)	55·1 (52·7 to 57·2)	173	11·3 (9·9 to 12·6)	25·9 (22·2 to 29·3)
Rwanda	38·4 (36·9 to 39·8)	52·3 (50·0 to 54·1)	54·7 (52·6 to 56·7)	53·2 (51·2 to 55·0)	54·6 (52·3 to 56·8)	54·7 (52·4 to 56·6)	179	16·3 (15·0 to 17·8)	42·6 (38·8 to 47·0)
Senegal	48·3 (46·2 to 50·2)	57·1 (54·8 to 59·3)	58·4 (56·1 to 60·3)	56·9 (54·5 to 58·9)	59·5 (56·9 to 62·1)	60·0 (57·5 to 62·3)	137	11·7 (10·4 to 12·9)	24·2 (21·2 to 27·1)
Sierra Leone	39·4 (37·6 to 41·0)	45·8 (43·9 to 47·5)	48·8 (46·6 to 50·6)	49·5 (47·3 to 51·4)	50·0 (48·0 to 52·0)	50·5 (48·2 to 52·5)	200	11·1 (9·7 to 12·5)	28·2 (23·8 to 32·0)
Somalia	44·3 (42·0 to 46·0)	44·5 (42·6 to 46·2)	52·9 (50·8 to 54·8)	49·8 (47·5 to 51·9)	54·2 (51·8 to 56·6)	54·8 (52·3 to 57·0)	177	10·5 (9·2 to 11·8)	23·8 (20·6 to 27·3)
South Africa	55·1 (52·8 to 57·2)	48·7 (46·9 to 50·4)	53·3 (51·2 to 55·3)	51·1 (49·1 to 52·9)	54·1 (51·8 to 56·1)	54·9 (52·6 to 57·0)	176	-0·3 (-1·1 to 0·5)	-0·5 (-1·9 to 1·0)
South Sudan	41·8 (39·8 to 43·6)	47·3 (45·0 to 49·0)	46·6 (44·3 to 48·4)	46·5 (44·1 to 48·3)	47·1 (44·5 to 49·0)	47·3 (44·7 to 49·0)	203	5·5 (4·1 to 6·8)	13·2 (9·8 to 16·4)
São Tomé and Príncipe	54·6 (52·3 to 56·6)	57·1 (54·9 to 59·0)	57·7 (55·5 to 59·6)	57·5 (55·2 to 59·4)	59·4 (57·1 to 61·5)	60·5 (58·2 to 62·6)	133	5·8 (4·6 to 7·2)	10·7 (8·3 to 13·1)
Tanzania	46·8 (44·8 to 48·5)	54·0 (51·7 to 56·0)	57·5 (55·1 to 59·5)	56·0 (53·7 to 58·0)	58·9 (56·3 to 61·1)	59·5 (56·9 to 61·6)	145	12·6 (11·5 to 13·9)	27·0 (24·1 to 29·8)
The Gambia	51·9 (49·7 to 53·8)	56·5 (54·2 to 58·7)	55·2 (52·9 to 57·2)	54·5 (52·0 to 56·6)	57·0 (54·5 to 59·1)	57·9 (55·3 to 60·0)	157	6·0 (4·8 to 7·2)	11·7 (9·3 to 14·0)
Togo	46·9 (45·0 to 48·6)	50·8 (48·7 to 52·6)	54·1 (51·9 to 55·9)	53·9 (51·8 to 55·9)	54·7 (52·5 to 57·0)	55·2 (52·8 to 57·5)	172	8·3 (7·1 to 9·6)	17·7 (15·2 to 20·5)
Uganda	38·6 (36·7 to 40·2)	49·8 (47·8 to 51·5)	54·4 (52·0 to 56·2)	51·7 (49·5 to 53·4)	54·9 (52·5 to 57·0)	55·6 (53·1 to 57·9)	168	17·0 (15·6 to 18·5)	44·0 (40·1 to 47·8)
Zambia	42·0 (40·2 to 43·8)	47·2 (45·0 to 49·1)	50·2 (47·9 to 52·2)	47·4 (45·4 to 49·3)	52·0 (49·6 to 54·1)	52·8 (50·5 to 55·0)	190	10·7 (9·4 to 12·2)	25·6 (22·3 to 29·1)
Zimbabwe	54·0 (51·7 to 56·1)	48·0 (46·3 to 49·7)	53·5 (51·4 to 55·4)	50·2 (47·9 to 52·0)	54·5 (51·9 to 56·7)	55·7 (53·3 to 58·1)	166	1·8 (0·6 to 3·2)	3·3 (1·0 to 5·9)
**Low SDI**	**45·6 (43·7 to 47·3)**	**53·0 (50·8 to 54·8)**	**55·2 (53·0 to 57·1)**	**54·0 (51·8 to 55·8)**	**56·3 (54·0 to 58·3)**	**56·8 (54·5 to 58·9)**		**11·2 (10·6 to 11·7)**	**24·5 (23·3 to 26·0)**
**Low-middle SDI**	**50·9 (48·8 to 52·9)**	**56·7 (54·4 to 58·6)**	**58·4 (55·9 to 60·4)**	**57·2 (54·7 to 59·1)**	**59·3 (57·0 to 61·4)**	**59·6 (57·1 to 61·7)**		**8·7 (7·9 to 9·4)**	**17·0 (15·4 to 18·6)**

**Middle SDI**	**55·8 (53·8 to 57·7)**	**60·6 (58·4 to 62·5)**	**61·6 (59·4 to 63·6)**	**60·4 (58·3 to 62·3)**	**62·0 (59·7 to 64·1)**	**62·4 (60·0 to 64·4)**	**6·5 (5·9 to 7·0)**	**11·7 (10·7 to 12·5)**
**High-middle SDI**	**56·6 (54·5 to 58·5)**	**62·3 (59·9 to 64·4)**	**63·7 (61·2 to 65·9)**	**62·7 (60·3 to 64·8)**	**64·1 (61·6 to 66·5)**	**64·6 (62·0 to 67·0)**	**8·0 (7·3 to 8·8)**	**14·1 (13·0 to 15·3)**
**High SDI**	**60·4 (58·2 to 62·4)**	**64·9 (62·4 to 67·1)**	**66·4 (63·7 to 68·6)**	**65·8 (63·2 to 68·1)**	**66·1 (63·5 to 68·4)**	**66·6 (63·9 to 69·0)**	**6·2 (5·6 to 6·7)**	**10·2 (9·4 to 10·8)**

*United Nations convention recognises Taiwan as a province of China

Table S6: Male morbidity gap, 1990, 2010, 2020, 2021, 2022, and 2023, and absolute and relative changes, 1990–2023, globally, by SDI quintile, and for GBD super-regions and countries and territories

Location	1990	2010	2020	2021	2022	2023	Rank (2023)	Absolute Change (1990-2023)	Relative Change, % (1990-2023)
**Global**	**7·6 (5·8 to 9·8)**	**8·6 (6·5 to 11·0)**	**9·0 (6·9 to 11·5)**	**8·8 (6·8 to 11·3)**	**9·2 (7·0 to 11·8)**	**9·3 (7·0 to 11·9)**		**1·7 (1·1 to 2·4)**	**22·2 (15·1 to 30·5)**
**Central Europe, Eastern Europe, and Central Asia**	**8·0 (6·0 to 10·2)**	**7·9 (6·0 to 10·2)**	**8·3 (6·3 to 10·6)**	**8·2 (6·3 to 10·5)**	**8·6 (6·4 to 11·0)**	**8·7 (6·6 to 11·2)**		**0·8 (0·2 to 1·4)**	**10·0 (2·7 to 17·1)**
Albania	8·7 (6·3 to 11·3)	9·5 (7·2 to 12·4)	9·4 (7·2 to 12·0)	9·6 (7·3 to 12·2)	9·9 (7·4 to 12·7)	10·0 (7·3 to 12·8)	59	1·3 (0·1 to 2·5)	14·8 (0·9 to 30·0)
Armenia	8·2 (5·9 to 10·7)	8·3 (6·2 to 10·8)	8·0 (5·9 to 10·4)	9·2 (6·9 to 11·9)	9·7 (7·2 to 12·4)	10·0 (7·5 to 13·2)	58	1·9 (0·6 to 3·2)	23·3 (7·0 to 44·4)
Azerbaijan	7·0 (5·1 to 9·2)	7·7 (5·8 to 10·0)	7·7 (5·8 to 9·9)	8·1 (6·0 to 10·4)	8·5 (6·3 to 11·0)	8·5 (6·1 to 11·0)	128	1·4 (0·1 to 2·7)	20·5 (2·0 to 41·2)
Belarus	7·8 (5·8 to 10·0)	7·5 (5·7 to 9·6)	8·1 (6·1 to 10·6)	8·3 (6·2 to 10·7)	8·3 (6·1 to 10·6)	8·3 (6·1 to 10·8)	139	0·6 (-0·4 to 1·5)	7·3 (-4·8 to 19·2)
Bosnia and Herzegovina	8·7 (6·6 to 11·3)	9·5 (7·2 to 12·2)	9·5 (7·3 to 12·2)	9·4 (7·2 to 12·2)	9·9 (7·7 to 12·7)	10·2 (7·7 to 13·2)	54	1·5 (0·4 to 2·7)	17·2 (4·6 to 31·8)
Bulgaria	8·3 (6·3 to 10·6)	8·5 (6·5 to 11·0)	8·6 (6·5 to 10·9)	8·3 (6·4 to 10·7)	8·8 (6·8 to 11·4)	9·1 (6·9 to 11·7)	102	0·8 (0·3 to 1·4)	9·6 (3·0 to 16·9)
Croatia	8·4 (6·3 to 10·7)	9·2 (6·9 to 11·8)	9·7 (7·5 to 12·3)	9·7 (7·5 to 12·4)	9·8 (7·5 to 12·6)	10·1 (7·6 to 13·0)	56	1·8 (1·0 to 2·5)	21·2 (12·6 to 30·0)
Czechia	8·3 (6·3 to 10·7)	9·8 (7·4 to 12·5)	10·0 (7·6 to 12·7)	9·9 (7·6 to 12·5)	10·3 (7·9 to 13·2)	10·5 (8·0 to 13·4)	38	2·1 (1·5 to 2·9)	25·8 (19·2 to 32·3)
Estonia	7·8 (5·8 to 10·1)	8·7 (6·5 to 11·1)	9·3 (7·1 to 11·8)	9·1 (7·0 to 11·6)	9·3 (7·0 to 12·0)	9·6 (7·2 to 12·2)	82	1·8 (1·0 to 2·5)	22·7 (12·9 to 32·9)
Georgia	7·4 (5·4 to 9·7)	7·8 (5·8 to 10·2)	8·4 (6·3 to 10·7)	8·2 (6·1 to 10·7)	8·7 (6·3 to 11·2)	8·8 (6·3 to 11·5)	112	1·4 (0·1 to 2·7)	19·8 (2·2 to 38·5)
Hungary	8·1 (6·1 to 10·4)	8·6 (6·5 to 11·1)	9·0 (6·9 to 11·5)	8·7 (6·7 to 11·3)	9·2 (7·0 to 11·7)	9·4 (7·1 to 12·1)	90	1·3 (0·8 to 1·9)	16·2 (9·8 to 23·0)
Kazakhstan	7·5 (5·6 to 9·8)	7·4 (5·5 to 9·6)	7·8 (5·9 to 10·0)	7·8 (5·9 to 10·0)	8·5 (6·3 to 11·0)	8·7 (6·5 to 11·2)	120	1·2 (0·3 to 2·0)	16·3 (4·5 to 28·3)
Kyrgyzstan	7·3 (5·4 to 9·5)	7·4 (5·5 to 9·7)	7·7 (5·8 to 10·1)	8·3 (6·2 to 10·7)	8·7 (6·4 to 11·3)	8·7 (6·4 to 11·4)	119	1·4 (0·1 to 2·6)	19·1 (1·7 to 39·8)
Latvia	8·0 (6·0 to 10·3)	8·0 (6·0 to 10·3)	8·5 (6·5 to 11·0)	8·1 (6·2 to 10·3)	8·4 (6·3 to 10·8)	8·7 (6·6 to 11·3)	118	0·6 (-0·1 to 1·4)	7·9 (-1·5 to 17·1)
Lithuania	8·2 (6·3 to 10·6)	8·2 (6·1 to 10·6)	8·7 (6·6 to 11·2)	8·7 (6·6 to 11·1)	9·0 (6·7 to 11·4)	9·3 (7·0 to 11·9)	96	1·0 (0·3 to 1·8)	12·4 (3·4 to 22·5)
Moldova	7·6 (5·6 to 9·9)	7·5 (5·5 to 9·9)	8·1 (6·1 to 10·3)	8·0 (6·0 to 10·3)	8·6 (6·3 to 11·3)	8·7 (6·4 to 11·3)	117	1·1 (-0·1 to 2·3)	14·2 (-1·8 to 30·9)
Mongolia	6·4 (4·6 to 8·6)	6·9 (5·1 to 9·0)	7·9 (5·9 to 10·3)	7·6 (5·7 to 9·9)	7·9 (5·7 to 10·3)	8·1 (5·6 to 10·7)	152	1·7 (0·1 to 3·3)	27·0 (1·7 to 57·2)
Montenegro	8·3 (6·2 to 10·7)	8·7 (6·7 to 11·3)	8·9 (6·8 to 11·5)	8·6 (6·6 to 11·0)	9·5 (7·2 to 12·1)	9·7 (7·3 to 12·5)	72	1·4 (0·6 to 2·2)	16·7 (7·6 to 27·2)
North Macedonia	8·2 (6·2 to 10·5)	8·8 (6·7 to 11·4)	8·9 (6·8 to 11·4)	8·9 (6·8 to 11·4)	9·5 (7·0 to 12·1)	9·7 (7·4 to 12·4)	77	1·5 (0·6 to 2·4)	18·7 (7·7 to 31·1)
Poland	8·3 (6·3 to 10·7)	9·0 (6·8 to 11·6)	9·2 (7·0 to 11·8)	9·1 (7·0 to 11·7)	9·6 (7·3 to 12·3)	9·9 (7·5 to 12·7)	62	1·6 (1·0 to 2·3)	19·6 (13·2 to 25·4)
Romania	8·4 (6·4 to 10·9)	8·2 (6·3 to 10·6)	8·3 (6·3 to 10·6)	8·2 (6·3 to 10·5)	8·7 (6·7 to 11·1)	9·0 (6·8 to 11·4)	109	0·6 (0·0 to 1·1)	6·6 (0·3 to 13·1)
Russia	7·9 (6·0 to 10·2)	7·6 (5·7 to 9·7)	8·0 (6·1 to 10·2)	8·0 (6·1 to 10·3)	8·4 (6·3 to 10·9)	8·4 (6·0 to 10·9)	133	0·5 (-0·7 to 1·5)	6·1 (-9·7 to 19·6)
Serbia	7·7 (5·7 to 10·0)	8·4 (6·4 to 10·8)	8·6 (6·5 to 11·0)	8·4 (6·4 to 10·8)	9·0 (6·8 to 11·5)	9·1 (6·6 to 12·1)	101	1·4 (0·1 to 2·7)	18·3 (1·3 to 35·2)
Slovakia	8·1 (6·2 to 10·4)	8·9 (6·8 to 11·5)	9·2 (7·0 to 11·8)	8·7 (6·6 to 11·2)	9·3 (7·1 to 12·0)	9·7 (7·4 to 12·4)	74	1·6 (0·9 to 2·4)	19·6 (11·8 to 27·5)
Slovenia	8·7 (6·5 to 11·2)	10·2 (7·8 to 13·1)	10·4 (8·0 to 13·3)	10·5 (8·0 to 13·5)	10·8 (8·2 to 13·9)	11·1 (8·3 to 14·2)	15	2·4 (1·6 to 3·2)	27·6 (20·7 to 35·6)
Tajikistan	7·4 (5·4 to 9·8)	8·1 (6·0 to 10·5)	8·4 (6·4 to 10·7)	8·3 (6·1 to 11·0)	8·4 (5·9 to 11·1)	8·4 (5·8 to 11·3)	137	1·0 (-0·6 to 2·6)	13·6 (-7·9 to 38·1)
Turkmenistan	7·2 (5·2 to 9·4)	7·6 (5·5 to 9·7)	7·5 (5·3 to 10·1)	7·6 (5·4 to 10·1)	8·0 (5·5 to 10·7)	8·0 (5·2 to 10·7)	160	0·8 (-0·9 to 2·8)	11·6 (-13·7 to 39·4)
Ukraine	8·2 (6·3 to 10·4)	7·6 (5·7 to 9·7)	8·0 (6·1 to 10·3)	7·9 (6·0 to 10·1)	7·4 (2·7 to 11·7)	8·2 (6·2 to 10·7)	145	0·1 (-0·8 to 1·0)	1·2 (-10·3 to 13·9)
Uzbekistan	7·4 (5·5 to 9·7)	7·4 (5·6 to 9·8)	7·8 (5·9 to 10·2)	8·1 (6·1 to 10·5)	8·1 (6·0 to 10·5)	8·0 (6·0 to 10·4)	164	0·6 (-0·2 to 1·5)	8·2 (-2·3 to 21·0)
**High income**	**9·1 (6·9 to 11·5)**	**10·4 (8·0 to 13·2)**	**10·8 (8·3 to 13·6)**	**10·8 (8·3 to 13·7)**	**10·9 (8·4 to 13·8)**	**11·2 (8·6 to 14·1)**		**2·1 (1·6 to 2·6)**	**23·0 (19·5 to 27·0)**
Andorra	9·1 (6·8 to 11·6)	10·1 (7·6 to 13·0)	10·2 (7·6 to 13·0)	10·6 (7·9 to 13·4)	10·5 (7·8 to 13·4)	10·6 (7·8 to 13·6)	27	1·6 (0·5 to 2·6)	17·4 (5·5 to 29·7)
Argentina	8·0 (6·0 to 10·3)	8·6 (6·6 to 11·1)	8·9 (6·8 to 11·4)	8·6 (6·5 to 11·1)	9·0 (6·8 to 11·5)	9·3 (7·0 to 12·0)	92	1·3 (0·6 to 2·0)	16·2 (7·8 to 24·7)
Australia	9·6 (7·3 to 12·2)	11·5 (8·8 to 14·6)	12·2 (9·4 to 15·4)	12·2 (9·4 to 15·3)	12·1 (9·3 to 15·3)	12·4 (9·4 to 15·6)	2	2·8 (2·0 to 3·5)	28·6 (23·8 to 33·8)
Austria	8·6 (6·5 to 11·0)	10·1 (7·6 to 12·8)	10·3 (8·0 to 13·2)	10·4 (8·0 to 13·2)	10·5 (8·1 to 13·3)	10·6 (8·1 to 13·5)	28	2·1 (1·5 to 2·7)	24·1 (18·7 to 29·6)
Belgium	8·4 (6·3 to 10·7)	9·8 (7·5 to 12·6)	10·0 (7·7 to 12·8)	10·5 (8·0 to 13·4)	10·6 (8·1 to 13·6)	10·7 (8·1 to 13·7)	26	2·3 (1·7 to 3·0)	27·5 (21·9 to 33·2)
Brunei	8·1 (6·1 to 10·5)	8·9 (6·7 to 11·3)	9·3 (7·1 to 12·0)	9·3 (7·1 to 11·8)	9·2 (6·9 to 11·7)	9·5 (7·2 to 12·1)	86	1·3 (0·3 to 2·4)	16·7 (3·7 to 30·7)
Canada	9·6 (7·3 to 12·4)	11·3 (8·6 to 14·4)	11·7 (9·0 to 14·7)	11·8 (9·0 to 14·8)	11·7 (9·0 to 14·8)	12·0 (9·2 to 15·0)	3	2·3 (1·6 to 3·1)	24·3 (17·7 to 30·7)
Chile	8·4 (6·3 to 10·8)	9·4 (7·1 to 11·9)	9·8 (7·4 to 12·5)	9·7 (7·3 to 12·4)	10·0 (7·6 to 12·8)	10·4 (7·9 to 13·3)	43	2·0 (1·3 to 2·8)	24·0 (17·1 to 31·9)
Cyprus	8·0 (5·7 to 10·8)	9·0 (6·8 to 11·8)	9·4 (7·2 to 12·3)	9·4 (7·0 to 12·2)	9·6 (6·8 to 12·5)	9·8 (6·9 to 12·8)	67	1·8 (0·0 to 3·4)	22·7 (0·6 to 47·9)
Denmark	8·5 (6·4 to 11·0)	9·6 (7·4 to 12·3)	10·3 (7·9 to 13·1)	10·3 (7·9 to 13·2)	10·3 (7·9 to 13·3)	10·4 (8·0 to 13·3)	42	1·9 (1·2 to 2·5)	22·1 (15·8 to 28·6)
Finland	8·6 (6·5 to 11·0)	10·3 (7·9 to 13·1)	10·7 (8·2 to 13·5)	10·7 (8·2 to 13·6)	10·6 (8·1 to 13·5)	10·8 (8·2 to 13·8)	23	2·2 (1·6 to 3·0)	26·1 (19·8 to 32·9)
France	8·7 (6·6 to 11·1)	10·1 (7·7 to 12·7)	10·6 (8·1 to 13·6)	10·8 (8·3 to 13·8)	10·8 (8·2 to 13·7)	10·9 (8·3 to 13·9)	21	2·2 (1·6 to 2·8)	24·8 (19·9 to 30·2)
Germany	8·5 (6·4 to 10·9)	10·0 (7·7 to 12·8)	10·3 (8·0 to 13·1)	10·2 (7·9 to 12·9)	10·2 (7·8 to 12·9)	10·3 (7·8 to 13·1)	46	1·8 (1·2 to 2·5)	21·4 (15·8 to 27·8)
Greece	8·8 (6·7 to 11·1)	9·9 (7·6 to 12·6)	10·2 (7·9 to 12·9)	10·0 (7·7 to 12·8)	10·1 (7·8 to 12·9)	10·3 (7·9 to 13·1)	50	1·5 (0·9 to 2·1)	16·8 (11·1 to 22·6)
Greenland	7·3 (5·2 to 9·5)	8·5 (6·4 to 11·0)	9·0 (6·7 to 11·7)	9·1 (6·7 to 11·6)	9·4 (7·0 to 12·0)	9·7 (7·3 to 12·5)	79	2·4 (1·2 to 3·6)	33·4 (16·5 to 51·6)
Iceland	8·9 (6·8 to 11·4)	10·2 (7·6 to 13·1)	10·8 (8·2 to 13·7)	10·9 (8·4 to 13·8)	10·8 (8·1 to 13·6)	10·8 (8·3 to 13·6)	24	2·0 (1·1 to 2·8)	22·4 (13·6 to 31·7)
Ireland	8·3 (6·2 to 10·7)	9·8 (7·5 to 12·6)	10·5 (8·0 to 13·6)	10·7 (8·1 to 13·8)	10·8 (8·2 to 13·8)	10·9 (8·3 to 13·9)	20	2·6 (1·9 to 3·5)	32·2 (24·9 to 39·9)
Israel	8·6 (6·6 to 11·0)	9·8 (7·5 to 12·6)	10·1 (7·7 to 12·9)	10·1 (7·7 to 13·0)	10·2 (7·7 to 13·0)	10·3 (7·9 to 13·3)	49	1·7 (1·1 to 2·4)	19·7 (13·0 to 28·0)
Italy	8·6 (6·5 to 10·9)	9·6 (7·3 to 12·2)	9·8 (7·5 to 12·6)	10·0 (7·7 to 12·8)	10·0 (7·6 to 12·7)	10·3 (7·8 to 13·0)	51	1·7 (1·2 to 2·3)	20·1 (15·4 to 24·0)

Japan	8·9 (6·7 to 11·3)	9·9 (7·6 to 12·6)	10·4 (8·0 to 13·2)	10·4 (8·0 to 13·2)	10·3 (7·9 to 13·1)	10·3 (7·9 to 13·1)	47	1·4 (1·1 to 1·8)	16·1 (12·5 to 20·2)
Luxembourg	8·3 (6·3 to 10·6)	9·6 (7·3 to 12·4)	10·2 (7·7 to 13·1)	10·4 (8·0 to 13·3)	10·5 (7·9 to 13·5)	10·6 (7·9 to 13·6)	29	2·3 (1·4 to 3·3)	27·6 (17·7 to 38·6)
Malta	8·1 (6·0 to 10·6)	9·3 (7·1 to 12·2)	9·9 (7·4 to 12·6)	10·1 (7·7 to 12·9)	10·1 (7·7 to 13·0)	10·4 (7·9 to 13·4)	41	2·3 (1·4 to 3·2)	28·3 (19·0 to 40·0)
Monaco	8·0 (5·8 to 10·5)	8·6 (6·3 to 11·4)	9·3 (6·8 to 12·1)	9·4 (6·8 to 12·1)	9·6 (7·0 to 12·6)	9·8 (7·2 to 12·9)	66	1·8 (0·1 to 3·6)	22·9 (1·7 to 51·2)
Netherlands	8·5 (6·5 to 10·9)	11·9 (9·0 to 15·1)	10·6 (8·1 to 13·5)	10·8 (8·2 to 13·8)	11·0 (8·4 to 14·0)	11·2 (8·5 to 14·3)	12	2·7 (1·9 to 3·5)	31·2 (25·8 to 36·9)
New Zealand	9·8 (7·4 to 12·6)	11·1 (8·4 to 14·0)	11·6 (8·9 to 14·8)	11·6 (8·9 to 14·7)	11·4 (8·7 to 14·6)	11·5 (8·8 to 14·7)	5	1·7 (1·2 to 2·4)	17·8 (13·1 to 23·0)
Norway	8·9 (6·7 to 11·3)	10·1 (7·7 to 12·9)	10·7 (8·2 to 13·7)	10·8 (8·3 to 13·8)	10·8 (8·2 to 13·7)	11·0 (8·4 to 14·0)	17	2·2 (1·6 to 2·8)	24·7 (19·3 to 29·9)
Portugal	8·5 (6·5 to 10·8)	9·6 (7·3 to 12·3)	10·1 (7·7 to 13·0)	10·1 (7·7 to 13·0)	10·2 (7·8 to 13·0)	10·5 (8·0 to 13·4)	36	2·0 (1·3 to 2·7)	23·6 (16·3 to 30·9)
San Marino	8·8 (6·4 to 11·3)	10·6 (8·0 to 13·6)	10·9 (8·1 to 14·1)	11·3 (8·4 to 14·7)	11·1 (8·3 to 14·4)	11·5 (8·5 to 14·6)	6	2·7 (0·8 to 4·4)	30·9 (10·3 to 55·9)
Singapore	8·0 (6·1 to 10·3)	9·4 (7·2 to 12·1)	10·4 (7·9 to 13·0)	10·3 (7·8 to 13·0)	10·2 (7·7 to 13·0)	10·4 (7·9 to 13·2)	45	2·3 (1·6 to 3·1)	29·5 (20·7 to 39·3)
South Korea	8·2 (6·2 to 10·7)	9·8 (7·5 to 12·6)	10·4 (8·0 to 13·2)	10·4 (8·0 to 13·2)	10·2 (7·8 to 13·0)	10·5 (8·0 to 13·3)	39	2·3 (1·5 to 3·1)	27·9 (18·3 to 37·6)
Spain	8·8 (6·6 to 11·2)	9·7 (7·4 to 12·4)	10·0 (7·7 to 12·7)	10·3 (7·9 to 13·0)	10·3 (7·9 to 12·9)	10·5 (8·0 to 13·1)	35	1·7 (1·1 to 2·4)	19·6 (14·5 to 25·1)
Sweden	8·6 (6·5 to 11·1)	10·0 (7·6 to 12·7)	10·8 (8·3 to 13·9)	11·0 (8·5 to 14·2)	11·2 (8·5 to 14·2)	11·2 (8·6 to 14·3)	11	2·6 (1·9 to 3·4)	30·5 (24·0 to 36·9)
Switzerland	9·4 (7·0 to 11·9)	10·5 (8·0 to 13·5)	10·8 (8·4 to 13·6)	11·0 (8·5 to 14·0)	11·2 (8·5 to 14·2)	11·3 (8·6 to 14·4)	8	2·0 (1·3 to 2·7)	21·0 (15·9 to 26·6)
UK	8·6 (6·6 to 10·9)	10·1 (7·8 to 12·9)	10·6 (8·1 to 13·4)	10·8 (8·3 to 13·7)	11·0 (8·4 to 13·9)	11·1 (8·5 to 14·0)	16	2·4 (1·8 to 3·1)	28·5 (25·0 to 32·8)
USA	10·2 (7·8 to 12·9)	11·6 (8·9 to 14·5)	11·7 (9·1 to 14·5)	11·6 (9·1 to 14·4)	12·0 (9·3 to 14·9)	12·4 (9·6 to 15·4)	1	2·2 (1·7 to 2·7)	21·4 (17·0 to 25·7)
Uruguay	8·0 (6·0 to 10·4)	8·7 (6·6 to 11·3)	9·2 (7·0 to 11·8)	8·5 (6·5 to 11·1)	8·8 (6·7 to 11·3)	9·3 (7·0 to 12·0)	95	1·2 (0·6 to 1·9)	15·5 (7·1 to 25·4)
**Latin America and Caribbean**	**9·1 (6·9 to 11·7)**	**9·5 (7·2 to 12·2)**	**9·3 (7·1 to 11·9)**	**9·2 (7·0 to 11·7)**	**10·1 (7·7 to 13·0)**	**10·3 (7·9 to 13·1)**		**1·2 (0·7 to 1·7)**	**12·7 (7·8 to 17·4)**
Antigua and Barbuda	8·9 (6·4 to 11·8)	9·9 (7·4 to 13·1)	10·1 (7·4 to 13·0)	9·9 (7·3 to 13·0)	10·3 (7·4 to 13·5)	10·5 (7·4 to 13·6)	40	1·6 (-0·3 to 3·3)	18·0 (-2·6 to 42·6)
Bahamas	8·0 (6·0 to 10·5)	8·5 (6·3 to 11·1)	8·3 (6·1 to 10·8)	7·6 (5·6 to 10·0)	8·6 (6·4 to 11·2)	9·0 (6·6 to 11·7)	106	1·0 (0·0 to 2·2)	13·2 (-0·6 to 28·5)
Barbados	8·7 (6·4 to 11·2)	9·5 (7·2 to 12·4)	9·4 (7·0 to 12·2)	9·3 (6·9 to 12·0)	9·3 (6·9 to 12·1)	9·7 (7·3 to 12·7)	70	1·0 (0·0 to 2·1)	12·3 (0·1 to 25·4)
Belize	8·9 (6·6 to 11·5)	9·2 (6·9 to 12·0)	9·2 (6·9 to 11·9)	9·1 (6·7 to 11·7)	9·7 (7·2 to 12·6)	9·9 (7·6 to 12·8)	61	1·1 (0·0 to 2·3)	12·5 (-0·1 to 28·0)
Bermuda	8·2 (6·1 to 10·5)	9·8 (7·3 to 12·7)	10·0 (7·5 to 12·9)	9·9 (7·5 to 12·8)	10·1 (7·6 to 13·0)	10·4 (7·8 to 13·4)	44	2·2 (1·2 to 3·3)	27·2 (15·1 to 39·6)
Bolivia	7·5 (5·6 to 9·8)	7·9 (6·0 to 10·4)	6·5 (4·8 to 8·7)	7·0 (5·1 to 9·1)	8·4 (6·1 to 10·9)	8·6 (6·2 to 11·3)	122	1·2 (0·1 to 2·1)	16·0 (1·1 to 31·3)
Brazil	8·9 (6·7 to 11·5)	9·5 (7·2 to 12·2)	9·8 (7·5 to 12·5)	9·5 (7·3 to 12·1)	10·3 (7·9 to 13·1)	10·5 (8·0 to 13·3)	33	1·7 (1·1 to 2·3)	18·9 (12·7 to 25·6)
Colombia	9·2 (7·0 to 11·8)	10·0 (7·5 to 12·8)	9·6 (7·4 to 12·2)	9·2 (7·0 to 11·8)	10·2 (7·8 to 13·0)	10·6 (8·1 to 13·5)	32	1·3 (0·7 to 2·0)	14·5 (7·3 to 21·8)
Costa Rica	9·7 (7·4 to 12·5)	10·5 (7·9 to 13·4)	10·7 (8·1 to 13·6)	10·5 (8·0 to 13·2)	10·9 (8·2 to 13·8)	10·9 (8·2 to 13·8)	22	1·2 (0·4 to 1·9)	12·0 (4·4 to 19·4)
Cuba	9·0 (6·6 to 11·6)	9·6 (7·2 to 12·5)	9·7 (7·4 to 12·5)	8·6 (6·5 to 11·2)	9·7 (7·0 to 12·7)	9·9 (7·0 to 13·1)	64	0·9 (-0·7 to 2·6)	10·5 (-8·3 to 30·5)
Dominica	9·0 (6·6 to 11·8)	9·4 (7·0 to 12·2)	9·5 (7·2 to 12·3)	9·4 (7·0 to 12·1)	9·7 (7·2 to 12·7)	9·9 (7·4 to 13·1)	63	0·9 (-0·1 to 1·9)	10·2 (-1·2 to 22·1)
Dominican Republic	8·5 (6·3 to 11·0)	9·3 (6·9 to 12·0)	8·6 (6·6 to 11·2)	8·9 (6·7 to 11·6)	8·9 (6·7 to 11·7)	9·0 (6·8 to 12·0)	103	0·6 (-0·5 to 1·7)	7·0 (-5·8 to 21·4)
Ecuador	8·5 (6·4 to 11·1)	9·0 (6·8 to 11·8)	8·5 (6·3 to 10·8)	9·1 (6·8 to 11·6)	9·7 (7·3 to 12·3)	9·7 (7·3 to 12·3)	73	1·1 (0·4 to 1·9)	13·4 (5·1 to 23·3)
El Salvador	9·0 (6·7 to 11·6)	9·5 (7·1 to 12·3)	8·8 (6·5 to 11·4)	8·6 (6·3 to 11·1)	9·3 (6·7 to 11·9)	9·3 (6·8 to 12·0)	93	0·3 (-1·1 to 1·8)	4·1 (-11·3 to 22·3)
Grenada	8·5 (6·2 to 11·2)	9·7 (7·2 to 12·6)	9·7 (7·1 to 12·4)	9·1 (6·7 to 11·9)	10·0 (7·2 to 13·0)	10·3 (7·3 to 13·2)	48	1·8 (0·1 to 3·5)	21·5 (0·8 to 45·9)
Guatemala	8·5 (6·3 to 11·3)	9·8 (7·3 to 12·6)	9·6 (7·4 to 12·4)	9·2 (7·1 to 11·8)	10·2 (7·7 to 13·1)	10·6 (7·7 to 14·1)	31	2·1 (0·4 to 3·8)	24·7 (4·3 to 46·9)
Guyana	8·0 (5·7 to 10·6)	8·1 (5·9 to 10·5)	8·5 (6·0 to 11·2)	8·0 (5·7 to 10·3)	8·4 (5·7 to 11·1)	8·5 (5·6 to 11·4)	127	0·4 (-1·6 to 2·4)	6·1 (-20·4 to 33·4)
Haiti	6·4 (4·4 to 8·6)	3·8 (1·3 to 6·0)	7·6 (5·3 to 9·9)	7·6 (5·2 to 9·9)	7·6 (5·1 to 10·2)	7·5 (5·1 to 10·0)	181	1·1 (-0·9 to 3·1)	19·0 (-13·7 to 59·2)
Honduras	9·2 (7·0 to 11·8)	9·9 (7·6 to 12·6)	9·3 (7·1 to 12·0)	9·0 (6·8 to 11·7)	10·2 (7·7 to 13·2)	10·2 (7·6 to 13·3)	52	1·0 (0·0 to 2·0)	10·9 (-0·3 to 23·8)
Jamaica	9·2 (6·8 to 11·8)	9·7 (7·2 to 12·6)	9·1 (6·4 to 11·8)	8·8 (6·2 to 11·7)	9·3 (6·4 to 12·3)	9·7 (7·0 to 12·7)	75	0·5 (-1·3 to 2·3)	5·5 (-14·0 to 27·9)
Mexico	10·5 (7·9 to 13·5)	10·6 (8·1 to 13·5)	9·3 (7·1 to 11·9)	9·5 (7·2 to 12·2)	10·8 (8·3 to 13·8)	11·1 (8·5 to 14·1)	14	0·6 (0·2 to 1·0)	5·4 (1·4 to 9·8)
Nicaragua	9·3 (6·9 to 12·2)	9·6 (7·3 to 12·4)	8·7 (6·6 to 11·2)	8·8 (6·7 to 11·5)	10·0 (7·5 to 12·8)	10·5 (7·5 to 13·5)	37	1·2 (-0·6 to 2·8)	12·8 (-5·9 to 32·3)
Panama	9·6 (7·3 to 12·3)	9·9 (7·5 to 12·7)	9·7 (7·4 to 12·4)	10·0 (7·6 to 12·8)	10·5 (8·0 to 13·3)	10·5 (7·9 to 13·7)	34	0·9 (0·1 to 1·9)	9·9 (0·7 to 20·3)
Paraguay	9·3 (7·0 to 12·0)	8·8 (6·6 to 11·4)	8·8 (6·7 to 11·3)	7·8 (5·8 to 10·1)	9·1 (6·6 to 11·8)	9·2 (6·7 to 11·9)	97	0·0 (-1·1 to 1·1)	-0·2 (-11·8 to 12·1)
Peru	9·3 (7·0 to 12·2)	9·9 (7·4 to 12·8)	8·2 (6·1 to 10·6)	8·1 (6·1 to 10·5)	9·9 (7·5 to 12·8)	9·7 (7·5 to 12·5)	68	0·4 (-0·5 to 1·3)	4·4 (-4·6 to 14·7)
Puerto Rico	9·2 (7·0 to 11·9)	10·3 (7·8 to 13·0)	10·8 (8·3 to 13·9)	10·9 (8·3 to 13·8)	11·0 (8·4 to 14·0)	11·3 (8·5 to 14·5)	9	2·1 (1·2 to 3·0)	23·0 (14·1 to 33·0)
Saint Kitts and Nevis	8·2 (6·0 to 10·7)	9·1 (6·8 to 11·8)	9·5 (7·0 to 12·3)	9·3 (6·9 to 12·0)	9·7 (7·2 to 12·5)	9·8 (7·3 to 12·8)	65	1·7 (0·5 to 2·7)	20·6 (6·3 to 35·7)
Saint Lucia	8·5 (6·2 to 11·3)	9·6 (7·2 to 12·3)	10·2 (7·5 to 13·3)	9·7 (6·9 to 13·1)	10·4 (7·3 to 13·8)	10·6 (7·5 to 14·2)	30	2·1 (-0·2 to 4·2)	24·9 (-1·8 to 53·1)
Saint Vincent and the Grenadines	8·7 (6·4 to 11·5)	9·5 (7·0 to 12·6)	9·7 (7·0 to 12·7)	9·8 (7·2 to 12·8)	10·0 (7·4 to 13·2)	10·0 (7·1 to 13·3)	57	1·3 (-0·5 to 3·0)	15·3 (-5·8 to 38·9)
Suriname	8·6 (6·4 to 11·1)	9·0 (6·8 to 11·8)	9·2 (7·0 to 11·9)	8·2 (6·1 to 10·6)	9·4 (7·0 to 12·2)	9·7 (7·2 to 12·6)	69	1·1 (0·2 to 2·2)	13·2 (1·7 to 26·4)
Trinidad and Tobago	8·4 (6·3 to 10·9)	9·4 (7·1 to 12·2)	9·4 (7·2 to 12·2)	8·6 (6·5 to 11·1)	9·2 (6·8 to 12·0)	9·7 (7·2 to 12·6)	78	1·2 (0·2 to 2·2)	14·9 (3·0 to 27·4)
Venezuela	8·9 (6·8 to 11·5)	9·3 (7·0 to 12·1)	8·8 (6·5 to 11·6)	8·7 (6·5 to 11·4)	9·0 (6·6 to 11·6)	9·0 (6·6 to 11·7)	104	0·1 (-0·8 to 1·1)	1·3 (-10·1 to 13·0)
Virgin Islands	8·1 (5·9 to 10·7)	9·1 (6·7 to 11·9)	9·6 (7·0 to 12·5)	9·3 (6·9 to 12·3)	9·6 (6·7 to 12·7)	9·6 (6·6 to 12·8)	80	1·5 (-0·5 to 3·5)	19·2 (-6·0 to 48·3)
**North Africa and Middle East**	**7·9 (6·0 to 10·2)**	**8·8 (6·7 to 11·4)**	**9·0 (6·9 to 11·5)**	**9·1 (7·0 to 11·7)**	**9·5 (7·2 to 12·1)**	**9·5 (7·2 to 12·2)**		**1·6 (0·9 to 2·4)**	**20·5 (12·1 to 30·0)**
Afghanistan	10·0 (6·8 to 13·6)	10·7 (7·9 to 14·3)	10·5 (7·6 to 13·7)	10·0 (7·1 to 13·1)	11·6 (8·5 to 15·0)	11·5 (8·3 to 14·9)	7	1·4 (-0·7 to 3·5)	15·3 (-6·1 to 42·0)

Algeria	8·0 (6·0 to 10·4)	8·9 (6·7 to 11·6)	9·1 (7·0 to 11·7)	9·2 (7·0 to 11·9)	9·4 (7·2 to 12·0)	9·4 (7·1 to 12·1)	91	1·3 (0·5 to 2·3)	16·8 (5·8 to 28·9)
Bahrain	8·1 (5·9 to 10·7)	9·1 (6·8 to 12·0)	9·6 (7·2 to 12·6)	9·6 (7·1 to 12·6)	9·9 (7·1 to 13·2)	9·9 (7·3 to 13·2)	60	1·9 (0·3 to 3·3)	23·4 (3·5 to 42·8)
Egypt	7·6 (5·7 to 9·9)	7·9 (6·0 to 10·1)	8·4 (6·3 to 10·6)	8·4 (6·3 to 10·7)	8·6 (6·0 to 11·1)	8·5 (6·1 to 11·4)	125	0·9 (-0·5 to 2·4)	12·1 (-6·7 to 33·5)
Iran	8·5 (6·4 to 11·0)	9·9 (7·5 to 12·8)	9·6 (7·3 to 12·4)	10·0 (7·6 to 12·8)	10·8 (8·3 to 13·7)	11·0 (8·5 to 14·0)	18	2·5 (1·4 to 3·7)	29·5 (17·4 to 42·6)
Iraq	8·5 (6·5 to 11·2)	8·5 (6·3 to 10·9)	8·4 (6·4 to 10·7)	8·9 (6·8 to 11·4)	9·2 (7·1 to 11·8)	9·1 (6·8 to 11·8)	100	0·6 (-0·4 to 1·6)	7·0 (-5·1 to 19·0)
Jordan	7·5 (5·7 to 9·7)	8·6 (6·4 to 11·1)	8·9 (6·8 to 11·3)	8·8 (6·7 to 11·2)	9·6 (7·3 to 12·2)	9·7 (7·2 to 12·5)	71	2·2 (1·2 to 3·2)	29·4 (16·9 to 43·7)
Kuwait	8·3 (6·2 to 10·8)	10·3 (7·8 to 13·3)	10·9 (8·3 to 13·8)	11·1 (8·4 to 14·0)	11·5 (8·7 to 14·6)	11·6 (8·7 to 15·0)	4	3·3 (2·2 to 4·5)	39·6 (27·8 to 53·2)
Lebanon	10·5 (7·6 to 13·8)	11·0 (8·3 to 14·1)	9·1 (6·9 to 11·8)	10·5 (8·0 to 13·5)	11·0 (8·2 to 14·2)	11·3 (8·2 to 14·9)	10	0·7 (-1·2 to 2·8)	7·6 (-11·4 to 29·4)
Libya	8·1 (6·1 to 10·6)	9·1 (6·9 to 11·7)	9·5 (7·1 to 12·5)	9·3 (6·9 to 12·1)	10·1 (7·5 to 13·2)	9·4 (7·0 to 12·3)	89	1·3 (0·3 to 2·4)	16·5 (4·0 to 31·3)
Morocco	7·7 (5·7 to 10·1)	8·5 (6·3 to 11·1)	8·7 (6·5 to 11·2)	9·0 (6·6 to 11·5)	9·0 (6·6 to 11·8)	9·0 (6·6 to 12·0)	108	1·3 (-0·2 to 2·6)	17·2 (-2·2 to 37·7)
Oman	8·0 (5·9 to 10·5)	8·5 (6·4 to 11·1)	8·6 (6·4 to 11·3)	8·6 (6·5 to 11·3)	9·5 (7·0 to 12·2)	9·6 (7·0 to 12·6)	81	1·6 (0·3 to 2·8)	19·8 (3·7 to 38·3)
Palestine	8·4 (6·4 to 10·9)	9·1 (6·9 to 11·8)	9·8 (7·5 to 12·5)	9·6 (7·3 to 12·3)	10·1 (7·6 to 12·9)	8·0 (5·3 to 10·6)	162	-0·4 (-2·2 to 1·2)	-4·7 (-27·2 to 16·2)
Qatar	8·4 (6·2 to 11·1)	9·8 (7·3 to 12·6)	10·7 (8·1 to 13·7)	11·0 (8·2 to 14·1)	11·1 (8·1 to 14·3)	10·9 (8·0 to 14·2)	19	2·6 (1·0 to 4·1)	30·8 (12·7 to 51·4)
Saudi Arabia	8·3 (6·0 to 10·9)	8·8 (6·6 to 11·4)	9·4 (6·9 to 12·0)	9·9 (7·5 to 12·6)	10·1 (7·6 to 13·1)	10·2 (7·6 to 13·2)	53	1·9 (0·5 to 3·3)	23·0 (5·8 to 41·9)
Sudan	6·5 (4·4 to 8·7)	7·7 (5·4 to 10·1)	8·2 (5·7 to 10·8)	8·4 (6·0 to 11·2)	8·5 (5·9 to 11·3)	8·4 (5·6 to 11·3)	138	1·9 (0·0 to 3·7)	30·5 (0·3 to 67·5)
Syria	8·4 (6·3 to 10·9)	9·2 (7·0 to 11·9)	10·7 (8·2 to 13·7)	10·9 (8·3 to 13·9)	11·3 (8·7 to 14·4)	11·2 (8·6 to 14·4)	13	2·8 (1·7 to 4·0)	33·7 (19·9 to 48·6)
Tunisia	8·1 (6·1 to 10·3)	8·6 (6·5 to 11·1)	8·7 (6·6 to 11·1)	8·4 (6·3 to 10·7)	9·5 (7·2 to 12·0)	9·7 (7·3 to 12·4)	76	1·6 (0·8 to 2·4)	20·1 (10·5 to 30·8)
Türkiye	7·7 (5·7 to 10·1)	9·0 (6·8 to 11·5)	8·9 (6·8 to 11·4)	8·9 (6·7 to 11·5)	9·2 (7·0 to 11·8)	9·2 (6·5 to 11·9)	98	1·4 (0·0 to 2·9)	18·8 (-0·6 to 39·9)
United Arab Emirates	8·2 (6·1 to 10·8)	9·5 (7·2 to 12·4)	10·6 (8·0 to 13·5)	10·7 (8·1 to 13·7)	10·9 (8·3 to 14·0)	10·8 (8·0 to 14·0)	25	2·6 (1·4 to 4·0)	31·8 (17·1 to 48·7)
Yemen	7·2 (5·0 to 9·7)	8·3 (6·0 to 10·9)	8·4 (6·0 to 10·9)	8·8 (6·2 to 11·5)	9·4 (6·8 to 12·3)	9·5 (6·7 to 12·4)	88	2·2 (0·3 to 4·1)	31·7 (4·0 to 65·6)
**South Asia**	**7·5 (5·4 to 9·8)**	**8·4 (6·3 to 10·9)**	**8·7 (6·6 to 11·3)**	**8·4 (6·4 to 10·9)**	**9·2 (6·6 to 11·9)**	**9·1 (6·5 to 11·9)**		**1·6 (0·2 to 3·1)**	**22·4 (2·4 to 44·9)**
Bangladesh	7·2 (5·2 to 9·6)	8·0 (5·9 to 10·6)	8·3 (6·1 to 10·9)	8·2 (5·8 to 10·7)	8·8 (6·2 to 11·5)	8·8 (6·2 to 11·6)	111	1·5 (-0·2 to 3·3)	22·2 (-2·2 to 54·9)
Bhutan	6·8 (4·8 to 8·9)	8·2 (5·9 to 10·6)	8·6 (6·1 to 11·2)	8·7 (6·0 to 11·3)	8·7 (6·1 to 11·5)	8·8 (6·1 to 11·6)	110	2·0 (0·1 to 3·8)	29·5 (1·9 to 65·9)
India	7·5 (5·4 to 9·9)	8·4 (6·3 to 10·9)	8·8 (6·6 to 11·5)	8·4 (6·3 to 10·9)	9·2 (6·5 to 11·9)	9·1 (6·4 to 12·0)	99	1·7 (-0·1 to 3·4)	23·1 (-1·5 to 51·6)
Nepal	7·6 (5·3 to 10·1)	8·7 (6·5 to 11·2)	8·8 (6·4 to 11·4)	8·3 (6·0 to 11·0)	9·3 (6·6 to 12·4)	9·3 (6·6 to 12·2)	94	1·7 (-0·3 to 3·7)	23·9 (-4·2 to 58·3)
Pakistan	7·8 (5·5 to 10·3)	8·6 (6·4 to 11·5)	8·7 (6·2 to 11·7)	8·8 (6·1 to 11·8)	9·6 (6·9 to 12·6)	9·5 (6·8 to 12·6)	85	1·7 (-0·1 to 3·8)	22·8 (-1·5 to 54·0)
**Southeast Asia, East Asia, and Oceania**	**7·2 (5·3 to 9·2)**	**8·3 (6·3 to 10·7)**	**9·0 (6·9 to 11·5)**	**8·7 (6·7 to 11·1)**	**8·9 (6·6 to 11·4)**	**9·0 (6·4 to 11·7)**		**1·8 (0·6 to 3·2)**	**25·6 (8·6 to 43·8)**
American Samoa	7·7 (5·5 to 10·3)	8·0 (5·6 to 10·5)	8·0 (5·8 to 10·6)	7·5 (5·1 to 10·0)	8·1 (5·5 to 11·1)	8·3 (5·6 to 11·3)	140	0·7 (-1·1 to 2·3)	9·3 (-14·7 to 33·4)
Cambodia	6·8 (4·6 to 9·3)	7·7 (5·5 to 10·5)	8·2 (5·8 to 10·9)	8·1 (5·8 to 10·9)	8·3 (5·8 to 11·1)	8·3 (5·8 to 11·1)	143	1·5 (-0·5 to 3·6)	23·7 (-6·5 to 65·9)
China	7·1 (5·2 to 9·2)	8·7 (6·5 to 11·2)	9·6 (7·4 to 12·2)	9·4 (7·2 to 12·1)	9·4 (6·8 to 12·1)	9·5 (6·6 to 12·6)	87	2·3 (0·6 to 4·1)	33·2 (9·2 to 58·9)
Cook Islands	7·2 (5·4 to 9·6)	8·1 (5·9 to 10·6)	8·2 (6·1 to 10·7)	8·0 (5·8 to 10·3)	8·4 (6·0 to 10·9)	8·4 (6·0 to 11·2)	129	1·2 (0·1 to 2·3)	16·8 (1·7 to 32·5)
Federated States of Micronesia	6·0 (4·5 to 7·8)	6·9 (5·2 to 9·0)	7·4 (5·6 to 9·8)	7·1 (5·3 to 9·5)	7·6 (5·6 to 10·0)	7·8 (5·7 to 10·3)	174	1·7 (0·7 to 2·8)	29·3 (12·2 to 48·9)
Fiji	7·4 (5·5 to 9·7)	7·4 (5·5 to 9·6)	7·9 (5·8 to 10·1)	7·7 (5·7 to 9·8)	8·1 (5·8 to 10·5)	8·2 (6·0 to 10·5)	147	0·7 (-0·2 to 1·8)	10·2 (-3·5 to 24·8)
Guam	7·6 (5·7 to 9·8)	8·2 (6·1 to 10·7)	8·4 (6·2 to 10·8)	8·2 (6·1 to 10·6)	8·3 (6·0 to 10·8)	8·4 (6·1 to 11·2)	131	0·8 (-0·1 to 1·9)	10·9 (-1·8 to 25·2)
Indonesia	7·2 (5·2 to 9·6)	7·4 (5·2 to 9·7)	7·5 (5·4 to 9·9)	7·0 (4·7 to 9·4)	7·9 (5·3 to 10·6)	7·9 (5·2 to 10·7)	168	0·6 (-1·2 to 2·3)	9·4 (-17·0 to 36·6)
Kiribati	7·8 (5·6 to 10·2)	8·0 (5·9 to 10·4)	8·4 (6·2 to 10·9)	8·1 (6·0 to 10·6)	8·6 (6·3 to 11·3)	8·7 (6·3 to 11·5)	116	0·9 (-0·3 to 2·2)	12·2 (-3·1 to 28·8)
Laos	5·2 (3·3 to 7·2)	6·2 (4·2 to 8·2)	6·5 (4·5 to 8·7)	6·5 (4·5 to 8·8)	6·6 (4·3 to 9·1)	6·6 (4·2 to 9·1)	202	1·4 (-0·9 to 3·7)	29·2 (-15·4 to 88·7)
Malaysia	7·7 (5·8 to 9·9)	8·1 (5·8 to 10·5)	8·4 (6·4 to 10·6)	7·8 (5·9 to 9·8)	8·2 (6·2 to 10·5)	8·6 (6·4 to 10·9)	123	0·9 (0·0 to 1·6)	11·4 (0·5 to 23·3)
Maldives	7·3 (5·5 to 9·4)	7·8 (5·8 to 10·1)	8·0 (6·0 to 10·4)	7·9 (5·8 to 10·2)	8·0 (6·0 to 10·4)	8·0 (6·0 to 10·4)	157	0·8 (-0·1 to 1·6)	11·2 (-2·1 to 25·5)
Marshall Islands	6·4 (4·7 to 8·4)	6·9 (5·0 to 9·0)	7·1 (5·2 to 9·3)	6·8 (5·0 to 9·0)	7·2 (5·3 to 9·5)	7·3 (5·3 to 9·7)	188	0·9 (-0·1 to 1·9)	14·7 (-1·3 to 33·1)
Mauritius	7·3 (5·4 to 9·6)	8·5 (6·4 to 11·0)	8·8 (6·7 to 11·4)	8·5 (6·4 to 10·8)	8·7 (6·5 to 11·1)	8·7 (6·4 to 11·2)	115	1·4 (0·5 to 2·4)	19·4 (6·9 to 34·1)
Myanmar	5·9 (4·2 to 7·9)	6·3 (4·4 to 8·5)	6·8 (4·6 to 9·2)	6·4 (4·2 to 8·8)	6·9 (4·1 to 9·5)	6·8 (4·2 to 9·5)	196	0·9 (-1·1 to 3·0)	17·0 (-20·1 to 58·6)
Nauru	5·7 (4·2 to 7·5)	5·9 (4·4 to 7·8)	6·1 (4·4 to 8·2)	5·7 (4·0 to 7·6)	6·2 (4·5 to 8·3)	6·3 (4·4 to 8·6)	204	0·6 (-0·6 to 1·8)	11·6 (-10·7 to 32·7)
Niue	7·3 (5·3 to 9·4)	8·1 (6·0 to 10·5)	8·3 (6·3 to 11·0)	7·9 (6·0 to 10·4)	8·3 (6·2 to 10·8)	8·4 (6·3 to 11·0)	136	1·1 (0·2 to 2·1)	16·1 (3·1 to 31·9)
North Korea	7·2 (5·1 to 9·6)	7·7 (5·6 to 10·2)	8·2 (6·0 to 10·9)	8·3 (5·9 to 11·0)	8·3 (6·0 to 11·0)	8·4 (5·8 to 11·2)	130	1·2 (-0·6 to 2·9)	17·0 (-8·1 to 43·5)
Northern Mariana Islands	7·0 (5·2 to 9·2)	7·6 (5·7 to 9·8)	7·7 (5·8 to 10·2)	7·5 (5·6 to 9·9)	8·0 (5·9 to 10·5)	8·1 (6·0 to 10·7)	149	1·1 (0·1 to 2·1)	16·0 (0·9 to 30·9)
Palau	7·1 (5·3 to 9·4)	7·7 (5·8 to 10·1)	8·0 (5·8 to 10·5)	7·7 (5·6 to 10·2)	8·2 (5·9 to 10·9)	8·4 (6·0 to 11·1)	135	1·3 (0·2 to 2·3)	17·9 (3·3 to 34·2)
Papua New Guinea	7·1 (5·0 to 9·6)	7·3 (5·3 to 9·6)	7·8 (5·4 to 10·3)	7·7 (5·4 to 10·3)	7·9 (5·4 to 10·4)	7·9 (5·2 to 10·7)	167	0·8 (-1·0 to 2·7)	12·0 (-15·5 to 41·7)
Philippines	7·6 (5·7 to 9·8)	7·1 (5·4 to 9·2)	7·3 (5·5 to 9·3)	6·7 (5·0 to 8·5)	7·3 (5·5 to 9·3)	7·6 (5·7 to 9·7)	178	0·0 (-0·6 to 0·6)	0·4 (-8·0 to 8·8)
Samoa	8·0 (6·0 to 10·4)	8·2 (6·2 to 10·6)	8·4 (6·3 to 10·9)	8·2 (6·1 to 10·6)	8·6 (6·3 to 11·2)	8·6 (6·2 to 11·4)	121	0·6 (-0·5 to 1·8)	8·0 (-6·3 to 22·7)
Seychelles	6·9 (5·1 to 9·0)	7·3 (5·4 to 9·6)	8·3 (6·2 to 10·7)	7·7 (5·8 to 10·0)	8·3 (6·1 to 10·9)	8·3 (6·2 to 10·8)	144	1·4 (0·4 to 2·4)	20·4 (6·2 to 35·5)
Solomon Islands	5·8 (4·1 to 7·6)	6·3 (4·6 to 8·4)	6·3 (4·6 to 8·5)	6·3 (4·6 to 8·5)	6·5 (4·7 to 8·7)	6·5 (4·6 to 8·7)	203	0·8 (-0·2 to 1·8)	13·9 (-4·4 to 33·7)
Sri Lanka	7·5 (5·6 to 9·6)	8·1 (6·1 to 10·4)	8·8 (6·7 to 11·3)	8·4 (6·5 to 10·8)	8·5 (6·4 to 10·9)	8·5 (6·4 to 11·0)	124	1·1 (0·3 to 1·8)	14·3 (4·1 to 25·5)

Taiwan*	8·2 (6·2 to 10·5)	9·3 (7·1 to 12·0)	10·1 (7·7 to 12·8)	10·1 (7·7 to 12·8)	9·9 (7·5 to 12·6)	10·1 (7·7 to 12·8)	55	2·0 (1·4 to 2·6)	24·1 (17·9 to 30·0)
Thailand	8·3 (6·2 to 10·6)	8·4 (6·4 to 10·6)	9·0 (6·9 to 11·4)	8·7 (6·6 to 11·1)	8·7 (6·6 to 11·1)	9·0 (6·9 to 11·5)	105	0·7 (0·2 to 1·2)	8·1 (2·4 to 14·7)
Timor-Leste	6·8 (4·7 to 8·9)	7·9 (5·7 to 10·3)	7·9 (5·7 to 10·3)	7·9 (5·4 to 10·4)	8·1 (5·7 to 11·0)	8·1 (5·5 to 10·9)	154	1·3 (-0·7 to 3·5)	20·1 (-9·3 to 58·1)
Tokelau	8·2 (6·1 to 10·8)	9·0 (6·7 to 11·7)	9·5 (7·1 to 12·4)	9·1 (6·8 to 12·0)	9·4 (7·0 to 12·5)	9·6 (7·0 to 12·8)	83	1·3 (0·2 to 2·4)	16·4 (2·7 to 29·9)
Tonga	7·6 (5·7 to 9·9)	7·9 (5·9 to 10·3)	8·2 (6·1 to 10·9)	7·8 (5·7 to 10·5)	8·3 (6·0 to 11·1)	8·4 (6·0 to 11·4)	132	0·9 (-0·3 to 1·9)	11·5 (-3·3 to 26·4)
Tuvalu	6·2 (4·5 to 8·2)	6·7 (5·0 to 8·8)	6·4 (4·7 to 8·7)	6·2 (4·5 to 8·3)	6·6 (4·8 to 8·8)	6·6 (4·7 to 8·8)	201	0·4 (-0·6 to 1·5)	7·2 (-10·0 to 25·3)
Vanuatu	7·1 (5·3 to 9·2)	7·4 (5·5 to 9·6)	7·6 (5·6 to 10·0)	7·5 (5·6 to 9·9)	7·8 (5·8 to 10·2)	7·9 (5·7 to 10·3)	169	0·8 (-0·4 to 2·0)	11·5 (-6·5 to 30·4)
Viet Nam	7·3 (5·2 to 9·5)	7·5 (5·4 to 9·8)	7·8 (5·7 to 10·2)	7·6 (5·7 to 9·9)	7·8 (5·5 to 10·4)	7·9 (5·4 to 10·7)	166	0·6 (-1·1 to 2·5)	8·7 (-15·0 to 38·9)
**Sub-Saharan Africa**	**6·2 (4·6 to 8·1)**	**7·0 (5·3 to 9·0)**	**7·3 (5·5 to 9·4)**	**7·2 (5·5 to 9·3)**	**7·7 (5·8 to 9·9)**	**7·7 (5·8 to 9·9)**		**1·5 (0·7 to 2·2)**	**23·7 (12·8 to 36·7)**
Angola	5·8 (3·9 to 7·9)	7·1 (5·1 to 9·5)	7·4 (5·4 to 9·9)	7·3 (5·3 to 9·9)	7·8 (5·4 to 10·7)	7·8 (5·1 to 10·5)	172	2·0 (-0·3 to 4·0)	36·3 (-4·9 to 87·3)
Benin	6·2 (4·4 to 8·4)	7·0 (4·9 to 9·1)	7·3 (5·2 to 9·6)	7·4 (5·1 to 9·8)	7·5 (5·2 to 10·1)	7·5 (5·1 to 10·2)	180	1·3 (-0·6 to 3·1)	21·5 (-8·8 to 59·3)
Botswana	7·5 (5·6 to 9·8)	8·4 (6·1 to 10·9)	8·6 (6·3 to 10·9)	8·0 (5·9 to 10·2)	8·7 (6·4 to 11·2)	8·8 (6·4 to 11·4)	113	1·2 (0·1 to 2·4)	16·5 (1·0 to 32·7)
Burkina Faso	6·3 (4·3 to 8·7)	7·4 (5·3 to 9·7)	7·9 (6·0 to 10·3)	8·2 (6·0 to 10·9)	8·3 (5·9 to 11·1)	8·1 (5·7 to 10·8)	150	1·9 (-0·2 to 3·8)	31·2 (-2·9 to 72·9)
Burundi	5·0 (3·2 to 7·0)	7·1 (5·2 to 9·5)	7·3 (5·2 to 9·5)	7·1 (5·1 to 9·3)	7·5 (5·2 to 9·8)	7·5 (5·0 to 10·0)	185	2·4 (0·3 to 4·4)	52·2 (4·8 to 121·3)
Cabo Verde	8·0 (5·9 to 10·4)	8·4 (6·1 to 10·9)	8·3 (6·1 to 10·9)	8·4 (6·0 to 11·1)	8·9 (6·2 to 11·8)	9·0 (6·4 to 12·1)	107	1·0 (-0·7 to 2·9)	13·1 (-8·7 to 42·2)
Cameroon	6·7 (4·6 to 9·1)	6·4 (4·6 to 8·5)	6·9 (4·9 to 9·0)	6·7 (4·7 to 8·8)	7·3 (5·0 to 9·6)	7·3 (4·9 to 9·6)	187	0·6 (-1·3 to 2·5)	9·7 (-17·8 to 42·9)
Central African Republic	6·6 (4·5 to 9·0)	6·9 (4·9 to 9·1)	7·7 (5·4 to 10·1)	7·9 (5·5 to 10·6)	8·0 (5·5 to 10·5)	8·0 (5·6 to 10·5)	159	1·4 (-0·5 to 3·4)	22·6 (-6·5 to 60·9)
Chad	5·5 (3·7 to 7·6)	6·3 (4·4 to 8·2)	6·6 (4·6 to 8·6)	7·0 (5·0 to 9·3)	6·8 (4·8 to 9·0)	6·8 (4·7 to 9·0)	197	1·3 (-0·6 to 3·2)	25·2 (-8·9 to 69·5)
Comoros	8·1 (5·8 to 10·8)	9·1 (6·6 to 12·1)	8·9 (6·6 to 11·7)	8·3 (6·0 to 11·0)	9·4 (6·6 to 12·5)	9·5 (6·6 to 12·5)	84	1·4 (-0·5 to 3·2)	18·0 (-6·4 to 45·4)
Congo (Brazzaville)	6·6 (4·6 to 8·8)	7·6 (5·5 to 10·0)	7·8 (5·5 to 10·3)	7·9 (5·5 to 10·4)	8·2 (5·7 to 10·8)	8·1 (5·5 to 11·0)	156	1·5 (-0·5 to 3·6)	23·6 (-7·1 to 61·9)
Côte d'Ivoire	7·1 (5·2 to 9·3)	7·3 (5·2 to 9·6)	7·4 (5·3 to 9·9)	7·6 (5·3 to 10·1)	7·7 (5·4 to 10·3)	7·6 (5·1 to 10·3)	176	0·5 (-1·4 to 2·4)	8·4 (-19·9 to 38·6)
DR Congo	6·8 (4·6 to 9·2)	7·3 (5·2 to 9·6)	7·3 (5·2 to 9·6)	7·6 (5·3 to 10·0)	7·7 (5·5 to 10·1)	7·7 (5·2 to 10·2)	175	0·9 (-1·0 to 2·9)	14·9 (-13·4 to 51·7)
Djibouti	6·7 (4·6 to 9·1)	7·3 (5·2 to 9·7)	7·6 (5·3 to 10·0)	7·3 (5·0 to 9·8)	7·9 (5·4 to 10·6)	7·9 (5·3 to 10·8)	165	1·2 (-0·7 to 3·1)	19·4 (-10·9 to 54·9)
Equatorial Guinea	5·9 (4·0 to 8·0)	6·8 (4·8 to 9·0)	6·9 (4·8 to 9·0)	7·0 (4·8 to 9·5)	7·2 (4·7 to 9·7)	7·1 (4·5 to 9·6)	192	1·2 (-0·9 to 3·3)	21·8 (-14·1 to 64·5)
Eritrea	5·8 (3·2 to 8·4)	7·9 (5·7 to 10·5)	8·0 (5·9 to 10·5)	8·0 (5·8 to 10·8)	7·6 (5·1 to 10·1)	8·0 (5·4 to 10·7)	161	2·1 (-0·3 to 4·7)	41·6 (-4·1 to 117·5)
Eswatini	5·4 (3·7 to 7·3)	5·3 (3·5 to 7·3)	7·0 (4·7 to 9·2)	6·9 (4·4 to 9·3)	7·5 (5·0 to 10·2)	7·5 (4·8 to 10·2)	182	2·1 (-0·2 to 4·4)	40·9 (-3·0 to 95·0)
Ethiopia	5·0 (3·2 to 7·1)	6·9 (5·1 to 9·1)	7·1 (5·2 to 9·6)	6·9 (4·8 to 9·2)	7·5 (5·2 to 10·1)	7·5 (5·0 to 10·0)	184	2·5 (0·3 to 4·4)	51·8 (6·4 to 113·2)
Gabon	7·4 (5·2 to 9·8)	8·0 (5·7 to 10·8)	8·5 (6·1 to 11·1)	8·7 (6·4 to 11·5)	8·8 (6·2 to 11·6)	8·7 (5·9 to 11·5)	114	1·2 (-0·7 to 3·3)	17·6 (-9·1 to 50·7)
Ghana	6·5 (4·4 to 8·8)	6·8 (4·9 to 9·0)	7·0 (4·9 to 9·2)	7·0 (4·9 to 9·3)	7·2 (4·8 to 9·6)	7·2 (4·7 to 10·0)	190	0·7 (-1·2 to 2·7)	12·1 (-18·7 to 52·2)
Guinea	5·7 (3·8 to 8·0)	6·7 (4·8 to 8·9)	7·2 (5·2 to 9·3)	7·3 (5·2 to 9·6)	7·4 (5·1 to 9·8)	7·4 (4·8 to 9·8)	186	1·6 (-0·5 to 3·7)	30·3 (-8·6 to 80·9)
Guinea-Bissau	6·0 (4·0 to 8·4)	6·8 (4·8 to 9·0)	6·9 (4·9 to 9·2)	7·3 (5·0 to 9·8)	7·3 (5·1 to 9·8)	7·3 (5·0 to 9·9)	189	1·2 (-0·8 to 3·3)	22·4 (-11·8 to 69·4)
Kenya	6·8 (4·8 to 8·9)	7·2 (5·3 to 9·4)	7·4 (5·3 to 9·8)	7·3 (5·1 to 9·8)	8·3 (5·8 to 11·0)	8·2 (5·6 to 11·1)	146	1·5 (-0·5 to 3·4)	22·4 (-7·5 to 55·5)
Lesotho	6·1 (4·2 to 8·1)	6·1 (4·2 to 8·1)	6·8 (4·6 to 9·2)	6·7 (4·5 to 9·1)	7·0 (4·6 to 9·5)	7·0 (4·5 to 9·5)	193	0·9 (-1·3 to 2·8)	15·3 (-20·7 to 55·1)
Liberia	6·0 (4·0 to 8·1)	7·8 (5·6 to 10·5)	7·1 (5·0 to 9·6)	6·7 (4·7 to 9·1)	7·8 (5·4 to 10·3)	7·8 (5·3 to 10·5)	173	1·8 (-0·3 to 3·8)	31·5 (-3·8 to 77·6)
Madagascar	5·7 (3·8 to 7·9)	6·4 (4·5 to 8·7)	6·6 (4·5 to 8·7)	6·5 (4·3 to 8·7)	6·7 (4·4 to 9·0)	6·7 (4·5 to 8·9)	199	1·0 (-0·8 to 2·8)	19·2 (-12·4 to 64·3)
Malawi	4·9 (3·2 to 6·7)	6·2 (4·3 to 8·4)	6·5 (4·6 to 8·7)	5·4 (3·7 to 7·5)	6·6 (4·4 to 9·2)	6·7 (4·5 to 9·1)	200	1·8 (-0·2 to 3·8)	40·6 (-3·9 to 99·2)
Mali	6·3 (4·2 to 8·5)	7·5 (5·4 to 9·9)	7·5 (5·4 to 10·0)	7·6 (5·4 to 10·0)	8·0 (5·8 to 10·6)	8·1 (5·6 to 11·0)	151	1·9 (-0·3 to 3·9)	32·0 (-4·6 to 77·2)
Mauritania	6·9 (4·9 to 9·2)	7·4 (5·3 to 9·8)	7·4 (5·4 to 9·8)	7·5 (5·3 to 10·1)	7·7 (5·4 to 10·3)	7·8 (5·5 to 10·5)	171	1·0 (-0·7 to 2·8)	14·6 (-10·5 to 43·4)
Mozambique	6·9 (4·7 to 9·2)	7·5 (5·4 to 9·9)	7·7 (5·5 to 10·1)	7·2 (5·1 to 9·5)	8·1 (5·6 to 10·9)	8·3 (5·6 to 11·2)	142	1·3 (-0·8 to 3·7)	20·5 (-10·5 to 61·7)
Namibia	6·6 (5·0 to 8·6)	7·1 (5·4 to 9·2)	7·4 (5·6 to 9·6)	6·2 (4·6 to 8·0)	7·7 (5·7 to 10·1)	8·0 (5·8 to 10·6)	158	1·4 (0·2 to 2·7)	22·3 (2·5 to 42·4)
Niger	5·0 (3·1 to 7·2)	6·2 (4·3 to 8·4)	6·4 (4·5 to 8·5)	6·6 (4·6 to 8·8)	6·7 (4·6 to 9·1)	6·7 (4·3 to 9·1)	198	1·7 (-0·5 to 3·8)	38·1 (-9·0 to 104·2)
Nigeria	6·5 (4·4 to 8·9)	7·1 (4·9 to 9·6)	7·6 (5·4 to 10·1)	7·8 (5·5 to 10·3)	8·0 (5·7 to 10·7)	8·0 (5·5 to 10·7)	163	1·5 (-0·6 to 3·5)	24·0 (-9·0 to 68·6)
Rwanda	4·6 (2·7 to 6·5)	7·1 (5·1 to 9·4)	7·1 (5·0 to 9·3)	6·9 (4·8 to 9·1)	7·2 (4·8 to 9·7)	7·1 (4·9 to 9·5)	191	2·6 (0·6 to 4·7)	59·7 (11·3 to 134·5)
Senegal	6·8 (4·8 to 9·3)	8·0 (5·8 to 10·5)	8·0 (5·9 to 10·5)	7·9 (5·6 to 10·4)	8·6 (5·9 to 11·3)	8·5 (5·8 to 11·2)	126	1·6 (-0·3 to 3·5)	25·0 (-5·0 to 59·8)
Sierra Leone	5·6 (3·5 to 7·6)	6·3 (4·3 to 8·3)	6·6 (4·6 to 8·9)	6·9 (4·7 to 9·1)	6·9 (4·6 to 9·4)	6·9 (4·5 to 9·3)	194	1·3 (-0·7 to 3·5)	26·5 (-11·0 to 84·1)
Somalia	6·0 (3·8 to 8·2)	5·8 (3·8 to 8·0)	7·5 (5·4 to 9·9)	6·9 (4·7 to 9·4)	7·9 (5·4 to 10·6)	7·9 (5·4 to 10·6)	170	1·9 (0·0 to 3·9)	33·4 (-0·5 to 78·4)
South Africa	7·9 (5·5 to 10·5)	6·7 (4·9 to 8·7)	7·8 (5·9 to 10·0)	7·4 (5·5 to 9·4)	8·3 (6·2 to 10·6)	8·4 (6·2 to 10·7)	134	0·5 (-0·8 to 1·9)	7·4 (-9·5 to 29·7)
South Sudan	6·0 (4·1 to 8·4)	6·7 (4·7 to 9·1)	6·8 (4·9 to 9·1)	7·0 (5·0 to 9·5)	7·0 (4·8 to 9·6)	6·9 (4·7 to 9·4)	195	0·9 (-1·1 to 2·9)	16·4 (-16·6 to 60·5)
São Tomé and Príncipe	7·1 (5·0 to 9·6)	7·2 (5·1 to 9·5)	7·3 (5·4 to 9·6)	7·5 (5·4 to 10·0)	7·9 (5·6 to 10·5)	8·1 (5·5 to 10·7)	153	1·0 (-0·9 to 2·8)	14·7 (-11·6 to 46·1)
Tanzania	6·9 (4·9 to 9·1)	7·5 (5·3 to 10·0)	7·9 (5·7 to 10·5)	7·6 (5·5 to 10·2)	8·3 (5·8 to 11·0)	8·3 (5·9 to 11·0)	141	1·4 (-0·6 to 3·3)	21·1 (-7·6 to 60·6)
The Gambia	7·1 (5·0 to 9·4)	7·7 (5·6 to 10·3)	7·4 (5·4 to 9·9)	7·5 (5·3 to 10·0)	8·1 (5·6 to 10·8)	8·2 (5·8 to 11·0)	148	1·1 (-0·7 to 2·9)	16·0 (-10·0 to 49·6)
Togo	6·6 (4·6 to 8·7)	6·9 (4·9 to 9·1)	7·3 (5·3 to 9·6)	7·4 (5·3 to 9·7)	7·5 (5·1 to 10·1)	7·6 (4·9 to 10·1)	177	1·0 (-0·8 to 2·9)	16·5 (-12·2 to 52·9)
Uganda	5·7 (3·8 to 7·7)	6·7 (4·7 to 8·9)	7·1 (5·1 to 9·7)	6·6 (4·5 to 8·9)	7·4 (5·0 to 10·0)	7·5 (5·0 to 10·3)	183	1·8 (-0·2 to 3·7)	33·5 (-4·0 to 79·1)

Zambia	5·8<br>(3·9 to 7·7)	6·7<br>(4·6 to 9·1)	7·0<br>(4·9 to 9·4)	6·4<br>(4·3 to 8·6)	7·6<br>(5·1 to 10·4)	7·6<br>(4·9 to 10·2)	179	1·8<br>(-0·3 to 3·8)	32·7<br>(-4·9 to 81·4)
Zimbabwe	7·4<br>(5·3 to 9·8)	6·7<br>(4·8 to 8·8)	7·5<br>(5·4 to 10·0)	6·8<br>(4·8 to 9·2)	8·0<br>(5·5 to 10·6)	8·1<br>(5·3 to 10·7)	155	0·6<br>(-1·3 to 2·7)	9·3<br>(-18·0 to 40·6)
**Low SDI**	**6·7<br>(4·9 to 8·7)**	**7·5<br>(5·8 to 9·8)**	**7·9<br>(6·0 to 10·2)**	**7·7<br>(5·9 to 9·9)**	**8·3<br>(6·1 to 10·6)**	**8·3<br>(6·2 to 10·7)**		**1·6<br>(0·8 to 2·4)**	**24·1<br>(12·2 to 37·4)**
**Low-middle SDI**	**7·4<br>(5·4 to 9·7)**	**8·1<br>(6·2 to 10·5)**	**8·4<br>(6·3 to 10·9)**	**8·1<br>(6·1 to 10·6)**	**8·8<br>(6·6 to 11·3)**	**8·8<br>(6·5 to 11·4)**		**1·4<br>(0·2 to 2·5)**	**18·9<br>(3·5 to 36·3)**
**Middle SDI**	**7·6<br>(5·6 to 9·7)**	**8·0<br>(6·0 to 10·2)**	**8·3<br>(6·2 to 10·5)**	**8·0<br>(6·1 to 10·3)**	**8·5<br>(6·4 to 10·9)**	**8·6<br>(6·4 to 11·0)**		**1·0<br>(0·3 to 1·7)**	**13·2<br>(3·7 to 22·7)**
**High-middle SDI**	**7·6<br>(5·7 to 9·8)**	**8·7<br>(6·6 to 11·1)**	**9·2<br>(7·0 to 11·7)**	**9·0<br>(6·9 to 11·5)**	**9·5<br>(7·1 to 12·1)**	**9·6<br>(7·1 to 12·2)**		**1·9<br>(1·0 to 3·0)**	**25·8<br>(14·3 to 39·7)**
**High SDI**	**8·4<br>(6·4 to 10·7)**	**9·5<br>(7·2 to 12·1)**	**9·9<br>(7·7 to 12·6)**	**9·9<br>(7·6 to 12·5)**	**10·0<br>(7·7 to 12·7)**	**10·2<br>(7·7 to 12·9)**		**1·9<br>(1·2 to 2·6)**	**22·3<br>(16·2 to 29·1)**

*United Nations convention recognises Taiwan as a province of China

Table S7: Female life expectancy, 1990, 2010, 2020, 2021, 2022, and 2023, and absolute and relative changes, 1990–2023, globally, by SDI quintile, and for GBD super-regions and countries and territories

Location	1990	2010	2020	2021	2022	2023	Rank (2023)	Absolute Change (1990-2023)	Relative Change, % (1990-2023)
**Global**	**67·3 (67·1 to 67·5)**	**73·7 (73·5 to 73·8)**	**75·7 (75·6 to 75·7)**	**74·7 (74·6 to 74·8)**	**76·0 (75·7 to 76·2)**	**76·3 (76·0 to 76·6)**		**9·0 (8·7 to 9·4)**	**13·4 (12·8 to 14·0)**
**Central Europe, Eastern Europe, and Central Asia**	**73·8 (73·7 to 73·8)**	**75·9 (75·9 to 76·0)**	**77·0 (77·0 to 77·0)**	**75·5 (75·5 to 75·6)**	**77·9 (77·7 to 78·0)**	**78·8 (78·7 to 79·0)**		**5·1 (4·9 to 5·2)**	**6·9 (6·7 to 7·1)**
Albania	74·9 (74·4 to 75·5)	80·5 (79·9 to 81·0)	80·0 (79·8 to 80·2)	79·5 (79·2 to 79·8)	81·3 (80·7 to 81·9)	82·1 (81·4 to 82·7)	44	7·1 (6·3 to 7·9)	9·5 (8·3 to 10·6)
Armenia	73·7 (73·2 to 74·2)	77·3 (76·9 to 77·7)	76·7 (76·4 to 77·0)	78·0 (77·8 to 78·3)	81·0 (80·4 to 81·6)	82·6 (82·0 to 83·1)	40	8·8 (8·0 to 9·6)	12·0 (10·8 to 13·0)
Azerbaijan	70·1 (69·6 to 70·6)	74·1 (73·7 to 74·5)	73·6 (73·3 to 73·8)	73·3 (73·0 to 73·5)	75·7 (75·2 to 76·2)	75·4 (74·7 to 76·1)	118	5·3 (4·5 to 6·2)	7·6 (6·4 to 8·8)
Belarus	75·6 (75·4 to 75·7)	76·5 (76·4 to 76·7)	79·0 (78·7 to 79·3)	78·5 (78·1 to 79·0)	78·4 (77·9 to 79·0)	79·2 (78·6 to 79·7)	79	3·6 (3·0 to 4·2)	4·8 (4·0 to 5·6)
Bosnia and Herzegovina	75·3 (75·0 to 75·6)	78·6 (78·3 to 78·8)	78·6 (78·5 to 78·8)	77·2 (77·0 to 77·3)	79·1 (78·8 to 79·5)	80·1 (79·4 to 80·8)	65	4·8 (4·0 to 5·5)	6·3 (5·3 to 7·3)
Bulgaria	74·7 (74·6 to 74·9)	77·6 (77·4 to 77·7)	77·9 (77·8 to 77·9)	75·8 (75·7 to 75·9)	78·2 (77·9 to 78·4)	79·5 (79·3 to 79·7)	71	4·8 (4·5 to 5·1)	6·4 (6·0 to 6·8)
Croatia	76·2 (76·0 to 76·3)	79·8 (79·7 to 80·0)	80·7 (80·6 to 80·9)	80·1 (80·0 to 80·3)	80·7 (80·4 to 80·9)	81·7 (81·4 to 81·9)	47	5·5 (5·2 to 5·8)	7·2 (6·9 to 7·6)
Czechia	75·4 (75·3 to 75·5)	80·7 (80·6 to 80·8)	81·4 (81·3 to 81·5)	80·6 (80·6 to 80·7)	82·0 (81·9 to 82·2)	82·8 (82·6 to 82·9)	37	7·4 (7·2 to 7·6)	9·8 (9·6 to 10·1)
Estonia	74·9 (74·7 to 75·1)	80·3 (80·1 to 80·4)	82·6 (82·4 to 82·7)	81·5 (81·4 to 81·6)	82·1 (81·8 to 82·4)	82·9 (82·6 to 83·2)	36	8·0 (7·7 to 8·4)	10·7 (10·2 to 11·2)
Georgia	73·1 (72·7 to 73·5)	76·4 (75·9 to 76·8)	77·8 (77·6 to 78·0)	75·5 (75·3 to 75·8)	78·1 (77·6 to 78·6)	79·4 (78·9 to 79·9)	76	6·3 (5·7 to 6·9)	8·6 (7·7 to 9·5)
Hungary	73·8 (73·7 to 73·9)	78·4 (78·2 to 78·5)	79·0 (78·9 to 79·1)	77·8 (77·7 to 77·8)	79·3 (79·2 to 79·5)	80·1 (79·9 to 80·2)	66	6·3 (6·0 to 6·5)	8·5 (8·2 to 8·8)
Kazakhstan	72·2 (71·9 to 72·5)	73·6 (73·2 to 73·9)	75·8 (75·6 to 75·9)	74·3 (74·2 to 74·4)	78·0 (77·7 to 78·4)	78·7 (78·3 to 79·1)	87	6·5 (6·0 to 7·0)	9·0 (8·3 to 9·8)
Kyrgyzstan	70·8 (70·3 to 71·3)	73·5 (73·0 to 73·9)	74·9 (74·7 to 75·1)	74·9 (74·7 to 75·2)	77·3 (76·7 to 77·9)	77·8 (77·2 to 78·5)	99	7·0 (6·3 to 7·9)	10·0 (8·9 to 11·1)
Latvia	74·8 (74·6 to 75·0)	78·1 (77·9 to 78·3)	80·1 (80·0 to 80·3)	78·5 (78·3 to 78·6)	79·7 (79·4 to 79·9)	80·6 (80·3 to 80·9)	54	5·8 (5·4 to 6·2)	7·8 (7·3 to 8·3)
Lithuania	75·8 (75·6 to 75·9)	78·4 (78·2 to 78·6)	80·1 (80·0 to 80·2)	78·9 (78·8 to 79·0)	80·0 (79·8 to 80·2)	81·2 (80·9 to 81·5)	51	5·4 (5·1 to 5·8)	7·2 (6·8 to 7·7)
Moldova	71·5 (71·1 to 71·9)	74·7 (74·2 to 75·1)	77·0 (76·8 to 77·3)	76·0 (75·8 to 76·3)	79·5 (78·9 to 80·1)	80·3 (79·6 to 81·0)	60	8·7 (8·0 to 9·6)	12·2 (11·1 to 13·4)
Mongolia	65·1 (64·4 to 65·7)	71·6 (71·0 to 72·1)	77·0 (76·7 to 77·4)	74·9 (74·5 to 75·2)	77·3 (76·5 to 78·0)	78·2 (77·4 to 79·0)	94	13·1 (12·1 to 14·1)	20·2 (18·5 to 21·8)
Montenegro	75·9 (75·7 to 76·2)	77·5 (77·3 to 77·8)	78·0 (77·8 to 78·1)	76·3 (76·1 to 76·5)	79·0 (78·7 to 79·3)	79·5 (79·1 to 79·9)	70	3·6 (3·1 to 4·0)	4·7 (4·1 to 5·3)
North Macedonia	73·3 (73·1 to 73·6)	76·8 (76·6 to 77·0)	76·9 (76·8 to 77·1)	75·4 (75·3 to 75·6)	78·0 (77·7 to 78·3)	78·9 (78·5 to 79·4)	83	5·6 (5·1 to 6·1)	7·6 (6·9 to 8·3)
Poland	75·7 (75·6 to 75·8)	80·5 (80·4 to 80·6)	80·8 (80·7 to 80·8)	79·7 (79·7 to 79·7)	81·3 (81·2 to 81·4)	82·2 (82·1 to 82·4)	42	6·5 (6·4 to 6·7)	8·6 (8·4 to 8·8)
Romania	73·3 (73·2 to 73·5)	77·7 (77·5 to 77·8)	78·4 (78·3 to 78·4)	76·8 (76·8 to 76·9)	79·2 (79·1 to 79·4)	80·1 (80·0 to 80·3)	64	6·8 (6·6 to 7·0)	9·3 (9·0 to 9·6)
Russia	74·4 (74·3 to 74·4)	75·0 (75·0 to 75·1)	76·6 (76·6 to 76·7)	74·9 (74·9 to 74·9)	78·1 (78·0 to 78·2)	78·8 (78·6 to 78·9)	85	4·4 (4·2 to 4·5)	5·9 (5·7 to 6·1)
Serbia	73·0 (72·7 to 73·3)	76·6 (76·4 to 76·8)	77·2 (77·1 to 77·3)	75·7 (75·6 to 75·8)	77·5 (77·2 to 77·8)	78·2 (77·4 to 78·9)	95	5·1 (4·3 to 6·0)	7·1 (5·8 to 8·2)
Slovakia	75·5 (75·3 to 75·6)	79·3 (79·1 to 79·4)	80·3 (80·2 to 80·4)	78·2 (78·2 to 78·3)	80·4 (80·2 to 80·6)	81·4 (81·2 to 81·7)	49	6·0 (5·7 to 6·3)	7·9 (7·6 to 8·3)
Slovenia	77·2 (77·1 to 77·4)	82·5 (82·3 to 82·6)	83·3 (83·2 to 83·4)	83·6 (83·5 to 83·7)	84·0 (83·8 to 84·2)	84·4 (84·1 to 84·6)	18	7·1 (6·8 to 7·4)	9·2 (8·8 to 9·6)
Tajikistan	69·5 (68·9 to 70·0)	73·6 (73·1 to 74·0)	73·4 (73·1 to 73·7)	72·2 (71·4 to 72·9)	72·6 (71·6 to 73·6)	73·0 (71·9 to 74·1)	135	3·5 (2·3 to 4·8)	5·1 (3·3 to 7·0)
Turkmenistan	69·4 (68·9 to 70·0)	72·9 (72·4 to 73·5)	72·4 (71·6 to 73·3)	71·9 (70·8 to 72·9)	73·5 (72·4 to 74·7)	74·1 (72·8 to 75·3)	132	4·7 (3·3 to 6·0)	6·7 (4·8 to 8·6)
Ukraine	75·0 (74·8 to 75·1)	75·2 (75·1 to 75·4)	77·0 (76·9 to 77·1)	75·1 (75·1 to 75·2)	75·9 (74·5 to 77·1)	78·5 (77·5 to 79·5)	91	3·5 (2·5 to 4·6)	4·7 (3·3 to 6·1)
Uzbekistan	71·2 (70·9 to 71·6)	72·8 (72·5 to 73·1)	73·9 (73·8 to 74·1)	74·2 (74·0 to 74·3)	74·7 (74·3 to 75·1)	75·0 (74·6 to 75·4)	124	3·8 (3·3 to 4·3)	5·3 (4·6 to 6·1)
**High income**	**79·4 (79·3 to 79·4)**	**83·0 (83·0 to 83·1)**	**83·2 (83·2 to 83·2)**	**83·0 (83·0 to 83·0)**	**83·2 (83·1 to 83·2)**	**83·7 (83·7 to 83·7)**		**4·4 (4·3 to 4·4)**	**5·5 (5·4 to 5·5)**
Andorra	83·1 (82·7 to 83·4)	85·7 (85·3 to 86·0)	85·3 (84·9 to 85·7)	85·9 (85·5 to 86·3)	85·6 (85·1 to 86·1)	86·2 (85·6 to 86·8)	4	3·1 (2·4 to 3·8)	3·7 (2·9 to 4·6)
Argentina	75·8 (75·7 to 75·9)	78·9 (78·8 to 79·0)	79·5 (79·5 to 79·5)	77·8 (77·7 to 77·8)	79·4 (79·3 to 79·5)	80·3 (79·9 to 80·7)	59	4·5 (4·1 to 4·9)	5·9 (5·4 to 6·4)
Australia	79·9 (79·8 to 80·0)	84·2 (84·1 to 84·3)	85·8 (85·8 to 85·9)	85·7 (85·6 to 85·7)	85·1 (85·0 to 85·2)	85·8 (85·8 to 85·9)	8	5·9 (5·8 to 6·1)	7·4 (7·3 to 7·6)
Austria	79·0 (78·9 to 79·1)	83·1 (83·0 to 83·2)	83·7 (83·6 to 83·7)	83·6 (83·6 to 83·7)	83·8 (83·7 to 84·0)	84·2 (84·1 to 84·4)	22	5·3 (5·1 to 5·4)	6·7 (6·5 to 6·9)
Belgium	79·3 (79·2 to 79·4)	82·6 (82·5 to 82·7)	82·8 (82·8 to 82·9)	83·8 (83·8 to 83·9)	83·7 (83·6 to 83·8)	84·2 (84·1 to 84·3)	23	4·9 (4·8 to 5·1)	6·2 (6·0 to 6·4)
Brunei	73·8 (73·5 to 74·2)	78·0 (77·7 to 78·3)	79·4 (79·1 to 79·8)	79·2 (78·8 to 79·6)	78·9 (78·4 to 79·3)	79·4 (78·8 to 80·0)	75	5·6 (4·9 to 6·3)	7·6 (6·6 to 8·6)
Canada	80·6 (80·5 to 80·6)	83·6 (83·6 to 83·7)	83·9 (83·9 to 83·9)	84·0 (83·9 to 84·0)	83·5 (83·5 to 83·6)	84·1 (83·8 to 84·4)	24	3·5 (3·3 to 3·8)	4·4 (4·1 to 4·7)
Chile	76·9 (76·7 to 77·0)	81·6 (81·4 to 81·7)	82·2 (82·1 to 82·2)	81·6 (81·6 to 81·7)	82·3 (82·2 to 82·5)	83·3 (83·1 to 83·4)	32	6·4 (6·2 to 6·6)	8·3 (8·0 to 8·6)
Cyprus	77·4 (76·7 to 78·1)	81·6 (80·9 to 82·3)	82·1 (81·7 to 82·6)	81·7 (81·2 to 82·2)	82·0 (81·3 to 82·8)	82·6 (81·8 to 83·4)	39	5·2 (4·2 to 6·2)	6·7 (5·4 to 8·1)
Denmark	77·8 (77·7 to 78·0)	81·3 (81·2 to 81·5)	83·4 (83·3 to 83·5)	83·3 (83·2 to 83·4)	83·1 (82·9 to 83·3)	83·6 (83·4 to 83·7)	27	5·7 (5·5 to 5·9)	7·4 (7·1 to 7·6)
Finland	79·1 (79·0 to 79·3)	83·3 (83·2 to 83·5)	84·6 (84·5 to 84·6)	84·5 (84·5 to 84·6)	83·7 (83·6 to 83·9)	84·3 (84·1 to 84·4)	19	5·1 (4·9 to 5·4)	6·5 (6·2 to 6·8)
France	81·4 (81·4 to 81·5)	84·7 (84·6 to 84·7)	85·2 (85·1 to 85·2)	85·6 (85·6 to 85·7)	85·3 (85·2 to 85·3)	85·5 (85·4 to 85·5)	9	4·0 (4·0 to 4·1)	5·0 (4·9 to 5·1)
Germany	78·6 (78·6 to 78·7)	82·8 (82·8 to 82·9)	83·4 (83·4 to 83·4)	83·1 (83·1 to 83·1)	82·9 (82·9 to 82·9)	83·4 (83·1 to 83·6)	30	4·8 (4·5 to 5·0)	6·0 (5·7 to 6·4)
Greece	79·4 (79·3 to 79·5)	82·8 (82·7 to 82·9)	83·7 (83·7 to 83·8)	82·8 (82·8 to 82·9)	83·1 (83·0 to 83·2)	83·8 (83·6 to 83·9)	26	4·3 (4·2 to 4·5)	5·5 (5·3 to 5·7)
Greenland	66·6 (66·2 to 67·0)	70·6 (70·2 to 71·0)	71·7 (71·3 to 72·1)	71·5 (71·1 to 72·0)	72·6 (72·0 to 73·1)	73·4 (72·8 to 74·1)	133	6·8 (6·0 to 7·6)	10·3 (9·0 to 11·4)
Iceland	80·4 (80·1 to 80·7)	83·5 (83·2 to 83·8)	84·3 (84·1 to 84·5)	84·3 (84·0 to 84·5)	83·8 (83·4 to 84·1)	84·3 (83·8 to 84·6)	21	3·9 (3·4 to 4·4)	4·8 (4·2 to 5·4)
Ireland	77·7 (77·6 to 77·9)	82·7 (82·6 to 82·9)	84·0 (83·9 to 84·1)	84·1 (84·0 to 84·2)	83·9 (83·7 to 84·1)	84·3 (84·1 to 84·5)	20	6·5 (6·3 to 6·8)	8·4 (8·1 to 8·8)
Israel	79·1 (78·9 to 79·2)	83·7 (83·6 to 83·9)	84·9 (84·9 to 85·0)	84·8 (84·7 to 84·8)	85·0 (84·8 to 85·2)	85·3 (85·2 to 85·5)	10	6·3 (6·0 to 6·5)	7·9 (7·6 to 8·3)
Italy	80·3 (80·2 to 80·3)	84·3 (84·2 to 84·3)	84·3 (84·3 to 84·4)	84·6 (84·6 to 84·6)	84·4 (84·4 to 84·5)	85·1 (85·1 to 85·2)	12	4·8 (4·8 to 4·9)	6·0 (5·9 to 6·1)
Japan	82·1 (82·1 to 82·2)	86·4 (86·4 to 86·4)	87·8 (87·8 to 87·8)	87·6 (87·5 to 87·6)	87·2 (87·1 to 87·2)	87·2 (87·2 to 87·2)	2	5·1 (5·0 to 5·1)	6·2 (6·1 to 6·2)

Luxembourg	78·6 (78·3 to 78·8)	83·3 (83·0 to 83·5)	84·6 (84·4 to 84·8)	84·9 (84·7 to 85·1)	85·0 (84·6 to 85·3)	85·1 (84·7 to 85·5)	11	6·6 (6·1 to 7·0)	8·3 (7·8 to 9·0)
Malta	78·4 (78·1 to 78·7)	82·9 (82·7 to 83·2)	84·3 (84·1 to 84·5)	84·0 (83·8 to 84·3)	84·1 (83·7 to 84·5)	84·7 (84·3 to 85·1)	15	6·3 (5·8 to 6·8)	8·1 (7·4 to 8·7)
Monaco	79·7 (79·0 to 80·5)	81·9 (81·2 to 82·6)	82·9 (82·3 to 83·5)	82·4 (81·6 to 83·2)	82·6 (81·6 to 83·6)	83·1 (82·1 to 84·1)	34	3·4 (2·1 to 4·5)	4·2 (2·7 to 5·7)
Netherlands	80·0 (79·9 to 80·1)	82·6 (82·5 to 82·7)	82·9 (82·9 to 83·0)	82·9 (82·8 to 82·9)	83·0 (82·9 to 83·1)	83·6 (83·5 to 83·7)	28	3·6 (3·4 to 3·7)	4·5 (4·3 to 4·7)
New Zealand	78·4 (78·2 to 78·6)	82·8 (82·6 to 83·0)	84·1 (84·0 to 84·2)	83·8 (83·7 to 83·9)	83·1 (82·9 to 83·3)	83·4 (83·2 to 83·6)	31	5·0 (4·7 to 5·3)	6·3 (6·0 to 6·7)
Norway	79·8 (79·7 to 80·0)	83·2 (83·1 to 83·3)	84·8 (84·7 to 84·8)	84·7 (84·6 to 84·7)	84·2 (84·1 to 84·4)	84·6 (84·5 to 84·8)	16	4·8 (4·6 to 5·0)	6·0 (5·8 to 6·3)
Portugal	77·7 (77·5 to 77·8)	83·1 (83·0 to 83·2)	84·2 (84·2 to 84·3)	84·2 (84·1 to 84·2)	84·2 (84·1 to 84·4)	84·8 (84·7 to 85·0)	14	7·2 (7·0 to 7·4)	9·2 (9·0 to 9·5)
San Marino	82·2 (81·5 to 83·0)	86·8 (86·1 to 87·4)	85·9 (85·3 to 86·5)	86·7 (85·9 to 87·4)	86·1 (85·3 to 86·9)	87·2 (86·3 to 88·1)	3	5·0 (3·8 to 6·2)	6·0 (4·6 to 7·6)
Singapore	78·3 (78·1 to 78·5)	85·1 (84·9 to 85·3)	87·9 (87·8 to 88·0)	87·4 (87·3 to 87·5)	86·6 (86·4 to 86·8)	87·5 (87·3 to 87·8)	1	9·2 (8·9 to 9·6)	11·8 (11·4 to 12·2)
South Korea	76·0 (75·7 to 76·3)	84·0 (83·7 to 84·2)	86·3 (86·2 to 86·3)	86·2 (86·2 to 86·3)	85·3 (85·1 to 85·5)	86·1 (85·9 to 86·2)	6	10·1 (9·7 to 10·4)	13·3 (12·7 to 13·8)
Spain	80·4 (80·3 to 80·5)	85·0 (84·9 to 85·0)	84·9 (84·9 to 84·9)	85·7 (85·6 to 85·7)	85·3 (85·2 to 85·3)	85·9 (85·8 to 85·9)	7	5·5 (5·4 to 5·5)	6·8 (6·7 to 6·9)
Sweden	80·6 (80·5 to 80·7)	83·5 (83·4 to 83·6)	84·3 (84·2 to 84·3)	84·7 (84·7 to 84·8)	84·7 (84·6 to 84·9)	85·0 (84·9 to 85·1)	13	4·4 (4·3 to 4·6)	5·5 (5·3 to 5·7)
Switzerland	81·0 (80·9 to 81·2)	84·6 (84·4 to 84·7)	85·1 (85·1 to 85·2)	85·6 (85·5 to 85·6)	85·5 (85·4 to 85·7)	86·2 (86·1 to 86·3)	5	5·2 (5·0 to 5·3)	6·4 (6·2 to 6·6)
UK	78·5 (78·4 to 78·5)	82·3 (82·3 to 82·4)	82·4 (82·4 to 82·4)	82·6 (82·6 to 82·6)	82·9 (82·9 to 83·0)	82·9 (82·9 to 83·0)	35	4·4 (4·4 to 4·5)	5·6 (5·6 to 5·7)
USA	78·9 (78·8 to 78·9)	81·0 (81·0 to 81·1)	79·9 (79·9 to 79·9)	79·4 (79·4 to 79·4)	80·3 (80·3 to 80·3)	81·1 (81·1 to 81·1)	52	2·3 (2·3 to 2·3)	2·9 (2·9 to 2·9)
Uruguay	76·8 (76·6 to 77·0)	80·0 (79·8 to 80·2)	81·2 (81·1 to 81·3)	78·8 (78·7 to 78·9)	79·7 (79·4 to 79·9)	80·6 (80·3 to 80·8)	56	3·8 (3·4 to 4·1)	4·9 (4·5 to 5·4)
**Latin America and Caribbean**	**73·2 (73·1 to 73·3)**	**76·4 (76·2 to 76·6)**	**77·1 (77·1 to 77·2)**	**75·6 (75·6 to 75·6)**	**78·4 (78·3 to 78·5)**	**79·0 (78·9 to 79·1)**		**5·8 (5·7 to 5·9)**	**7·9 (7·7 to 8·1)**
Antigua and Barbuda	76·1 (75·8 to 76·5)	80·1 (79·8 to 80·4)	79·7 (79·3 to 80·0)	78·7 (78·2 to 79·1)	79·2 (78·7 to 79·7)	79·7 (79·2 to 80·3)	68	3·6 (2·9 to 4·3)	4·7 (3·8 to 5·7)
Bahamas	74·7 (74·4 to 75·1)	75·5 (75·2 to 75·8)	74·6 (74·2 to 75·0)	71·0 (70·6 to 71·5)	74·6 (74·2 to 75·1)	75·8 (75·2 to 76·4)	115	1·0 (0·3 to 1·7)	1·4 (0·4 to 2·3)
Barbados	76·4 (76·1 to 76·8)	79·5 (79·2 to 79·8)	78·8 (78·4 to 79·2)	77·8 (77·3 to 78·2)	77·9 (77·4 to 78·4)	79·0 (78·4 to 79·5)	82	2·5 (1·9 to 3·2)	3·3 (2·4 to 4·2)
Belize	75·5 (75·1 to 75·9)	76·5 (76·0 to 76·9)	76·4 (76·0 to 76·7)	74·7 (74·2 to 75·2)	76·7 (76·0 to 77·3)	78·0 (77·3 to 78·7)	96	2·5 (1·6 to 3·3)	3·3 (2·2 to 4·4)
Bermuda	78·1 (77·8 to 78·4)	83·3 (83·1 to 83·6)	83·9 (83·6 to 84·2)	82·7 (82·4 to 83·0)	82·7 (82·3 to 83·1)	83·4 (82·9 to 83·8)	29	5·3 (4·8 to 5·8)	6·8 (6·1 to 7·5)
Bolivia	65·6 (65·3 to 66·0)	71·9 (71·6 to 72·2)	68·4 (67·9 to 68·8)	68·9 (68·4 to 69·3)	73·4 (72·8 to 73·9)	74·5 (73·9 to 75·2)	127	8·9 (8·2 to 9·7)	13·6 (12·4 to 14·8)
Brazil	73·5 (73·3 to 73·6)	78·5 (78·4 to 78·7)	78·9 (78·8 to 78·9)	76·9 (76·9 to 77·0)	79·4 (79·3 to 79·6)	80·2 (80·0 to 80·3)	63	6·7 (6·5 to 7·0)	9·2 (8·9 to 9·5)
Colombia	75·4 (75·2 to 75·6)	81·1 (81·0 to 81·3)	81·0 (80·9 to 81·0)	78·9 (78·9 to 79·0)	81·9 (81·7 to 82·1)	83·2 (83·0 to 83·4)	33	7·8 (7·5 to 8·1)	10·3 (9·9 to 10·7)
Costa Rica	79·1 (78·8 to 79·3)	82·4 (82·2 to 82·6)	82·3 (82·2 to 82·4)	80·9 (80·7 to 81·0)	81·8 (81·5 to 82·1)	82·2 (81·9 to 82·4)	43	3·1 (2·7 to 3·5)	3·9 (3·4 to 4·4)
Cuba	77·3 (76·9 to 77·7)	80·5 (80·2 to 80·9)	80·3 (80·2 to 80·5)	76·1 (76·0 to 76·3)	79·8 (78·8 to 80·8)	80·6 (79·4 to 81·7)	57	3·2 (2·0 to 4·4)	4·2 (2·6 to 5·7)
Dominica	76·8 (76·5 to 77·2)	78·2 (77·9 to 78·5)	78·1 (77·7 to 78·5)	77·0 (76·5 to 77·5)	78·3 (77·8 to 78·9)	78·7 (78·2 to 79·3)	86	1·9 (1·2 to 2·6)	2·5 (1·6 to 3·4)
Dominican Republic	73·4 (73·1 to 73·8)	78·2 (77·8 to 78·5)	76·0 (75·7 to 76·2)	75·9 (75·6 to 76·1)	75·9 (75·6 to 76·3)	76·4 (75·8 to 76·9)	109	2·9 (2·3 to 3·6)	4·0 (3·0 to 4·9)
Ecuador	73·3 (73·0 to 73·5)	78·5 (78·3 to 78·7)	77·4 (77·3 to 77·5)	77·9 (77·8 to 78·0)	79·8 (79·5 to 80·0)	79·9 (79·6 to 80·1)	67	6·6 (6·2 to 7·0)	9·0 (8·5 to 9·5)
El Salvador	73·3 (73·0 to 73·6)	77·7 (77·4 to 78·0)	75·9 (75·5 to 76·2)	74·9 (74·3 to 75·4)	76·5 (75·8 to 77·1)	76·8 (76·1 to 77·5)	107	3·5 (2·7 to 4·2)	4·7 (3·7 to 5·8)
Grenada	73·1 (72·4 to 73·7)	77·6 (76·9 to 78·2)	77·6 (76·9 to 78·2)	75·4 (74·6 to 76·3)	76·5 (75·4 to 77·6)	77·2 (76·0 to 78·3)	104	4·1 (2·7 to 5·5)	5·6 (3·7 to 7·5)
Guatemala	64·8 (64·3 to 65·3)	74·2 (73·8 to 74·6)	74·3 (74·1 to 74·5)	71·9 (71·8 to 72·1)	74·3 (73·8 to 74·8)	75·5 (74·4 to 76·5)	117	10·7 (9·5 to 11·8)	16·5 (14·7 to 18·4)
Guyana	68·2 (67·4 to 68·9)	71·1 (70·4 to 71·8)	72·8 (72·1 to 73·5)	70·2 (69·6 to 70·8)	71·7 (70·7 to 72·7)	72·5 (71·3 to 73·8)	141	4·3 (2·8 to 5·8)	6·3 (4·2 to 8·5)
Haiti	52·2 (51·4 to 53·1)	27·9 (26·1 to 29·7)	62·7 (61·9 to 63·4)	61·9 (61·0 to 62·8)	62·3 (61·2 to 63·3)	62·2 (60·9 to 63·3)	196	9·9 (8·4 to 11·3)	19·0 (16·0 to 21·9)
Honduras	73·0 (72·7 to 73·3)	75·7 (75·4 to 76·0)	74·6 (74·3 to 75·0)	72·9 (72·5 to 73·3)	77·1 (76·7 to 77·5)	77·6 (77·1 to 78·1)	100	4·6 (4·0 to 5·2)	6·3 (5·5 to 7·1)
Jamaica	76·4 (75·7 to 76·9)	79·2 (78·6 to 79·8)	77·8 (77·1 to 78·6)	76·1 (75·1 to 77·0)	77·9 (76·8 to 79·0)	78·9 (77·6 to 80·1)	84	2·5 (1·1 to 3·8)	3·3 (1·4 to 5·0)
Mexico	74·0 (73·8 to 74·1)	78·1 (78·0 to 78·2)	75·5 (75·4 to 75·5)	74·9 (74·8 to 74·9)	78·4 (78·3 to 78·5)	79·2 (79·1 to 79·4)	77	5·2 (5·1 to 5·4)	7·1 (6·8 to 7·4)
Nicaragua	73·9 (73·3 to 74·5)	77·7 (77·2 to 78·3)	75·5 (75·2 to 75·8)	74·6 (74·3 to 74·9)	77·9 (77·2 to 78·5)	79·2 (78·1 to 80·2)	78	5·3 (4·1 to 6·6)	7·2 (5·5 to 9·0)
Panama	78·6 (78·3 to 78·9)	81·2 (81·0 to 81·5)	80·0 (79·9 to 80·2)	80·2 (80·0 to 80·3)	81·3 (81·0 to 81·6)	81·9 (81·4 to 82·3)	45	3·3 (2·7 to 3·8)	4·1 (3·4 to 4·8)
Paraguay	76·7 (76·4 to 77·0)	76·8 (76·5 to 77·0)	75·9 (75·7 to 76·0)	70·8 (70·6 to 70·9)	75·7 (75·1 to 76·3)	76·2 (75·6 to 76·9)	110	-0·4 (-1·1 to 0·3)	-0·6 (-1·4 to 0·4)
Peru	74·8 (74·4 to 75·1)	80·7 (80·4 to 81·0)	76·1 (76·0 to 76·2)	74·2 (74·1 to 74·3)	80·0 (79·7 to 80·3)	79·0 (78·6 to 79·3)	81	4·2 (3·7 to 4·7)	5·6 (4·9 to 6·3)
Puerto Rico	79·2 (79·0 to 79·4)	82·7 (82·6 to 83·0)	84·5 (84·3 to 84·6)	84·3 (84·1 to 84·4)	83·9 (83·7 to 84·2)	84·5 (84·2 to 84·8)	17	5·3 (5·0 to 5·7)	6·7 (6·2 to 7·2)
Saint Kitts and Nevis	71·5 (71·2 to 71·9)	76·9 (76·6 to 77·2)	77·0 (76·6 to 77·3)	76·0 (75·6 to 76·4)	77·0 (76·5 to 77·5)	77·5 (76·9 to 78·0)	103	5·9 (5·3 to 6·5)	8·3 (7·3 to 9·2)
Saint Lucia	73·2 (72·5 to 73·9)	78·8 (78·1 to 79·4)	79·9 (79·3 to 80·5)	77·4 (76·6 to 78·3)	79·4 (78·5 to 80·5)	80·2 (79·0 to 81·3)	62	7·0 (5·7 to 8·2)	9·5 (7·7 to 11·3)
Saint Vincent and the Grenadines	74·0 (73·3 to 74·7)	76·1 (75·5 to 76·7)	77·3 (76·6 to 77·9)	77·1 (76·3 to 77·8)	77·7 (76·6 to 78·7)	77·9 (76·8 to 79·1)	97	3·9 (2·6 to 5·4)	5·3 (3·5 to 7·3)
Suriname	73·5 (73·3 to 73·8)	75·4 (75·2 to 75·7)	75·3 (75·1 to 75·6)	71·5 (71·2 to 71·8)	75·0 (74·6 to 75·3)	75·9 (75·4 to 76·3)	114	2·3 (1·8 to 2·8)	3·1 (2·4 to 3·8)
Trinidad and Tobago	71·4 (71·1 to 71·6)	76·2 (75·9 to 76·5)	76·3 (75·9 to 76·6)	73·2 (72·7 to 73·6)	74·9 (74·4 to 75·4)	76·2 (75·8 to 76·7)	111	4·8 (4·3 to 5·4)	6·8 (6·0 to 7·6)
Venezuela	74·6 (74·5 to 74·8)	78·8 (78·6 to 78·9)	75·9 (75·5 to 76·2)	74·9 (74·4 to 75·3)	75·3 (74·7 to 75·8)	75·3 (74·7 to 75·9)	120	0·7 (0·1 to 1·3)	0·9 (0·1 to 1·7)
Virgin Islands	75·0 (74·4 to 75·6)	80·7 (80·1 to 81·3)	83·0 (82·4 to 83·7)	81·8 (81·0 to 82·5)	81·9 (81·0 to 83·0)	81·6 (80·5 to 82·7)	48	6·6 (5·4 to 7·8)	8·8 (7·2 to 10·5)
**North Africa and Middle East**	**67·2 (67·0 to 67·3)**	**74·1 (74·0 to 74·3)**	**73·9 (73·7 to 74·0)**	**73·4 (73·2 to 73·5)**	**75·2 (75·0 to 75·5)**	**75·4 (75·0 to 75·7)**		**8·2 (7·8 to 8·6)**	**12·2 (11·6 to 12·9)**
Afghanistan	59·8 (58·8 to 60·7)	66·3 (65·6 to 67·1)	66·4 (65·6 to 67·2)	64·4 (63·5 to 65·5)	68·7 (67·5 to 69·8)	69·0 (67·7 to 70·3)	163	9·2 (7·7 to 10·7)	15·4 (12·8 to 18·1)
Algeria	69·9 (69·6 to 70·2)	75·7 (75·4 to 76·0)	76·1 (75·9 to 76·4)	76·4 (76·1 to 76·7)	77·2 (76·9 to 77·6)	77·6 (77·1 to 78·2)	101	7·7 (7·1 to 8·4)	11·0 (10·1 to 12·0)
Bahrain	71·1 (70·3 to 71·7)	75·8 (75·1 to 76·4)	77·4 (76·9 to 78·0)	77·1 (76·3 to 77·9)	78·8 (77·8 to 79·8)	79·1 (78·0 to 80·2)	80	8·1 (6·8 to 9·4)	11·3 (9·5 to 13·3)

Egypt	64·7 (64·4 to 64·9)	69·9 (69·7 to 70·1)	70·3 (70·1 to 70·4)	69·3 (69·1 to 69·4)	70·6 (69·6 to 71·5)	71·1 (69·9 to 72·2)	149	6·4 (5·2 to 7·6)	9·9 (8·0 to 11·7)
Iran	67·8 (67·4 to 68·2)	78·3 (78·0 to 78·6)	76·2 (75·9 to 76·5)	76·6 (76·2 to 76·9)	79·8 (79·4 to 80·2)	80·6 (80·1 to 81·1)	55	12·8 (12·1 to 13·4)	18·8 (17·8 to 19·9)
Iraq	70·3 (69·9 to 70·6)	71·8 (71·5 to 72·2)	71·3 (70·9 to 71·7)	72·1 (71·7 to 72·5)	73·9 (73·4 to 74·4)	74·2 (73·5 to 74·8)	131	3·9 (3·2 to 4·6)	5·6 (4·5 to 6·6)
Jordan	74·1 (73·8 to 74·5)	76·6 (76·4 to 76·9)	76·9 (76·7 to 77·1)	75·0 (74·8 to 75·2)	79·3 (78·9 to 79·6)	80·4 (79·9 to 80·9)	58	6·3 (5·6 to 7·0)	8·5 (7·5 to 9·4)
Kuwait	74·1 (73·8 to 74·4)	78·8 (78·4 to 79·1)	79·5 (79·1 to 79·8)	79·3 (78·9 to 79·6)	81·2 (80·8 to 81·7)	81·7 (81·2 to 82·3)	46	7·6 (7·0 to 8·3)	10·3 (9·4 to 11·3)
Lebanon	77·2 (76·5 to 78·0)	82·0 (81·5 to 82·6)	76·2 (75·9 to 76·5)	80·0 (79·6 to 80·4)	80·2 (79·2 to 81·3)	80·9 (79·8 to 82·2)	53	3·7 (2·3 to 5·2)	4·8 (3·0 to 6·7)
Libya	73·1 (72·7 to 73·5)	75·4 (75·1 to 75·7)	73·8 (73·4 to 74·2)	71·0 (70·6 to 71·5)	75·6 (75·1 to 76·1)	70·0 (69·2 to 70·7)	156	-3·1 (-3·9 to -2·2)	-4·2 (-5·3 to -3·1)
Morocco	65·1 (64·5 to 65·7)	70·7 (70·2 to 71·3)	71·4 (70·9 to 71·8)	71·4 (70·9 to 71·9)	72·5 (71·7 to 73·3)	72·8 (71·8 to 73·8)	139	7·7 (6·6 to 8·8)	11·8 (10·1 to 13·6)
Oman	72·7 (72·0 to 73·3)	77·7 (77·2 to 78·3)	76·6 (76·3 to 77·0)	75·3 (74·9 to 75·8)	78·7 (78·1 to 79·4)	79·5 (78·6 to 80·3)	72	6·8 (5·8 to 7·8)	9·4 (7·9 to 10·9)
Palestine	73·4 (73·0 to 73·7)	76·2 (76·0 to 76·5)	77·8 (77·6 to 78·0)	76·4 (76·2 to 76·7)	78·4 (78·0 to 78·7)	72·0 (71·2 to 72·7)	144	-1·3 (-2·1 to -0·5)	-1·8 (-2·9 to -0·7)
Qatar	72·4 (71·7 to 73·1)	77·8 (77·0 to 78·5)	81·5 (80·9 to 82·2)	81·9 (81·2 to 82·6)	82·5 (81·5 to 83·5)	82·4 (81·3 to 83·5)	41	10·0 (8·7 to 11·3)	13·8 (11·9 to 15·6)
Saudi Arabia	71·6 (71·0 to 72·2)	74·5 (74·1 to 74·9)	73·6 (73·2 to 74·0)	74·4 (74·2 to 74·6)	75·0 (74·5 to 75·5)	75·6 (74·6 to 76·5)	116	4·0 (2·8 to 5·1)	5·6 (4·0 to 7·1)
Sudan	57·4 (56·4 to 58·3)	67·2 (66·4 to 68·0)	68·7 (68·0 to 69·6)	69·2 (68·2 to 70·2)	70·3 (69·0 to 71·4)	70·5 (69·3 to 71·7)	151	13·2 (11·6 to 14·7)	23·0 (20·0 to 26·1)
Syria	71·2 (70·9 to 71·6)	76·0 (75·8 to 76·2)	76·6 (76·2 to 76·9)	76·1 (75·7 to 76·5)	77·9 (77·5 to 78·2)	77·1 (76·6 to 77·6)	106	5·8 (5·2 to 6·4)	8·2 (7·3 to 9·0)
Tunisia	71·4 (71·1 to 71·8)	76·7 (76·4 to 77·0)	75·5 (75·3 to 75·6)	73·0 (72·8 to 73·1)	77·3 (77·1 to 77·5)	77·9 (77·5 to 78·4)	98	6·5 (6·0 to 7·1)	9·1 (8·3 to 9·9)
Türkiye	71·2 (70·8 to 71·6)	80·0 (79·7 to 80·3)	79·2 (79·2 to 79·3)	78·2 (78·1 to 78·2)	79·6 (79·4 to 79·8)	78·3 (77·2 to 79·4)	93	7·2 (6·0 to 8·3)	10·1 (8·4 to 11·7)
United Arab Emirates	71·9 (71·3 to 72·4)	77·3 (76·8 to 77·8)	78·8 (78·3 to 79·2)	78·7 (78·2 to 79·2)	79·4 (78·8 to 80·1)	79·6 (78·9 to 80·4)	69	7·8 (6·8 to 8·7)	10·8 (9·4 to 12·1)
Yemen	62·6 (61·7 to 63·4)	71·8 (71·0 to 72·5)	72·2 (71·5 to 73·0)	72·4 (71·4 to 73·3)	73·8 (72·6 to 74·9)	74·2 (72·9 to 75·3)	130	11·6 (10·0 to 13·0)	18·5 (15·9 to 21·0)
**South Asia**	**59·0 (58·4 to 59·6)**	**69·3 (68·9 to 69·6)**	**71·4 (71·2 to 71·6)**	**70·5 (70·2 to 70·7)**	**72·1 (71·3 to 73·0)**	**72·4 (71·5 to 73·3)**		**13·4 (12·2 to 14·4)**	**22·6 (20·6 to 24·6)**
Bangladesh	56·6 (55·8 to 57·4)	69·3 (68·7 to 69·9)	70·5 (69·8 to 71·2)	69·3 (68·4 to 70·2)	71·2 (70·3 to 72·2)	71·2 (70·1 to 72·2)	147	14·6 (13·3 to 16·0)	25·8 (23·2 to 28·6)
Bhutan	58·7 (57·8 to 59·6)	69·5 (68·8 to 70·2)	72·2 (71·4 to 73·0)	72·3 (71·4 to 73·2)	72·3 (71·2 to 73·3)	72·4 (71·2 to 73·6)	142	13·7 (12·3 to 15·3)	23·3 (20·7 to 26·3)
India	58·9 (58·2 to 59·6)	69·6 (69·1 to 70·0)	72·1 (71·9 to 72·4)	71·3 (71·0 to 71·5)	72·7 (71·7 to 73·7)	73·0 (71·8 to 74·1)	136	14·0 (12·6 to 15·4)	23·8 (21·3 to 26·4)
Nepal	58·6 (57·7 to 59·4)	72·8 (72·1 to 73·4)	74·6 (73·8 to 75·3)	72·9 (72·1 to 73·8)	75·1 (74·0 to 76·1)	75·1 (74·0 to 76·3)	123	16·6 (15·1 to 18·0)	28·3 (25·6 to 31·0)
Pakistan	62·6 (61·7 to 63·4)	67·3 (66·6 to 68·1)	67·3 (66·5 to 68·2)	66·6 (65·5 to 67·6)	69·6 (68·4 to 70·8)	70·1 (68·8 to 71·3)	155	7·5 (6·0 to 9·1)	12·0 (9·6 to 14·6)
**Southeast Asia, East Asia, and Oceania**	**69·5 (69·0 to 70·0)**	**77·3 (77·0 to 77·6)**	**80·8 (80·7 to 80·9)**	**79·8 (79·5 to 80·1)**	**80·2 (79·6 to 80·9)**	**80·2 (79·6 to 81·0)**		**10·7 (9·9 to 11·6)**	**15·4 (14·1 to 16·7)**
American Samoa	73·2 (72·6 to 73·8)	72·6 (71·9 to 73·2)	73·9 (73·2 to 74·6)	72·2 (71·2 to 73·2)	74·2 (73·2 to 75·3)	74·4 (73·2 to 75·5)	129	1·2 (-0·2 to 2·4)	1·6 (-0·2 to 3·3)
Cambodia	61·3 (60·4 to 62·1)	72·8 (72·1 to 73·5)	75·7 (74·9 to 76·4)	75·5 (74·6 to 76·3)	75·5 (74·5 to 76·5)	75·2 (74·1 to 76·3)	121	14·0 (12·6 to 15·4)	22·8 (20·3 to 25·3)
China	69·8 (69·2 to 70·4)	78·9 (78·4 to 79·3)	83·3 (83·2 to 83·5)	83·0 (82·7 to 83·4)	82·9 (82·0 to 83·7)	82·8 (81·8 to 83·7)	38	13·0 (11·8 to 14·1)	18·6 (16·8 to 20·4)
Cook Islands	71·3 (71·0 to 71·6)	75·5 (75·2 to 75·9)	76·1 (75·8 to 76·6)	75·1 (74·7 to 75·6)	76·2 (75·7 to 76·6)	76·5 (75·9 to 77·1)	108	5·2 (4·5 to 5·9)	7·3 (6·3 to 8·4)
Federated States of Micronesia	68·0 (67·7 to 68·3)	70·1 (69·8 to 70·5)	70·5 (70·2 to 70·9)	69·3 (68·9 to 69·7)	70·6 (70·2 to 71·1)	70·9 (70·3 to 71·5)	150	2·9 (2·2 to 3·6)	4·3 (3·2 to 5·3)
Fiji	70·6 (70·3 to 70·9)	69·2 (68·9 to 69·5)	69·8 (69·6 to 70·1)	68·0 (67·7 to 68·3)	69·5 (69·1 to 69·8)	69·5 (68·9 to 70·0)	159	-1·1 (-1·8 to -0·4)	-1·6 (-2·5 to -0·6)
Guam	77·2 (76·9 to 77·6)	80·4 (80·1 to 80·7)	79·1 (78·8 to 79·5)	78·2 (77·8 to 78·6)	79·1 (78·6 to 79·6)	79·5 (78·9 to 80·0)	73	2·2 (1·6 to 2·9)	2·9 (2·0 to 3·7)
Indonesia	67·9 (67·0 to 68·8)	73·4 (72·6 to 74·2)	74·5 (73·6 to 75·4)	71·9 (70·9 to 72·9)	75·1 (74·0 to 76·4)	75·2 (74·0 to 76·4)	122	7·3 (5·8 to 8·8)	10·7 (8·6 to 13·0)
Kiribati	69·3 (68·9 to 69·7)	72·6 (72·2 to 72·9)	73·9 (73·4 to 74·3)	72·6 (72·0 to 73·1)	74·1 (73·5 to 74·7)	74·5 (73·7 to 75·2)	128	5·2 (4·3 to 6·0)	7·5 (6·3 to 8·7)
Laos	52·7 (51·6 to 53·8)	65·8 (65·0 to 66·5)	67·3 (66·5 to 68·1)	67·3 (66·3 to 68·3)	67·1 (65·9 to 68·2)	67·2 (66·0 to 68·4)	173	14·5 (12·8 to 16·1)	27·4 (24·0 to 30·8)
Malaysia	73·7 (73·3 to 74·1)	77·2 (76·6 to 77·8)	78·8 (78·6 to 79·1)	75·6 (75·4 to 75·8)	77·3 (76·9 to 77·7)	78·5 (78·1 to 78·9)	90	4·8 (4·2 to 5·4)	6·5 (5·7 to 7·4)
Maldives	64·9 (64·6 to 65·2)	75·6 (75·3 to 75·8)	77·0 (76·7 to 77·3)	76·0 (75·7 to 76·3)	76·6 (76·2 to 77·0)	77·1 (76·5 to 77·6)	105	12·2 (11·6 to 12·8)	18·8 (17·9 to 19·8)
Marshall Islands	65·2 (64·9 to 65·5)	65·0 (64·6 to 65·3)	65·0 (64·6 to 65·4)	63·6 (63·1 to 64·1)	65·0 (64·5 to 65·5)	65·2 (64·5 to 65·9)	182	0·0 (-0·7 to 0·7)	0·0 (-1·1 to 1·1)
Mauritius	73·9 (73·6 to 74·3)	77·5 (77·3 to 77·9)	78·1 (77·9 to 78·3)	76·5 (76·3 to 76·7)	76·9 (76·5 to 77·3)	77·6 (77·1 to 78·0)	102	3·6 (3·1 to 4·2)	4·9 (4·2 to 5·7)
Myanmar	59·2 (58·3 to 60·0)	67·5 (66·7 to 68·2)	69·7 (69·0 to 70·4)	68·2 (67·3 to 69·1)	69·4 (68·3 to 70·6)	69·4 (68·2 to 70·5)	160	10·2 (8·7 to 11·6)	17·3 (14·6 to 19·9)
Nauru	61·3 (61·0 to 61·6)	59·4 (59·0 to 59·7)	60·4 (60·0 to 60·8)	58·3 (57·9 to 58·8)	60·7 (60·1 to 61·2)	61·1 (60·4 to 61·7)	198	-0·2 (-0·9 to 0·5)	-0·3 (-1·5 to 0·9)
Niue	73·2 (72·9 to 73·5)	74·6 (74·3 to 74·9)	75·9 (75·5 to 76·2)	74·5 (74·0 to 74·9)	74·5 (74·0 to 75·0)	74·8 (74·2 to 75·4)	125	1·6 (1·0 to 2·3)	2·3 (1·4 to 3·2)
North Korea	74·7 (74·0 to 75·3)	75·3 (74·7 to 76·0)	75·9 (75·3 to 76·7)	76·1 (75·2 to 77·0)	75·9 (75·0 to 77·0)	76·0 (74·9 to 77·1)	113	1·3 (0·0 to 2·6)	1·8 (0·0 to 3·5)
Northern Mariana Islands	71·7 (71·4 to 71·9)	72·3 (72·0 to 72·6)	72·3 (72·0 to 72·7)	70·7 (70·3 to 71·1)	72·3 (71·9 to 72·8)	72·8 (72·3 to 73·4)	138	1·2 (0·5 to 1·8)	1·6 (0·7 to 2·5)
Palau	69·0 (68·7 to 69·3)	70·0 (69·7 to 70·3)	69·8 (69·4 to 70·2)	68·3 (67·9 to 68·7)	69·9 (69·4 to 70·4)	70·2 (69·6 to 70·8)	152	1·2 (0·5 to 1·8)	1·7 (0·8 to 2·6)
Papua New Guinea	63·7 (63·0 to 64·4)	65·5 (64·8 to 66·3)	67·2 (66·4 to 68·0)	67·0 (66·0 to 67·9)	66·8 (65·7 to 68·0)	67·0 (65·8 to 68·2)	175	3·2 (1·9 to 4·7)	5·1 (2·9 to 7·4)
Philippines	72·2 (71·9 to 72·6)	74·4 (74·2 to 74·6)	75·2 (75·1 to 75·3)	71·1 (71·1 to 71·2)	74·1 (73·9 to 74·3)	75·4 (75·1 to 75·6)	119	3·1 (2·7 to 3·5)	4·3 (3·7 to 4·9)
Samoa	74·6 (74·3 to 74·9)	75·1 (74·8 to 75·4)	74·5 (74·1 to 74·9)	73·2 (72·8 to 73·7)	74·5 (74·0 to 74·9)	74·5 (73·9 to 75·1)	126	0·0 (-0·8 to 0·6)	0·0 (-1·0 to 0·9)
Seychelles	75·4 (75·1 to 75·7)	77·5 (77·2 to 77·8)	79·4 (79·1 to 79·7)	74·6 (74·3 to 75·0)	77·9 (77·5 to 78·4)	78·5 (78·1 to 79·0)	88	3·1 (2·5 to 3·6)	4·1 (3·3 to 4·8)
Solomon Islands	65·5 (65·2 to 65·9)	67·8 (67·5 to 68·1)	66·2 (65·8 to 66·6)	66·0 (65·6 to 66·5)	66·0 (65·5 to 66·6)	66·2 (65·5 to 66·8)	179	0·6 (-0·1 to 1·4)	1·0 (-0·2 to 2·1)
Sri Lanka	74·0 (73·8 to 74·3)	77·8 (77·6 to 78·0)	80·8 (80·6 to 81·0)	79·0 (78·8 to 79·1)	79·3 (79·1 to 79·6)	79·5 (79·2 to 79·8)	74	5·5 (5·1 to 5·9)	7·4 (6·8 to 7·9)
Taiwan*	77·0 (76·8 to 77·1)	82·6 (82·5 to 82·7)	84·3 (84·2 to 84·4)	84·1 (84·0 to 84·2)	83·2 (83·1 to 83·4)	83·9 (83·7 to 84·0)	25	6·9 (6·7 to 7·1)	9·0 (8·7 to 9·2)
Thailand	75·3 (75·2 to 75·5)	78·4 (78·3 to 78·5)	81·7 (81·6 to 81·8)	80·8 (80·7 to 80·9)	80·5 (80·3 to 80·7)	81·4 (81·2 to 81·7)	50	6·1 (5·8 to 6·4)	8·1 (7·7 to 8·5)
Timor-Leste	58·2 (57·2 to 59·0)	69·4 (68·7 to 70·1)	69·8 (69·0 to 70·6)	69·6 (68·7 to 70·4)	70·0 (69·0 to 71·1)	70·1 (68·9 to 71·3)	154	11·9 (10·4 to 13·4)	20·5 (17·8 to 23·2)

Tokelau	73·7 (73·4 to 74·0)	75·2 (74·9 to 75·5)	76·9 (76·5 to 77·2)	75·9 (75·5 to 76·3)	75·8 (75·4 to 76·3)	76·1 (75·5 to 76·7)	112	2·4 (1·7 to 3·1)	3·3 (2·4 to 4·2)
Tonga	73·5 (73·2 to 73·8)	73·6 (73·2 to 73·9)	72·9 (72·5 to 73·3)	70·9 (70·4 to 71·3)	72·6 (72·1 to 73·1)	72·9 (72·3 to 73·6)	137	-0·6 (-1·3 to 0·1)	-0·8 (-1·8 to 0·2)
Tuvalu	63·1 (62·8 to 63·5)	66·1 (65·8 to 66·5)	66·5 (66·1 to 66·9)	65·5 (65·0 to 65·9)	66·6 (66·1 to 67·1)	66·8 (66·2 to 67·4)	176	3·7 (2·9 to 4·4)	5·8 (4·6 to 7·0)
Vanuatu	71·5 (71·2 to 71·9)	73·2 (72·9 to 73·6)	73·1 (72·7 to 73·5)	72·7 (72·3 to 73·2)	73·2 (72·7 to 73·7)	73·3 (72·7 to 74·0)	134	1·8 (1·1 to 2·5)	2·5 (1·5 to 3·5)
Viet Nam	73·5 (72·9 to 74·1)	78·6 (77·9 to 79·4)	80·1 (79·3 to 80·9)	79·5 (78·5 to 80·4)	79·9 (78·8 to 81·0)	80·2 (79·0 to 81·5)	61	6·7 (5·3 to 8·1)	9·1 (7·2 to 11·1)
**Sub-Saharan Africa**	**54·6 (54·3 to 54·9)**	**61·2 (61·0 to 61·4)**	**65·0 (64·8 to 65·2)**	**64·0 (63·8 to 64·2)**	**65·7 (65·5 to 66·0)**	**66·1 (65·8 to 66·4)**		**11·5 (11·1 to 11·9)**	**21·1 (20·3 to 21·9)**
Angola	54·0 (53·0 to 55·0)	64·1 (63·4 to 64·9)	67·0 (66·2 to 67·8)	66·0 (65·1 to 67·0)	67·7 (66·7 to 68·8)	67·9 (66·6 to 69·1)	169	13·9 (12·3 to 15·4)	25·8 (22·6 to 28·9)
Benin	57·7 (56·7 to 58·7)	65·9 (65·1 to 66·7)	68·3 (67·5 to 69·0)	68·5 (67·7 to 69·4)	68·9 (67·8 to 69·9)	69·2 (68·1 to 70·3)	161	11·5 (10·1 to 13·0)	20·0 (17·3 to 22·8)
Botswana	67·8 (67·5 to 68·2)	68·4 (68·1 to 68·8)	71·7 (71·4 to 72·1)	67·9 (67·5 to 68·2)	71·8 (71·3 to 72·2)	72·7 (72·0 to 73·3)	140	4·8 (4·2 to 5·6)	7·2 (6·1 to 8·2)
Burkina Faso	51·8 (50·7 to 52·9)	65·5 (64·7 to 66·2)	69·7 (69·1 to 70·4)	70·0 (69·1 to 70·9)	70·2 (69·2 to 71·2)	70·1 (69·1 to 71·2)	153	18·3 (16·8 to 19·8)	35·3 (32·0 to 38·8)
Burundi	50·6 (49·6 to 51·6)	61·7 (60·9 to 62·5)	64·1 (63·4 to 64·9)	62·7 (61·8 to 63·6)	64·1 (63·0 to 65·2)	64·2 (63·1 to 65·4)	187	13·6 (12·1 to 15·2)	26·9 (23·6 to 30·4)
Cabo Verde	72·3 (71·8 to 72·9)	78·5 (78·0 to 79·1)	77·1 (76·5 to 77·7)	76·0 (75·3 to 76·8)	77·9 (76·9 to 78·7)	78·5 (77·6 to 79·5)	89	6·2 (5·1 to 7·3)	8·5 (7·0 to 10·1)
Cameroon	56·8 (56·0 to 57·6)	56·5 (55·7 to 57·4)	61·4 (60·6 to 62·2)	60·2 (59·3 to 61·2)	62·5 (61·5 to 63·6)	62·9 (61·7 to 64·0)	191	6·1 (4·7 to 7·5)	10·8 (8·2 to 13·2)
Central African Republic	50·2 (49·2 to 51·0)	50·9 (50·0 to 51·8)	54·8 (53·9 to 55·6)	55·0 (54·0 to 56·0)	55·5 (54·3 to 56·6)	55·9 (54·6 to 57·1)	204	5·7 (4·1 to 7·3)	11·4 (8·1 to 14·7)
Chad	49·7 (48·7 to 50·7)	56·3 (55·5 to 57·1)	58·7 (57·9 to 59·4)	59·7 (58·8 to 60·6)	59·5 (58·5 to 60·5)	59·7 (58·6 to 60·8)	202	10·0 (8·5 to 11·5)	20·1 (16·9 to 23·3)
Comoros	68·8 (68·1 to 69·6)	76·7 (76·0 to 77·4)	77·0 (76·2 to 77·7)	74·9 (74·1 to 75·7)	77·7 (76·8 to 78·8)	78·4 (77·2 to 79·5)	92	9·5 (8·2 to 10·9)	13·9 (11·8 to 15·9)
Congo (Brazzaville)	56·7 (55·8 to 57·6)	63·0 (62·3 to 63·7)	63·9 (63·1 to 64·6)	62·9 (61·9 to 63·8)	64·3 (63·3 to 65·4)	64·5 (63·3 to 65·8)	185	7·8 (6·3 to 9·3)	13·8 (10·9 to 16·6)
Côte d'Ivoire	56·2 (55·4 to 57·1)	60·9 (60·1 to 61·7)	65·2 (64·4 to 65·9)	65·5 (64·7 to 66·3)	66·0 (64·9 to 67·0)	66·2 (65·0 to 67·3)	180	9·9 (8·5 to 11·5)	17·7 (15·1 to 20·7)
DR Congo	54·6 (53·7 to 55·5)	58·5 (57·7 to 59·2)	61·5 (60·7 to 62·3)	61·6 (60·7 to 62·5)	62·2 (61·0 to 63·2)	62·6 (61·4 to 63·8)	193	8·1 (6·5 to 9·5)	14·8 (11·9 to 17·6)
Djibouti	64·0 (63·2 to 64·7)	66·7 (66·0 to 67·5)	69·1 (68·3 to 69·9)	67·8 (66·9 to 68·7)	69·5 (68·3 to 70·6)	69·9 (68·7 to 71·2)	157	5·9 (4·5 to 7·4)	9·3 (7·0 to 11·7)
Equatorial Guinea	52·5 (51·5 to 53·3)	61·9 (61·1 to 62·7)	63·2 (62·4 to 64·1)	62·8 (61·8 to 63·8)	63·7 (62·5 to 64·9)	63·9 (62·5 to 65·2)	189	11·4 (9·7 to 13·1)	21·7 (18·3 to 25·2)
Eritrea	50·3 (49·3 to 51·2)	61·8 (61·0 to 62·6)	63·9 (63·2 to 64·7)	64·1 (63·1 to 65·0)	63·6 (62·5 to 64·7)	64·5 (63·2 to 65·8)	184	14·2 (12·5 to 15·9)	28·3 (24·7 to 32·0)
Eswatini	60·6 (59·8 to 61·4)	51·4 (50·3 to 52·5)	58·1 (57·2 to 59·1)	56·4 (55·3 to 57·6)	60·7 (59·3 to 62·0)	62·4 (61·0 to 63·9)	195	1·8 (0·2 to 3·5)	2·9 (0·3 to 5·7)
Ethiopia	48·0 (47·0 to 49·0)	62·4 (61·7 to 63·1)	66·5 (65·7 to 67·2)	65·5 (64·7 to 66·3)	66·6 (65·6 to 67·6)	66·7 (65·5 to 67·9)	177	18·7 (17·3 to 20·3)	38·9 (35·4 to 42·9)
Gabon	64·4 (63·7 to 65·1)	65·6 (64·8 to 66·3)	66·0 (65·3 to 66·8)	65·7 (64·6 to 66·6)	67·3 (66·1 to 68·4)	67·8 (66·6 to 69·0)	170	3·4 (1·9 to 4·6)	5·2 (3·0 to 7·2)
Ghana	58·4 (57·6 to 59·3)	65·6 (64·8 to 66·3)	67·4 (66·7 to 68·2)	66·9 (66·0 to 67·8)	67·8 (66·6 to 68·8)	68·0 (66·9 to 69·2)	168	9·6 (8·2 to 11·1)	16·4 (13·9 to 19·1)
Guinea	49·3 (48·2 to 50·4)	59·7 (59·0 to 60·5)	62·4 (61·5 to 63·1)	62·6 (61·5 to 63·4)	63·0 (62·0 to 64·1)	63·4 (62·3 to 64·6)	190	14·1 (12·7 to 15·7)	28·7 (25·3 to 32·3)
Guinea-Bissau	52·8 (51·9 to 53·8)	58·6 (57·8 to 59·4)	60·6 (59·8 to 61·4)	60·9 (60·0 to 61·8)	61·4 (60·4 to 62·5)	61·9 (60·7 to 63·0)	197	9·1 (7·5 to 10·6)	17·2 (14·1 to 20·5)
Kenya	63·9 (63·2 to 64·7)	66·3 (65·6 to 67·0)	69·5 (68·8 to 70·3)	68·2 (67·2 to 69·2)	71·2 (70·1 to 72·3)	71·7 (70·5 to 73·0)	146	7·8 (6·3 to 9·3)	12·2 (9·9 to 14·6)
Lesotho	62·0 (61·4 to 62·7)	56·2 (55·3 to 57·1)	58·4 (57·6 to 59·4)	56·7 (55·7 to 57·7)	59·6 (58·4 to 60·8)	60·4 (59·1 to 61·8)	201	-1·6 (-3·2 to 0·1)	-2·6 (-5·1 to 0·1)
Liberia	51·0 (50·0 to 52·0)	59·2 (58·4 to 60·0)	60·6 (59·8 to 61·4)	58·3 (57·3 to 59·2)	62·2 (61·1 to 63·4)	62·7 (61·5 to 63·9)	192	11·7 (10·1 to 13·2)	23·0 (19·5 to 26·3)
Madagascar	53·2 (52·4 to 54·1)	62·2 (61·5 to 62·9)	63·6 (62·9 to 64·3)	63·2 (62·3 to 64·1)	63·5 (62·4 to 64·5)	63·9 (62·8 to 65·0)	188	10·7 (9·3 to 12·0)	20·1 (17·3 to 22·7)
Malawi	47·6 (46·6 to 48·6)	59·8 (59·0 to 60·6)	64·3 (63·5 to 65·1)	59·8 (58·8 to 60·8)	64·0 (62·9 to 65·2)	64·3 (63·0 to 65·6)	186	16·7 (15·0 to 18·2)	35·1 (31·0 to 38·9)
Mali	48·9 (47·8 to 50·0)	62·7 (61·9 to 63·5)	63·4 (62·6 to 64·2)	63·1 (62·1 to 64·0)	64·4 (63·4 to 65·5)	64·9 (63·8 to 66·1)	183	16·0 (14·5 to 17·6)	32·7 (29·2 to 36·5)
Mauritania	61·9 (61·1 to 62·6)	67·2 (66·6 to 67·9)	67·3 (66·6 to 68·1)	66·5 (65·6 to 67·3)	67·8 (66·9 to 68·8)	68·8 (67·6 to 69·8)	164	6·9 (5·5 to 8·1)	11·1 (8·9 to 13·3)
Mozambique	55·3 (54·3 to 56·4)	63·2 (62·4 to 64·0)	66·3 (65·6 to 67·2)	64·7 (63·8 to 65·6)	67·1 (65·9 to 68·1)	67·7 (66·6 to 68·9)	171	12·4 (11·0 to 13·9)	22·4 (19·5 to 25·5)
Namibia	64·1 (63·7 to 64·5)	65·4 (65·0 to 65·7)	67·4 (67·0 to 67·9)	62·6 (62·2 to 63·1)	67·9 (67·4 to 68·5)	69·1 (68·5 to 69·8)	162	5·0 (4·2 to 5·8)	7·9 (6·6 to 9·1)
Niger	43·1 (41·8 to 44·4)	57·0 (56·1 to 57·8)	59·3 (58·5 to 60·1)	59·9 (59·1 to 60·9)	60·1 (59·0 to 61·1)	60·6 (59·4 to 61·6)	200	17·5 (15·7 to 19·2)	40·5 (35·6 to 45·7)
Nigeria	52·8 (51·7 to 53·9)	61·1 (60·3 to 61·9)	64·8 (64·1 to 65·6)	65·1 (64·3 to 66·0)	65·7 (64·8 to 66·7)	65·9 (64·9 to 67·1)	181	13·1 (11·6 to 14·6)	24·8 (21·7 to 28·2)
Rwanda	49·7 (48·8 to 50·7)	65·2 (64·5 to 65·9)	68·0 (67·4 to 68·8)	67·0 (66·2 to 67·9)	67·7 (66·6 to 68·6)	67·6 (66·5 to 68·7)	172	17·8 (16·5 to 19·3)	35·9 (32·6 to 39·3)
Senegal	60·1 (59·2 to 60·9)	69·3 (68·6 to 70·0)	70·7 (70·1 to 71·5)	69·8 (69·0 to 70·7)	71·5 (70·4 to 72·4)	71·7 (70·7 to 72·9)	145	11·6 (10·2 to 13·1)	19·3 (16·9 to 22·0)
Sierra Leone	49·6 (48·6 to 50·6)	55·7 (54·9 to 56·6)	59·6 (58·9 to 60·4)	60·3 (59·4 to 61·2)	60·5 (59·4 to 61·6)	60·8 (59·6 to 61·9)	199	11·2 (9·7 to 12·7)	22·6 (19·5 to 26·1)
Somalia	54·5 (53·5 to 55·5)	54·7 (53·7 to 55·6)	65·8 (65·0 to 66·6)	63·2 (62·2 to 64·2)	66·6 (65·6 to 67·7)	67·0 (65·9 to 68·1)	174	12·6 (11·1 to 14·1)	23·1 (20·1 to 26·3)
South Africa	72·5 (71·6 to 73·3)	60·8 (60·4 to 61·2)	67·6 (67·5 to 67·7)	64·9 (64·8 to 65·0)	68·8 (68·6 to 69·2)	69·7 (69·4 to 70·1)	158	-2·8 (-3·7 to -1·8)	-3·9 (-5·0 to -2·6)
South Sudan	53·3 (52·4 to 54·3)	58·6 (57·7 to 59·4)	59·0 (58·1 to 59·7)	59·1 (58·2 to 60·1)	58·9 (57·8 to 60·1)	59·0 (57·8 to 60·2)	203	5·7 (4·1 to 7·1)	10·7 (7·7 to 13·6)
São Tomé and Príncipe	65·4 (64·7 to 66·2)	68·8 (68·2 to 69·5)	69·2 (68·5 to 69·9)	69·1 (68·2 to 70·0)	71·0 (70·0 to 72·0)	72·1 (71·0 to 73·2)	143	6·7 (5·4 to 8·0)	10·2 (8·2 to 12·3)
Tanzania	56·3 (55·4 to 57·2)	63·4 (62·6 to 64·2)	69·0 (68·3 to 69·7)	66·8 (66·0 to 67·7)	70·7 (69·6 to 71·7)	71·1 (70·0 to 72·2)	148	14·8 (13·4 to 16·3)	26·3 (23·4 to 29·2)
The Gambia	63·2 (62·4 to 63·9)	67·5 (66·7 to 68·1)	66·4 (65·6 to 67·1)	65·5 (64·6 to 66·4)	67·8 (66·7 to 68·7)	68·6 (67·4 to 69·7)	165	5·4 (4·0 to 6·7)	8·5 (6·3 to 10·7)
Togo	58·2 (57·3 to 59·1)	61·3 (60·5 to 62·1)	65·3 (64·5 to 66·1)	65·2 (64·3 to 66·0)	65·8 (64·8 to 66·8)	66·2 (65·1 to 67·4)	178	8·0 (6·5 to 9·4)	13·7 (11·1 to 16·4)
Uganda	50·1 (49·1 to 51·0)	62·3 (61·4 to 63·0)	67·6 (66·8 to 68·3)	65·2 (64·3 to 66·1)	67·7 (66·6 to 68·8)	68·1 (66·9 to 69·3)	167	18·1 (16·5 to 19·5)	36·1 (32·6 to 39·6)
Zambia	50·1 (49·1 to 51·0)	57·0 (56·3 to 57·8)	61·3 (60·5 to 62·1)	59·0 (58·1 to 60·0)	62·3 (61·2 to 63·4)	62·6 (61·3 to 63·8)	194	12·5 (10·9 to 14·1)	25·0 (21·5 to 28·6)
Zimbabwe	68·6 (67·9 to 69·4)	58·6 (57·7 to 59·4)	65·9 (65·0 to 66·8)	62·2 (61·0 to 63·3)	67·1 (66·0 to 68·2)	68·4 (67·1 to 69·7)	166	-0·2 (-1·7 to 1·3)	-0·3 (-2·4 to 1·9)
**Low SDI**	**54·5 (54·2 to 54·8)**	**63·5 (63·3 to 63·7)**	**66·6 (66·5 to 66·8)**	**65·9 (65·7 to 66·1)**	**67·3 (67·0 to 67·7)**	**67·6 (67·1 to 68·0)**		**13·1 (12·5 to 13·5)**	**24·0 (22·9 to 24·9)**
**Low-middle SDI**	**60·6 (60·1 to 61·0)**	**69·0 (68·7 to 69·3)**	**70·7 (70·5 to 70·9)**	**69·9 (69·7 to 70·1)**	**71·4 (70·8 to 72·0)**	**71·6 (70·9 to 72·3)**		**11·1 (10·3 to 11·8)**	**18·3 (16·9 to 19·6)**

**Middle SDI**	**68·7 (68·4 to 68·9)**	**74·4 (74·2 to 74·7)**	**75·6 (75·4 to 75·8)**	**74·1 (73·8 to 74·4)**	**75·8 (75·4 to 76·2)**	**76·1 (75·7 to 76·5)**	**7·5 (7·0 to 7·9)**	**10·9 (10·2 to 11·6)**
**High-middle SDI**	**69·2 (68·8 to 69·6)**	**76·3 (76·0 to 76·5)**	**78·5 (78·4 to 78·6)**	**77·5 (77·3 to 77·6)**	**78·9 (78·5 to 79·4)**	**79·2 (78·7 to 79·7)**	**10·0 (9·3 to 10·6)**	**14·4 (13·5 to 15·4)**
**High SDI**	**75·8 (75·7 to 76·0)**	**80·5 (80·4 to 80·6)**	**81·9 (81·9 to 82·0)**	**81·3 (81·2 to 81·4)**	**82·1 (81·9 to 82·3)**	**82·5 (82·2 to 82·7)**	**6·6 (6·3 to 6·9)**	**8·8 (8·4 to 9·1)**

*United Nations convention recognises Taiwan as a province of China

Table S8: Female healthy life expectancy, 1990, 2010, 2020, 2021, 2022, and 2023, and absolute and relative changes, 1990–2023, globally, by SDI quintile, and for GBD super-regions and countries and territories

Location	1990	2010	2020	2021	2022	2023	Rank (2023)	Absolute Change (1990-2023)	Relative Change, % (1990-2023)
**Global**	**57·3 (54·6 to 59·7)**	**62·5 (59·6 to 65·1)**	**63·8 (60·5 to 66·6)**	**63·0 (59·7 to 65·7)**	**63·9 (60·5 to 66·7)**	**64·2 (60·7 to 67·0)**		**6·9 (6·2 to 7·5)**	**12·0 (11·1 to 12·8)**
**Central Europe, Eastern Europe, and Central Asia**	**63·1 (60·1 to 65·7)**	**65·0 (62·0 to 67·6)**	**65·5 (62·2 to 68·2)**	**64·2 (61·0 to 66·9)**	**65·9 (62·4 to 68·8)**	**66·8 (63·3 to 69·6)**		**3·7 (3·0 to 4·2)**	**5·8 (4·9 to 6·5)**
Albania	64·4 (61·3 to 67·0)	69·2 (65·9 to 71·9)	68·3 (64·9 to 71·1)	67·5 (64·1 to 70·3)	69·0 (65·5 to 71·6)	69·9 (66·3 to 72·7)	34	5·4 (4·4 to 6·3)	8·4 (6·8 to 9·9)
Armenia	63·4 (60·7 to 65·8)	66·4 (63·4 to 68·9)	65·1 (62·0 to 67·8)	66·0 (62·7 to 68·8)	68·4 (65·0 to 71·5)	69·7 (66·1 to 72·9)	35	6·3 (5·2 to 7·3)	10·0 (8·5 to 11·3)
Azerbaijan	60·1 (57·2 to 62·5)	63·7 (60·8 to 66·3)	63·0 (60·2 to 65·5)	62·6 (59·8 to 65·1)	64·4 (61·4 to 67·2)	64·2 (61·2 to 67·1)	116	4·2 (3·4 to 5·0)	7·0 (5·6 to 8·4)
Belarus	64·9 (61·8 to 67·5)	65·7 (62·6 to 68·3)	67·4 (64·0 to 70·2)	66·8 (63·4 to 69·6)	66·7 (63·2 to 69·6)	67·4 (63·9 to 70·2)	68	2·5 (1·6 to 3·2)	3·8 (2·6 to 5·0)
Bosnia and Herzegovina	65·0 (62·0 to 67·4)	67·4 (64·2 to 70·0)	66·9 (63·5 to 69·6)	65·5 (62·2 to 68·3)	67·0 (63·6 to 69·9)	67·9 (64·4 to 70·8)	61	2·9 (2·0 to 3·9)	4·5 (3·2 to 5·9)
Bulgaria	64·6 (61·7 to 67·0)	66·8 (63·8 to 69·4)	66·7 (63·3 to 69·3)	64·9 (61·6 to 67·5)	66·7 (63·4 to 69·5)	67·9 (64·5 to 70·7)	58	3·3 (2·6 to 4·0)	5·1 (4·1 to 6·0)
Croatia	65·7 (62·8 to 68·2)	68·5 (65·2 to 71·2)	68·7 (65·3 to 71·5)	68·0 (64·5 to 70·8)	68·4 (64·8 to 71·3)	69·2 (65·7 to 72·0)	42	3·5 (2·7 to 4·1)	5·3 (4·2 to 6·2)
Czechia	64·5 (61·5 to 67·1)	68·5 (65·2 to 71·3)	68·7 (65·1 to 71·7)	67·8 (64·3 to 70·8)	69·0 (65·4 to 72·1)	69·7 (66·0 to 72·7)	36	5·2 (4·4 to 5·9)	8·0 (7·0 to 8·9)
Estonia	64·3 (61·3 to 66·9)	68·4 (65·1 to 71·3)	69·9 (66·3 to 72·9)	69·0 (65·5 to 72·0)	69·3 (65·8 to 72·4)	69·9 (66·1 to 73·1)	31	5·6 (4·7 to 6·3)	8·7 (7·6 to 9·7)
Georgia	63·1 (60·2 to 65·5)	65·6 (62·5 to 68·2)	66·1 (62·8 to 68·9)	64·2 (60·9 to 66·9)	66·1 (62·8 to 69·0)	67·3 (63·9 to 70·3)	71	4·2 (3·2 to 5·2)	6·7 (5·1 to 8·2)
Hungary	62·9 (59·8 to 65·5)	66·9 (63·7 to 69·7)	67·3 (64·0 to 70·0)	66·2 (62·8 to 68·9)	67·3 (63·9 to 70·2)	67·9 (64·4 to 70·7)	59	5·0 (4·3 to 5·5)	8·0 (7·0 to 8·8)
Kazakhstan	61·7 (58·9 to 64·2)	63·1 (60·4 to 65·7)	64·9 (61·8 to 67·4)	63·7 (60·6 to 66·2)	66·3 (63·0 to 69·1)	66·9 (63·6 to 69·6)	76	5·2 (4·3 to 5·9)	8·4 (6·8 to 9·7)
Kyrgyzstan	60·6 (57·8 to 63·1)	63·2 (60·4 to 65·7)	64·1 (61·1 to 66·6)	63·8 (60·6 to 66·4)	65·8 (62·6 to 68·6)	66·4 (63·2 to 69·2)	87	5·8 (4·9 to 6·6)	9·5 (8·1 to 10·8)
Latvia	64·0 (61·0 to 66·6)	66·9 (63·5 to 69·6)	68·1 (64·7 to 71·1)	66·7 (63·3 to 69·5)	67·6 (64·2 to 70·5)	68·3 (64·7 to 71·3)	55	4·2 (3·4 to 4·9)	6·6 (5·4 to 7·6)
Lithuania	64·7 (61·7 to 67·5)	66·8 (63·5 to 69·6)	67·8 (64·3 to 70·7)	66·8 (63·2 to 69·7)	67·6 (64·0 to 70·5)	68·5 (65·0 to 71·6)	52	3·7 (2·9 to 4·4)	5·8 (4·7 to 6·7)
Moldova	61·5 (58·5 to 63·8)	64·1 (61·1 to 66·8)	65·5 (62·3 to 68·2)	64·6 (61·5 to 67·2)	67·2 (63·8 to 70·0)	68·0 (64·4 to 71·0)	57	6·5 (5·6 to 7·5)	10·6 (9·2 to 12·0)
Mongolia	56·3 (53·7 to 58·5)	62·1 (59·4 to 64·4)	66·4 (63·3 to 69·0)	64·6 (61·5 to 67·1)	66·2 (63·1 to 68·9)	66·9 (63·7 to 69·8)	77	10·6 (9·3 to 11·8)	18·9 (16·8 to 20·7)
Montenegro	65·8 (63·0 to 68·2)	66·9 (63·9 to 69·5)	66·7 (63·4 to 69·4)	65·2 (62·1 to 67·8)	67·1 (63·7 to 69·9)	67·7 (64·3 to 70·4)	67	1·9 (1·1 to 2·5)	2·9 (1·7 to 3·8)
North Macedonia	63·5 (60·7 to 65·9)	66·4 (63·4 to 68·9)	65·8 (62·8 to 68·4)	64·4 (61·2 to 66·9)	66·5 (63·3 to 69·1)	67·4 (64·1 to 70·1)	69	3·9 (3·1 to 4·7)	6·2 (5·1 to 7·3)
Poland	64·6 (61·5 to 67·2)	68·7 (65·4 to 71·5)	68·6 (65·1 to 71·5)	67·7 (64·1 to 70·5)	68·8 (65·2 to 71·8)	69·5 (65·8 to 72·6)	38	4·9 (4·2 to 5·5)	7·6 (6·7 to 8·4)
Romania	63·3 (60·5 to 65·7)	67·1 (64·0 to 69·6)	67·4 (64·1 to 70·0)	66·0 (62·9 to 68·6)	67·9 (64·3 to 70·5)	68·6 (65·3 to 71·3)	47	5·3 (4·6 to 5·9)	8·4 (7·4 to 9·3)
Russia	63·5 (60·4 to 66·2)	64·1 (60·9 to 66·8)	64·9 (61·5 to 67·8)	63·4 (59·9 to 66·1)	65·7 (62·1 to 68·7)	66·5 (62·9 to 69·4)	86	2·9 (2·2 to 3·5)	4·6 (3·7 to 5·4)
Serbia	63·7 (61·1 to 65·9)	66·5 (63·6 to 68·9)	66·7 (63·7 to 69·2)	65·3 (62·4 to 67·8)	66·7 (63·7 to 69·3)	67·2 (64·2 to 69·9)	72	3·5 (2·8 to 4·4)	5·5 (4·4 to 6·7)
Slovakia	64·9 (61·9 to 67·4)	67·9 (64·5 to 70·5)	68·5 (65·0 to 71·3)	66·9 (63·6 to 69·6)	68·5 (64·9 to 71·4)	69·2 (65·7 to 72·2)	41	4·3 (3·5 to 4·9)	6·6 (5·5 to 7·5)
Slovenia	66·3 (63·2 to 69·0)	70·1 (66·7 to 73·0)	70·4 (66·8 to 73·4)	70·5 (66·8 to 73·5)	70·6 (66·9 to 73·7)	70·8 (67·1 to 74·0)	19	4·5 (3·6 to 5·3)	6·8 (5·7 to 7·7)
Tajikistan	59·9 (57·0 to 62·2)	63·5 (60·5 to 65·9)	63·1 (60·2 to 65·5)	62·0 (59·0 to 64·5)	62·4 (59·4 to 64·9)	62·9 (59·8 to 65·5)	130	3·0 (1·9 to 4·1)	5·0 (3·2 to 6·9)
Turkmenistan	60·0 (57·4 to 62·2)	63·0 (60·4 to 65·3)	62·3 (59·6 to 64·7)	61·7 (58·9 to 64·2)	63·0 (60·1 to 65·9)	63·6 (60·5 to 66·4)	122	3·7 (2·7 to 4·8)	6·1 (4·4 to 8·0)
Ukraine	63·9 (60·8 to 66·5)	64·4 (61·5 to 67·0)	65·4 (62·2 to 68·1)	63·8 (60·6 to 66·5)	64·1 (60·8 to 67·2)	66·2 (62·7 to 69·4)	89	2·3 (1·4 to 3·2)	3·5 (2·2 to 4·9)
Uzbekistan	61·2 (58·4 to 63·8)	63·0 (60·6 to 65·5)	63·6 (60·6 to 66·1)	63·5 (60·5 to 66·1)	64·1 (60·9 to 66·6)	64·6 (61·5 to 67·1)	113	3·4 (2·5 to 4·0)	5·5 (4·2 to 6·6)
**High-income**	**67·3 (64·1 to 70·2)**	**69·9 (66·3 to 72·9)**	**69·4 (65·7 to 72·6)**	**69·1 (65·3 to 72·3)**	**69·2 (65·4 to 72·4)**	**69·5 (65·7 to 72·8)**		**2·2 (1·5 to 2·8)**	**3·2 (2·3 to 4·0)**
Andorra	70·8 (67·3 to 73·7)	72·4 (68·7 to 75·5)	71·5 (67·9 to 74·8)	71·7 (67·8 to 75·0)	71·6 (67·8 to 74·9)	72·1 (68·4 to 75·4)	7	1·3 (0·5 to 2·1)	1·8 (0·7 to 2·9)
Argentina	65·1 (62·1 to 67·7)	67·6 (64·4 to 70·2)	67·6 (64·3 to 70·5)	66·0 (62·9 to 68·9)	67·2 (63·8 to 70·1)	67·9 (64·6 to 70·8)	62	2·8 (2·0 to 3·4)	4·3 (3·2 to 5·2)
Australia	67·6 (64·2 to 70·6)	69·9 (66·1 to 73·3)	70·6 (66·6 to 74·2)	70·5 (66·5 to 74·0)	70·0 (65·9 to 73·6)	70·5 (66·4 to 74·0)	23	2·8 (1·9 to 3·6)	4·2 (3·0 to 5·2)
Austria	67·9 (64·7 to 70·5)	70·7 (67·3 to 73·6)	70·7 (66·9 to 73·7)	70·6 (66·8 to 73·6)	70·7 (67·0 to 73·8)	71·0 (67·2 to 74·1)	18	3·1 (2·3 to 3·7)	4·6 (3·5 to 5·4)
Belgium	67·8 (64·8 to 70·6)	69·7 (66·2 to 72·7)	69·5 (65·7 to 72·6)	70·0 (66·2 to 73·2)	69·8 (66·0 to 73·1)	70·3 (66·5 to 73·5)	27	2·5 (1·7 to 3·2)	3·6 (2·5 to 4·6)
Brunei	64·3 (61·6 to 66·6)	67·8 (64·8 to 70·3)	68·7 (65·7 to 71·3)	68·5 (65·4 to 71·0)	68·2 (65·1 to 70·9)	68·5 (65·5 to 71·1)	49	4·2 (3·5 to 4·8)	6·5 (5·5 to 7·5)
Canada	67·5 (63·9 to 70·6)	69·2 (65·5 to 72·5)	68·9 (65·1 to 72·4)	68·8 (64·9 to 72·3)	68·5 (64·7 to 72·0)	68·8 (64·8 to 72·2)	45	1·3 (0·6 to 2·0)	1·9 (0·8 to 2·9)
Chile	65·5 (62·3 to 68·3)	69·3 (66·0 to 72·4)	69·2 (65·7 to 72·4)	68·7 (65·2 to 71·9)	69·1 (65·5 to 72·3)	69·6 (65·8 to 72·9)	37	4·1 (3·2 to 4·8)	6·2 (5·1 to 7·1)
Cyprus	66·9 (63·6 to 69·3)	70·1 (66·9 to 72·8)	69·9 (66·5 to 72·8)	69·5 (66·0 to 72·3)	69·5 (66·2 to 72·5)	69·9 (66·3 to 72·9)	33	3·0 (1·9 to 4·1)	4·5 (2·9 to 6·0)
Denmark	66·8 (63·7 to 69·4)	69·6 (66·3 to 72·4)	70·7 (67·2 to 73·7)	70·6 (67·1 to 73·6)	70·3 (66·8 to 73·4)	70·6 (67·1 to 73·7)	22	3·8 (3·0 to 4·4)	5·7 (4·7 to 6·5)
Finland	68·1 (65·0 to 70·7)	70·8 (67·4 to 73·7)	71·6 (68·0 to 74·6)	71·5 (67·9 to 74·5)	70·9 (67·2 to 73·8)	71·2 (67·5 to 74·3)	15	3·1 (2·3 to 3·7)	4·5 (3·6 to 5·4)
France	69·6 (66·5 to 72·4)	71·6 (68·1 to 74·8)	71·4 (67·7 to 74·6)	71·6 (67·7 to 75·0)	71·3 (67·4 to 74·6)	71·4 (67·5 to 74·8)	12	1·8 (1·0 to 2·5)	2·6 (1·5 to 3·5)
Germany	67·1 (63·8 to 69·9)	69·8 (66·0 to 72·9)	70·0 (66·3 to 73·2)	69·7 (66·1 to 72·9)	69·6 (66·1 to 72·7)	69·9 (66·2 to 73·0)	32	2·8 (2·0 to 3·5)	4·2 (3·1 to 5·0)
Greece	68·2 (65·2 to 70·8)	70·3 (67·0 to 73·3)	70·5 (66·8 to 73·6)	69·8 (66·1 to 72·8)	69·8 (66·1 to 73·0)	70·3 (66·5 to 73·4)	28	2·1 (1·3 to 2·8)	3·1 (2·0 to 4·0)
Greenland	56·7 (53·8 to 59·1)	59·8 (56·8 to 62·5)	60·3 (57·2 to 63·2)	60·0 (56·8 to 62·9)	60·8 (57·4 to 63·7)	61·4 (57·9 to 64·3)	139	4·7 (3·7 to 5·6)	8·2 (6·8 to 9·7)
Iceland	68·7 (65·8 to 71·5)	70·7 (67·4 to 73·7)	70·7 (67·1 to 73·9)	70·6 (67·0 to 73·9)	70·2 (66·5 to 73·5)	70·4 (66·8 to 73·7)	25	1·7 (1·0 to 2·3)	2·4 (1·4 to 3·3)
Ireland	67·1 (64·1 to 69·6)	70·5 (67·1 to 73·4)	70·8 (67·1 to 74·0)	70·7 (66·9 to 73·9)	70·5 (66·7 to 73·7)	70·7 (66·8 to 73·9)	21	3·6 (2·6 to 4·4)	5·3 (4·0 to 6·4)
Israel	68·2 (65·1 to 70·8)	71·4 (67·9 to 74·3)	72·0 (68·4 to 75·1)	71·8 (68·2 to 74·9)	72·0 (68·4 to 75·2)	72·2 (68·4 to 75·4)	6	4·0 (3·2 to 4·7)	5·9 (4·8 to 6·8)
Italy	68·9 (65·7 to 71·6)	72·2 (68·8 to 75·1)	71·8 (68·1 to 74·8)	71·8 (68·1 to 74·8)	71·6 (68·0 to 74·7)	71·9 (68·2 to 75·0)	8	3·0 (2·4 to 3·6)	4·4 (3·5 to 5·1)
Japan	70·6 (67·3 to 73·4)	73·6 (69·8 to 76·7)	74·6 (71·0 to 77·7)	74·4 (70·7 to 77·5)	74·0 (70·3 to 77·1)	73·9 (70·2 to 77·1)	2	3·3 (2·8 to 3·9)	4·7 (4·0 to 5·3)

Luxembourg	67·6 (64·5 to 70·3)	70·9 (67·4 to 73·9)	71·4 (67·8 to 74·5)	71·4 (67·8 to 74·7)	71·5 (67·7 to 74·7)	71·6 (67·7 to 74·9)	10	4·0 (3·1 to 4·8)	6·0 (4·8 to 7·0)
Malta	67·8 (64·8 to 70·3)	71·0 (67·8 to 73·8)	71·4 (67·9 to 74·5)	71·2 (67·5 to 74·2)	71·1 (67·6 to 74·4)	71·5 (67·9 to 74·7)	11	3·7 (2·7 to 4·5)	5·4 (4·2 to 6·5)
Monaco	68·8 (65·6 to 71·4)	70·3 (67·0 to 73·3)	70·3 (67·0 to 73·4)	69·7 (66·3 to 72·9)	69·8 (66·3 to 72·6)	70·2 (66·4 to 73·2)	29	1·4 (0·3 to 2·3)	2·0 (0·4 to 3·3)
Netherlands	68·2 (64·9 to 71·0)	68·1 (64·2 to 71·5)	69·0 (65·2 to 72·3)	68·7 (64·8 to 72·0)	68·7 (64·8 to 72·1)	68·9 (65·0 to 72·4)	44	0·7 (-0·2 to 1·5)	1·0 (-0·3 to 2·1)
New Zealand	65·5 (62·0 to 68·6)	68·9 (65·2 to 72·2)	69·4 (65·5 to 72·9)	69·3 (65·3 to 72·7)	68·7 (65·0 to 72·1)	68·7 (64·8 to 72·2)	46	3·2 (2·5 to 3·8)	4·8 (4·0 to 5·7)
Norway	68·1 (64·8 to 70·9)	70·5 (67·0 to 73·5)	71·2 (67·5 to 74·4)	71·0 (67·2 to 74·2)	70·6 (66·9 to 73·8)	70·8 (66·9 to 74·0)	20	2·7 (1·9 to 3·3)	4·0 (2·8 to 4·7)
Portugal	66·7 (63·4 to 69·3)	70·6 (67·1 to 73·6)	71·0 (67·2 to 74·1)	70·9 (67·1 to 74·0)	70·9 (67·0 to 74·0)	71·2 (67·3 to 74·4)	14	4·5 (3·6 to 5·3)	6·8 (5·5 to 7·8)
San Marino	70·4 (67·2 to 73·1)	73·6 (69·9 to 76·7)	72·2 (68·3 to 75·6)	72·5 (68·5 to 75·9)	72·1 (68·1 to 75·4)	72·8 (69·1 to 76·2)	4	2·4 (1·4 to 3·6)	3·4 (2·0 to 5·1)
Singapore	68·6 (65·5 to 70·9)	74·0 (70·8 to 76·7)	75·9 (72·4 to 78·8)	75·4 (72·1 to 78·3)	74·8 (71·4 to 77·7)	75·4 (72·0 to 78·3)	1	6·9 (6·1 to 7·6)	10·0 (9·1 to 10·8)
South Korea	65·4 (62·3 to 68·0)	72·1 (68·7 to 74·8)	73·7 (70·1 to 76·7)	73·6 (70·1 to 76·7)	72·9 (69·5 to 75·9)	73·4 (69·9 to 76·5)	3	8·0 (7·3 to 8·7)	12·2 (11·3 to 13·1)
Spain	68·7 (65·6 to 71·6)	72·4 (69·0 to 75·4)	71·8 (68·3 to 74·9)	72·1 (68·5 to 75·3)	71·8 (68·2 to 75·1)	72·2 (68·5 to 75·4)	5	3·5 (2·7 to 4·2)	5·0 (4·1 to 5·9)
Sweden	69·8 (66·8 to 72·4)	71·6 (68·5 to 74·4)	71·2 (67·7 to 74·2)	71·4 (67·8 to 74·5)	71·3 (67·7 to 74·4)	71·3 (67·7 to 74·5)	13	1·5 (0·7 to 2·3)	2·2 (1·0 to 3·2)
Switzerland	69·2 (65·9 to 72·0)	71·5 (67·9 to 74·5)	71·3 (67·5 to 74·6)	71·5 (67·5 to 74·9)	71·4 (67·3 to 74·7)	71·9 (67·7 to 75·2)	9	2·7 (1·8 to 3·4)	3·9 (2·7 to 4·8)
UK	67·2 (64·1 to 69·9)	69·4 (65·7 to 72·5)	68·6 (64·9 to 71·8)	68·5 (64·7 to 71·8)	68·6 (64·8 to 72·0)	68·6 (64·8 to 71·9)	48	1·4 (0·5 to 2·2)	2·1 (0·8 to 3·2)
USA	65·2 (61·7 to 68·4)	66·7 (63·0 to 70·0)	65·0 (61·3 to 68·3)	64·5 (60·7 to 67·8)	65·1 (61·2 to 68·5)	65·5 (61·6 to 69·1)	97	0·3 (-0·3 to 0·8)	0·5 (-0·4 to 1·2)
Uruguay	65·8 (62·8 to 68·5)	68·3 (65·1 to 71·1)	68·8 (65·3 to 71·7)	66·9 (63·6 to 69·7)	67·4 (64·0 to 70·4)	68·0 (64·7 to 71·0)	56	2·2 (1·5 to 2·8)	3·3 (2·2 to 4·3)
**Latin America and Caribbean**	**61·5 (58·2 to 64·2)**	**64·2 (60·9 to 67·1)**	**64·4 (60·9 to 67·3)**	**63·0 (59·7 to 65·9)**	**65·0 (61·4 to 68·1)**	**65·4 (61·7 to 68·6)**		**4·0 (3·3 to 4·6)**	**6·5 (5·5 to 7·3)**
Antigua and Barbuda	65·0 (61·7 to 67·7)	67·9 (64·4 to 70·9)	67·2 (63·8 to 70·2)	66·4 (63·0 to 69·3)	66·7 (63·1 to 69·7)	67·0 (63·2 to 70·0)	74	2·1 (1·1 to 3·0)	3·2 (1·8 to 4·6)
Bahamas	63·8 (60·6 to 66·4)	64·4 (61·1 to 67·1)	63·4 (60·0 to 66·1)	60·5 (57·3 to 63·1)	63·3 (59·8 to 66·2)	64·1 (60·4 to 66·9)	117	0·3 (-0·6 to 1·1)	0·5 (-1·0 to 1·8)
Barbados	65·3 (62·3 to 68·1)	67·4 (64·0 to 70·3)	66·7 (63·3 to 69·5)	65·9 (62·6 to 68·7)	65·9 (62·5 to 68·8)	66·6 (63·0 to 69·6)	82	1·2 (0·3 to 2·1)	1·9 (0·5 to 3·1)
Belize	64·3 (61·1 to 66·9)	64·8 (61·4 to 67·5)	64·3 (61·0 to 67·3)	62·8 (59·6 to 65·7)	64·3 (60·8 to 67·3)	65·4 (61·7 to 68·4)	102	1·1 (0·2 to 2·0)	1·7 (0·2 to 3·1)
Bermuda	66·9 (63·7 to 69·6)	70·9 (67·4 to 73·8)	71·1 (67·5 to 74·1)	70·1 (66·5 to 73·1)	69·9 (66·4 to 73·0)	70·3 (66·6 to 73·5)	26	3·4 (2·7 to 4·2)	5·1 (4·0 to 6·2)
Bolivia	55·8 (52·9 to 58·3)	61·3 (58·5 to 63·9)	58·4 (55·6 to 60·8)	58·4 (55·5 to 60·9)	61·8 (58·6 to 64·7)	62·8 (59·6 to 65·7)	132	7·0 (6·0 to 7·9)	12·5 (10·8 to 14·0)
Brazil	61·5 (58·1 to 64·3)	65·7 (62·2 to 68·6)	65·3 (61·7 to 68·5)	63·7 (60·2 to 66·8)	65·5 (61·7 to 68·7)	65·8 (62·0 to 69·2)	95	4·4 (3·5 to 5·1)	7·1 (5·9 to 8·1)
Colombia	63·5 (60·1 to 66·2)	68·2 (64·8 to 71·2)	67·9 (64·3 to 70·9)	66·2 (62·6 to 69·2)	68·3 (64·6 to 71·5)	69·3 (65·6 to 72·5)	40	5·8 (4·9 to 6·6)	9·2 (7·8 to 10·3)
Costa Rica	66·3 (62·8 to 69·3)	68·7 (65·1 to 71·8)	68·1 (64·5 to 71·2)	66·9 (63·3 to 70·0)	67·5 (63·9 to 70·8)	67·9 (64·2 to 71·2)	60	1·6 (0·9 to 2·2)	2·4 (1·4 to 3·3)
Cuba	65·9 (62·6 to 68·6)	68·4 (64·9 to 71·4)	67·9 (64·3 to 70·8)	64·9 (61·6 to 67·5)	67·4 (63·8 to 70·3)	67·8 (64·2 to 71·0)	65	1·9 (1·0 to 2·8)	2·8 (1·5 to 4·3)
Dominica	65·3 (61·9 to 68·0)	66·1 (62·6 to 69·1)	65·8 (62·0 to 68·8)	64·9 (61·2 to 67·8)	65·9 (62·2 to 68·9)	66·1 (62·2 to 69·1)	90	0·9 (-0·1 to 1·7)	1·3 (-0·1 to 2·6)
Dominican Republic	62·4 (59·1 to 65·0)	66·0 (62·4 to 69·1)	64·0 (60·5 to 66·8)	63·8 (60·3 to 66·6)	63·9 (60·3 to 66·6)	64·3 (60·6 to 67·0)	115	1·9 (0·9 to 2·8)	3·1 (1·4 to 4·5)
Ecuador	62·6 (59·6 to 65·1)	67·1 (64·1 to 69·9)	65·8 (62·6 to 68·6)	65·9 (62·7 to 68·8)	67·2 (63·8 to 70·2)	67·4 (64·0 to 70·3)	70	4·8 (4·0 to 5·5)	7·7 (6·4 to 8·8)
El Salvador	62·1 (59·0 to 64·8)	65·9 (62·7 to 68·7)	64·4 (61·3 to 67·0)	63·5 (60·5 to 66·3)	64·7 (61·5 to 67·4)	65·0 (61·9 to 67·8)	109	2·9 (2·2 to 3·6)	4·6 (3·6 to 5·9)
Grenada	62·3 (59·3 to 65·0)	65·6 (62·5 to 68·4)	65·4 (61·9 to 68·3)	63·6 (60·4 to 66·8)	64·4 (61·2 to 67·4)	64·9 (61·8 to 67·9)	110	2·6 (1·6 to 3·7)	4·1 (2·6 to 5·7)
Guatemala	54·5 (51·6 to 56·9)	62·2 (58·8 to 65·1)	62·0 (58·7 to 64·8)	60·1 (56·9 to 62·8)	61·9 (58·3 to 64·8)	62·7 (59·2 to 65·9)	133	8·3 (7·1 to 9·5)	15·2 (13·0 to 17·1)
Guyana	57·6 (54·7 to 60·3)	60·0 (57·0 to 62·8)	61·3 (58·2 to 64·2)	59·1 (55·9 to 61·8)	60·3 (57·0 to 63·1)	60·9 (57·6 to 63·5)	145	3·3 (1·9 to 4·7)	5·7 (3·4 to 8·1)
Haiti	44·3 (42·1 to 46·2)	24·3 (22·6 to 26·0)	52·7 (50·0 to 55·2)	51·9 (49·1 to 54·3)	52·3 (49·4 to 54·7)	52·3 (49·6 to 54·8)	198	8·0 (6·7 to 9·3)	18·2 (15·0 to 21·3)
Honduras	61·7 (58·6 to 64·3)	63·9 (60·9 to 66·8)	62·8 (59·8 to 65·6)	61·3 (58·1 to 64·0)	64·4 (61·0 to 67·5)	65·0 (61·6 to 68·1)	108	3·3 (2·5 to 4·1)	5·4 (4·1 to 6·6)
Jamaica	65·0 (61·6 to 67·7)	67·1 (63·6 to 70·0)	65·7 (62·2 to 68·7)	64·3 (60·8 to 67·3)	65·6 (62·0 to 68·7)	66·3 (62·5 to 69·3)	88	1·3 (0·3 to 2·4)	2·0 (0·4 to 3·8)
Mexico	61·4 (58·0 to 64·4)	65·0 (61·5 to 68·1)	62·7 (59·2 to 65·6)	62·0 (58·5 to 65·0)	64·4 (60·7 to 67·6)	65·1 (61·1 to 68·3)	107	3·7 (3·0 to 4·3)	6·0 (5·2 to 6·7)
Nicaragua	62·3 (58·9 to 64·9)	65·8 (62·7 to 68·5)	63·8 (60·7 to 66·6)	62·9 (59·9 to 65·6)	65·2 (61·7 to 68·2)	66·1 (62·5 to 69·3)	91	3·8 (2·9 to 4·9)	6·1 (4·6 to 7·9)
Panama	66·6 (63·3 to 69·5)	68·5 (65·1 to 71·4)	67·0 (63·6 to 70·0)	66·9 (63·5 to 70·0)	67·8 (64·3 to 71·0)	68·3 (64·8 to 71·5)	54	1·7 (1·1 to 2·3)	2·6 (1·7 to 3·4)
Paraguay	64·9 (61·5 to 67·7)	65·0 (61·8 to 67·8)	63·9 (60·6 to 66·8)	60·0 (57·0 to 62·5)	63·6 (60·3 to 66·6)	63·9 (60·6 to 66·9)	119	-0·9 (-1·8 to -0·2)	-1·4 (-2·8 to -0·3)
Peru	63·0 (59·5 to 65·9)	68·4 (65·0 to 71·3)	64·6 (61·3 to 67·3)	62·9 (59·9 to 65·6)	67·2 (63·8 to 70·2)	66·5 (63·1 to 69·5)	83	3·6 (2·8 to 4·3)	5·7 (4·5 to 7·0)
Puerto Rico	66·9 (63·4 to 69·8)	69·7 (66·0 to 72·9)	70·6 (66·8 to 73·8)	70·4 (66·5 to 73·5)	70·1 (66·2 to 73·1)	70·4 (66·5 to 73·8)	24	3·6 (2·7 to 4·4)	5·3 (4·1 to 6·5)
Saint Kitts and Nevis	61·0 (57·7 to 63·7)	65·5 (62·2 to 68·2)	65·3 (62·0 to 68·1)	64·5 (61·1 to 67·3)	65·1 (61·5 to 68·1)	65·5 (61·9 to 68·4)	100	4·5 (3·5 to 5·5)	7·3 (5·7 to 8·9)
Saint Lucia	61·8 (58·5 to 64·6)	66·2 (62·5 to 69·4)	66·9 (63·2 to 70·1)	65·0 (61·5 to 67·9)	66·4 (62·5 to 69·6)	67·0 (63·2 to 70·3)	75	5·1 (3·8 to 6·4)	8·3 (6·2 to 10·2)
Saint Vincent and the Grenadines	62·7 (59·4 to 65·5)	64·4 (60·9 to 67·3)	65·2 (61·5 to 68·1)	64·9 (61·3 to 68·0)	65·3 (61·1 to 68·5)	65·5 (61·9 to 68·7)	98	2·8 (1·5 to 4·0)	4·4 (2·5 to 6·3)
Suriname	62·3 (59·0 to 64·9)	63·7 (60·3 to 66·5)	63·3 (59·7 to 66·1)	60·2 (56·9 to 62·9)	62·7 (59·1 to 65·6)	63·4 (59·6 to 66·4)	125	1·1 (0·2 to 2·0)	1·8 (0·3 to 3·2)
Trinidad and Tobago	60·5 (57·2 to 63·1)	64·1 (60·4 to 66·9)	64·0 (60·3 to 66·9)	61·6 (58·2 to 64·3)	62·8 (59·5 to 65·7)	63·8 (60·3 to 66·7)	120	3·4 (2·4 to 4·2)	5·6 (4·0 to 7·0)
Venezuela	63·2 (59·9 to 65·8)	66·4 (63·2 to 69·4)	64·0 (60·7 to 66·9)	63·1 (59·7 to 66·0)	63·3 (60·0 to 66·2)	63·3 (59·9 to 66·3)	126	0·2 (-0·6 to 1·0)	0·3 (-0·9 to 1·5)
Virgin Islands	64·5 (61·4 to 67·1)	68·9 (65·2 to 71·9)	70·2 (66·4 to 73·3)	69·0 (65·2 to 71·9)	69·1 (65·6 to 72·2)	68·9 (65·3 to 72·0)	43	4·4 (3·1 to 5·4)	6·8 (4·9 to 8·4)
**North Africa and Middle East**	**56·5 (53·6 to 59·1)**	**62·3 (59·1 to 65·1)**	**61·7 (58·4 to 64·6)**	**61·2 (57·9 to 64·1)**	**62·6 (59·1 to 65·5)**	**62·8 (59·3 to 65·8)**		**6·3 (5·4 to 7·0)**	**11·1 (9·8 to 12·4)**
Afghanistan	48·0 (44·0 to 51·2)	53·8 (49·9 to 56·9)	53·7 (49·7 to 56·9)	52·0 (48·3 to 55·0)	55·3 (51·8 to 58·7)	55·9 (52·1 to 59·2)	180	7·9 (6·3 to 9·5)	16·5 (13·0 to 21·0)
Algeria	59·3 (56·2 to 61·8)	64·1 (60·6 to 66·8)	64·2 (60·7 to 67·0)	64·3 (60·9 to 67·2)	65·0 (61·4 to 67·9)	65·3 (61·7 to 68·2)	104	6·0 (5·1 to 6·8)	10·1 (8·6 to 11·4)
Bahrain	60·2 (56·9 to 62·8)	64·0 (60·4 to 66·9)	64·8 (61·2 to 67·9)	64·3 (60·9 to 67·2)	65·6 (61·7 to 68·9)	65·9 (62·0 to 69·1)	94	5·7 (4·4 to 6·8)	9·5 (7·3 to 11·3)

Egypt	54·7 (52·0 to 57·1)	59·3 (56·5 to 61·9)	59·2 (56·0 to 61·8)	58·2 (55·1 to 60·8)	59·4 (56·2 to 62·0)	59·9 (56·7 to 62·7)	153	5·2 (4·0 to 6·3)	9·5 (7·1 to 11·5)
Iran	56·8 (53·7 to 59·5)	65·4 (61·9 to 68·5)	63·5 (60·0 to 66·7)	63·6 (60·0 to 66·8)	65·9 (62·0 to 69·3)	66·5 (62·4 to 70·0)	84	9·7 (8·5 to 10·7)	17·0 (15·5 to 18·4)
Iraq	59·0 (55·8 to 61·8)	60·5 (57·3 to 63·2)	59·5 (56·2 to 62·3)	59·8 (56·4 to 62·8)	61·4 (58·0 to 64·4)	61·9 (58·5 to 64·9)	135	2·8 (2·0 to 3·7)	4·8 (3·4 to 6·3)
Jordan	62·5 (59·2 to 65·2)	64·6 (61·3 to 67·5)	64·4 (61·1 to 67·3)	62·8 (59·6 to 65·7)	65·9 (62·3 to 69·1)	66·9 (63·2 to 70·1)	78	4·4 (3·5 to 5·2)	7·0 (5·7 to 8·2)
Kuwait	62·9 (59·7 to 65·6)	66·1 (62·8 to 69·1)	66·3 (62·7 to 69·3)	65·9 (62·3 to 69·1)	67·4 (63·6 to 70·7)	67·8 (64·0 to 71·2)	63	4·9 (3·9 to 5·9)	7·9 (6·5 to 9·2)
Lebanon	63·9 (60·0 to 67·1)	68·3 (64·4 to 71·7)	63·6 (60·0 to 66·7)	65·9 (62·1 to 69·2)	66·2 (62·1 to 69·7)	66·7 (62·7 to 70·1)	79	2·8 (1·6 to 4·2)	4·3 (2·4 to 6·9)
Libya	61·7 (58·3 to 64·4)	63·7 (60·2 to 66·5)	61·7 (58·1 to 64·6)	59·4 (56·2 to 62·3)	62·9 (59·2 to 66·1)	58·5 (55·1 to 61·4)	163	-3·2 (-4·2 to -2·4)	-5·2 (-6·8 to -3·9)
Morocco	55·5 (52·7 to 57·9)	60·2 (57·1 to 62·8)	60·4 (57·3 to 63·0)	60·2 (57·0 to 62·9)	61·2 (57·8 to 64·0)	61·6 (58·5 to 64·5)	136	6·1 (4·9 to 7·4)	11·1 (8·7 to 13·2)
Oman	60·6 (57·4 to 63·6)	65·3 (61·9 to 68·3)	64·5 (61·2 to 67·6)	63·4 (60·1 to 66·4)	65·9 (62·3 to 69·1)	66·5 (62·8 to 69·7)	85	5·9 (4·9 to 6·8)	9·7 (8·0 to 11·3)
Palestine	61·6 (58·1 to 64·4)	64·1 (60·6 to 67·1)	64·9 (61·4 to 68·0)	63·7 (60·1 to 66·6)	65·3 (61·5 to 68·2)	60·0 (56·6 to 62·8)	152	-1·6 (-2·5 to -0·7)	-2·5 (-4·0 to -1·2)
Qatar	61·3 (58·1 to 64·1)	65·5 (61·9 to 68·5)	67·9 (64·0 to 71·4)	67·9 (64·1 to 71·3)	68·5 (64·4 to 72·0)	68·5 (64·6 to 72·0)	50	7·2 (6·0 to 8·3)	11·7 (10·0 to 13·5)
Saudi Arabia	60·2 (56·8 to 62·9)	62·7 (59·4 to 65·5)	61·8 (58·2 to 64·6)	62·2 (58·6 to 65·2)	62·6 (59·1 to 65·6)	63·0 (59·2 to 66·0)	129	2·8 (1·7 to 3·8)	4·7 (2·9 to 6·3)
Sudan	48·6 (45·8 to 50·7)	56·9 (53·8 to 59·3)	58·0 (54·8 to 60·8)	58·2 (54·9 to 60·9)	59·2 (55·6 to 62·0)	59·4 (56·0 to 62·5)	156	10·8 (9·4 to 12·3)	22·3 (19·1 to 25·9)
Syria	59·5 (56·0 to 62·3)	63·7 (60·2 to 66·7)	62·8 (58·9 to 66·2)	62·4 (58·5 to 65·8)	63·7 (59·8 to 67·2)	63·1 (59·1 to 66·4)	128	3·6 (2·5 to 4·5)	6·0 (4·3 to 7·6)
Tunisia	60·6 (57·7 to 63·1)	64·9 (61·8 to 67·7)	63·6 (60·5 to 66·5)	61·6 (58·6 to 64·4)	64·7 (61·3 to 67·8)	65·2 (61·6 to 68·3)	105	4·7 (3·8 to 5·3)	7·7 (6·4 to 8·7)
Türkiye	60·0 (56·9 to 62·7)	67·1 (63·7 to 70·1)	66·2 (62·8 to 69·3)	65·3 (61·9 to 68·3)	66·5 (62·9 to 69·6)	65·5 (61·7 to 68·6)	101	5·5 (4·4 to 6·4)	9·1 (7·4 to 10·7)
United Arab Emirates	61·1 (58·0 to 63·8)	65·3 (62·1 to 68·3)	66·2 (62·7 to 69·2)	66·0 (62·5 to 69·0)	66·4 (62·9 to 69·5)	66·6 (63·0 to 69·7)	81	5·5 (4·5 to 6·5)	9·0 (7·5 to 10·4)
Yemen	51·7 (48·4 to 54·5)	59·3 (55·7 to 62·6)	59·2 (55·5 to 62·4)	59·1 (55·5 to 62·4)	60·2 (56·4 to 63·7)	60·7 (56·7 to 63·8)	146	8·9 (7·6 to 10·3)	17·3 (14·8 to 20·0)
**South Asia**	**49·7 (46·8 to 51·9)**	**58·1 (55·0 to 60·8)**	**59·8 (56·5 to 62·6)**	**59·0 (55·7 to 61·8)**	**60·3 (56·9 to 63·1)**	**60·6 (57·1 to 63·5)**		**11·0 (9·9 to 12·0)**	**22·1 (20·1 to 23·8)**
Bangladesh	47·6 (44·9 to 49·8)	58·9 (56·0 to 61·5)	59·7 (56·5 to 62·2)	58·5 (55·5 to 61·1)	60·2 (57·2 to 62·9)	60·4 (57·3 to 63·0)	150	12·7 (11·5 to 14·0)	26·7 (24·4 to 29·4)
Bhutan	50·1 (47·4 to 52·4)	59·1 (56·1 to 61·7)	61·4 (58·3 to 63·9)	61·6 (58·3 to 64·2)	61·4 (58·1 to 64·1)	61·5 (58·3 to 64·2)	138	11·5 (10·1 to 12·9)	22·9 (20·3 to 26·1)
India	49·5 (46·7 to 51·9)	58·3 (55·1 to 60·9)	60·3 (57·0 to 63·1)	59·5 (56·2 to 62·4)	60·6 (57·2 to 63·5)	61·0 (57·4 to 63·9)	144	11·4 (10·2 to 12·7)	23·1 (20·8 to 25·2)
Nepal	48·8 (45·9 to 51·5)	61·4 (58·3 to 64·1)	63·0 (59·7 to 65·9)	61·6 (58·5 to 64·3)	63·2 (60·1 to 66·2)	63·4 (60·3 to 66·2)	124	14·6 (13·3 to 15·8)	29·9 (27·2 to 32·3)
Pakistan	53·0 (50·2 to 55·5)	56·6 (53·6 to 59·2)	56·4 (53·1 to 59·4)	55·7 (52·5 to 58·5)	58·1 (54·4 to 61·1)	58·7 (55·1 to 61·6)	160	5·7 (4·4 to 7·1)	10·7 (8·3 to 13·3)
**Southeast Asia, East Asia, and Oceania**	**60·2 (57·8 to 62·4)**	**66·8 (64·0 to 69·2)**	**69·4 (66·4 to 72·0)**	**68·6 (65·6 to 71·2)**	**68·9 (65·7 to 71·7)**	**68·9 (65·7 to 71·7)**		**8·6 (7·8 to 9·4)**	**14·3 (13·2 to 15·3)**
American Samoa	63·6 (60·8 to 66·2)	62·8 (59·8 to 65·2)	63·5 (60·5 to 66·1)	62·2 (59·1 to 64·7)	63·6 (60·5 to 66·4)	63·7 (60·8 to 66·4)	121	0·1 (-1·0 to 1·1)	0·2 (-1·5 to 1·7)
Cambodia	52·3 (49·6 to 54·6)	62·6 (59·6 to 65·0)	65·1 (62·1 to 67·6)	64·8 (61·7 to 67·5)	64·8 (61·5 to 67·4)	64·6 (61·6 to 67·1)	112	12·3 (11·1 to 13·6)	23·6 (21·3 to 25·9)
China	60·6 (58·2 to 62·8)	68·2 (65·3 to 70·7)	71·5 (68·3 to 74·2)	71·3 (68·1 to 74·1)	71·2 (67·8 to 74·0)	71·0 (67·7 to 73·8)	17	10·4 (9·4 to 11·4)	17·2 (15·7 to 18·6)
Cook Islands	62·0 (59·4 to 64·2)	65·2 (62·2 to 67·7)	65·5 (62·6 to 68·1)	64·6 (61·6 to 67·2)	65·3 (62·2 to 68·1)	65·5 (62·3 to 68·3)	99	3·5 (2·7 to 4·3)	5·7 (4·3 to 6·9)
Federated States of Micronesia	59·4 (57·0 to 61·5)	60·9 (58·4 to 63·2)	61·0 (58·1 to 63·4)	59·9 (57·1 to 62·2)	60·8 (57·9 to 63·1)	61·0 (58·1 to 63·3)	142	1·6 (1·0 to 2·4)	2·7 (1·6 to 4·0)
Fiji	60·9 (58·1 to 63·2)	59·8 (57·2 to 62·2)	60·2 (57·2 to 62·5)	58·6 (55·8 to 60·9)	59·6 (56·7 to 62·0)	59·6 (56·7 to 62·1)	155	-1·3 (-2·1 to -0·4)	-2·1 (-3·4 to -0·8)
Guam	67·4 (64·7 to 69·7)	69·6 (66·6 to 72·2)	68·3 (65·0 to 70·9)	67·4 (64·3 to 69·9)	68·1 (64·8 to 70·7)	68·4 (65·2 to 71·1)	53	1·0 (0·2 to 1·7)	1·5 (0·3 to 2·5)
Indonesia	58·2 (55·4 to 60·7)	63·2 (60·4 to 65·7)	64·2 (61·4 to 66·7)	62·1 (59·0 to 64·6)	64·5 (61·3 to 67·2)	64·5 (61·5 to 67·3)	114	6·3 (5·2 to 7·4)	10·8 (9·0 to 12·8)
Kiribati	58·9 (55·9 to 61·3)	61·7 (58·7 to 64·2)	62·6 (59·4 to 65·3)	61·6 (58·3 to 64·2)	62·6 (59·2 to 65·4)	62·9 (59·4 to 65·8)	131	4·0 (3·0 to 4·8)	6·7 (5·3 to 8·2)
Laos	46·1 (44·0 to 48·0)	57·8 (55·4 to 59·8)	59·2 (56·9 to 61·4)	59·1 (56·8 to 61·2)	58·8 (56·5 to 61·3)	59·0 (56·4 to 61·3)	158	12·9 (11·7 to 14·3)	27·9 (24·8 to 31·0)
Malaysia	64·2 (61·8 to 66·4)	67·0 (64·3 to 69·4)	68·1 (65·1 to 70·7)	65·5 (62·8 to 68·0)	66·8 (64·0 to 69·4)	67·7 (64·9 to 70·3)	66	3·5 (2·8 to 4·0)	5·4 (4·4 to 6·3)
Maldives	56·1 (53·5 to 58·2)	65·9 (63·1 to 68·2)	67·0 (64·0 to 69·3)	66·1 (63·3 to 68·4)	66·6 (63·7 to 69·0)	67·1 (64·2 to 69·5)	73	11·0 (10·1 to 11·7)	19·5 (17·9 to 20·8)
Marshall Islands	56·6 (54·2 to 58·7)	56·5 (53·9 to 58·6)	56·5 (53·9 to 58·6)	55·4 (52·8 to 57·3)	56·4 (53·8 to 58·4)	56·5 (53·9 to 58·6)	177	-0·1 (-0·8 to 0·6)	-0·2 (-1·4 to 1·0)
Mauritius	63·7 (60·7 to 66·1)	66·3 (63·2 to 69·0)	66·5 (63·2 to 69·2)	65·2 (62·1 to 67·9)	65·5 (62·3 to 68·1)	66·0 (62·7 to 68·7)	93	2·3 (1·6 to 3·1)	3·6 (2·6 to 4·8)
Myanmar	51·2 (48·8 to 53·3)	58·7 (56·3 to 61·2)	60·5 (57·9 to 62·9)	59·3 (56·7 to 61·7)	60·2 (57·6 to 62·6)	60·2 (57·5 to 62·7)	151	9·0 (7·8 to 10·1)	17·5 (15·1 to 19·8)
Nauru	54·0 (51·8 to 55·8)	52·2 (50·2 to 54·1)	53·1 (50·9 to 55·0)	51·3 (49·2 to 53·2)	53·1 (50·9 to 55·1)	53·5 (51·1 to 55·6)	193	-0·5 (-1·3 to 0·2)	-0·9 (-2·5 to 0·3)
Niue	63·9 (61·4 to 66·3)	64·8 (62·1 to 67·2)	65·7 (62·8 to 68·0)	64·6 (61·8 to 66·9)	64·6 (61·8 to 67·0)	64·8 (62·0 to 67·3)	111	0·9 (0·3 to 1·5)	1·4 (0·5 to 2·3)
North Korea	65·0 (62·2 to 67·5)	65·5 (62·7 to 67·9)	65·8 (62·8 to 68·2)	65·9 (62·8 to 68·4)	65·7 (62·5 to 68·3)	65·7 (62·6 to 68·3)	96	0·6 (-0·4 to 1·7)	1·0 (-0·6 to 2·7)
Northern Mariana Islands	62·7 (60·1 to 64·9)	63·1 (60·5 to 65·4)	62·9 (60·3 to 65·2)	61·5 (59·0 to 63·7)	62·7 (60·1 to 65·1)	63·1 (60·5 to 65·5)	127	0·4 (-0·2 to 1·0)	0·7 (-0·3 to 1·6)
Palau	60·3 (57·7 to 62·4)	60·7 (58·0 to 63·0)	60·4 (57·8 to 62·7)	59·3 (56·7 to 61·5)	60·4 (57·7 to 62·7)	60·6 (57·8 to 62·9)	147	0·3 (-0·4 to 1·0)	0·6 (-0·7 to 1·6)
Papua New Guinea	55·3 (52·7 to 57·6)	57·0 (54·5 to 59·2)	58·4 (55·7 to 60·7)	58·0 (55·5 to 60·4)	57·9 (55·2 to 60·4)	58·0 (55·4 to 60·3)	166	2·7 (1·6 to 4·0)	5·0 (2·8 to 7·4)
Philippines	62·3 (59·5 to 64·7)	64·6 (61·9 to 66·9)	65·1 (62·3 to 67·5)	61·9 (59·4 to 64·1)	64·1 (61·4 to 66·5)	65·1 (62·3 to 67·6)	106	2·8 (2·1 to 3·4)	4·5 (3·3 to 5·5)
Samoa	64·3 (61·4 to 66·8)	64·8 (62·1 to 67·3)	64·2 (61·4 to 66·6)	63·1 (60·4 to 65·5)	64·0 (61·1 to 66·4)	64·0 (61·1 to 66·5)	118	-0·3 (-1·0 to 0·3)	-0·5 (-1·6 to 0·5)
Seychelles	65·8 (63·0 to 68·1)	67·4 (64·5 to 69·9)	68·5 (65·3 to 71·3)	64·9 (61·9 to 67·4)	67·3 (64·2 to 69·9)	67·8 (64·6 to 70·4)	64	2·0 (1·1 to 2·8)	3·0 (1·8 to 4·2)
Solomon Islands	57·8 (55·5 to 59·6)	59·6 (57·2 to 61·7)	58·1 (55·8 to 60·1)	57·8 (55·5 to 59·9)	57·8 (55·4 to 59·9)	57·9 (55·6 to 60·0)	171	0·1 (-0·5 to 0·8)	0·2 (-0·9 to 1·3)
Sri Lanka	63·9 (61·2 to 66·4)	67·2 (64·4 to 69·7)	69·6 (66·6 to 72·3)	68·3 (65·4 to 70·7)	68·4 (65·6 to 71·0)	68·5 (65·5 to 71·0)	51	4·6 (3·9 to 5·2)	7·2 (6·1 to 8·2)
Taiwan*	66·9 (64·0 to 69·3)	70·7 (67·4 to 73·5)	71·6 (68·2 to 74·5)	71·5 (68·0 to 74·4)	70·8 (67·4 to 73·7)	71·1 (67·6 to 74·0)	16	4·2 (3·4 to 5·0)	6·3 (5·2 to 7·2)
Thailand	65·0 (62·2 to 67·4)	67·9 (65·1 to 70·5)	70·3 (67·3 to 73·0)	69·6 (66·7 to 72·2)	69·4 (66·4 to 72·0)	70·0 (67·1 to 72·7)	30	5·0 (4·4 to 5·5)	7·7 (6·9 to 8·6)
Timor-Leste	50·4 (48·1 to 52·5)	60·4 (57·9 to 62·6)	60·8 (58·4 to 63·0)	60·6 (58·1 to 62·8)	60·9 (58·3 to 63·3)	61·0 (58·5 to 63·6)	143	10·6 (9·3 to 11·9)	21·0 (18·4 to 23·6)

Tokelau	63·8 (61·0 to 66·3)	64·9 (61·8 to 67·4)	66·1 (62·8 to 68·7)	65·3 (62·0 to 67·9)	65·3 (62·0 to 67·9)	65·4 (62·3 to 68·1)	103	1·6 (0·9 to 2·2)	2·5 (1·4 to 3·4)
Tonga	63·3 (60·4 to 65·8)	63·4 (60·4 to 66·0)	62·7 (59·7 to 65·3)	61·1 (58·2 to 63·6)	62·4 (59·3 to 65·0)	62·6 (59·3 to 65·3)	134	-0·7 (-1·5 to 0·0)	-1·2 (-2·4 to 0·0)
Tuvalu	54·9 (52·4 to 56·8)	57·5 (54·9 to 59·7)	58·0 (55·3 to 60·2)	57·0 (54·4 to 59·2)	57·8 (55·2 to 60·1)	58·0 (55·5 to 60·1)	169	3·1 (2·4 to 3·8)	5·6 (4·3 to 7·0)
Vanuatu	62·0 (59·4 to 64·3)	63·6 (60·8 to 65·8)	63·4 (60·6 to 65·7)	63·0 (60·3 to 65·4)	63·3 (60·6 to 65·7)	63·4 (60·7 to 65·8)	123	1·4 (0·6 to 2·1)	2·2 (1·0 to 3·4)
Viet Nam	63·5 (60·7 to 66·0)	68·2 (65·4 to 70·7)	69·4 (66·2 to 72·1)	68·8 (65·7 to 71·5)	69·2 (66·1 to 72·2)	69·4 (66·3 to 72·0)	39	5·9 (4·8 to 7·0)	9·3 (7·7 to 11·1)
**Sub-Saharan Africa**	**46·2 (43·9 to 48·3)**	**52·0 (49·4 to 54·2)**	**55·0 (52·2 to 57·4)**	**54·0 (51·3 to 56·4)**	**55·4 (52·6 to 57·9)**	**55·9 (53·1 to 58·4)**		**9·7 (8·9 to 10·4)**	**21·0 (19·5 to 22·3)**
Angola	45·3 (42·8 to 47·4)	53·9 (51·0 to 56·4)	56·1 (53·0 to 58·9)	55·2 (52·0 to 57·9)	56·5 (53·2 to 59·3)	56·8 (53·6 to 59·9)	174	11·5 (10·0 to 12·9)	25·5 (22·2 to 28·6)
Benin	48·6 (45·9 to 51·0)	55·8 (52·7 to 58·3)	57·7 (54·6 to 60·4)	57·9 (54·7 to 60·5)	58·2 (55·0 to 60·8)	58·5 (55·5 to 61·2)	162	9·9 (8·4 to 11·1)	20·3 (17·3 to 23·0)
Botswana	57·5 (54·7 to 60·0)	57·4 (54·2 to 60·1)	60·4 (57·2 to 63·1)	57·5 (54·5 to 59·9)	60·3 (57·1 to 63·1)	61·1 (57·9 to 63·8)	140	3·6 (2·6 to 4·4)	6·3 (4·7 to 7·6)
Burkina Faso	43·8 (41·4 to 46·1)	55·6 (52·9 to 58·0)	58·9 (55·9 to 61·7)	58·6 (55·5 to 61·7)	59·0 (55·9 to 62·1)	59·3 (56·2 to 62·2)	157	15·5 (14·0 to 16·9)	35·3 (31·6 to 39·1)
Burundi	43·5 (41·3 to 45·6)	52·7 (50·2 to 55·0)	54·7 (52·0 to 57·1)	53·3 (50·8 to 55·8)	54·5 (52·0 to 57·1)	54·8 (52·1 to 57·5)	186	11·2 (9·9 to 12·8)	25·8 (22·6 to 29·3)
Cabo Verde	61·7 (58·6 to 64·4)	66·9 (63·6 to 69·6)	65·4 (62·1 to 68·2)	64·2 (60·8 to 67·2)	65·9 (62·5 to 68·8)	66·7 (63·2 to 69·7)	80	5·0 (3·5 to 6·2)	8·1 (5·8 to 10·1)
Cameroon	48·1 (45·5 to 50·6)	48·3 (46·0 to 50·3)	52·4 (49·9 to 54·7)	51·4 (48·9 to 53·6)	53·1 (50·4 to 55·6)	53·6 (51·0 to 56·0)	191	5·4 (4·2 to 6·6)	11·3 (8·6 to 13·9)
Central African Republic	41·8 (39·3 to 44·0)	42·9 (40·6 to 45·1)	45·6 (43·1 to 47·8)	45·5 (42·7 to 47·7)	46·0 (43·3 to 48·2)	46·4 (44·0 to 49·0)	204	4·6 (3·2 to 6·0)	11·1 (7·5 to 14·7)
Chad	42·7 (40·3 to 44·4)	48·2 (46·0 to 50·3)	49·9 (47·5 to 52·1)	50·6 (47·9 to 52·9)	50·6 (47·8 to 52·9)	50·9 (48·3 to 53·2)	202	8·2 (7·1 to 9·4)	19·3 (16·3 to 22·3)
Comoros	58·3 (55·2 to 61·0)	65·2 (61·7 to 67·9)	65·0 (61·5 to 67·9)	63·2 (59·8 to 66·2)	65·4 (61·8 to 68·5)	66·0 (62·3 to 69·1)	92	7·7 (6·3 to 9·0)	13·2 (10·9 to 15·1)
Congo (Brazzaville)	47·8 (44·9 to 50·3)	53·1 (50·3 to 55·7)	53·7 (50·9 to 56·1)	52·7 (49·9 to 55·2)	53·9 (51·1 to 56·5)	54·3 (51·2 to 56·9)	188	6·4 (5·3 to 7·6)	13·5 (10·9 to 16·2)
Côte d'Ivoire	47·1 (44·1 to 49·4)	51·6 (49·0 to 53·8)	55·0 (52·2 to 57·6)	54·9 (52·0 to 57·7)	55·5 (52·8 to 58·2)	55·9 (52·9 to 58·7)	181	8·8 (7·4 to 10·3)	18·8 (15·6 to 22·0)
DR Congo	45·2 (42·5 to 47·8)	48·9 (46·2 to 51·3)	51·6 (49·0 to 54·1)	51·4 (48·5 to 54·0)	52·0 (49·2 to 54·9)	52·6 (50·0 to 55·5)	196	7·4 (6·2 to 8·7)	16·4 (13·6 to 19·4)
Djibouti	55·0 (52·3 to 57·1)	57·4 (54·7 to 59·8)	59·2 (56·2 to 61·7)	57·9 (55·0 to 60·6)	59·3 (56·2 to 62·1)	59·9 (56·8 to 62·8)	154	4·8 (3·5 to 6·3)	8·8 (6·3 to 11·4)
Equatorial Guinea	44·2 (41·7 to 46·4)	52·7 (50·4 to 55·1)	53·5 (50·7 to 55·9)	53·0 (49·7 to 55·5)	53·9 (50·9 to 56·6)	54·2 (51·3 to 57·1)	189	10·0 (8·5 to 11·6)	22·6 (19·4 to 26·4)
Eritrea	42·5 (40·1 to 44·8)	52·6 (49·7 to 55·1)	54·3 (51·4 to 56·7)	54·2 (51·3 to 56·9)	54·1 (51·1 to 56·5)	54·9 (52·1 to 57·6)	185	12·5 (11·0 to 13·9)	29·4 (25·8 to 33·1)
Eswatini	52·4 (49·8 to 54·7)	43·7 (41·4 to 46·1)	49·3 (46·9 to 51·6)	47·8 (45·1 to 50·1)	51·1 (48·2 to 53·8)	52·7 (49·6 to 55·4)	195	0·3 (-1·1 to 1·8)	0·5 (-2·1 to 3·3)
Ethiopia	41·0 (38·8 to 42·9)	53·8 (51·4 to 55·9)	57·0 (54·3 to 59·5)	56·0 (53·1 to 58·4)	56·9 (54·0 to 59·4)	57·2 (54·4 to 59·6)	173	16·2 (15·0 to 17·5)	39·5 (36·2 to 43·3)
Gabon	54·0 (50·8 to 56·4)	55·0 (52·0 to 57·6)	55·2 (52·3 to 57·9)	54·5 (51·2 to 57·6)	56·0 (52·8 to 59·2)	56·7 (53·6 to 59·6)	175	2·7 (1·7 to 3·9)	5·0 (3·1 to 7·3)
Ghana	49·6 (47·0 to 51·9)	55·9 (52·8 to 58·2)	57·4 (54·5 to 59·9)	56·7 (53·6 to 59·4)	57·5 (54·4 to 60·2)	58·0 (54·9 to 60·6)	170	8·4 (7·1 to 9·6)	16·9 (14·3 to 19·9)
Guinea	42·2 (39·8 to 44·2)	51·1 (48·5 to 53·2)	53·0 (50·4 to 55·4)	52·9 (49·8 to 55·4)	53·4 (50·5 to 55·9)	54·0 (51·2 to 56·8)	190	11·8 (10·3 to 13·3)	28·0 (24·3 to 31·7)
Guinea-Bissau	44·9 (42·4 to 46·9)	49·9 (47·3 to 52·4)	51·3 (48·4 to 53·6)	51·3 (48·3 to 53·8)	51·8 (49·0 to 54·5)	52·5 (49·8 to 54·9)	197	7·6 (6·2 to 8·9)	17·0 (13·9 to 20·7)
Kenya	54·6 (51·9 to 57·1)	56·7 (54·1 to 59·1)	59·2 (56·3 to 61·8)	57·9 (54·9 to 60·7)	60·4 (57·1 to 63·0)	61·0 (58·0 to 63·6)	141	6·5 (5·2 to 7·6)	11·8 (9·6 to 14·1)
Lesotho	53·7 (51·3 to 55·8)	47·9 (45·6 to 50·0)	49·6 (47·2 to 51·7)	48·1 (45·5 to 50·3)	50·4 (47·5 to 52·9)	51·2 (48·5 to 53·9)	201	-2·5 (-3·9 to -1·1)	-4·7 (-7·3 to -2·1)
Liberia	42·4 (39·8 to 44·7)	49·5 (46·6 to 52·1)	50·9 (47·9 to 53·3)	49·0 (46·0 to 51·5)	52·1 (48·9 to 54·7)	52·7 (49·6 to 55·3)	194	10·3 (8·8 to 11·7)	24·2 (20·4 to 28·3)
Madagascar	46·0 (43·8 to 47·9)	54·0 (51·7 to 56·1)	54·9 (52·4 to 57·3)	54·4 (51·7 to 56·6)	54·8 (52·3 to 57·1)	55·3 (52·9 to 57·6)	183	9·3 (8·0 to 10·5)	20·1 (17·1 to 22·7)
Malawi	41·0 (38·7 to 42·8)	51·2 (48·6 to 53·4)	55·0 (51·8 to 57·7)	51·4 (48·6 to 53·7)	54·6 (51·7 to 57·1)	55·0 (52·2 to 57·7)	184	14·1 (12·7 to 15·6)	34·4 (31·0 to 38·1)
Mali	41·2 (38·7 to 43·5)	52·8 (49·8 to 55·4)	53·1 (50·2 to 55·8)	52·6 (49·6 to 55·5)	53·8 (51·0 to 56·7)	54·4 (51·3 to 57·2)	187	13·2 (11·6 to 14·9)	31·9 (28·4 to 36·0)
Mauritania	52·8 (50·1 to 55·2)	57·5 (54·8 to 59·9)	57·4 (54·5 to 59·9)	56·5 (53·5 to 59·0)	57·7 (54·7 to 60·3)	58·7 (55·7 to 61·2)	159	5·9 (4·6 to 7·0)	11·2 (8·7 to 13·3)
Mozambique	46·4 (43·7 to 48·7)	53·3 (50·2 to 55·8)	55·4 (52·1 to 58·2)	54·1 (51·0 to 56·7)	55·9 (52·4 to 58·7)	56·5 (52·9 to 59·4)	178	10·1 (8·6 to 11·7)	21·8 (18·4 to 24·9)
Namibia	55·0 (52·4 to 57·1)	55·9 (53·1 to 58·2)	57·5 (54·4 to 59·9)	53·7 (51·0 to 55·8)	57·7 (54·7 to 60·1)	58·7 (55·7 to 61·1)	161	3·7 (2·8 to 4·5)	6·8 (5·1 to 8·2)
Niger	36·9 (34·4 to 38·6)	48·9 (46·5 to 51·0)	50·6 (48·0 to 52·7)	50·9 (48·2 to 53·2)	51·2 (48·7 to 53·6)	51·7 (49·1 to 54·2)	199	14·7 (13·1 to 16·1)	39·9 (35·0 to 44·9)
Nigeria	44·6 (42·0 to 46·8)	51·7 (49·0 to 54·1)	54·5 (51·5 to 57·3)	54·5 (51·4 to 57·2)	55·1 (51·9 to 57·8)	55·4 (52·2 to 58·3)	182	10·9 (9·6 to 12·2)	24·4 (21·2 to 27·9)
Rwanda	43·1 (41·0 to 44·9)	55·8 (53·1 to 58·1)	58·5 (55·8 to 60·9)	57·5 (54·7 to 59·9)	58·0 (55·1 to 60·2)	58·0 (55·1 to 60·4)	168	14·9 (13·6 to 16·4)	34·5 (31·3 to 37·7)
Senegal	50·7 (47·8 to 53·2)	58·5 (55·5 to 61·3)	59·7 (56·5 to 62·7)	58·6 (55·3 to 61·5)	60·0 (56·7 to 63·1)	60·6 (57·3 to 63·6)	148	9·8 (8·7 to 11·3)	19·4 (17·2 to 22·4)
Sierra Leone	42·0 (39·8 to 44·1)	47·1 (44·6 to 49·2)	50·2 (47·6 to 52·6)	50·6 (47·9 to 53·2)	50·9 (48·1 to 53·3)	51·2 (48·3 to 53·6)	200	9·2 (7·8 to 10·5)	21·8 (18·8 to 24·9)
Somalia	46·0 (43·4 to 48·4)	46·8 (44·3 to 48·9)	55·4 (52·4 to 58·0)	53·2 (50·1 to 55·8)	56·0 (52·9 to 58·8)	56·6 (53·6 to 59·4)	176	10·6 (9·1 to 12·0)	23·0 (19·6 to 26·3)
South Africa	61·1 (57·8 to 63·9)	51·5 (48·8 to 53·7)	56·7 (53·7 to 59·2)	54·5 (51·6 to 56·9)	57·4 (54·3 to 60·1)	58·2 (55·0 to 61·0)	164	-2·9 (-3·8 to -1·9)	-4·7 (-6·2 to -3·2)
South Sudan	45·1 (42·4 to 47·3)	49·7 (47·2 to 52·1)	49·7 (46·8 to 52·0)	49·6 (46·6 to 51·8)	49·6 (46·7 to 52·3)	49·9 (46·9 to 52·3)	203	4·8 (3·3 to 6·1)	10·7 (7·4 to 13·9)
São Tomé and Príncipe	56·1 (53·3 to 58·4)	59·2 (56·4 to 61·7)	59·4 (56·5 to 61·9)	59·0 (56·2 to 61·6)	60·6 (57·6 to 63·0)	61·6 (58·5 to 64·3)	137	5·5 (4·3 to 6·5)	9·8 (7·6 to 11·8)
Tanzania	47·8 (45·3 to 50·0)	54·1 (51·4 to 56·5)	58·7 (55·7 to 61·1)	56·9 (54·0 to 59·4)	59·8 (56·8 to 62·5)	60·4 (57·1 to 63·3)	149	12·6 (11·2 to 13·8)	26·4 (23·8 to 29·4)
The Gambia	53·6 (50·9 to 56·0)	57·2 (54·1 to 59·8)	56·3 (53·4 to 59·0)	55·3 (52·1 to 58·1)	57·1 (54·0 to 59·9)	58·0 (54·6 to 60·6)	167	4·4 (3·1 to 5·5)	8·2 (5·8 to 10·4)
Togo	49·4 (46·7 to 51·7)	52·0 (49·2 to 54·2)	55·2 (52·3 to 57·6)	55·0 (51·9 to 57·4)	55·5 (52·5 to 58·0)	55·9 (52·5 to 58·7)	179	6·5 (5·2 to 7·8)	13·2 (10·3 to 15·7)
Uganda	42·5 (40·0 to 44·4)	53·2 (50·6 to 55·4)	57·9 (55·1 to 60·4)	55·8 (53·2 to 58·1)	57·8 (54·6 to 60·3)	58·2 (55·5 to 60·9)	165	15·8 (14·6 to 17·2)	37·1 (33·9 to 40·5)
Zambia	43·0 (40·8 to 45·0)	48·8 (46·4 to 50·9)	52·4 (49·8 to 54·7)	50·4 (47·8 to 52·6)	53·0 (50·1 to 55·4)	53·5 (50·8 to 55·9)	192	10·5 (9·1 to 11·9)	24·5 (20·8 to 27·8)
Zimbabwe	58·3 (55·4 to 60·9)	49·7 (47·2 to 51·8)	55·7 (52·7 to 58·2)	52·6 (49·4 to 55·2)	56·5 (53·3 to 59·4)	57·8 (54·6 to 60·6)	172	-0·6 (-1·9 to 0·6)	-1·0 (-3·1 to 1·1)
**Low SDI**	**46·1 (43·7 to 48·1)**	**53·7 (51·0 to 56·1)**	**56·2 (53·3 to 58·7)**	**55·4 (52·5 to 57·9)**	**56·7 (53·6 to 59·1)**	**57·0 (53·9 to 59·7)**		**11·0 (10·2 to 11·7)**	**23·8 (22·3 to 25·2)**
**Low-middle SDI**	**51·2 (48·5 to 53·5)**	**58·2 (55·3 to 60·8)**	**59·5 (56·3 to 62·2)**	**58·8 (55·6 to 61·5)**	**59·9 (56·7 to 62·6)**	**60·2 (56·8 to 63·0)**		**9·1 (8·2 to 9·9)**	**17·7 (16·1 to 19·3)**

**Middle SDI**	**58·8 (56·1 to 61·1)**	**63·8 (60·8 to 66·2)**	**64·5 (61·4 to 67·1)**	**63·2 (60·2 to 65·8)**	**64·5 (61·4 to 67·1)**	**64·8 (61·6 to 67·5)**	**6·0 (5·3 to 6·7)**	**10·2 (9·2 to 11·2)**
**High-middle SDI**	**59·3 (56·6 to 61·6)**	**65·0 (61·9 to 67·6)**	**66·4 (63·0 to 69·1)**	**65·5 (62·1 to 68·3)**	**66·5 (63·0 to 69·3)**	**66·7 (63·1 to 69·7)**	**7·5 (6·6 to 8·3)**	**12·6 (11·5 to 13·6)**
**High SDI**	**64·7 (61·8 to 67·4)**	**68·4 (65·2 to 71·2)**	**69·2 (65·7 to 72·1)**	**68·6 (65·1 to 71·5)**	**69·1 (65·5 to 72·0)**	**69·3 (65·7 to 72·3)**	**4·6 (3·9 to 5·2)**	**7·1 (6·2 to 7·8)**

*United Nations convention recognises Taiwan as a province of China

Table S9: Female morbidity gap, 1990, 2010, 2020, 2021, 2022, and 2023, and absolute and relative changes, 1990–2023, globally, by SDI quintile, and for GBD super-regions and countries and territories

Location	1990	2010	2020	2021	2022	2023	Rank (2023)	Absolute Change (1990-2023)	Relative Change, % (1990-2023)
**Global**	**10·0 (7·6 to 12·8)**	**11·2 (8·6 to 14·1)**	**11·8 (9·1 to 15·2)**	**11·8 (9·0 to 15·1)**	**12·1 (9·3 to 15·4)**	**12·1 (9·2 to 15·6)**		**2·1 (1·3 to 2·9)**	**21·3 (14·7 to 28·6)**
**Central Europe, Eastern Europe, and Central Asia**	**10·6 (8·1 to 13·6)**	**10·9 (8·3 to 13·9)**	**11·5 (8·8 to 14·9)**	**11·3 (8·6 to 14·6)**	**11·9 (9·1 to 15·5)**	**12·0 (9·1 to 15·6)**		**1·4 (0·8 to 2·1)**	**13·2 (8·5 to 18·0)**
Albania	10·5 (7·9 to 13·8)	11·3 (8·4 to 14·7)	11·7 (8·9 to 15·3)	11·9 (9·2 to 15·4)	12·3 (9·4 to 15·9)	12·2 (9·2 to 15·7)	89	1·7 (0·4 to 3·0)	16·4 (3·7 to 30·7)
Armenia	10·3 (7·8 to 13·2)	10·8 (8·3 to 13·8)	11·6 (8·8 to 14·8)	12·0 (9·2 to 15·3)	12·6 (9·4 to 16·0)	12·8 (9·6 to 16·6)	54	2·5 (1·2 to 3·9)	24·5 (12·3 to 36·8)
Azerbaijan	10·0 (7·4 to 13·1)	10·4 (7·8 to 13·4)	10·5 (8·0 to 13·5)	10·6 (8·1 to 13·5)	11·3 (8·5 to 14·4)	11·2 (8·2 to 14·4)	125	1·1 (0·0 to 2·4)	11·6 (-0·3 to 24·8)
Belarus	10·6 (8·0 to 13·7)	10·9 (8·3 to 13·9)	11·6 (8·9 to 15·0)	11·8 (8·9 to 15·1)	11·7 (8·8 to 15·1)	11·8 (8·9 to 15·3)	103	1·2 (0·3 to 2·1)	10·9 (2·6 to 20·0)
Bosnia and Herzegovina	10·3 (7·9 to 13·3)	11·2 (8·6 to 14·4)	11·7 (9·0 to 15·2)	11·7 (8·8 to 15·0)	12·1 (9·2 to 15·7)	12·2 (9·1 to 15·8)	90	1·8 (0·6 to 3·0)	17·7 (5·9 to 29·0)
Bulgaria	10·1 (7·7 to 13·1)	10·7 (8·2 to 13·8)	11·2 (8·5 to 14·6)	10·9 (8·3 to 14·2)	11·4 (8·6 to 14·9)	11·6 (8·7 to 15·1)	113	1·5 (0·7 to 2·2)	14·5 (7·6 to 21·5)
Croatia	10·5 (8·0 to 13·4)	11·3 (8·6 to 14·7)	12·1 (9·2 to 15·5)	12·2 (9·3 to 15·8)	12·2 (9·3 to 15·9)	12·5 (9·6 to 16·1)	72	2·0 (1·3 to 2·9)	19·2 (13·3 to 25·9)
Czechia	10·9 (8·3 to 14·0)	12·2 (9·3 to 15·6)	12·7 (9·7 to 16·3)	12·8 (9·7 to 16·3)	13·0 (9·9 to 16·7)	13·1 (10·1 to 16·8)	44	2·2 (1·5 to 3·1)	20·4 (14·8 to 25·5)
Estonia	10·6 (8·1 to 13·6)	11·8 (8·9 to 15·2)	12·7 (9·6 to 16·3)	12·5 (9·5 to 16·1)	12·8 (9·8 to 16·4)	13·0 (9·8 to 16·7)	50	2·4 (1·6 to 3·3)	22·9 (15·9 to 29·2)
Georgia	10·0 (7·5 to 13·2)	10·8 (8·1 to 14·0)	11·7 (8·8 to 15·1)	11·4 (8·6 to 14·7)	12·0 (8·9 to 15·5)	12·1 (9·1 to 15·6)	93	2·1 (0·9 to 3·4)	20·8 (9·3 to 34·1)
Hungary	10·9 (8·3 to 14·0)	11·4 (8·6 to 14·8)	11·7 (9·0 to 15·2)	11·6 (8·9 to 15·1)	12·0 (9·1 to 15·5)	12·1 (9·3 to 15·7)	91	1·3 (0·7 to 2·0)	11·6 (6·3 to 17·2)
Kazakhstan	10·4 (8·0 to 13·3)	10·4 (7·8 to 13·3)	10·9 (8·3 to 14·0)	10·7 (8·1 to 13·8)	11·7 (8·8 to 15·1)	11·7 (9·0 to 15·2)	105	1·3 (0·4 to 2·5)	12·6 (3·8 to 23·3)
Kyrgyzstan	10·2 (7·6 to 13·2)	10·3 (7·8 to 13·3)	10·8 (8·3 to 13·8)	11·1 (8·5 to 14·3)	11·5 (8·7 to 15·0)	11·5 (8·6 to 14·8)	118	1·3 (0·1 to 2·4)	12·7 (0·9 to 24·6)
Latvia	10·8 (8·2 to 13·8)	11·3 (8·5 to 14·6)	12·0 (9·0 to 15·5)	11·7 (8·9 to 15·2)	12·1 (9·1 to 15·6)	12·4 (9·3 to 16·0)	79	1·6 (0·8 to 2·5)	14·6 (7·6 to 21·7)
Lithuania	11·0 (8·3 to 14·2)	11·6 (8·8 to 14·9)	12·3 (9·3 to 15·9)	12·1 (9·2 to 15·8)	12·4 (9·4 to 16·0)	12·7 (9·7 to 16·3)	60	1·7 (1·0 to 2·5)	15·4 (9·7 to 21·8)
Moldova	10·1 (7·6 to 13·1)	10·5 (7·8 to 13·8)	11·5 (8·8 to 14·7)	11·4 (8·7 to 14·6)	12·3 (9·2 to 15·7)	12·3 (9·1 to 16·0)	81	2·3 (1·0 to 3·5)	22·6 (10·8 to 34·8)
Mongolia	8·8 (6·5 to 11·5)	9·5 (7·0 to 12·2)	10·6 (8·1 to 13·8)	10·3 (7·8 to 13·4)	11·1 (8·1 to 14·6)	11·3 (8·2 to 14·8)	121	2·5 (0·9 to 4·1)	28·7 (11·3 to 48·3)
Montenegro	10·1 (7·7 to 13·0)	10·7 (8·1 to 13·7)	11·2 (8·5 to 14·6)	11·1 (8·5 to 14·2)	11·9 (9·0 to 15·3)	11·8 (8·9 to 15·3)	100	1·7 (0·8 to 2·6)	16·7 (9·3 to 24·4)
North Macedonia	9·9 (7·5 to 12·7)	10·4 (7·9 to 13·5)	11·1 (8·5 to 14·2)	11·1 (8·5 to 14·2)	11·6 (8·9 to 14·9)	11·6 (8·7 to 14·8)	114	1·7 (0·7 to 2·7)	17·1 (7·7 to 26·4)
Poland	11·1 (8·5 to 14·3)	11·8 (9·0 to 15·2)	12·1 (9·3 to 15·8)	12·0 (9·2 to 15·7)	12·5 (9·5 to 16·2)	12·7 (9·7 to 16·5)	59	1·6 (1·0 to 2·4)	14·6 (9·4 to 19·4)
Romania	10·0 (7·6 to 12·9)	10·6 (8·0 to 13·6)	11·0 (8·4 to 14·4)	10·8 (8·3 to 14·0)	11·4 (8·7 to 15·0)	11·5 (8·8 to 15·0)	115	1·5 (0·8 to 2·2)	14·9 (8·2 to 21·1)
Russia	10·9 (8·2 to 14·0)	11·0 (8·3 to 14·1)	11·7 (8·9 to 15·2)	11·5 (8·7 to 15·0)	12·3 (9·4 to 16·0)	12·3 (9·3 to 16·0)	82	1·5 (0·9 to 2·2)	13·5 (8·7 to 19·0)
Serbia	9·3 (7·0 to 12·0)	10·1 (7·7 to 13·1)	10·6 (8·1 to 13·5)	10·4 (8·0 to 13·4)	10·8 (8·2 to 13·8)	10·9 (8·1 to 14·0)	133	1·6 (0·4 to 2·8)	17·5 (4·2 to 31·3)
Slovakia	10·5 (8·0 to 13·5)	11·4 (8·7 to 14·8)	11·8 (8·9 to 15·3)	11·4 (8·6 to 14·8)	12·0 (8·9 to 15·5)	12·2 (9·3 to 15·8)	88	1·7 (1·0 to 2·5)	16·3 (10·0 to 23·3)
Slovenia	11·0 (8·3 to 14·0)	12·4 (9·4 to 15·9)	12·9 (9·9 to 16·5)	13·2 (10·0 to 16·9)	13·4 (10·2 to 17·1)	13·6 (10·3 to 17·2)	29	2·6 (1·8 to 3·6)	24·0 (18·2 to 30·3)
Tajikistan	9·6 (7·2 to 12·5)	10·1 (7·5 to 13·2)	10·3 (7·8 to 13·3)	10·2 (7·5 to 13·2)	10·2 (7·3 to 13·4)	10·1 (7·2 to 13·5)	164	0·5 (-1·1 to 2·2)	5·8 (-11·4 to 25·8)
Turkmenistan	9·5 (7·1 to 12·3)	9·9 (7·4 to 12·6)	10·1 (7·4 to 13·1)	10·1 (7·3 to 13·3)	10·5 (7·4 to 13·8)	10·5 (7·3 to 13·8)	155	1·0 (-0·8 to 2·7)	10·6 (-8·1 to 30·0)
Ukraine	11·1 (8·5 to 14·1)	10·9 (8·2 to 13·8)	11·7 (8·9 to 14·9)	11·4 (8·6 to 14·6)	11·8 (8·5 to 15·6)	12·3 (8·9 to 16·1)	83	1·2 (-0·1 to 2·6)	11·3 (-1·0 to 23·6)
Uzbekistan	10·0 (7·4 to 12·9)	9·7 (7·2 to 12·4)	10·3 (7·9 to 13·4)	10·7 (8·0 to 13·7)	10·6 (8·0 to 13·7)	10·5 (7·8 to 13·6)	154	0·5 (-0·5 to 1·5)	4·6 (-4·7 to 15·1)
**High income**	**12·1 (9·2 to 15·3)**	**13·2 (10·1 to 16·7)**	**13·8 (10·6 to 17·5)**	**13·9 (10·7 to 17·7)**	**14·0 (10·7 to 17·8)**	**14·2 (10·9 to 18·1)**		**2·2 (1·6 to 2·9)**	**18·3 (14·9 to 21·6)**
Andorra	12·3 (9·3 to 15·9)	13·3 (10·1 to 17·0)	13·8 (10·5 to 17·5)	14·2 (10·8 to 18·1)	14·0 (10·7 to 17·8)	14·1 (10·7 to 17·8)	13	1·8 (0·7 to 2·8)	15·0 (6·6 to 23·8)
Argentina	10·8 (8·1 to 13·7)	11·4 (8·7 to 14·5)	11·9 (9·0 to 15·2)	11·7 (8·9 to 14·9)	12·2 (9·2 to 15·6)	12·5 (9·5 to 15·9)	74	1·7 (0·9 to 2·6)	15·8 (9·3 to 22·7)
Australia	12·3 (9·3 to 15·8)	14·3 (10·9 to 18·1)	15·2 (11·6 to 19·2)	15·2 (11·6 to 19·2)	15·1 (11·5 to 19·2)	15·4 (11·8 to 19·5)	2	3·1 (2·3 to 4·0)	25·2 (21·1 to 30·3)
Austria	11·1 (8·5 to 14·3)	12·4 (9·4 to 15·9)	13·0 (9·9 to 16·9)	13·1 (10·0 to 16·9)	13·1 (10·0 to 16·9)	13·3 (10·1 to 17·1)	37	2·2 (1·5 to 3·0)	19·8 (14·4 to 24·5)
Belgium	11·4 (8·7 to 14·6)	12·9 (9·9 to 16·4)	13·4 (10·2 to 17·1)	13·8 (10·6 to 17·7)	13·9 (10·6 to 17·8)	13·9 (10·6 to 17·8)	19	2·5 (1·7 to 3·3)	21·6 (16·4 to 26·7)
Brunei	9·5 (7·1 to 12·3)	10·3 (7·7 to 13·3)	10·7 (8·1 to 13·8)	10·7 (8·0 to 13·9)	10·7 (8·0 to 14·0)	10·9 (8·1 to 14·3)	134	1·4 (0·4 to 2·5)	14·9 (4·8 to 26·2)
Canada	13·1 (9·9 to 16·7)	14·4 (11·1 to 18·2)	15·0 (11·5 to 18·9)	15·2 (11·7 to 19·0)	15·1 (11·6 to 18·9)	15·3 (11·7 to 19·3)	3	2·3 (1·4 to 3·0)	17·4 (11·8 to 22·4)
Chile	11·4 (8·6 to 14·5)	12·3 (9·2 to 15·6)	13·0 (9·8 to 16·5)	13·0 (9·8 to 16·4)	13·3 (10·0 to 16·9)	13·7 (10·3 to 17·5)	27	2·3 (1·5 to 3·2)	20·3 (14·7 to 26·4)
Cyprus	10·5 (7·9 to 13·8)	11·5 (8·6 to 14·9)	12·2 (9·2 to 15·9)	12·2 (9·2 to 15·7)	12·4 (9·2 to 16·0)	12·7 (9·5 to 16·4)	61	2·2 (0·7 to 3·7)	21·0 (6·8 to 37·2)
Denmark	11·0 (8·4 to 14·2)	11·7 (8·9 to 15·1)	12·7 (9·7 to 16·2)	12·7 (9·7 to 16·3)	12·8 (9·8 to 16·3)	13·0 (9·9 to 16·5)	51	1·9 (1·3 to 2·7)	17·7 (12·6 to 22·7)
Finland	11·1 (8·4 to 14·2)	12·6 (9·6 to 16·0)	13·0 (9·9 to 16·6)	13·0 (10·0 to 16·7)	12·9 (9·8 to 16·5)	13·1 (10·0 to 16·9)	45	2·0 (1·4 to 2·8)	18·6 (13·5 to 23·7)
France	11·9 (9·0 to 15·0)	13·1 (9·8 to 16·6)	13·7 (10·5 to 17·5)	14·0 (10·7 to 18·0)	14·0 (10·6 to 18·0)	14·1 (10·6 to 18·1)	14	2·3 (1·5 to 3·1)	19·0 (14·3 to 23·4)
Germany	11·5 (8·7 to 14·8)	13·0 (9·9 to 16·9)	13·4 (10·2 to 17·2)	13·4 (10·2 to 17·1)	13·3 (10·2 to 16·8)	13·5 (10·3 to 17·2)	35	2·0 (1·2 to 2·8)	17·0 (11·7 to 22·4)
Greece	11·2 (8·5 to 14·3)	12·5 (9·5 to 15·8)	13·2 (10·1 to 16·9)	13·1 (10·0 to 16·8)	13·2 (10·1 to 17·2)	13·5 (10·3 to 17·3)	34	2·3 (1·5 to 3·1)	20·2 (14·9 to 25·8)
Greenland	9·9 (7·4 to 12·8)	10·8 (8·2 to 13·8)	11·4 (8·5 to 14·6)	11·5 (8·6 to 14·7)	11·8 (8·9 to 15·3)	12·1 (9·1 to 15·6)	94	2·2 (0·9 to 3·3)	22·1 (10·0 to 34·2)
Iceland	11·6 (8·8 to 14·8)	12·8 (9·8 to 16·3)	13·6 (10·3 to 17·3)	13·7 (10·3 to 17·4)	13·6 (10·3 to 17·3)	13·8 (10·5 to 17·5)	23	2·2 (1·4 to 3·0)	19·0 (13·1 to 26·0)
Ireland	10·6 (8·1 to 13·6)	12·2 (9·3 to 15·7)	13·2 (10·0 to 17·0)	13·4 (10·2 to 17·3)	13·4 (10·2 to 17·3)	13·6 (10·3 to 17·6)	28	2·9 (2·0 to 3·9)	27·8 (21·5 to 34·1)
Israel	10·9 (8·3 to 14·0)	12·3 (9·3 to 15·9)	12·9 (9·8 to 16·5)	12·9 (9·8 to 16·6)	13·0 (9·8 to 16·6)	13·2 (9·9 to 17·1)	42	2·3 (1·5 to 3·1)	21·0 (15·0 to 27·1)
Italy	11·4 (8·6 to 14·7)	12·1 (9·2 to 15·5)	12·6 (9·6 to 16·3)	12·8 (9·7 to 16·5)	12·8 (9·8 to 16·5)	13·2 (10·0 to 17·0)	40	1·8 (1·2 to 2·5)	15·8 (11·7 to 19·7)
Japan	11·5 (8·8 to 14·8)	12·8 (9·7 to 16·6)	13·1 (10·0 to 16·8)	13·2 (10·0 to 16·9)	13·2 (10·0 to 16·9)	13·3 (10·1 to 17·0)	36	1·7 (1·2 to 2·3)	15·0 (11·2 to 18·7)

Luxembourg	11·0 (8·3 to 14·1)	12·4 (9·4 to 15·8)	13·2 (10·1 to 17·0)	13·4 (10·2 to 17·1)	13·5 (10·2 to 17·4)	13·5 (10·1 to 17·4)	32	2·5 (1·6 to 3·5)	23·0 (16·2 to 30·6)
Malta	10·6 (8·1 to 13·8)	11·9 (9·0 to 15·2)	12·9 (9·8 to 16·4)	12·9 (9·8 to 16·5)	13·0 (9·7 to 16·6)	13·2 (9·9 to 16·9)	38	2·7 (1·7 to 3·8)	25·2 (17·7 to 33·8)
Monaco	10·9 (8·0 to 14·2)	11·6 (8·6 to 15·0)	12·5 (9·3 to 16·0)	12·7 (9·4 to 16·4)	12·8 (9·5 to 16·3)	12·9 (9·5 to 16·9)	52	2·0 (0·5 to 3·6)	18·3 (4·2 to 34·8)
Netherlands	11·8 (9·0 to 15·1)	14·6 (11·2 to 18·4)	14·0 (10·6 to 17·7)	14·2 (10·9 to 18·0)	14·3 (10·9 to 18·3)	14·6 (11·2 to 18·6)	5	2·9 (2·1 to 3·7)	24·3 (19·7 to 29·1)
New Zealand	12·9 (9·7 to 16·4)	13·9 (10·6 to 17·7)	14·6 (11·2 to 18·6)	14·6 (11·1 to 18·5)	14·4 (10·9 to 18·2)	14·7 (11·2 to 18·6)	4	1·8 (1·1 to 2·5)	13·9 (9·1 to 18·9)
Norway	11·7 (8·9 to 15·1)	12·7 (9·7 to 16·3)	13·6 (10·4 to 17·3)	13·6 (10·5 to 17·5)	13·6 (10·5 to 17·4)	13·9 (10·6 to 17·7)	21	2·1 (1·5 to 3·0)	18·2 (13·7 to 23·0)
Portugal	11·0 (8·4 to 14·3)	12·5 (9·5 to 16·0)	13·2 (10·1 to 17·1)	13·3 (10·2 to 17·2)	13·4 (10·2 to 17·3)	13·7 (10·4 to 17·6)	25	2·7 (1·8 to 3·7)	24·3 (18·0 to 30·7)
San Marino	11·8 (8·8 to 15·1)	13·2 (9·9 to 17·0)	13·7 (10·3 to 17·6)	14·2 (10·7 to 18·1)	14·0 (10·4 to 18·0)	14·3 (10·9 to 18·6)	7	2·6 (0·9 to 4·4)	21·9 (8·4 to 37·1)
Singapore	9·8 (7·4 to 12·8)	11·1 (8·5 to 14·3)	12·1 (9·1 to 15·6)	12·0 (9·1 to 15·3)	11·8 (8·9 to 15·2)	12·1 (9·1 to 15·7)	92	2·3 (1·6 to 3·1)	24·2 (17·2 to 31·4)
South Korea	10·5 (8·0 to 13·8)	11·9 (9·1 to 15·2)	12·6 (9·6 to 16·2)	12·6 (9·5 to 16·1)	12·3 (9·4 to 15·9)	12·6 (9·5 to 16·3)	64	2·1 (1·3 to 2·9)	20·1 (13·2 to 27·2)
Spain	11·7 (8·9 to 14·9)	12·5 (9·5 to 16·0)	13·1 (10·0 to 16·7)	13·5 (10·3 to 17·2)	13·5 (10·2 to 17·1)	13·7 (10·4 to 17·4)	26	2·0 (1·3 to 2·8)	17·0 (12·1 to 22·4)
Sweden	10·8 (8·1 to 13·8)	11·9 (9·1 to 15·1)	13·1 (10·0 to 16·6)	13·3 (10·2 to 17·0)	13·5 (10·3 to 17·1)	13·7 (10·5 to 17·3)	24	2·9 (2·1 to 3·8)	27·0 (22·3 to 32·1)
Switzerland	11·9 (9·0 to 15·2)	13·1 (10·0 to 16·7)	13·8 (10·5 to 17·6)	14·0 (10·7 to 18·0)	14·2 (10·7 to 18·2)	14·3 (10·9 to 18·5)	8	2·5 (1·7 to 3·4)	20·9 (15·7 to 26·0)
UK	11·3 (8·6 to 14·4)	12·9 (9·9 to 16·7)	13·8 (10·6 to 17·5)	14·1 (10·8 to 17·9)	14·3 (10·9 to 18·2)	14·3 (11·0 to 18·2)	9	3·0 (2·2 to 3·9)	26·7 (22·9 to 30·7)
USA	13·6 (10·5 to 17·1)	14·3 (11·0 to 18·0)	14·9 (11·5 to 18·6)	14·9 (11·5 to 18·7)	15·2 (11·8 to 19·1)	15·6 (12·0 to 19·6)	1	2·0 (1·4 to 2·6)	14·5 (11·2 to 18·0)
Uruguay	11·0 (8·3 to 14·1)	11·7 (9·0 to 15·0)	12·4 (9·5 to 15·9)	11·9 (9·1 to 15·3)	12·3 (9·3 to 15·7)	12·6 (9·5 to 16·1)	69	1·6 (0·8 to 2·4)	14·7 (8·0 to 21·6)
**Latin America and Caribbean**	**11·8 (9·0 to 15·1)**	**12·2 (9·3 to 15·6)**	**12·8 (9·8 to 16·3)**	**12·6 (9·6 to 15·9)**	**13·4 (10·3 to 17·1)**	**13·6 (10·4 to 17·4)**		**1·8 (1·2 to 2·5)**	**15·4 (10·6 to 20·5)**
Antigua and Barbuda	11·2 (8·4 to 14·6)	12·2 (9·2 to 15·8)	12·4 (9·5 to 16·1)	12·3 (9·3 to 15·9)	12·5 (9·4 to 16·3)	12·7 (9·6 to 16·6)	62	1·5 (0·4 to 2·7)	14·0 (3·3 to 25·2)
Bahamas	11·0 (8·3 to 14·2)	11·1 (8·4 to 14·6)	11·1 (8·4 to 14·6)	10·5 (7·9 to 13·8)	11·3 (8·5 to 14·9)	11·7 (8·7 to 15·4)	108	0·7 (-0·4 to 1·9)	6·6 (-3·4 to 17·2)
Barbados	11·1 (8·2 to 14·3)	12·1 (9·1 to 15·7)	12·1 (9·2 to 15·6)	11·9 (9·1 to 15·4)	12·0 (9·0 to 15·6)	12·4 (9·3 to 16·2)	77	1·3 (0·2 to 2·4)	11·8 (2·3 to 22·1)
Belize	11·2 (8·4 to 14·5)	11·6 (8·9 to 15·1)	12·0 (9·1 to 15·5)	11·9 (9·0 to 15·2)	12·4 (9·3 to 16·1)	12·6 (9·4 to 16·3)	68	1·4 (0·2 to 2·6)	12·6 (2·2 to 24·1)
Bermuda	11·2 (8·4 to 14·5)	12·5 (9·5 to 16·0)	12·8 (9·8 to 16·5)	12·7 (9·6 to 16·3)	12·8 (9·5 to 16·5)	13·0 (9·8 to 16·7)	47	1·9 (0·9 to 2·8)	17·0 (8·5 to 25·7)
Bolivia	9·8 (7·3 to 12·7)	10·6 (8·1 to 13·5)	10·0 (7·5 to 12·9)	10·5 (7·9 to 13·5)	11·6 (8·8 to 14·9)	11·7 (8·8 to 15·2)	106	1·9 (0·7 to 3·2)	19·7 (7·5 to 33·1)
Brazil	12·0 (9·2 to 15·4)	12·9 (9·8 to 16·4)	13·5 (10·4 to 17·2)	13·2 (10·1 to 16·7)	14·0 (10·7 to 17·8)	14·4 (11·0 to 18·3)	6	2·4 (1·6 to 3·3)	19·9 (14·1 to 26·1)
Colombia	11·9 (9·1 to 15·3)	12·9 (9·8 to 16·3)	13·1 (10·1 to 16·8)	12·7 (9·8 to 16·4)	13·6 (10·4 to 17·5)	13·9 (10·7 to 17·6)	20	2·0 (1·2 to 2·9)	16·8 (10·0 to 24·1)
Costa Rica	12·8 (9·8 to 16·4)	13·7 (10·5 to 17·3)	14·2 (11·0 to 17·8)	14·0 (10·8 to 17·6)	14·3 (10·9 to 17·9)	14·3 (10·9 to 18·1)	10	1·5 (0·8 to 2·3)	11·7 (6·3 to 17·6)
Cuba	11·4 (8·6 to 14·8)	12·2 (9·2 to 15·6)	12·4 (9·5 to 16·1)	11·3 (8·6 to 14·6)	12·4 (9·1 to 16·4)	12·8 (9·3 to 16·8)	57	1·3 (-0·2 to 2·9)	12·0 (-1·6 to 25·5)
Dominica	11·6 (8·7 to 14·9)	12·0 (9·1 to 15·6)	12·3 (9·3 to 16·0)	12·1 (9·2 to 15·7)	12·4 (9·4 to 16·1)	12·6 (9·5 to 16·5)	65	1·1 (0·0 to 2·2)	9·2 (0·2 to 18·8)
Dominican Republic	11·1 (8·4 to 14·4)	12·2 (9·1 to 15·9)	11·9 (9·1 to 15·5)	12·0 (9·2 to 15·6)	12·0 (9·3 to 15·6)	12·1 (9·2 to 15·7)	95	1·0 (-0·1 to 2·2)	9·3 (-1·0 to 20·7)
Ecuador	10·7 (8·2 to 13·8)	11·4 (8·5 to 14·5)	11·6 (8·8 to 14·8)	12·0 (9·1 to 15·3)	12·5 (9·5 to 16·0)	12·5 (9·5 to 15·9)	71	1·8 (1·0 to 2·8)	17·1 (10·2 to 24·9)
El Salvador	11·2 (8·4 to 14·4)	11·8 (9·0 to 15·2)	11·5 (8·8 to 14·7)	11·4 (8·5 to 14·5)	11·8 (9·0 to 15·0)	11·8 (8·8 to 15·1)	102	0·6 (-0·5 to 1·6)	5·4 (-4·1 to 14·6)
Grenada	10·8 (7·9 to 13·9)	11·9 (8·9 to 15·2)	12·2 (9·1 to 15·7)	11·8 (8·5 to 15·1)	12·1 (8·8 to 15·7)	12·3 (9·1 to 15·7)	87	1·5 (-0·1 to 3·2)	14·5 (-1·4 to 31·7)
Guatemala	10·3 (7·8 to 13·4)	12·0 (9·1 to 15·5)	12·3 (9·5 to 15·6)	11·8 (9·2 to 15·1)	12·4 (9·5 to 16·1)	12·8 (9·4 to 16·5)	55	2·4 (0·8 to 4·1)	23·7 (8·7 to 40·4)
Guyana	10·6 (7·5 to 13·7)	11·1 (8·2 to 14·3)	11·5 (8·4 to 15·0)	11·1 (8·2 to 14·3)	11·4 (8·3 to 14·8)	11·6 (8·4 to 15·0)	110	1·0 (-1·0 to 3·0)	10·0 (-9·3 to 30·1)
Haiti	8·0 (5·6 to 10·5)	3·6 (1·0 to 6·1)	10·0 (7·3 to 12·9)	10·0 (7·3 to 13·0)	10·0 (7·3 to 12·9)	9·9 (7·0 to 12·9)	176	1·9 (-0·1 to 3·9)	24·8 (-0·6 to 56·3)
Honduras	11·3 (8·7 to 14·4)	11·8 (8·9 to 14·9)	11·8 (9·0 to 14·8)	11·6 (8·8 to 14·9)	12·7 (9·6 to 16·1)	12·6 (9·5 to 16·1)	66	1·3 (0·3 to 2·3)	11·4 (3·1 to 20·0)
Jamaica	11·4 (8·6 to 14·8)	12·1 (9·1 to 15·8)	12·1 (9·0 to 15·9)	11·8 (8·6 to 15·5)	12·3 (8·8 to 16·1)	12·6 (9·1 to 16·4)	70	1·2 (-0·6 to 2·9)	10·6 (-5·0 to 27·1)
Mexico	12·6 (9·6 to 16·1)	13·1 (10·0 to 16·7)	12·8 (9·8 to 16·3)	12·9 (9·9 to 16·4)	14·0 (10·7 to 17·8)	14·2 (10·9 to 18·2)	12	1·6 (1·0 to 2·2)	12·4 (8·6 to 16·9)
Nicaragua	11·7 (8·8 to 15·2)	11·9 (9·1 to 15·3)	11·7 (8·9 to 14·9)	11·7 (9·0 to 14·9)	12·7 (9·5 to 16·3)	13·1 (9·5 to 17·2)	43	1·5 (-0·1 to 3·2)	13·0 (-1·1 to 28·5)
Panama	12·0 (9·1 to 15·4)	12·8 (9·8 to 16·1)	13·0 (10·0 to 16·5)	13·2 (10·1 to 16·7)	13·5 (10·3 to 17·1)	13·6 (10·4 to 17·2)	30	1·6 (0·8 to 2·4)	13·0 (6·8 to 20·2)
Paraguay	11·8 (9·0 to 15·2)	11·7 (9·0 to 15·1)	12·0 (9·1 to 15·3)	10·8 (8·2 to 13·8)	12·1 (9·0 to 15·6)	12·3 (9·2 to 15·7)	85	0·5 (-0·5 to 1·6)	4·3 (-4·8 to 14·3)
Peru	11·8 (8·9 to 15·3)	12·4 (9·4 to 15·7)	11·6 (8·8 to 14·8)	11·3 (8·6 to 14·3)	12·8 (9·7 to 16·2)	12·4 (9·5 to 15·9)	75	0·6 (-0·2 to 1·6)	5·5 (-2·3 to 14·3)
Puerto Rico	12·3 (9·3 to 15·8)	13·0 (9·9 to 16·8)	13·8 (10·5 to 17·7)	13·9 (10·7 to 17·9)	13·9 (10·7 to 17·9)	14·1 (10·7 to 18·0)	16	1·8 (0·9 to 2·7)	14·4 (7·5 to 22·5)
Saint Kitts and Nevis	10·6 (7·9 to 13·9)	11·4 (8·5 to 14·8)	11·6 (8·7 to 15·2)	11·5 (8·6 to 15·0)	11·8 (8·8 to 15·7)	12·0 (8·9 to 15·7)	97	1·4 (0·3 to 2·7)	13·8 (2·4 to 26·6)
Saint Lucia	11·4 (8·5 to 14·8)	12·6 (9·3 to 16·4)	12·9 (9·7 to 16·8)	12·4 (9·4 to 16·2)	13·0 (9·4 to 17·2)	13·2 (9·5 to 17·3)	41	1·8 (0·1 to 3·6)	16·3 (0·6 to 33·7)
Saint Vincent and the Grenadines	11·3 (8·3 to 14·7)	11·7 (8·7 to 15·3)	12·1 (9·0 to 16·0)	12·2 (9·0 to 15·9)	12·4 (9·0 to 16·6)	12·4 (8·9 to 16·4)	76	1·1 (-0·8 to 3·0)	10·4 (-7·2 to 28·9)
Suriname	11·3 (8·6 to 14·6)	11·8 (8·9 to 15·2)	12·0 (9·1 to 15·7)	11·2 (8·5 to 14·7)	12·2 (9·3 to 15·9)	12·5 (9·4 to 16·3)	73	1·2 (0·2 to 2·2)	10·7 (1·2 to 20·0)
Trinidad and Tobago	10·9 (8·2 to 14·2)	12·1 (9·3 to 16·0)	12·2 (9·3 to 16·0)	11·6 (8·7 to 15·1)	12·1 (9·0 to 15·5)	12·4 (9·3 to 15·8)	78	1·5 (0·4 to 2·7)	13·6 (4·2 to 24·1)
Venezuela	11·5 (8·8 to 14·8)	12·4 (9·4 to 15·7)	11·9 (8·9 to 15·4)	11·8 (8·9 to 15·2)	11·9 (9·0 to 15·5)	12·0 (9·0 to 15·5)	99	0·5 (-0·5 to 1·5)	4·2 (-4·0 to 13·0)
Virgin Islands	10·5 (7·7 to 13·7)	11·9 (8·9 to 15·5)	12·9 (9·4 to 16·6)	12·8 (9·6 to 16·6)	12·9 (9·3 to 16·7)	12·7 (9·4 to 16·6)	63	2·2 (0·6 to 3·9)	21·4 (5·6 to 39·0)
**North Africa and Middle East**	**10·6 (8·1 to 13·6)**	**11·8 (9·0 to 15·1)**	**12·1 (9·3 to 15·6)**	**12·2 (9·3 to 15·6)**	**12·6 (9·7 to 16·2)**	**12·5 (9·6 to 16·2)**		**1·9 (1·1 to 2·8)**	**18·4 (10·7 to 26·7)**
Afghanistan	11·8 (8·5 to 15·9)	12·5 (9·2 to 16·6)	12·7 (9·3 to 16·7)	12·5 (9·2 to 16·3)	13·3 (9·8 to 17·3)	13·1 (9·5 to 17·1)	46	1·3 (-0·9 to 3·5)	11·9 (-6·7 to 35·8)
Algeria	10·6 (8·0 to 13·8)	11·6 (8·9 to 15·1)	11·9 (9·1 to 15·5)	12·1 (9·1 to 15·6)	12·3 (9·3 to 15·9)	12·3 (9·2 to 16·1)	84	1·7 (0·7 to 2·7)	16·4 (6·8 to 26·8)
Bahrain	10·9 (8·1 to 14·3)	11·8 (8·7 to 15·4)	12·7 (9·4 to 16·4)	12·7 (9·4 to 16·6)	13·2 (9·6 to 17·3)	13·2 (9·8 to 17·4)	39	2·4 (0·7 to 4·1)	22·0 (6·3 to 39·2)

Egypt	10·0 (7·5 to 12·8)	10·5 (8·0 to 13·4)	11·1 (8·4 to 14·3)	11·0 (8·4 to 14·1)	11·2 (8·2 to 14·7)	11·2 (8·1 to 14·7)	124	1·2 (-0·3 to 2·7)	12·2 (-3·4 to 29·3)
Iran	11·0 (8·2 to 14·2)	12·9 (9·9 to 16·6)	12·7 (9·6 to 16·3)	13·0 (9·8 to 16·6)	13·9 (10·5 to 17·8)	14·1 (10·7 to 18·1)	15	3·1 (1·9 to 4·4)	28·3 (18·4 to 39·5)
Iraq	11·2 (8·5 to 14·5)	11·3 (8·5 to 14·6)	11·8 (8·9 to 15·2)	12·3 (9·3 to 15·8)	12·5 (9·4 to 16·1)	12·3 (9·1 to 15·9)	86	1·1 (0·0 to 2·3)	9·8 (-0·1 to 20·4)
Jordan	11·6 (8·7 to 15·1)	12·1 (9·2 to 15·5)	12·5 (9·5 to 16·0)	12·2 (9·2 to 15·5)	13·4 (10·2 to 17·0)	13·5 (10·2 to 17·3)	33	1·9 (0·9 to 3·0)	16·7 (8·0 to 27·6)
Kuwait	11·2 (8·4 to 14·5)	12·6 (9·6 to 16·0)	13·2 (10·0 to 16·9)	13·3 (10·1 to 17·0)	13·9 (10·5 to 17·8)	13·9 (10·3 to 17·9)	18	2·7 (1·5 to 3·9)	24·1 (14·1 to 33·2)
Lebanon	13·3 (10·0 to 17·3)	13·8 (10·4 to 17·7)	12·6 (9·4 to 16·3)	14·1 (10·8 to 17·9)	14·1 (10·5 to 18·3)	14·3 (10·5 to 18·4)	11	1·0 (-1·1 to 2·8)	7·5 (-7·5 to 22·8)
Libya	11·4 (8·6 to 14·9)	11·7 (8·9 to 15·3)	12·1 (9·1 to 15·8)	11·6 (8·7 to 15·0)	12·8 (9·5 to 16·5)	11·5 (8·4 to 15·1)	116	0·1 (-1·2 to 1·5)	1·2 (-10·2 to 13·6)
Morocco	9·6 (7·1 to 12·6)	10·6 (7·9 to 13·7)	10·9 (8·3 to 14·3)	11·2 (8·4 to 14·7)	11·3 (8·4 to 14·8)	11·2 (8·1 to 14·7)	126	1·5 (-0·1 to 3·2)	16·0 (-0·7 to 35·3)
Oman	12·1 (8·9 to 15·6)	12·4 (9·3 to 16·0)	12·2 (9·1 to 15·5)	12·0 (8·9 to 15·3)	12·8 (9·5 to 16·5)	13·0 (9·8 to 16·6)	48	1·0 (-0·5 to 2·3)	8·1 (-3·7 to 21·0)
Palestine	11·8 (8·9 to 15·3)	12·2 (9·1 to 15·8)	12·8 (9·8 to 16·5)	12·7 (9·7 to 16·4)	13·1 (10·0 to 16·9)	12·0 (8·9 to 15·6)	96	0·2 (-1·0 to 1·4)	2·1 (-7·8 to 12·2)
Qatar	11·0 (8·1 to 14·6)	12·2 (9·1 to 16·0)	13·7 (10·1 to 17·6)	14·0 (10·4 to 18·0)	14·0 (10·3 to 18·4)	13·8 (10·1 to 17·9)	22	2·8 (1·1 to 4·7)	25·8 (9·3 to 43·3)
Saudi Arabia	11·4 (8·5 to 14·9)	11·8 (8·8 to 15·3)	11·8 (8·9 to 15·4)	12·2 (9·2 to 15·9)	12·4 (9·3 to 16·0)	12·6 (9·4 to 16·6)	67	1·2 (-0·3 to 2·9)	10·7 (-2·3 to 25·1)
Sudan	8·8 (6·3 to 11·8)	10·3 (7·5 to 13·5)	10·8 (7·8 to 14·1)	11·1 (8·1 to 14·5)	11·1 (7·9 to 14·7)	11·1 (7·9 to 14·7)	130	2·3 (0·1 to 4·5)	27·3 (1·5 to 58·3)
Syria	11·8 (8·9 to 15·3)	12·3 (9·3 to 15·9)	13·7 (10·4 to 17·7)	13·8 (10·3 to 17·6)	14·1 (10·7 to 18·2)	14·0 (10·5 to 18·1)	17	2·2 (1·2 to 3·5)	19·3 (9·9 to 29·7)
Tunisia	10·9 (8·3 to 13·8)	11·8 (9·0 to 15·0)	11·9 (9·0 to 15·1)	11·4 (8·6 to 14·5)	12·6 (9·5 to 16·0)	12·7 (9·4 to 16·3)	58	1·9 (0·9 to 2·8)	17·2 (9·2 to 25·4)
Türkiye	11·2 (8·4 to 14·3)	12·9 (9·8 to 16·4)	13·0 (9·9 to 16·5)	12·9 (9·8 to 16·2)	13·1 (10·0 to 16·7)	12·9 (9·6 to 16·7)	53	1·7 (0·1 to 3·3)	15·4 (0·7 to 31·7)
United Arab Emirates	10·8 (8·0 to 13·9)	12·0 (9·0 to 15·4)	12·6 (9·6 to 16·2)	12·7 (9·7 to 16·2)	13·1 (9·8 to 16·7)	13·0 (9·6 to 16·7)	49	2·2 (0·8 to 3·6)	20·8 (7·9 to 35·0)
Yemen	10·9 (7·9 to 14·3)	12·4 (9·0 to 16·2)	13·0 (9·6 to 16·9)	13·3 (9·8 to 17·0)	13·6 (9·9 to 17·7)	13·5 (10·0 to 17·8)	31	2·7 (0·7 to 4·7)	25·0 (5·6 to 46·5)
**South Asia**	**9·4 (6·9 to 12·3)**	**11·2 (8·4 to 14·3)**	**11·6 (8·8 to 14·9)**	**11·5 (8·7 to 14·8)**	**11·9 (8·8 to 15·4)**	**11·8 (8·6 to 15·3)**		**2·4 (0·8 to 4·0)**	**26·0 (8·2 to 44·7)**
Bangladesh	9·0 (6·6 to 11·7)	10·5 (7·8 to 13·6)	10·8 (8·0 to 14·1)	10·8 (7·9 to 14·0)	11·1 (8·2 to 14·5)	10·9 (7·8 to 14·0)	137	1·9 (0·0 to 3·7)	21·6 (-0·2 to 45·0)
Bhutan	8·7 (6·1 to 11·4)	10·3 (7·7 to 13·6)	10·8 (7·9 to 14·2)	10·8 (7·8 to 14·1)	10·9 (7·7 to 14·5)	10·9 (7·9 to 14·4)	135	2·2 (0·3 to 4·3)	26·6 (3·6 to 55·5)
India	9·4 (6·9 to 12·3)	11·3 (8·5 to 14·5)	11·8 (9·0 to 15·1)	11·7 (8·9 to 15·0)	12·1 (8·9 to 15·7)	12·0 (8·8 to 15·7)	98	2·6 (0·7 to 4·4)	28·2 (7·0 to 49·9)
Nepal	9·7 (6·9 to 12·9)	11·3 (8·5 to 14·7)	11·6 (8·6 to 15·0)	11·4 (8·4 to 14·5)	11·9 (8·6 to 15·2)	11·7 (8·4 to 15·2)	107	2·0 (0·2 to 3·9)	21·3 (2·0 to 46·8)
Pakistan	9·6 (6·9 to 12·6)	10·8 (8·0 to 13·9)	10·9 (8·0 to 14·1)	10·9 (7·8 to 14·2)	11·5 (8·3 to 15·3)	11·4 (8·2 to 15·2)	120	1·8 (-0·2 to 3·9)	19·4 (-1·5 to 45·0)
**Southeast Asia, East Asia, and Oceania**	**9·3 (7·0 to 11·8)**	**10·5 (8·0 to 13·4)**	**11·4 (8·7 to 14·5)**	**11·2 (8·5 to 14·1)**	**11·3 (8·4 to 14·6)**	**11·4 (8·5 to 14·6)**		**2·1 (0·9 to 3·2)**	**22·7 (9·8 to 36·0)**
American Samoa	9·6 (7·1 to 12·6)	9·8 (7·3 to 12·7)	10·3 (7·5 to 13·5)	10·1 (7·1 to 13·2)	10·6 (7·5 to 13·9)	10·7 (7·5 to 14·0)	147	1·1 (-0·5 to 2·7)	11·2 (-6·0 to 31·5)
Cambodia	9·0 (6·4 to 11·9)	10·3 (7·5 to 13·3)	10·6 (7·9 to 13·7)	10·7 (7·9 to 14·0)	10·7 (8·0 to 14·0)	10·7 (7·8 to 14·0)	145	1·7 (-0·3 to 3·5)	19·1 (-3·3 to 44·5)
China	9·2 (6·9 to 11·8)	10·7 (8·0 to 13·8)	11·8 (9·1 to 15·1)	11·7 (8·9 to 14·9)	11·7 (8·6 to 15·2)	11·8 (8·7 to 15·2)	104	2·6 (1·0 to 4·0)	28·2 (11·5 to 46·4)
Cook Islands	9·3 (7·0 to 12·1)	10·3 (7·8 to 13·3)	10·6 (8·0 to 13·7)	10·5 (7·8 to 13·6)	10·9 (8·1 to 14·1)	11·0 (8·1 to 14·2)	131	1·7 (0·6 to 2·8)	18·2 (7·3 to 28·9)
Federated States of Micronesia	8·6 (6·5 to 11·2)	9·2 (6·9 to 11·9)	9·6 (7·1 to 12·5)	9·4 (7·0 to 12·2)	9·8 (7·3 to 12·7)	9·9 (7·4 to 12·9)	174	1·3 (0·2 to 2·3)	15·0 (2·7 to 27·3)
Fiji	9·7 (7·4 to 12·6)	9·4 (6·9 to 12·2)	9·7 (7·3 to 12·7)	9·4 (7·1 to 12·3)	9·9 (7·3 to 12·8)	9·9 (7·2 to 13·0)	175	0·1 (-0·8 to 1·2)	1·5 (-8·2 to 12·9)
Guam	9·9 (7·5 to 12·7)	10·8 (8·1 to 13·9)	10·8 (8·2 to 14·1)	10·8 (8·1 to 14·0)	11·0 (8·3 to 14·3)	11·1 (8·3 to 14·4)	127	1·2 (0·3 to 2·2)	12·7 (3·6 to 23·3)
Indonesia	9·7 (7·1 to 12·8)	10·2 (7·4 to 13·3)	10·4 (7·5 to 13·4)	9·9 (7·0 to 13·0)	10·6 (7·6 to 14·0)	10·7 (7·4 to 14·0)	146	1·0 (-1·0 to 2·9)	10·4 (-9·5 to 32·1)
Kiribati	10·4 (7·8 to 13·4)	10·9 (8·2 to 14·0)	11·3 (8·5 to 14·7)	11·0 (8·3 to 14·4)	11·5 (8·6 to 15·0)	11·6 (8·6 to 15·1)	112	1·2 (0·0 to 2·5)	11·8 (0·0 to 24·9)
Laos	6·6 (4·4 to 9·2)	8·0 (5·7 to 10·4)	8·1 (5·8 to 10·7)	8·2 (5·8 to 10·9)	8·2 (5·5 to 11·0)	8·2 (5·3 to 10·9)	203	1·6 (-0·5 to 3·7)	25·9 (-8·2 to 68·5)
Malaysia	9·5 (7·1 to 11·9)	10·2 (7·6 to 13·1)	10·7 (8·1 to 13·7)	10·1 (7·6 to 12·8)	10·5 (7·8 to 13·4)	10·8 (8·1 to 13·7)	139	1·3 (0·5 to 2·3)	14·3 (5·3 to 24·7)
Maldives	8·8 (6·6 to 11·4)	9·6 (7·3 to 12·6)	10·0 (7·6 to 13·0)	9·8 (7·5 to 12·8)	10·0 (7·5 to 13·0)	10·0 (7·6 to 13·0)	170	1·3 (0·3 to 2·3)	14·6 (3·3 to 27·6)
Marshall Islands	8·5 (6·4 to 11·0)	8·5 (6·3 to 11·2)	8·5 (6·3 to 11·1)	8·2 (6·1 to 10·8)	8·6 (6·5 to 11·2)	8·6 (6·4 to 11·4)	200	0·1 (-0·9 to 1·2)	1·3 (-10·7 to 15·3)
Mauritius	10·3 (7·8 to 13·3)	11·2 (8·5 to 14·5)	11·6 (8·8 to 15·0)	11·3 (8·6 to 14·5)	11·4 (8·7 to 14·8)	11·6 (8·7 to 15·0)	111	1·3 (0·4 to 2·3)	13·0 (3·9 to 22·5)
Myanmar	7·9 (5·6 to 10·6)	8·7 (6·2 to 11·3)	9·2 (6·6 to 11·9)	8·9 (6·4 to 11·7)	9·2 (6·5 to 12·1)	9·2 (6·4 to 12·1)	192	1·2 (-0·7 to 3·1)	16·5 (-8·1 to 44·4)
Nauru	7·3 (5·4 to 9·5)	7·2 (5·3 to 9·2)	7·3 (5·4 to 9·7)	7·0 (5·0 to 9·3)	7·5 (5·5 to 9·8)	7·6 (5·5 to 10·0)	204	0·3 (-0·7 to 1·4)	4·2 (-9·8 to 19·3)
Niue	9·3 (6·9 to 11·9)	9·8 (7·3 to 12·5)	10·2 (7·8 to 13·1)	9·9 (7·5 to 12·7)	9·9 (7·4 to 12·9)	10·0 (7·5 to 13·1)	169	0·8 (-0·1 to 1·7)	8·5 (-1·6 to 19·6)
North Korea	9·6 (7·1 to 12·6)	9·8 (7·3 to 12·8)	10·2 (7·7 to 13·5)	10·2 (7·5 to 13·3)	10·2 (7·3 to 13·3)	10·3 (7·5 to 13·6)	159	0·7 (-0·9 to 2·4)	7·3 (-9·3 to 27·2)
Northern Mariana Islands	9·0 (6·8 to 11·6)	9·2 (6·9 to 11·9)	9·4 (7·1 to 12·2)	9·1 (6·9 to 11·7)	9·6 (7·1 to 12·5)	9·7 (7·2 to 12·6)	177	0·8 (-0·2 to 1·7)	8·6 (-2·1 to 19·9)
Palau	8·8 (6·5 to 11·3)	9·3 (7·0 to 12·1)	9·3 (7·0 to 12·1)	9·1 (6·8 to 11·8)	9·5 (7·1 to 12·5)	9·6 (7·1 to 12·4)	180	0·8 (-0·1 to 1·8)	9·4 (-1·3 to 20·8)
Papua New Guinea	8·4 (6·1 to 11·2)	8·5 (6·1 to 11·1)	8·9 (6·3 to 11·8)	8·9 (6·4 to 11·8)	8·9 (6·0 to 11·9)	8·9 (6·2 to 11·9)	196	0·5 (-1·3 to 2·3)	6·5 (-15·4 to 30·8)
Philippines	9·9 (7·5 to 12·8)	9·8 (7·5 to 12·5)	10·1 (7·7 to 13·0)	9·2 (7·0 to 11·8)	10·0 (7·6 to 12·8)	10·2 (7·7 to 13·1)	163	0·3 (-0·4 to 1·1)	3·3 (-4·0 to 11·7)
Samoa	10·2 (7·7 to 13·2)	10·3 (7·7 to 13·1)	10·3 (7·9 to 13·1)	10·1 (7·6 to 12·9)	10·5 (7·9 to 13·3)	10·5 (7·9 to 13·3)	151	0·3 (-0·7 to 1·2)	2·9 (-7·1 to 13·0)
Seychelles	9·7 (7·2 to 12·5)	10·1 (7·6 to 13·0)	10·8 (8·1 to 14·2)	9·7 (7·2 to 12·8)	10·6 (7·9 to 13·8)	10·7 (8·0 to 14·0)	141	1·1 (0·1 to 2·0)	11·2 (1·3 to 21·6)
Solomon Islands	7·7 (5·8 to 10·1)	8·2 (6·1 to 10·6)	8·1 (6·0 to 10·4)	8·2 (6·0 to 10·6)	8·2 (6·0 to 10·6)	8·2 (5·9 to 10·7)	202	0·5 (-0·5 to 1·6)	6·9 (-6·0 to 21·6)
Sri Lanka	10·1 (7·6 to 12·9)	10·6 (8·0 to 13·5)	11·1 (8·5 to 14·2)	10·7 (8·2 to 13·6)	10·9 (8·3 to 13·8)	11·0 (8·4 to 14·0)	132	0·9 (0·1 to 1·6)	8·9 (1·1 to 16·9)
Taiwan*	10·1 (7·7 to 13·0)	11·9 (9·0 to 15·3)	12·7 (9·8 to 16·1)	12·7 (9·8 to 16·1)	12·5 (9·6 to 15·9)	12·8 (9·8 to 16·3)	56	2·7 (1·9 to 3·5)	26·4 (21·0 to 31·3)
Thailand	10·4 (7·8 to 13·1)	10·5 (7·9 to 13·4)	11·4 (8·7 to 14·4)	11·2 (8·6 to 14·2)	11·2 (8·5 to 14·1)	11·4 (8·7 to 14·4)	119	1·1 (0·5 to 1·7)	10·5 (4·8 to 16·6)
Timor-Leste	7·8 (5·3 to 10·3)	9·0 (6·5 to 11·6)	9·0 (6·6 to 11·6)	9·0 (6·4 to 11·7)	9·1 (6·5 to 12·0)	9·1 (6·3 to 11·9)	193	1·3 (-0·7 to 3·1)	18·2 (-8·9 to 48·4)

Tokelau	9·9 (7·4 to 12·8)	10·3 (7·7 to 13·4)	10·8 (8·2 to 14·1)	10·6 (8·0 to 13·9)	10·6 (8·0 to 13·9)	10·7 (8·1 to 13·9)	142	0·8 (-0·1 to 1·8)	8·4 (-1·3 to 18·8)
Tonga	10·2 (7·6 to 13·2)	10·1 (7·5 to 13·2)	10·1 (7·5 to 13·3)	9·7 (7·1 to 12·7)	10·2 (7·5 to 13·3)	10·3 (7·4 to 13·7)	158	0·1 (-0·9 to 1·1)	1·4 (-8·6 to 11·9)
Tuvalu	8·2 (6·3 to 10·7)	8·6 (6·5 to 11·3)	8·6 (6·3 to 11·2)	8·5 (6·2 to 11·1)	8·8 (6·5 to 11·4)	8·8 (6·5 to 11·5)	199	0·6 (-0·5 to 1·7)	7·1 (-6·0 to 21·3)
Vanuatu	9·5 (7·2 to 12·2)	9·6 (7·3 to 12·5)	9·7 (7·3 to 12·5)	9·7 (7·4 to 12·5)	9·9 (7·5 to 12·7)	9·9 (7·4 to 12·7)	172	0·4 (-0·6 to 1·4)	4·6 (-6·7 to 16·8)
Viet Nam	10·1 (7·5 to 13·1)	10·5 (7·8 to 13·6)	10·7 (7·8 to 13·9)	10·6 (7·7 to 13·8)	10·8 (7·7 to 14·0)	10·9 (7·8 to 14·0)	136	0·8 (-1·0 to 2·7)	8·5 (-9·9 to 28·5)
**Sub-Saharan Africa**	**8·4 (6·3 to 10·8)**	**9·2 (7·0 to 11·8)**	**10·0 (7·6 to 12·8)**	**10·0 (7·7 to 12·7)**	**10·3 (7·8 to 13·2)**	**10·2 (7·7 to 13·2)**		**1·8 (1·0 to 2·7)**	**22·1 (13·4 to 32·2)**
Angola	8·7 (6·2 to 11·4)	10·3 (7·4 to 13·4)	10·9 (8·1 to 14·1)	10·9 (7·9 to 14·2)	11·2 (8·1 to 14·8)	11·1 (7·8 to 14·7)	128	2·4 (0·3 to 4·7)	28·1 (3·8 to 58·5)
Benin	9·0 (6·3 to 12·0)	10·1 (7·5 to 13·3)	10·5 (7·7 to 13·7)	10·7 (7·8 to 14·1)	10·7 (7·7 to 14·2)	10·7 (7·5 to 14·1)	144	1·6 (-0·4 to 3·8)	19·0 (-3·9 to 48·5)
Botswana	10·4 (7·8 to 13·1)	11·0 (8·3 to 14·2)	11·3 (8·5 to 14·5)	10·4 (7·8 to 13·4)	11·5 (8·6 to 14·8)	11·6 (8·7 to 14·9)	109	1·3 (0·2 to 2·4)	12·2 (1·8 to 22·9)
Burkina Faso	8·0 (5·4 to 10·6)	9·8 (7·2 to 12·8)	10·9 (8·0 to 13·9)	11·4 (8·3 to 14·8)	11·2 (7·9 to 14·6)	10·8 (7·8 to 14·2)	138	2·8 (0·7 to 4·9)	36·6 (8·2 to 69·9)
Burundi	7·1 (5·0 to 9·7)	8·9 (6·4 to 11·6)	9·4 (6·9 to 12·3)	9·3 (6·8 to 12·2)	9·6 (6·9 to 12·5)	9·5 (6·5 to 12·5)	185	2·4 (0·1 to 4·4)	34·6 (1·5 to 75·6)
Cabo Verde	10·6 (7·9 to 14·0)	11·6 (8·8 to 15·0)	11·7 (8·6 to 15·3)	11·8 (8·7 to 15·5)	12·0 (8·9 to 15·5)	11·8 (8·6 to 15·4)	101	1·2 (-0·4 to 3·0)	11·4 (-3·3 to 29·7)
Cameroon	8·7 (6·2 to 11·5)	8·3 (6·0 to 10·8)	9·0 (6·6 to 11·8)	8·8 (6·4 to 11·5)	9·4 (6·7 to 12·2)	9·4 (6·6 to 12·3)	188	0·7 (-1·2 to 2·6)	8·3 (-12·6 to 33·2)
Central African Republic	8·3 (5·8 to 11·0)	8·0 (5·7 to 10·6)	9·2 (6·8 to 12·0)	9·5 (6·7 to 12·4)	9·5 (6·7 to 12·8)	9·4 (6·6 to 12·4)	187	1·1 (-1·0 to 3·2)	14·1 (-10·9 to 43·5)
Chad	7·1 (4·9 to 9·6)	8·1 (5·8 to 10·4)	8·7 (6·4 to 11·3)	9·1 (6·5 to 12·0)	8·9 (6·2 to 11·6)	8·8 (6·3 to 11·7)	198	1·8 (-0·1 to 3·6)	26·0 (-1·5 to 58·1)
Comoros	10·5 (7·8 to 13·9)	11·5 (8·7 to 15·0)	12·0 (8·9 to 15·7)	11·7 (8·4 to 15·2)	12·4 (8·8 to 16·4)	12·4 (8·9 to 16·3)	80	1·8 (0·0 to 3·7)	17·7 (-0·1 to 38·1)
Congo (Brazzaville)	8·9 (6·2 to 11·9)	9·9 (7·1 to 12·9)	10·2 (7·5 to 13·2)	10·2 (7·3 to 13·2)	10·5 (7·5 to 13·6)	10·2 (7·3 to 13·5)	162	1·4 (-0·7 to 3·2)	16·2 (-6·5 to 40·5)
Côte d'Ivoire	9·2 (6·6 to 12·2)	9·3 (6·7 to 12·1)	10·2 (7·5 to 13·2)	10·6 (7·7 to 13·7)	10·5 (7·4 to 13·6)	10·3 (7·3 to 13·5)	160	1·1 (-0·9 to 3·1)	12·7 (-9·7 to 36·9)
DR Congo	9·4 (6·6 to 12·3)	9·6 (7·2 to 12·6)	9·9 (7·2 to 12·9)	10·2 (7·4 to 13·2)	10·2 (7·1 to 13·2)	10·0 (7·0 to 12·9)	171	0·6 (-1·3 to 2·6)	7·4 (-13·3 to 31·5)
Djibouti	9·0 (6·7 to 11·9)	9·3 (6·8 to 12·3)	9·9 (7·2 to 13·1)	9·8 (6·9 to 12·9)	10·1 (7·0 to 13·6)	10·1 (6·9 to 13·3)	166	1·1 (-0·7 to 3·0)	12·7 (-8·6 to 37·5)
Equatorial Guinea	8·2 (5·8 to 11·0)	9·2 (6·6 to 11·8)	9·7 (7·0 to 12·7)	9·9 (7·0 to 13·3)	9·8 (6·9 to 13·3)	9·6 (6·6 to 12·9)	179	1·4 (-0·6 to 3·5)	17·6 (-8·2 to 48·9)
Eritrea	7·8 (5·5 to 10·6)	9·2 (6·6 to 12·2)	9·6 (7·1 to 12·7)	9·8 (7·1 to 12·8)	9·6 (6·8 to 12·8)	9·6 (6·6 to 12·8)	181	1·8 (-0·3 to 4·0)	23·3 (-4·2 to 57·6)
Eswatini	8·2 (5·9 to 10·9)	7·8 (5·3 to 10·2)	8·8 (6·2 to 11·5)	8·6 (6·0 to 11·4)	9·5 (6·5 to 12·8)	9·7 (6·7 to 13·1)	178	1·5 (-0·8 to 3·6)	18·8 (-9·1 to 49·4)
Ethiopia	7·0 (4·8 to 9·4)	8·7 (6·3 to 11·3)	9·5 (6·8 to 12·3)	9·6 (6·8 to 12·5)	9·7 (6·9 to 12·8)	9·5 (6·8 to 12·5)	184	2·5 (0·6 to 4·5)	37·0 (7·9 to 74·2)
Gabon	10·4 (7·7 to 13·6)	10·6 (7·8 to 13·8)	10·9 (7·9 to 14·0)	11·1 (7·7 to 14·4)	11·3 (8·1 to 14·7)	11·1 (7·8 to 14·7)	129	0·7 (-1·1 to 2·4)	6·6 (-10·4 to 25·7)
Ghana	8·9 (6·3 to 11·7)	9·7 (7·2 to 12·8)	10·0 (7·4 to 13·2)	10·1 (7·3 to 13·4)	10·2 (7·3 to 13·6)	10·1 (7·1 to 13·7)	165	1·2 (-0·7 to 3·2)	14·3 (-7·8 to 39·9)
Guinea	7·1 (4·7 to 9·5)	8·7 (6·3 to 11·4)	9·4 (6·8 to 12·2)	9·7 (7·0 to 12·8)	9·6 (6·8 to 12·6)	9·4 (6·4 to 12·5)	186	2·3 (0·1 to 4·6)	34·3 (1·6 to 79·1)
Guinea-Bissau	7·9 (5·6 to 10·6)	8·6 (6·0 to 11·4)	9·3 (6·8 to 12·1)	9·6 (6·8 to 12·7)	9·5 (6·7 to 12·6)	9·4 (6·6 to 12·1)	189	1·4 (-0·6 to 3·5)	18·9 (-6·7 to 49·4)
Kenya	9·4 (6·7 to 12·1)	9·6 (7·0 to 12·6)	10·4 (7·7 to 13·5)	10·3 (7·5 to 13·7)	10·9 (8·0 to 14·3)	10·7 (7·7 to 14·0)	143	1·3 (-0·6 to 3·2)	14·7 (-6·5 to 38·0)
Lesotho	8·3 (6·1 to 10·8)	8·3 (6·0 to 10·9)	8·8 (6·4 to 11·5)	8·5 (6·1 to 11·3)	9·2 (6·3 to 12·3)	9·2 (6·1 to 12·4)	191	0·9 (-1·1 to 3·1)	11·5 (-13·2 to 39·5)
Liberia	8·6 (6·0 to 11·4)	9·6 (7·0 to 12·8)	9·7 (6·9 to 12·7)	9·3 (6·6 to 12·4)	10·1 (7·3 to 13·4)	10·0 (7·1 to 13·4)	167	1·4 (-0·7 to 3·7)	17·8 (-8·0 to 49·4)
Madagascar	7·2 (5·0 to 9·7)	8·2 (5·8 to 10·6)	8·7 (6·3 to 11·3)	8·8 (6·4 to 11·7)	8·7 (6·2 to 11·3)	8·6 (6·0 to 11·4)	201	1·4 (-0·3 to 3·2)	20·6 (-4·2 to 52·7)
Malawi	6·7 (4·5 to 9·1)	8·6 (6·2 to 11·2)	9·3 (6·4 to 12·4)	8·4 (5·7 to 11·1)	9·4 (6·5 to 12·6)	9·3 (6·3 to 12·3)	190	2·6 (0·3 to 4·7)	40·8 (4·0 to 84·9)
Mali	7·7 (5·2 to 10·6)	9·9 (7·1 to 12·9)	10·2 (7·6 to 13·3)	10·4 (7·6 to 13·7)	10·6 (7·7 to 13·9)	10·6 (7·4 to 13·9)	149	2·9 (0·7 to 5·1)	38·7 (8·4 to 81·4)
Mauritania	9·0 (6·6 to 12·1)	9·7 (7·2 to 12·7)	9·9 (7·3 to 13·0)	10·0 (7·3 to 13·3)	10·1 (7·3 to 13·3)	10·0 (7·2 to 13·3)	168	1·0 (-0·7 to 2·7)	11·6 (-7·3 to 34·7)
Mozambique	8·9 (6·1 to 11·9)	9·9 (7·1 to 12·8)	10·9 (7·9 to 14·3)	10·6 (7·7 to 13·9)	11·2 (8·1 to 14·9)	11·2 (8·0 to 14·9)	122	2·3 (0·3 to 4·5)	26·7 (3·1 to 56·4)
Namibia	9·1 (7·0 to 11·8)	9·5 (7·1 to 12·4)	10·0 (7·4 to 12·9)	9·0 (6·7 to 11·6)	10·3 (7·7 to 13·3)	10·5 (7·7 to 13·6)	156	1·3 (0·1 to 2·5)	14·5 (1·7 to 28·3)
Niger	6·2 (4·0 to 8·8)	8·1 (5·8 to 10·6)	8·7 (6·4 to 11·3)	9·0 (6·5 to 11·8)	8·9 (6·4 to 11·9)	8·9 (6·4 to 11·7)	197	2·7 (0·4 to 5·0)	47·5 (6·1 to 107·3)
Nigeria	8·2 (5·6 to 11·1)	9·4 (6·8 to 12·4)	10·3 (7·6 to 13·5)	10·6 (7·9 to 13·9)	10·7 (7·7 to 13·9)	10·5 (7·4 to 13·8)	153	2·2 (0·3 to 4·3)	28·5 (3·3 to 62·4)
Rwanda	6·6 (4·6 to 8·9)	9·4 (7·0 to 12·3)	9·6 (7·2 to 12·5)	9·6 (7·0 to 12·5)	9·7 (7·0 to 12·7)	9·6 (6·7 to 12·7)	182	3·0 (0·9 to 5·0)	46·5 (13·2 to 89·2)
Senegal	9·4 (6·6 to 12·4)	10·7 (7·8 to 14·0)	11·1 (8·1 to 14·3)	11·2 (8·1 to 14·6)	11·4 (8·1 to 14·8)	11·2 (8·0 to 14·8)	123	1·8 (-0·2 to 3·7)	19·7 (-2·1 to 44·3)
Sierra Leone	7·5 (5·2 to 10·1)	8·6 (6·2 to 11·3)	9·4 (6·8 to 12·4)	9·7 (6·8 to 12·6)	9·6 (6·9 to 12·6)	9·5 (6·7 to 12·4)	183	2·0 (0·1 to 4·1)	27·9 (1·9 to 61·5)
Somalia	8·5 (5·8 to 11·3)	7·9 (5·5 to 10·5)	10·4 (7·6 to 13·6)	10·0 (7·1 to 13·4)	10·6 (7·6 to 14·0)	10·4 (7·4 to 13·8)	157	2·0 (-0·1 to 4·2)	24·5 (-1·7 to 57·0)
South Africa	11·5 (8·5 to 15·0)	9·3 (7·0 to 12·0)	10·9 (8·3 to 13·9)	10·4 (8·0 to 13·3)	11·4 (8·6 to 14·6)	11·5 (8·7 to 14·6)	117	0·1 (-1·3 to 1·4)	0·7 (-10·6 to 14·2)
South Sudan	8·2 (5·7 to 11·1)	8·8 (6·5 to 11·6)	9·3 (6·7 to 12·2)	9·6 (7·0 to 12·7)	9·3 (6·6 to 12·3)	9·1 (6·2 to 12·1)	194	0·9 (-1·2 to 2·9)	11·6 (-14·0 to 40·4)
São Tomé and Príncipe	9·3 (6·9 to 12·4)	9·7 (7·0 to 12·7)	9·8 (7·1 to 12·7)	10·1 (7·2 to 13·0)	10·4 (7·6 to 13·7)	10·5 (7·2 to 13·8)	152	1·2 (-0·6 to 2·9)	13·0 (-6·2 to 33·9)
Tanzania	8·6 (6·1 to 11·3)	9·3 (6·8 to 12·2)	10·3 (7·5 to 13·6)	9·9 (7·2 to 13·1)	10·8 (7·8 to 14·2)	10·8 (7·7 to 14·2)	140	2·2 (0·2 to 4·2)	26·6 (2·2 to 56·3)
The Gambia	9·6 (7·0 to 12·4)	10·3 (7·5 to 13·6)	10·1 (7·2 to 13·2)	10·2 (7·2 to 13·3)	10·6 (7·6 to 13·8)	10·6 (7·6 to 13·9)	150	1·0 (-0·8 to 2·8)	10·8 (-8·5 to 32·1)
Togo	8·8 (6·3 to 11·7)	9·3 (6·8 to 12·2)	10·0 (7·4 to 13·2)	10·2 (7·5 to 13·4)	10·3 (7·5 to 13·6)	10·3 (7·3 to 13·8)	161	1·4 (-0·5 to 3·5)	16·9 (-5·5 to 44·1)
Uganda	7·6 (5·3 to 10·2)	9·0 (6·6 to 11·8)	9·7 (7·1 to 12·6)	9·4 (6·7 to 12·4)	9·9 (7·1 to 13·3)	9·9 (7·0 to 13·1)	173	2·3 (0·3 to 4·4)	31·5 (4·2 to 66·1)
Zambia	7·1 (4·9 to 9·6)	8·2 (6·0 to 10·8)	8·9 (6·4 to 11·8)	8·6 (6·0 to 11·5)	9·3 (6·4 to 12·4)	9·1 (6·4 to 12·1)	195	2·0 (-0·1 to 4·2)	29·7 (-1·3 to 72·6)
Zimbabwe	10·3 (7·6 to 13·5)	8·9 (6·6 to 11·7)	10·2 (7·4 to 13·5)	9·6 (6·7 to 13·0)	10·6 (7·7 to 14·1)	10·7 (7·4 to 14·1)	148	0·4 (-1·6 to 2·4)	4·0 (-15·4 to 25·3)
**Low SDI**	**8·5 (6·4 to 11·0)**	**9·8 (7·4 to 12·5)**	**10·4 (8·0 to 13·5)**	**10·4 (7·9 to 13·4)**	**10·7 (8·1 to 13·9)**	**10·5 (7·9 to 13·6)**		**2·1 (1·2 to 3·0)**	**24·7 (15·2 to 36·6)**
**Low-middle SDI**	**9·4 (7·0 to 12·3)**	**10·8 (8·2 to 13·8)**	**11·2 (8·4 to 14·4)**	**11·1 (8·4 to 14·3)**	**11·5 (8·6 to 14·9)**	**11·4 (8·6 to 14·8)**		**2·0 (0·8 to 3·2)**	**21·3 (8·0 to 35·3)**

**Middle SDI**	**9·9 (7·6 to 12·7)**	**10·7 (8·1 to 13·6)**	**11·1 (8·5 to 14·2)**	**10·9 (8·3 to 13·9)**	**11·3 (8·6 to 14·5)**	**11·4 (8·6 to 14·7)**	**1·4 (0·6 to 2·3)**	**14·7 (6·7 to 23·0)**
**High-middle SDI**	**9·9 (7·5 to 12·8)**	**11·3 (8·6 to 14·4)**	**12·1 (9·3 to 15·6)**	**12·0 (9·2 to 15·3)**	**12·4 (9·4 to 16·0)**	**12·5 (9·4 to 16·1)**	**2·5 (1·5 to 3·6)**	**25·5 (16·1 to 35·9)**
**High SDI**	**11·1 (8·4 to 14·1)**	**12·1 (9·3 to 15·3)**	**12·8 (9·8 to 16·3)**	**12·7 (9·8 to 16·3)**	**13·0 (10·0 to 16·6)**	**13·2 (10·1 to 16·9)**	**2·1 (1·4 to 2·8)**	**18·7 (13·7 to 23·9)**

*United Nations convention recognises Taiwan as a province of China
