# Supplementary material for: Global, regional, and national trends in the morbidity gap and contributing diseases, injuries, and risk factors, 1990–2023: a systematic analysis for the Global Burden of Disease Study 2023
Source: Lancet Public Health. 2026 Jul 21;11(8):e487–505. doi: 10.1016/S2468-2667(26)00098-8 (PMC13752251; doi:10.1016/S2468-2667(26)00098-8)
Supplement: Supplementary file 2 [file mmc2.pdf]

THE LANCET
Public Health

Supplementary appendix 2

This appendix formed part of the original submission and has been peer reviewed. We post it as supplied by the authors.

Supplement to: Hay SI, Nam P, Saqib H, et al. Global, regional, and national trends in the morbidity gap and contributing diseases, injuries, and risk factors, 1990–2023: a systematic analysis for the Global Burden of Disease Study 2023. *Lancet Public Health* 2026; **11:** e487–505.

Appendix 2: Interpreting period life-table survival curves in "Global, regional, and national trends in the morbidity gap and contributing diseases, injuries, and risk factors, 1990–2023: a systematic analysis for the Global Burden of Disease Study 2023"

Table of Contents

Section 1: Appendix overview....3

Section 2: Description of period life-table survival curves....3

Section 3: What a survival plot represents....3

Section 4: Interpreting individual country panels....4

Section 4.1: Early childhood (birth to age 5) 4

Section 4.2: Adolescence and working ages (approximately 15–60 years) 4

Section 4.3: Older ages and terminal decline 4

Section 5: Interpreting temporal trends 5

Section 6: Interpreting health-adjusted survival and the morbidity gap 5

Section 7: Key points....5

Section 8: Bibliography 5

Section 9: Figures 7

Section 1: Appendix overview

The appendix presents a complementary framework for interpreting the divergence between gains in life expectancy and healthy life expectancy across countries. To aid in the interpretation of the survival curves presented in the main text, we have provided a plain-language overview here. It explains how to read the plots, illustrates their value through selected country narratives, and clarifies how these visualisations deepen understanding of demographic change. Together, these examples reinforce our central findings at the national level, while highlighting the heterogeneity and complexity of health and survival transitions worldwide.

Section 2: Description of period life-table survival curves

Section 9 (below) presents figures of country-specific (for all 204 countries and territories in GBD 2023[1]) survival probability (from 0 to 1) for 1990 and 2023, by age (0 to 95 years). The blue line in each panel is the period life-table survival curve (hereafter simply survival curve(s)), which represents the proportion of a synthetic birth cohort expected to be alive at each age under the mortality conditions of the specified year.[2–4] A survival probability of 0·90 at age 50 indicates that 90% of the cohort is expected to survive to age 50. The survival curves are shown up to age 95 to facilitate comparison of mortality patterns at older ages.

Each panel also includes a health-adjusted survival curve (red), representing the proportion of the cohort expected to be alive and in full health at each age, using the same disability weighting framework applied in the estimation of healthy life expectancy (HALE).[5,6] The shaded area between the survival curve and the health-adjusted survival curve represents the morbidity gap (MG), that is, the accumulation of years lived with disability among those who survive.[7–9] For ease of interpretation, the total duration of the MG, expressed in years, is reported numerically in the bottom left of each panel. Dashed reference curves are included to aid visual comparison between 1990 and 2023 by allowing each year's trajectory to be viewed in relation to the other within the same panel. All 204 countries and territories are shown in alphabetical order.

Section 3: What a survival plot represents

Survival curves provide a concise summary of mortality across the full life course, without collapsing age-specific patterns into a single metric such as life expectancy.[7,10] They complement disease-specific outcomes, case fatality rates, and post-diagnosis survival by reflecting the cumulative effect of all causes of death operating within a population over time.[1] When paired with health-adjusted survival, these plots extend beyond mortality to incorporate non-fatal health loss. They therefore allow readers to assess not only whether people are living longer, but also whether additional years of life are being lived in good health or with disability, a distinction that is central to the interpretation of trends in HALE and to the core findings of this analysis.[7–9]

Section 4: Interpreting individual country panels

Section 4.1: Early childhood (birth to age 5)

The initial segment of the curve reflects mortality in infancy and early childhood. In very high-survival settings, the survival curve remains close to 1·0 throughout this period, as seen in Singapore in both 1990 and 2023. In contrast, visible early declines, such as those observed in the Central African Republic, indicate substantial mortality before age five. Several countries show especially large improvements in the early-life segment between 1990 and 2023, with the survival curve in 2023 beginning much closer to 1·0 than in 1990. Notable examples include Rwanda, Ethiopia, Sierra Leone, South Sudan, and Madagascar, each of which shows a pronounced early-life drop in 1990 that is substantially attenuated by 2023, indicating major gains in child survival across the first five years of life.[6,11]

Section 4.2: Adolescence and working ages (approximately 15–60 years)

The slope of the survival curve across young and middle adulthood reflects premature adult mortality. A relatively flat trajectory indicates low mortality in these ages, whereas a progressive downward slope indicates substantial losses from injuries, infectious diseases, maternal causes, or earlier onset non-communicable diseases. The USA illustrates how survival through mid-adulthood can be less favourable than expected for a high-income country, with excess mortality in working ages that has been linked to deaths of despair. In several settings affected by major crises, the working-age segment provides a clear visual signal of excess adult mortality. Afghanistan, Iraq, Syria, Yemen, and Palestine show steeper declines through adolescence and working ages, with less concentration of mortality at older ages than seen in stable high-survival settings, consistent with elevated adult mortality during periods of conflict and prolonged instability. South Sudan shows a similar pattern, with substantial survival losses persisting across working ages despite improvements in early-childhood survival. By contrast, in southern Africa, the defining feature of the curve is the pronounced attrition through ages 20–60 in South Africa and Lesotho, a pattern classically associated with high adult mortality from the HIV epidemic and its downstream consequences, which continues to shape overall survival even as child survival improves.

Section 4.3: Older ages and terminal decline

Across countries, the distinguishing feature at older ages is not that mortality increases, but how late in life survival begins to fall and how concentrated deaths are once decline begins. In high-life expectancy settings, the survival curve remains elevated into the seventh or eighth decade and then drops steeply over a relatively narrow age range, indicating strong concentration of mortality in late life. Switzerland and Singapore exemplify this pattern, with minimal attrition through earlier adulthood and a sharp terminal decline occurring predominantly at older ages. Similar late-life concentration is evident in Japan, Spain, Italy, and Australia. Despite these favourable survival profiles, the plots show a consistent widening of the morbidity gap in these settings. The health-adjusted survival curve diverges progressively from the survival curve across older ages, indicating that gains in longevity have not been matched by equivalent gains in healthy survival. As a result, a growing proportion of later life is spent with disability, demonstrating that pronounced late-life concentration of mortality can coexist with expansion of morbidity.

Section 5: Interpreting temporal trends

Comparison between 1990 and 2023 should focus on whether the survival curve has shifted upward, indicating higher survival at a given age, and whether it has shifted rightward, indicating postponement of mortality to later ages. Australia provides a clear example of sustained improvement, with survival increasing across most ages and deaths occurring later in life. In practice, purely upward or purely rightward shifts are uncommon, and most countries exhibit a combination of both. In lower- and middle-income settings, improvements are often dominated by upward shifts at young ages, reflecting large reductions in under-5 mortality, as seen in Rwanda, Ethiopia, Sierra Leone, and Madagascar, where early-life survival improves markedly between 1990 and 2023 with more modest change in the timing of later-life mortality. By contrast, in high-income settings, change is driven primarily by rightward shifts at older ages, with limited scope for improvement in early-life survival; this pattern is evident in Japan, Switzerland, Spain, and Italy, where mortality is increasingly concentrated at older ages.

Section 6: Interpreting health-adjusted survival and the morbidity gap

The separation between the survival curve and the health-adjusted survival curve reflects the accumulation of non-fatal health loss across the life course (ie, the morbidity gap [MG]).[5,6] This separation often begins well before mortality accelerates, indicating that disability typically precedes death by many years. The shaded area therefore captures both the timing and the duration of morbidity among survivors, with the total duration summarised numerically in each panel. A central finding of this analysis is that, between 1990 and 2023, the morbidity gap increased in every country and territory included in GBD 2023, with the sole exception of Palestine.[6] In other words, gains in survival have not been matched by equivalent gains in healthy survival, resulting in longer periods lived with disability.[8,9] In high-income countries such as Switzerland and Australia, this expansion of the MG largely reflects prolonged survival into older ages combined with persistent chronic disease burden.[7,10,12] In lower-income settings, including the Central African Republic and Lesotho, the widening gap often reflects earlier onset of disability, persistent infectious causes, injuries, and chronic conditions occurring at younger ages.

Section 7: Key points

These examples allow readers to identify the contribution of early-life mortality, assess survival through working ages, observe the age concentration of deaths in later life, and evaluate both the magnitude and duration of the morbidity gap, for all 204 countries and territories. Read together, these plots show that while survival has improved almost everywhere since 1990, the years spent in poor health have increased in nearly all countries.

Section 8: Bibliography

1 Schumacher A, Zheng P, Barber RM. Global age-sex-specific all-cause mortality and life expectancy estimates for 204 countries and territories and 660 subnational locations, 1950-2023: a demographic analysis for the Global Burden of Disease Study 2023. *The Lancet* 2025; **406**: 1731–810.

2 Harper S. Demography: a very short introduction, First edition. Oxford: Oxford University Press, 2018.

3 Murray CJ, Lopez AD. Global mortality, disability, and the contribution of risk factors: Global Burden of Disease Study. *The Lancet* 1997; **349**: 1436–42.

4 Dicker D, Nguyen G, Abate D, *et al.* Global, regional, and national age-sex-specific mortality and life expectancy, 1950–2017: a systematic analysis for the Global Burden of Disease Study 2017. *The Lancet* 2018; **392**: 1684–735.

5 Salomon JA, Vos T, Hogan DR, *et al.* Common values in assessing health outcomes from disease and injury: disability weights measurement study for the Global Burden of Disease Study 2010. *The Lancet* 2012; **380**: 2129–43.

6 Hay SI, Ong LK, Santomauro DF. Burden of 375 diseases and injuries, risk-attributable burden of 88 risk factors, and healthy life expectancy in 204 countries and territories, including 660 subnational locations, 1990-2023: a systematic analysis for the Global Burden of Disease Study 2023. *The Lancet* 2025; **406**: 1873–922.

7 Fries JF. Aging, natural death, and the compression of morbidity. *N Engl J Med* 1980; **303**: 130–5.

8 Gruenberg EM. The failures of success. *Milbank Q* 2005; **83**: 779–800.

9 Crimmins EM, Beltran-Sanchez H. Mortality and morbidity trends: is there compression of morbidity? *J Gerontol B Psychol Sci Soc Sci* 2011; **66B**: 75–86.

10 Nusselder WJ, Mackenbach JP. Rectangularization of the survival curve in the Netherlands, 1950-1992. *The Gerontologist* 1996; **36**: 773–82.

11 Burstein R, Henry NJ, Collison ML, *et al.* Mapping 123 million neonatal, infant and child deaths between 2000 and 2017. *Nature* 2019; **574**: 353–8.

12 Manton KG. Changing concepts of morbidity and mortality in the elderly population. *Milbank Mem Fund Q Health Soc* 1982; **60**: 183–244.

Section 9: Figures

Figure S1. Survival and health-adjusted survival probability by age, for 204 countries and territories, 1990 and 2023

Survival probability (blue) and health-adjusted survival probability (red) are shown by single year of age from birth to 95 years for 204 countries and territories, in alphabetical order. Panels show estimates for 1990 and 2023; dotted curves are included to aid visual comparison between time periods. The grey shaded area between the survival and health-adjusted survival curves represents the morbidity gap across the life course.

Numbers in the lower left of each panel indicate the average morbidity gap in years.

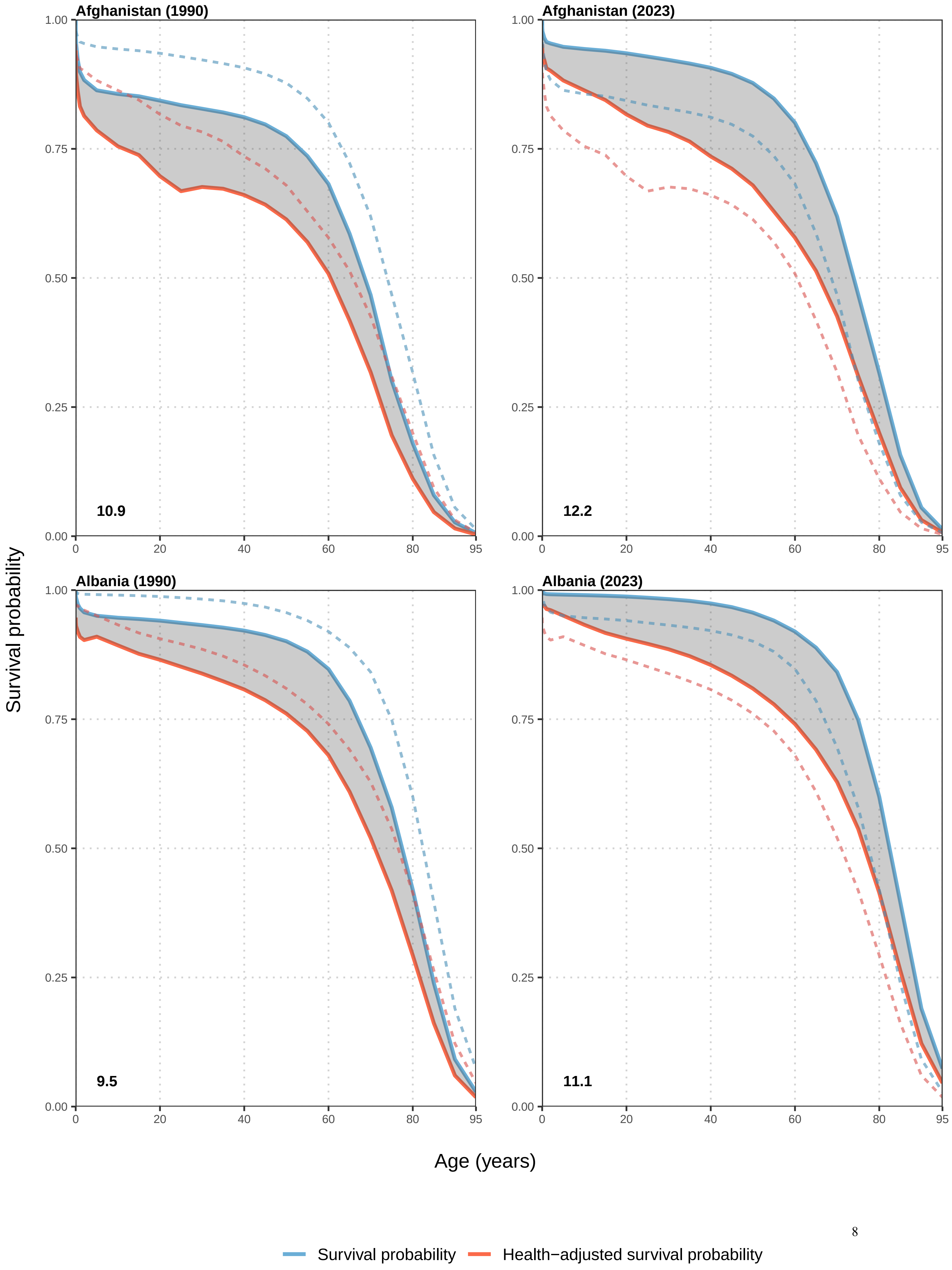
Survival probability
Afghanistan (1990)
10.9
Afghanistan (2023)
12.2
Albania (1990)
9.5
Albania (2023)
11.1
1.00
0.75
0.50
0.25
0.00
0
20
40
60
80
95
Age (years)
Survival probability
Health−adjusted survival probability

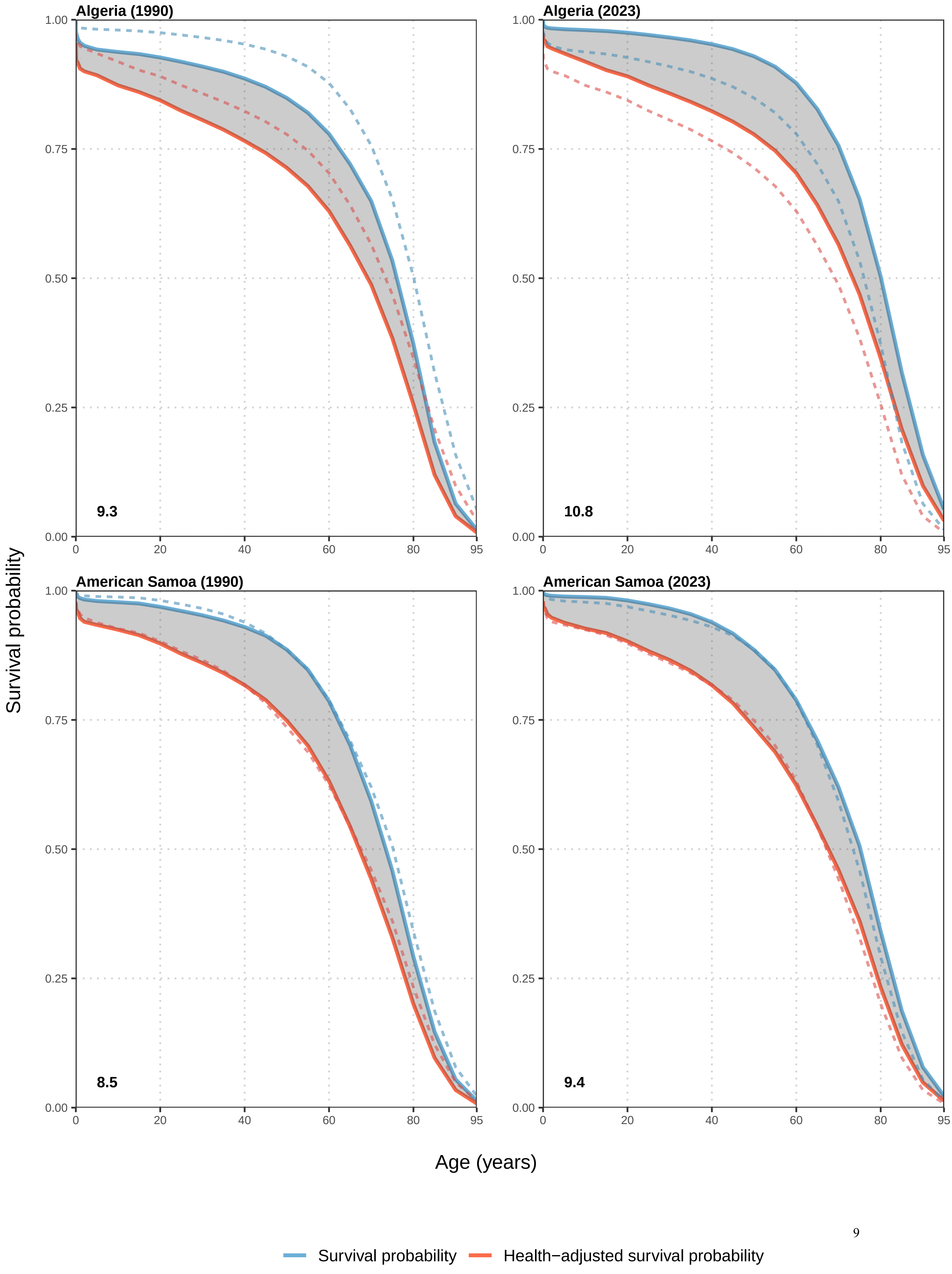
Algeria (1990)
9.3
Algeria (2023)
10.8
American Samoa (1990)
8.5
American Samoa (2023)
9.4
Survival probability
Age (years)
1.00
0.75
0.50
0.25
0.00
0
20
40
60
80
95
Survival probability
Health−adjusted survival probability

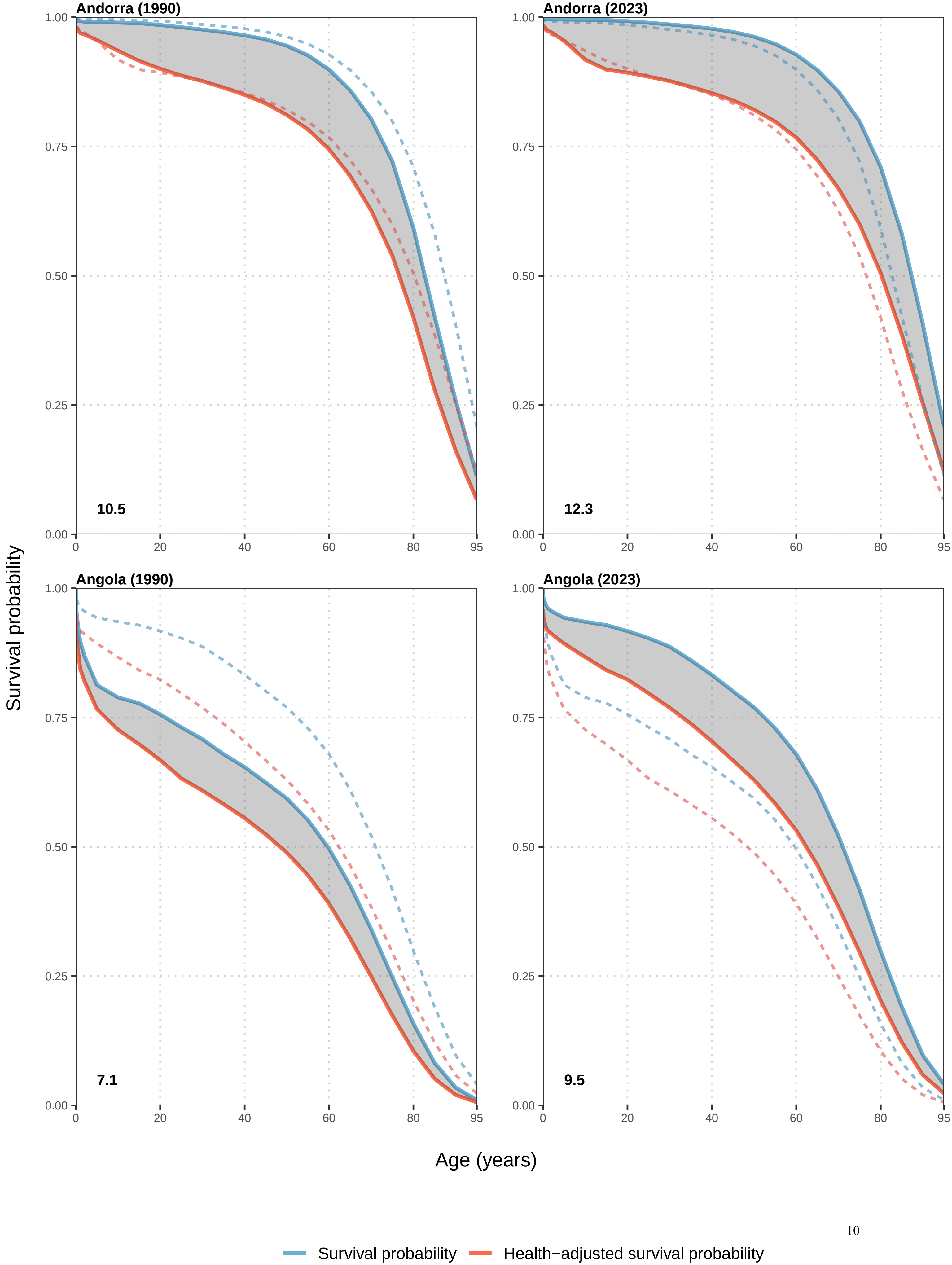
Andorra (1990)
Andorra (2023)
Angola (1990)
Angola (2023)
10.5
12.3
7.1
9.5
Survival probability
Age (years)
0.00
0.25
0.50
0.75
1.00
0
20
40
60
80
95
Survival probability
Health−adjusted survival probability

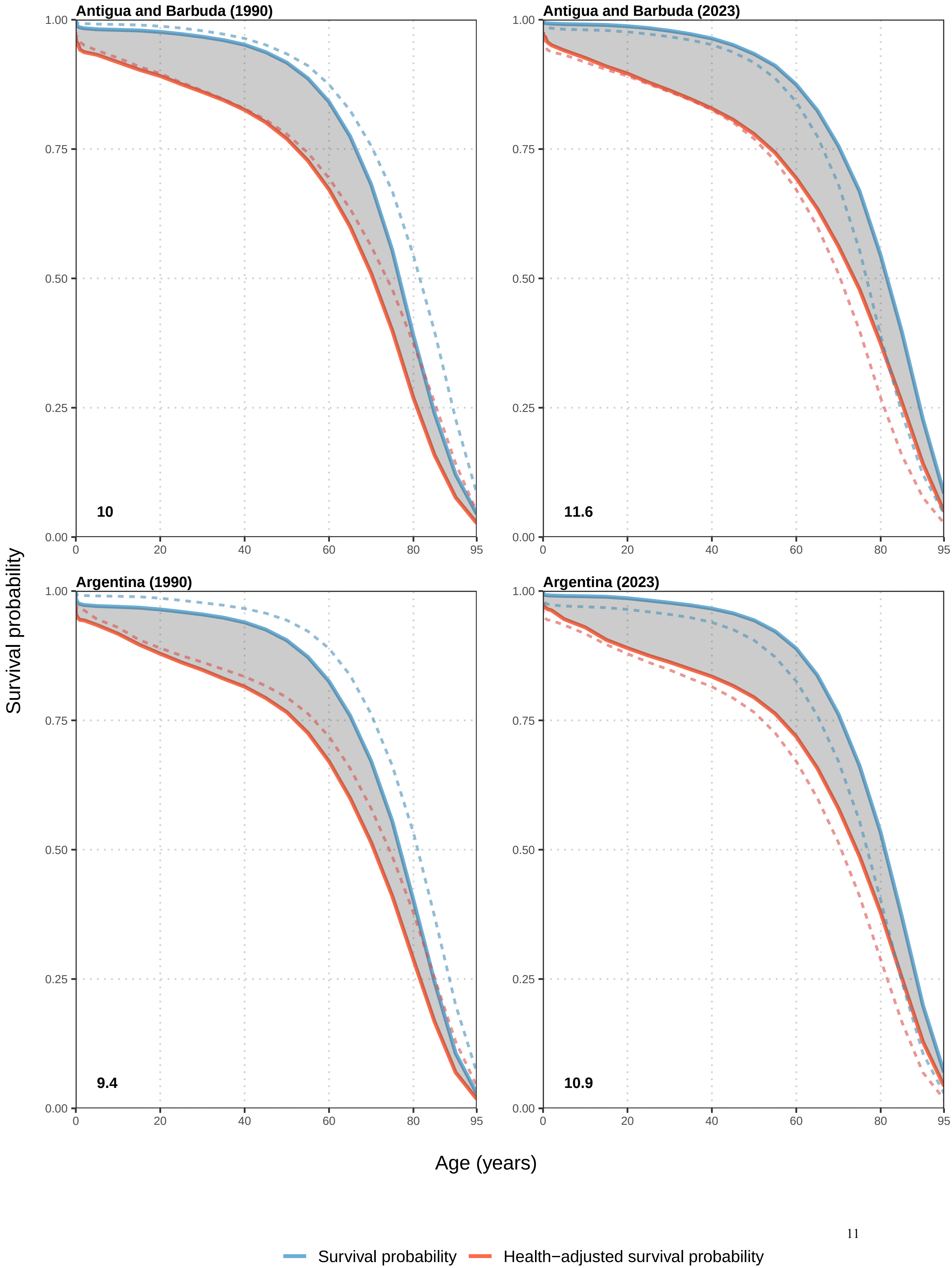

Antigua and Barbuda (1990)
Antigua and Barbuda (2023)
Argentina (1990)
Argentina (2023)
10
11.6
9.4
10.9
Survival probability
Age (years)
1.00
0.75
0.50
0.25
0.00
0
20
40
60
80
95
Survival probability
Health−adjusted survival probability

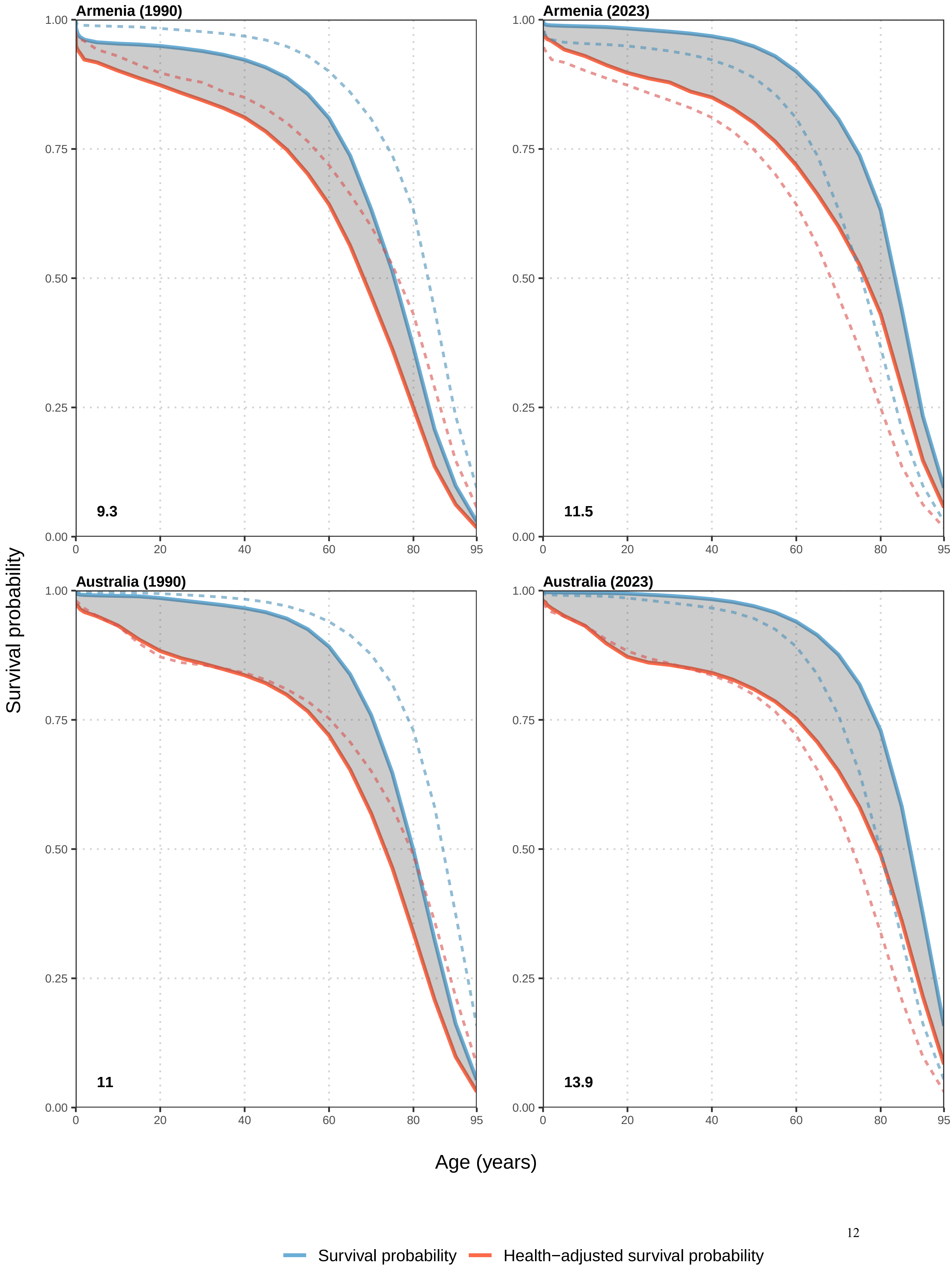

Armenia (1990)
Armenia (2023)
Australia (1990)
Australia (2023)
9.3
11.5
11
13.9
Survival probability
Age (years)
1.00
0.75
0.50
0.25
0.00
0
20
40
60
80
95
Survival probability
Health−adjusted survival probability

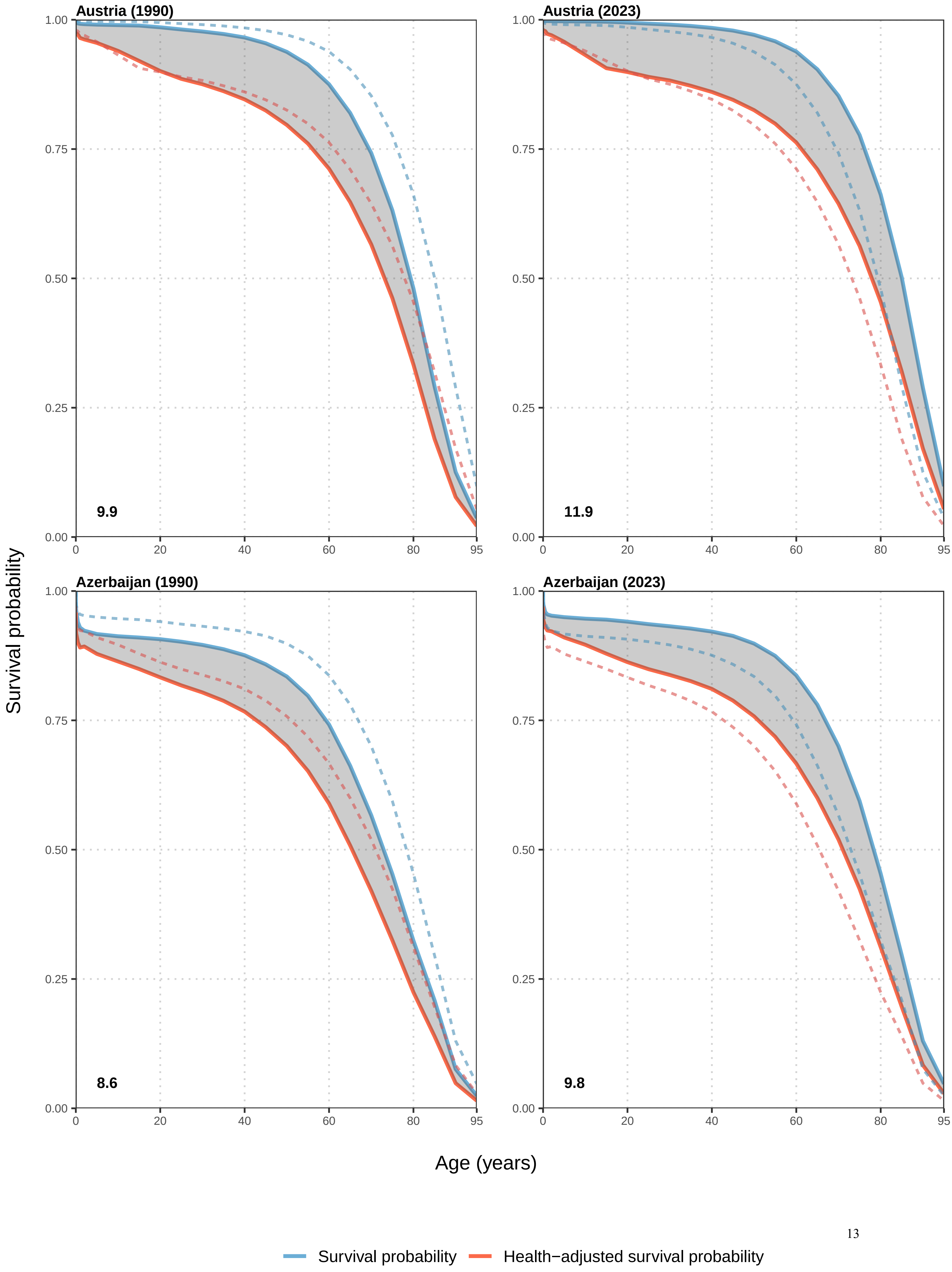
Austria (1990)
9.9
Austria (2023)
11.9
Azerbaijan (1990)
8.6
Azerbaijan (2023)
9.8
Survival probability
Age (years)
Survival probability
Health−adjusted survival probability

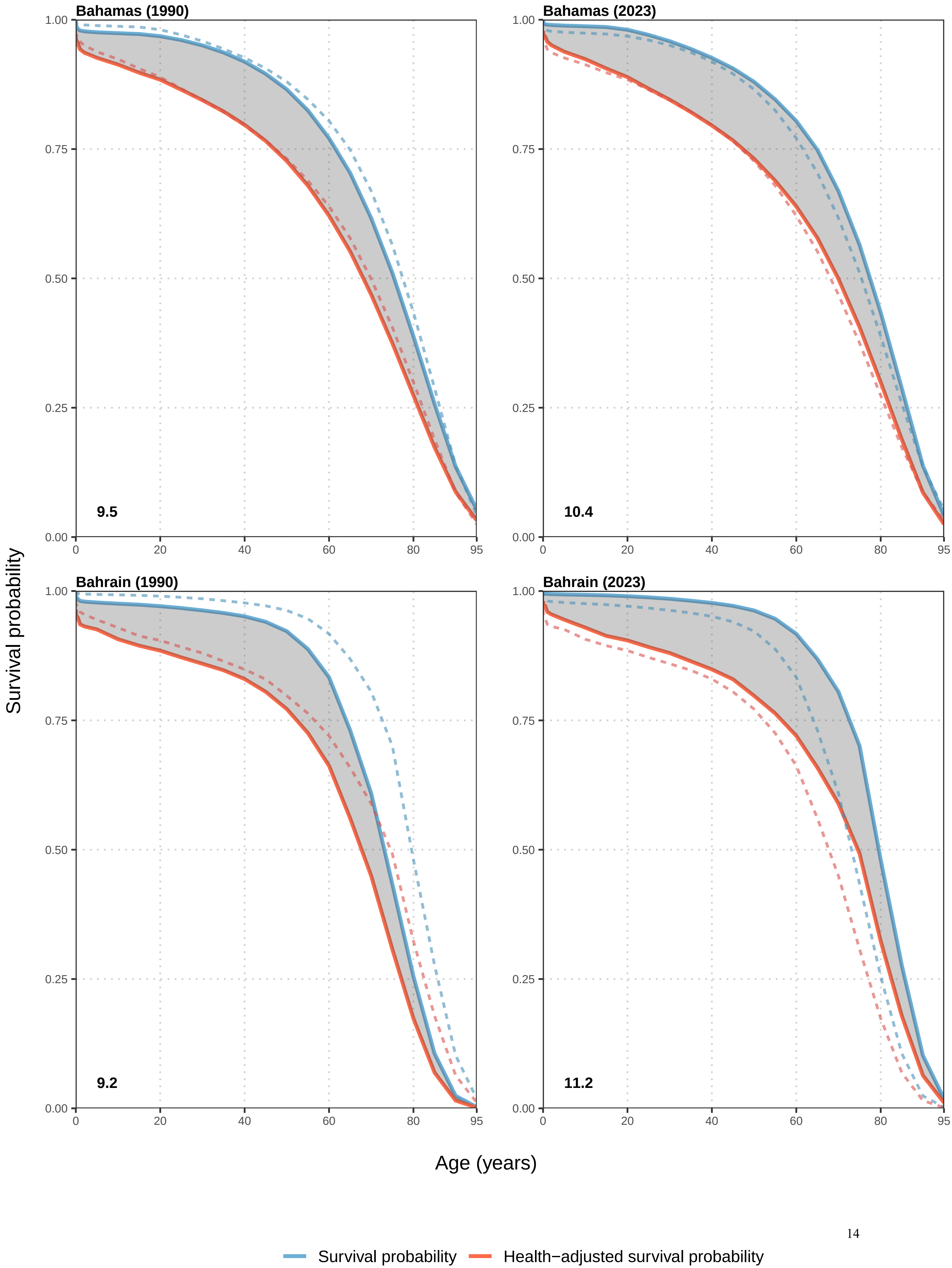

Bahamas (1990)
Bahamas (2023)
Bahrain (1990)
Bahrain (2023)
9.5
10.4
9.2
11.2
1.00
0.75
0.50
0.25
0.00
0
20
40
60
80
95
Survival probability
Age (years)
Survival probability
Health−adjusted survival probability

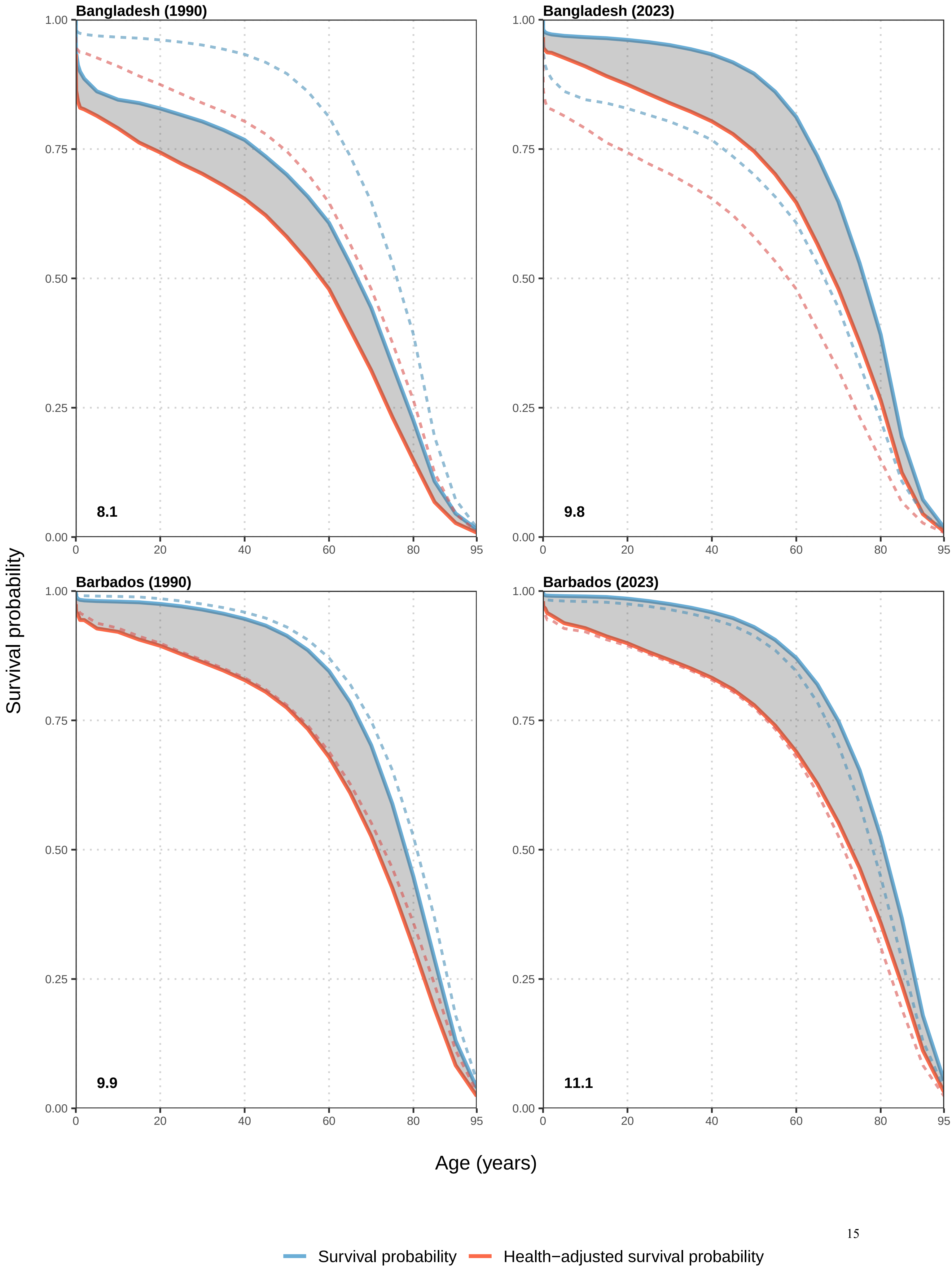
Bangladesh (1990)
Bangladesh (2023)
Barbados (1990)
Barbados (2023)
8.1
9.8
9.9
11.1
1.00
0.75
0.50
0.25
0.00
0
20
40
60
80
95
Survival probability
Age (years)
Survival probability
Health−adjusted survival probability

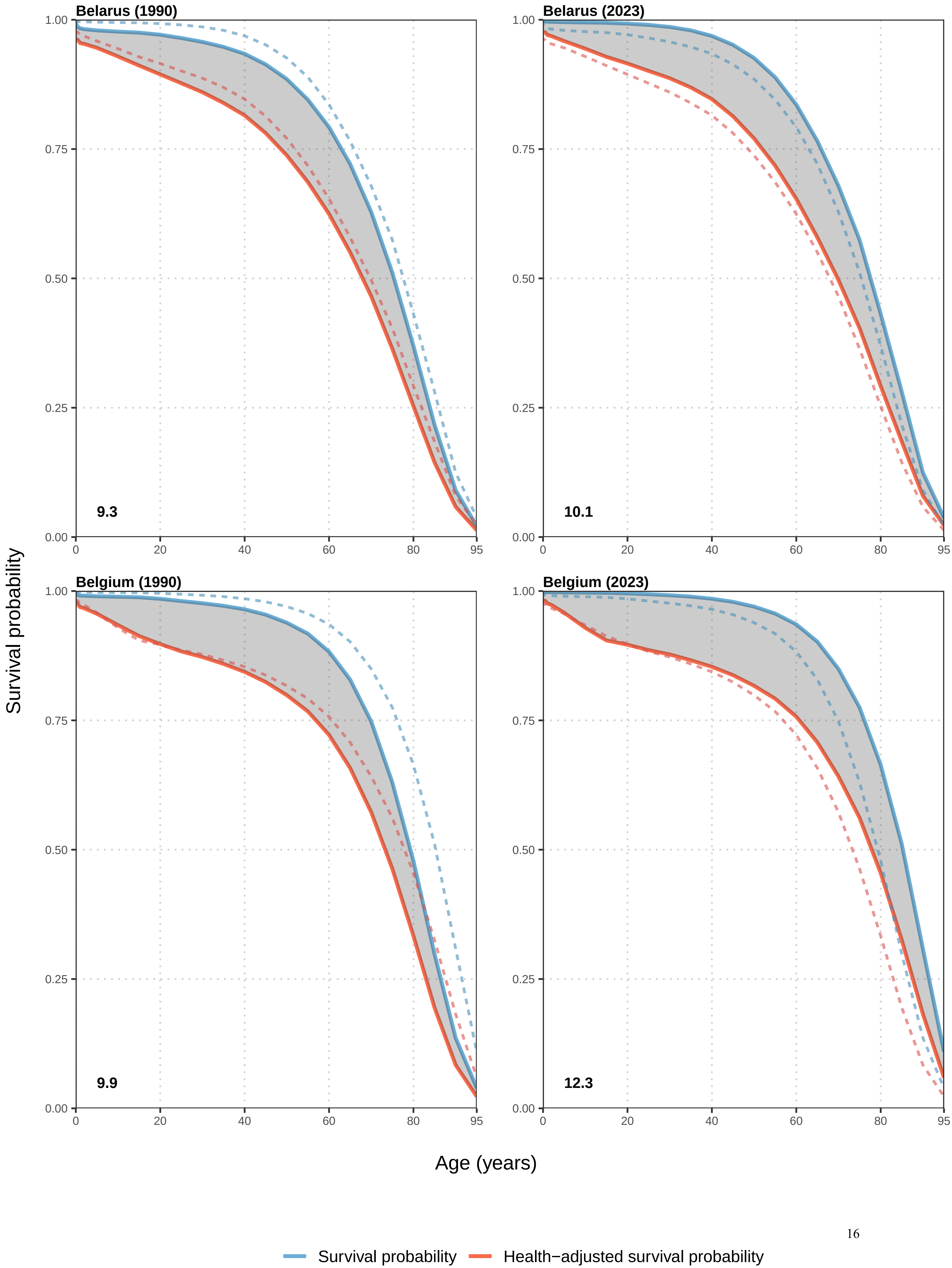
Belarus (1990)
Belarus (2023)
Belgium (1990)
Belgium (2023)
9.3
10.1
9.9
12.3
1.00
0.75
0.50
0.25
0.00
0
20
40
60
80
95
Survival probability
Age (years)
Survival probability
Health−adjusted survival probability

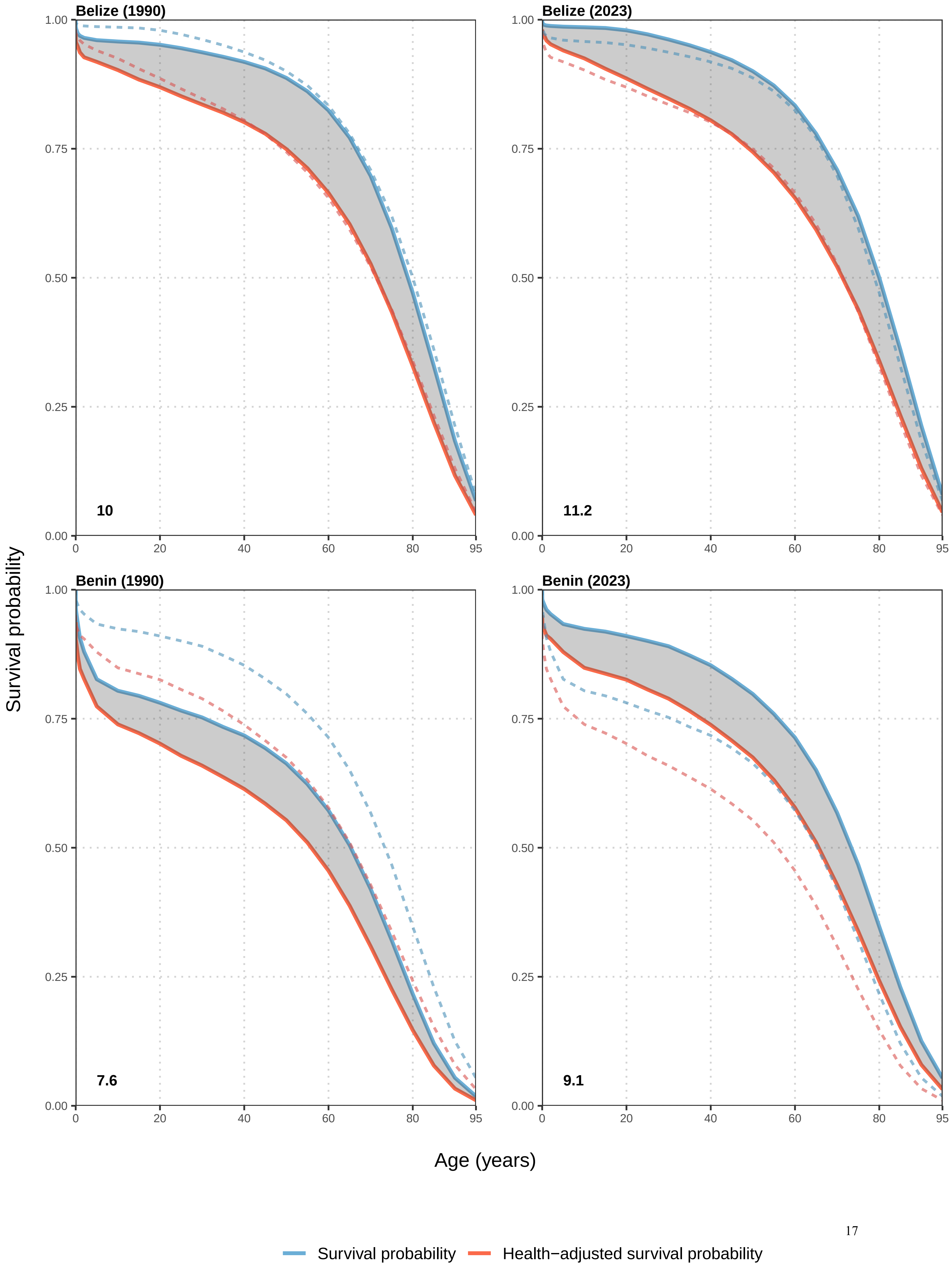

Belize (1990)
Belize (2023)
Benin (1990)
Benin (2023)
10
11.2
7.6
9.1
Survival probability
Age (years)
0.00
0.25
0.50
0.75
1.00
0
20
40
60
80
95
Survival probability
Health−adjusted survival probability

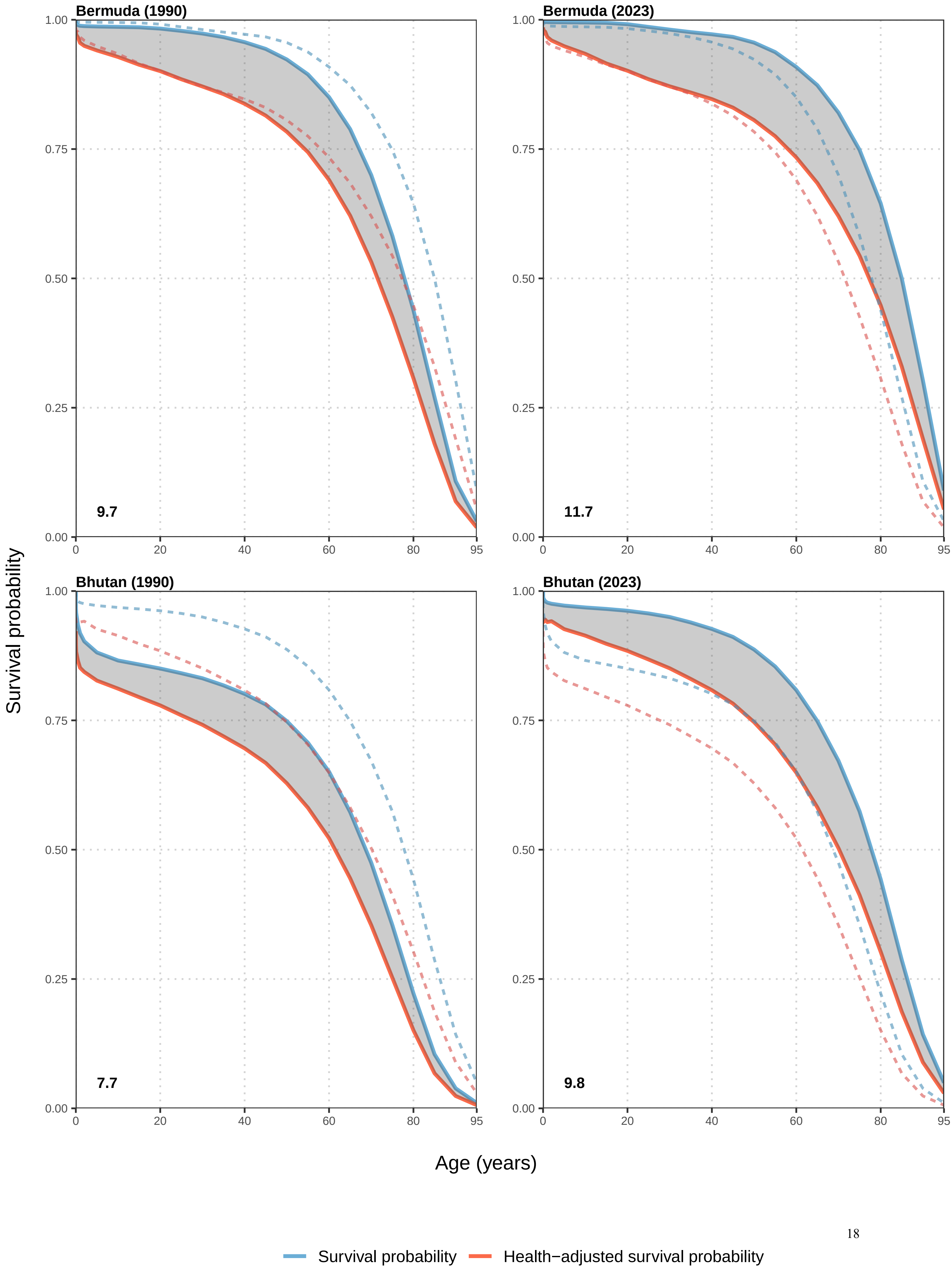

Bermuda (1990)
Bermuda (2023)
Bhutan (1990)
Bhutan (2023)
9.7
11.7
7.7
9.8
Survival probability
Age (years)
1.00
0.75
0.50
0.25
0.00
0
20
40
60
80
95
Survival probability
Health−adjusted survival probability

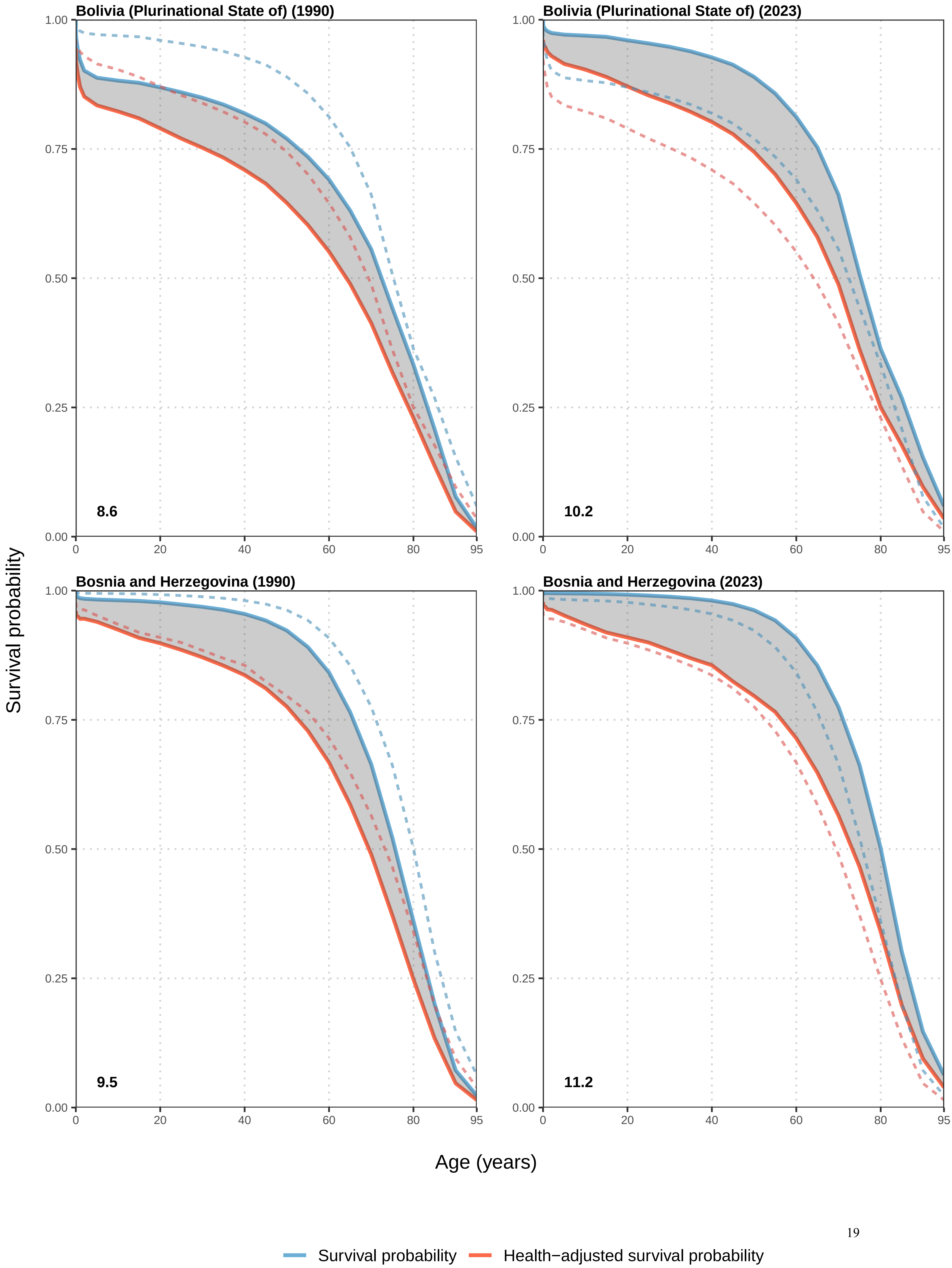
Bolivia (Plurinational State of) (1990)
8.6
Bolivia (Plurinational State of) (2023)
10.2
Bosnia and Herzegovina (1990)
9.5
Bosnia and Herzegovina (2023)
11.2
Survival probability
Age (years)
1.00
0.75
0.50
0.25
0.00
0
20
40
60
80
95
Survival probability
Health−adjusted survival probability

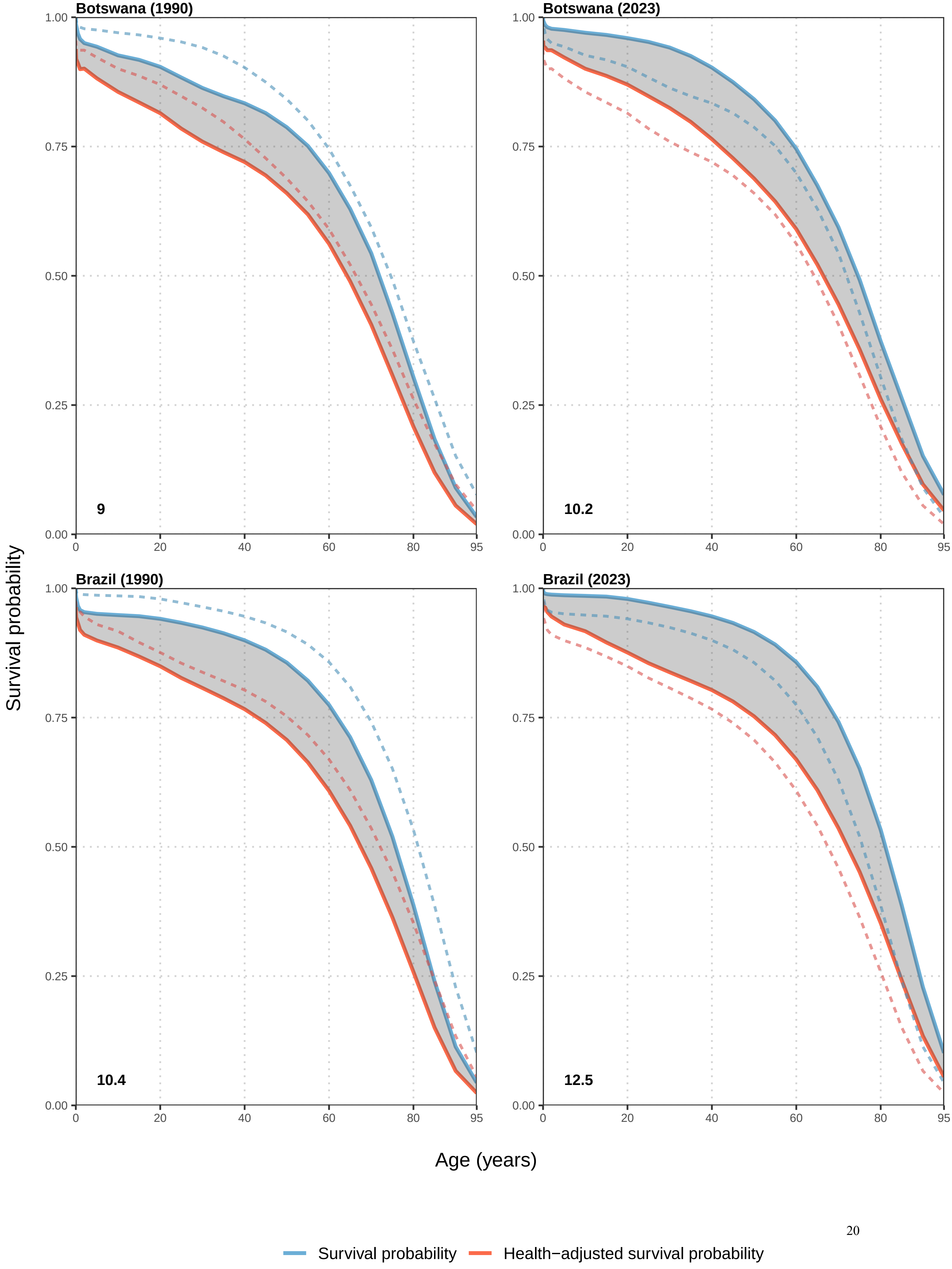
Botswana (1990)
9
Botswana (2023)
10.2
Brazil (1990)
10.4
Brazil (2023)
12.5
1.00
0.75
0.50
0.25
0.00
0
20
40
60
80
95
Survival probability
Age (years)
Survival probability
Health−adjusted survival probability

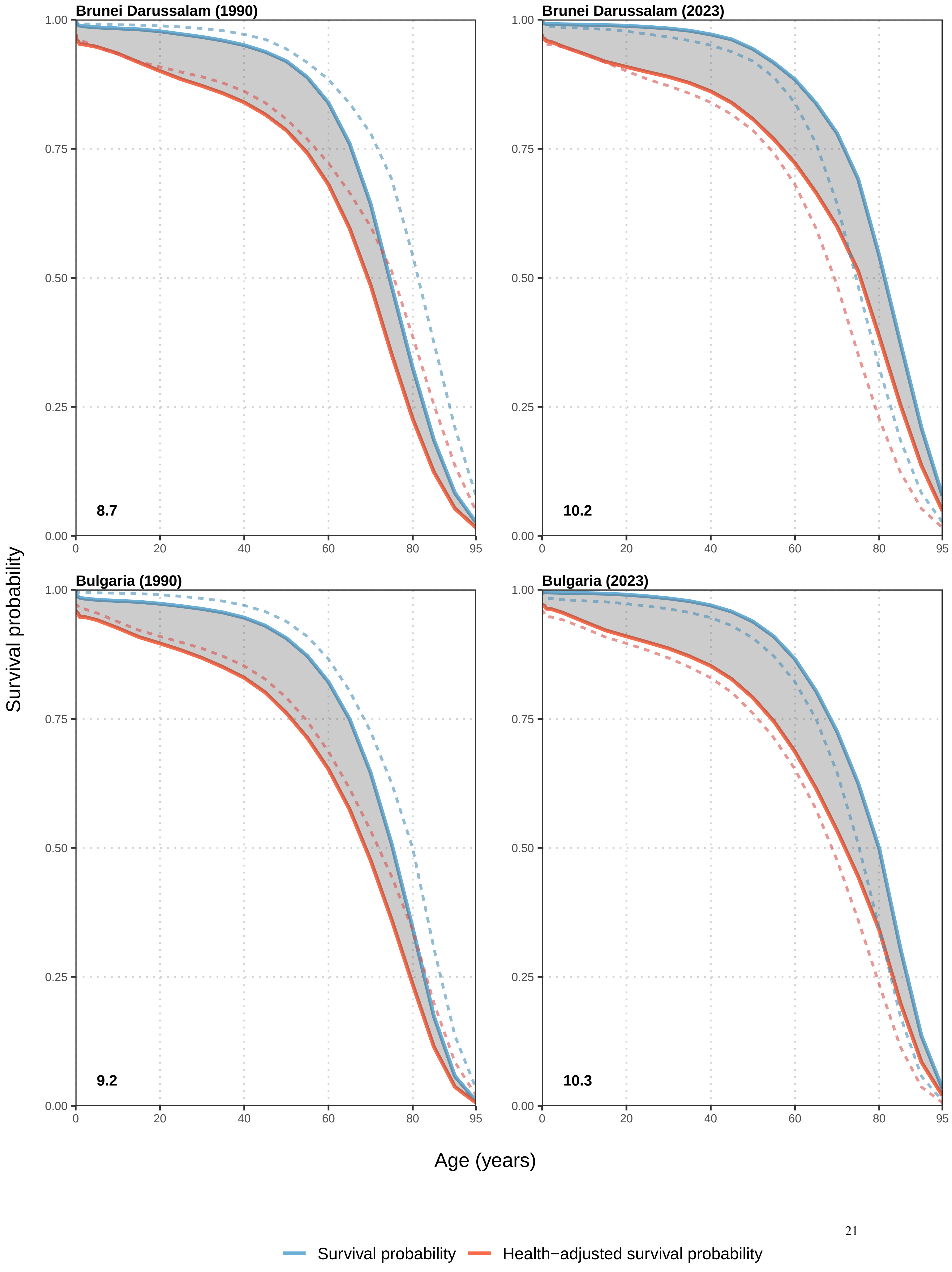
Brunei Darussalam (1990)
Brunei Darussalam (2023)
Bulgaria (1990)
Bulgaria (2023)
8.7
10.2
9.2
10.3
1.00
0.75
0.50
0.25
0.00
0
20
40
60
80
95
Survival probability
Age (years)
Survival probability
Health−adjusted survival probability

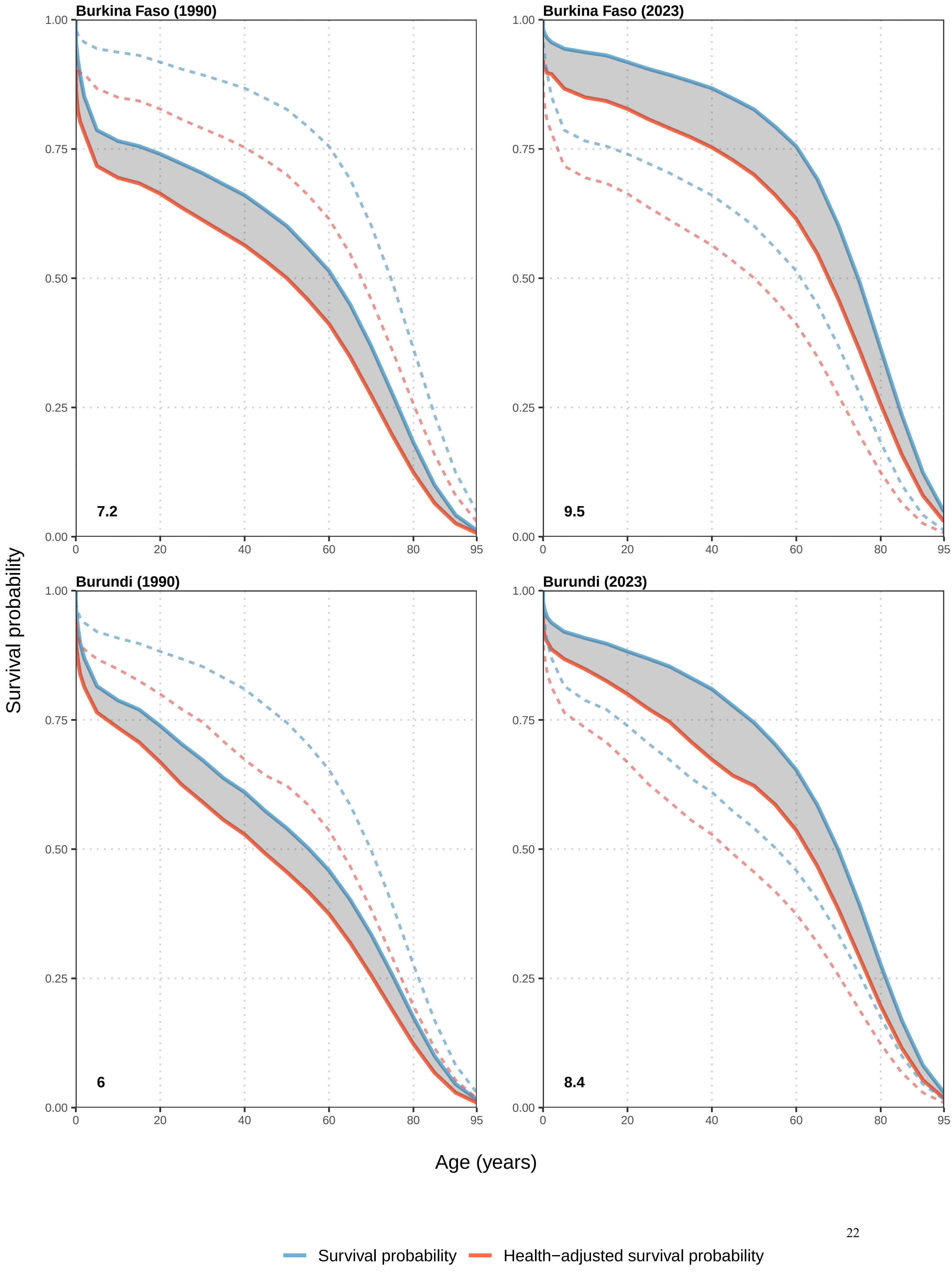
Burkina Faso (1990)
Burkina Faso (2023)
Burundi (1990)
Burundi (2023)
7.2
9.5
6
8.4
1.00
0.75
0.50
0.25
0.00
0
20
40
60
80
95
Survival probability
Age (years)
Survival probability
Health−adjusted survival probability

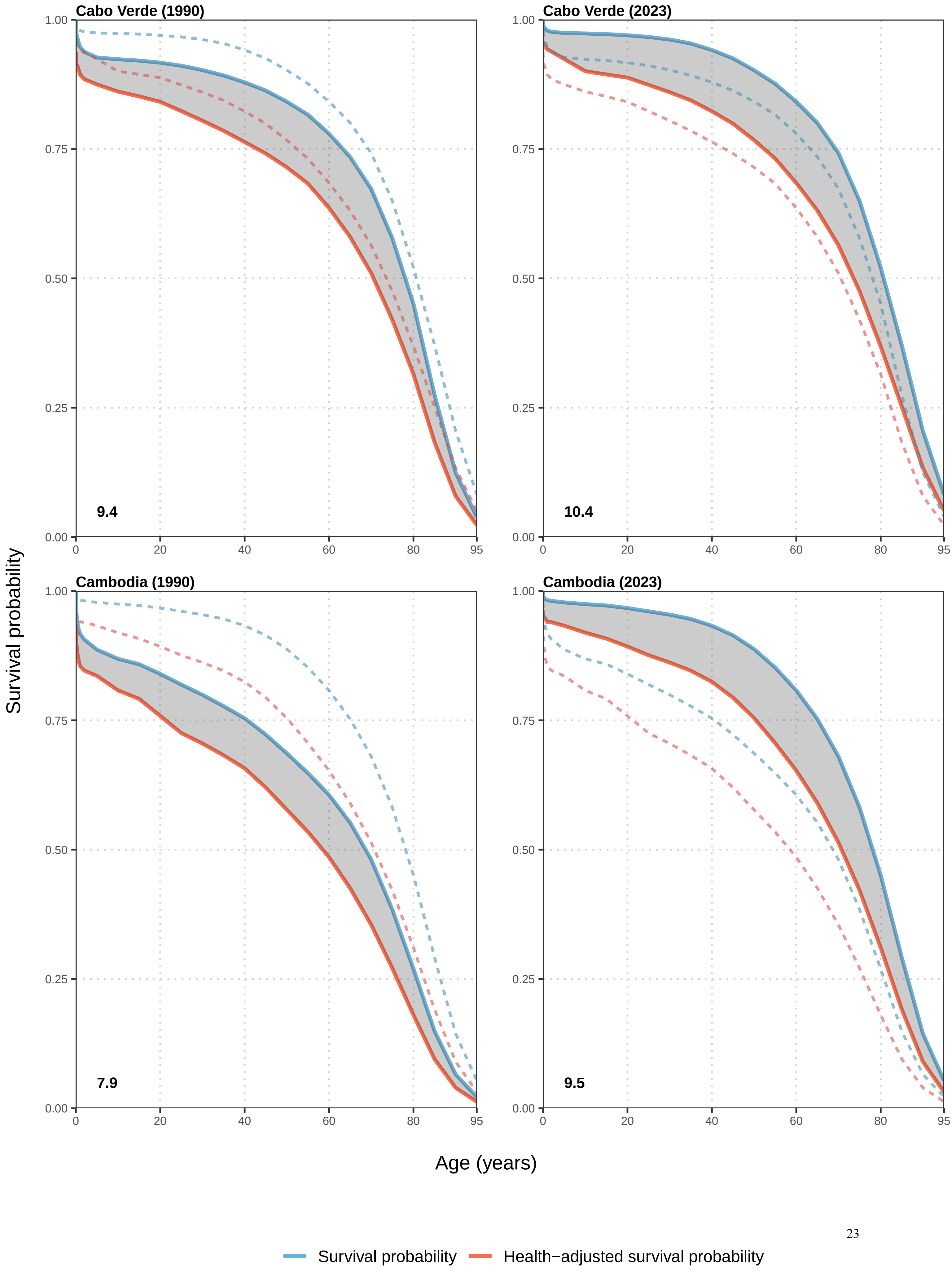
Cabo Verde (1990)
Cabo Verde (2023)
Cambodia (1990)
Cambodia (2023)
9.4
10.4
7.9
9.5
Survival probability
Age (years)
1.00
0.75
0.50
0.25
0.00
0
20
40
60
80
95
Survival probability
Health−adjusted survival probability

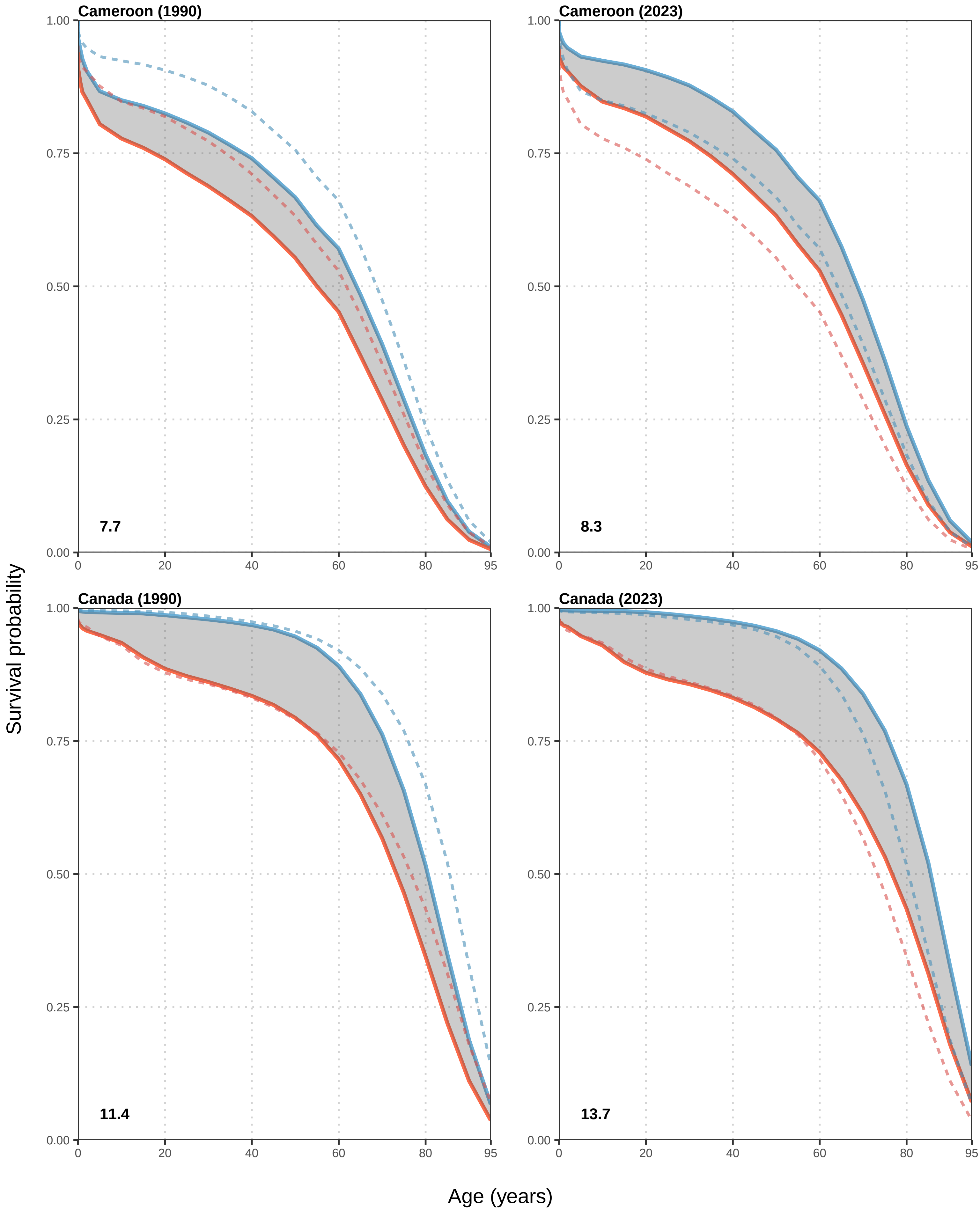

Cameroon (1990)
Cameroon (2023)
Canada (1990)
Canada (2023)
7.7
8.3
11.4
13.7
1.00
0.75
0.50
0.25
0.00
0
20
40
60
80
95
Survival probability
Age (years)

Survival probability
Health−adjusted survival probability

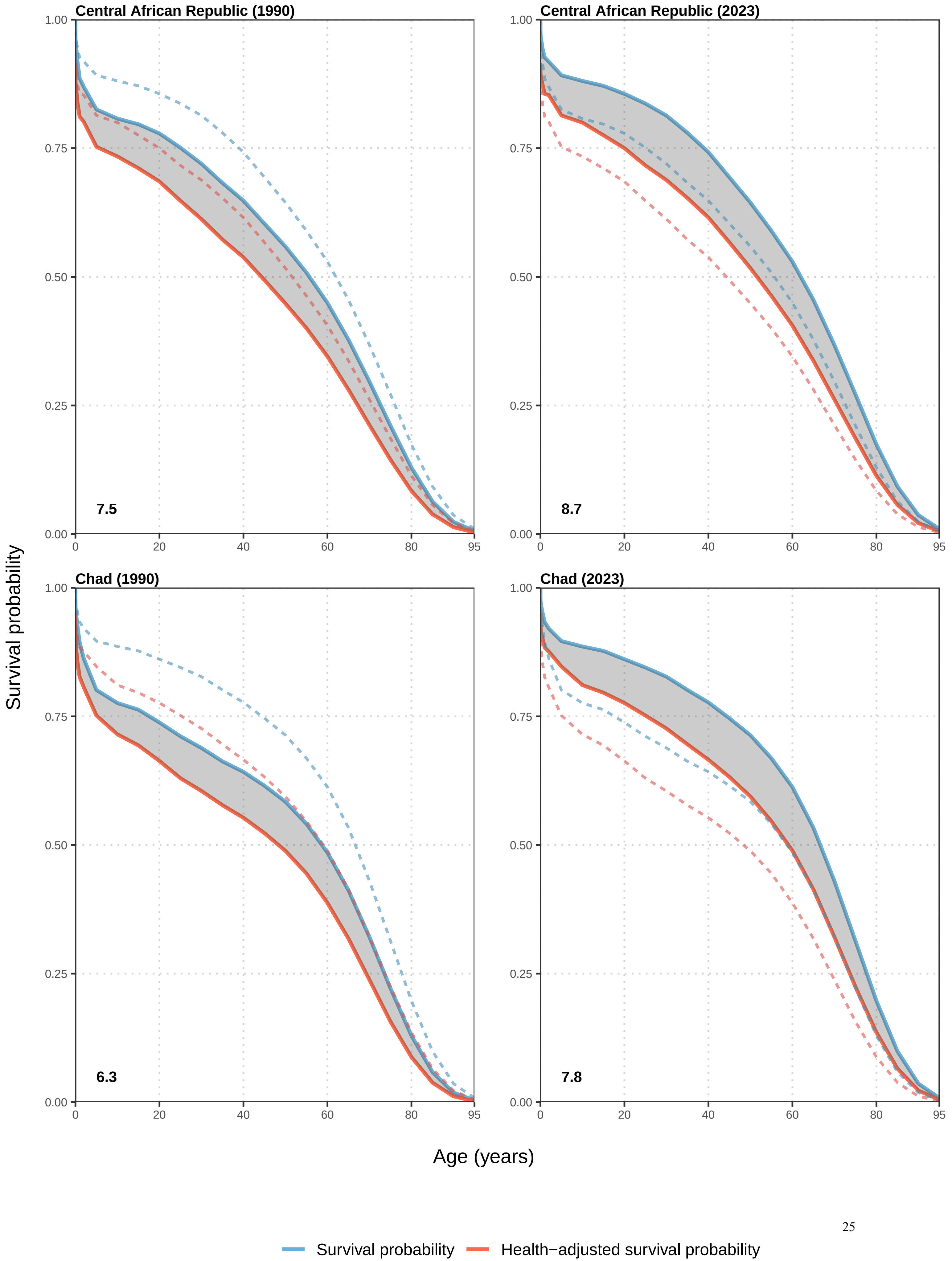

Central African Republic (1990)
Central African Republic (2023)
Chad (1990)
Chad (2023)
7.5
8.7
6.3
7.8
Survival probability
Age (years)
Survival probability
Health−adjusted survival probability

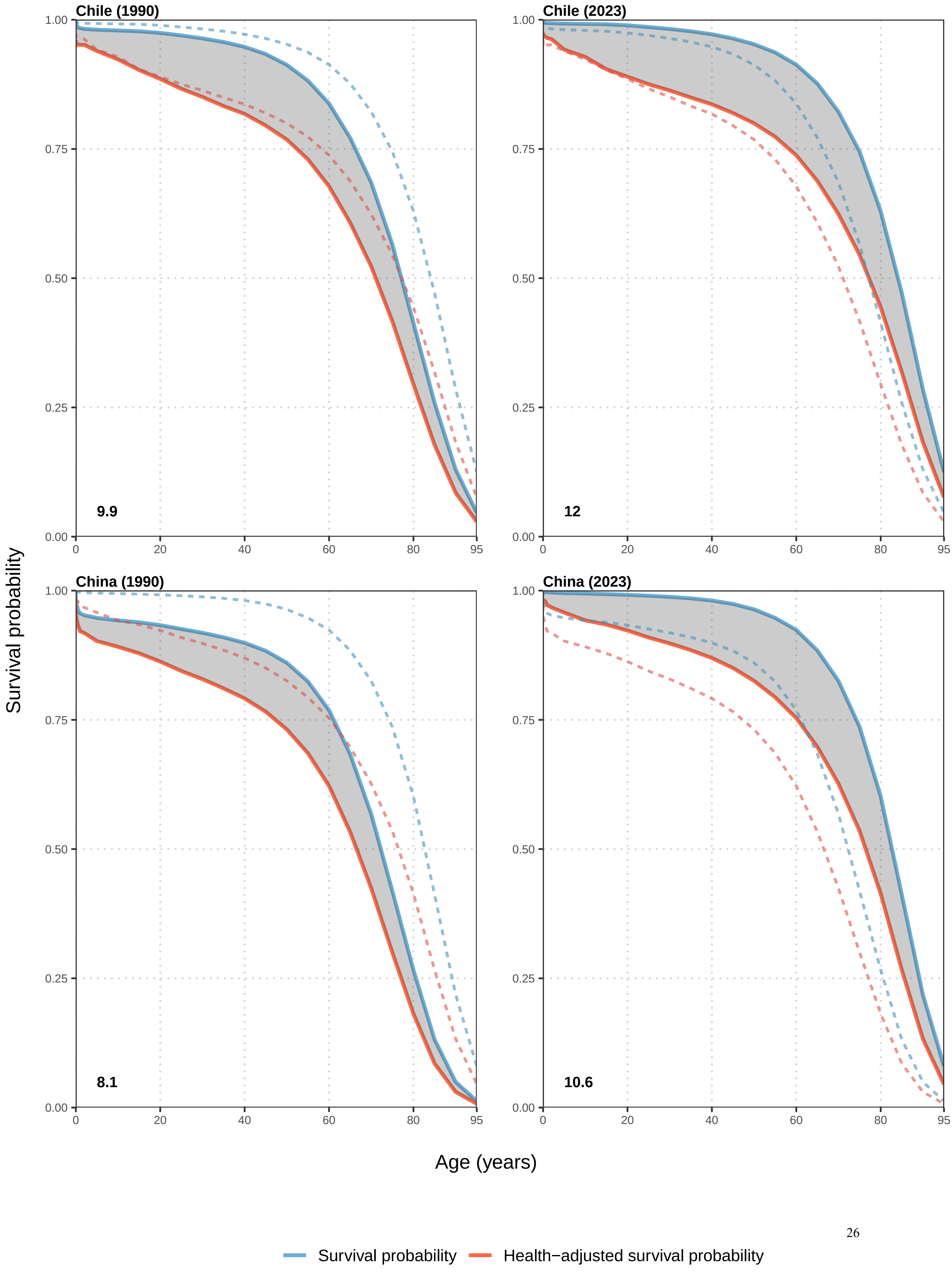

Chile (1990)
9.9
Chile (2023)
12
China (1990)
8.1
China (2023)
10.6
Survival probability
Age (years)
1.00
0.75
0.50
0.25
0.00
0
20
40
60
80
95
Survival probability
Health−adjusted survival probability

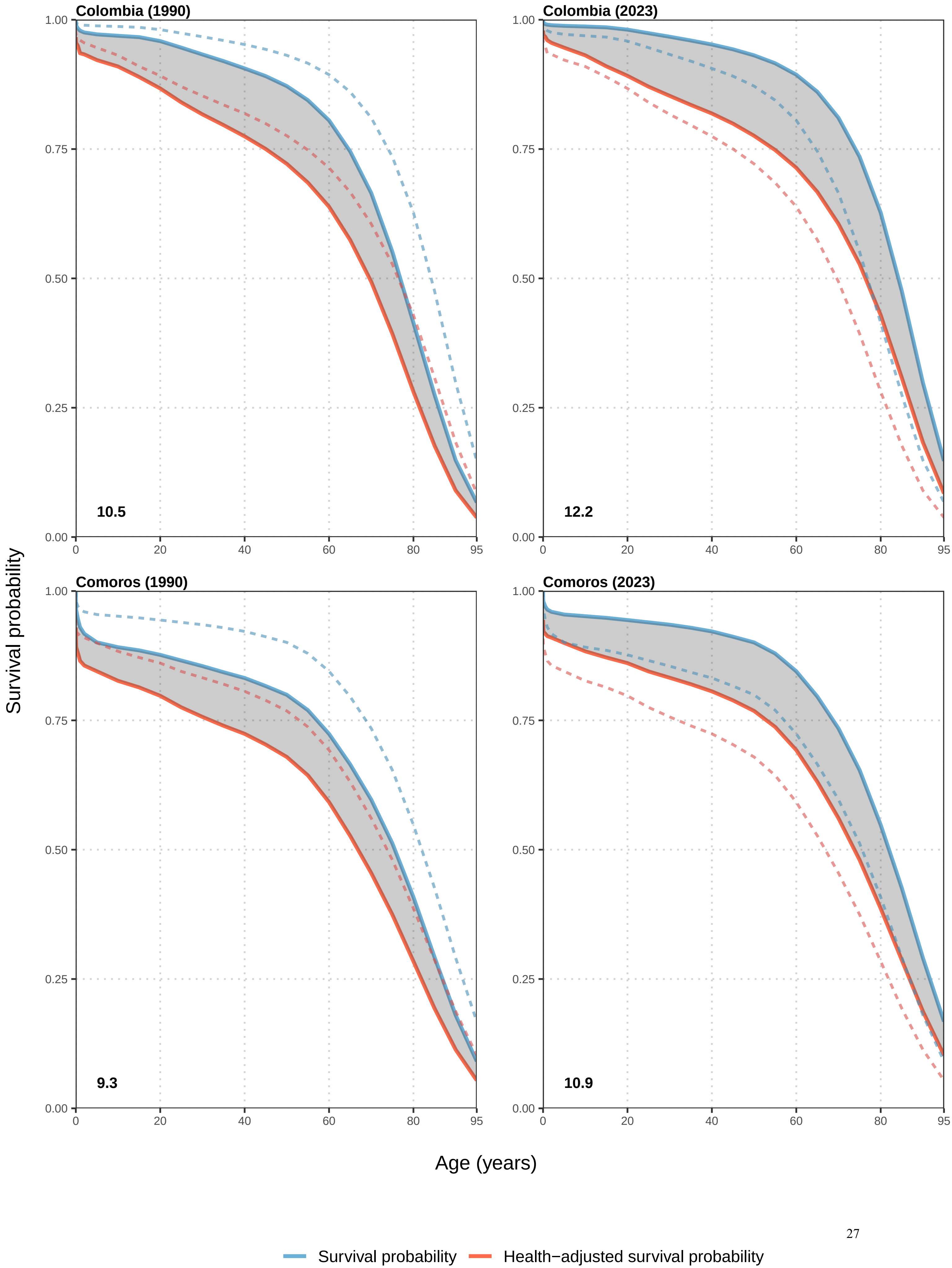

Colombia (1990)
Colombia (2023)
Comoros (1990)
Comoros (2023)
10.5
12.2
9.3
10.9
1.00
0.75
0.50
0.25
0.00
0
20
40
60
80
95
Survival probability
Age (years)
Survival probability
Health−adjusted survival probability

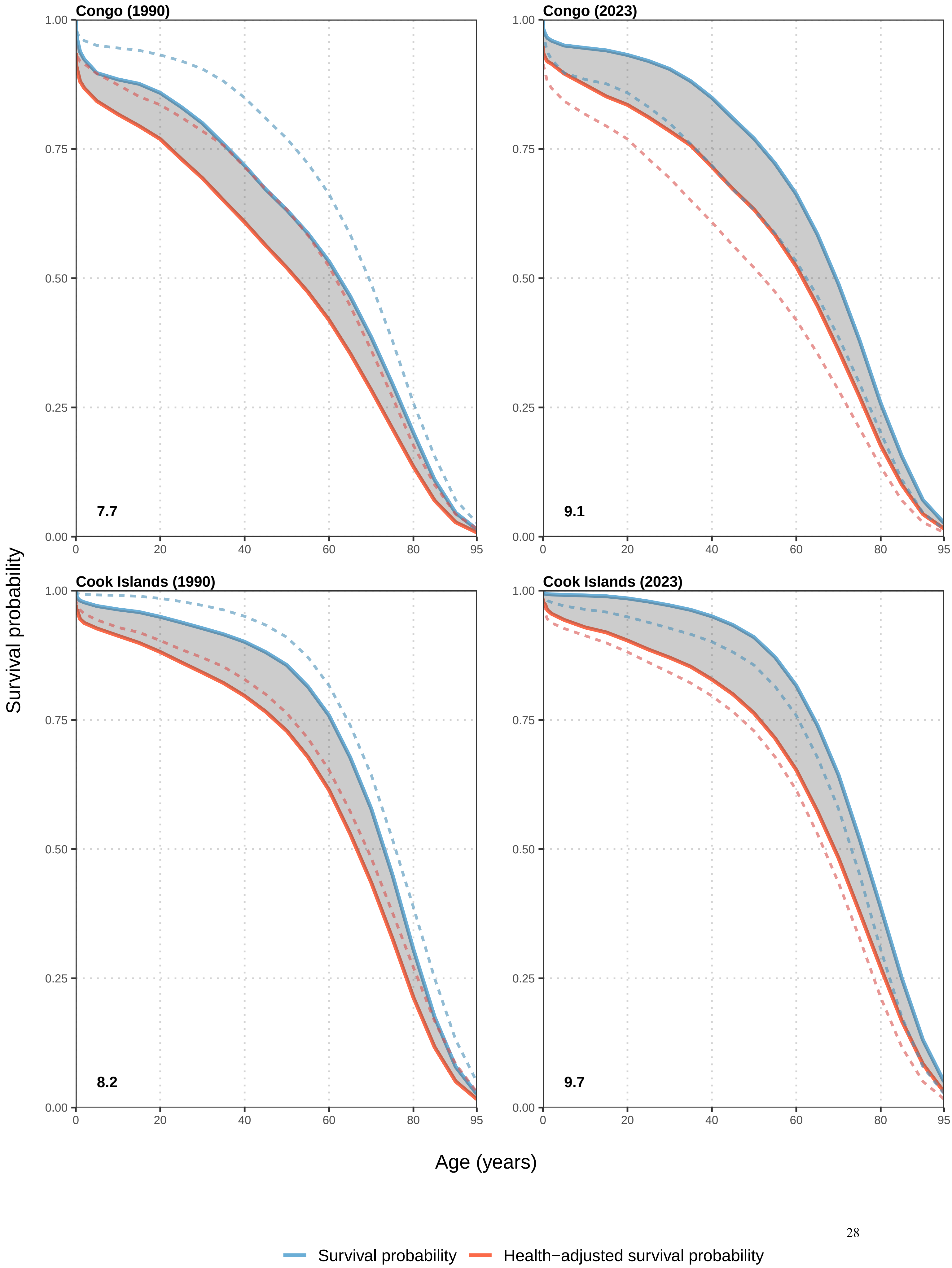

Congo (1990)
Congo (2023)
Cook Islands (1990)
Cook Islands (2023)
7.7
9.1
8.2
9.7
Survival probability
Age (years)
1.00
0.75
0.50
0.25
0.00
0
20
40
60
80
95
Survival probability
Health−adjusted survival probability

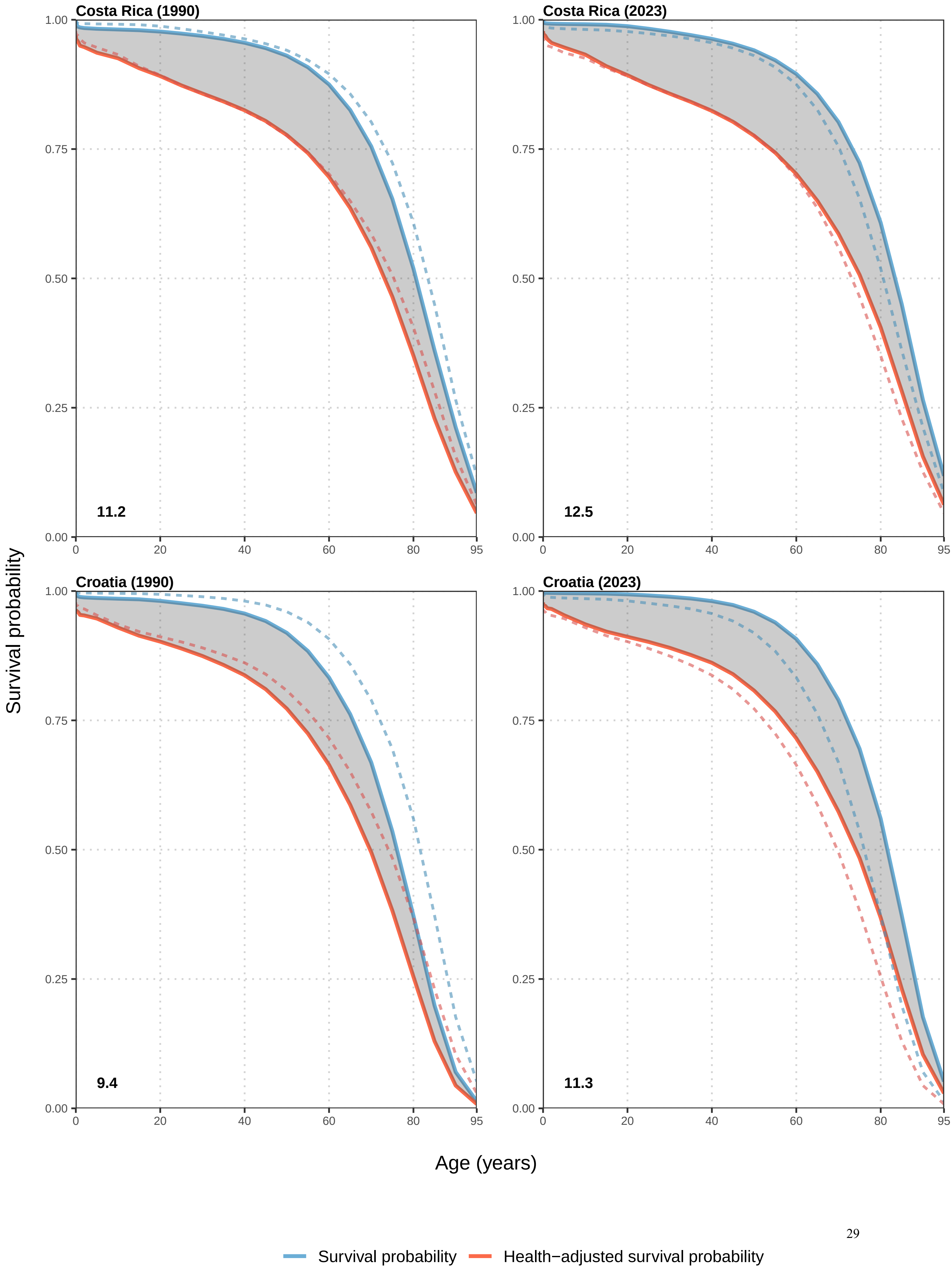
Costa Rica (1990)
Costa Rica (2023)
Croatia (1990)
Croatia (2023)
11.2
12.5
9.4
11.3
1.00
0.75
0.50
0.25
0.00
0
20
40
60
80
95
Survival probability
Age (years)
Survival probability
Health−adjusted survival probability

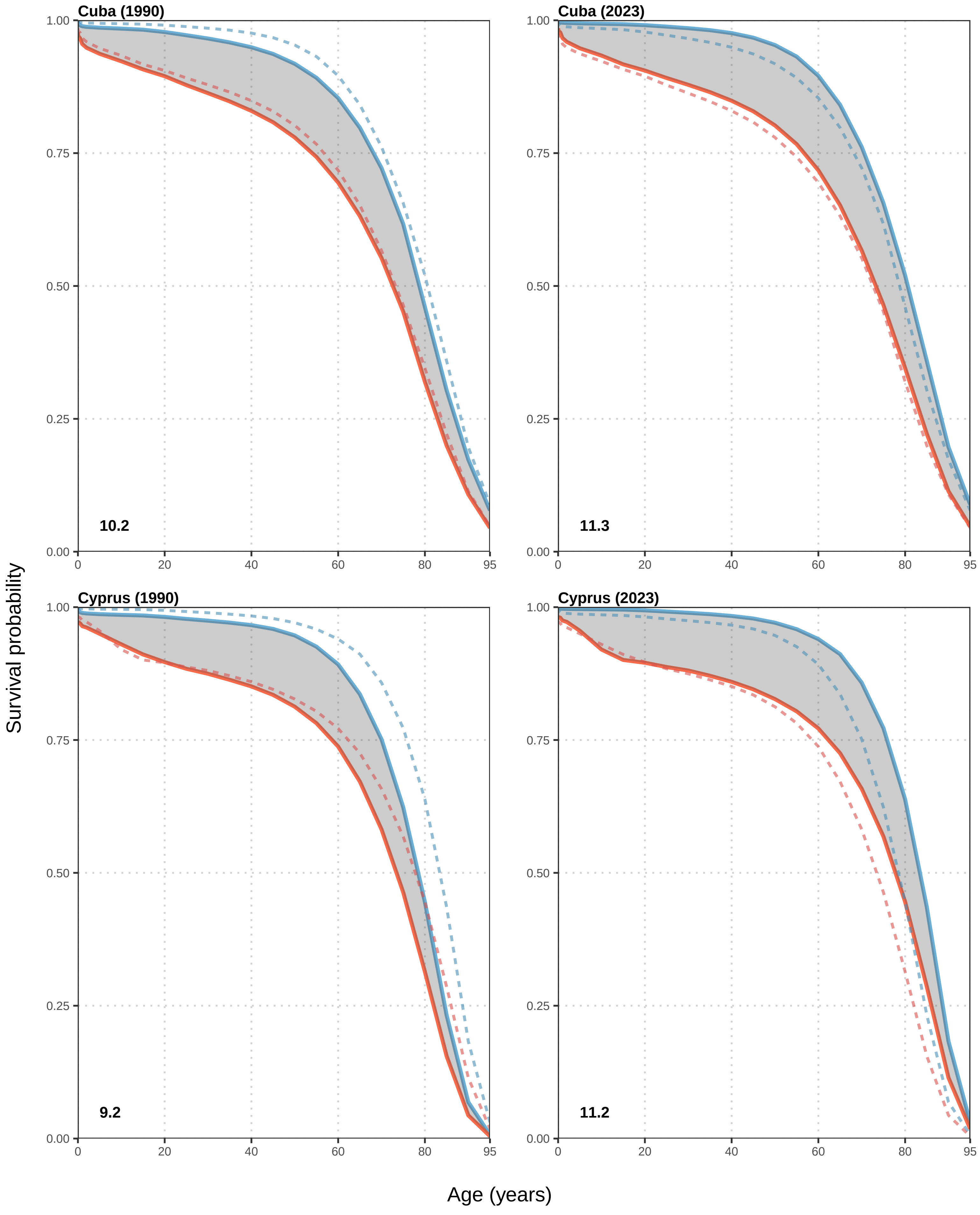

Cuba (1990)
Cuba (2023)
Cyprus (1990)
Cyprus (2023)
10.2
11.3
9.2
11.2
Survival probability
Age (years)
0.00
0.25
0.50
0.75
1.00
0
20
40
60
80
95

Survival probability
Health−adjusted survival probability

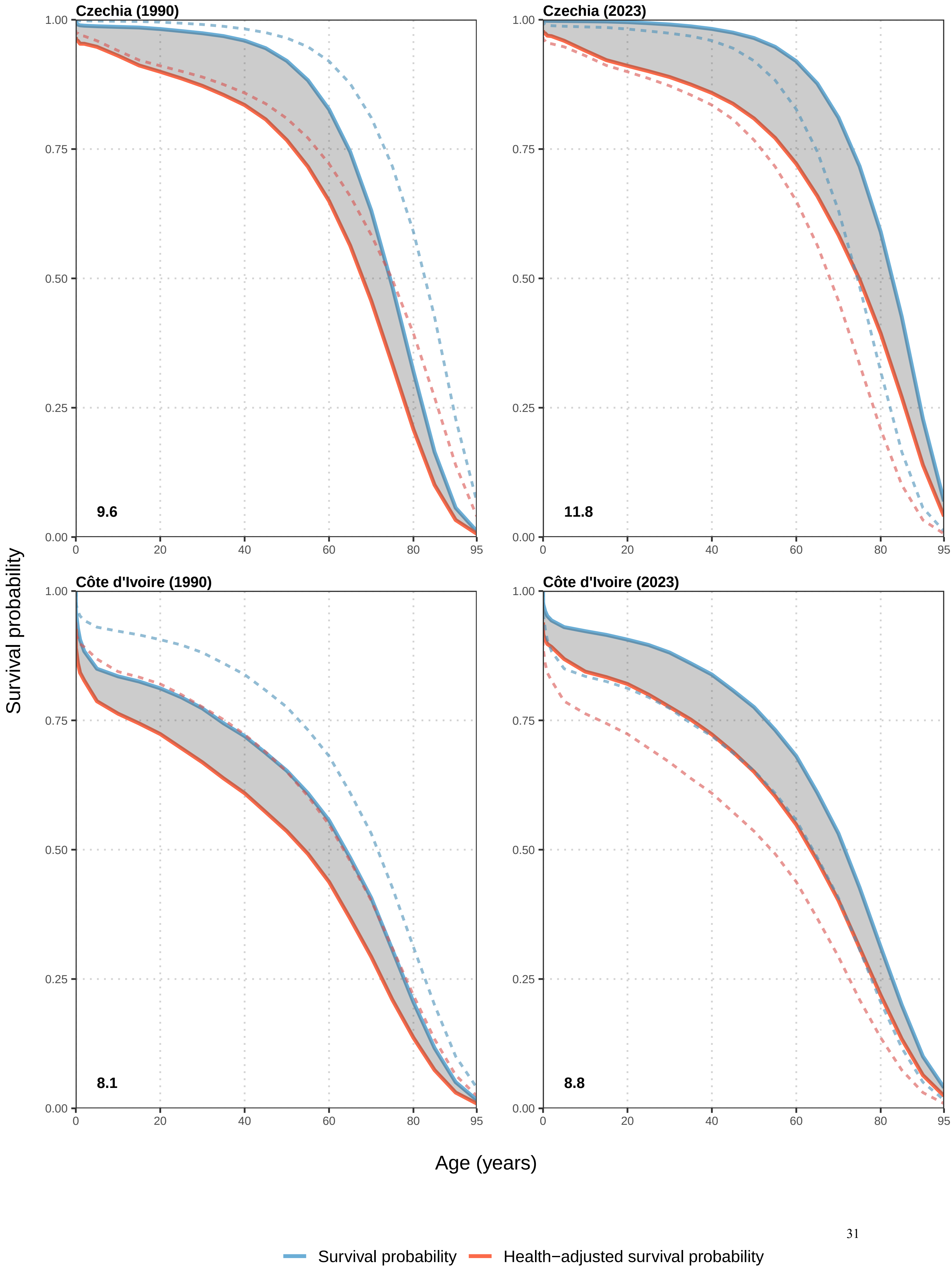
Czechia (1990)
Czechia (2023)
Côte d'Ivoire (1990)
Côte d'Ivoire (2023)
9.6
11.8
8.1
8.8
1.00
0.75
0.50
0.25
0.00
0
20
40
60
80
95
Survival probability
Age (years)

Survival probability
Health−adjusted survival probability

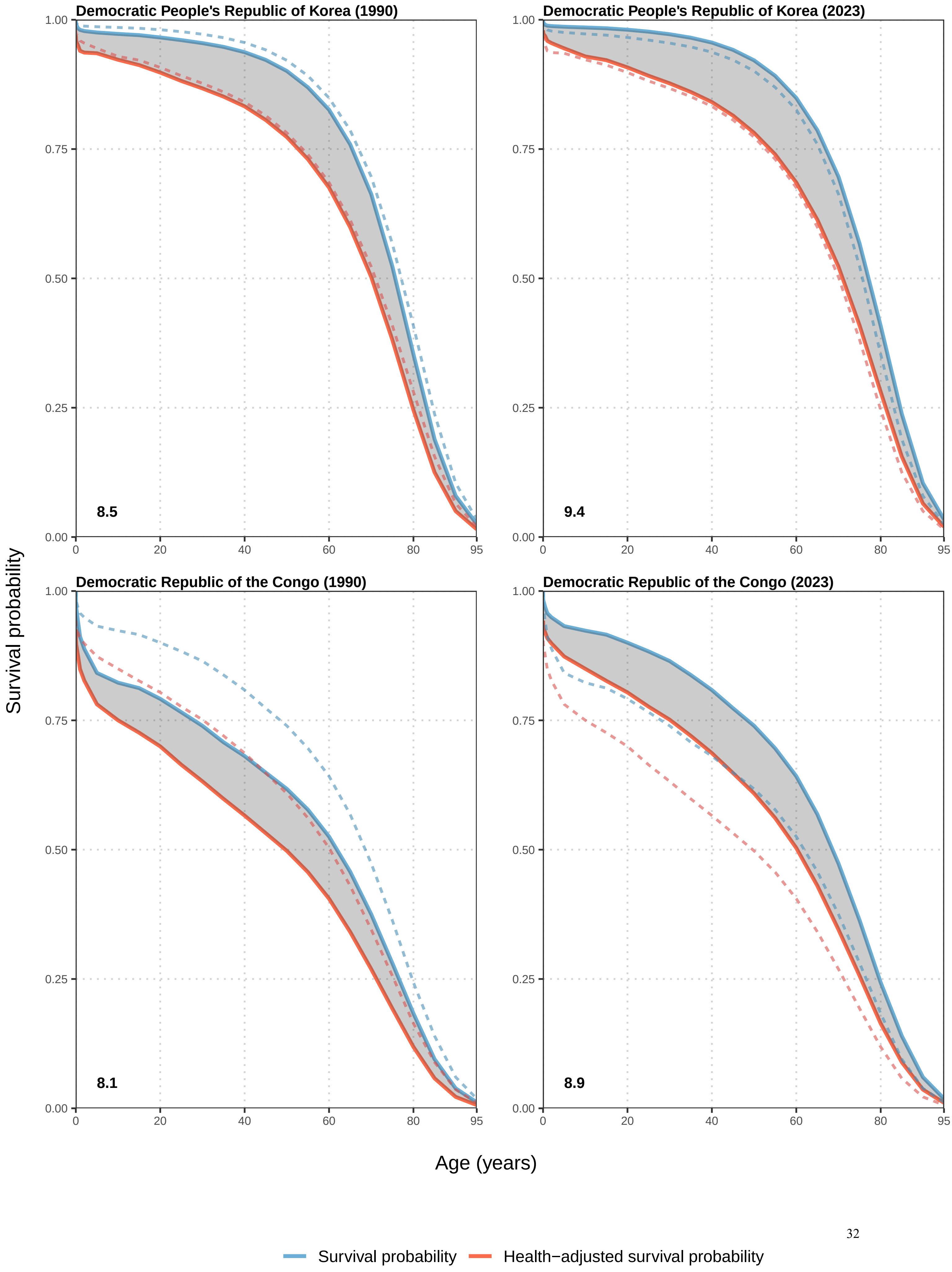

Democratic People's Republic of Korea (1990)
Democratic People's Republic of Korea (2023)
Democratic Republic of the Congo (1990)
Democratic Republic of the Congo (2023)
8.5
9.4
8.1
8.9
1.00
0.75
0.50
0.25
0.00
0
20
40
60
80
95
Survival probability
Age (years)
Survival probability
Health−adjusted survival probability

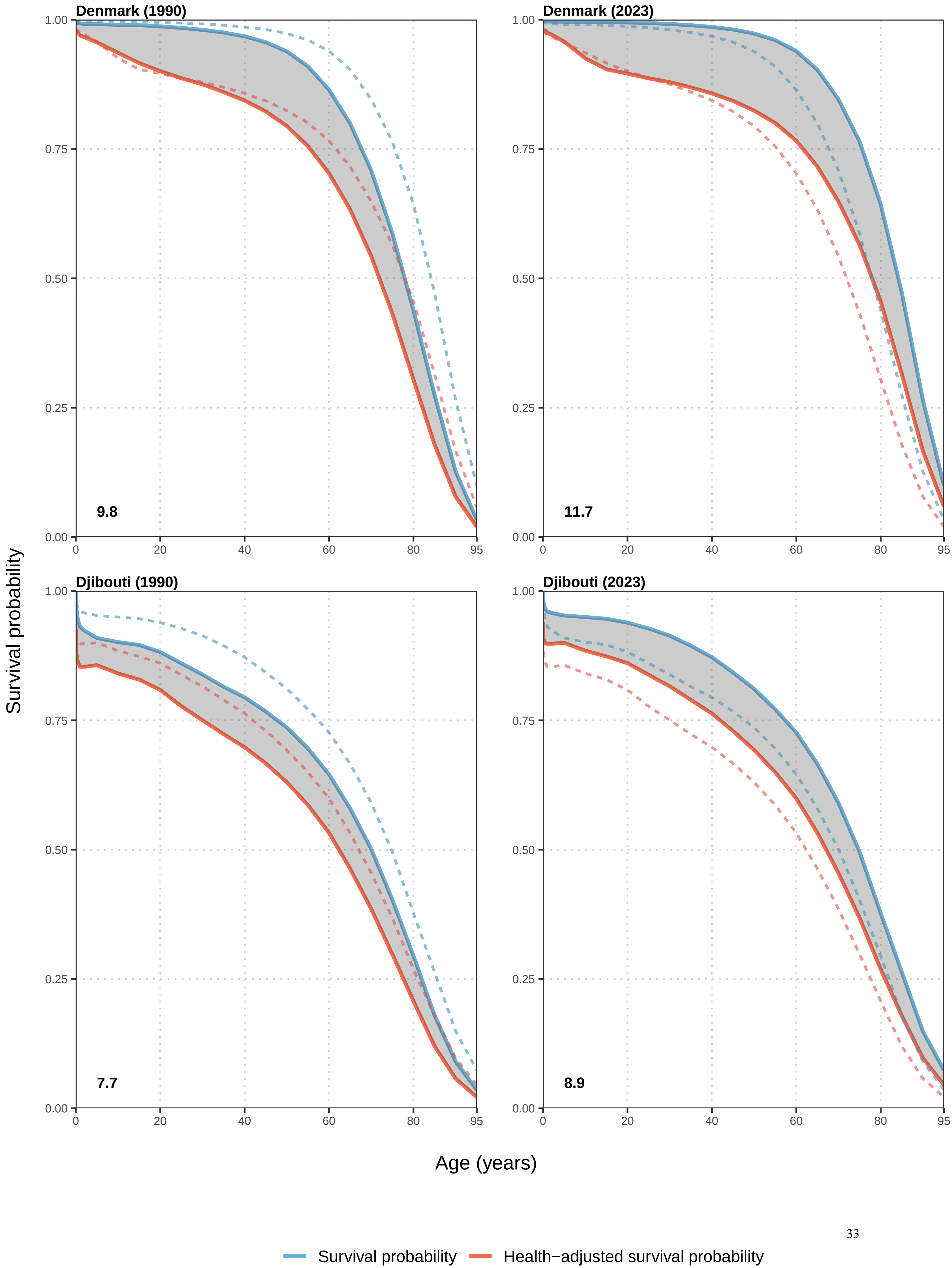
Denmark (1990)
Denmark (2023)
Djibouti (1990)
Djibouti (2023)
9.8
11.7
7.7
8.9
1.00
0.75
0.50
0.25
0.00
0
20
40
60
80
95
Survival probability
Age (years)
Survival probability
Health−adjusted survival probability

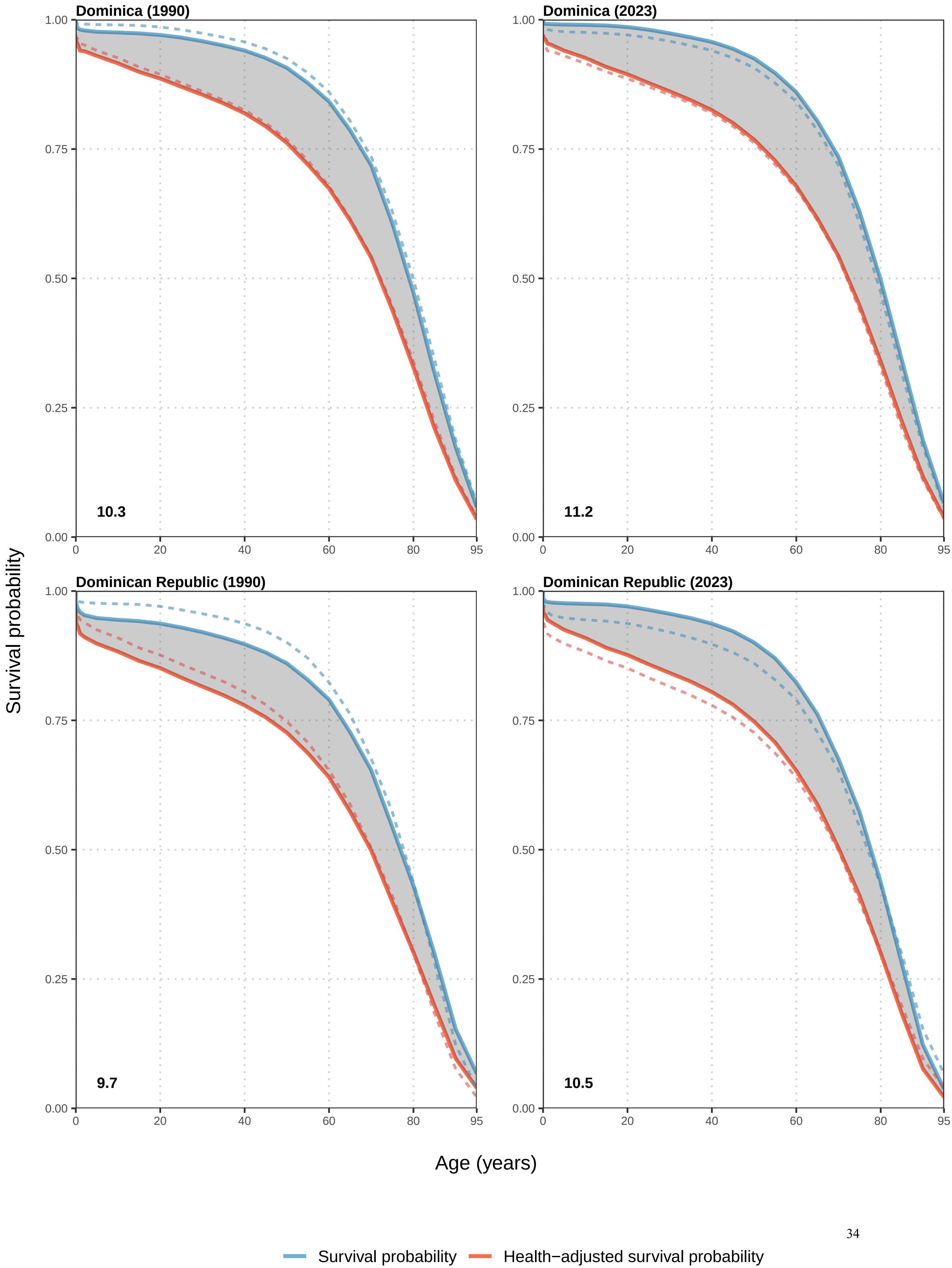

Dominica (1990)
Dominica (2023)
Dominican Republic (1990)
Dominican Republic (2023)
10.3
11.2
9.7
10.5
Survival probability
Age (years)
1.00
0.75
0.50
0.25
0.00
0
20
40
60
80
95
Survival probability
Health−adjusted survival probability

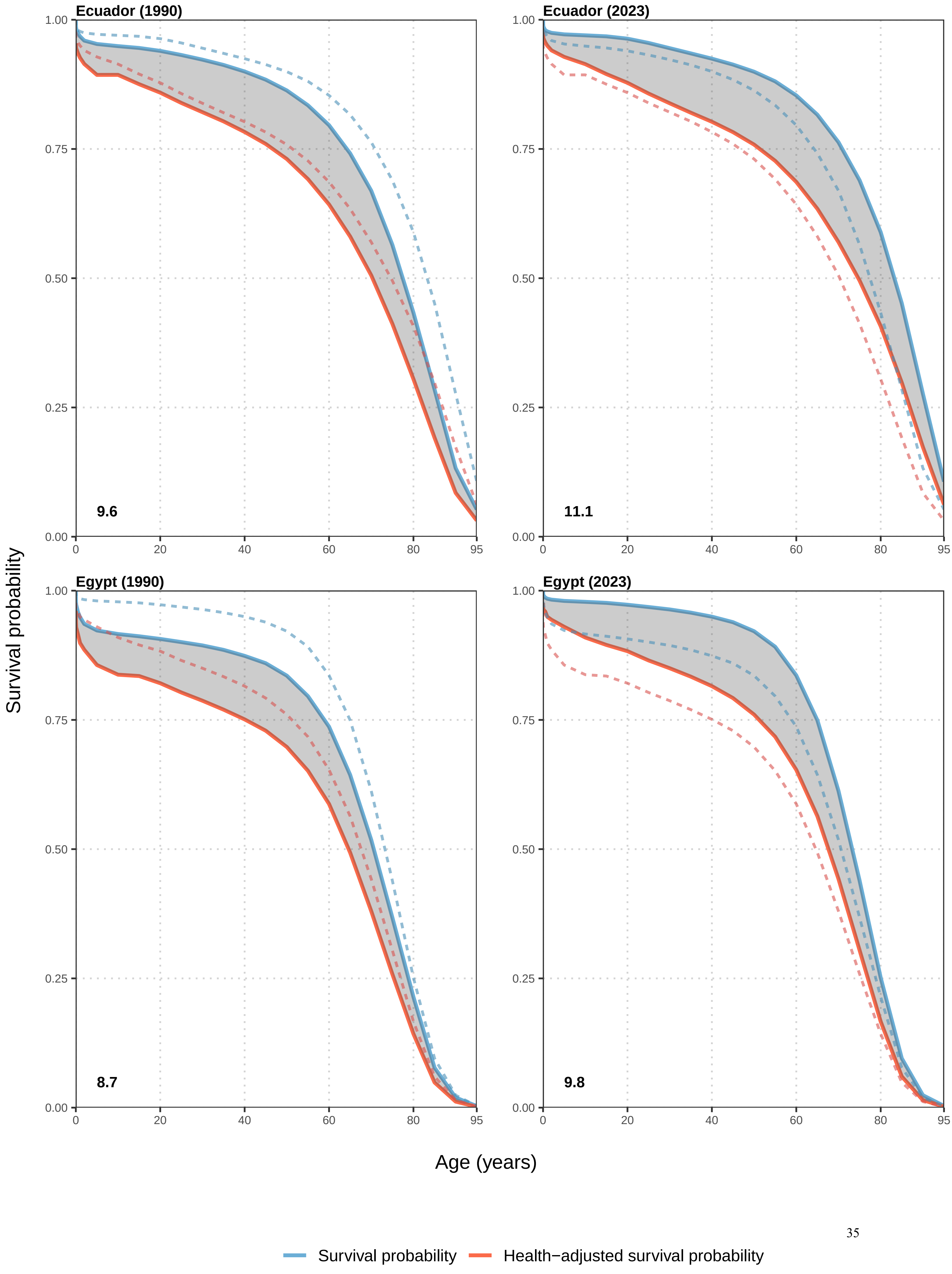
Ecuador (1990)
Ecuador (2023)
Egypt (1990)
Egypt (2023)
9.6
11.1
8.7
9.8
1.00
0.75
0.50
0.25
0.00
0
20
40
60
80
95
Survival probability
Age (years)
Survival probability
Health−adjusted survival probability

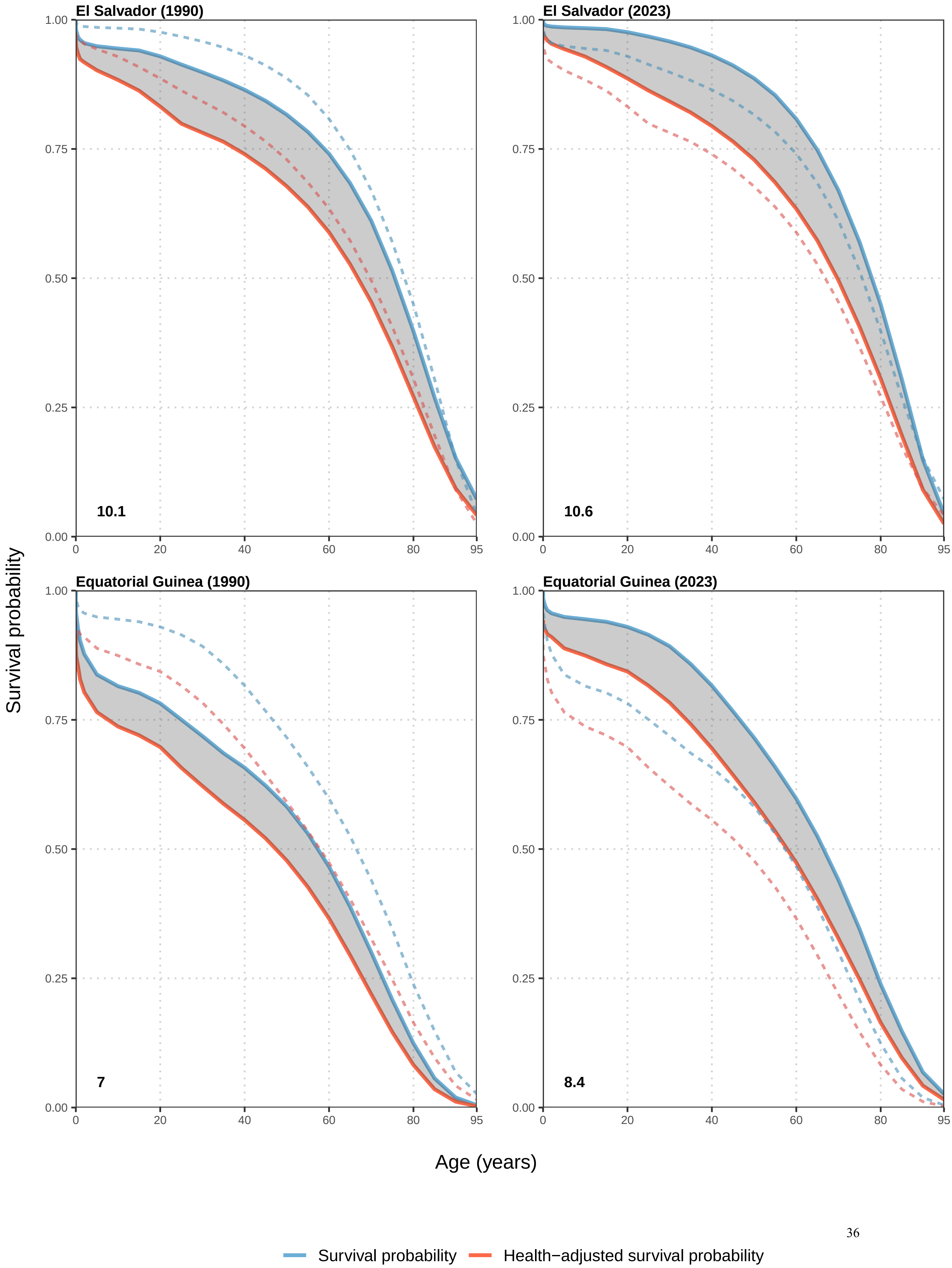
El Salvador (1990)
10.1
El Salvador (2023)
10.6
Equatorial Guinea (1990)
7
Equatorial Guinea (2023)
8.4
Survival probability
Age (years)
1.00
0.75
0.50
0.25
0.00
0
20
40
60
80
95
Survival probability
Health−adjusted survival probability

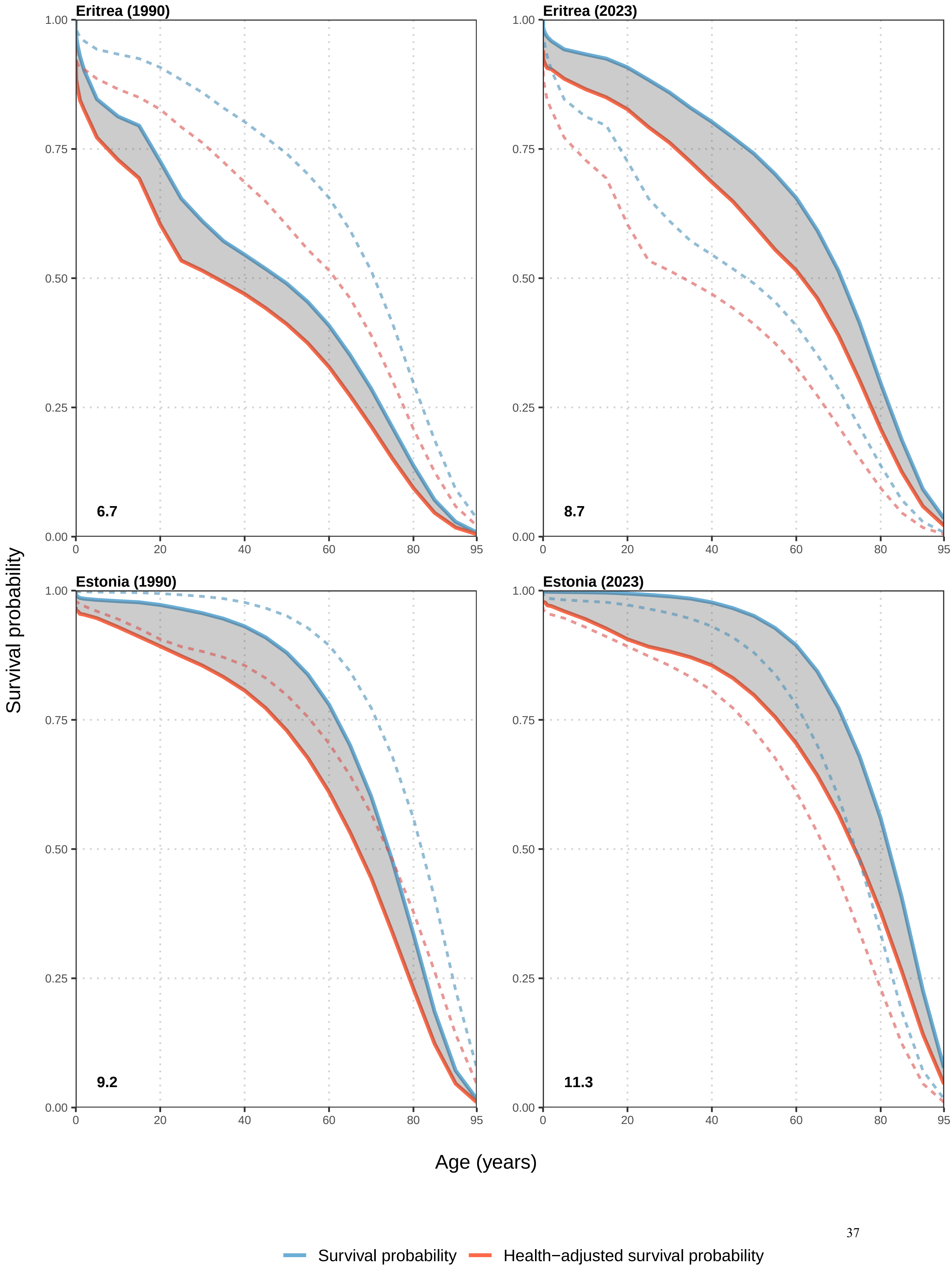
Eritrea (1990)
Eritrea (2023)
Estonia (1990)
Estonia (2023)
6.7
8.7
9.2
11.3
Survival probability
Age (years)
Survival probability
Health−adjusted survival probability

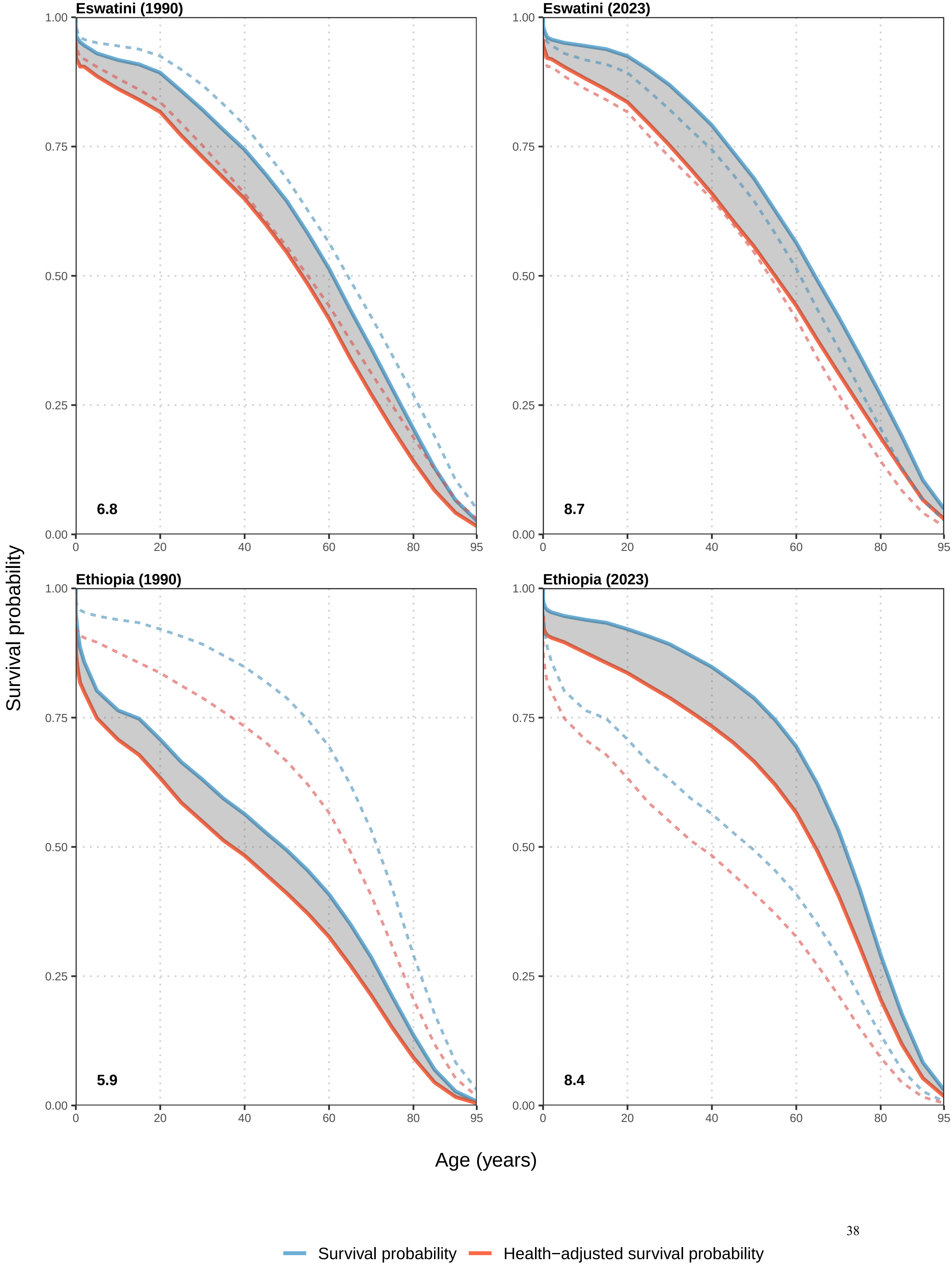
Eswatini (1990)
Eswatini (2023)
Ethiopia (1990)
Ethiopia (2023)
6.8
8.7
5.9
8.4
1.00
0.75
0.50
0.25
0.00
0
20
40
60
80
95
Survival probability
Age (years)
Survival probability
Health−adjusted survival probability

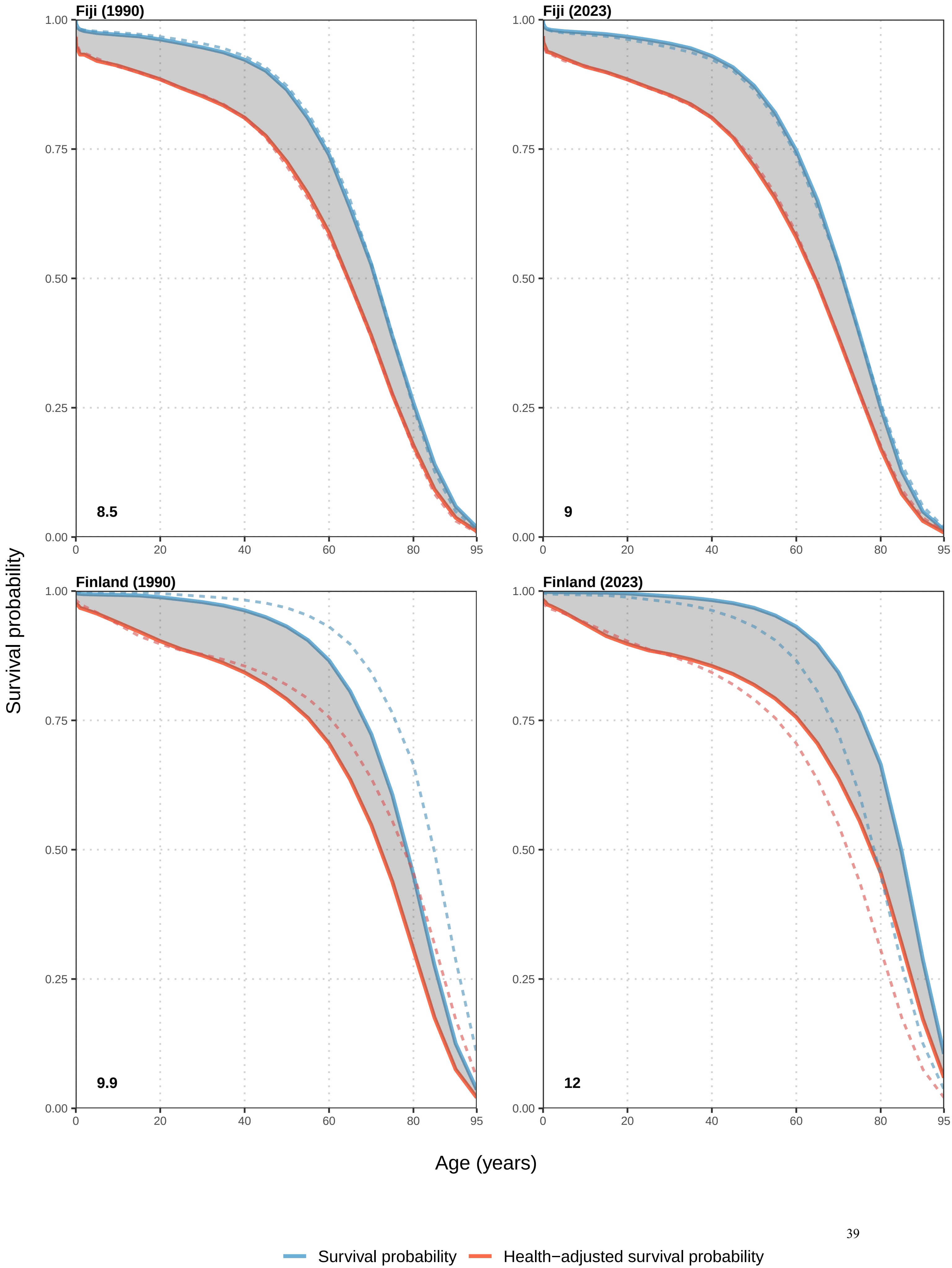

Fiji (1990)
Fiji (2023)
Finland (1990)
Finland (2023)
8.5
9
9.9
12
1.00
0.75
0.50
0.25
0.00
0
20
40
60
80
95
Survival probability
Age (years)
Survival probability
Health−adjusted survival probability

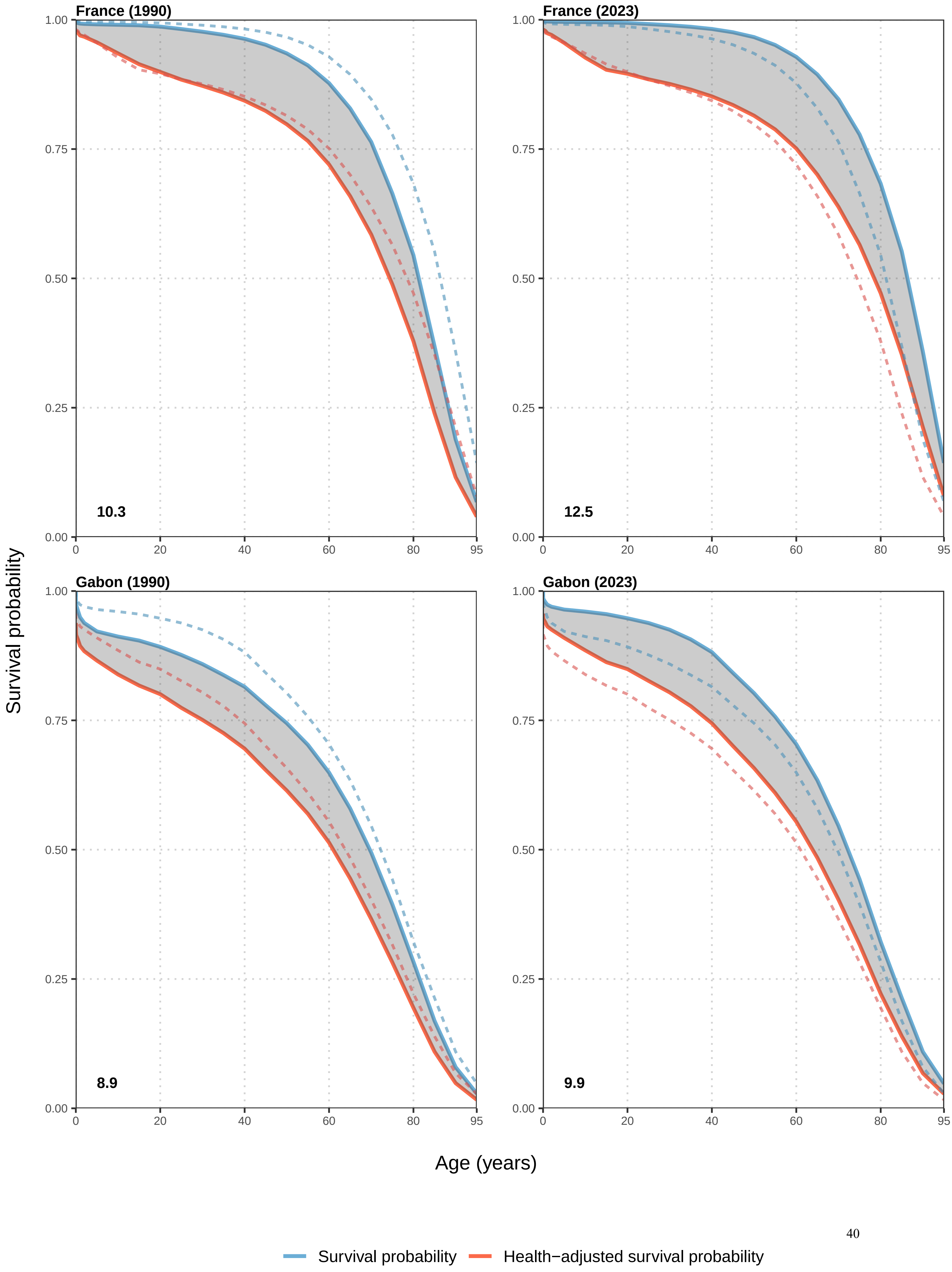
France (1990)
France (2023)
Gabon (1990)
Gabon (2023)
10.3
12.5
8.9
9.9
Survival probability
Age (years)
0.00
0.25
0.50
0.75
1.00
0
20
40
60
80
95
Survival probability
Health−adjusted survival probability

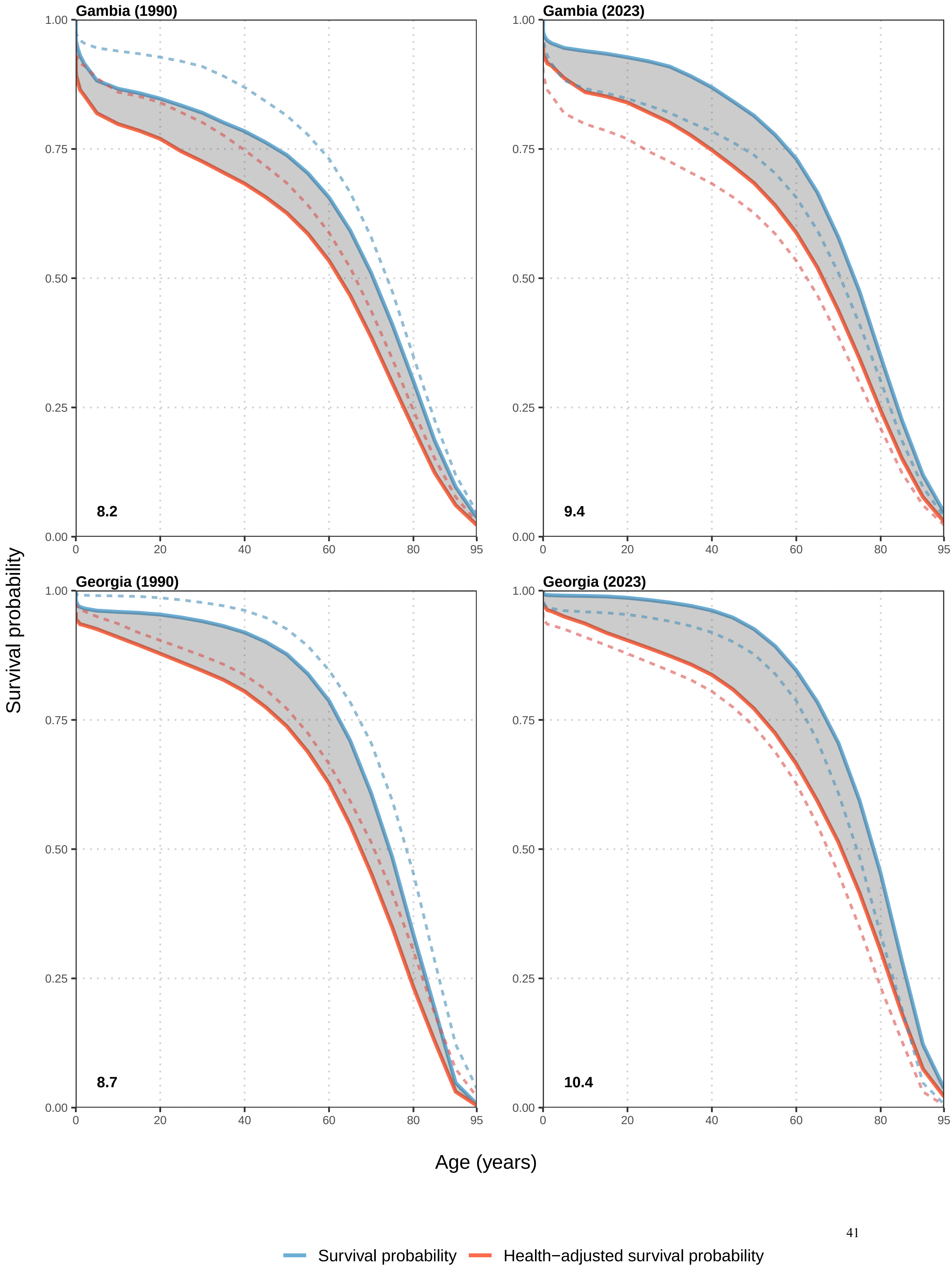

Gambia (1990)
Gambia (2023)
Georgia (1990)
Georgia (2023)
8.2
9.4
8.7
10.4
Survival probability
Age (years)
Survival probability
Health−adjusted survival probability

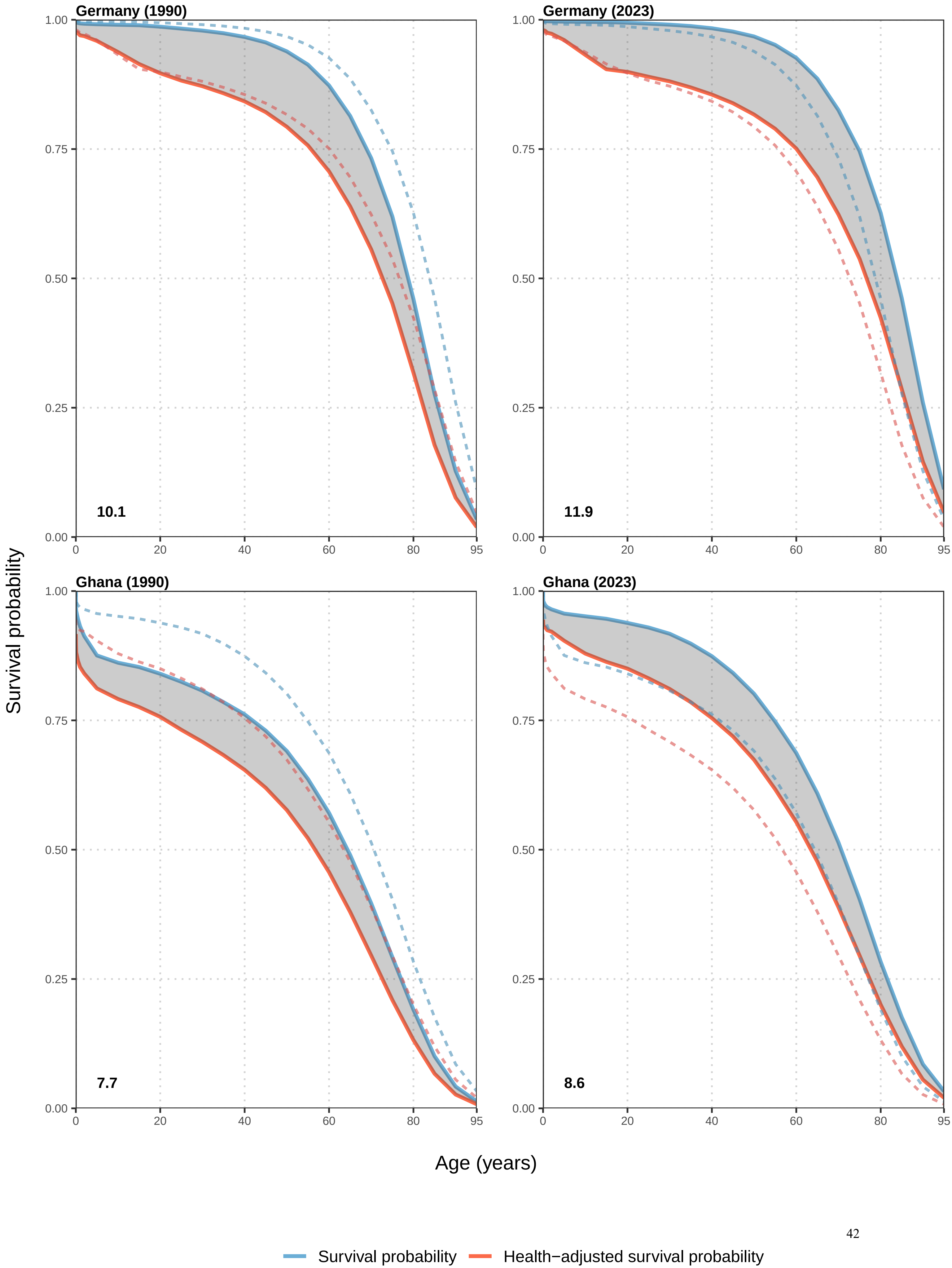
Germany (1990)
Germany (2023)
Ghana (1990)
Ghana (2023)
10.1
11.9
7.7
8.6
1.00
0.75
0.50
0.25
0.00
0
20
40
60
80
95
Survival probability
Age (years)
Survival probability
Health−adjusted survival probability

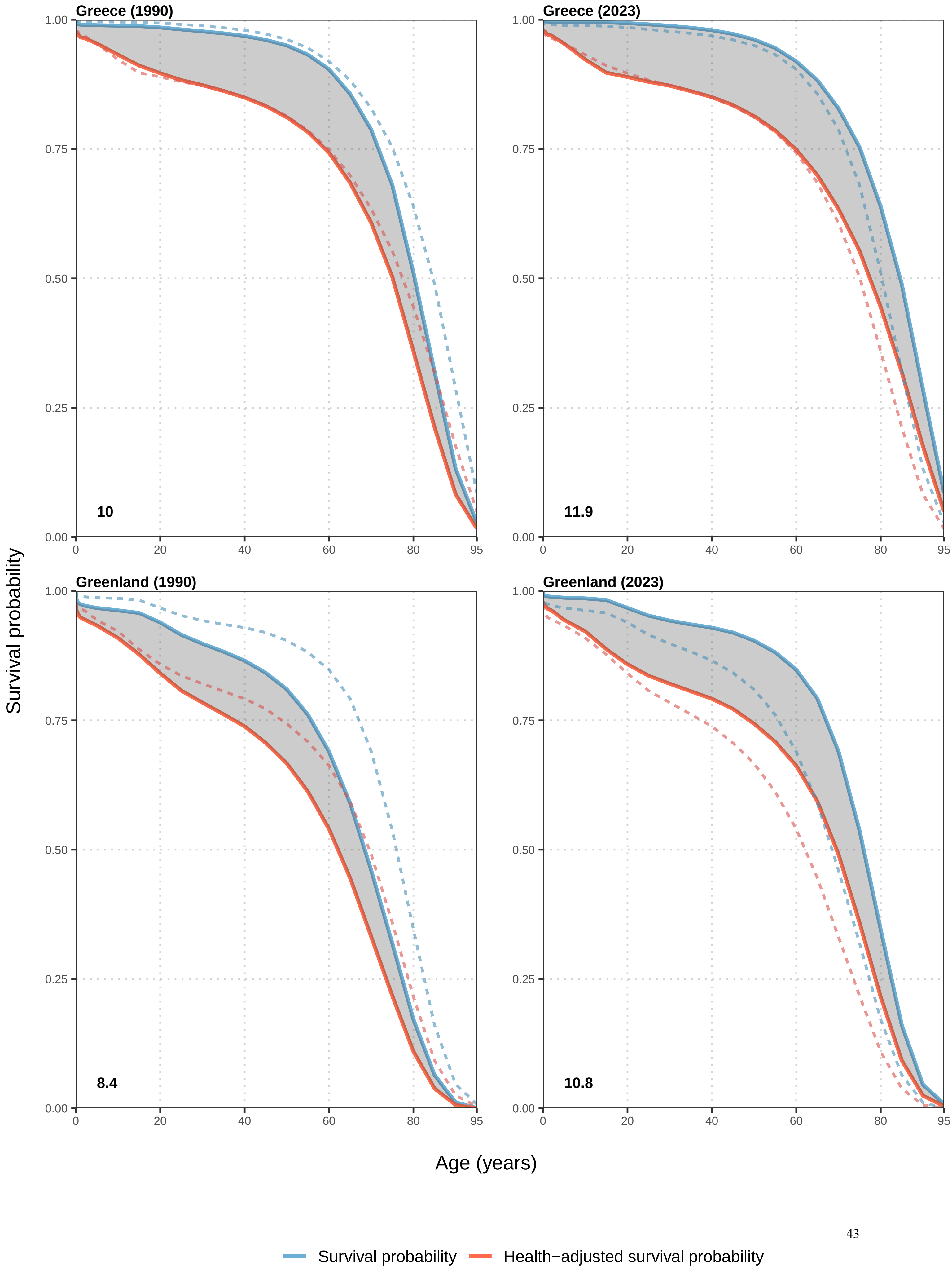

Greece (1990)
Greece (2023)
Greenland (1990)
Greenland (2023)
10
11.9
8.4
10.8
1.00
0.75
0.50
0.25
0.00
0
20
40
60
80
95
Survival probability
Age (years)
Survival probability
Health−adjusted survival probability

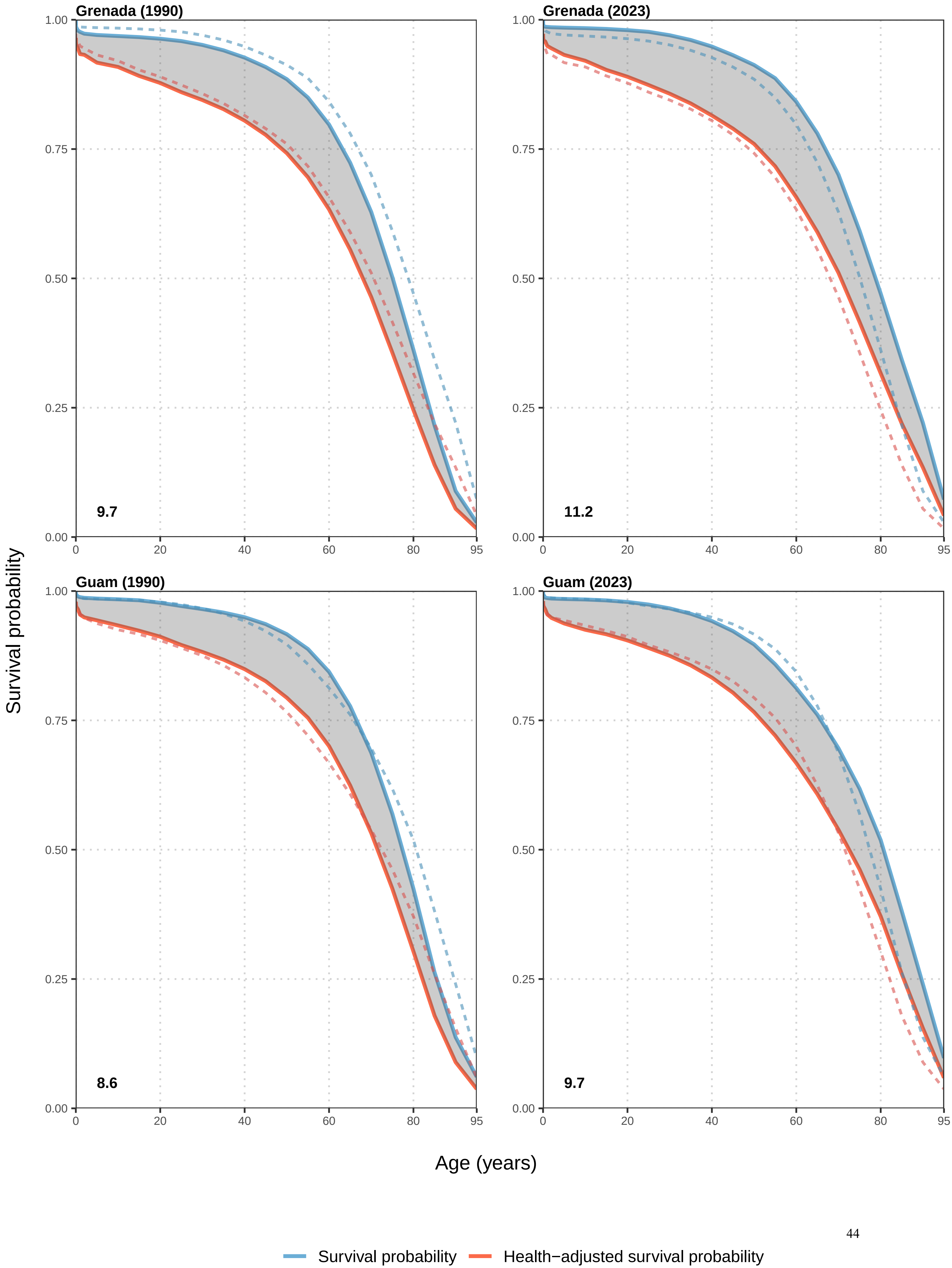

Grenada (1990)
Grenada (2023)
Guam (1990)
Guam (2023)
9.7
11.2
8.6
9.7
1.00
0.75
0.50
0.25
0.00
0
20
40
60
80
95
Survival probability
Age (years)
Survival probability
Health−adjusted survival probability

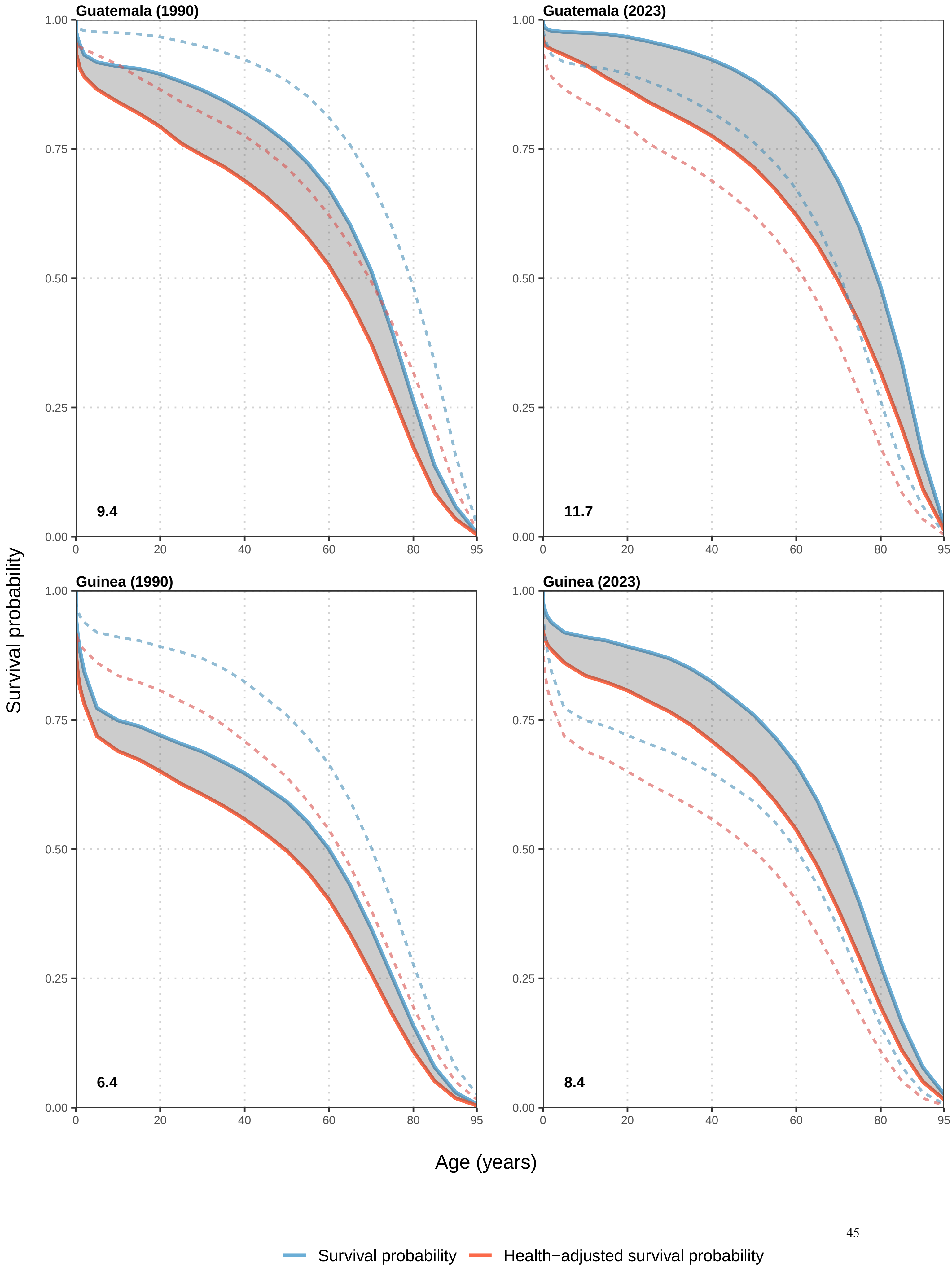
Guatemala (1990)
9.4
Guatemala (2023)
11.7
Guinea (1990)
6.4
Guinea (2023)
8.4
Survival probability
Age (years)
Survival probability
Health−adjusted survival probability

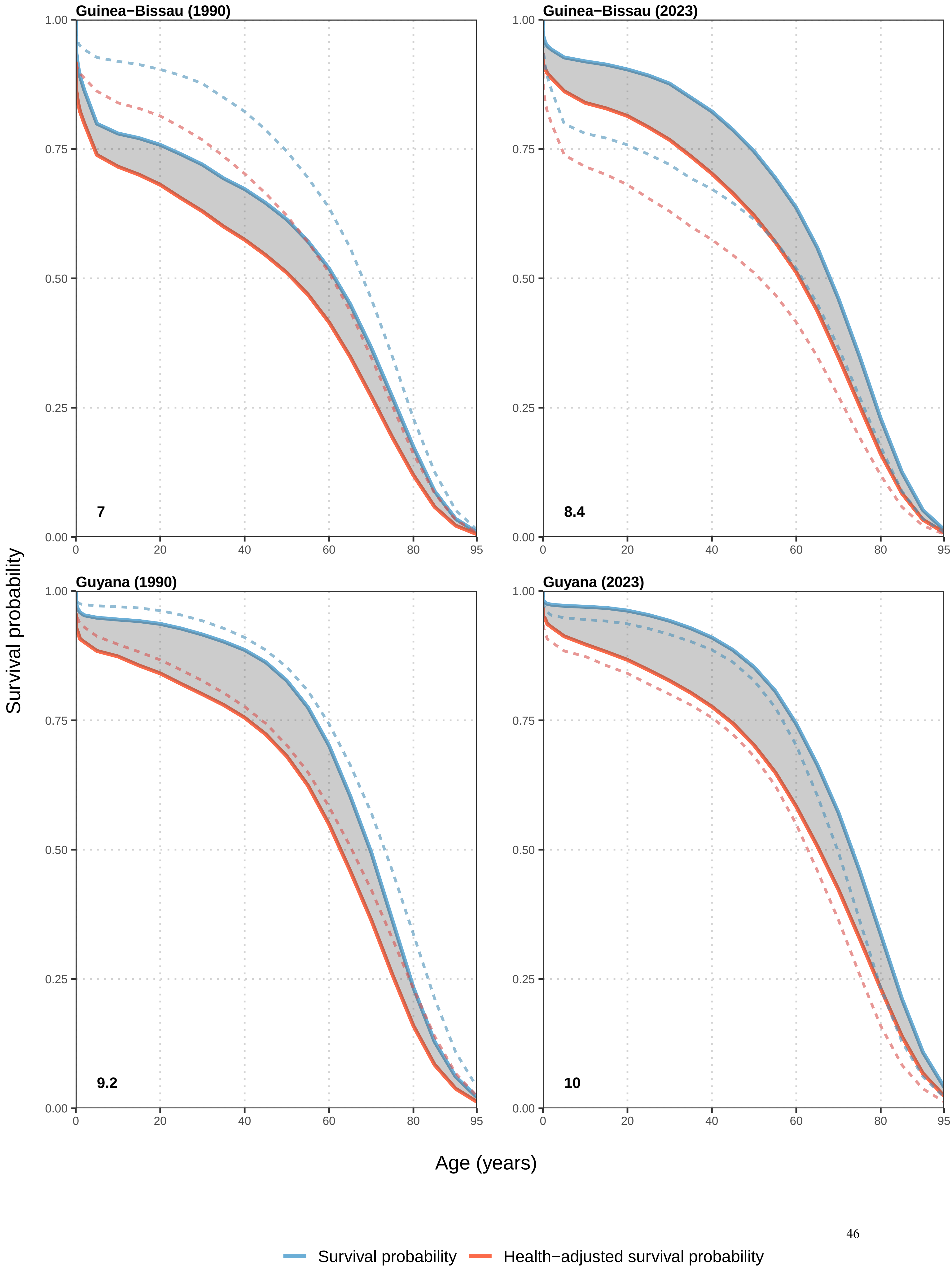
Guinea−Bissau (1990)
Guinea−Bissau (2023)
Guyana (1990)
Guyana (2023)
7
8.4
9.2
10
1.00
0.75
0.50
0.25
0.00
0
20
40
60
80
95
Survival probability
Age (years)
Survival probability
Health−adjusted survival probability

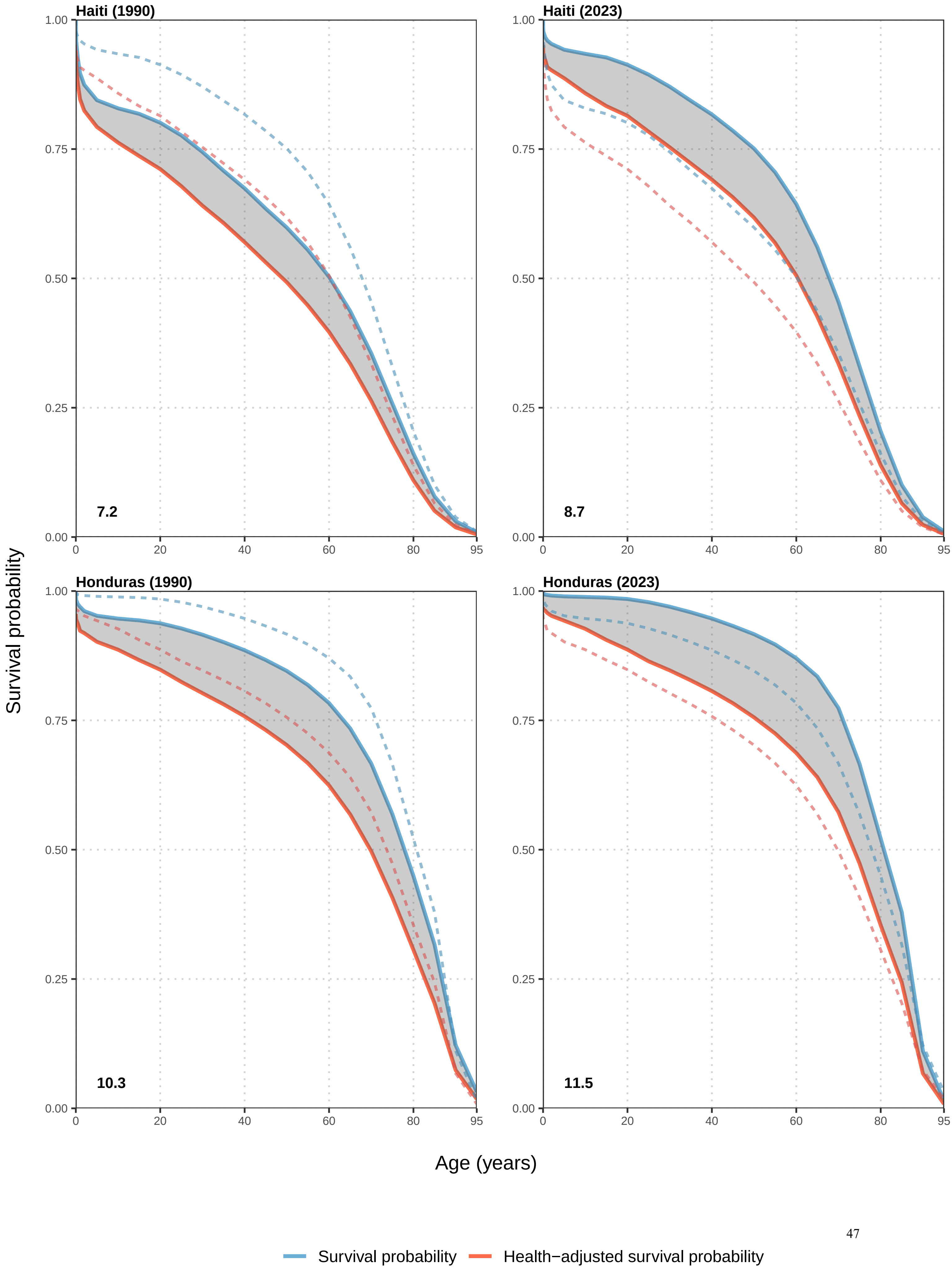

Haiti (1990)
Haiti (2023)
Honduras (1990)
Honduras (2023)
7.2
8.7
10.3
11.5
1.00
0.75
0.50
0.25
0.00
0
20
40
60
80
95
Survival probability
Age (years)
Survival probability
Health−adjusted survival probability

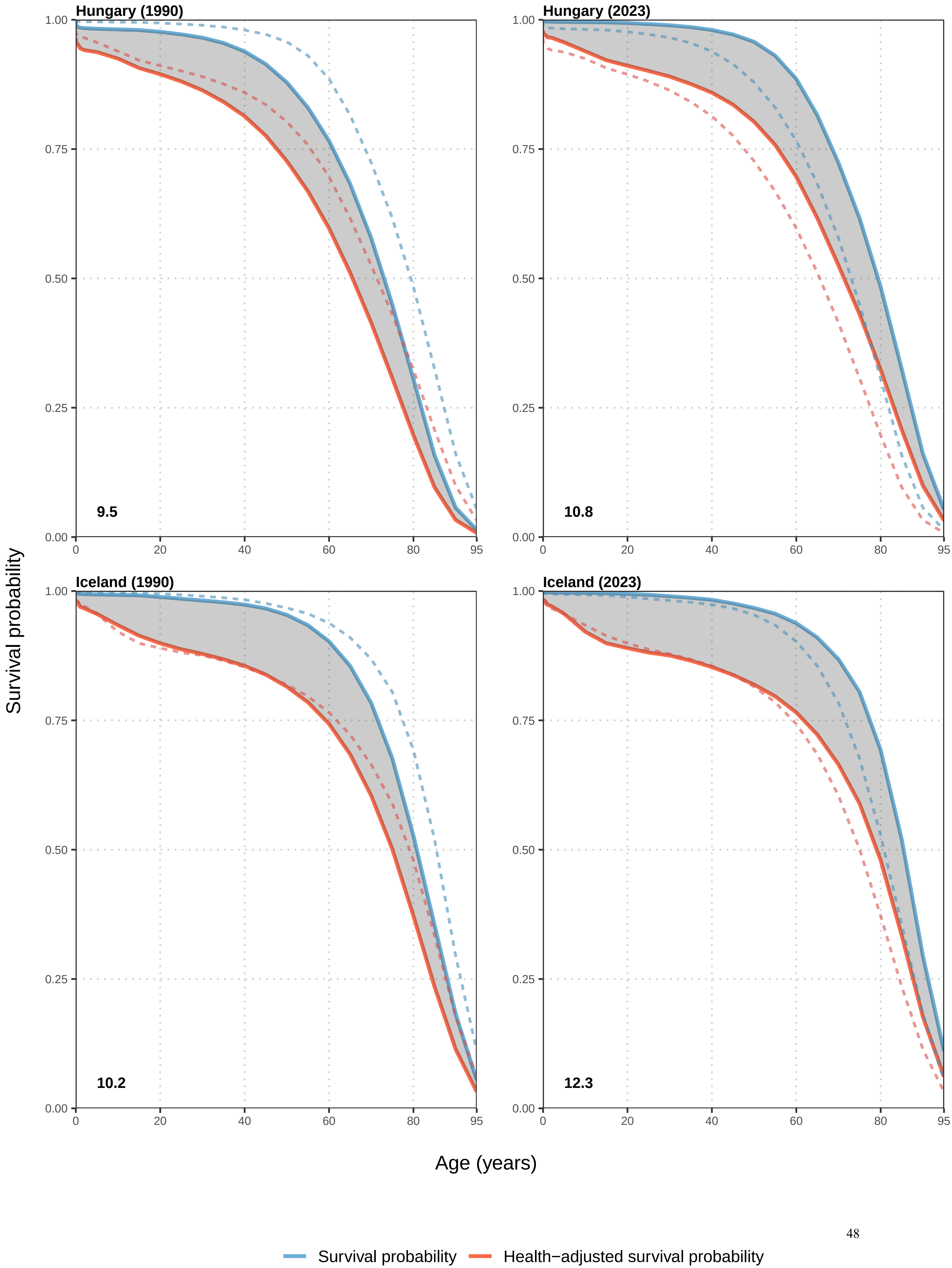
Hungary (1990)
Hungary (2023)
Iceland (1990)
Iceland (2023)
9.5
10.8
10.2
12.3
1.00
0.75
0.50
0.25
0.00
0
20
40
60
80
95
Survival probability
Age (years)
Survival probability
Health−adjusted survival probability

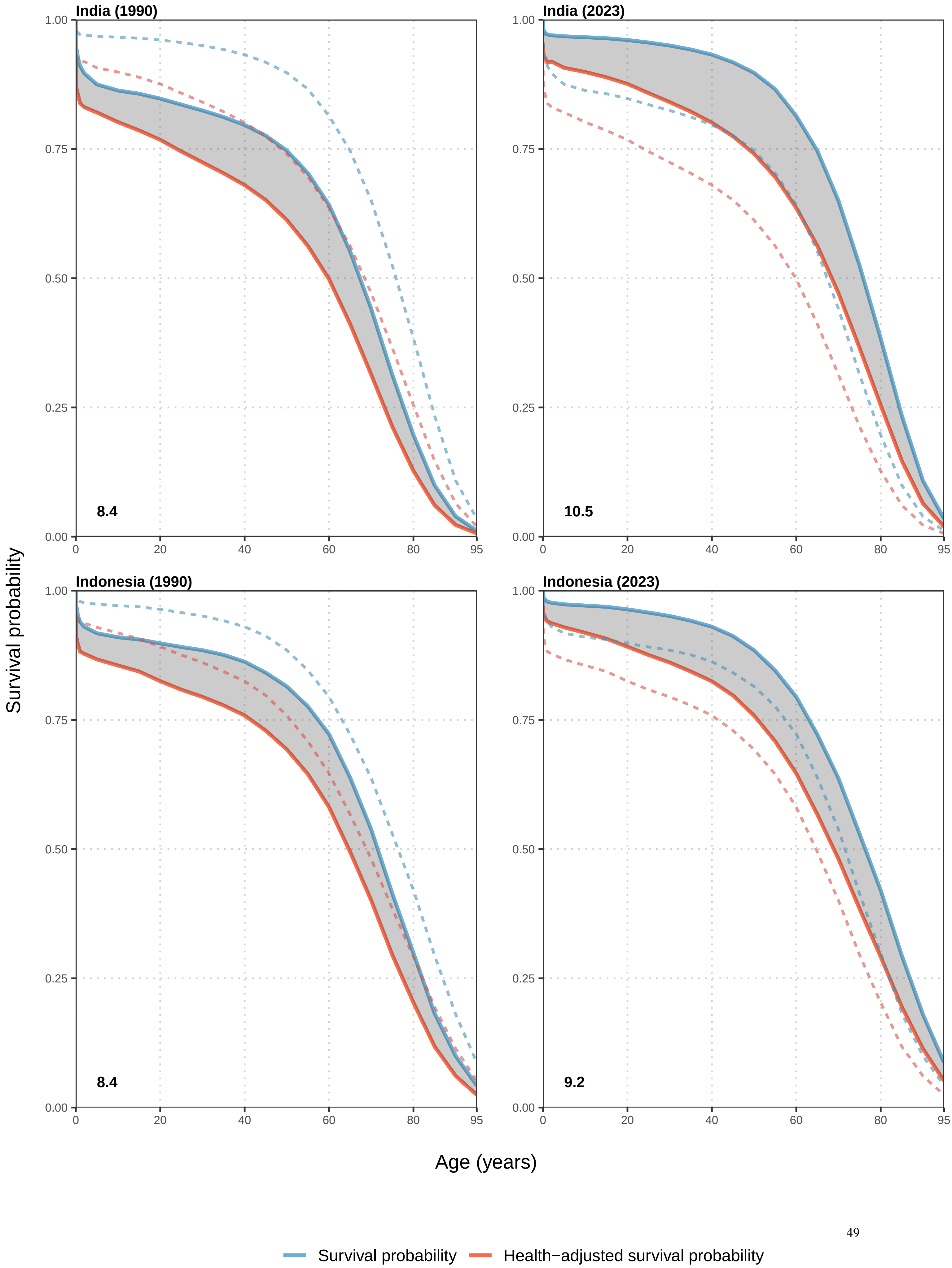
India (1990)
India (2023)
Indonesia (1990)
Indonesia (2023)
8.4
10.5
8.4
9.2
Survival probability
Age (years)
1.00
0.75
0.50
0.25
0.00
0
20
40
60
80
95
Survival probability
Health−adjusted survival probability

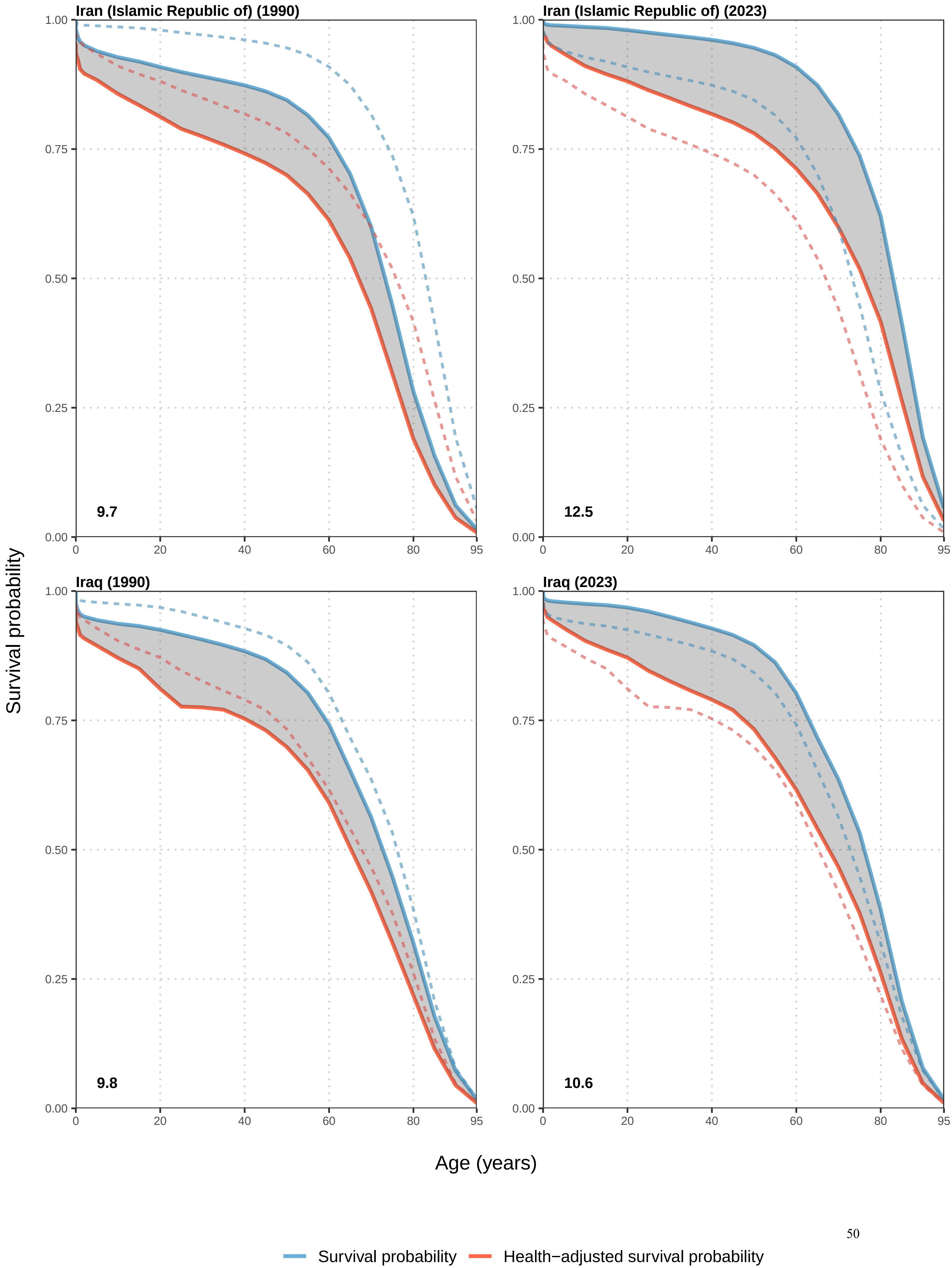

Iran (Islamic Republic of) (1990)
9.7
Iran (Islamic Republic of) (2023)
12.5
Iraq (1990)
9.8
Iraq (2023)
10.6
1.00
0.75
0.50
0.25
0.00
0
20
40
60
80
95
Survival probability
Age (years)
Survival probability
Health−adjusted survival probability

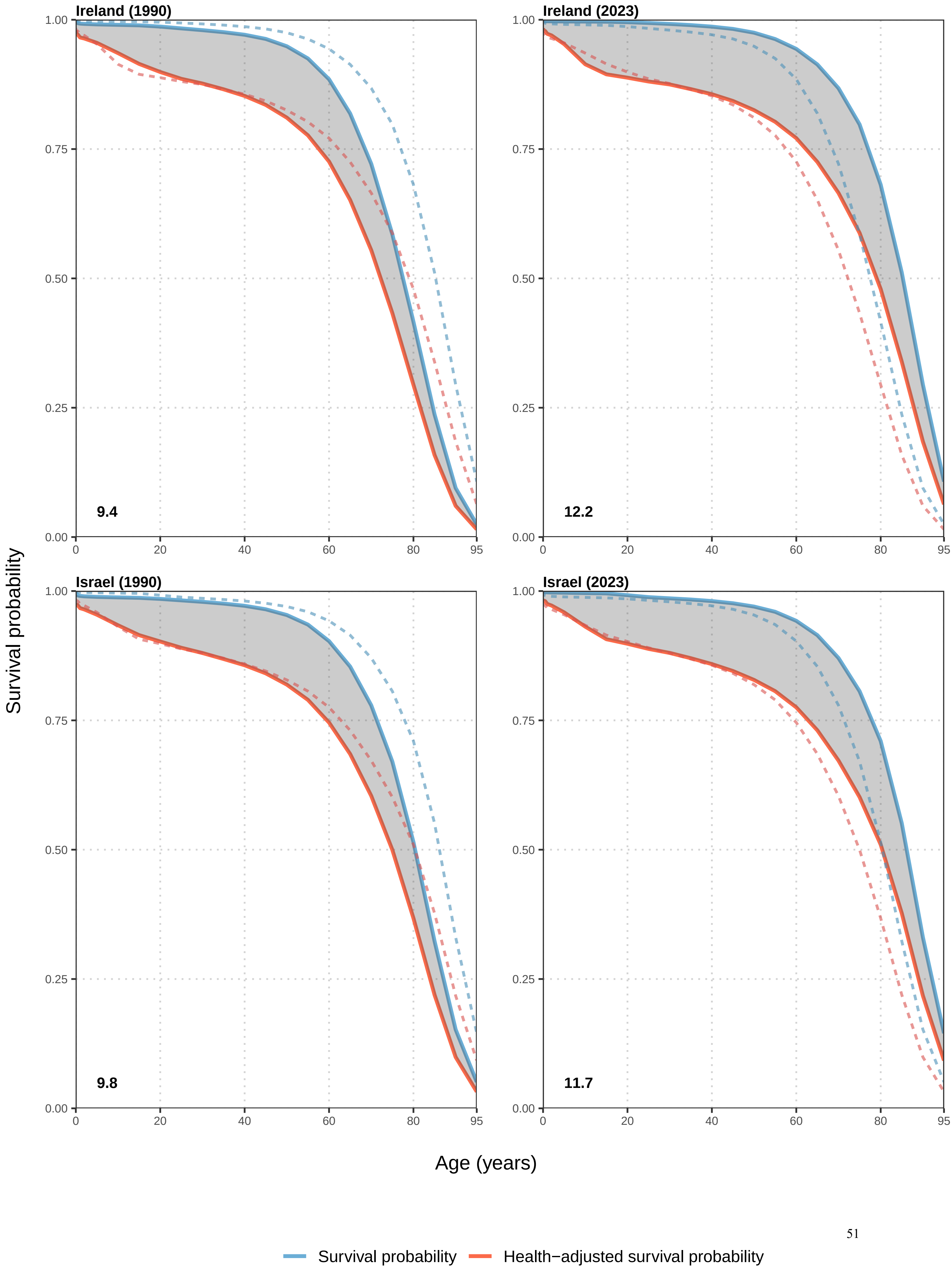

Ireland (1990)
Ireland (2023)
Israel (1990)
Israel (2023)
9.4
12.2
9.8
11.7
Survival probability
Age (years)
1.00
0.75
0.50
0.25
0.00
0
20
40
60
80
95
Survival probability
Health−adjusted survival probability

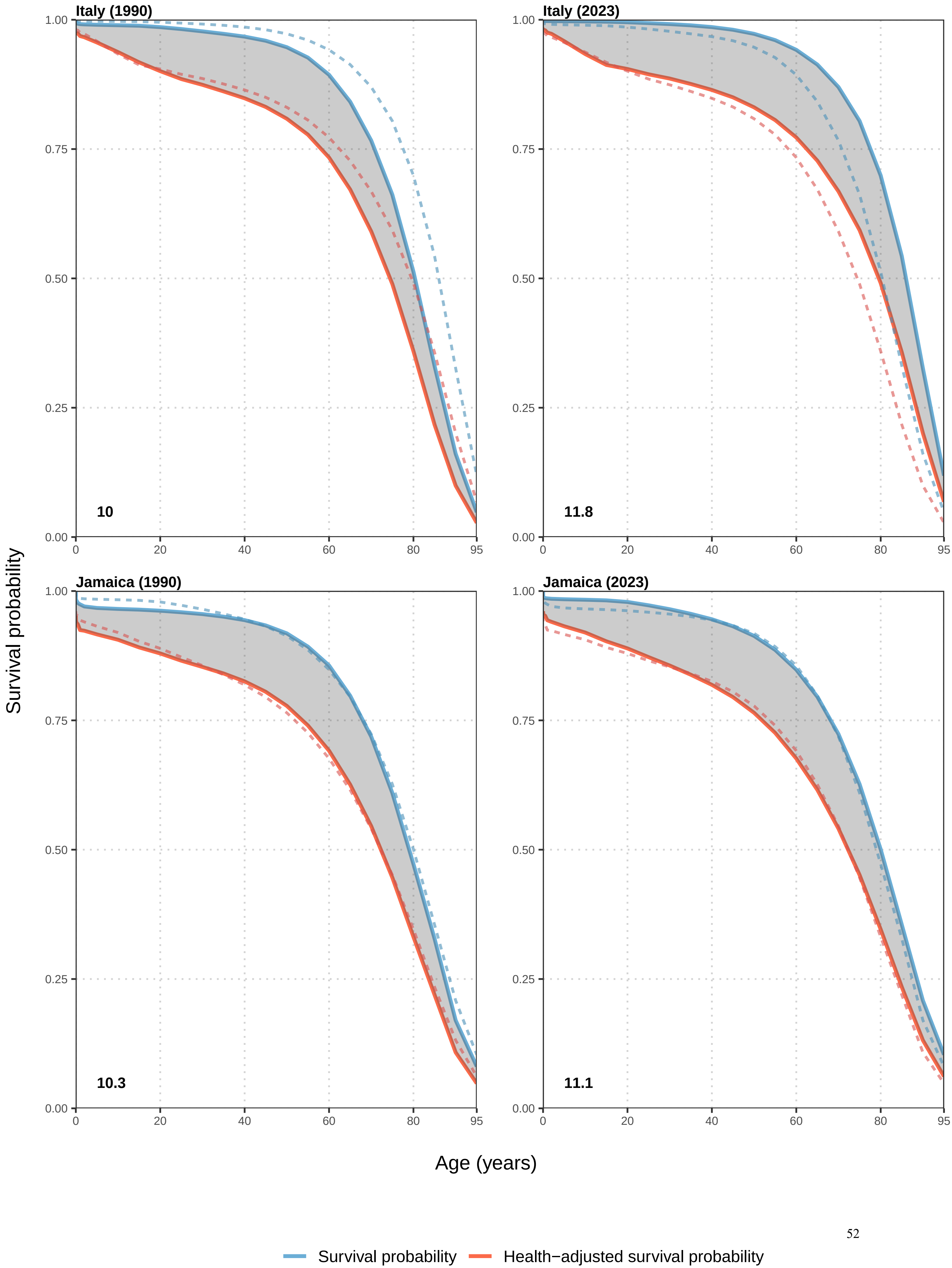

Italy (1990)
10
Italy (2023)
11.8
Jamaica (1990)
10.3
Jamaica (2023)
11.1
Survival probability
Age (years)
Survival probability
Health−adjusted survival probability

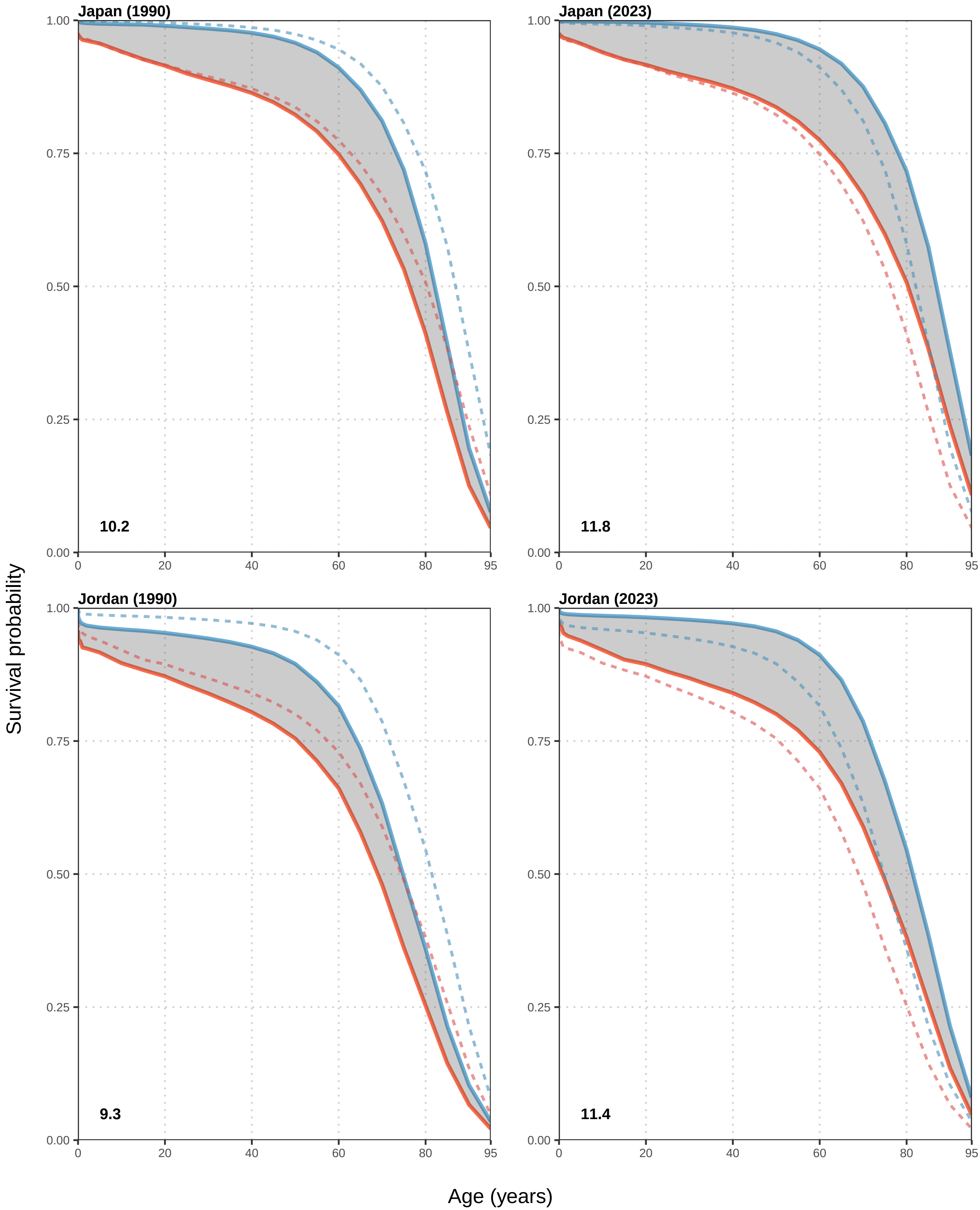

Survival probability Health−adjusted survival probability

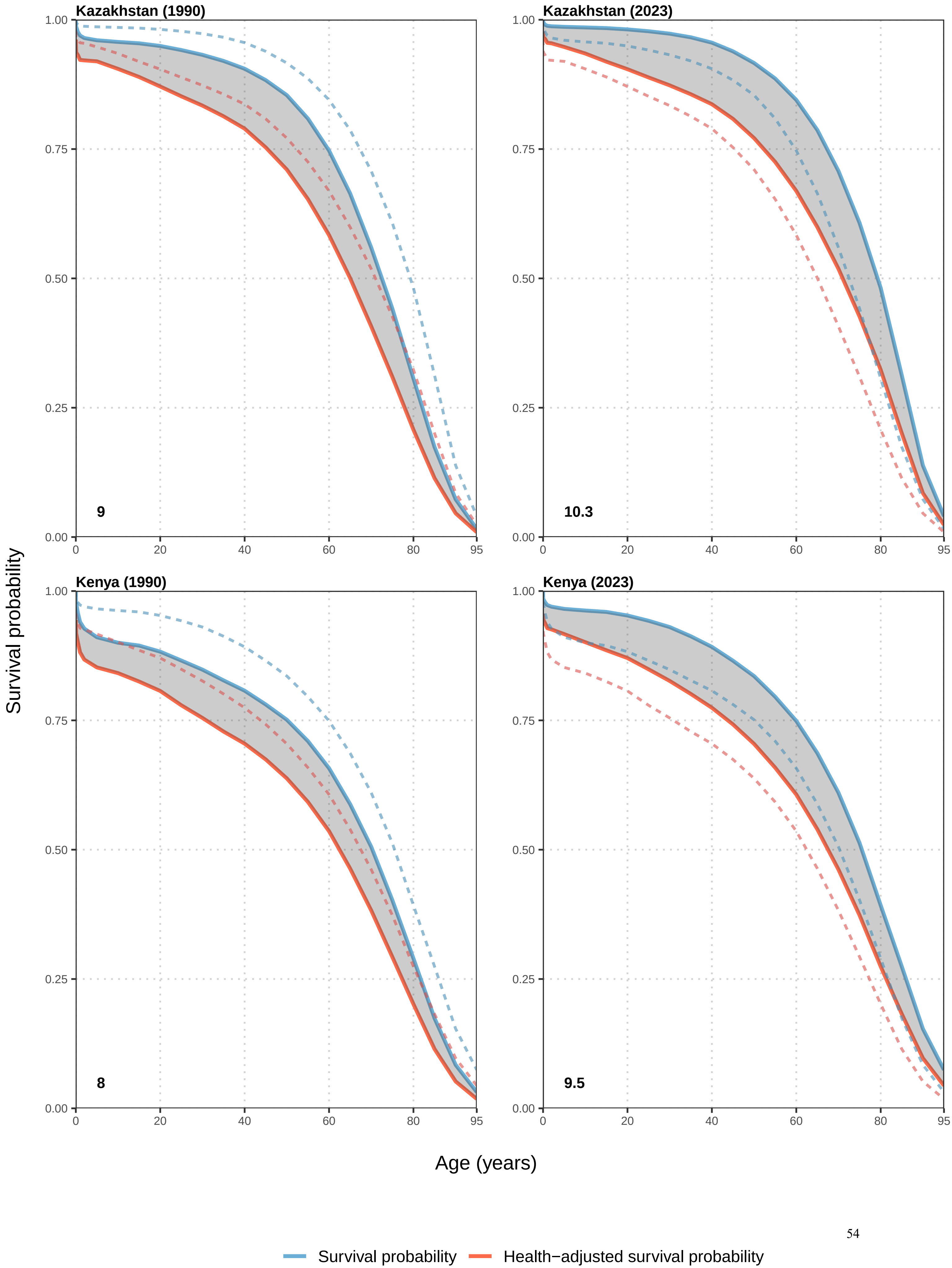

Kazakhstan (1990)
Kazakhstan (2023)
Kenya (1990)
Kenya (2023)
9
10.3
8
9.5
1.00
0.75
0.50
0.25
0.00
0
20
40
60
80
95
Survival probability
Age (years)
Survival probability
Health−adjusted survival probability

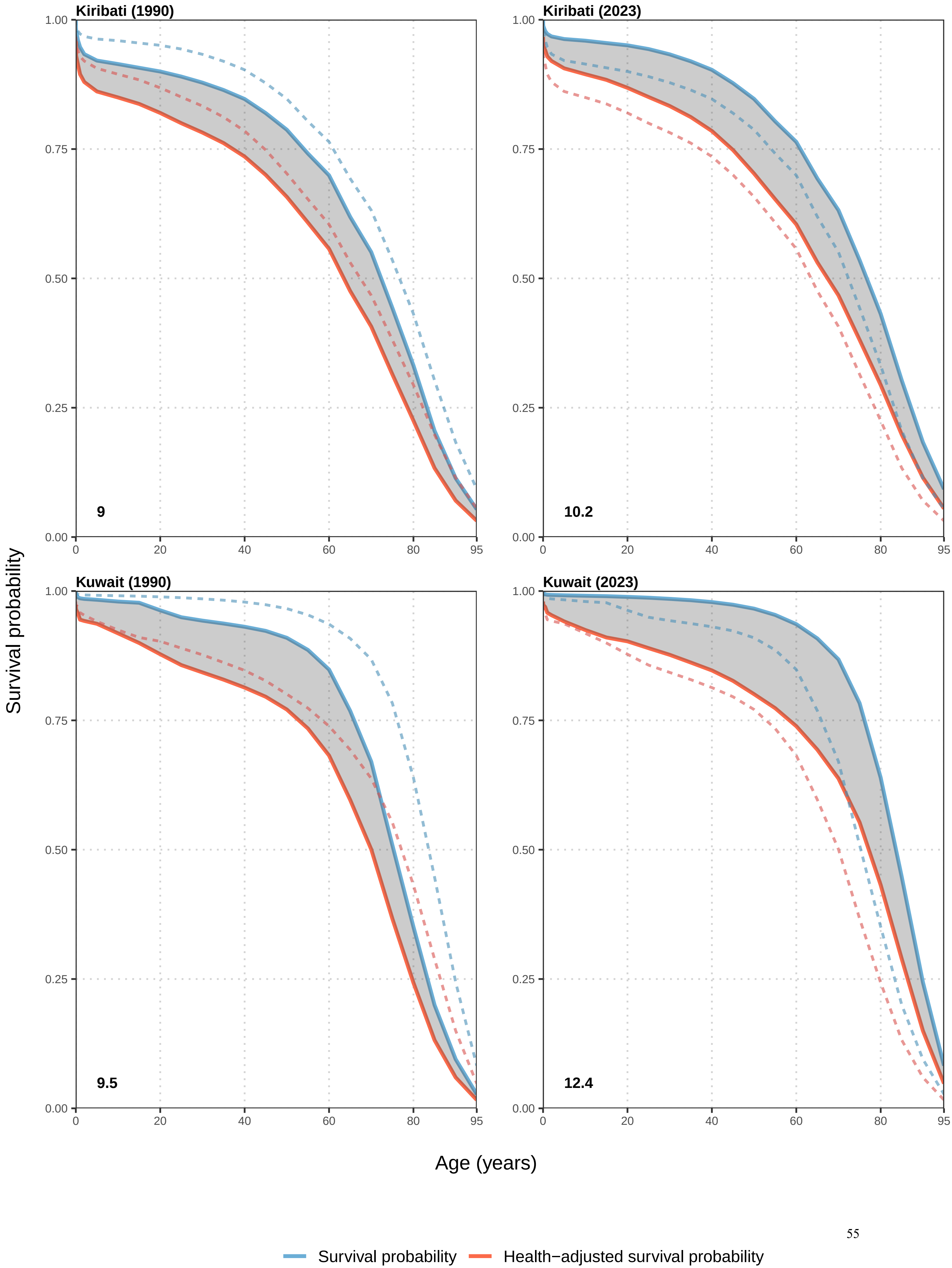

Kiribati (1990)
Kiribati (2023)
Kuwait (1990)
Kuwait (2023)
9
10.2
9.5
12.4
Survival probability
Age (years)
Survival probability
Health−adjusted survival probability

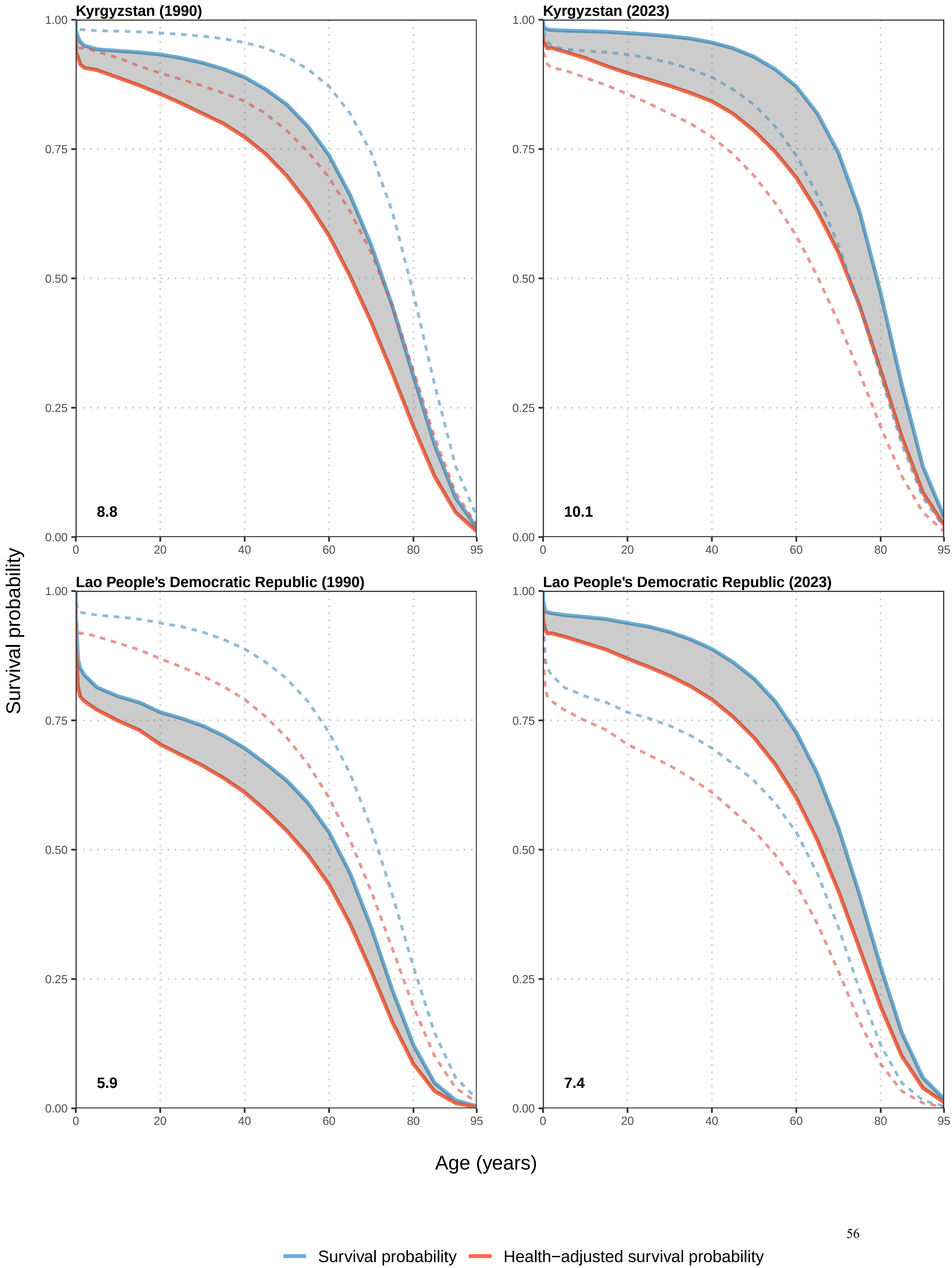
Kyrgyzstan (1990)
Kyrgyzstan (2023)
Lao People's Democratic Republic (1990)
Lao People's Democratic Republic (2023)
8.8
10.1
5.9
7.4
1.00
0.75
0.50
0.25
0.00
0
20
40
60
80
95
Survival probability
Age (years)
Survival probability
Health−adjusted survival probability

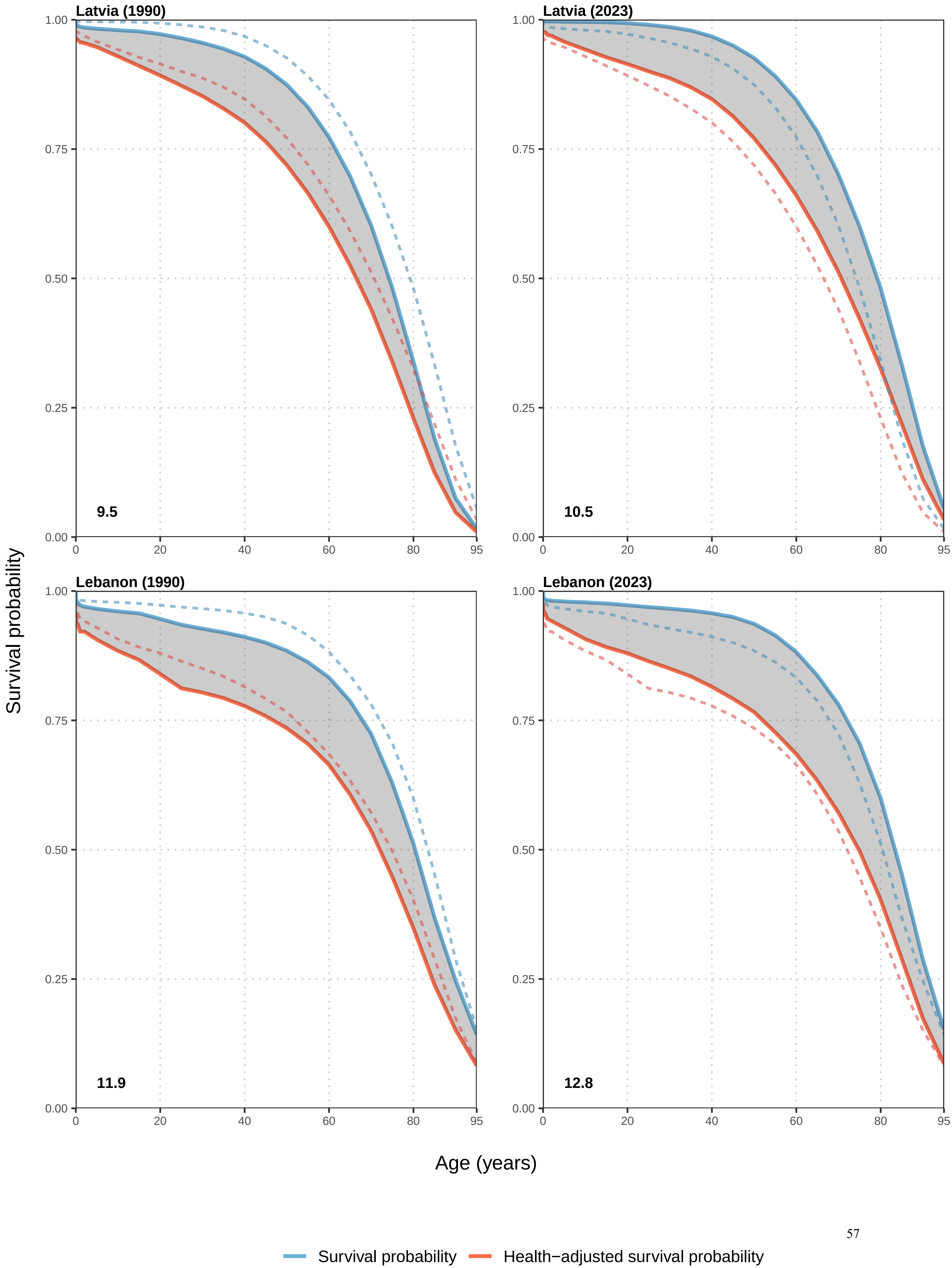

Latvia (1990)
Latvia (2023)
Lebanon (1990)
Lebanon (2023)
9.5
10.5
11.9
12.8
1.00
0.75
0.50
0.25
0.00
0
20
40
60
80
95
Survival probability
Age (years)
Survival probability
Health−adjusted survival probability

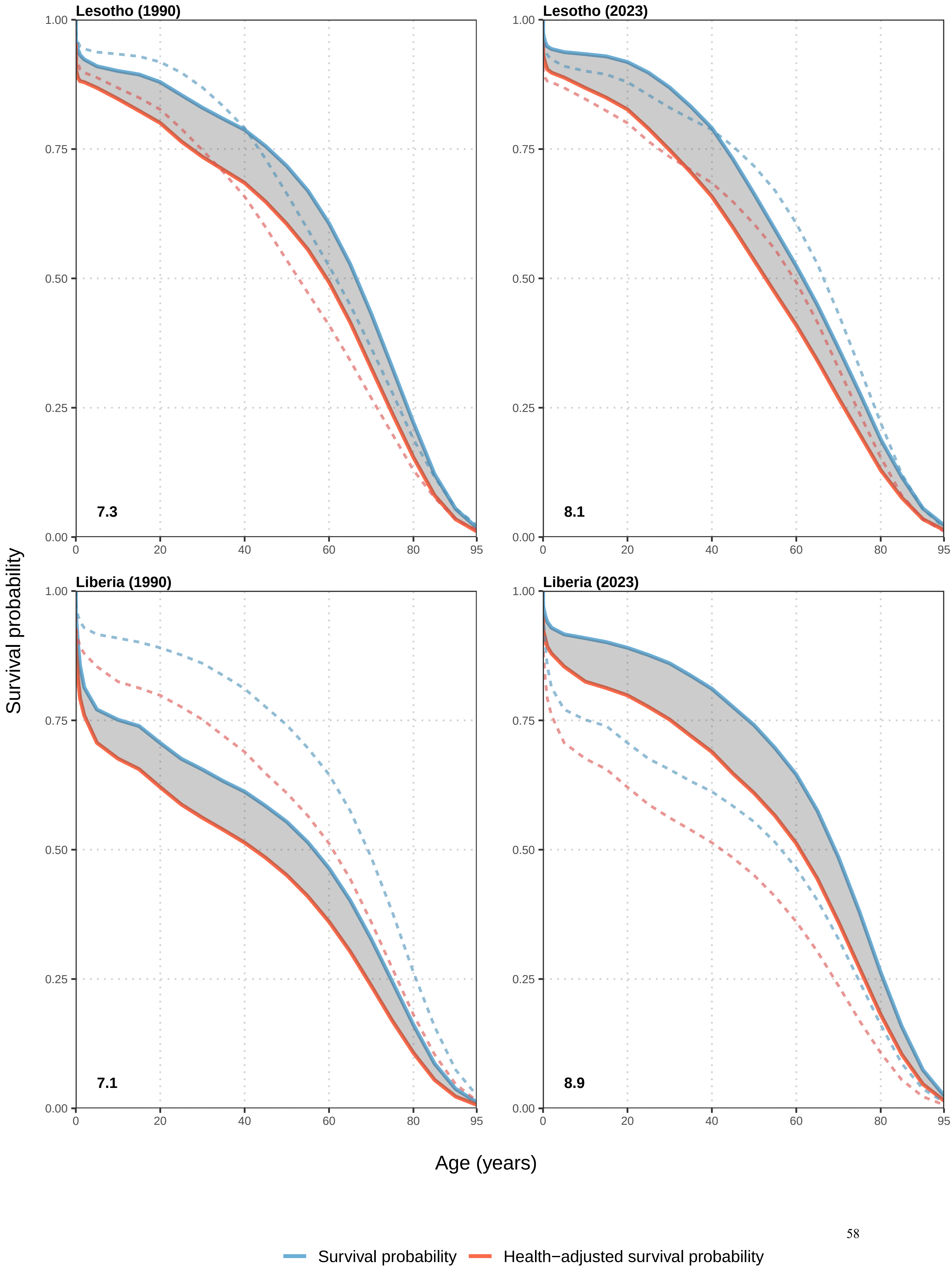

Lesotho (1990)
Lesotho (2023)
Liberia (1990)
Liberia (2023)
7.3
8.1
7.1
8.9
1.00
0.75
0.50
0.25
0.00
0
20
40
60
80
95
Survival probability
Age (years)
Survival probability
Health−adjusted survival probability

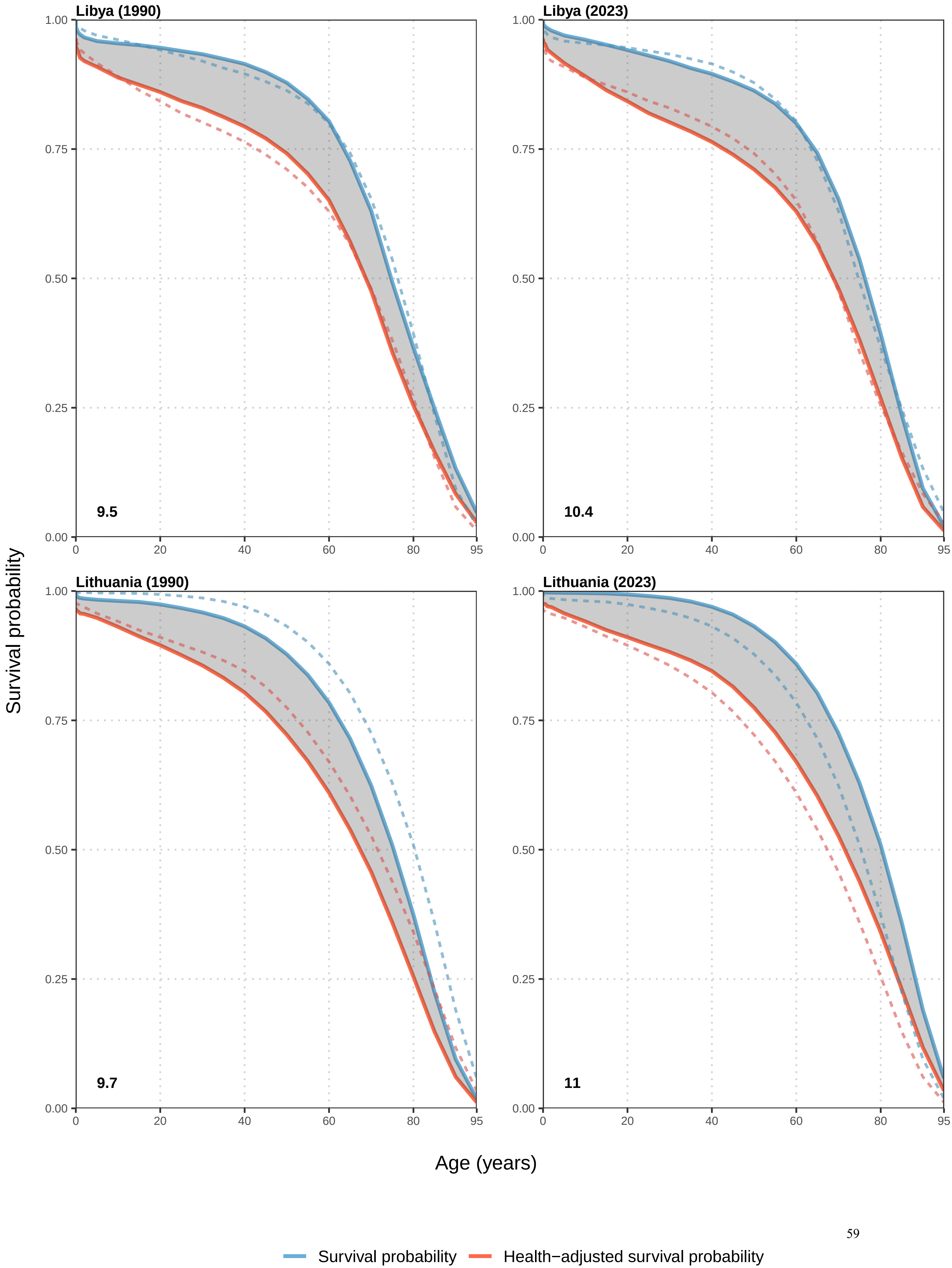

Libya (1990)
Libya (2023)
Lithuania (1990)
Lithuania (2023)
9.5
10.4
9.7
11
1.00
0.75
0.50
0.25
0.00
0
20
40
60
80
95
Survival probability
Age (years)
Survival probability
Health−adjusted survival probability

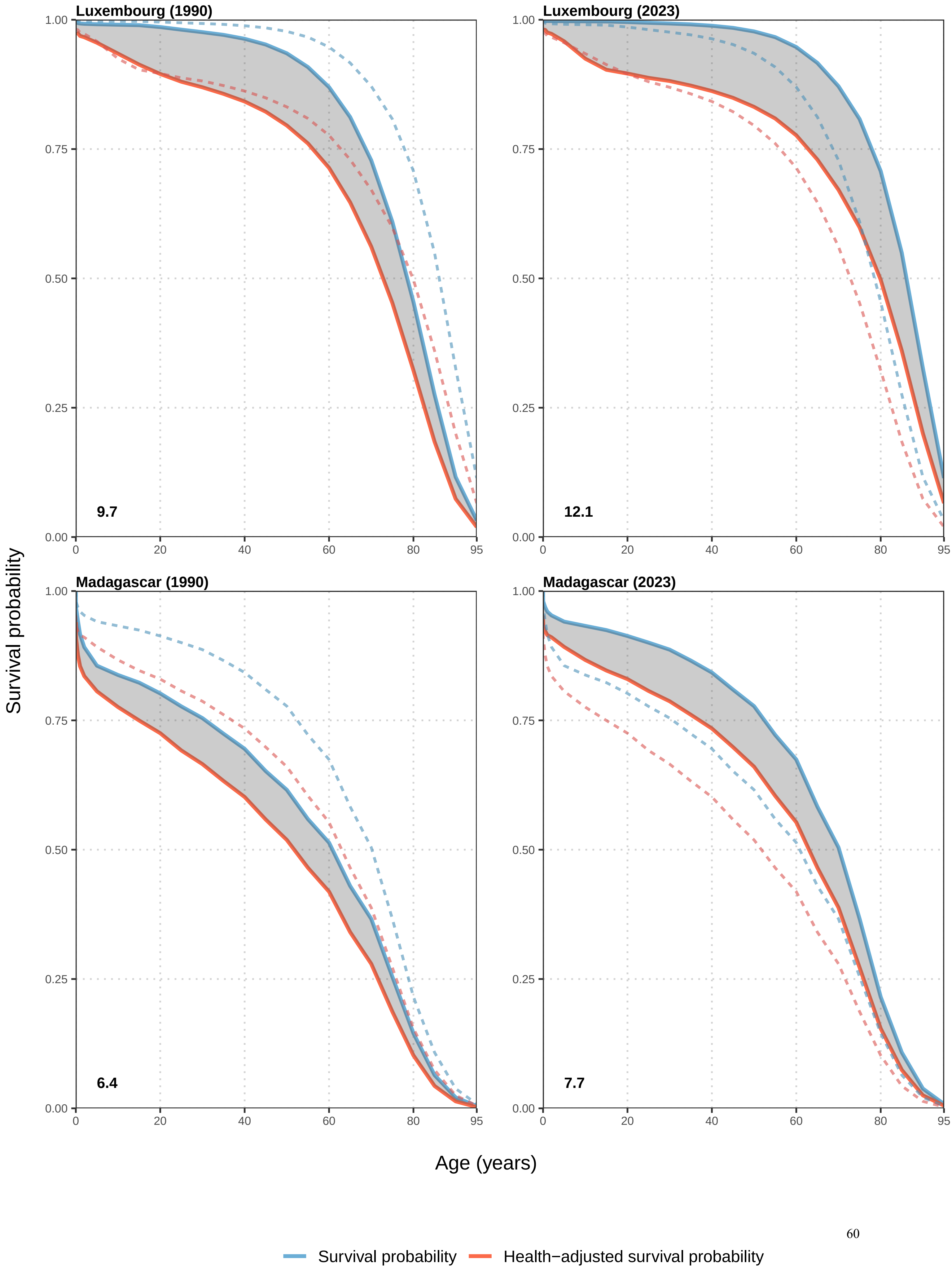
Luxembourg (1990)
9.7
Luxembourg (2023)
12.1
Madagascar (1990)
6.4
Madagascar (2023)
7.7
1.00
0.75
0.50
0.25
0.00
0
20
40
60
80
95
Survival probability
Age (years)
Survival probability
Health−adjusted survival probability

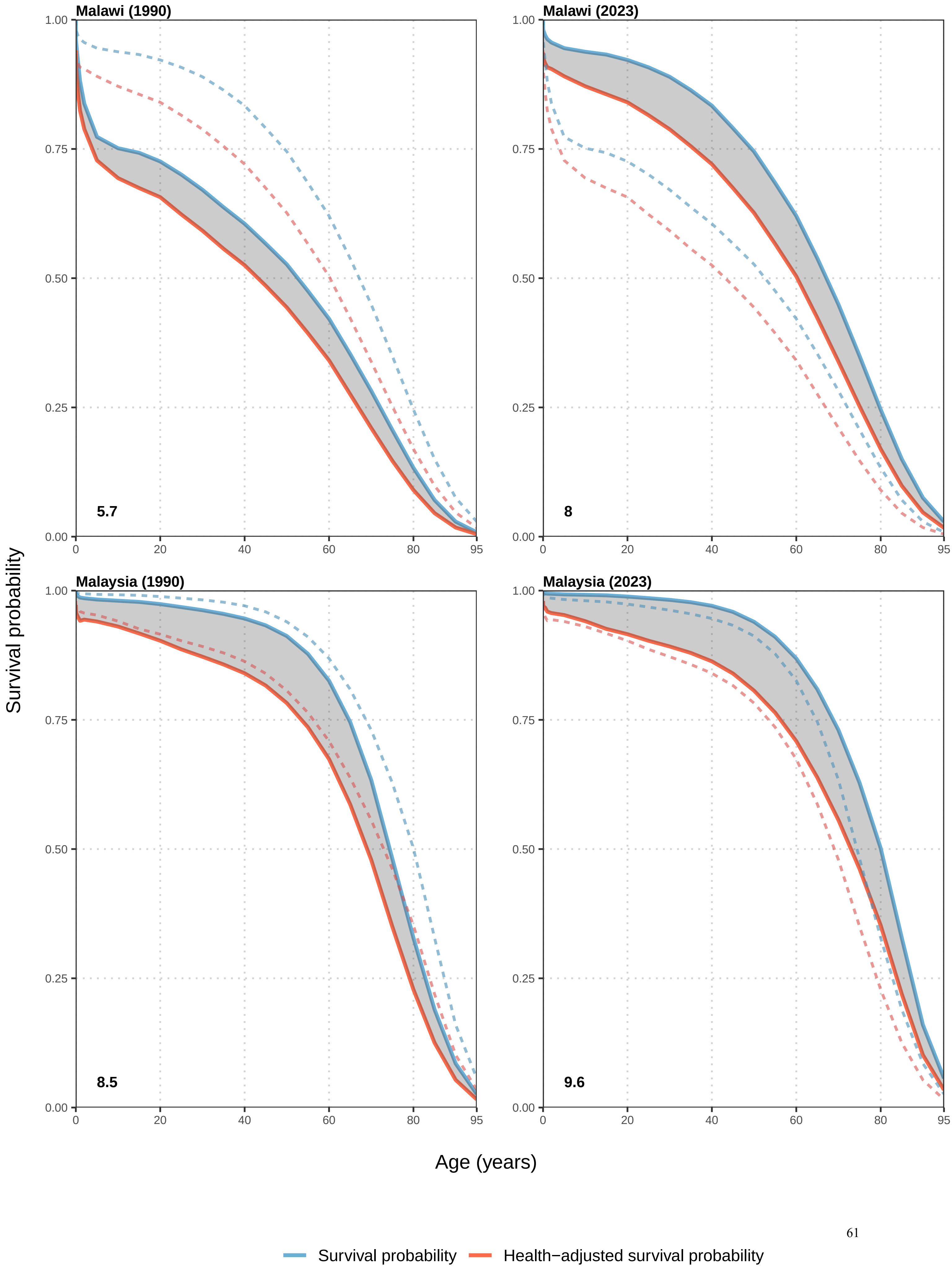
Malawi (1990)
5.7
Malawi (2023)
8
Malaysia (1990)
8.5
Malaysia (2023)
9.6
Survival probability
Age (years)
1.00
0.75
0.50
0.25
0.00
0
20
40
60
80
95
Survival probability
Health−adjusted survival probability

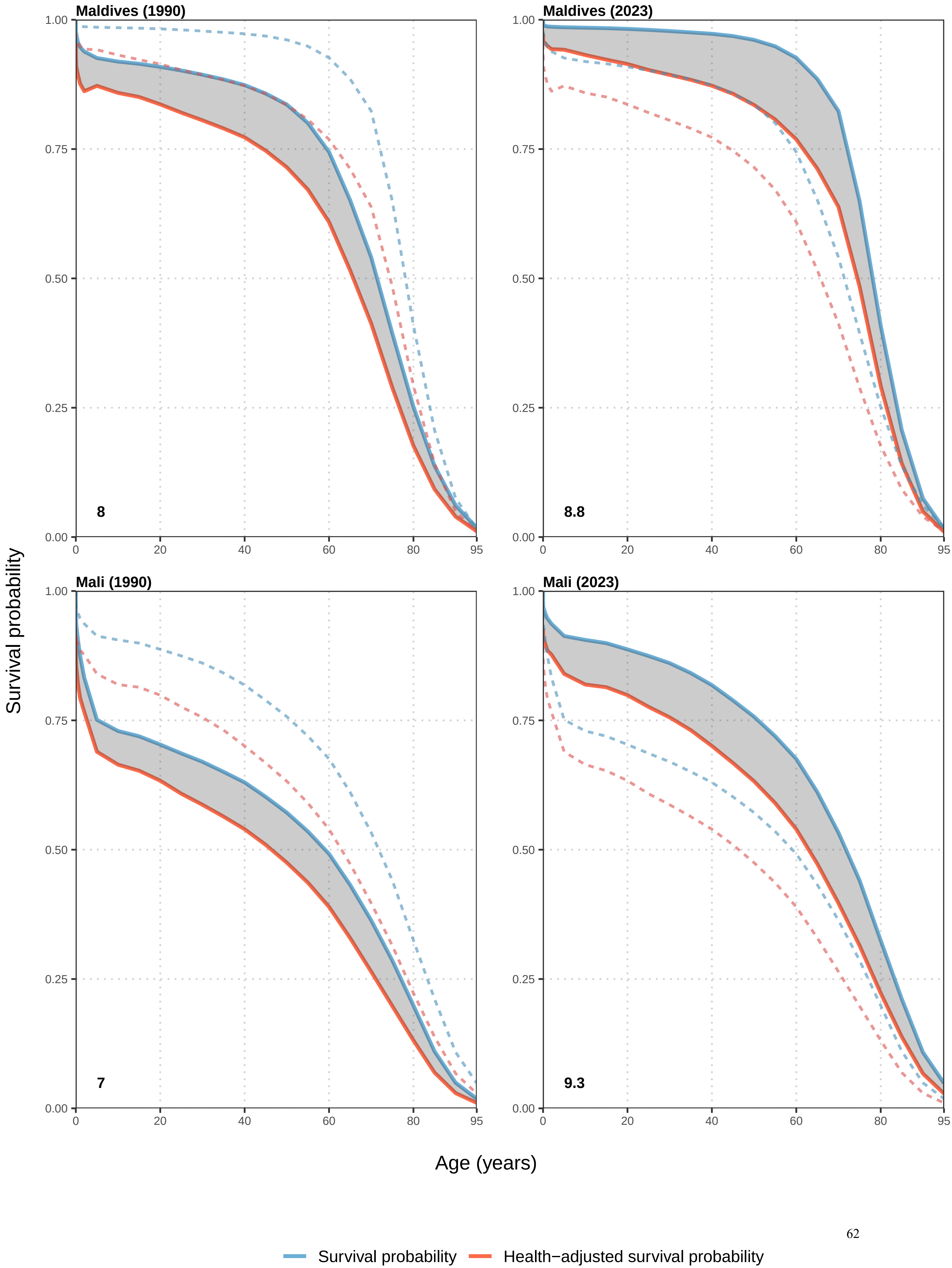

Maldives (1990)
Maldives (2023)
Mali (1990)
Mali (2023)
8
8.8
7
9.3
Survival probability
Age (years)
1.00
0.75
0.50
0.25
0.00
0
20
40
60
80
95
Survival probability
Health−adjusted survival probability

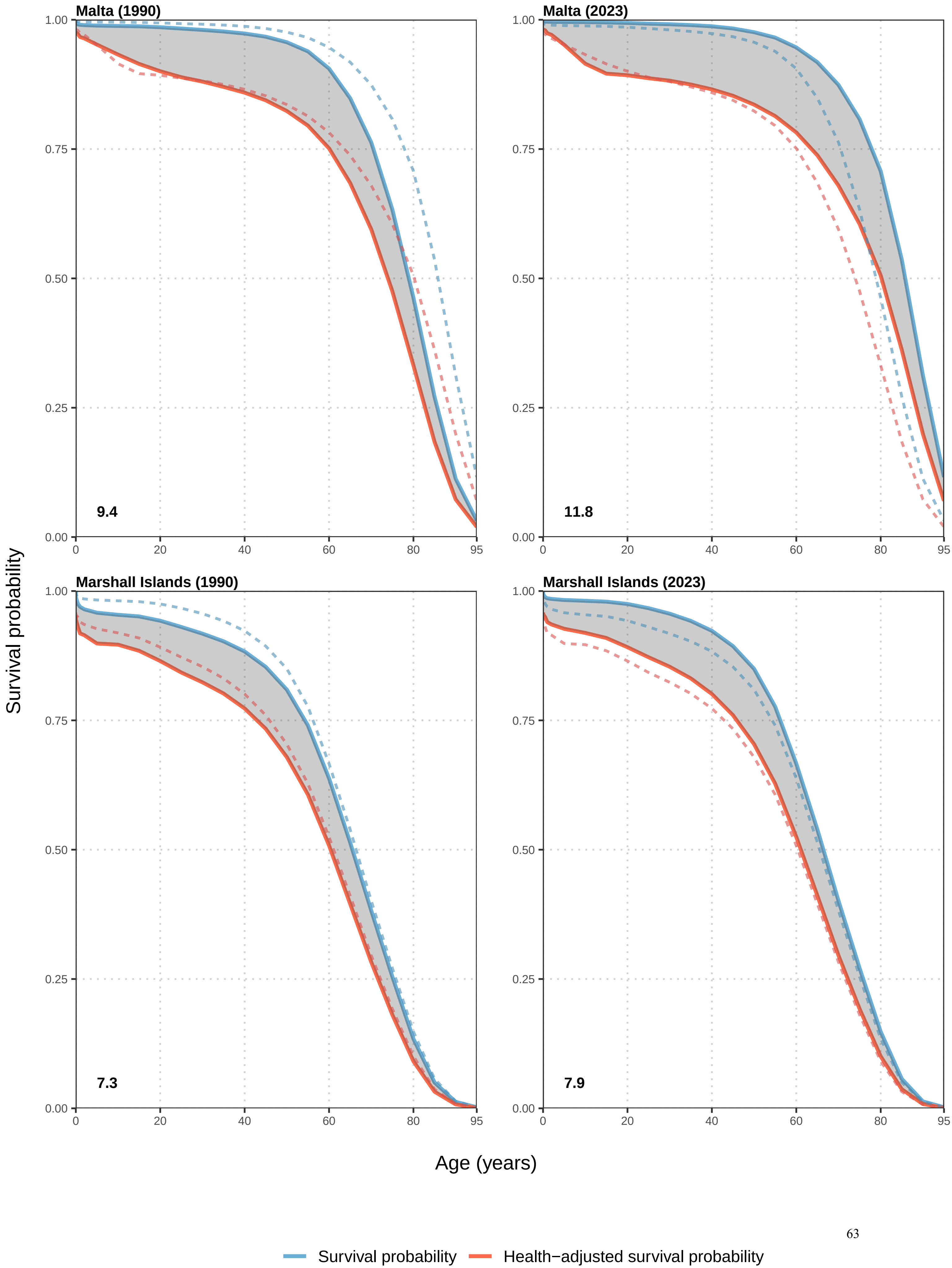

Malta (1990)
Malta (2023)
Marshall Islands (1990)
Marshall Islands (2023)
9.4
11.8
7.3
7.9
1.00
0.75
0.50
0.25
0.00
0
20
40
60
80
95
Survival probability
Age (years)
Survival probability
Health−adjusted survival probability

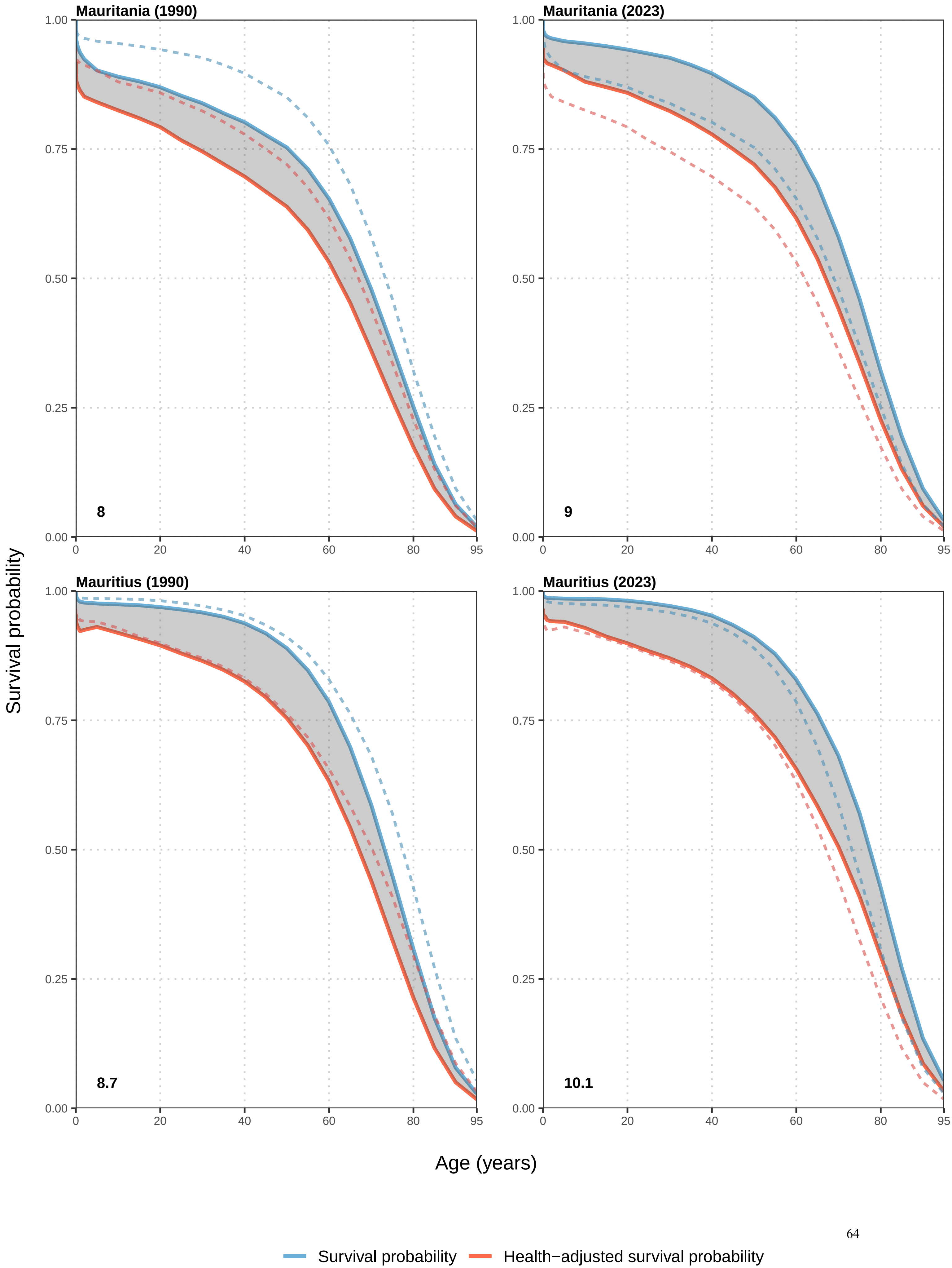
Mauritania (1990)
Mauritania (2023)
Mauritius (1990)
Mauritius (2023)
8
9
8.7
10.1
1.00
0.75
0.50
0.25
0.00
0
20
40
60
80
95
Survival probability
Age (years)
Survival probability
Health−adjusted survival probability

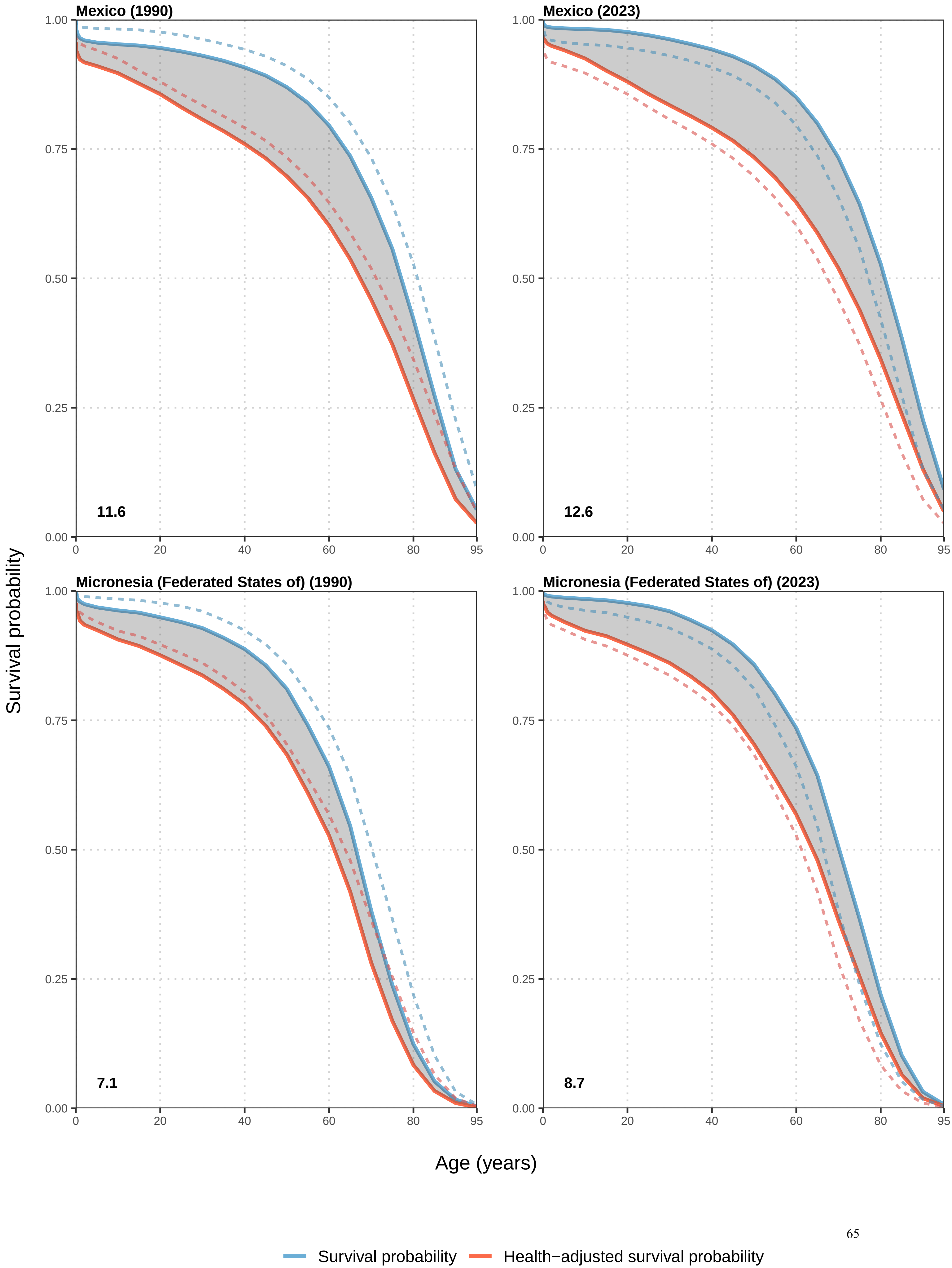
Mexico (1990)
Mexico (2023)
Micronesia (Federated States of) (1990)
Micronesia (Federated States of) (2023)
11.6
12.6
7.1
8.7
1.00
0.75
0.50
0.25
0.00
0
20
40
60
80
95
Survival probability
Age (years)
Survival probability
Health−adjusted survival probability

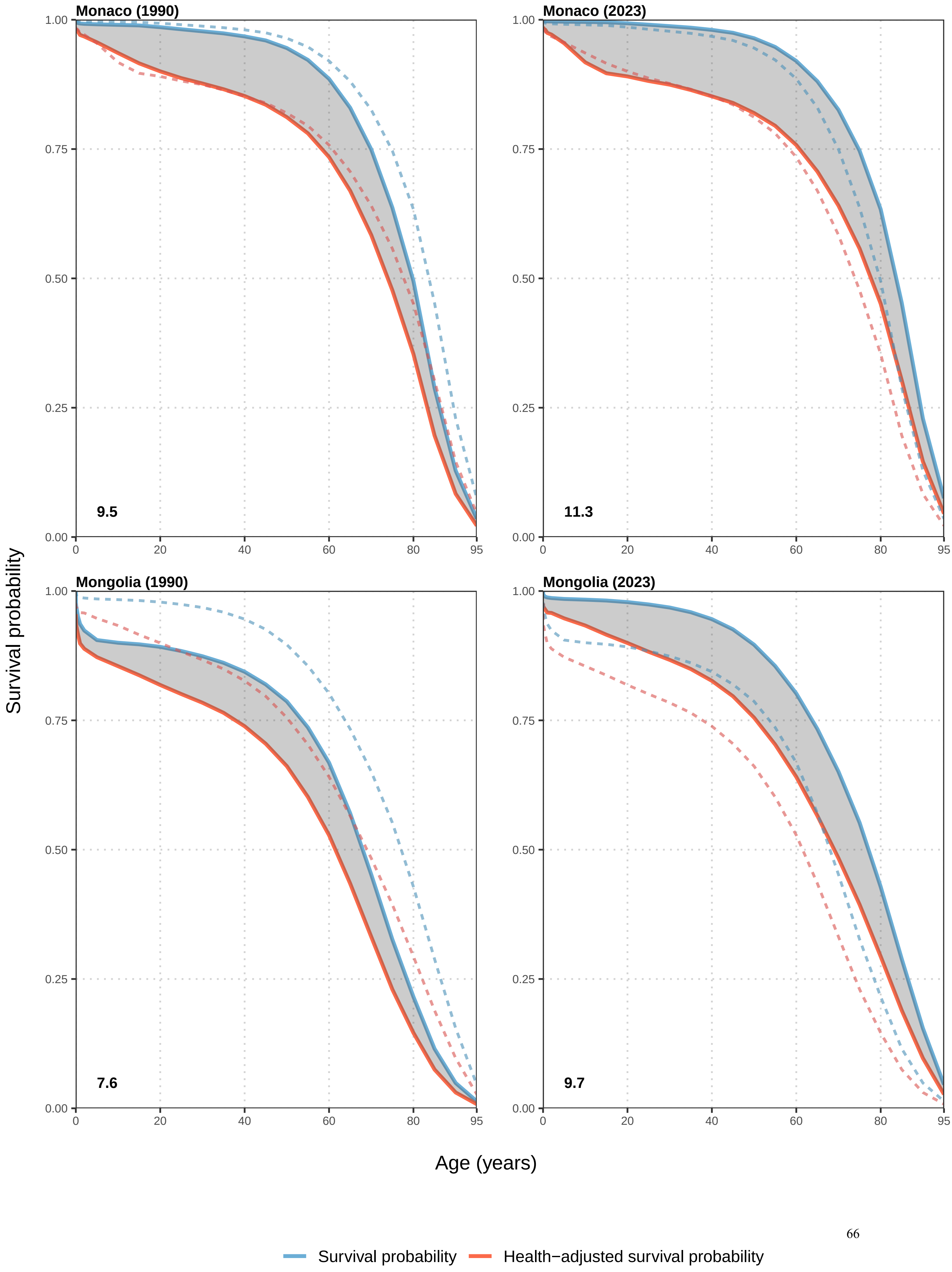

Monaco (1990)
Monaco (2023)
Mongolia (1990)
Mongolia (2023)
9.5
11.3
7.6
9.7
Survival probability
Age (years)
1.00
0.75
0.50
0.25
0.00
0
20
40
60
80
95
Survival probability
Health−adjusted survival probability

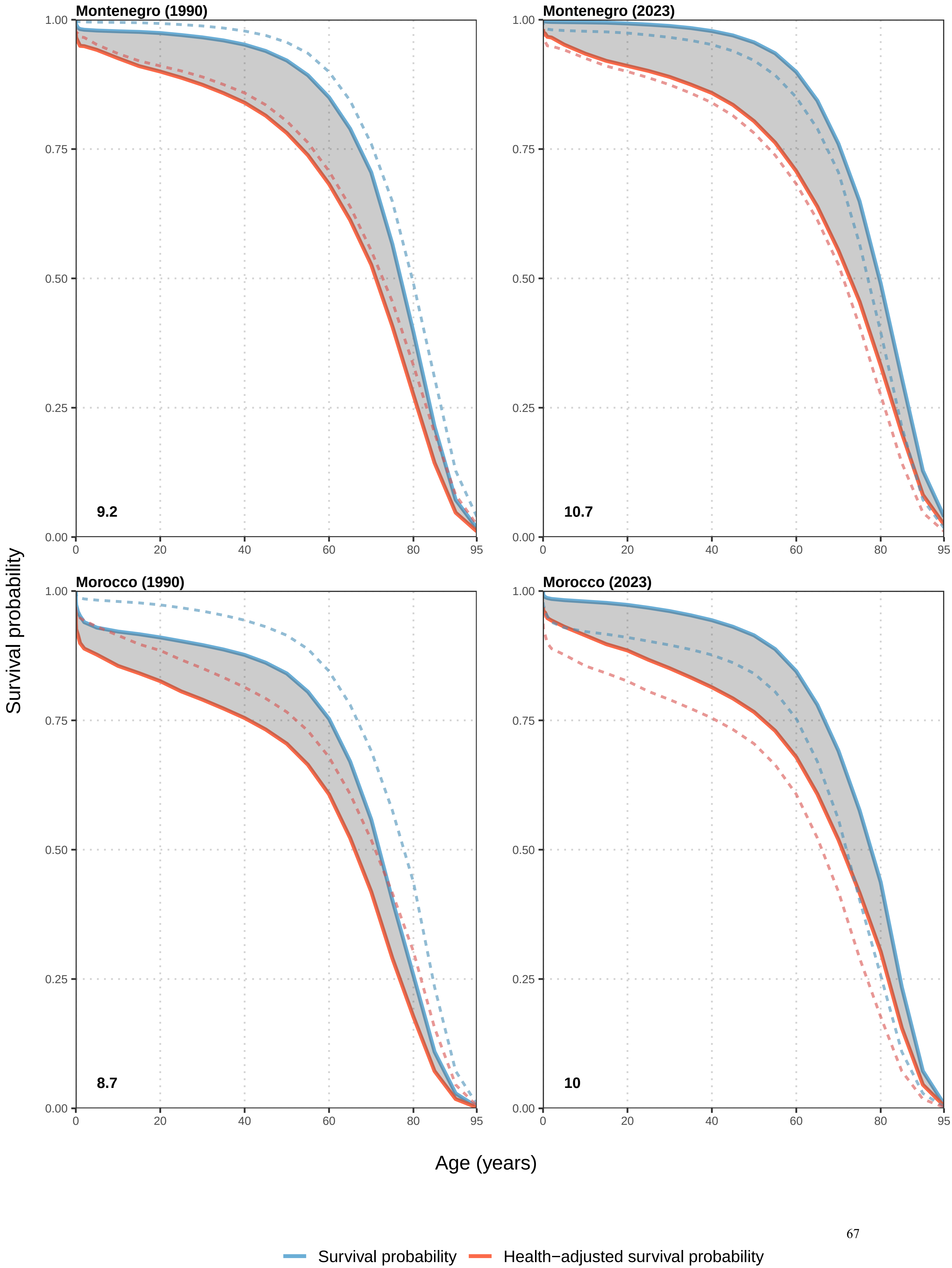

Montenegro (1990)
9.2
Montenegro (2023)
10.7
Morocco (1990)
8.7
Morocco (2023)
10
Survival probability
Age (years)
1.00
0.75
0.50
0.25
0.00
0
20
40
60
80
95
Survival probability
Health−adjusted survival probability

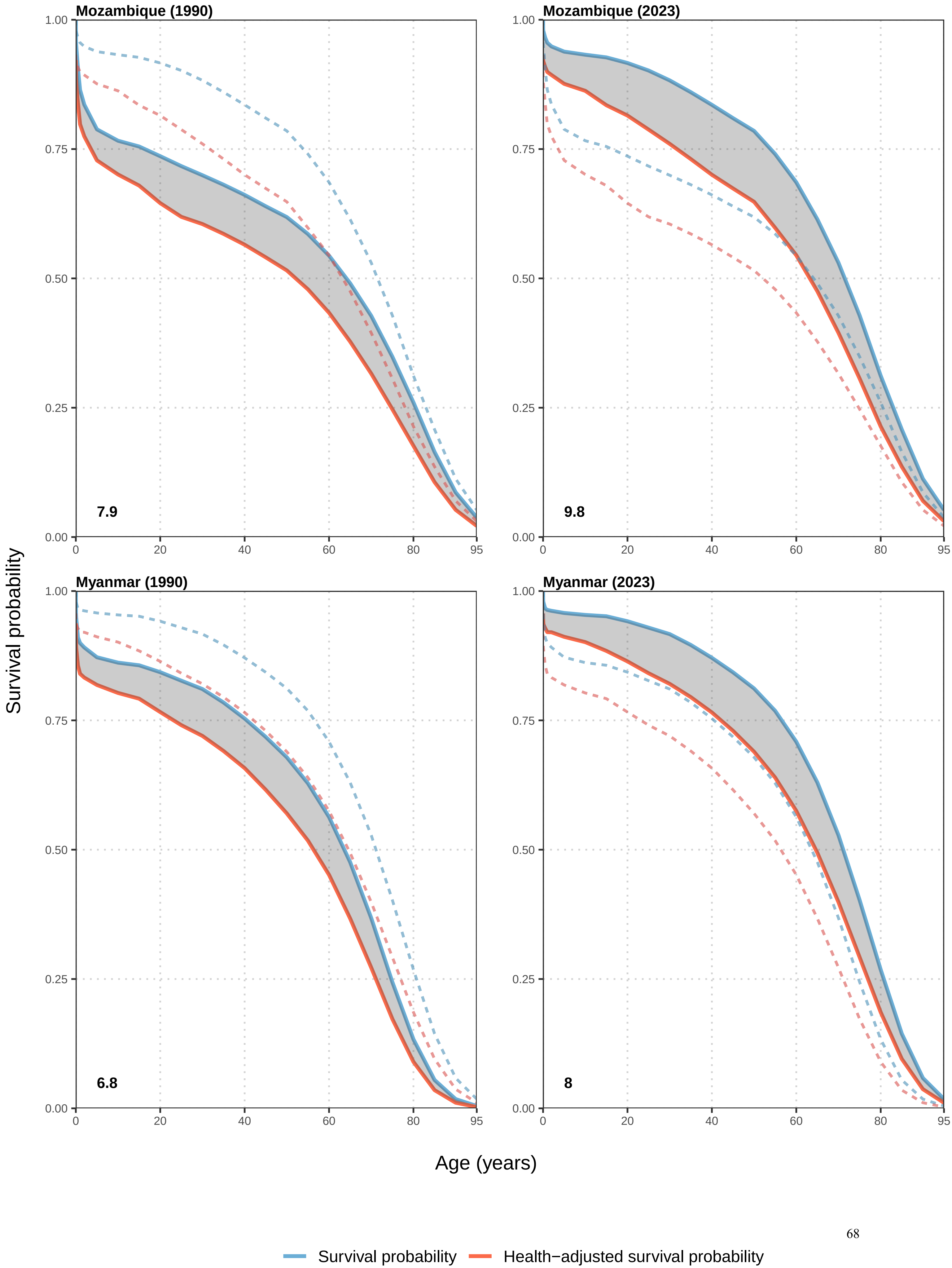
Mozambique (1990)
Mozambique (2023)
Myanmar (1990)
Myanmar (2023)
7.9
9.8
6.8
8
Survival probability
Age (years)
Survival probability
Health−adjusted survival probability

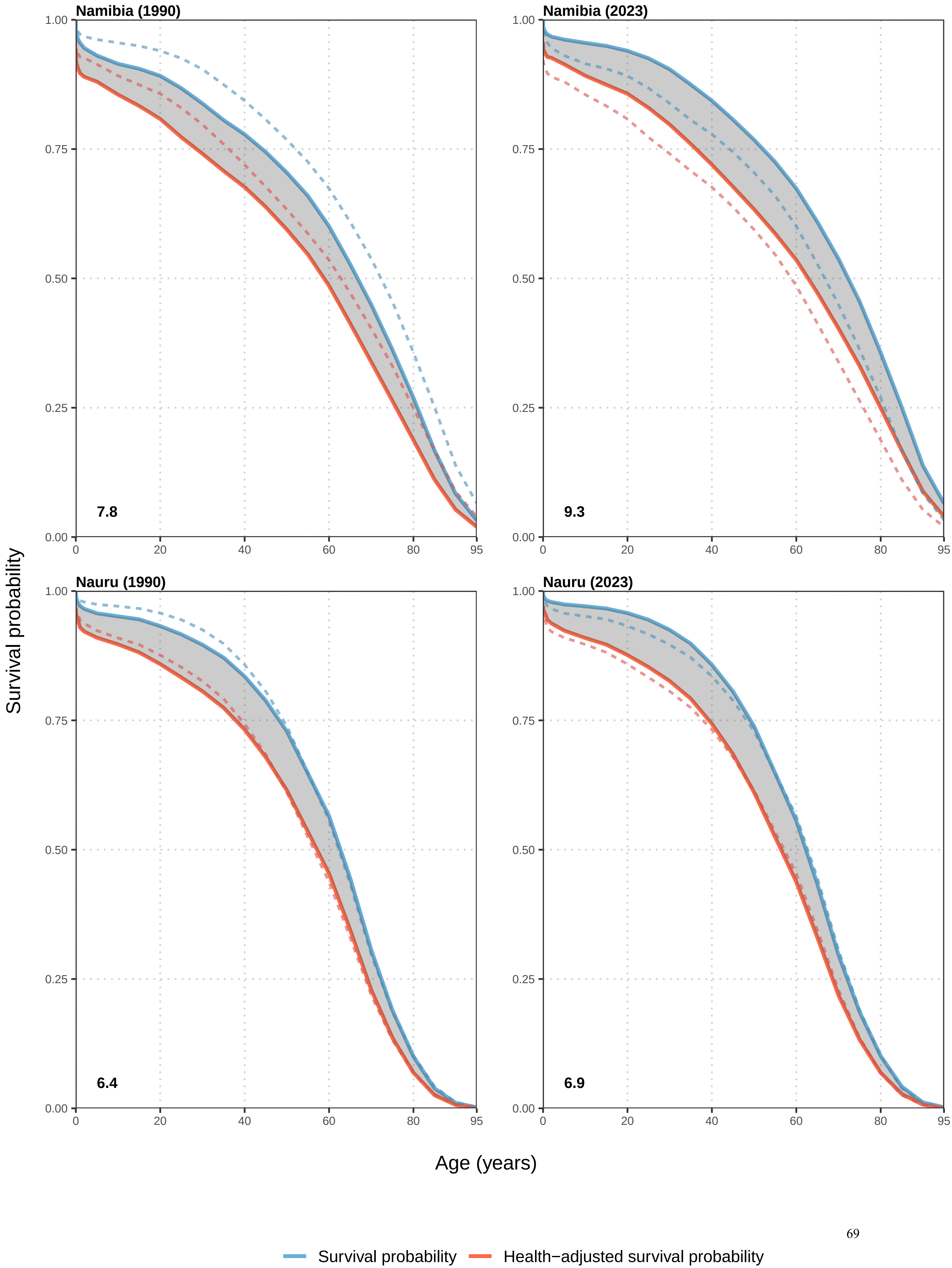
Namibia (1990)
Namibia (2023)
Nauru (1990)
Nauru (2023)
7.8
9.3
6.4
6.9
1.00
0.75
0.50
0.25
0.00
0
20
40
60
80
95
Survival probability
Age (years)
Survival probability
Health−adjusted survival probability

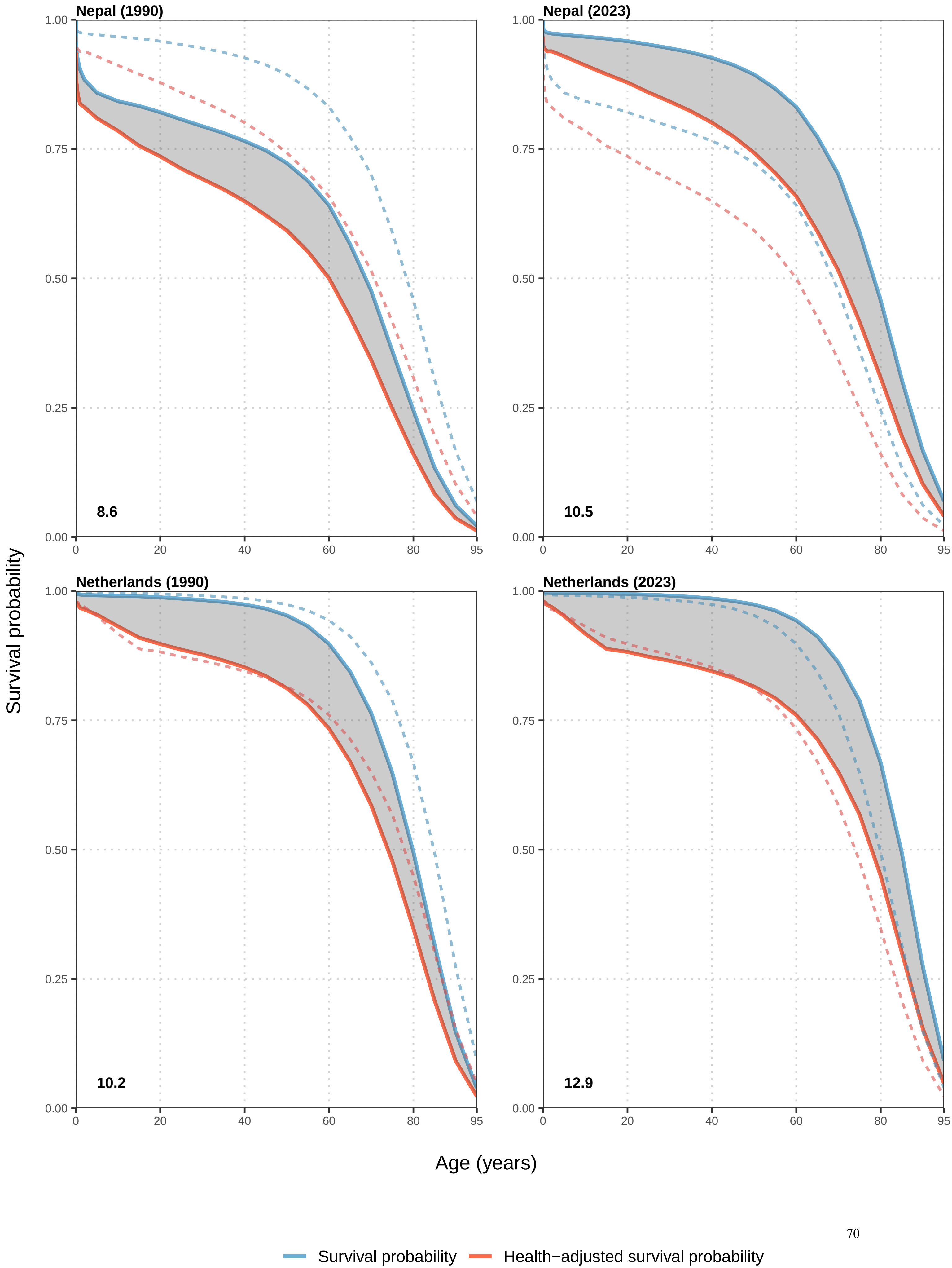

Nepal (1990)
Nepal (2023)
Netherlands (1990)
Netherlands (2023)
8.6
10.5
10.2
12.9
Survival probability
Age (years)
1.00
0.75
0.50
0.25
0.00
0
20
40
60
80
95
Survival probability
Health−adjusted survival probability

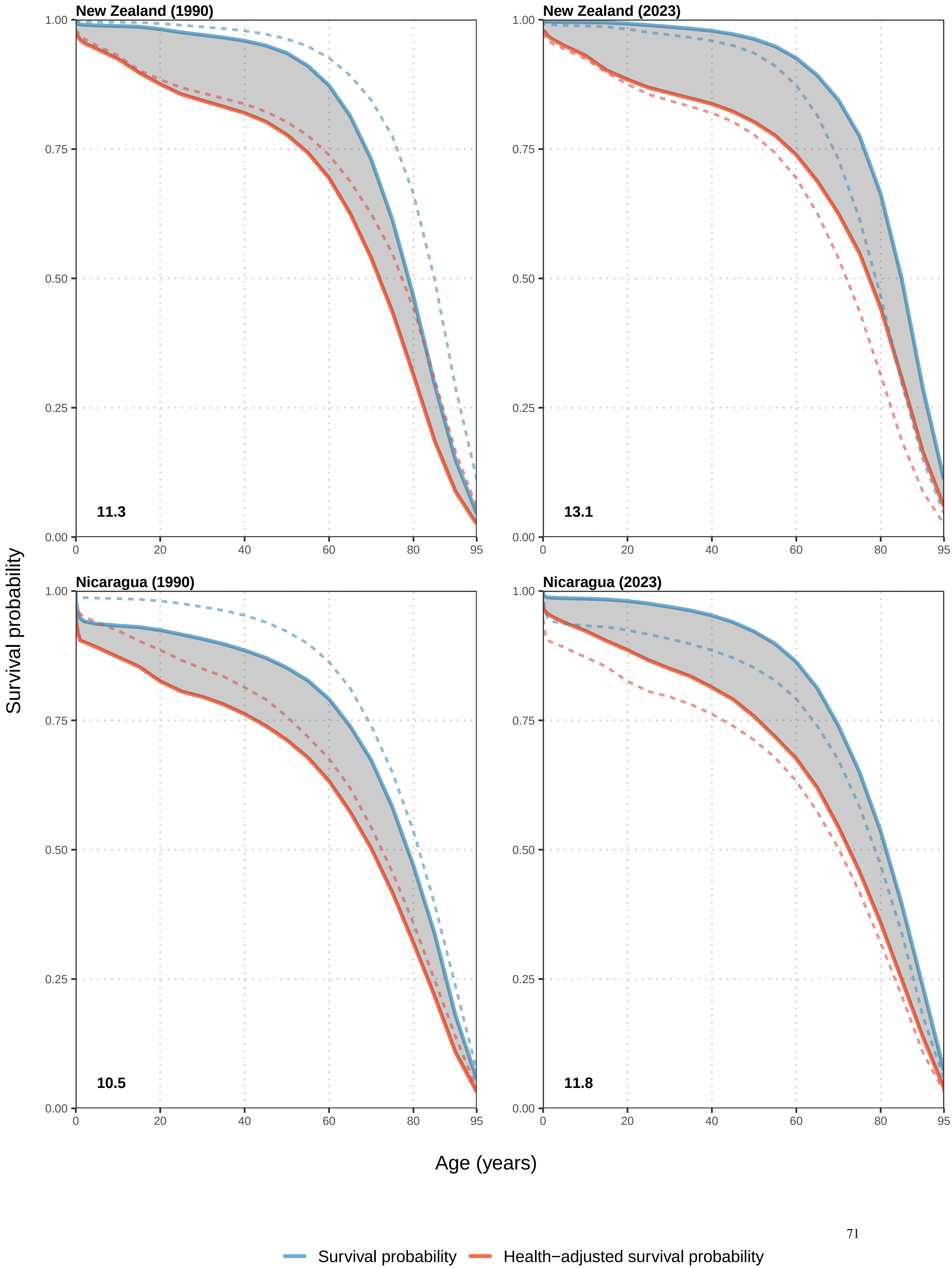

New Zealand (1990)
New Zealand (2023)
Nicaragua (1990)
Nicaragua (2023)
11.3
13.1
10.5
11.8
1.00
0.75
0.50
0.25
0.00
0
20
40
60
80
95
Survival probability
Age (years)
Survival probability
Health−adjusted survival probability

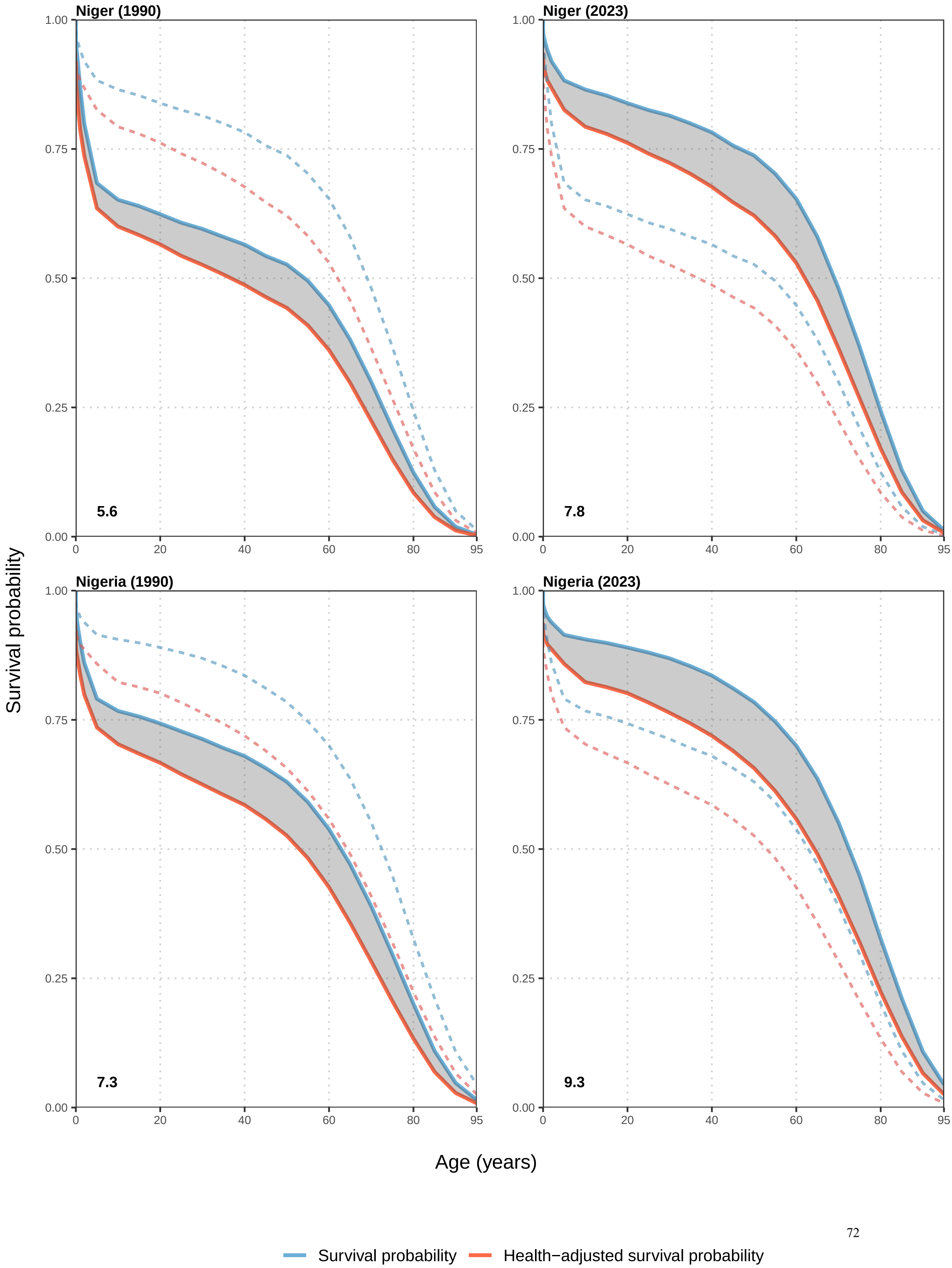

Niger (1990)
Niger (2023)
Nigeria (1990)
Nigeria (2023)
5.6
7.8
7.3
9.3
1.00
0.75
0.50
0.25
0.00
0
20
40
60
80
95
Survival probability
Age (years)
Survival probability
Health−adjusted survival probability

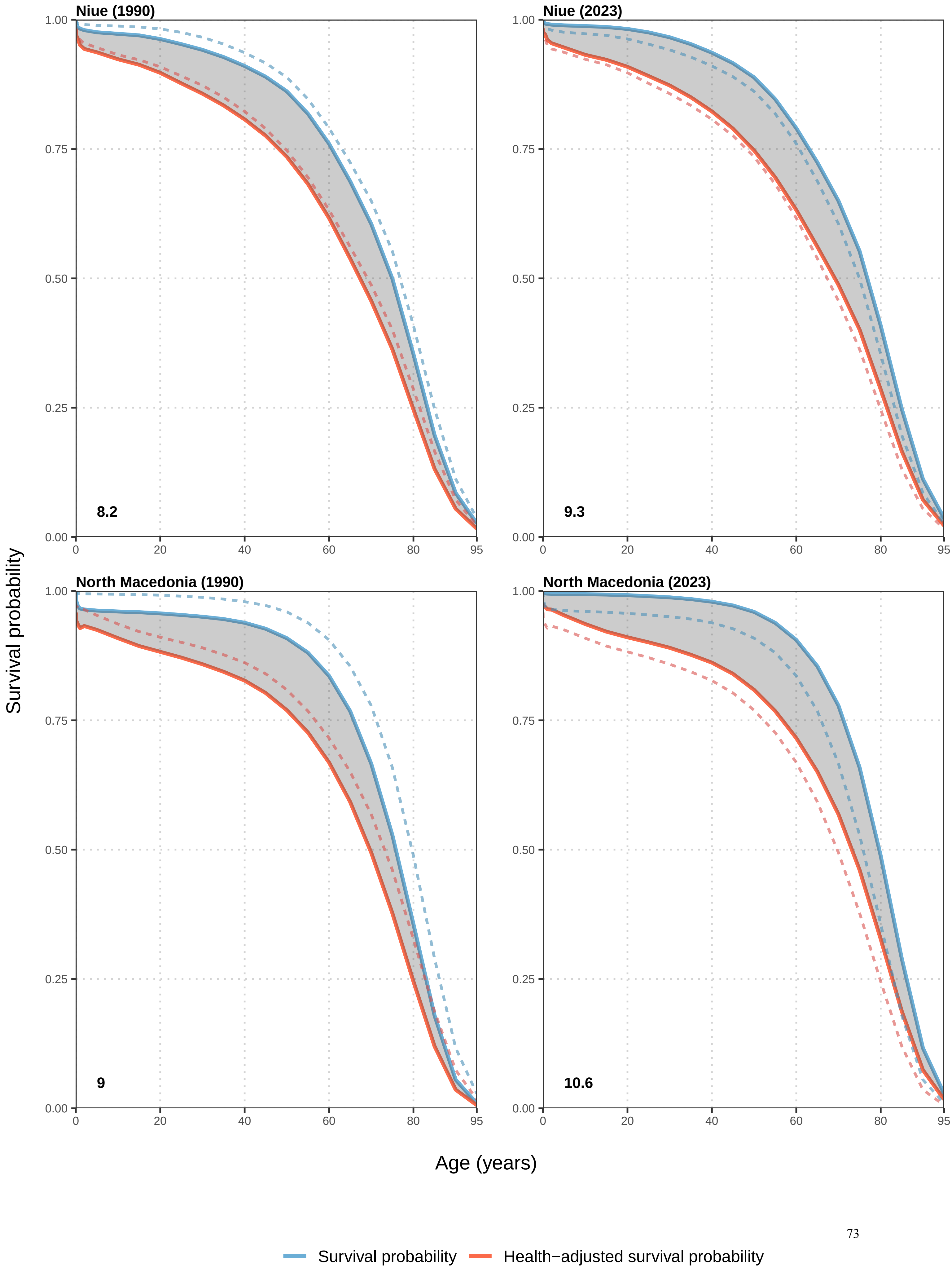
Niue (1990)
8.2
Niue (2023)
9.3
North Macedonia (1990)
9
North Macedonia (2023)
10.6
Survival probability
Age (years)
0.00
0.25
0.50
0.75
1.00
0
20
40
60
80
95
Survival probability
Health−adjusted survival probability

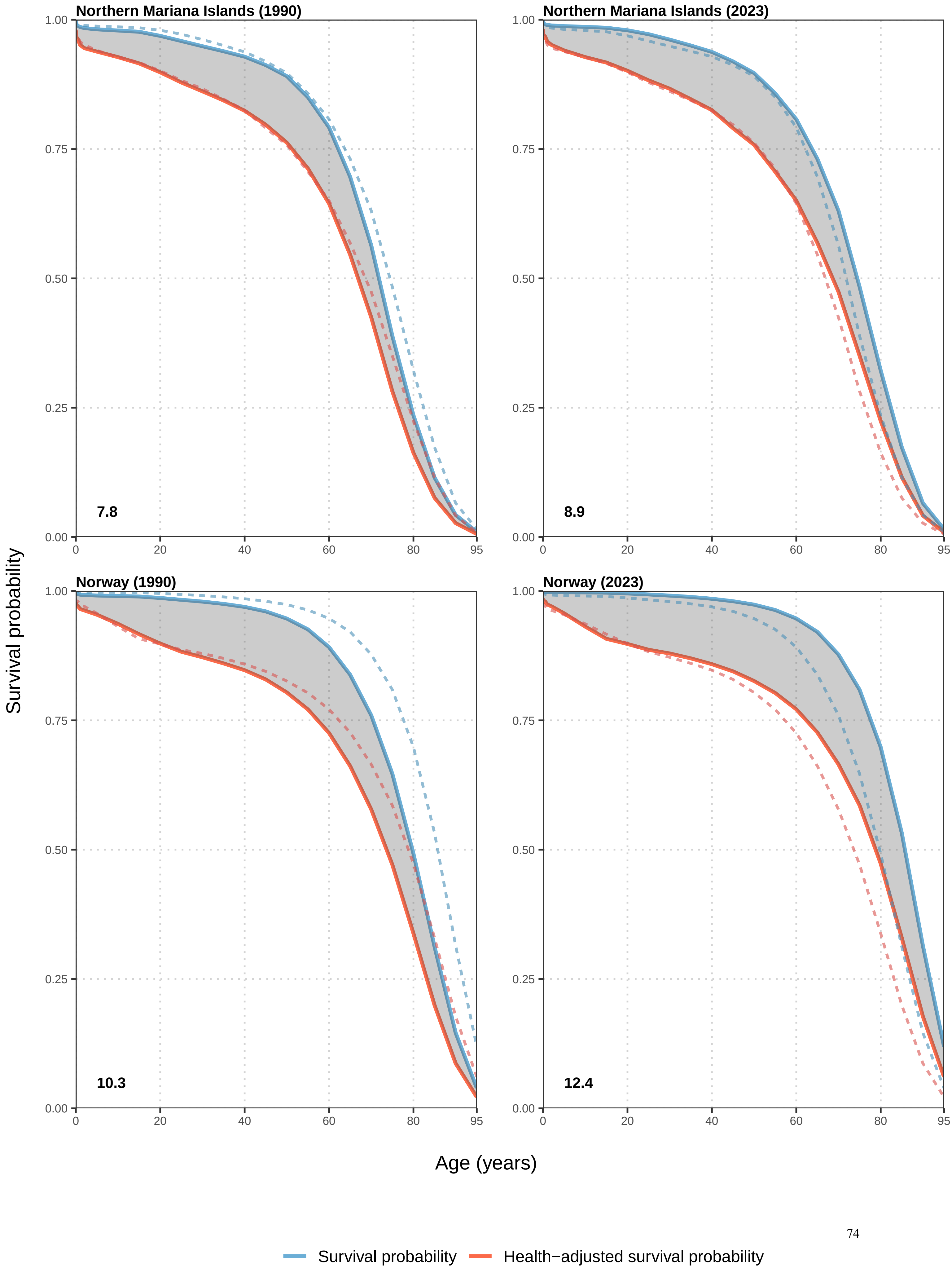

Northern Mariana Islands (1990)
Northern Mariana Islands (2023)
Norway (1990)
Norway (2023)
7.8
8.9
10.3
12.4
Survival probability
Age (years)
Survival probability
Health−adjusted survival probability

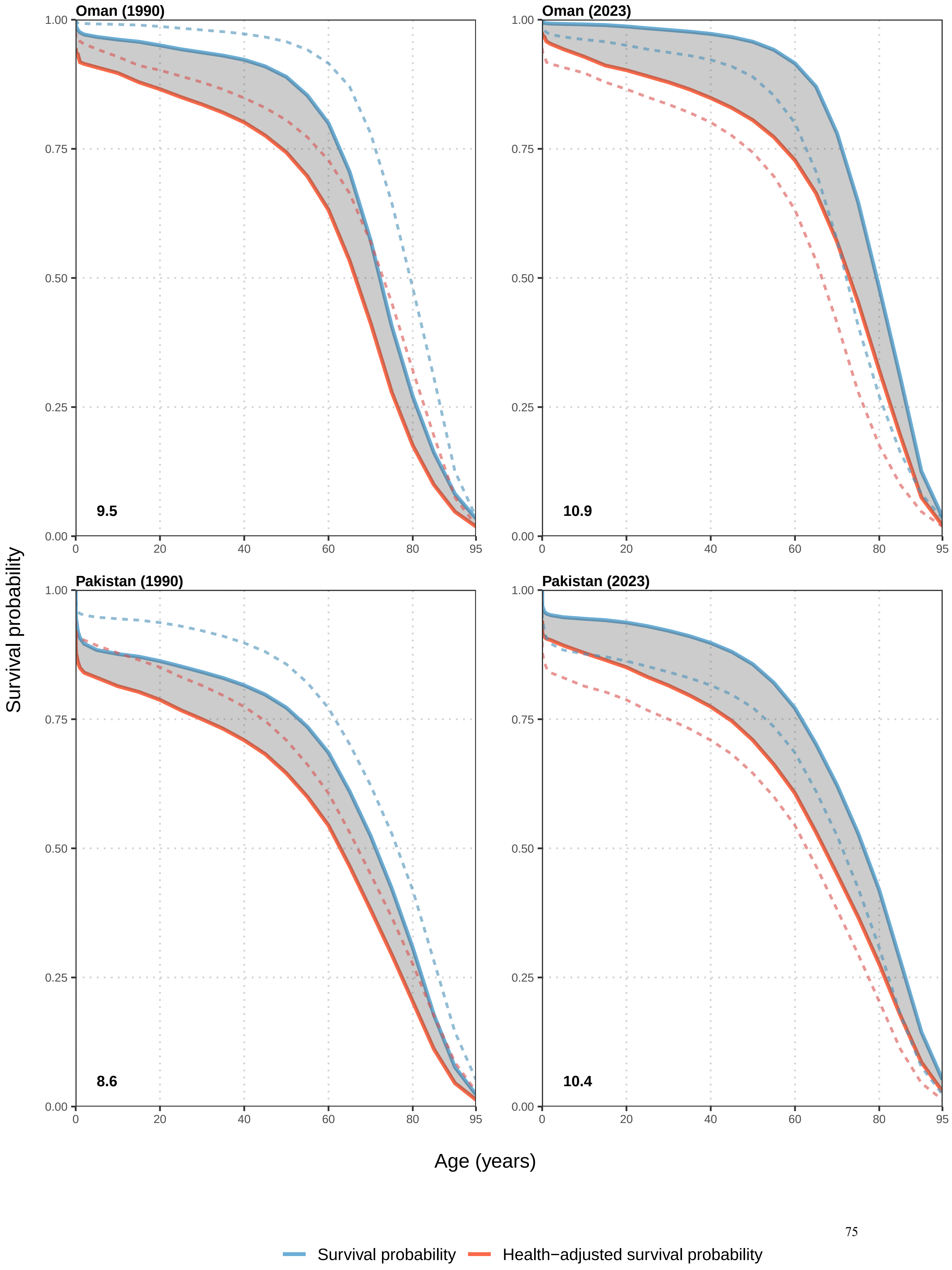

Oman (1990)
Oman (2023)
Pakistan (1990)
Pakistan (2023)
9.5
10.9
8.6
10.4
Survival probability
Age (years)
1.00
0.75
0.50
0.25
0.00
0
20
40
60
80
95
Survival probability
Health−adjusted survival probability

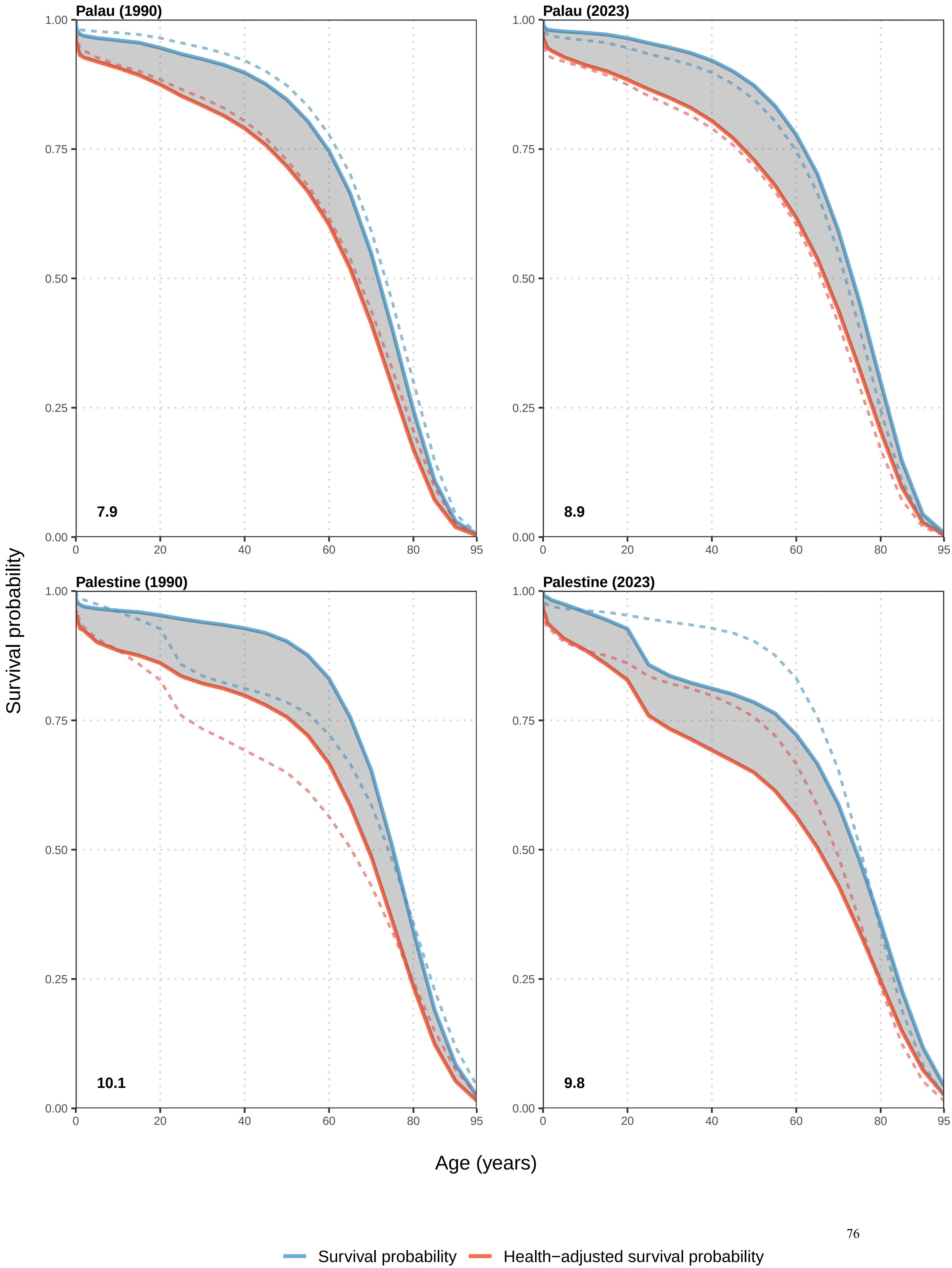
Palau (1990)
Palau (2023)
Palestine (1990)
Palestine (2023)
7.9
8.9
10.1
9.8
1.00
0.75
0.50
0.25
0.00
0
20
40
60
80
95
Survival probability
Age (years)
Survival probability
Health−adjusted survival probability

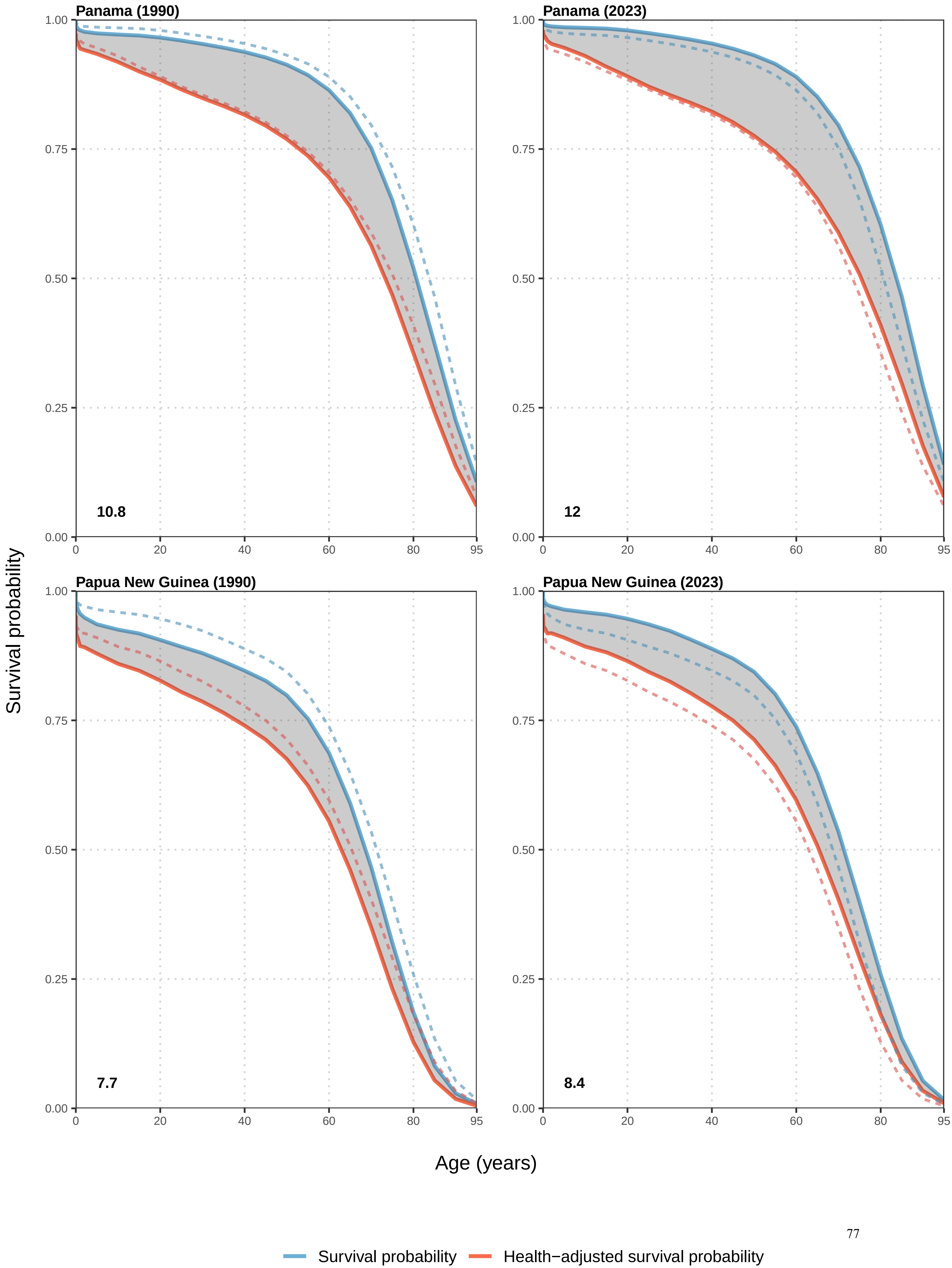
Panama (1990)
10.8
Panama (2023)
12
Papua New Guinea (1990)
7.7
Papua New Guinea (2023)
8.4
Survival probability
Age (years)
1.00
0.75
0.50
0.25
0.00
0
20
40
60
80
95
Survival probability
Health−adjusted survival probability

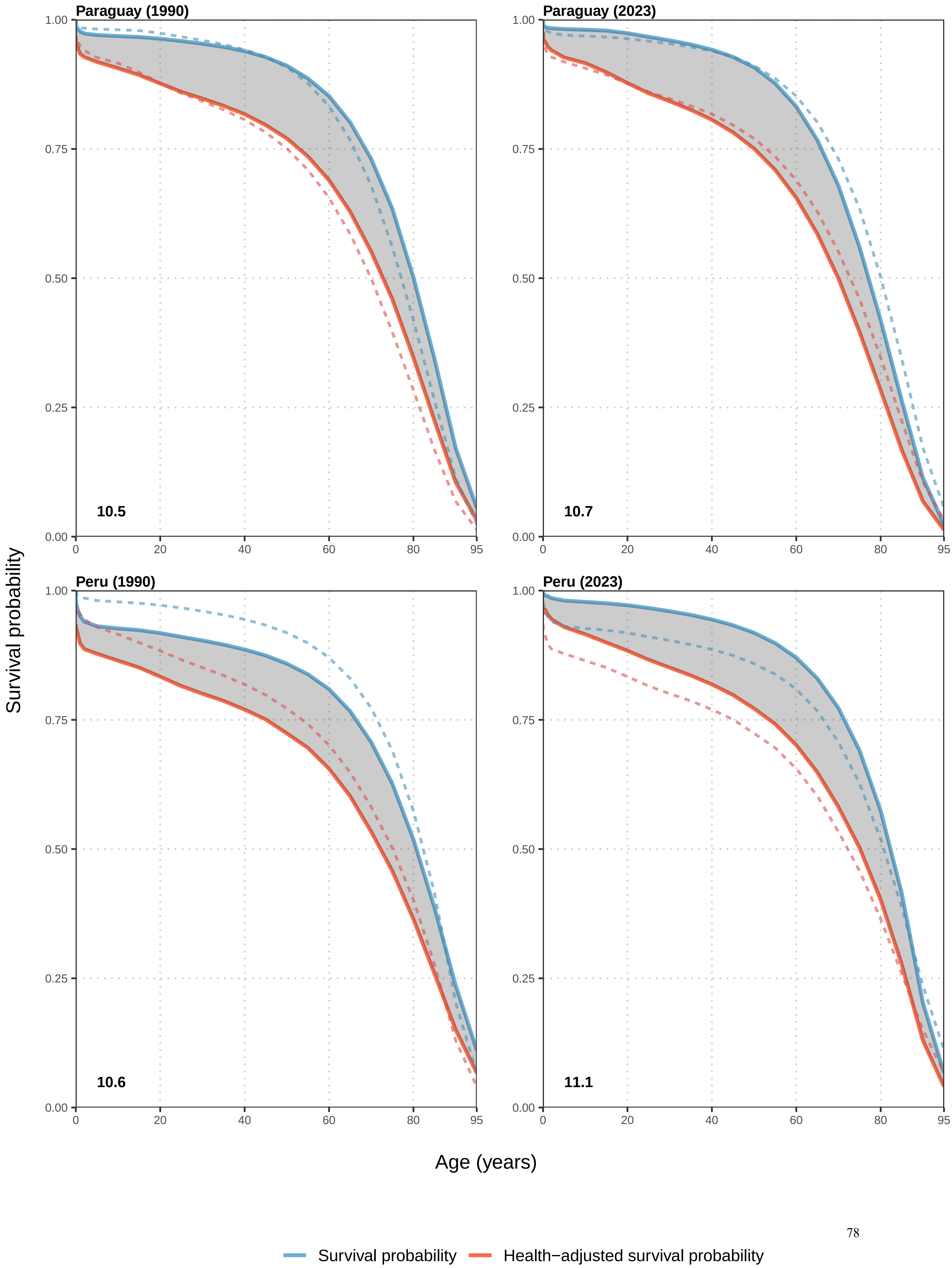
Paraguay (1990)
Paraguay (2023)
Peru (1990)
Peru (2023)
10.5
10.7
10.6
11.1
Survival probability
Age (years)
Survival probability
Health−adjusted survival probability

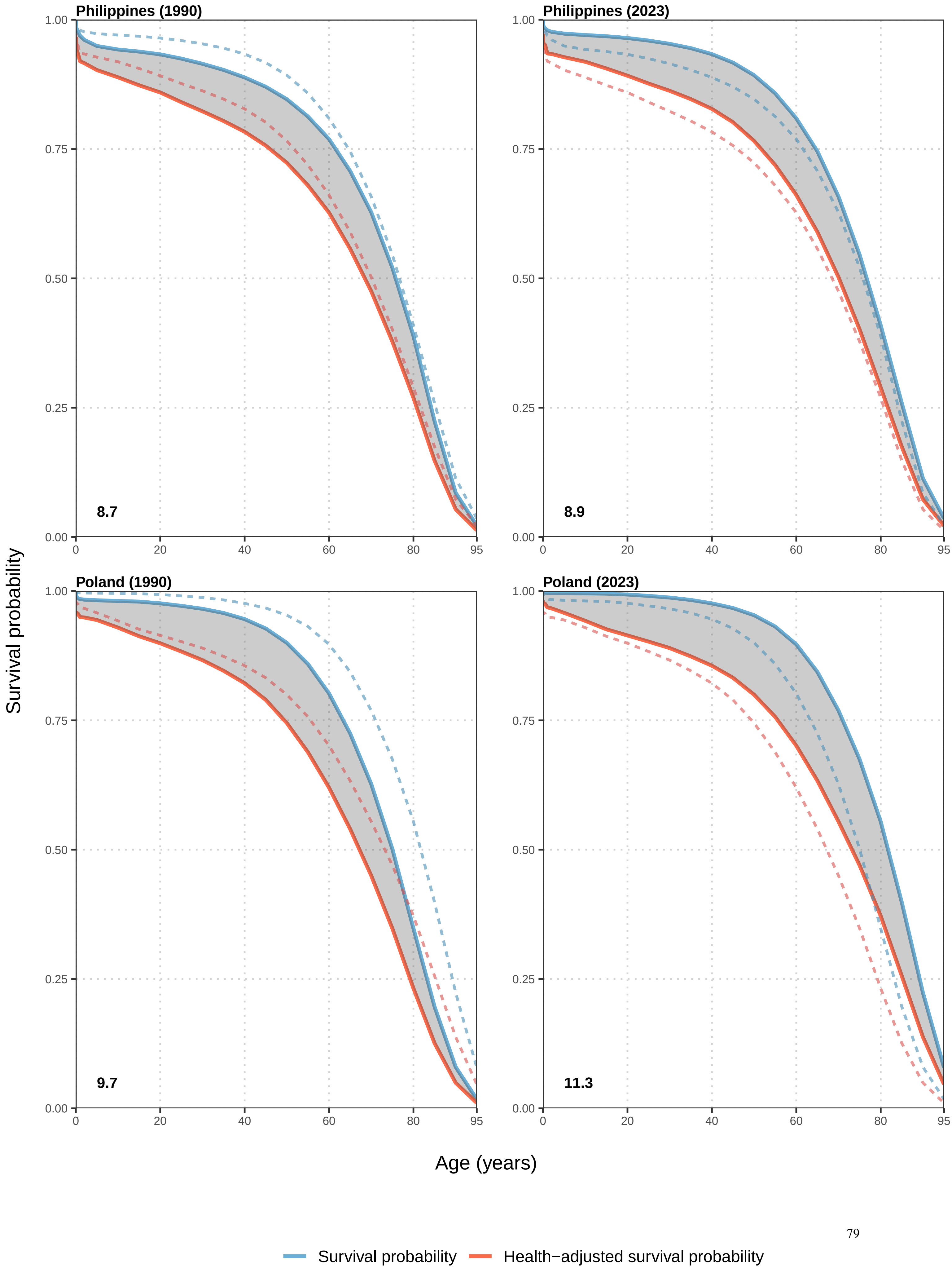
Philippines (1990)
Philippines (2023)
Poland (1990)
Poland (2023)
8.7
8.9
9.7
11.3
1.00
0.75
0.50
0.25
0.00
0
20
40
60
80
95
Survival probability
Age (years)
Survival probability
Health−adjusted survival probability

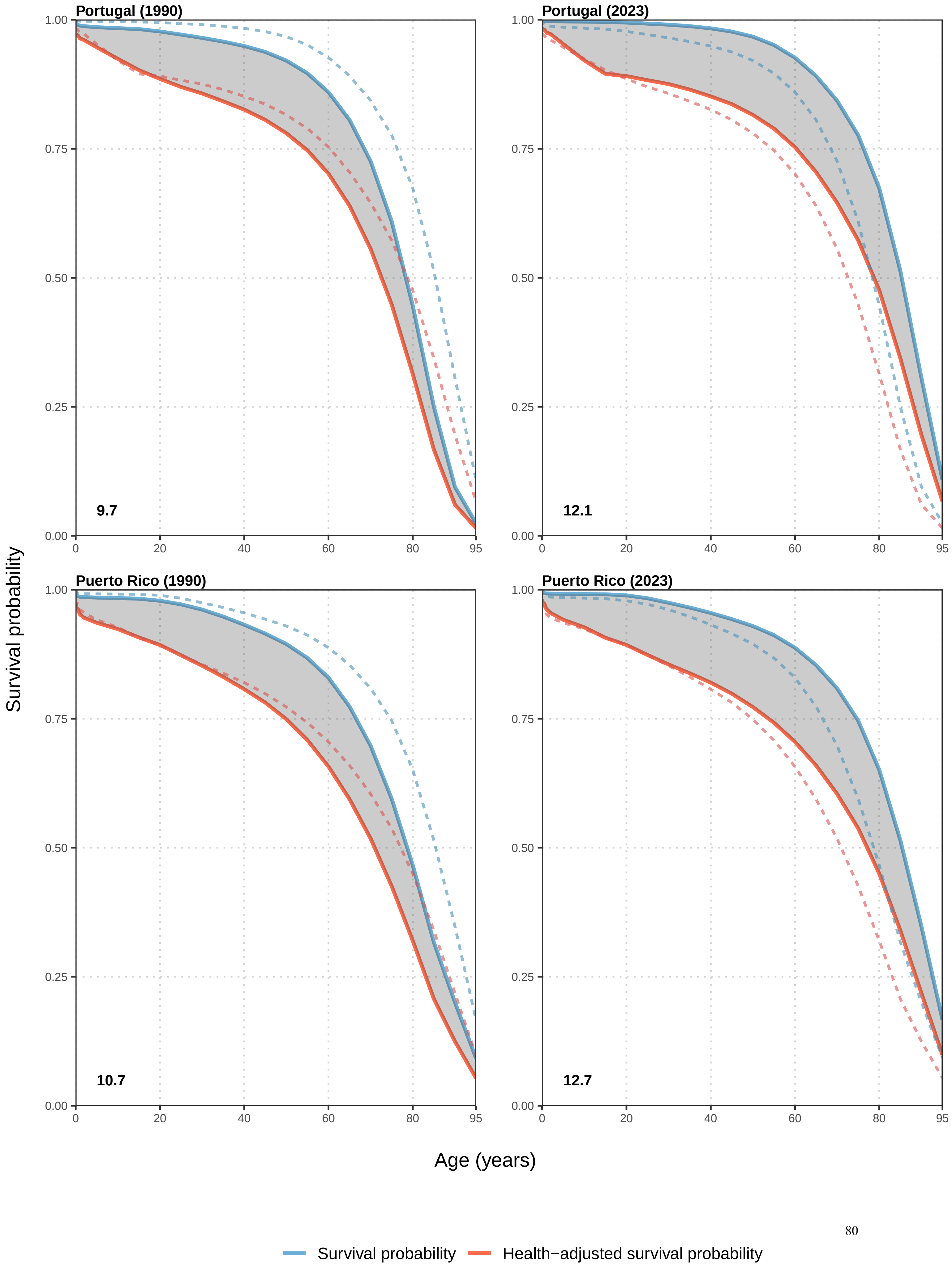

Portugal (1990)
9.7
Portugal (2023)
12.1
Puerto Rico (1990)
10.7
Puerto Rico (2023)
12.7
Survival probability
Age (years)
0.00
0.25
0.50
0.75
1.00
0
20
40
60
80
95
Survival probability
Health−adjusted survival probability

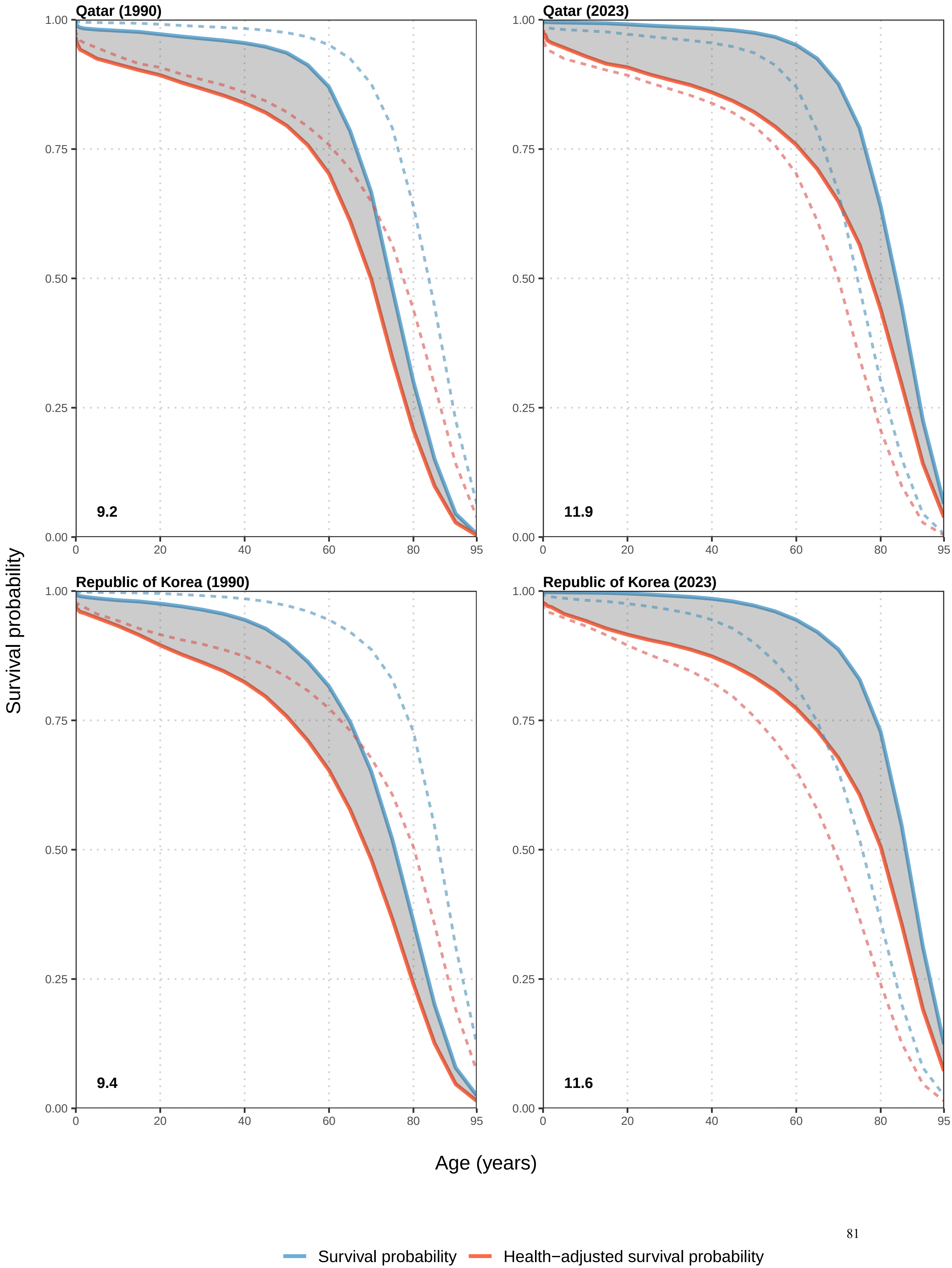
Qatar (1990)
9.2
Qatar (2023)
11.9
Republic of Korea (1990)
9.4
Republic of Korea (2023)
11.6
Survival probability
Age (years)
1.00
0.75
0.50
0.25
0.00
0
20
40
60
80
95
Survival probability
Health−adjusted survival probability

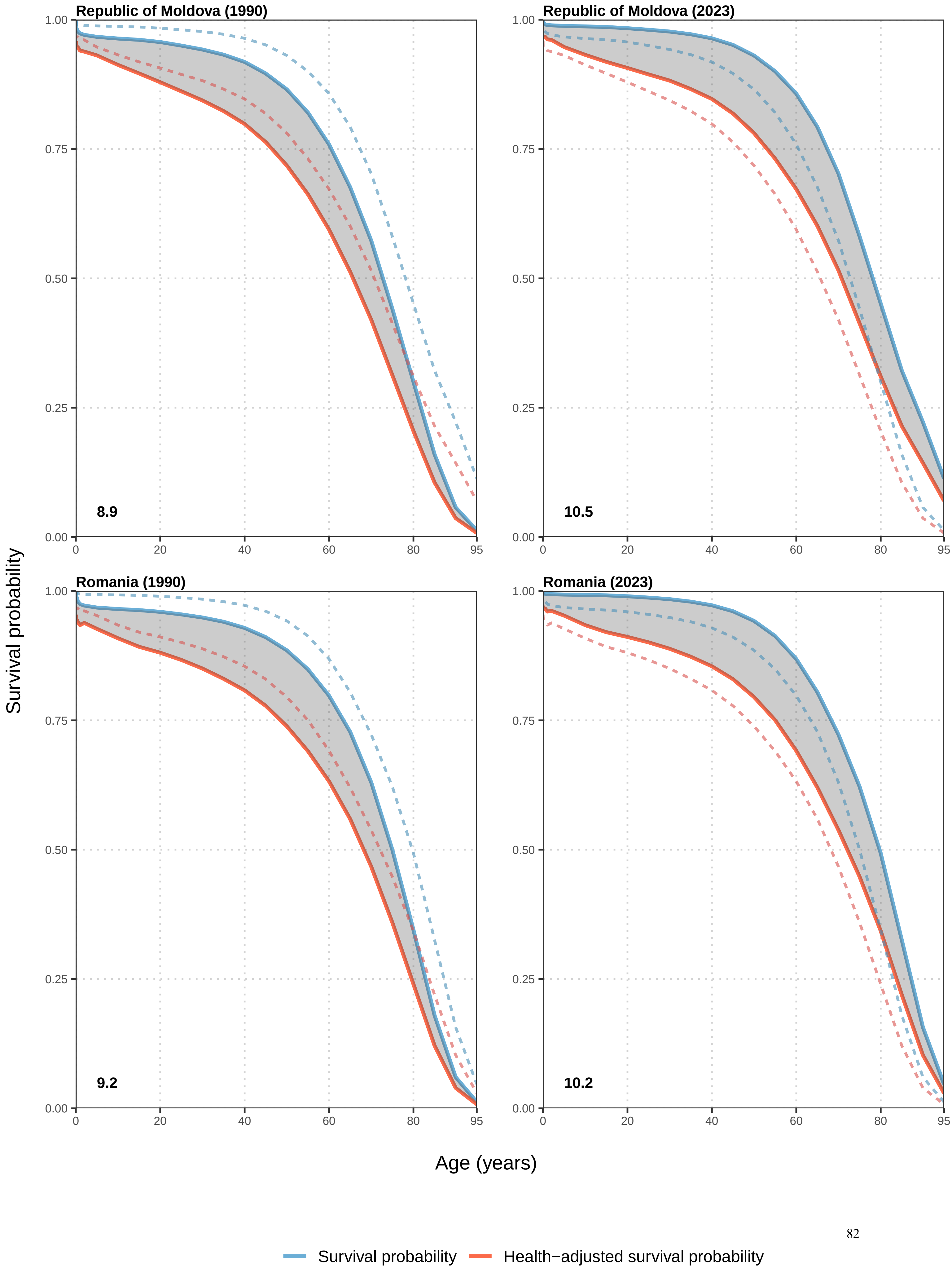

Republic of Moldova (1990)
Republic of Moldova (2023)
Romania (1990)
Romania (2023)
8.9
10.5
9.2
10.2
1.00
0.75
0.50
0.25
0.00
0
20
40
60
80
95
Survival probability
Age (years)
Survival probability
Health−adjusted survival probability

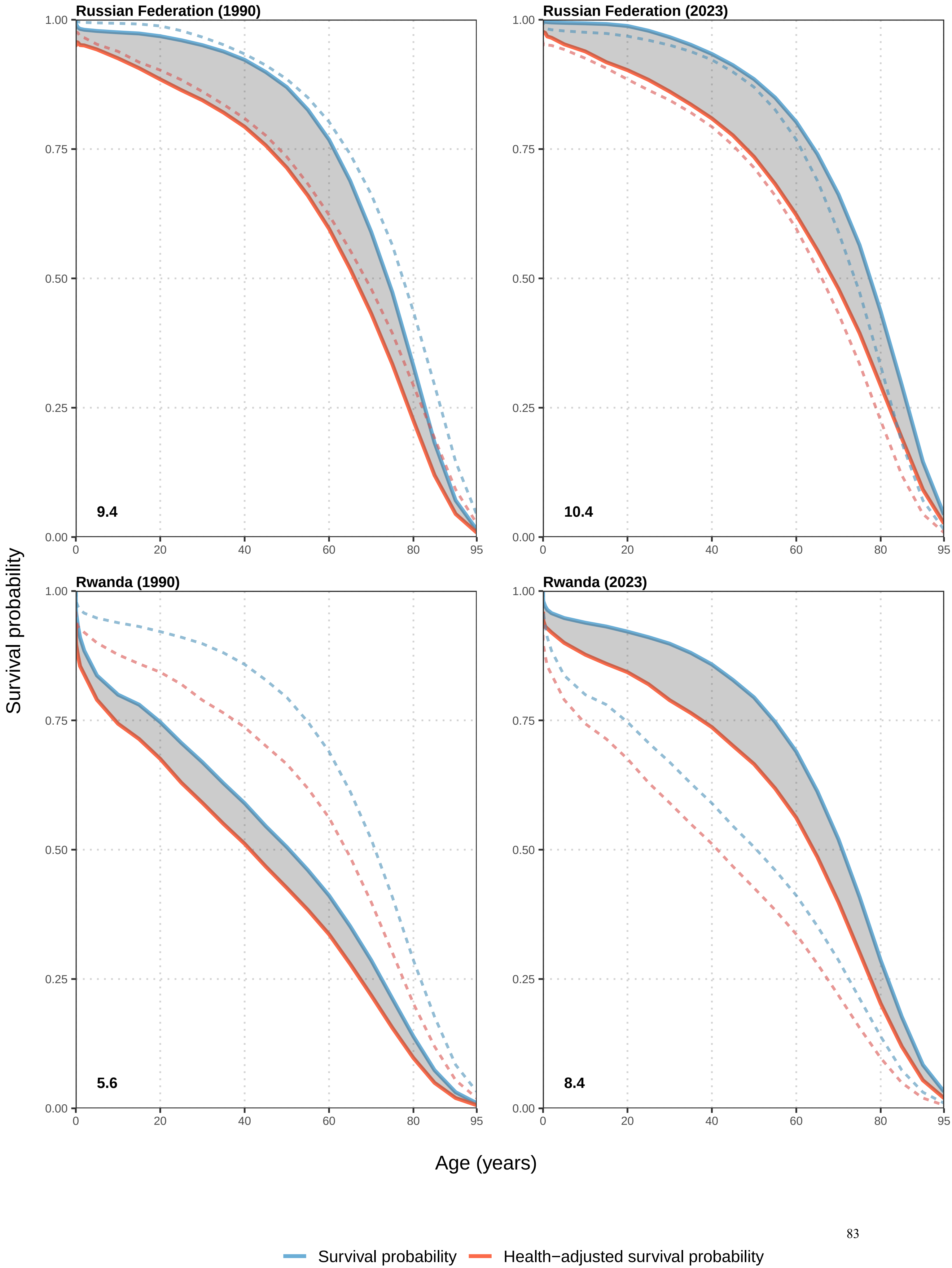

Russian Federation (1990)
9.4
Russian Federation (2023)
10.4
Rwanda (1990)
5.6
Rwanda (2023)
8.4
1.00
0.75
0.50
0.25
0.00
0
20
40
60
80
95
Survival probability
Age (years)

Survival probability
Health−adjusted survival probability

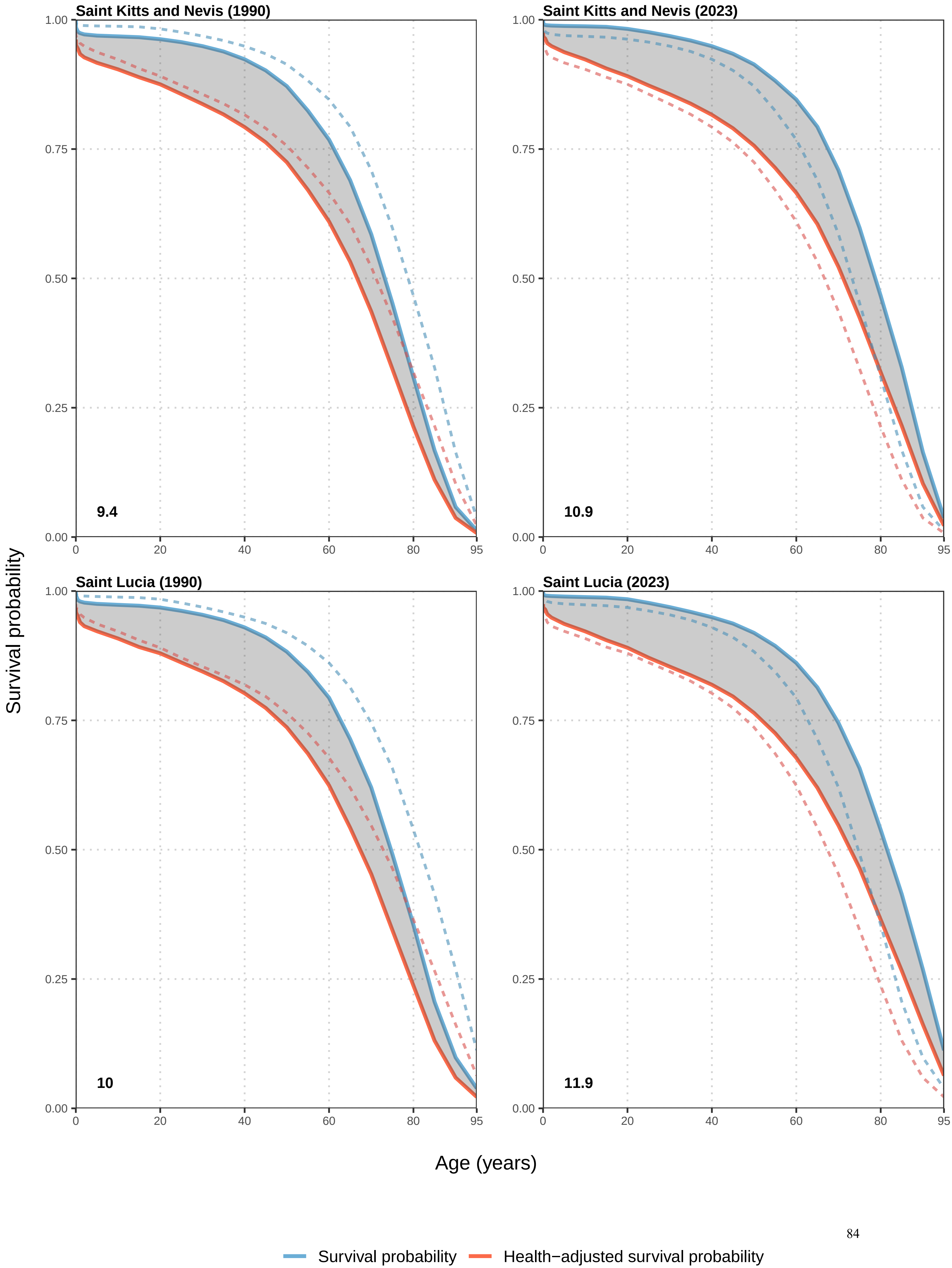
Saint Kitts and Nevis (1990)
Saint Kitts and Nevis (2023)
Saint Lucia (1990)
Saint Lucia (2023)
9.4
10.9
10
11.9
Survival probability
Age (years)
Survival probability
Health−adjusted survival probability

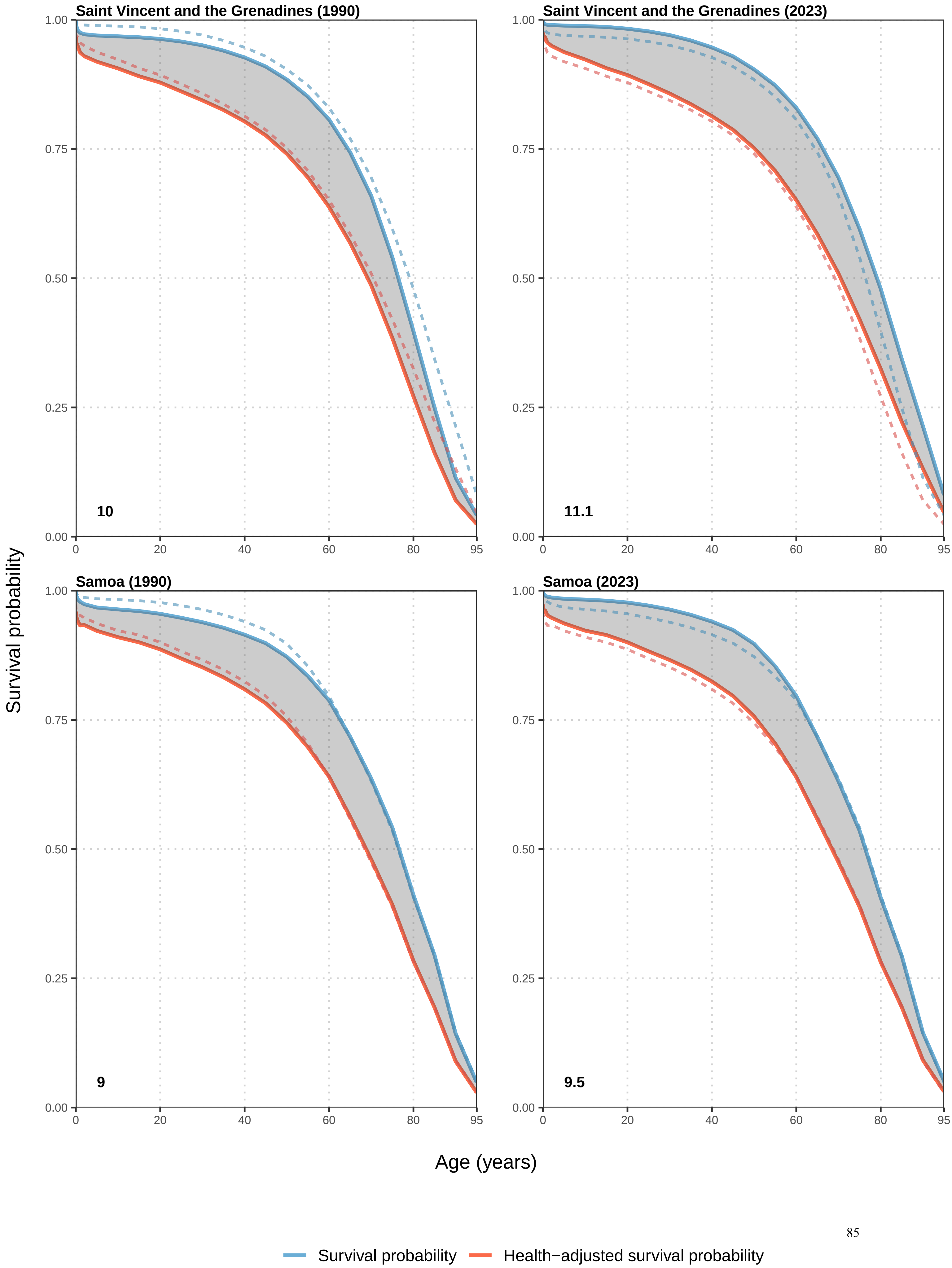
Saint Vincent and the Grenadines (1990)
Saint Vincent and the Grenadines (2023)
Samoa (1990)
Samoa (2023)
10
11.1
9
9.5
Survival probability
Age (years)
Survival probability
Health−adjusted survival probability

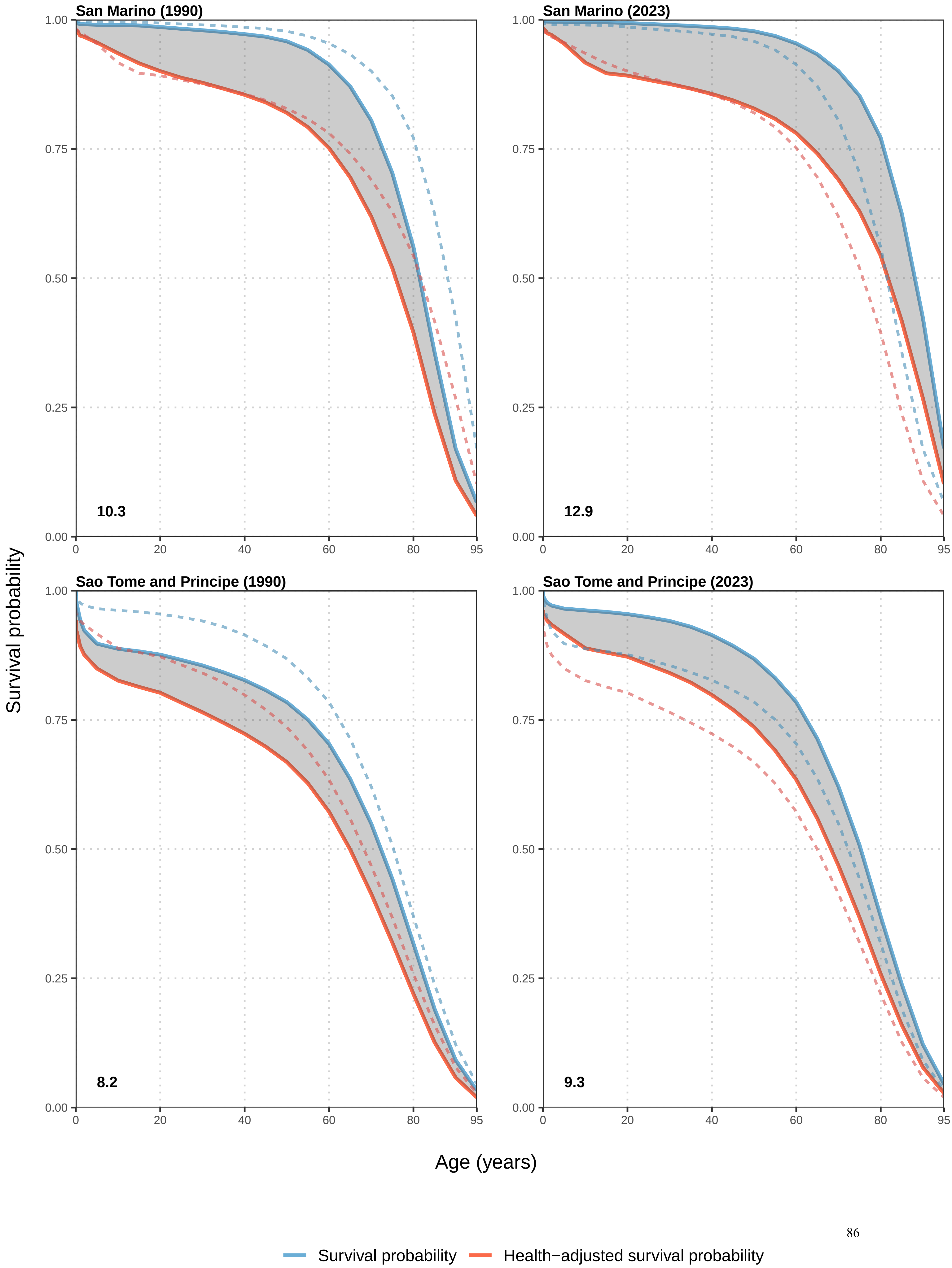

San Marino (1990)
San Marino (2023)
Sao Tome and Principe (1990)
Sao Tome and Principe (2023)
10.3
12.9
8.2
9.3
Survival probability
Age (years)
1.00
0.75
0.50
0.25
0.00
0
20
40
60
80
95
Survival probability
Health−adjusted survival probability

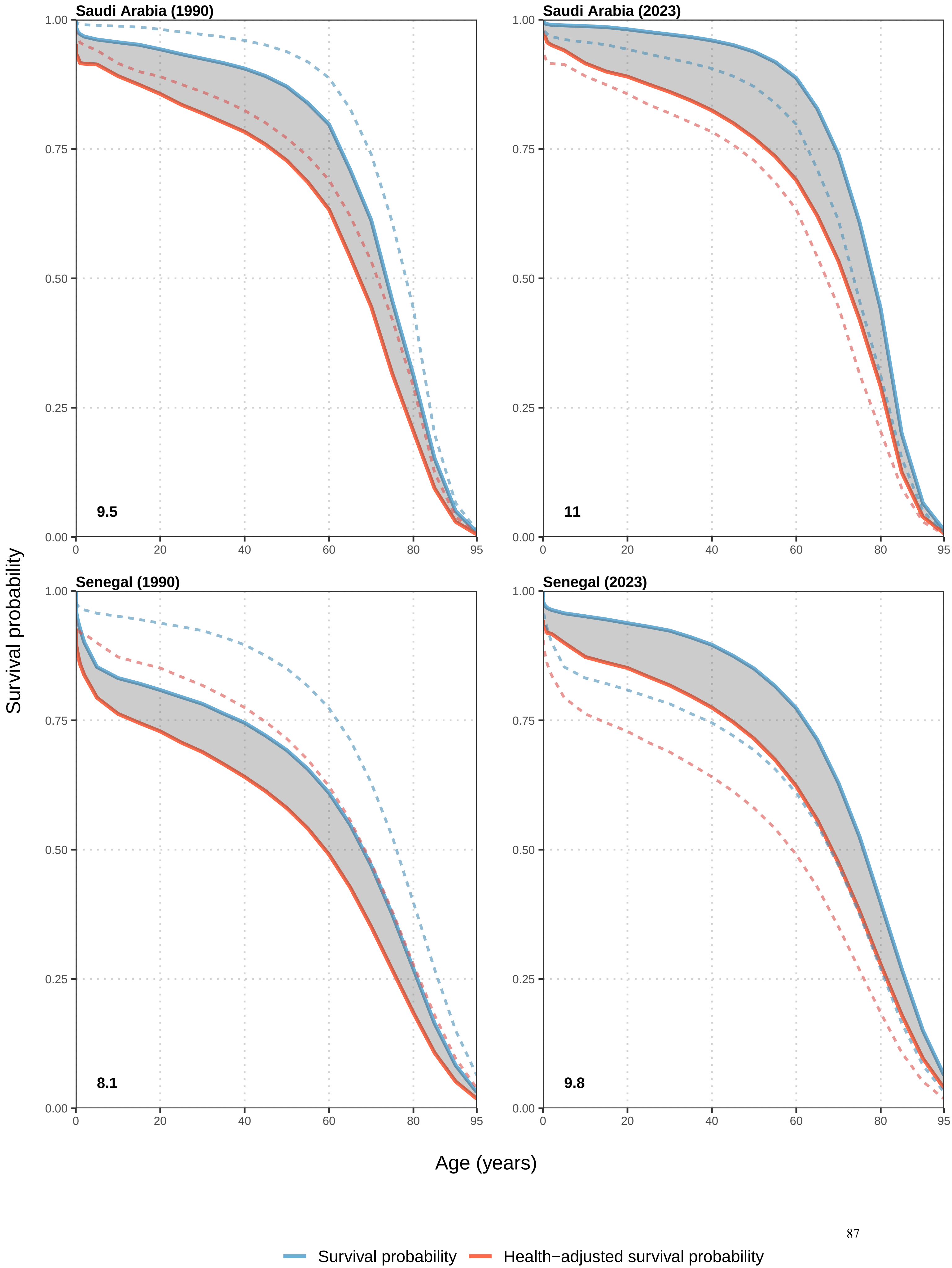
Saudi Arabia (1990)
Saudi Arabia (2023)
Senegal (1990)
Senegal (2023)
9.5
11
8.1
9.8
1.00
0.75
0.50
0.25
0.00
0
20
40
60
80
95
Survival probability
Age (years)
Survival probability
Health−adjusted survival probability

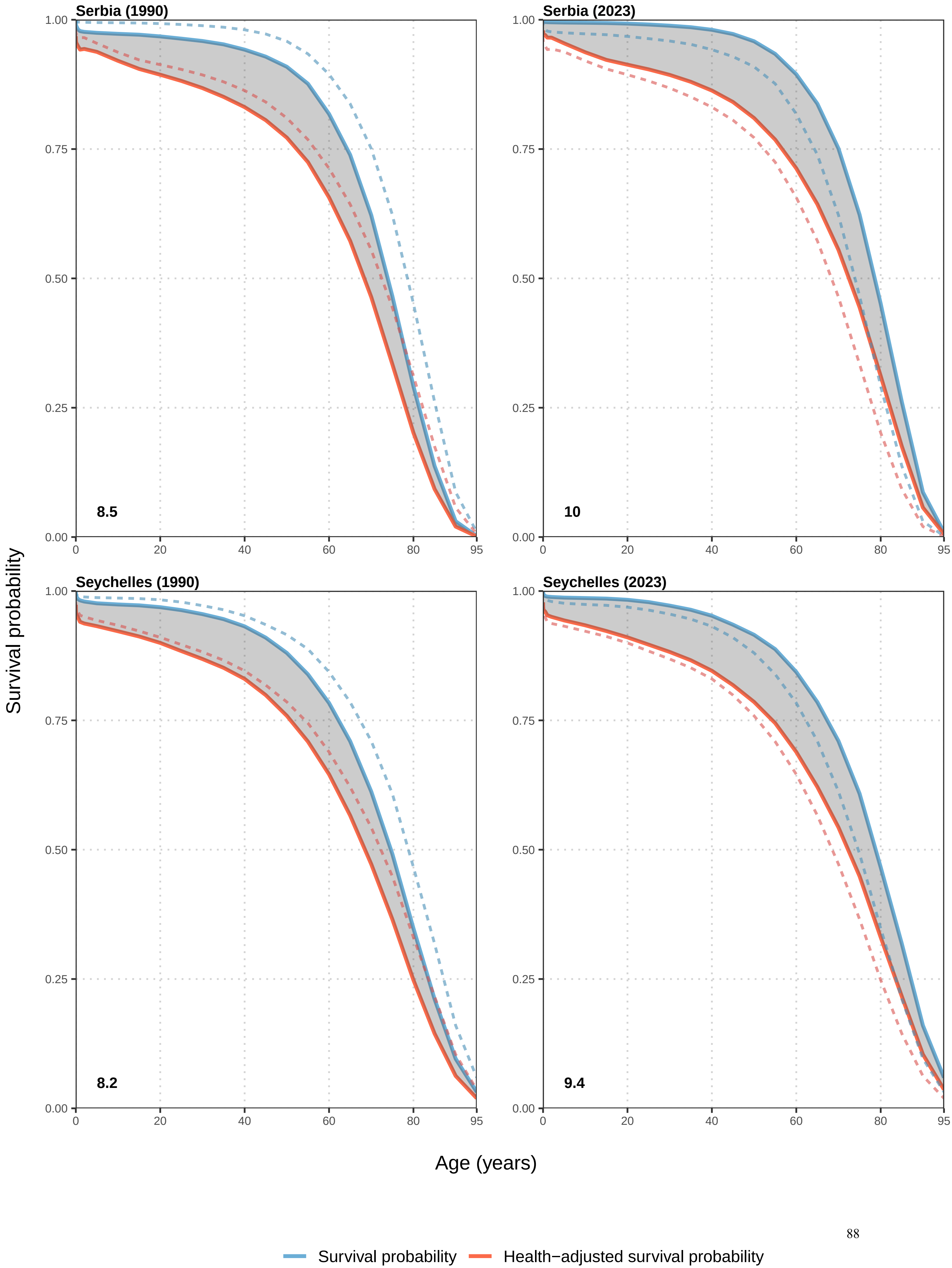
Serbia (1990)
8.5
Serbia (2023)
10
Seychelles (1990)
8.2
Seychelles (2023)
9.4
Survival probability
Age (years)
1.00
0.75
0.50
0.25
0.00
0
20
40
60
80
95
Survival probability
Health−adjusted survival probability

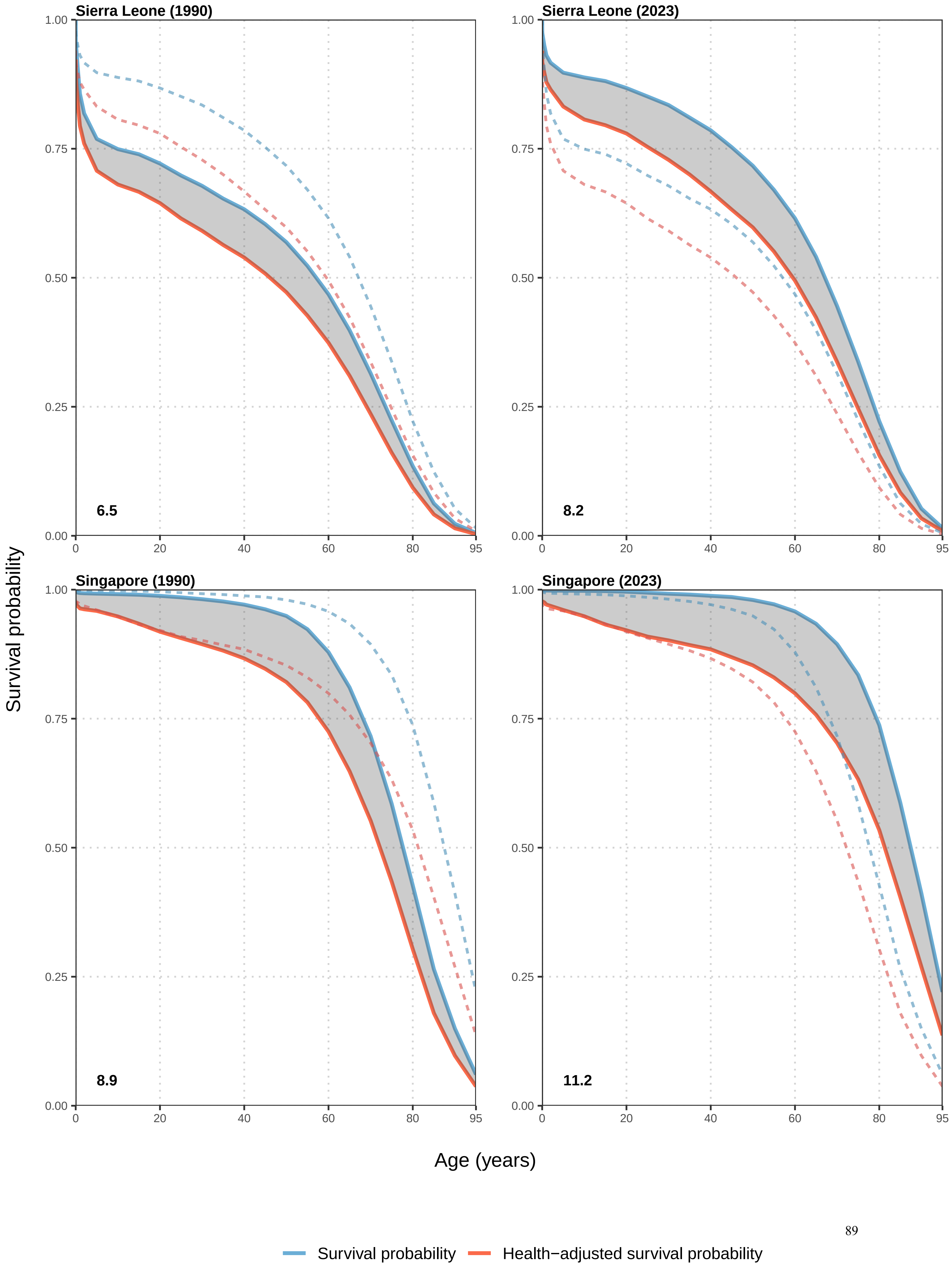
Sierra Leone (1990)
Sierra Leone (2023)
Singapore (1990)
Singapore (2023)
6.5
8.2
8.9
11.2
Survival probability
Age (years)
1.00
0.75
0.50
0.25
0.00
0
20
40
60
80
95
Survival probability
Health−adjusted survival probability

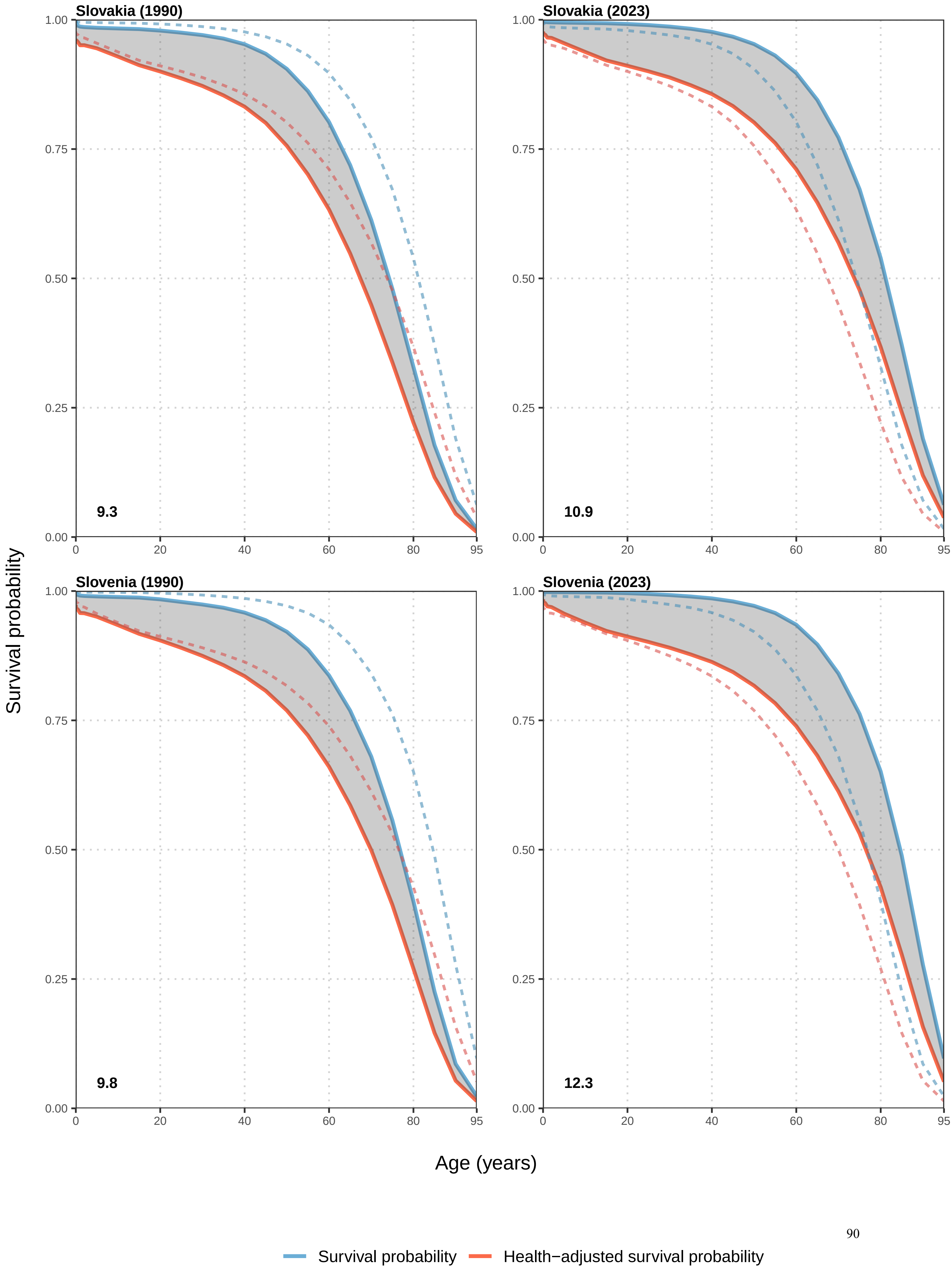

Slovakia (1990)
Slovakia (2023)
Slovenia (1990)
Slovenia (2023)
9.3
10.9
9.8
12.3
Survival probability
Age (years)
Survival probability
Health−adjusted survival probability

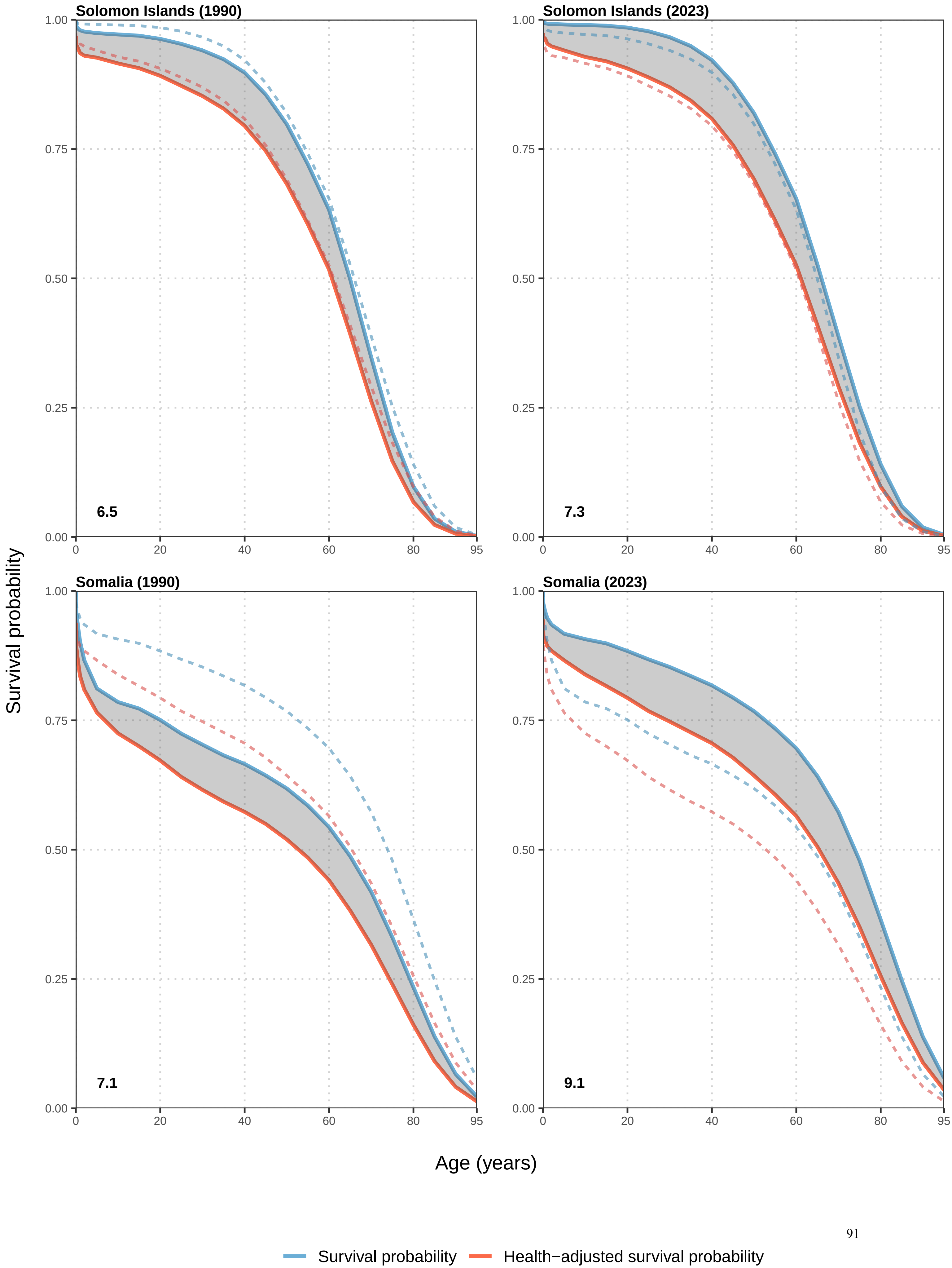

Solomon Islands (1990)
Solomon Islands (2023)
Somalia (1990)
Somalia (2023)
6.5
7.3
7.1
9.1
1.00
0.75
0.50
0.25
0.00
0
20
40
60
80
95
Survival probability
Age (years)
Survival probability
Health−adjusted survival probability

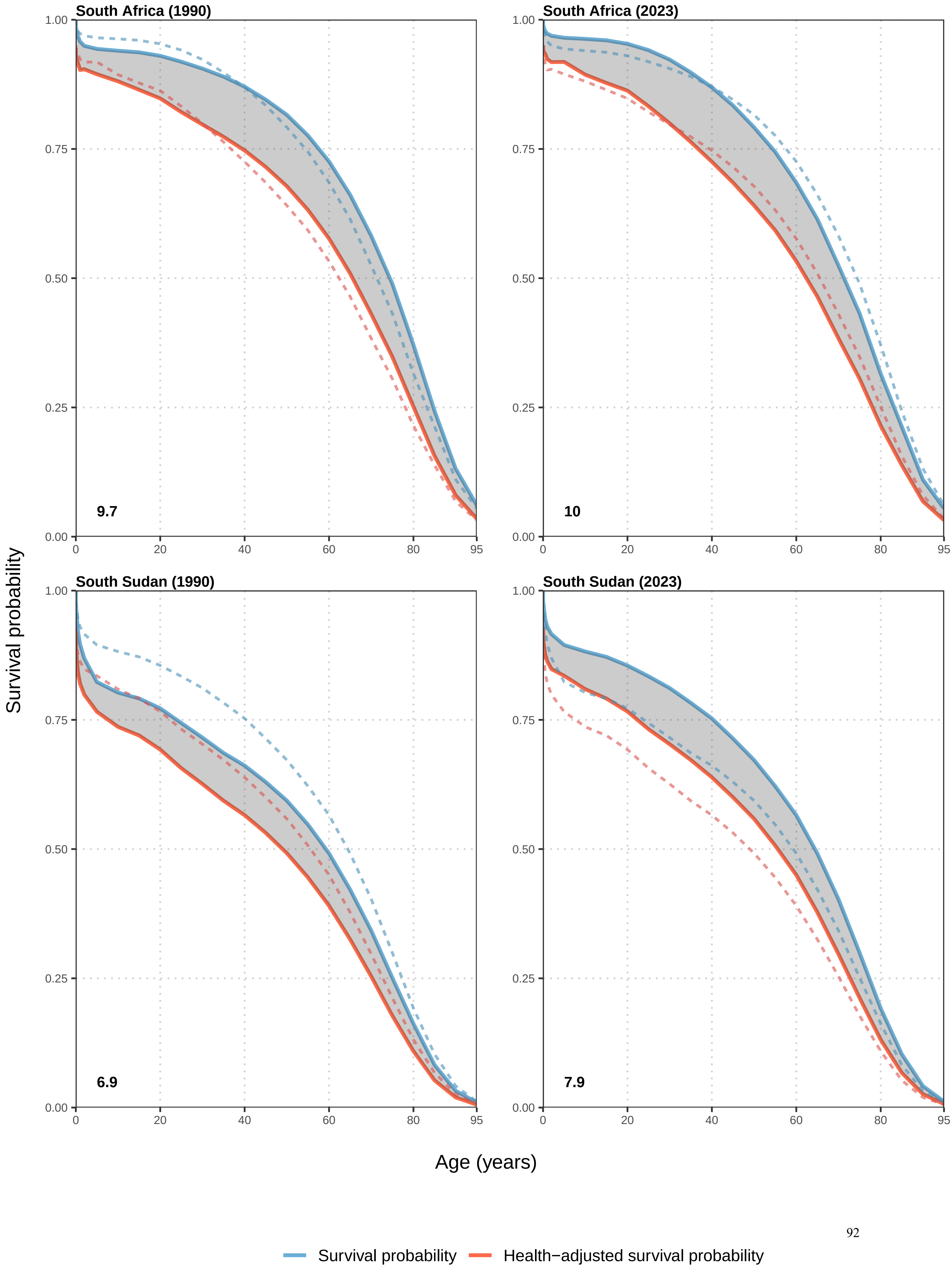

South Africa (1990)
9.7
South Africa (2023)
10
South Sudan (1990)
6.9
South Sudan (2023)
7.9
Survival probability
Age (years)
1.00
0.75
0.50
0.25
0.00
0
20
40
60
80
95
Survival probability
Health−adjusted survival probability

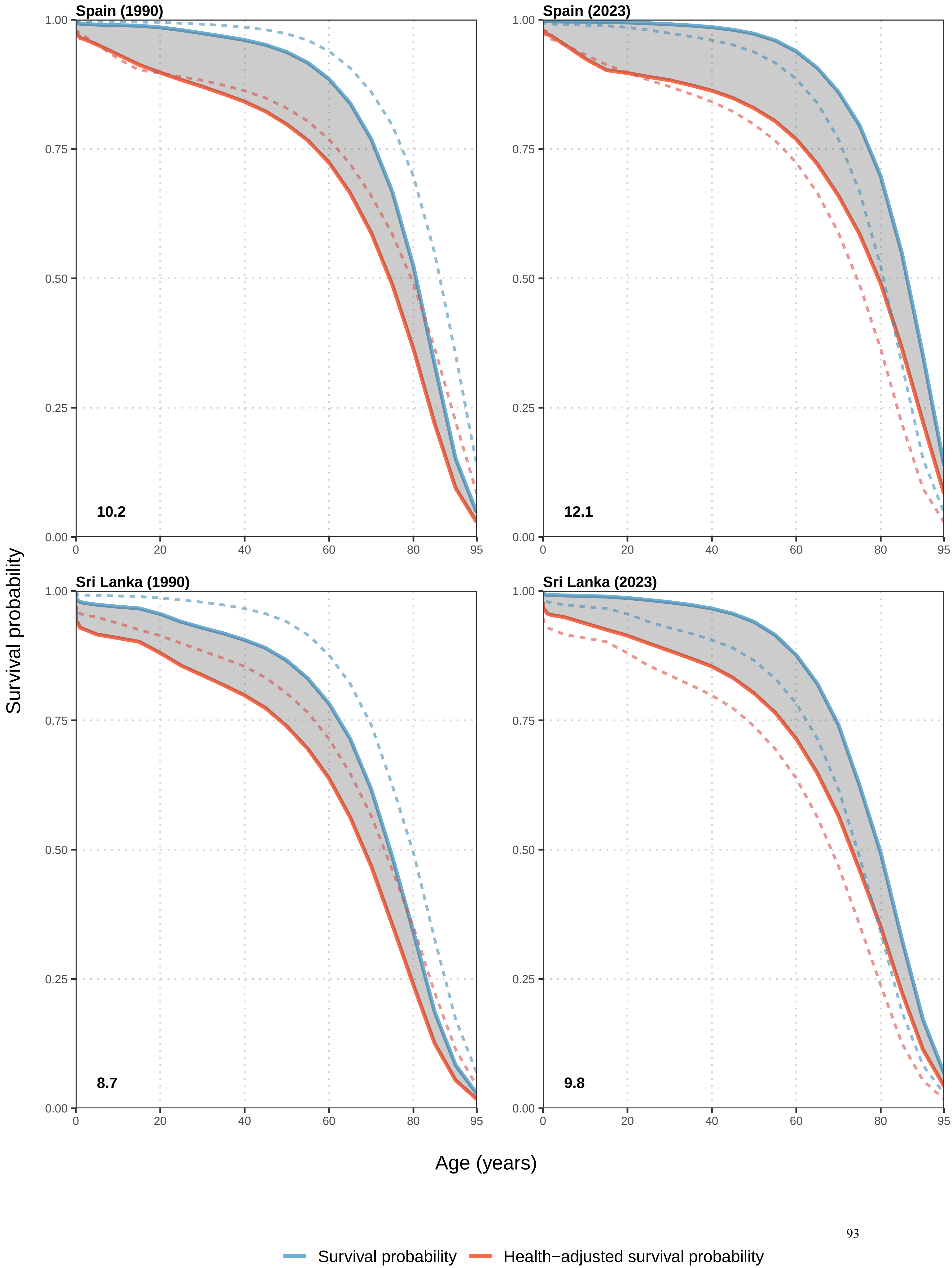
Spain (1990)
10.2
Spain (2023)
12.1
Sri Lanka (1990)
8.7
Sri Lanka (2023)
9.8
Survival probability
Age (years)
Survival probability
Health−adjusted survival probability

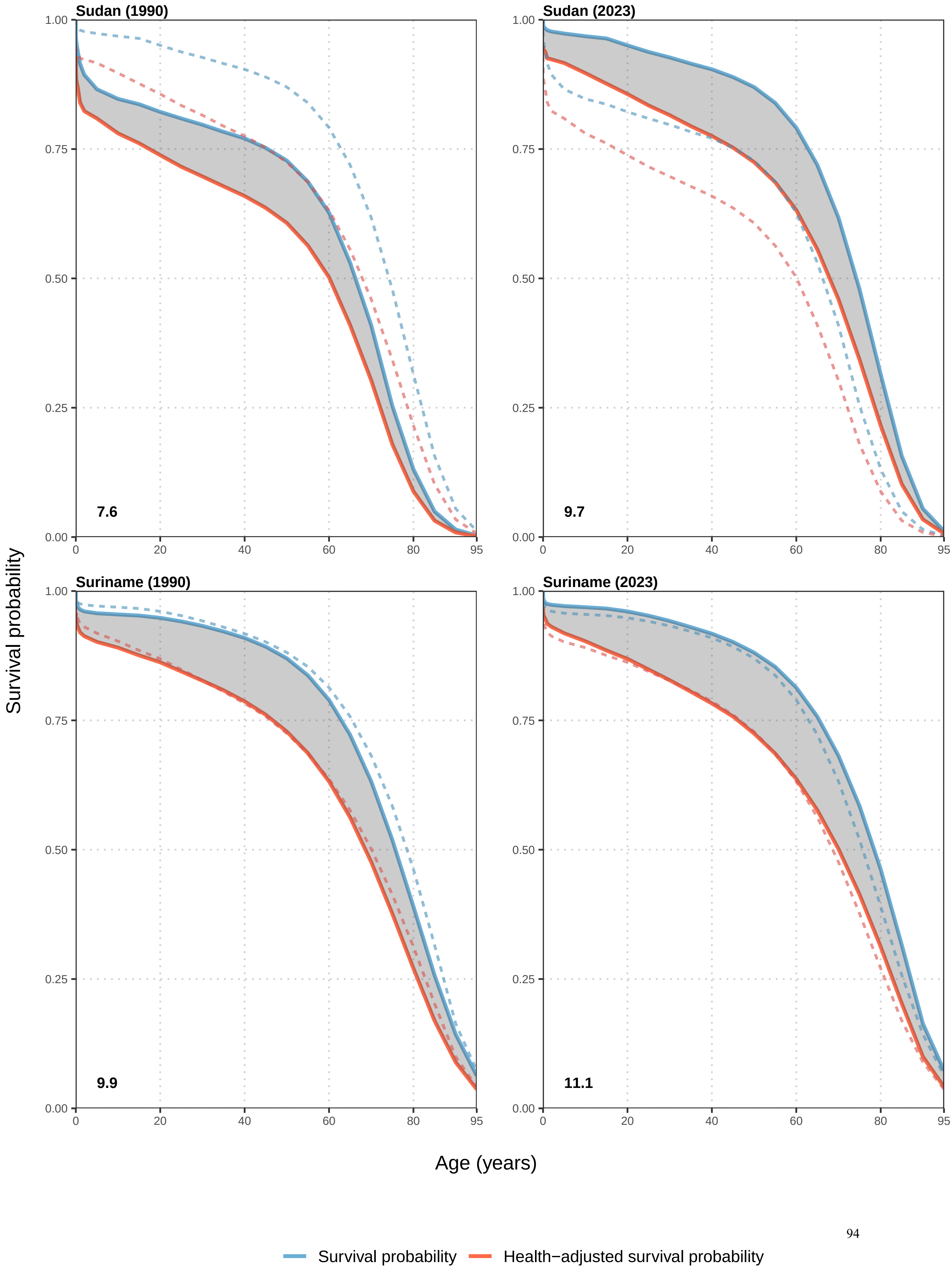

Sudan (1990)
7.6
Sudan (2023)
9.7
Suriname (1990)
9.9
Suriname (2023)
11.1
Survival probability
Age (years)
1.00
0.75
0.50
0.25
0.00
0
20
40
60
80
95
Survival probability
Health−adjusted survival probability

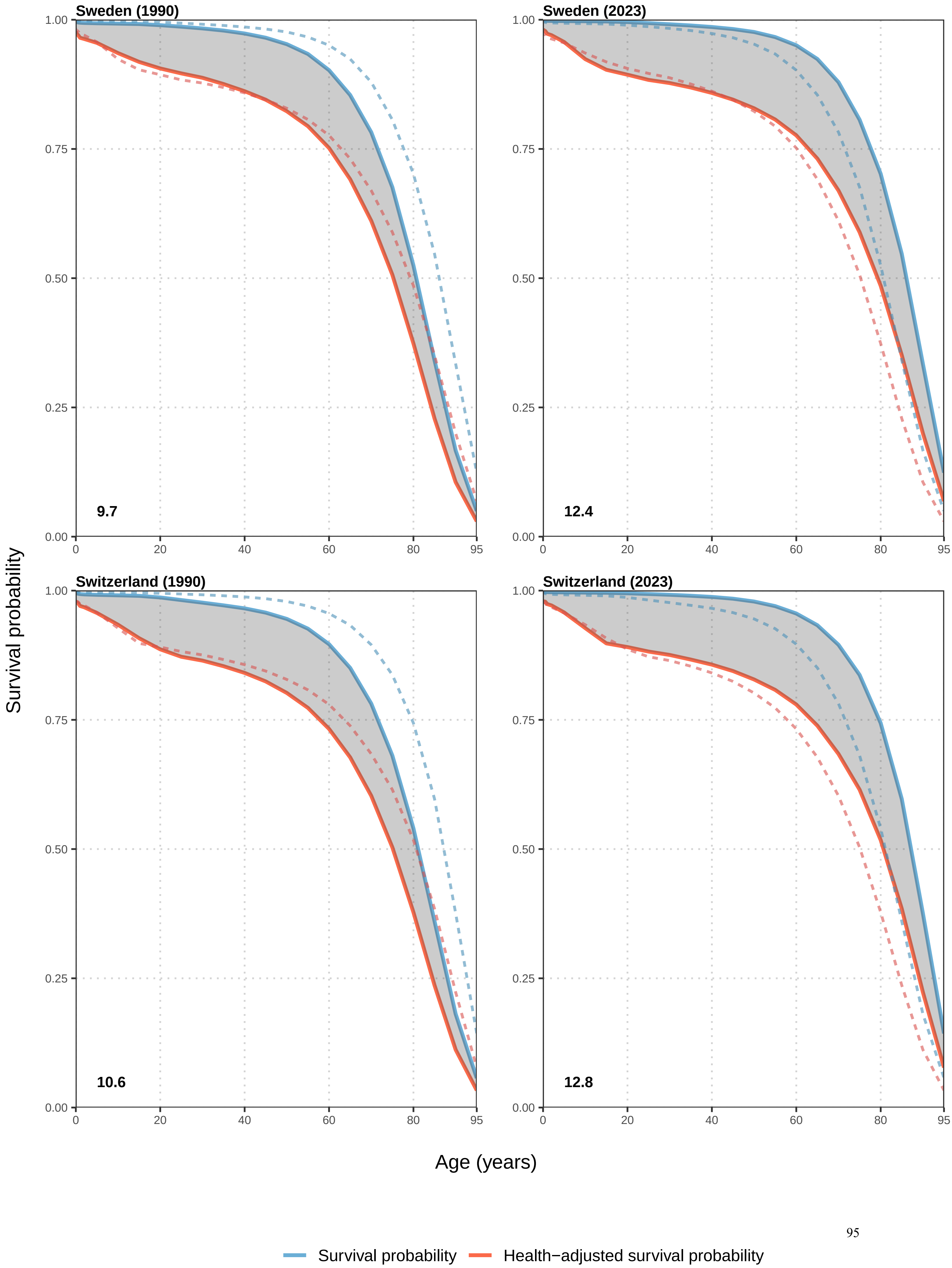
Sweden (1990)
9.7
Sweden (2023)
12.4
Switzerland (1990)
10.6
Switzerland (2023)
12.8
Survival probability
Age (years)
Survival probability
Health−adjusted survival probability

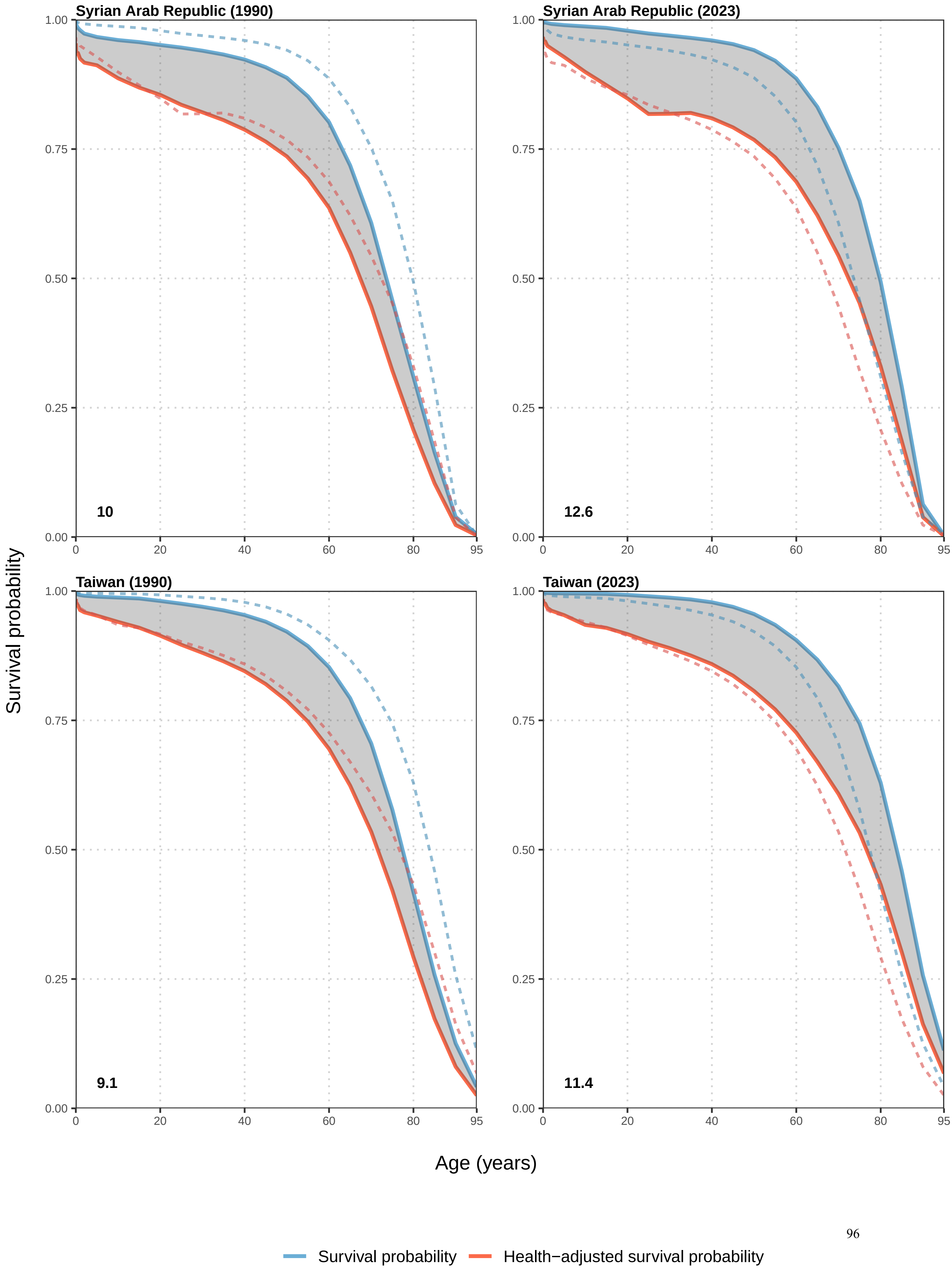
Syrian Arab Republic (1990)
10
Syrian Arab Republic (2023)
12.6
Taiwan (1990)
9.1
Taiwan (2023)
11.4
1.00
0.75
0.50
0.25
0.00
0
20
40
60
80
95
Survival probability
Age (years)
Survival probability
Health−adjusted survival probability

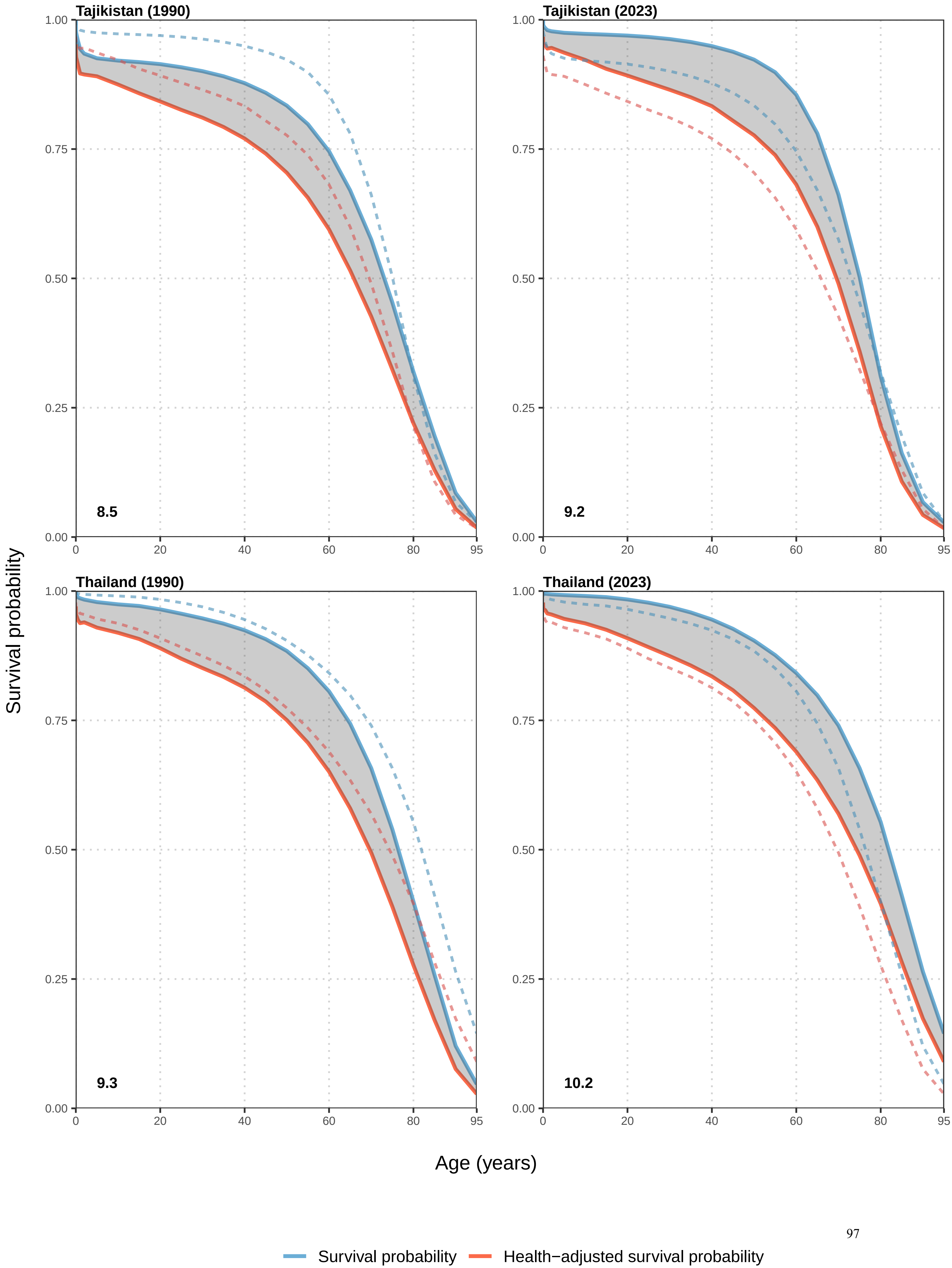

Tajikistan (1990)
Tajikistan (2023)
Thailand (1990)
Thailand (2023)
8.5
9.2
9.3
10.2
Survival probability
Age (years)
1.00
0.75
0.50
0.25
0.00
0
20
40
60
80
95
Survival probability
Health−adjusted survival probability

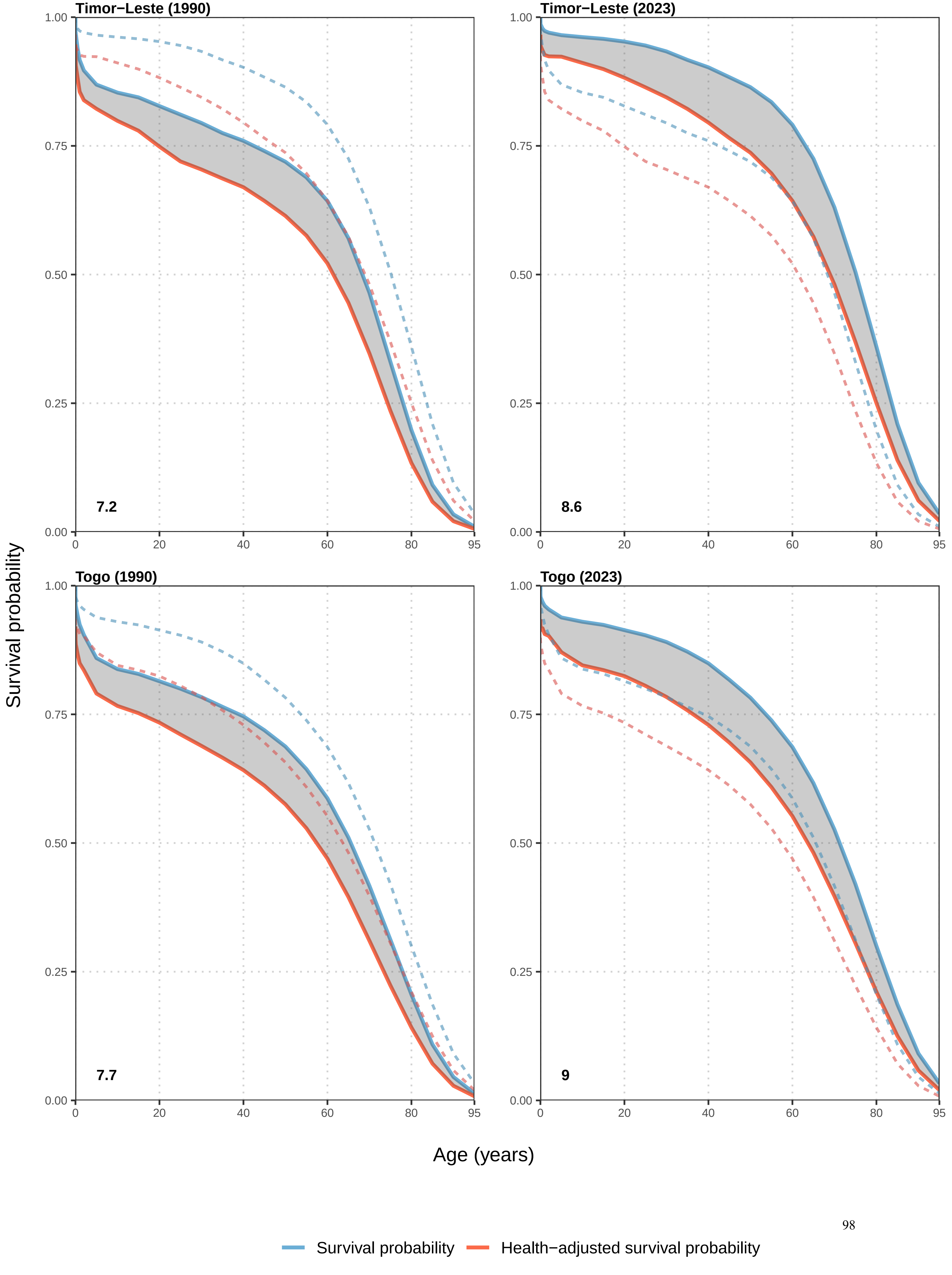
Timor−Leste (1990)
Timor−Leste (2023)
Togo (1990)
Togo (2023)
7.2
8.6
7.7
9
Survival probability
Age (years)
1.00
0.75
0.50
0.25
0.00
0
20
40
60
80
95
Survival probability
Health−adjusted survival probability

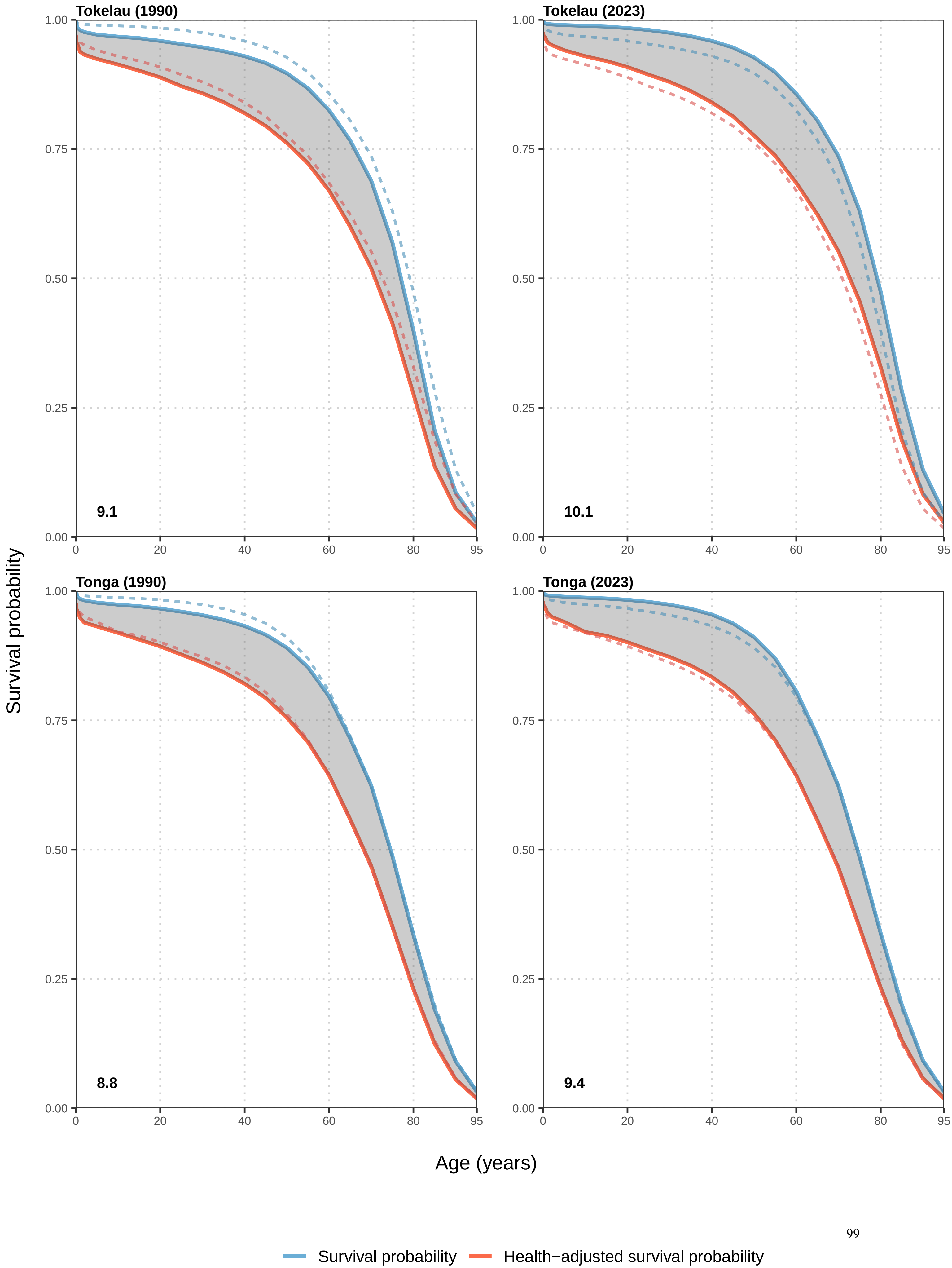
Tokelau (1990)
Tokelau (2023)
Tonga (1990)
Tonga (2023)
9.1
10.1
8.8
9.4
Survival probability
Age (years)
0.00
0.25
0.50
0.75
1.00
0
20
40
60
80
95
Survival probability
Health−adjusted survival probability

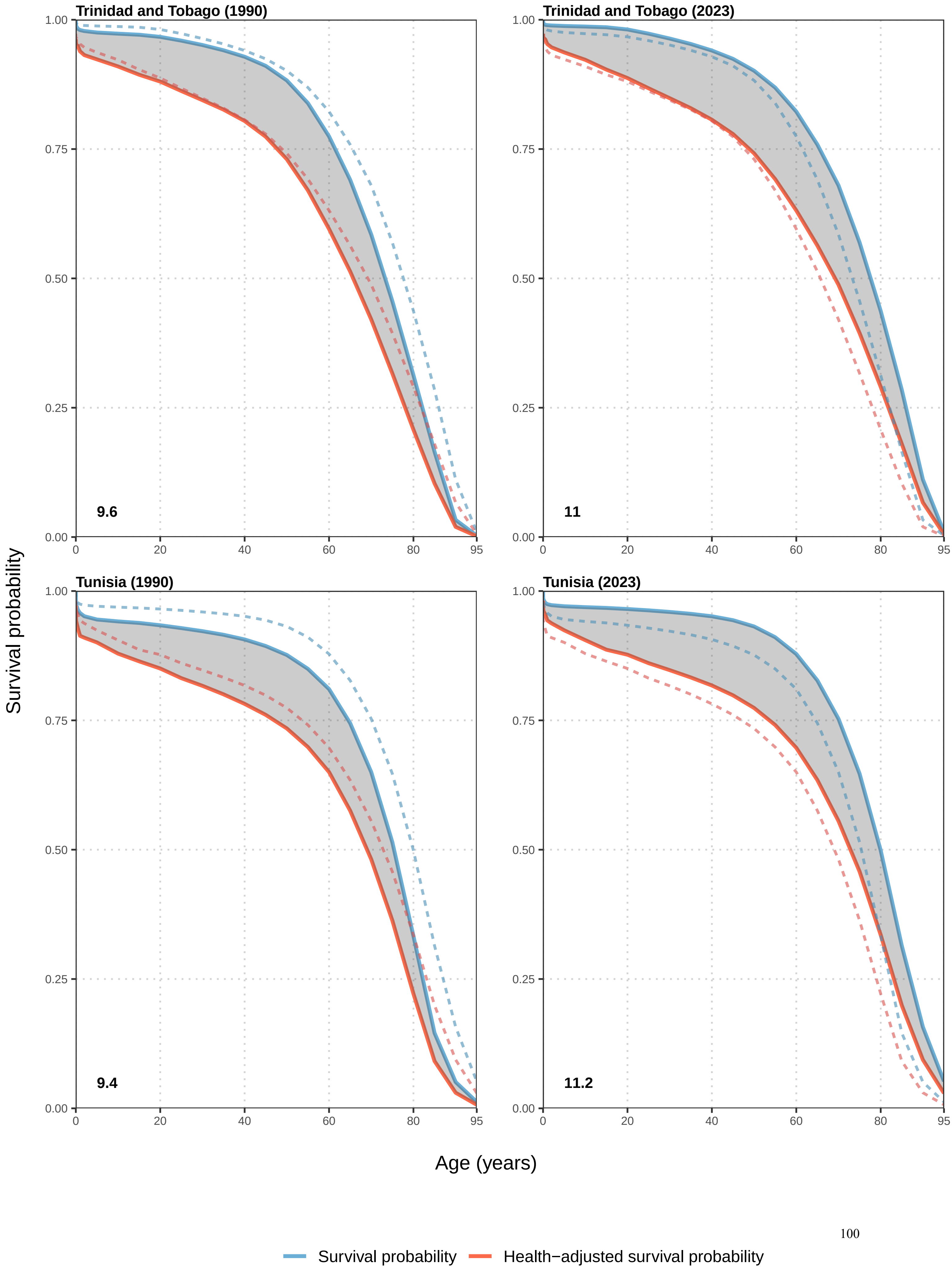
Trinidad and Tobago (1990)
Trinidad and Tobago (2023)
Tunisia (1990)
Tunisia (2023)
9.6
11
9.4
11.2
Survival probability
Age (years)
1.00
0.75
0.50
0.25
0.00
0
20
40
60
80
95
Survival probability
Health−adjusted survival probability

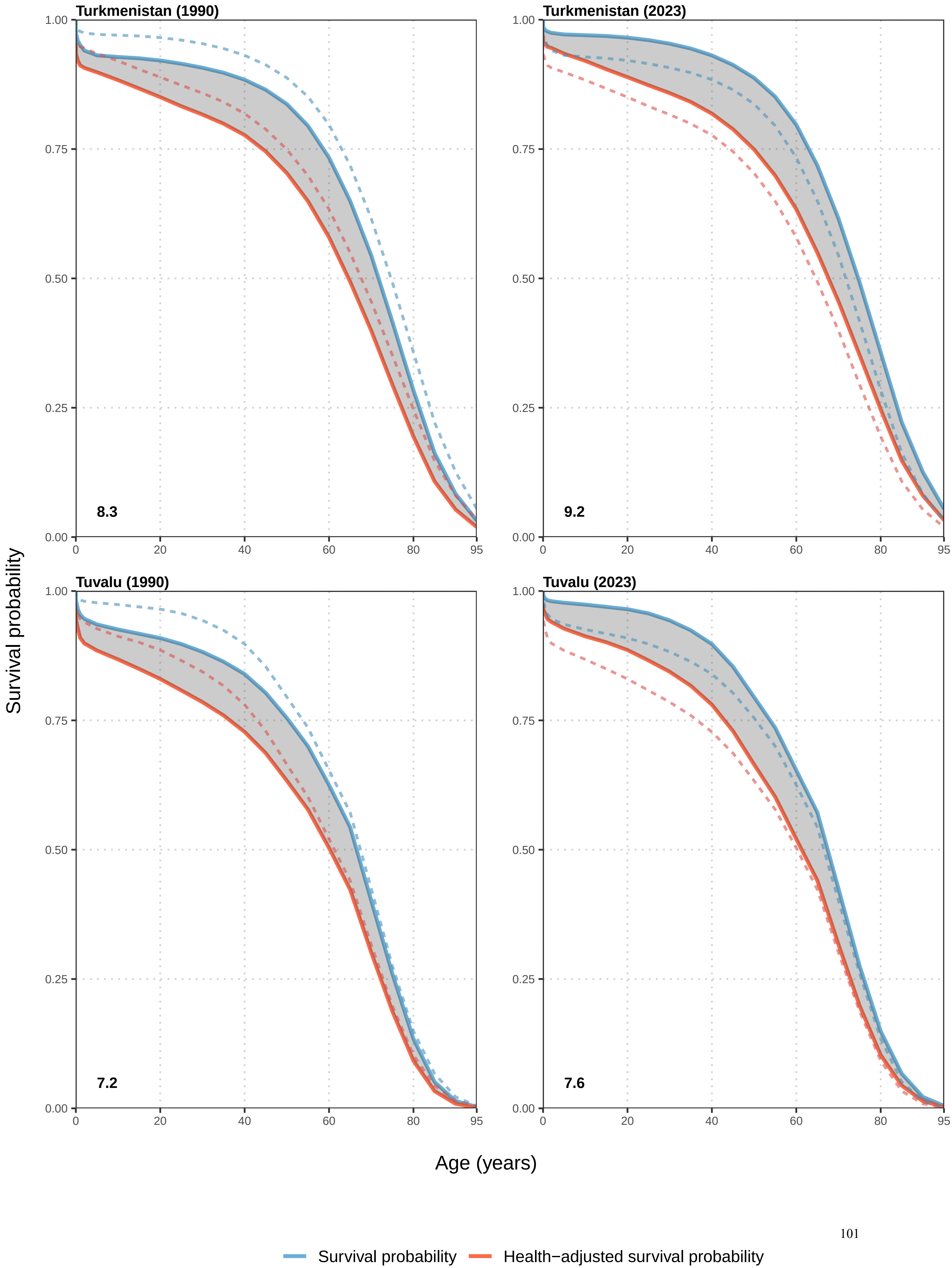
Turkmenistan (1990)
8.3
Turkmenistan (2023)
9.2
Tuvalu (1990)
7.2
Tuvalu (2023)
7.6
Survival probability
Age (years)
1.00
0.75
0.50
0.25
0.00
0
20
40
60
80
95
Survival probability
Health−adjusted survival probability

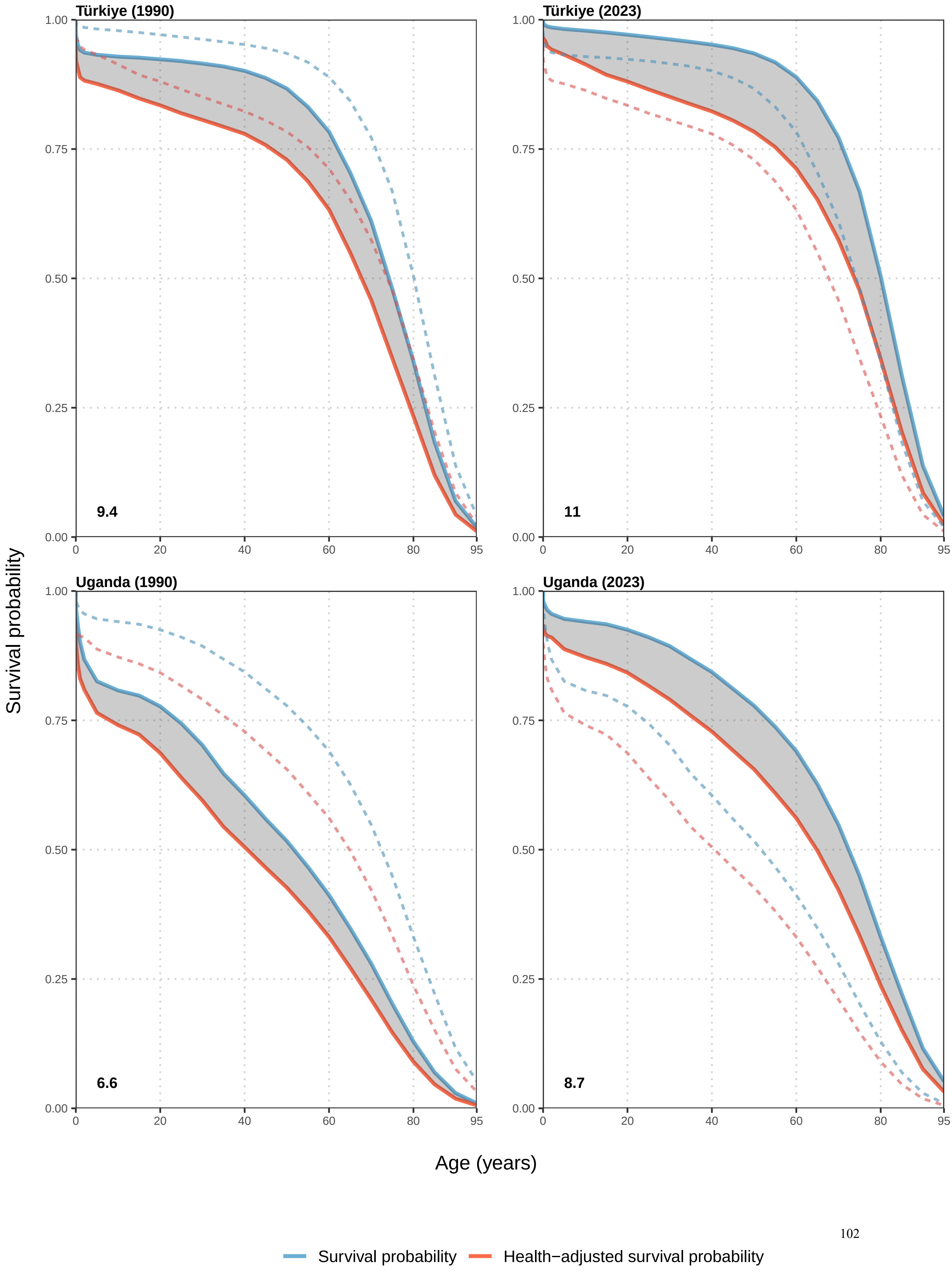
Türkiye (1990)
Türkiye (2023)
Uganda (1990)
Uganda (2023)
9.4
11
6.6
8.7
Survival probability
Age (years)
1.00
0.75
0.50
0.25
0.00
0
20
40
60
80
95
Survival probability
Health−adjusted survival probability

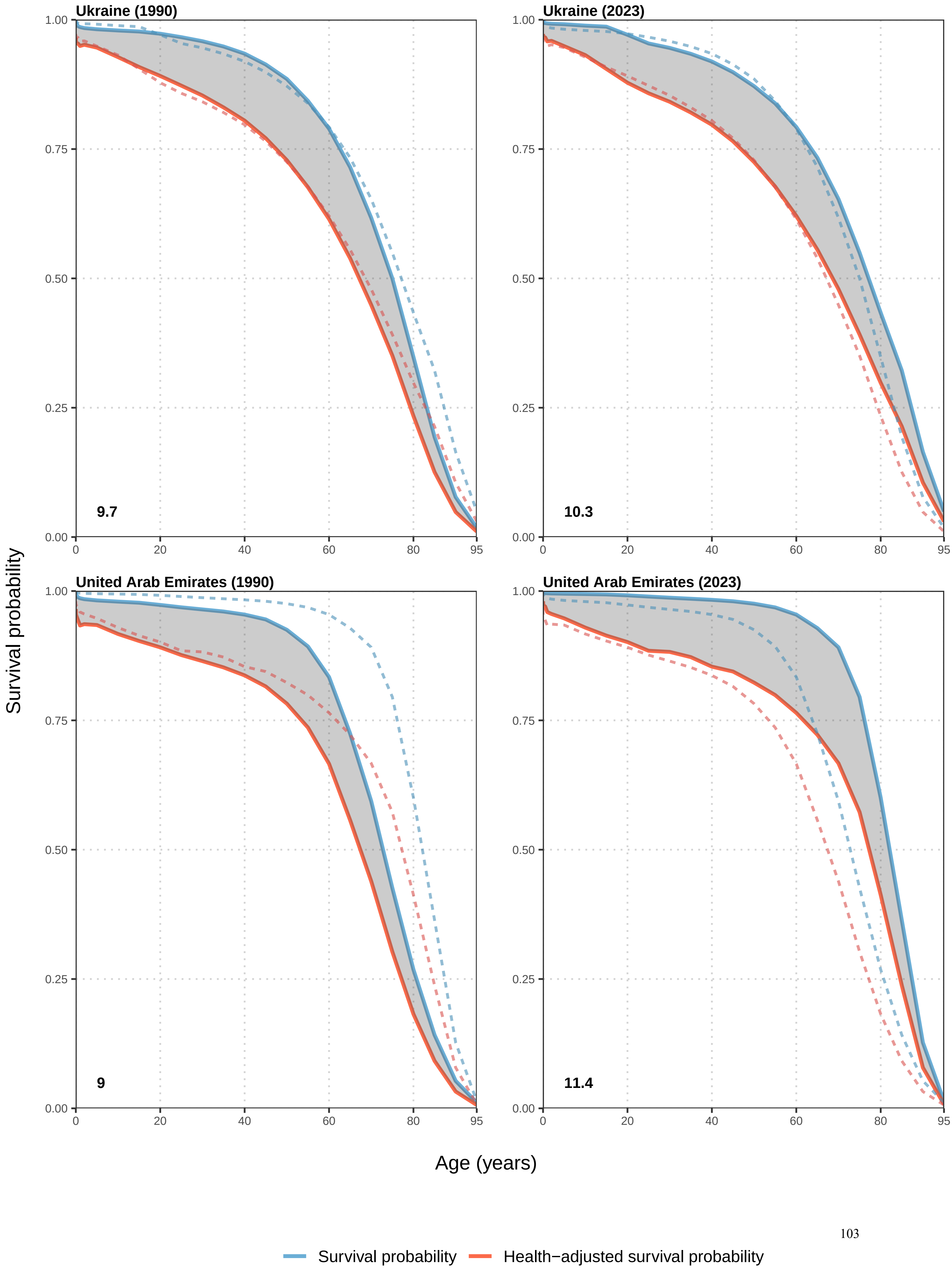
Ukraine (1990)
Ukraine (2023)
United Arab Emirates (1990)
United Arab Emirates (2023)
9.7
10.3
9
11.4
1.00
0.75
0.50
0.25
0.00
0
20
40
60
80
95
Survival probability
Age (years)
Survival probability
Health−adjusted survival probability

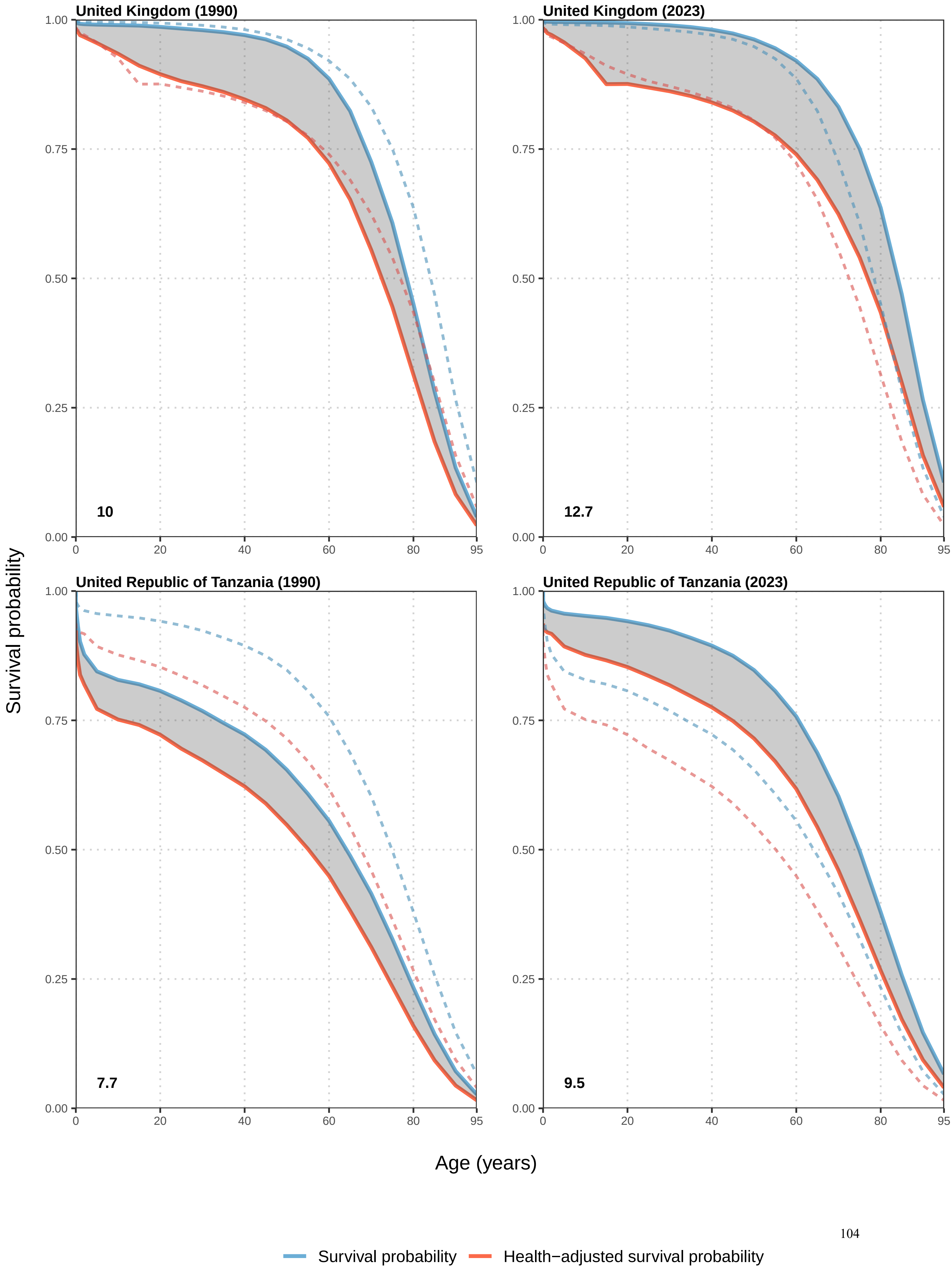

United Kingdom (1990)
United Kingdom (2023)
United Republic of Tanzania (1990)
United Republic of Tanzania (2023)
10
12.7
7.7
9.5
Survival probability
Age (years)
1.00
0.75
0.50
0.25
0.00
0
20
40
60
80
95
Survival probability
Health−adjusted survival probability

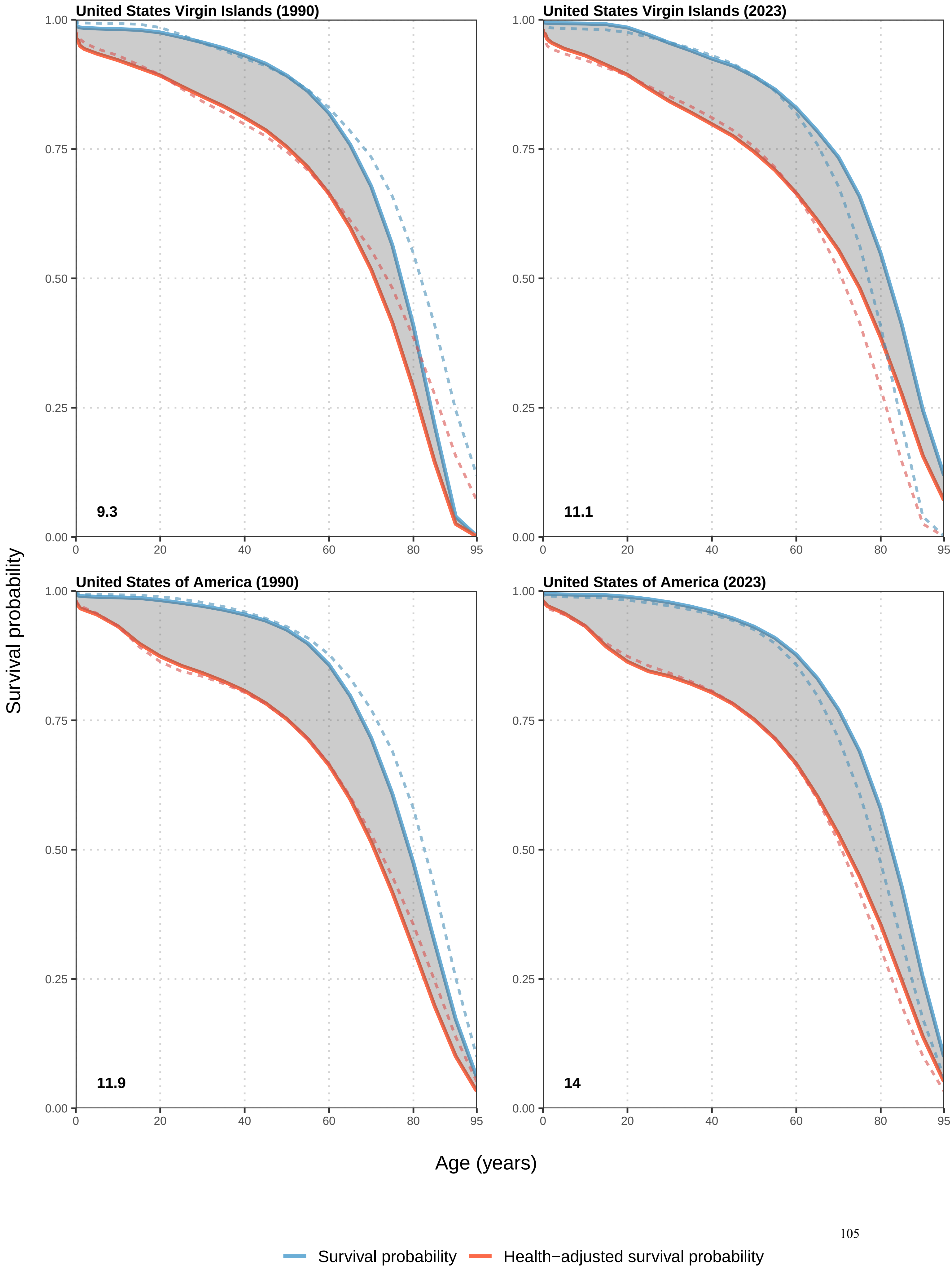
United States Virgin Islands (1990)
United States Virgin Islands (2023)
United States of America (1990)
United States of America (2023)
9.3
11.1
11.9
14
Survival probability
Age (years)
0.00
0.25
0.50
0.75
1.00
0
20
40
60
80
95
Survival probability
Health−adjusted survival probability

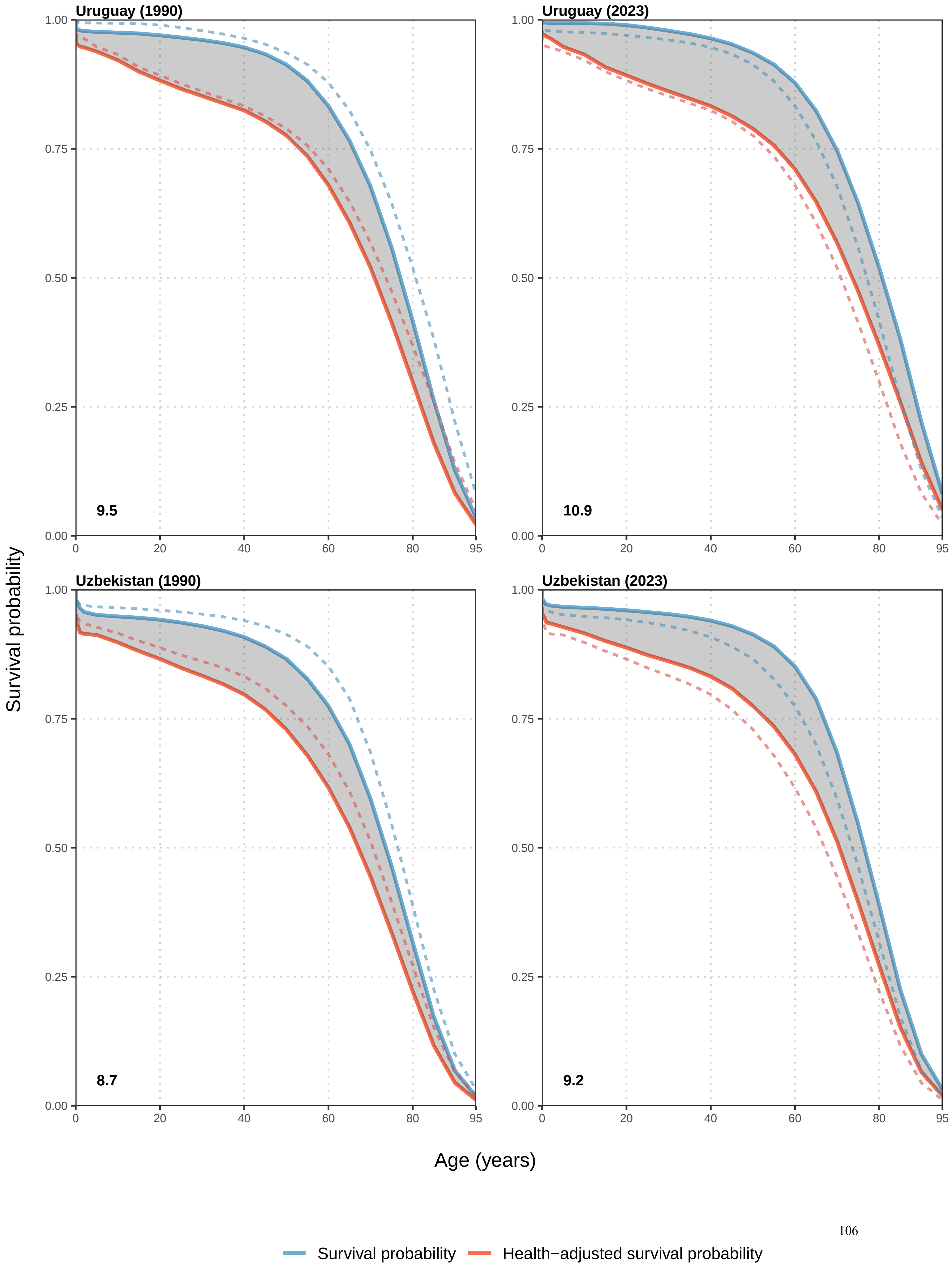

Uruguay (1990)
9.5
Uruguay (2023)
10.9
Uzbekistan (1990)
8.7
Uzbekistan (2023)
9.2
1.00
0.75
0.50
0.25
0.00
0
20
40
60
80
95
Survival probability
Age (years)
Survival probability
Health−adjusted survival probability

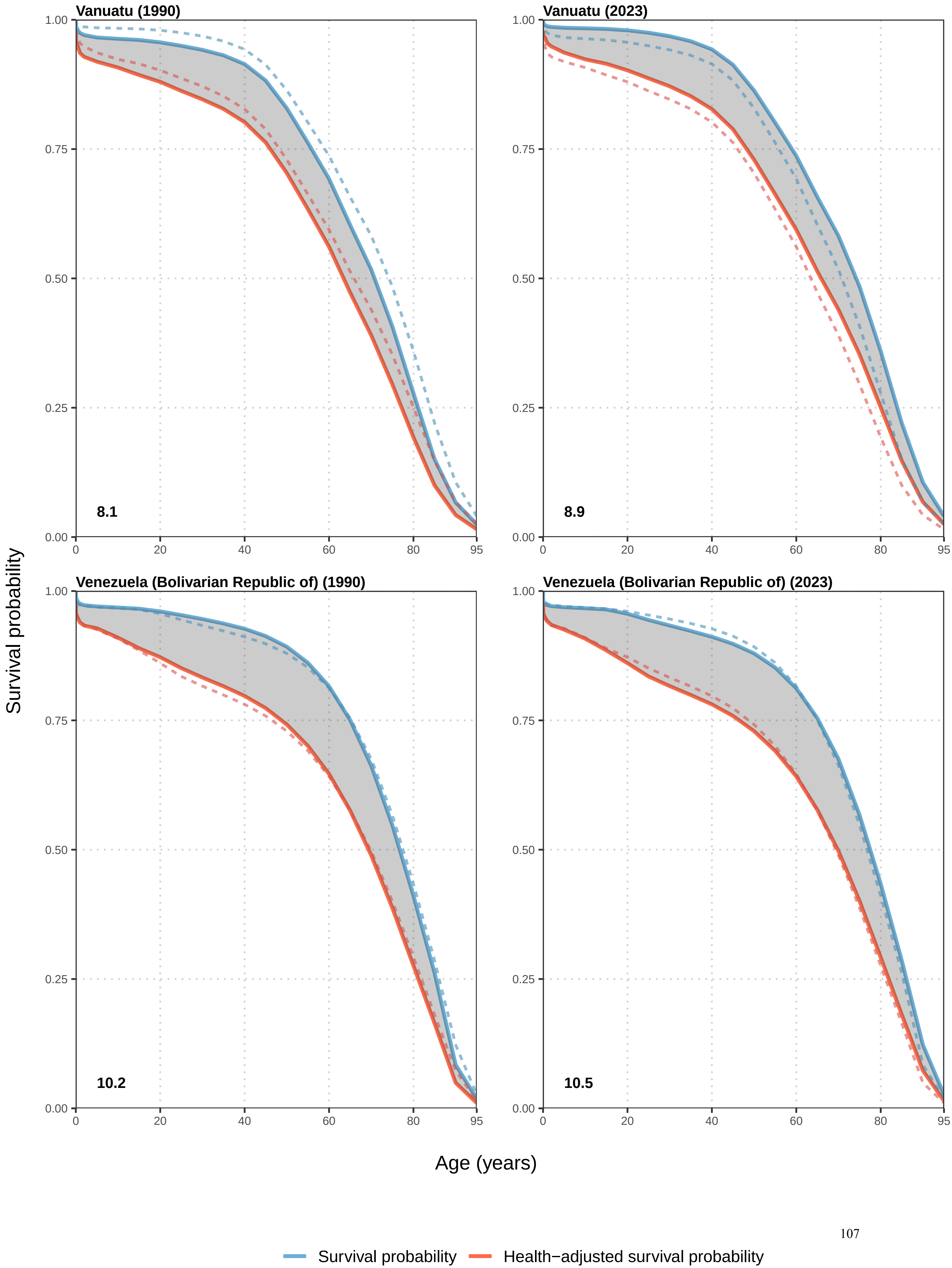
Vanuatu (1990)
8.1
Vanuatu (2023)
8.9
Venezuela (Bolivarian Republic of) (1990)
10.2
Venezuela (Bolivarian Republic of) (2023)
10.5
Survival probability
Age (years)
1.00
0.75
0.50
0.25
0.00
0
20
40
60
80
95
Survival probability
Health−adjusted survival probability

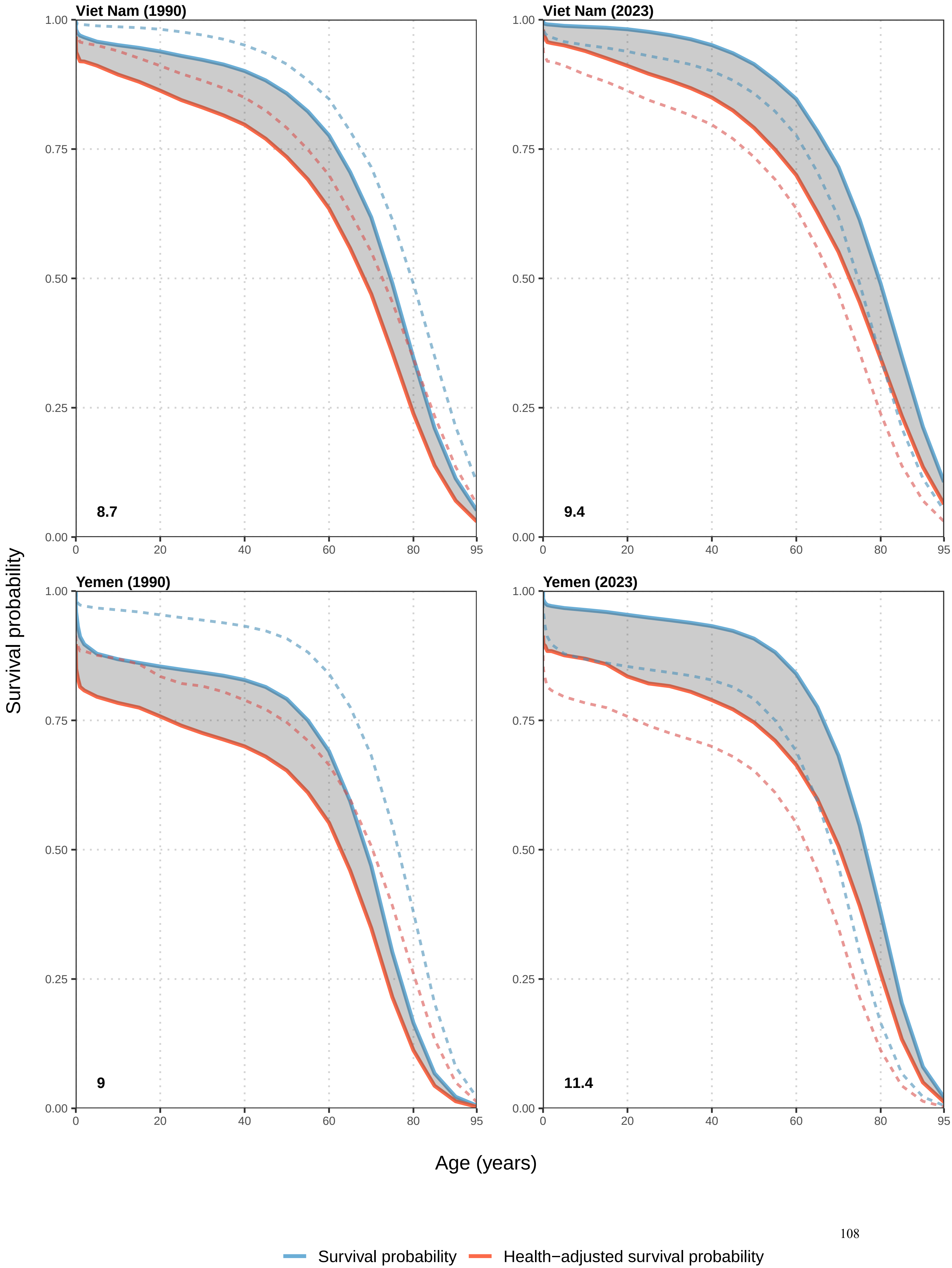

Viet Nam (1990)
Viet Nam (2023)
Yemen (1990)
Yemen (2023)
8.7
9.4
9
11.4
Survival probability
Age (years)
Survival probability
Health−adjusted survival probability

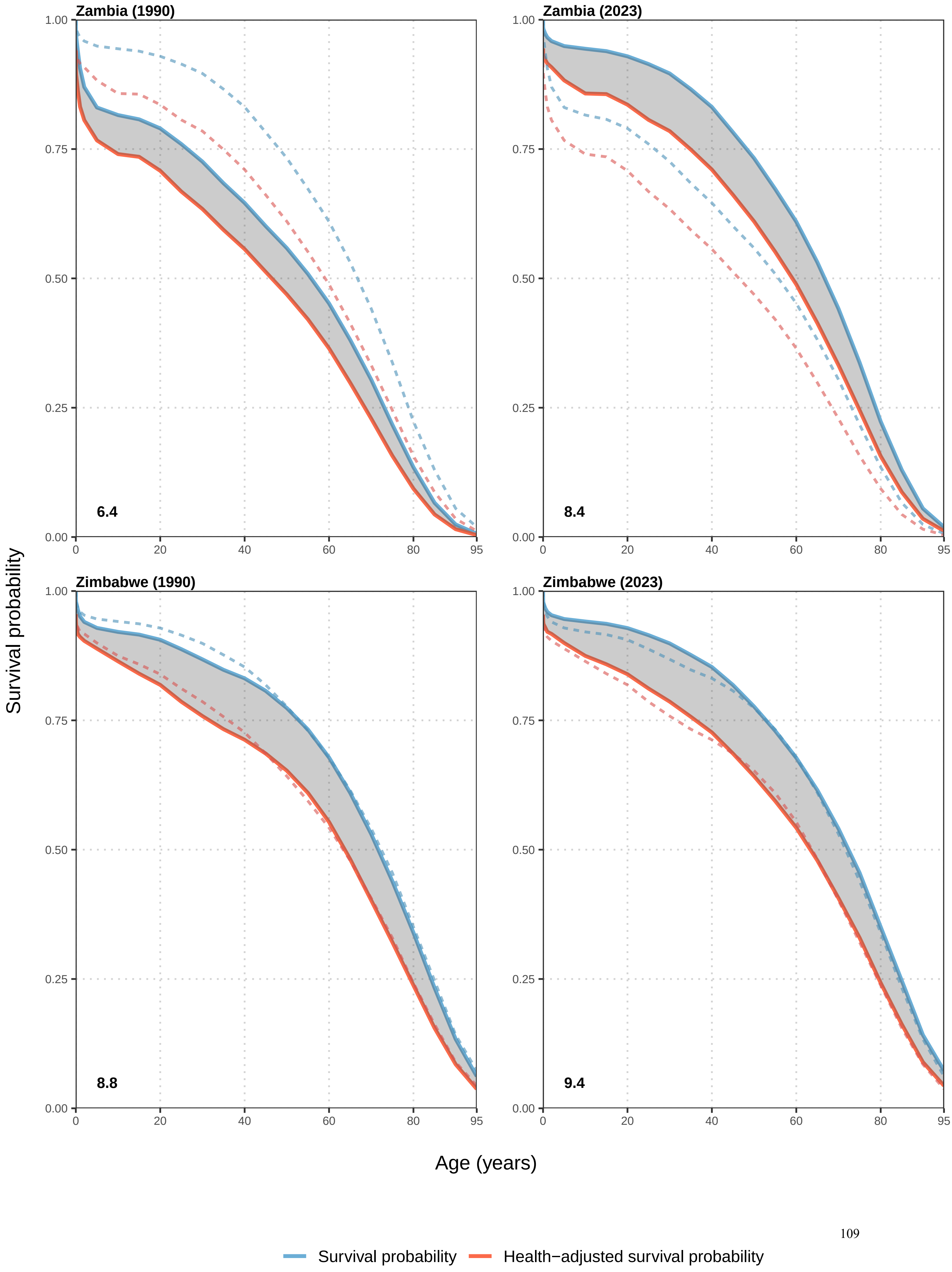
Zambia (1990)
Zambia (2023)
Zimbabwe (1990)
Zimbabwe (2023)
6.4
8.4
8.8
9.4
1.00
0.75
0.50
0.25
0.00
0
20
40
60
80
95
Survival probability
Age (years)
Survival probability
Health−adjusted survival probability
